# Using intervention mapping to develop a culturally appropriate intervention to prevent childhood obesity: the HAPPY (Healthy and Active Parenting Programme for Early Years) study

**DOI:** 10.1186/1479-5868-10-142

**Published:** 2013-12-28

**Authors:** Natalie J Taylor, Pinki Sahota, Judith Sargent, Sally Barber, Jackie Loach, Gemma Louch, John Wright

**Affiliations:** 1 Bradford Institute of Health Research, Bradford Teaching Hospitals NHS Trust, Bradford BD9 6RJ, UK; 2 Australian Institute of Health Innovation Faculty of Medicine, University of New South Wales, SYDNEY NSW 2052, UK; 3 Nutrition and Dietetics, Leeds Metropolitan University, Leeds LS1 3HE, UK; 4 School of Health and Wellbeing Faculty of Health and Social Sciences, Leeds Metropolitan University City Campus, Calverley Street, Leeds LS1 3HE, UK

**Keywords:** Childhood obesity prevention, Intervention mapping, Behaviour change, Cultural adaptation, Diet, Physical activity, Parenting, Theoretical domains framework

## Abstract

**Introduction:**

Interventions that make extensive use of theory tend to have larger effects on behaviour. The Intervention Mapping (IM) framework incorporates theory into intervention design, implementation and evaluation, and was applied to the development of a community-based childhood obesity prevention intervention for a multi-ethnic population.

**Methods:**

IM was applied as follows: 1) Needs assessment of the community and culture; consideration of evidence-base, policy and practice; 2) Identification of desired outcomes and change objectives following identification of barriers to behaviour change mapped alongside psychological determinants (e.g. knowledge, self-efficacy, intention); 3) Selection of theory-based methods and practical applications to address barriers to behaviour change (e.g., strategies for responsive feeding); 4) Design of the intervention by developing evidence-based interactive activities and resources (e.g., visual aids to show babies stomach size). The activities were integrated into an existing parenting programme; 5) Adoption and implementation: parenting practitioners were trained by healthcare professionals to deliver the programme within Children Centres.

**Results:**

HAPPY (Healthy and Active Parenting Programme for Early Years) is aimed at overweight and obese pregnant women (BMI > 25); consists of 12 × 2.5 hr. sessions (6 ante-natal from 24 weeks; 6 postnatal up to 9 months); it addresses mother’s diet and physical activity, breast or bottle feeding, infant diet and parental feeding practices, and infant physical activity.

**Conclusion:**

We have demonstrated that IM is a feasible and helpful method for providing an evidence based and theoretical structure to a complex health behaviour change intervention. The next stage will be to assess the impact of the intervention on behaviour change and clinical factors associated with childhood obesity. The HAPPY programme is currently being tested as part of a randomised controlled feasibility trial.

## Background

Childhood obesity and its attendant co-morbidities is one of the most prevalent threats to global public health [1], with higher prevalence observed in children from low income households and those of South Asian origin [2]. Despite evidence for growth trajectories in children indicating that the risk of childhood obesity starts in the first few months of life [3], there is limited evidence of interventions targeting 0-3 year old children [4]. Although there has been a recent focus on interventions in infancy [5-7] which have shown some positive effects on feeding behaviours and weight outcomes, most are from Australia and therefore have limited generalizability to the UK [8,9]. Given that 'parents are receptive to and capable of some behavioural changes that may promote healthy weight in their young children’ [10]; p.337, it has been recommended that parenting programme components (parenting skills and practices) are incorporated into childhood obesity prevention programmes [11,12].

Ineffective childhood obesity interventions may be associated with inadequate intervention design. Generally, intervention programmes often fail to report on factors such as a rationale, development, theoretical basis, exact content, and method of implementation [9]. This lack of transparency may be forcing researchers and practitioners to 'guess’ how interventions have been developed, what works and why. Understanding how to address the complex causes of childhood obesity requires explicit information about the factors associated with achieving behaviour change, and exactly how these factors have been addressed in an intervention [4].

The UK Medical Research Council’s (MRC) guidance for developing complex interventions informed by theory [13,14] is useful as a general approach to designing, implementing, and evaluating a complex behavioural intervention. The MRC framework recommends that the first step in developing a complex intervention is to establish a theoretical basis that suggests that an intervention should have the desired effect. This approach is supported by recent studies which indicate that interventions which making extensive use of theory tend to have larger effects on behaviour than interventions that make less or no use of theory [15,16]. This phase of assessing theory and evidence may identify the types of individual beliefs that promote or inhibit behavioural change, and start to shape the kind of intervention needed and identify the most appropriate study design [14]. However, a limitation of the MRC framework is that it does not provide detailed guidance about how to develop a complex intervention [17]. Intervention mapping (IM) [18] is a logical process for intervention development, implementation and evaluation that can be used to fulfil the criteria stipulated in the MRC framework.

IM describes a protocol for the development of a theory and evidence based intervention in five steps: 1) needs assessment, 2) identification of outcomes and change objectives, 3) selection of theory based methods and practical applications to change health related behaviour, 4) designing an intervention, and 5) creation of an implementation plan. IM is a stepwise approach for the planning and development of health promotion interventions. This approach has previously been used to develop a range of health behaviour change interventions [19,20], including those to tackle childhood obesity [9,21-23]. The process follows the development of an intervention, mapping the path from recognition of a need or problem to the identification of a solution [18]. In this paper, we aim to inform future programme planners about the process of developing a multifaceted intervention programme to prevent childhood obesity, which is integrated into existing health services within a multi-ethnic community in the UK, using the IM protocol.

Bradford is the fifth largest Metropolitan area in the UK and one of the most deprived. Fifty percent of the 6,000 babies born in Bradford each year are from South Asian origin (predominantly Pakistani). Rates of obesity in the city are higher than the national average and standards of healthy eating and physical exercise among the lowest in the country [24,25]. The importance of targeting minority ethnic communities in efforts to promote healthier lifestyles and prevent disease is recognised [26,27]. However, in doing so, it is vital that deep-rooted cultural (e.g., beliefs, traditions) and structural (e.g., socioeconomic status) influences on groups 'at risk’ of obesity are considered when developing interventions to impact on health behaviours [28-30]. This work therefore draws upon evidence for culturally adapting behavioural interventions in community settings [31-33] to develop a culturally appropriate intervention for reducing the risk of childhood obesity within the city of Bradford.

## Methods

This section presents details of the intervention development group, the approach used for cultural adaptation, and the six IM steps used to develop the intervention. Ethical approval for this work was obtained from the Bradford Research Ethics Committee (Reference: 07/H1302/112 and 11/YH/0458). Full written consent was obtained from all the participants in compliance with research ethics committee obligations.

### Intervention development group

An Intervention Development Group was convened at the outset of this project, which comprised of the Intervention Programme Manager, an IM specialist, experts in the research areas of each of the behaviours being targeted, and a group of experienced community health practitioners. This group also included the coordinator of an existing Parenting Programme within the city, given that the intervention would be integrated within this established local service. The group met every 6-8 weeks throughout the intervention development process; this was pivotal in contributing to the understanding of the challenges encountered by individuals in the community with respect to risk factors related to childhood obesity, and the feasibility of delivering a suitable intervention.

### Cultural adaptation

For each stage of the IM process, five recommendations from a recent review (24) were applied to ensure that White and South Asian perspectives were considered throughout: 1) use community resources to develop and publicise the intervention, 2) identify and address barriers to access and participation, 3) develop communication strategies which are sensitive to language and information requirements, 4) consideration of cultural/religious values that promote or hinder behavioural change, 5) recognise degrees of ethnic identification. In addition, best practice principles for community based interventions [33] were also referred to: 1) community-engagement to assess interest in concept and programme content, to ensure relevancy and cultural appropriateness; 2) implementation partnerships to ensure ownership and consistency of key messages and complementary actions, 3) embed in existing local NHS and Social Care structures and utilise existing community resources and facilities to promote sustainability; 4) development underpinned by behaviour change theory. Furthermore, during each step of IM, we worked with local practitioners (e.g., dieticians, infant feeding advisors, parenting practitioners, community health workers) with a wealth of experience in delivering community based interventions to a range of ethnic groups. We also worked with a team from the University of Edinburgh to use a theoretically underpinned typology for culturally adapting interventions, which was based on a realist synthesis of approaches employed to maximise the cross-cultural appropriateness and effectiveness of health promotion interventions for African-, Chinese- and South Asian-origin populations [32].

### Step 1: needs assessment

The initial step of the process involved conducting a literature review and a needs assessment.

#### Epidemiological evidence review

Relevant epidemiological literature was reviewed to identify modifiable risk factors for childhood obesity prevention. In addition, results from the Born in Bradford (BiB) 1000 cohort study were used to inform specific factors to target (e.g., maternal and infant diet, parenting practices, lifestyle behaviours, etc.). BiB is a multi-ethnic, birth cohort study which aims to examine environmental, psychological and genetic factors that affect maternal and child health and wellbeing [34,35]. BiB1000 is a subsample of this cohort which recruited specifically to examine the determinants of childhood obesity in order to aid development the current intervention by recruiting 1707 women from Bradford during pregnancy and following them up until the infant is aged 3 years [36].

#### Theoretical literature review

Literature was reviewed to identify theoretical determinants associated with the targeted behaviours, and previously effective practical intervention applications.

#### Community needs assessment

Qualitative interviews (*n* = 12), three focus groups (*n* = 27), and surveys (*n* = 1242) were undertaken with parents and grandparents, and surveys (*n* = 20) were undertaken with community healthcare professionals (e.g., health visitors, community dieticians). These activities sought to elicit information regarding current behaviours, specific barriers to performing behaviours to prevent childhood obesity, and to determine what might constitute a feasible community-based, family targeted, culturally appropriate childhood obesity prevention intervention.

### Step 2: identification of outcomes, performance objectives and change objectives

The next step involved specifying in detail the desired outcomes of the intervention. The overall desired outcome was to prevent childhood obesity. To achieve this outcome involved different behaviours [e.g., increasing physical activity (PA), eating a balanced diet, etc.], contexts (e.g., ante-natal, post-natal, home, childcare settings, etc.), and influences (e.g., personal, interpersonal, environmental, etc.), which were identified in the literature review. Desired outcomes were therefore defined to account for these factors.

Second, for each desired outcome, a set of performance objectives were specified. Performance objectives are used to refine, focus and make more specific what programme participants must do as a result of the intervention [18]. For example, if an outcome was for the mother to make antenatal healthy food choices and maintain a healthy diet postnatally, performance objectives could include: 1) Mother makes healthy food choices for herself, 2) Mother makes healthy food choices for her unborn baby, 3) Mother increases consumption of fruit and vegetables for herself, 4) Mother reduces the consumption of high-calorie, energy-dense foods and drinks for herself, 5) Mother copes with problems faced with eating a healthy diet. Each performance objective was refined and validated by an expert panel of researchers and community practitioners through an iterative process of examining the associated literature, the outcomes from the BIB cohort study, and consulting current practice guidance and policy about national recommendations (e.g., for PA in pregnancy, infant feeding, infant PA, etc.).

Next, the objectives of the intervention had to be specified in terms of the changes that need to be observed in the theoretical determinants of behaviour. This is crucial because it allows the intervention developer to identify exactly what psychological constructs need to change in order to have the desired impact on the performance objective, and ultimately the programme outcome. Performance objectives were scrutinised individually to identify what barriers are associated with achieving each desired outcome. Barriers provide information about the specific problems that people face to performing a behaviour, and can be clearly mapped to psychological determinants of behaviour [37]. For example, if a performance objective was for mothers to initiate breastfeeding at birth, associated barriers may include a lack of intent during pregnancy to breastfeed, pressure from family members to bottle feed, and/or a lack of experience of breastfeeding. These barriers can be mapped to the theoretical determinants of 'intention’, 'social influences’, and 'skills’, respectively. Following this mapping exercise, each barrier was transformed into a change objective, which specified exactly what needed to be changed in order to achieve each performance objective. Undertaking this process is useful because it enables those designing the intervention to a) state exactly what needs to be addressed in order to affect the performance objective, and b) select evidence-based behaviour change techniques (BCTs) that are effective in addressing specific psychological constructs. The output of this stage is a matrix of change objectives detailing what will be targeted in the intervention.

### Step 3: selecting methods and practical applications

Following the development of the change matrices, the next step was to select appropriate theoretical methods to change behaviour and operationalise these into practical applications. Guidance from Michie et al. [37,38] was used to map BCTs [39] to each theoretically derived behavioural determinant area (e.g., beliefs about capabilities, social influences, emotion, etc.) from an evidence based theoretical framework of behaviour change [37,38]. The most appropriate techniques were selected to address individual change objectives associated with each determinant area and translated into practical applications suitable for implementation in a community based programme. It is important that the practical application retains those characteristics that reflect the theoretical methods to avoid undermining effective behaviour change or counterproductive effects during the translation of the method to application and to the programme. Therefore, for each practical application, we ensured that the theoretical parameters of the selected methods were addressed. For example, for the BCT 'use opportunities for social comparison’ it may be more appropriate to use upward comparison to help set better goals, and downward comparison to help a person feel more self-efficacious; or for the BCT 'self-monitoring of behaviour’, the monitoring must be of the specific behaviour, the data must be interpreted and used, and any rewards must reinforce the individual [18]. Furthermore, existing resources currently being utilised by the community by practitioners as part of various health-promotion programmes were collated. A decision was made about which change objectives(s) would be addressed by the existing materials, and what BCT(s) the existing materials represented, therefore ensuring that all practical applications were based on sound theoretical methods. This process was undertaken separately for each target behaviour with expert researchers and practitioners in the respective areas from the intervention development subgroup. The suggested practical applications, mapped to determinants, change objectives, performance objectives and BCTs were then refined based on further discussion within the intervention group.

### Step 4: creating an organised programme plan

Once the initial set of practical applications had been created for each target behaviour, the intervention subgroup designed a structured programme plan for which delivery in the community would be realistic and feasible. Six key objectives were achieved by the end of this phase of programme development: 1) decide on the scope and limits of the intervention, 2) seamlessly weave the childhood obesity prevention programme elements into the selected existing health service (parenting programme), 3) decide on the exact content and mode of delivery for each practical intervention application, 4) ensure each of the change objectives had been addressed with appropriate practical application, 5) ensure the proposed intervention content was appropriate for the South Asian and White populations in the community, and 6) develop session materials.

### Step 5: creating an implementation plan

In the fifth step, an implementation plan was formulated. First, the location and frequency of the sessions was confirmed. Although a number of national and international obesity trials have been able to provide additional support for services such as health care support and visits e.g., [40,41], it is likely that resource constraints will continue for the health services internationally for the foreseeable future. As such, an important consideration for the generalisability of an intervention is the ease with which it can be integrated into existing health services without the need for additional resources. Therefore, it was decided that individuals currently delivering the existing Parenting Programme within the local community would receive additional training to deliver the childhood obesity prevention intervention as part of a holistic programme. The use of existing employees from the area provided a number of benefits, for example: 1) they were familiar with the existing programme, 2) they were identified due to their expertise for delivering a high quality service, 3) they were familiar with the local community which would ensure that the intervention could be further tailored to the needs of the target population, and 4) the intervention could be delivered as part of an existing community service without the need for additional input from the intervention development team or outside organisations, thus enhancing the chances of sustainability. Members of the delivery team were identified via the coordinator of the existing local service and the intervention programme manager to take on the role and were subsequently invited by the research team. A key component of this stage was the production of a training manual, development and delivery of specialist training sessions, and development of a protocol for liaising with delivery specialists as they implemented the intervention.

### Step 6: creating an evaluation plan

In the final step of the IM process, an evaluation plan was created. This involved the development of effect and process evaluation objectives, selection and/or development of indicators and outcome measures, and the development of an evaluation design specification.

## Results

### Step 1: needs assessment

#### Epidemiological evidence

Table 1 presents a summary of the key literature regarding modifiable risk factors for childhood obesity, and prevalence statistics based on local evidence from the BIB cohort study. To summarise, the literature review indicated that antenatal and postnatal BMI, diet, and PA, breastfeeding, and infant diet and PA were all modifiable risk factors associated with childhood obesity. Evidence from BiB 1000 cohort study [36,42-44] indicated that there is a prevalence of obesity in pregnancy in Bradford, and that there are relationships between each of these modifiable risk factors and overweight/obese infants up to 3 years of age.

**Table 1:** Literature, local population evidence, desired outcomes and performance objectives for the BIB intervention

**Literature**	**Epidemiological evidence for prevalence and sequelae from BIB**	**Desired outcome**	**Performance objectives (PO)**
Maternal and paternal BMI are two of the strongest predictors of childhood overweight/obesity [12]. Children of overweight and obese mothers are at particular risk of childhood obesity. Parental obesity more than doubles the risk of adult obesity among both obese and non-obese children under 10 years of age [45-48]. PA is a key component of weight control [49]. National UK guidance recommends at least 30 minutes of moderate intensity PA throughout pregnancy for most women [50-52].	Evidence from BIB 1000 cohort study indicated that 25.6% and 18.2% of the sample overweight (BMI 25-29.9) or obese (BMI ≥30) respectively. Over 7% of BiB1000 women had a BMI greater than 35 [36]. Mothers who were overweight or obese had infants with higher BMI z-scores at age 3 compared to women who were underweight or normal weight [42]. Infants of mothers who were overweight or obese at 26-28 weeks gestation were more likely to be overweight at age 3 [42]. 86.4% of pregnant women were inactive or moderately inactive at 26-28 weeks gestation, 62.4% were sedentary or insufficiently active at 6 months postnatally, and 63.9% were sedentary or insufficiently active at 12 months postnatally [43].	a) Mothers make antenatal healthy food choices and maintain a healthy diet postnatally	1 = Mother makes healthy food choices for herself 2 = Mother makes healthy food choices for her unborn baby 3 = Mother increases consumption of fruit and vegetables for herself 4 = Mother reduces the consumption of high-calorie, energy-dense foods and drinks for herself 5 = Mother copes with problems faced with eating a healthy diet
		b) Mother increases PA during pregnancy and meets guidelines (150mins mod intensity/wk) within 12 months of giving birth	1 = Mother meets the recommended guidelines of 150 minutes moderate PA/wk (can be done in 10 minute bouts) during and after pregnancy 2 = Mother performs physical activities that are safe during pregnancy 3 = Mother tries new physical activities during and after pregnancy 4 = Mother resists pressure from family/friends not to do PA during or after pregnancy 5 = Mother copes with problems faced with doing PA during or after pregnancy
Systematic review: initial breastfeeding protective against obesity in later life [53]. Meta-analysis concluded that the duration of breastfeeding was inversely and linearly associated with the risk of overweight. The risk was reduced by 4 per cent per month of breastfeeding. The effect lasted up to duration of breastfeeding for 9 months [54]. Current recommendations are that babies are exclusively breastfed for 6 months and that 6 months is the optimum age for the introduction of solid food for both breastfed and formula fed infants [55]. If parents choose to wean earlier than this, 4 months (17 weeks) should be regarded as the earliest age at which solids should be introduce [56].	The overall mean duration of breastfeeding was 1.7 months (range 0.03 – 8 mths), indicating that although a high percentage of mothers initiate breastfeeding, the duration is well below the national recommendation to exclusively breastfeed for 6 months duration and the ranges suggest some mothers gave up in the first few days [44]. In a sample of 1365 mothers, although 75.5% initiated breastfeeding, only 11% of babies were exclusively breastfed until 4 months of age, and by 4 months of age, only 28% of babies were receiving any breast milk [44].	c) Breast feeding is encouraged until at least six months	1 = Mother initiates breastfeeding at birth 2 = Mother exclusively breast feeds (or offers expressed milk) for 6 months 3 = Mother continues to breast feed once solids are introduced 4 = Mother introduces solids at about 6 months 5 = Mother/other guardian(s) uses bottle feed appropriately if this is the preferred feeding choice 6 = Mother copes with problems faced which are associated with breastfeeding
Inappropriate early dietary patterns that are established during weaning may persist into the second year of life and beyond [57]. The introduction of a variety of foods, tastes and textures during weaning and in early childhood is likely to contribute to a more varied and balanced diet in later life [58].	Overall 93% of mothers had introduced savoury solids by 6 months [44]. 71% of all mothers had offered sweet solids by 6 month. The mean age when sweet solids were introduced by all mothers was 4.9 months [44]. The overall mean age at which sweetened drinks were introduced was 4.8 months. Sweetened drinks are associated with the risk of development of obesity and the data indicate that all infants were offered these with some Pakistani infants being offered sweetened drinks as early as 5 weeks of age [44].	d) Infant develops healthy food preferences and dietary intake	1 = Mother/other guardian(s) is responsive to infant cues for hunger and fullness 2 = Mother/other guardian(s) adopts an authoritative parental feeding style (high control, high warmth) 3 = Mother/other guardian(s) does not use high energy foods as a reward 4 = Mother/other guardian(s) gives correct portion size for age of child. 5 = Mother/other guardian(s) does not feed baby in front of television. 6 = Mother/other guardian(s) encourages consumption of fruit and vegetables for child. 7 = Mother/other guardian(s) discourages inappropriate consumption of high-calorie, energy-dense foods and drinks for child. 8 = Mother/other guardian(s) copes with problems associated with ensuring infant has healthy dietary intake. NB: The interventions in this section also combat performance objectives 1 and 2 from 'infant feeding’, and performance objectives 1 and 2 from 'infant diet’.
Engaging in more sedentary activities (including television viewing) has been linked to the development of childhood obesity [59]. 40% of 3 month olds watching TV [60]. Expert groups recommend exposing infants to prone play or 'tummy time’ to help facilitate motor milstone development [61]. Many parents do not encourage prone play in their infants because of their infant resistance, or misperceptions around positioning during sleep and awake [62].	50.2% of infants had up to 1 hour of screen time per day, and 22.9% had > 1hour per day; these figures were similar for infants at 12 months. However, at 24 months, 37.7% of infants had up to 1 hour of screen time per day, and 54.7% were receiving > 1 hour [43].	e) PA for infant is facilitated and sedentary time is limited	1 = Mother/other guardian(s) ensures that infant has daily PA interactions in several bouts of both structured and unstructured play across the day. 2 = Mother/other guardian(s) provides a safe, clean floor space large enough for playing, rolling, crawling and other large muscle activities. 3 = Mother/other guardian(s) provide age appropriate equipment which promotes motor skill acquisition. 4 = Mother learns about the importance of PA for motor skill development and consequences for later life health. 5 = Mother/other guardian(s) encourages and motivates the infant’s PA participation. 6 = Mother/other guardian(s) ensures infant is not restrained in highchair/buggy/cot whilst awake for >1hr or watches TV for > 1hr. 7 = Mother/other guardian(s) copes with problems faced with ensuring infant has daily PA interactions.

Given that the mother is the usually the main care-giver and feeder, this evidence indicated that mothers would be the key individuals to target with an intervention that commences during pregnancy and continues up to the first 12 months of life. A systematic review of maternity experiences of women with BMI > 30 supports this idea by concluding that pregnancy is an ideal time to commence weight management programmes as women are more receptive to discussions about the benefits of a healthy lifestyle [63]. In addition, evidence highlights the importance of the whole family as the target for the intervention [64]. This is particularly important in South Asian communities where extended family members frequently play a key role in feeding and shopping [65-68]. A recent systematic review of culturally appropriate obesity prevention interventions also identified evidence for the use of experienced, respected and trusted community link workers, and the role of 'significant others’ such as grandmothers of South Asian children [32]. Given this evidence, and national guidelines (National Institute for Health and Clinical Excellence) [49] for multidimensional approaches to tackling childhood obesity (that involve local healthcare teams and communities that address the issues of lifestyle, diet, PA, working with the family, motivation and behaviour change), the decision to develop a multifaceted intervention encompassing each of these factors was taken.

#### Theoretical evidence

Literature was reviewed to identify theoretical determinants useful in predicting and explaining the performance of the behaviours the BiB intervention aimed to target. In attempting to explain behaviour a number of psychological models have been developed, such as the Health Belief Model [69], the Theory of Reasoned Action [70], the Theory of Planned Behaviour [71], and Social Cognitive Theory [72]. Several of these models include common attributes, such as a focus on our motivation to perform the behaviour (intention), the influence of others in doing so (social norms), our views about the behaviour (beliefs/attitudes), and whether or not we feel we can do it (control/self-efficacy), etc. Each of these models have predicted and explained a range of health behaviours e.g., [73-75]. With shared or overlapping constructs being applied by many of these theories [37], various authors have suggested that a range of theories might be used together to develop interventions [76,77].

Recent attempts have been made to assimilate these varying behaviour change constructs into a simple framework [37,78]. Michie et al. [37] developed a framework consisting of 11 theoretical domains (plus nature of the behaviours) said to encompass the determinants of behaviour change: (1) knowledge, (2) environmental context and resources, (3) motivation and goals (intention), (4) beliefs about capabilities (self-efficacy), (5) emotion, (6) social influences (norms), (7) skills, (8) beliefs about consequences (anticipated outcomes), (9) action planning, 10) memory, attention and decision processes, and 11) social and professional role and identity. It was intended that the use of this explanatory determinant list would allow for the selection and combination of effective behaviour change techniques (BCTs) and methods, based on over a century of research, to achieve desired behaviour change [79,80]. Since then, BCTs based on empirically supported theory have been mapped on to each of the 11 behavioural determinants [38]. These resources provide interventionists not only with an evidence-based foothold for developing theoretically underpinned behaviour change interventions, but also with the tools to explain how these interventions work once they have been tested [81]. Therefore, the Theoretical Domains Framework (TDF) was chosen to identify and address theoretical determinants of behaviour change through this intervention.

#### Community needs evidence

Upon completion of the literature review, the next step was to collect information using surveys and focus groups from families and practitioners in the community regarding the barriers to performing behaviours to prevent childhood obesity. The information collected was then combined with that from the literature to produce a final list of barriers (which were later transformed into change objectives). For example, for maternal diet, frequently cited barriers included 'a lack of confidence to cook healthy meals’, 'confusion about food labelling’, and 'lack of awareness about the impact of unhealthy foods on the unborn baby’. Additional file 1 presents a list of the barriers to performing each of the behaviours and how some of these differed between White and South Asian populations.

### Step 2: identification of outcomes, performance objectives and change objectives

The overall outcome of the current intervention was to prevent childhood obesity. Health behaviours that can reduce this risk were identified in the literature review. Although there were 'ideal’ levels of each of the health behaviours to aim for using the intervention, the in depth knowledge the BIB team had of the population (based on the cohort study and focus groups), and the input from health practitioners working in the community meant that desired outcomes were formulated based on realistic and achievable aims. Table 1 displays the desired outcomes for the BIB intervention mapped against the key literature and evidence from the BIB cohort study.

Next, the barriers identified for each of the 5 desired outcomes were grouped according to the determinant areas in the TDF (Additional file 1). For example, for desired outcome (3), the barrier 'lack of information about how to increase milk supply’ was mapped against the determinant 'knowledge’, whereas the barrier 'lack of private places to breastfeed’ was mapped against the determinant 'environmental context and resources’. Where barriers may have represented more than one determinant, they were placed in both. For example, the barrier 'men are offended by women who breastfeed in public’ was mapped against the determinant 'social influences’, and 'environmental context and resources’.

The next stage of the IM process was to specify the performance objectives for each of the desired outcomes. In a brainstorming session, the practitioner working group listed all the steps that would need to be taken in order to achieve the five outcomes. This process was informed by the applied knowledge of practitioners, and the theoretical knowledge about the determinants of behaviour change stipulated in the TDF from the research team. Various cycles of scrutiny and amendments were undertaken to narrow down the extensive list to a set of performance objectives that could be pragmatically achieved. A list of the performance objectives for each desired outcome is also listed in Table 1. Once the performance objectives had been finalised, the next stage was to match these to the barriers associated with the determinants of behaviour change from the TDF (Table 2). This helped the team to see which barriers would prevent each performance objective from being achieved. For example, for antenatal and post-natal diet (desired outcome a), performance objective 3 (PO3: Mother increases consumption of fruit and vegetables for herself) was mapped against barriers that included 'do not know how to cook a meal from scratch’, and 'no motivation to eat healthily’, which had been previously matched to the determinants 'skills’ and 'motivation and goals’, respectively. Barriers were found to represent all 11 domains, with the exception of 'social and professional role and identity’. Next, each barrier was transformed into a change objective, which specified exactly what needed to be changed in order to achieve each performance objective. For example, the barrier 'does not know how to cook a healthy meal from scratch’ was transformed into the change objective 'develops skills to cook healthy meals from scratch’. Finally, using guidance from Michie et al. [38], a list of psychological constructs associated with each determinant area from the TDF were used to identify what would need to be targeted in order to address each change objective and achieve each performance objective. Tables listing the determinants, change objectives, performance objectives and constructs for each desired outcome are presented in Additional file 2.

**Table 2:** Antenatal and postnatal diet (desired outcome 1) example of mapping determinant areas, barriers, change objectives, performance objectives and constructs

**Determinant area**	**Barriers**	**Change objectives**	**Performance objectives**	**Constructs**
Skills	Do not know how to cook a meal from scratch	Develops ability to cook a meal from scratch	PO3, PO4	- Skills, competence
- Does target group know how to do x?				- Skills, assessment
				- Practice
				- Skill development
Motivation and goals	No motivation to eat healthily	Increases motivation to eat healthily	PO3, PO4, PO5	- Intention/certainty of intention
- How much does target group want to do x?				- Intrinsic motivation
				- Commitment, stability of intention

### Step 3: selecting methods and practical applications

The third stage of the IM process involved identifying theoretical methods which have been suggested as effective in changing theoretical determinants. BCTs from a taxonomy of behaviour change techniques [82] were mapped against each of the determinant areas. Change objectives for each determinant area were then addressed on an individual basis by operationalising the mapped BCTs into pragmatic and practical applications. Practical intervention applications were developed together by the behaviour change experts and practitioners in order to ensure that each one a) was theoretically underpinned, b) met the theoretical parameters of use, c) addressed a specific change objective(s), and d) was realistically implementable in a programme to be delivered by practitioners in a community setting. Examples of theoretical methods and practical applications relating to 'skills’ and 'motivation and goals’ change objectives for antenatal and postnatal diet (desired outcome 1) performance objectives two, three, and four (mother makes healthy food choices for her unborn baby, mother increases consumption of fruit and vegetables for herself, mother reduces the consumption of high-calorie, energy-dense foods and drinks for herself) are displayed in Table 3. For example one change objective was to increase mothers’ motivation to eat healthily. Theoretical methods suggested by Michie et al. [38] that were deemed useful for this particular change objective include 'provide information about consequences’ (focusing on what will happen if the person performs the behaviour, including the benefits and costs of action/inaction) and 'prompt barrier identification’ (think about potential barriers and ways of overcoming them). The theoretical parameters for use for these BCTs involve raising awareness, which must be quickly followed by an increase in problem-solving and self-efficacy [18]. In light of these theoretical methods we concluded that practical applications could be for the practitioner to provide information about the consequences of eating an unhealthy diet, and run a group exercise to identify and overcome mother’s barriers to eating healthily. The full mapping exercise is available in Additional file 1.

**Table 3:** Antenatal and postnatal diet (desired outcome a) example of mapping determinant areas, change objectives, behaviour change objectives, and practical intervention applications

**Determinant area**	**Change objectives**	**BCTs**	**Practical application**
Skills	Develops ability to cook a meal from scratch	- Increasing skills; Prompt self-monitoring	- Cook a healthy meal and report outcomes
- Does target group know how to do x?			
Motivation and goals	Increases motivation to eat healthily	- Provide information about consequences	- Importance of eating well for the baby
How much does target group want to do x?		- Prompt barrier identification	- Address barriers to healthy eating and plan for ways to overcome them

### Step 4: creating an organised programme plan

Given the evidence from a recent systematic review of culturally appropriate obesity prevention interventions, advocating the use of experienced, respected and trusted community link workers, and the need to ensure the intervention was integrated into existing public services [33], a decision was made early in the process to develop an intervention in combination with an established community parenting programme. The Family Links Nurturing Programme (FLNP) is a parenting programme designed to promote emotional health and well-being, relationship skills and positive behaviour management strategies for parents/carers [83], which has been successfully delivered to families across Bradford for the past 5 years. A working relationship was established between the BIB and Family Links teams at the outset of this project. The coordinator of the FLNP was involved in all stages the intervention development process as a member of the working group and played a pivotal role in steps 4 and 5 to create an organised programme plan. With members of the research team, the FLNP, and community practitioners working together with a degree of flexibility, this ensured that the objectives of the intervention were fulfilled without compromising the philosophies or key content of the existing parenting programme.

**Figure 1:**
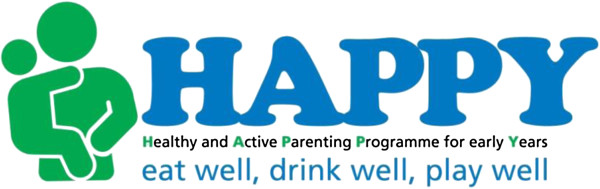
HAPPY logo.

The first task in step 4 was for the Intervention Development Group to devise a structured programme plan that accounted for the scope and limits of the intervention. The evidence from the needs assessment indicated that pregnancy and the first few months of infant life are critical periods influencing the risk of childhood obesity, and an extensive list of barriers were found to be associated with the desired outcomes defined for these two phases. Therefore, a decision was made to develop an intervention that addressed the identified modifiable risk behaviours in the antenatal and postnatal phases of pregnancy. Consequently, the timing of the delivery of the intervention also needed to be considered at this point, and a survey of (*n* = 125) mothers in the maternity unit at the hospital and in local children’s centres was undertaken to elicit opinions about the most effective way to run the intervention to facilitate attendance. Taking into account the evidence, resources and time available, the level of flexibility that the FLNP could offer, and the opinions from mothers, the following structure was agreed: a 12 session programme, split into 2 components (antenatal and postnatal), both of which consist of six 2.5 hour sessions. The antenatal sessions were scheduled weekly and the postnatal sessions were scheduled at timely intervals related to key developmental milestones in infancy and to provide optimum intervals for sharing health related messages with parents. The antenatal programme was scheduled to start in the second trimester (between 26 and 28 weeks) of pregnancy, and the postnatal programme was scheduled to start between 4-6 weeks after the birth and continue up to the infants were approximately nine months old. The programme was named 'HAPPY’ (Healthy and Active Parenting Programme for early Years; Figure 1), and was designed to be delivered to parents by the existing FLNP practitioners who had established skills and expertise in parenting approaches, thereby increasing the likelihood of sustainability.

Next, the Intervention Development Group worked together to integrate the practical intervention applications into the existing parenting programme, and decide on the exact content of each practical intervention application, as well as the nature by which it would be delivered. Key themes integrated into the programme focussed on the five desired outcomes (relating to responsive feeding, antenatal and postnatal diet, antenatal and postnatal PA, infant diet, infant PA), and were delivered according to the stage of the pregnancy. For example, in the 6 week antenatal programme, although each of the key themes was addressed, more time was dedicated to addressing antenatal diet and PA, and breastfeeding; in the postnatal programme, time was dedicated to breastfeeding, postnatal diet and PA, and infant diet and PA. Throughout the process of developing the programme, considerations were made regarding budget, time/skill of the delivery staff, pragmatism of the suggested practical intervention applications, cultural adaptation, and the FLNP philosophies, in order to ensure implementation would be successful. For example, to address the change objective to 'develop skills for cooking healthy meals from scratch’, the 'modelling/demonstration’ and 'rehearsal of relevant skills’ BCTs led to the development of a practical application for mothers to be able to participate in a 'cook and eat’ class as part of the intervention. However, time/resources, and the expertise of the delivery team did not allow for this. Therefore, the practical application was refined to look at recipe resources (provide instruction) during the session and make plans for cooking a health meal (action planning), and ask that mothers try this at home (rehearsal of relevant skills) and feedback on the outcomes. Furthermore, in the development of this practical application (and all other applications), we referred to the cultural adaptation typology for interventions [32]; see Additional file 3; this tool can be used for the systematic and transparent consideration of approaches to planning and reporting on adapted interventions. Examples of criteria include: use of an ethnically-matched intervention staff or facilitator (with qualifications), utilises local/respected religious/spiritual leaders, and material depicts individuals from target population. Each member of the intervention development team circulated the Lui et al 46-item typology to identify ways in which we could ensure that the intervention met each of the criteria and encompassed the necessary level of cultural adaptation. For example, referring back to the change objective 'develop skills for cooking healthy meals from scratch’ the practical application incorporated instructions for cooking a healthy meal that were relevant to both South Asian and White cultural traditions (e.g., suggesting recipes that were differentiated to include foods more traditionally eaten by South Asian and White populations).

Throughout this process of refining practical application, the intervention development team were mindful of ensuring that change objectives were being addressed using appropriate BCTs suggested in the IM guidance [38]. However, the guidance provided matched different numbers of BCTs to determinants. For example, the determinant 'knowledge’ has one agreed associated BCT; however, 'skills’ has ten associated BCTs. If the situation arose where it became difficult to create practical applications for all the change objectives in a determinant area based on one BCT, techniques that had not been previously matched with determinants by Michie et al [38] were considered, and after detailed assessment, the decision of whether or not to include additional techniques was made (see Additional file 2 for full mapping exercise).

The final intervention was designed to be delivered in a systematic fashion by FLNP practitioners (see Table 3 for a summary of structure and content). Using practitioners in the delivery removed the need for expertise of the research team and enhanced the likelihood of sustainability. An intervention manual (Additional file 4 – this is cross referenced with the full mapping exercise presented in Additional file 2) was developed for practitioners and included detailed instructions for how to deliver each session, accompanied with a series of resources to be used each week. Resources included equipment (e.g., 'Eat-Well plate’ handouts and laminated 'pictures of foods’ for a group activity to improve knowledge about healthy foods and skills for combining foods according to correct portion sizes), work-books (e.g., food, activity and mood diaries), leaflets (e.g., appropriate physical activities for babies at 3 months). Practitioners familiar with both the FLNP and other programmes addressing factors associated with the desired outcomes here were directly involved in the production of the manual, which was circulated at the end of each drafting stage. The input here was vital as it provided a way to ensure that the practical applications included in the intervention were perceived as appropriate and feasible from those familiar with working at the sharp end of delivery.

### Step 5: creating an implementation plan

Once the intervention manual had been finalised, the next stage was to ensure successful adoption and implementation of the intervention amongst the target group. A crucial element of this was to ensure that the practitioners received appropriate training and instruction in order to implement the intervention in the intended way to ensure programme fidelity. In addition to the intervention manual described in Step 4, the outcome of this was a comprehensive training programme for practitioners delivering the intervention (see Table 4). The delivery team were informed that their sessions would be sporadically monitored by members of the research team to assess the fidelity of the intended intervention. Finally, the team were also informed that they would be requested to complete an evaluation form at the end of each session to provide an overview of the practical intervention applications delivered, information about how the session was received, what worked well, what did not, and what could be improved.

**Table 4:** Contents of the BIB training programme for practitioners

**Session**	**Description of content**
Background	The background and context for the BIB obesity intervention study
Overview	An overview of the HAPPY programme, an introduction to the 'Family Links’ parenting programme
Intervention mapping	An introduction to intervention mapping. A lay explanation about how the intervention mapping approach was used as the foundation of the HAPPY intervention
BIB and Family Links	How the intervention has been carefully woven into the existing family links programme
Evidence based practical education on the key messages	Ensuring clear understanding and consistent approach to the nutrition, infant feeding and physical activity elements of the programme (new to these parenting practitioners), and their delivery using the manual and activities including cultural adaptation delivered by specialist practitioners (dietician , infant feeding and PA specialists)
The manual	Time to familiarise themselves with the manual and resources
Boundaries and scope	An overview of boundaries, scope, roles and responsibilities
Difficult questions	Information about how to deal with difficult questions
Time to have a go	Participation in activities included in the manual
Sticking to the manual	An explanation of the importance of adhering to the manual in order to obtain a true test of the impact of the intervention (and that there would be times whereby the BIB team would be monitoring sessions to assess intervention fidelity)
Recording delivery experiences	Information about how to log details about the way they delivered the intervention in order to determine which components of the intervention were delivered consistently, which were not, and the reasons for any discrepancies.

### Step 6: evaluation plan

The evaluation plan for a feasibility randomised controlled design has been developed, which will assess acceptability, fidelity, attrition and follow up, and estimates of effect size. To summarise the recruitment and outcome measures, overweight pregnant women (defined as Body Mass Index (BMI) ≥25 kg/m2) will be recruited between 10-26 weeks gestation of pregnancy and allocated on a 1:1 basis to either a 12 week intervention programme (6 sessions antenatal, 6 sessions postnatal) or usual care. Mothers’ height and weight at the time of 'booking’ (the first contact with midwifery services, around 8-12 weeks gestation) will be collected from maternity notes in order to calculate BMI. At baseline and 12 months, mother’s weight will be measured by a member of the research team. Baby’s birth weight will be extracted from the mother’s maternity record, and self-reported by mothers (from routine measurements recorded in the child’s 'red book’ health record) at 6 months. At 12 months baby’s length and weight will be measured by a member of the research team.

Given the uncertainty of an appropriate outcome measure, the aim of the feasibility study is to explore the variability and responsiveness of a variety of outcome measures, including estimates of effect sizes between control and intervention, in preparation for the full RCT. The potential primary outcomes that will be explored are: a) the proportion of children who cross two centile bands (>1.33 standard deviation score: SDS) for weight age 1 year, b) the proportion of children who cross one centile (>0.67 SDS) for weight at age 1 year, c) age and sex adjusted weight SDS at one year, and d) proportion of children aged 1 with weight > 85^th^ centile. Other validated process and outcome measures include baseline, six, and 12 monthly objective (where possible) and self-reported assessments of maternal diet, breastfeeding, physical activity, and sedentary behaviours, infant diet, physical activity, and development, and parenting practices.

Participants in the control groups will receive usual care. In Bradford this includes support from health professionals and support agencies including midwives, health workers and self-accessed services delivered in a range of locations (e.g. children’s centres, health clinics, voluntary provision). Due to resource constraints, for the purposes of the feasibility trial, content and delivery mechanisms of usual care services will not be directly observed at the same level of detail as that which has been provided for the development of intervention content (i.e., the audit trail for intervention development), or will be provided for the delivery (i.e., session observations). However, for the large scale RCT, it will be important to build in plans to assess usual care (including frequency of delivery, ease of access, content of resources available, etc.) to enable conclusions to be drawn about the combination of components unique to the intervention condition [84,85].

## Discussion

In this paper, we have presented a detailed outline of how the IM protocol was used to develop a multifaceted and evidence based early intervention programme to prevent childhood obesity, which is integrated into existing health services within a multi-ethnic community in the UK. To our knowledge, this is the first childhood obesity prevention intervention of its kind that incorporates evidence based on epidemiological and psychosocial data collected from the local community, and combines parenting programme components with childhood obesity risk factors. As a result of this complex process, a 12 week antenatal and postnatal HAPPY intervention has been embedded into an existing parenting programme within the community. The intervention addresses key barriers to health behaviour change amongst pregnant women and their families in the community using theoretically underpinned behaviour change techniques. To assess the feasibility of a full scale RCT of the HAPPY intervention, a randomised controlled pilot trial will examine: recruitment rates, attendance and attrition, acceptability of the trial procedures and of the HAPPY intervention to parents, fidelity of intervention implementation, capability and capacity for practitioners to deliver the intervention and the feasibility of the tools used to measure health (e.g. BMI), behavioural (e.g. diet and physical activity), and psychological (e.g. intention, self efficacy) outcomes.

### Strengths, challenges, and limitations

A perceived strength, and perhaps the most vital and novel aspect of this work is the use of the BiB1000 cohort study evidence to inform the design and content of the intervention to ensure it was relevant and culturally appropriate for the target population. The use of MRC guidelines for complex interventions [14], and national guidelines and policy to develop a multidimensional approach to tackling childhood obesity by involving local healthcare teams who work with the family to enhance motivation and behaviour change, are also likely strengths of this approach. Given the evidence that indicates antenatal and early postnatal factors play a key role in the development of childhood obesity [86,87], potential advantages of this intervention over some other previously developed programmes to tackle childhood obesity might also include the timing of delivery (i.e., prior to birth and early in the post-natal phase). Furthermore, using IM has enabled the team to develop a theoretically underpinned and evidence-based intervention, the contents of which are transparent and replicable.

There are a number of challenges associated with our approach. First, using IM to develop such a large scale intervention which addresses multiple behaviours (e.g., family diet and PA, child diet and PA, responsive feeding, parenting) is a time consuming process, which involves a number of individuals from a range of areas of expertise (e.g., dieticians, PA specialists, epidemiology and health behaviour change researchers, midwives, community health practitioners, parenting specialists, etc.). The intervention development lead must carefully manage the perspectives of all parties involved to create a programme that balances evidence and pragmatism effectively. For example, the lead must ensure that those involved in programme development have sufficient knowledge of the IM process requirements, such that they can develop practical intervention applications that address change objectives using appropriate BCTs which are likely to be feasible and effective in practice. These tasks often involve a considerable amount of time spent reviewing, revising and refining (e.g., to performance objectives, practical intervention applications, etc.) based on suggested amendments from development team members, and double checking that each step in the process has been undertaken in the correct way.

The current intervention is not without limitations. As with any rigorous research into epidemiology, the speed at which data collected from the local population can be used to inform the content of the intervention is slow. However, evidence from the wider scope of literature for both South Asian and White communities has also been used throughout this design process. Although systematic and evidence-based, the IM process has an element of subjectivity due to the need to merge epidemiological and psychosocial research evidence, matrix mapping, and feasibility information [17]. However, some of the outcome measures to be used in the feasibility trial should allow for links to be made between the psychological determinants targeted, the BCTs used, and any changes in behaviour. Another limitation is related to the assessment of the fidelity of intervention delivery. Although we have informed delivery staff of the theoretical foundation and rationale for this approach in intervention design, and provided all the resources to be able to deliver the interventions as intended, the risk remains that they may go 'off message’. However, delivery staff will be required to complete session evaluation forms to provide them with the opportunity to highlight any problems associated with programme delivery, whether something has/has not worked well, etc., information from which can inform refinements of the intervention. These plans should also allow for identification (or explanation) of any potential negative effects of the intervention [49]. In addition, a selection of sessions will be observed by the intervention development group for to assess programme fidelity. Future plans also include using experienced facilitators from the current programme to help a) refine the intervention before further testing, and to involve them in the training that will be provided to new members of the delivery team – this is likely to be a useful way of imparting knowledge and building enthusiasm [73].

## Conclusions

The current paper presents a detailed outline of the HAPPY programme which has been developed using IM. Making use of this approach produces a transparent and replicable intervention, whereby mechanisms of change can be investigated and identified, and practical applications used to manipulate them can be appropriately refined. We have demonstrated that IM is a feasible and helpful method for providing an evidence based and theoretical structure to a complex health behaviour change intervention. The next stage will be to assess the impact of the intervention on behaviour change and clinical factors associated with childhood obesity. The HAPPY programme is currently being tested in a randomised controlled pilot trial.

## Abbreviations

BCTs: Behavioural change techniques; BiB: Born in Bradford; FLNP: Family links nurturing programme; HAPPY: Healthy and Active Parenting Programme for Early Years; IM: Intervention mapping; NHS: National health service; PA: Physical activity; TDF: Theoretical domains framework; UK MRC: UK medical research council.

## Competing interests

The authors declare that they have no competing interests

## Authors’ contributions

NT led the design of the intervention from a theoretical standpoint and led the writing process. PS brought infant nutrition expertise to the group, contributed to the design of the intervention and helped to edit the manuscript. JW is the principle investigator for this study, and helped to design the intervention and edit the manuscript. JS contributed to the design of the intervention in conjunction with the existing parenting programme and helped to edit the manuscript. SB led the design of the physical activity aspects of the intervention. JL led the design of the diet and infant feeding aspects of the intervention. GL double coded all intervention content according to BCTs and helped to edit the manuscript. All authors read and approved the final manuscript

## Authors’ information

NT is a member of the BiB1000 steering group, and has previously (and currently) worked on projects that involve using the TDF to identify barriers and design interventions using theoretically underpinned behaviour change techniques to design tailored interventions to address key barriers for a range of health behaviours. PS is a Professor of Nutrition and Childhood Obesity, and is a member of the BiB1000 steering group. JW is a Professor of Epidemiology, and is the director for the overall BiB programme. JL is a dietician and a member of the BiB1000 steering group. SB is a physical activity specialist, has previously developed interventions for promoting physical activity in a range of populations, and is a member of the BiB1000 steering group. GL has previous experience of coding interventions according to BCTs.

## Supplementary Material

Additional file 1 Key barriers for each desired outcome mapped against TDF domains. Click here for file

Additional file 2 Matrix of change objectives, performance objectives, BCTs, and practical intervention applications, cross-referenced with the intervention manuals. Click here for file

Additional file 3 Cultural adaptation table. Click here for file

Additional file 4 **a. MASTER HAPPY Antenatal Manual FINAL VERSION. b.** MASTER HAPPY Postnatal Manual FINAL VERSION. **c.** HAPPY manual resources. Click here for file
