# Supplementary material for: Using intervention mapping to develop a culturally appropriate intervention to prevent childhood obesity: the HAPPY (Healthy and Active Parenting Programme for Early Years) study
Source: Int J Behav Nutr Phys Act. 2013 Dec 28;10:142. doi: 10.1186/1479-5868-10-142 (PMC3895739; doi:10.1186/1479-5868-10-142)
Supplement: Additional file 1 — Key barriers for each desired outcome mapped against TDF domains. [file 1479-5868-10-142-S1.doc]

Additional file 1. Key barriers for each desired outcome mapped against TDF domains

**Table 1. Desired Outcome: Mothers make antenatal healthy food choices and maintain a healthy diet postnatally (according to UK guidelines)**

| **Barriers** | **TDF domains** |
| --- | --- |
| - Don’t know what constitutes physical activity and the guidelines for physical activity in pregnancy/postnatal | Knowledge and Beliefs about Consequences |
| - Worry about loss of baby – self or friend has had previous miscarriage or received artificial insemination | Knowledge and Beliefs about Consequences |
| - Information from health professionals is inconsistent | Knowledge |
| - Mother believes physical activity is unsafe during pregnancy | Knowledge |
| - Do not know what activities are available to them and types of appropriate activities | Knowledge |
| - Perceives physical activity to be unsafe for self during pregnancy/postnatal | Knowledge, Beliefs and Consequences |
| - Lack of energy | Emotion |
| - Social expectation that pregnant women should not exercise | Social Influences |
| - Information from media is conflicting and impersonal | Social Influences |
| - Conflict of advice from family members | Social Influences |
| - Not confident that what they are doing is right | Beliefs about Capabilities |
| - Mother does not have the skills to engage in physical activity e.g. swimming | Skills |
| - Low self-confidence due to size | Beliefs about Capabilities |
| - Need to prioritise day-to-day tasks and spending time with family/child | Motivation and Goals; Behavioural Regulation |
| - Sickness | Emotion; Behavioural Regulation |
| - Feels neighbourhood is unsafe | Environmental Context and Resources |
| - No money for classes/equipment | Environmental Context and Resources |
| - Weather is too hot/cold/rainy/snowy to go out | Environmental Context and Resources |
| - Lack of facilities, pre-/postnatal classes, no facilities with childcare | Environmental Context and Resources |
| - No motivation | Motivation and Goals |

**Table 2. Desired Outcome: Mother increases physical activity during pregnancy and meets the guidelines of 150 minutes moderate intensity exercise per week by six months postnatal**

| **Barriers** | **TDF domains** |
| --- | --- |
| - Don’t know what constitutes physical activity and the guidelines for physical activity in pregnancy/postnatal | Knowledge and Beliefs about Consequences |
| - Worry about loss of baby – self or friend has had previous miscarriage or received artificial insemination | Knowledge and Beliefs about Consequences |
| - Information from health professionals is inconsistent | Knowledge |
| - Mother believes physical activity is unsafe during pregnancy | Knowledge |
| - Do not know what activities are available to them and types of appropriate activities | Knowledge |
| - Perceives physical activity to be unsafe for self during pregnancy/postnatal | Knowledge, Beliefs and Consequences |
| - Lack of energy | Emotion |
| - Social expectation that pregnant women should not exercise | Social Influences |
| - Information from media is conflicting and impersonal | Social Influences |
| - Conflict of advice from family members | Social Influences |
| - Not confident that what they are doing is right | Beliefs about Capabilities |
| - Mother does not have the skills to engage in physical activity e.g. swimming | Skills |
| - Low self-confidence due to size | Beliefs about Capabilities |
| - Lack of facilities, pre-/postnatal classes, no facilities with childcare | Environmental Context and Resources |
| - Need to prioritise day-to-day tasks and spending time with family/child | Motivation and Goals; Behavioural Regulation |
| - Sickness | Emotion; Behavioural Regulation |
| - No motivation | Motivation and Goals |
| - Feels neighbourhood is unsafe | Environmental Context and Resources |
| - No money for classes/equipment | Environmental Context and Resources |
| - Weather is too hot/cold/rainy/snowy to go out | Environmental Context and Resources |

**Table 3. Desired Outcome:** Breastfeeding is encouraged until at least six months

| **Barriers** | **TDF domains** |
| --- | --- |
| - Do not intend to breastfeed | Motivation and Goals |
| - Lack of information on how to breastfeed successfully/how to bottle feed correctly | Knowledge |
| - Believe that infant needs to finish the whole bottle to put on enough weight | Beliefs about Consequences |
| - Believe that need to make baby wait between bottle feeds so they will sleep longer, so they give them too much milk in one feed | Beliefs about Consequences |
| - Amongst South Asian generally, and older white generation suggestion that mothers need to maximize weight gain by feeding child whole bottle | Social Influences |
| - Mothers do not want to waste milk as it is very expensive, so want to make sure baby consumes all the bottle in one sitting | Environmental Context and Resources |
| - No information on how to increase milk supply | Knowledge |
| - Not confident with breastfeeding at first | Beliefs about Capabilities |
| - Breastfeeding alone would lead to babies sleeping for less at night | Beliefs about Consequences |
| - Time-consuming in comparison to bottle feeding | Motivation and Goals; Behavioural Regulation |
| - Lengthy hours spent breastfeeding | Motivation and Goals |
| - Too stressed to produce milk; worry that child is not happy breastfeeding; feelings of inadequacy and failure if think not producing enough milk; feel as though child needs something ‘more’ than they can provide | Emotion |
| - Main reason parents and in-laws suggested formula feeding appears to stem from belief that breast milk not enough to feed a new-born; mothers have difficulties telling relatives that breastfeeding is adequate and no need for mixed-feeding | Social Influences |
| - No information on how much breast milk is enough | Knowledge |
| - Not sure how to get the baby to latch on | Skills |
| - Put off by frequent feeds and sore nipples and breasts | Beliefs about Consequences |
| - Lack of private places to breastfeed | Environmental Context and Resources |
| - Pressure from parents and in-laws leads to early introduction of formula milk for the infant leading to feelings of inadequacy and failure | Emotion; Social Influences |
| - Pressure from mother-in-laws who will take over the care of the babies, because they can give the baby a bottle while the mother is getting on with the chores | Social Influences |
| - Does not have the skills to make bottle up correctly | Skills |
| - Not confident enough to ask for help | Beliefs about Capabilities; Social Influences |
| - Breastfeeding restricts movement outside of the house with the infant | Environmental Context and Resources; Behavioural Regulation |
| - No support with housework | Social Influences |
| - Husbands want to pitch in so they can have interaction with child and help out resulting in pressure to bottle feed | Social Influences |
| - Have to return to work which increases chance of discontinuing breastfeeding | Environmental Context and Resources; Behavioural Regulation |
| - Put off by frequent feeds and sore nipples and breasts | Beliefs about Consequences |
| - Inappropriate response to babies distress by feeding every time baby cries | Skills |

**Table 4. Desired Outcome: Physical activity for infant is facilitated and sedentary time is limited**

| **Barriers** | **TDF domains** |
| --- | --- |
| - Lack of understanding about what constitutes physical activity and what the guidelines for physical activity are | Knowledge |
| - Unaware of the benefits of active play and consequences of sedentary behavior | Knowledge |
| - No information on importance of motor skill development for later life health | Knowledge; Beliefs about Consequences |
| - Sport is for boys and should not be encouraged in girls | Social Influences |
| - Playing outside is unsafe | Environmental Context and Resources; Behavioural Regulation |
| - Weather is too hot/cold/rainy/snowy to go out | Environmental Context and Resources; Behavioural Regulation |
| - No space/safe space inside the house to play actively | Environmental Context and Resources; Behavioural Regulation |
| - No money for age-appropriate equipment | Environmental Context and Resources Behavioural Regulation |
| - No money for activities or to travel to activities | Environmental Context and Resources Behavioural Regulation |
| - No time to engage child in structured play | Motivation and Goals; Behavioural Regulation |
| - Child does not want to engage when mother is free to do so | Motivation and Goals; Behavioural Regulation |
| - Cannot prepare nutritious meals and engage infant in activities | Skills |
| - Family has no interest in physical activity | Social Influences |
| - Not confident that what they are doing/giving is right | Beliefs about Capabilities |
| - Mother doesn’t know how to play in a stimulating way with her baby | Knowledge; Skills |
| - TV is used as a baby-sitter | Environmental context and Resources |
| - Mother not confident to take child out of the house | Beliefs about Capabilities |
| - No time to engage child in structured play | Motivation and Goals; Behavioural Regulation |
| - Child does not want to engage when mother is free to do so | Motivation and Goals; Behavioural Regulation |
| - Too busy to go to activities outside the home | Motivation and Goals; Behavioural Regulation |
| - Mother feels embarrassed playing with child in public | Social Influences and Emotion |

**Table 5. Desired Outcome:** **Infant develops healthy food preferences and dietary intake**

| **Barriers** | **TDF domains** |
| --- | --- |
| - Work makes it difficult to cook healthy meals for the child | Motivation and Goals; Behavioural Regulation |
| - Variation in routine makes it difficult to plan | Motivation and Goals; Behavioural Regulation |
| - Lack of knowledge about why/when to start weaning - including signs of readiness | Knowledge |
| - Lack of knowledge about what food and drinks to give | Knowledge |
| - Mothers may not be sure what the right portion sizes are for their children | Knowledge |
| - Not sure how to cook healthy (weaning) meals | Skills |
| - Lack of knowledge of how to prepare fresh baby foods, therefore take the easier option of ready meals | Knowledge; Skills |
| - Not sure what the best way is to get my child to want to eat fruit and vegetables | Skills |
| - Mothers feeding their children an unhealthy diet don’t believe there is a problem with the food they feed their child | Knowledge; Beliefs about Consequences |
| - Lack of knowledge about how to give food and drinks- weaning | Skills |
| - Do not have the correct equipment for preparing and feeding nutritious –weaning meals | Environmental Context and Resources |
| - Lack of time to prepare nutritious (weaning) meals for infant | Motivation and Goals; Behavioural Regulation |
| - Takes too long to plan/prepare/cook healthier meals | Motivation and Goals; Behavioural Regulation |
| - Mothers may not be sure what the right portion sizes are for their children | Knowledge |
| - Mothers may feel it’s too much of an effort to constantly make sure child is eating healthy | Motivation and Goals |
