# Supplementary material for: Using intervention mapping to develop a culturally appropriate intervention to prevent childhood obesity: the HAPPY (Healthy and Active Parenting Programme for Early Years) study
Source: Int J Behav Nutr Phys Act. 2013 Dec 28;10:142. doi: 10.1186/1479-5868-10-142 (PMC3895739; doi:10.1186/1479-5868-10-142)
Supplement: Additional file 2 — Matrix of change objectives, performance objectives, BCTs, and practical intervention applications, cross-referenced with the intervention manuals. [file 1479-5868-10-142-S2.docx]

**Additional File 2. Matrix of Change Objectives Mapped against Behaviour Change Techniques and Cross Referenced against the Intervention Manual (Additional file 4)**

**Table 1. Behaviour Change Technique Names and Definitions**

| **Number** | **Name** | **Definition** |
| --- | --- | --- |
| BCT 1 | Provide general information on behaviour-health link | Information about the relationship between the behaviour and health – including susceptibility or factual risk and/or mortality information OR. health education material relevant to the behaviour. |
| BCT 2 | Provide information on consequences | Involves providing information focusing on what will happen if the person performs the behaviour including the benefits and costs of action or inaction. |
| BCT 3 | Provide information about others’ approval | Involves information about what other people think about the reader’s or target person’s behaviour. It clarifies whether others will like, approve or disapprove of what the person is doing or will do. |
| BCT 4 | Prompt intention formation | Involves encouraging the person to set a general goal or make a behavioural resolution e.g., “I will take more exercise next week” would count as a prompt to intention formation. This is directed towards encouraging people to decide to change. |
| BCT 5 | Prompt barrier Identification | Think about potential barriers and plan ways of overcoming them. Barriers may include competing goals in specified situations. This may be described as “problem solving” and if it is problem solving in relation performance of the behaviour i.e., then it is an instance of this technique. |
| BCT 6 | Provide general encouragement | Involves praising or rewarding the person for effort or performance without making this contingent on specific behavioural performance; or “motivating” the person in an unspecified manner. This will include attempts to enhance self efficacy through argument or persuasion (e.g., telling someone the will be able to perform a behaviour). |
| BCT 7 | Set graded tasks | Set the person easy-to-perform tasks, making them increasingly difficult until target behaviour is performed. |
| BCT 8 | Provide instruction | Involves telling the person how to perform a behaviour or preaparatory behaviours. For example, providing individual face to face instructions, offering an instructional group class or providing “tips” on how to take action in text form. |
| BCT 9 | Model/ Demonstrate the behaviour | Involves showing the person how to correctly perform a behaviour e.g., face-to-face as in a group class or using video. |
| BCT 10 | Prompt specific goal setting | Involves detailed planning of what the person will do including, at least, a very specific definition of the behaviour e.g., frequency (such as how many times a day/week), intensity (e.g., sped) or duration (e.g., for how long for). In addition, at least one of the following contexts i.e., where, when, how or with whom must be specified. This could include identification of sub-goals or preparatory behaviours and/or specific contexts in which the behaviour will be performed. |
| BCT 11 | Prompt review of behavioural goals | Involves reconsideration of previously set goals/ intentions. In most cases this will follow previous goal setting and an attempt to act on those goals. |
| BCT 12 | Prompt self-monitoring of behaviour | The person is asked to keep a record of specified behaviour/s. This could e.g., take the form of a diary or completing a questionnaire about their behaviour. |
| BCT 13 | Provide feedback on performance | This involves either receiving data about recorded behaviour or commenting on how well or badly a person has performed an action, or a discrepancy in relation to the performance of others. |
| BCT 14 | Provide contingent rewards | This can include praise and encouragement as well as material rewards but the reward/ incentive must be explicitly linked to the achievement of specified goals i.e. the person receives the reward if they perform the specified behaviour (or preparatory behaviour) but not if they do not perform the behaviour. |
| BCT 15 | Teach to use prompts/ cues | Teach the person to identify environmental prompts which can be used to remind them to perform the behaviour. This could include times of day, particular contexts or elements of contexts which prompt them to perform the target behaviour. |
| BCT 16 | Agree behavioural contract | Must involve agreement (e.g., signing) of an explicitly specifying behaviour so that there is a written record of the person’s resolution witnessed by another. |
| BCT 17 | Prompt practice | Prompt the person to rehearse and repeat the behaviour or preparatory behaviours numerous times. Note this will also include parts of the behaviour e.g., refusal skills in relation to quitting smoking. This could be described as “building habits or routines” but is still practice so long as the person is prompted to try the behaviour (or parts of it) during the intervention. |
| BCT 18 | Use of follow up prompts | Involves sending letters, making telephone calls, visits or follow up meetings after the major part to the behaviour change intervention has been completed. If spaced contacts is an intrinsic part of the behaviour change intervention these in themselves do not count as follow up. |
| BCT 19 | Provide opportunities for social comparison | This will most commonly be seen in the case of group practice (e.g., group classes) but could also be employed using detailed case studies in text or video or by pairing people as supports. It provides a setting in which processes such as social comparison could occur. |
| BCT 20 | Plan social support/ social change | Involves prompting the person to think about how others’ could change their behaviour to offer him/her help and/or (instrumental) social support. This will also include provision of such support during the interventions e.g., setting up a “buddy” system or other forms of support. |
| BCT 21 | Prompt identification as role model/ position advocate | Involves focusing on how the person may be an example to others and affect their behaviour e.g., being a good example to children. Also includes providing opportunities for participants to persuade others of the importance of adopting/ changing the behaviour. For example, giving a talk or writing a persuasive leaflet. |
| BCT 22 | Prompt Self talk | Encourage the person to use talk to themselves (aloud or silently) before and during planned behaviours to encourage and support action. |
| BCT 23 | Relapse prevention | Following an initial change help the person identify situations that increase the likelihood of returning to a risk behaviour or failing to perform a new health behaviour – and help them plan how to avoid or manage the situation so that new behavioural routines are maintained. |
| BCT 24 | Stress management | This may involve a variety of specific techniques (e.g., progressive relaxation) which do not target the behaviour directly but seek to reduce anxiety and stress to facilitate the performance of the behaviour. |
| BCT 25 | Motivational interviewing | This is a specific set of techniques involving prompting the person to provide self-motivating statements and evaluations of own behaviour to minimise resistance to change (includes motivational counselling). |
| BCT 26 | Time management | This includes any technique designed to help a person make time for the behaviour (e.g., how to fit it into a daily or weekly schedule). These techniques are not directed towards performance of target behaviour but rather seek to facilitate it by freeing up times when it could be performed. This technique may or may not be mentioned by name. |
| BCT 27* | Prompt anticipated regret | Involves inducing expectations of future regret about the performance or non-performance of behaviour. This includes focusing on how the person will *feel* in the future and specifically whether they will feel regret or feel sorry that they did or did not take a different course of action. |
| BCT 28* | Use of imagery | Teach the person to use images of performing the behaviour in situations conductive to success. For example, this could include practicing bringing to mind images of succeeding with the task or finding it easy to perform the behaviour, and be conducted in graded fashion, starting with component or easy versions of the behaviour. |
| BCT 29* | Environmental re-structuring | The person is instructed or shown how to alter the environment in ways to support the behaviour e.g. altering cues or reinforcers. For example, they might be asked to destroy all their cigarettes or all their high calorie snacks, or take their running clothes to work. |
| BCT 30* | Homework | Set homework tasks. |
| BCT 31* | Planning implementation | Identify component parts of behaviour and make plan to execute each  one or consider when and/or where a behaviour will be performed, i.e. schedule behaviours. |
| BCT 32* | Coping planning | Identify and plan ways of overcoming barriers (note, this must include identification of specific barriers. For example, “problem-solving how to fit into  weekly schedule” would not count). |
| BCT 33* | Monitoring | Record specified behaviour. Person has access to recorded data of  behavioural performance. For example, from diary. |
| BCT 34* | Persuasive communication | Verbal persuasion/persuasive communication: credible source presents arguments in favour of the behaviour. |
| BCT 35* | Increasing skills | Problem solving, decision making, goal setting. |
| BCT 36* | Social support (emotional) | Others listen, provide empathy and give generalized positive feedback. |
| BCT 37* | Decision making | Generate alternative courses of action, and pros and cons of each, and weigh them up. |
| BCT 38* | Implementation intentions | Planning the when, where, and how of initiating goal-directed behaviours. |
| BCT 39* | Collaborative implementation intentions | Two people planning when and where they will perform the behavior together. |
| BCT 40* | Behavioural rehearsal | Perform behaviour (repeatedly). |

Note: * = Additional behaviour change technique definitions (Abraham & Michie; 2008; Michie et al, 2008; Gollwitzer, 1993; Prestwich et al, 2012)

**Please note * = Behaviour Change Technique coding for intervention additional resources; PN= Postnatal; AN= Antenatal**

**Table 2. Mapping HAPPY Intervention against Antenatal/Postnatal Diet Performance Objectives**

**Desired Outcome: Mothers make antenatal healthy food choices and maintain a healthy diet postnatally (according to UK guidelines)**

PERFORMANCE OBJECTIVES

1 = Mother makes healthy food choices for herself

2 = Mother makes healthy food choices for her unborn baby

3 = Mother increases consumption of fruit and vegetables for herself

4 = Mother reduces the consumption of high-calorie, energy-dense foods and drinks for herself

5 = Mother copes with problems faced with eating a healthy diet

| **Change Objectives and Theoretical Domains Framework area(s)** | **Antenatal Diet Performance Objectives** | **Session Number** | **Content** | **Behaviour Change Techniques** | **Manual Mapping** |
| --- | --- | --- | --- | --- | --- |
| Develops an understanding about what a healthy diet for pregnancy should consist of (Knowledge)  Develops and understanding of what food is good for the baby (Knowledge)  Develops an understanding of why nutritious food is good for the unborn baby (Knowledge)  Acknowledges the short term gains of eating healthily (Beliefs about Consequences)  Acknowledges that eating nutritious food will impact on the unborn baby (Beliefs about Consequences) | PO1, PO2  PO2  PO2  PO1, PO2, PO3  PO2, PO3 | Session 1 AN | Information about a healthy diet in pregnancy (including foods to avoid); links between what you eat when pregnant, you and your baby; link between maternal and childhood obesity  Time to spend reading the information they have been given | BCT 1*; BCT 2*  BCT 30 | P14-17  Additional resources: [Eating Well in Pregnancy hand-out](https://dl-web.dropbox.com/get/BIB%20WORK%20FOR%20NAT/Final%20folder%20for%20submission/Happy%20Intervention%20Table%20and%20Handouts/Eating%20well%20in%20pregnancy%20handout%20.pdf); [Pregnancy Eating and Drinking Facts -Quiz hand-out](https://dl-web.dropbox.com/get/BIB%20WORK%20FOR%20NAT/Final%20folder%20for%20submission/Happy%20Intervention%20Table%20and%20Handouts/Pregnancy%20eating%20and%20drinking%20facts%20quiz%20handout.doc)  P24 |
| **Change Objectives and Theoretical Domains Framework area(s)** | **Antenatal Diet Performance Objectives** | **Session Number** | **Content** | **Behaviour Change Techniques** | **Manual Mapping** |
| Increases motivation to eat healthy foods (Motivation and Goals) | PO1, PO2, PO3 | Session 1 AN | Reflect on own diet and importance of food in the family | BCT 5 | P14 |
| Increases motivation to eat healthy foods (Motivation and Goals)  Develops and understanding about why nutritious food is good for the unborn baby (Knowledge)  Develops an understanding about what a healthy weight gain should be (Knowledge) | PO1, PO2, PO3  PO2  PO1, PO3, PO4 | Session 2 AN | Importance of eating well for the baby  Dispel myths about weight gain in pregnancy and provide up to date information | BCT 2*; BCT 8*  BCT 1*; BCT 2* | P30-38  Additional resources: [Tips for Coping with Common Food and Health Problems in Pregnancy hand-out](https://dl-web.dropbox.com/get/BIB%20WORK%20FOR%20NAT/Final%20folder%20for%20submission/Happy%20Intervention%20Table%20and%20Handouts/Tips%20for%20coping%20with%20common%20food%20and%20health%20problems%20in%20pregnancy%20handout.docx)  P33-35  Additional resources: [Pregnancy and Health Myth Buster hand-out](https://dl-web.dropbox.com/get/BIB%20WORK%20FOR%20NAT/Final%20folder%20for%20submission/Happy%20Intervention%20Table%20and%20Handouts/Pregnancy%20and%20health%20myth%20buster%20handout.doc) |
| **Change Objectives and Theoretical Domains Framework area(s)** | **Antenatal Diet Performance Objectives** | **Session Number** | **Content** | **Behaviour Change Techniques** | **Manual Mapping** |
| Learns how to resist unhealthy foods when upset or stressed (Emotion)  Improves ability to recollect what they have actually eaten (Memory, Attention, and Decision Processes) | PO4, PO5  PO5 | Session 2 AN | Smart Snacks hand-out  Food diaries at the end of the session | BCT 1*; BCT 8*  BCT 12*; BCT 5*; BCT 24* | P38  Additional resources: [Smart Snacks hand-out](https://dl-web.dropbox.com/get/BIB%20WORK%20FOR%20NAT/Final%20folder%20for%20submission/Happy%20Intervention%20Table%20and%20Handouts/Smart%20snacks%20handout.docx)  Additional resources: [How am I Doing Diary hand-out](file:///C:\Documents%20and%20Settings\ps06gl\My%20Documents\My%20Dropbox\BIB%20WORK%20FOR%20NAT\Final%20folder%20for%20submission\Happy%20Intervention%20Table%20and%20Handouts\How%20am%20I%20doing%20diary%20handout.doc); Memory Book |
| Increases motivation to eat healthy foods (Motivation and Goals)  Recognises alternatives for when unhealthy foods are more convenient (Environmental Context and Resources) | PO1, PO2, PO3  PO1, PO3, PO4, PO5 | Session 3 AN | Address barriers to healthy eating and plan for ways to overcome them | BCT 5 | P53-55 |
| Improves confidence to face task (Beliefs about Capabilities)  Develops skills to cook a healthy meal from scratch (Skills) | PO5  PO1, PO2, PO3 |  | Sign post to cooking information points | BCT 8; BCT 29 | P53-56 |
| Recognises alternatives for when unhealthy foods are more convenient (Environmental Context and Resources) | PO1, PO2, PO3, PO4, PO5 |  | Information about how healthy foods can be convenient and inexpensive | BCT 1 | P54 |
| **Change Objectives and Theoretical Domains Framework area(s)** | **Antenatal Diet Performance Objectives** | **Session Number** | **Content** | **Behaviour Change Techniques** | **Manual Mapping** |
| Recognises alternatives for when unhealthy foods are more convenient (Environmental Context and Resources) | PO1, PO2, PO3, PO4, PO5 | Session 3 AN | Impact of mother eating unhealthy foods  Two-three changes | BCT 2  BCT 4 | P56-57  P55 |
| Learns how to resist unhealthy foods when upset or stressed (Emotion)  Improves ability to recollect what they have actually eaten (Memory, Attention, and Decision Processes) | PO4, PO5  PO5 |  | Self-discussion and feedback – food, pa and mood diaries at the end of the session | BCT 12 | P65 |
| Overcomes tradition/culture pressures which encourage using fatty foods such as butter, egg, milk, ghee (Social Influences)  Overcomes pressure to eat/cook high fat foods (Social Influences) | PO4, PO5  PO4, PO5 |  | Plan alternative cooking methods  Healthy Eating Choices hand-out | BCT 31  BCT 4*; BCT 15* | P56  Additional resources: [Healthy Eating Choices hand-out](file:///C:\Documents%20and%20Settings\ps06gl\My%20Documents\My%20Dropbox\BIB%20WORK%20FOR%20NAT\Final%20folder%20for%20submission\Happy%20Intervention%20Table%20and%20Handouts\Healthy%20eating%20choices%20handout.doc) |
| Overcomes pressure from other family members who may be a bad influence; rest of family don’t want healthy foods e.g. often South Asian women won’t change the way they prepare the food as their husbands and other family members won’t like it (Social Influences) | PO1, PO4 |  | Plan a healthy meal. Then feedback to group about it next week | BCT 19; BCT 31 | P65 |
| Learns how to resist unhealthy foods when upset or stressed (Emotion) | PO4, PO5 | Session 4 AN | Additional information about Snacks, food treats, swaps etc | BCT 1; BCT 8; BCT 31 | P77 |
|  |  |  |  |  |  |
| **Change Objectives and Theoretical Domains Framework area(s)** | **Antenatal Diet Performance Objectives** | **Session Number** | **Content** | **Behaviour Change Techniques** | **Manual Mapping** |
| Overcomes tradition/culture pressures which encourage using fatty foods such as butter, egg, milk, ghee (Social Influences)  Overcomes pressure to eat/cook high fat foods (Social Influences)  Overcomes pressure from other family members who may be a bad influence; rest of family don’t want healthy foods e.g. often South Asian women won’t change the way they prepare the food as their husbands and other family members won’t like it (Social Influences) | PO4, PO5  PO1, PO2, PO4  PO1, PO4 | Session 5 AN | Food swaps and healthy meals feedback | BCT 12; BCT 19; BCT 31 | P68, P77 |
| Learns how to resist unhealthy foods when upset or stressed (Emotion)  Increases motivation to make lifestyle changes and not revert back to old ways (Motivation and Goals) | PO4, PO5  PO1, PO2, PO3, PO5 |  | Identify the times when you’ll want to eat more because tired, stressed and have contingencies for when want unhealthy foods  Mingle activity – small changes big difference | BCT 13; BCT 12; BCT 8; BCT 2; BCT 32  BCT 15; BCT 4 | P91  P100-P101 |
| **Change Objectives and Theoretical Domains Framework area(s)** | **Antenatal Diet Performance Objectives** | **Session Number** | **Content** | **Behaviour Change Techniques** | **Manual Mapping** |
| Overcomes having no-one to consult with, or to discuss problems with, etc. (Social Influences)  Learns how to resist unhealthy foods when upset or stressed (Emotion)  Recognises alternatives for when unhealthy foods are more convenient (Environmental Context and Resources) | PO5  PO4, PO5  PO1, PO2, PO3, PO4, PO5 | Session 6 AN | Identify someone at home/friend to discuss food with.  Looking after mum  Planning ahead | BCT 20; BCT 4 | P107 |
| **Change Objectives and Theoretical Domains Framework area(s)** | **Postnatal Diet Performance Objectives** | **Session Number** | **Content** | **Behaviour Change Techniques** | **Manual Mapping** |
| Develops an understanding about what a healthy diet for post-pregnancy should consist of (Knowledge)  Learns how to resist unhealthy foods when upset or stressed (Emotion)  Improves ability to recollect what they have actually eaten (Memory, Attention, and Decision Processes) | PO1, PO3  PO4, PO5  PO5 | Session 1 PN | Information about what a healthy diet should consist of (refers to hand-outs from AN sessions 2 and 3- smart snacks and healthy eating choices)  Food diaries at the end of the session | BCT 1; BCT 2; BCT 12 | P24-26 |
| Develops an understanding about what a healthy diet for post-pregnancy should consist of (Knowledge)  Learns how to resist unhealthy foods when upset or stressed (Emotion)  Improves ability to recollect what they have actually eaten (Memory, Attention, and Decision Processes) | PO1, PO3  PO4, PO5  PO5 |  | Reflect on own diet and importance of food in the family | BCT 5 | P24 |
| **Change Objectives and Theoretical Domains Framework area(s)** | **Postnatal Diet Performance Objectives** | **Session Number** | **Content** | **Behaviour Change Techniques** | **Manual Mapping** |
| Increases motivation to eat healthy foods (Motivation and Goals) | PO1, PO2, PO3 | Session 2 PN | Healthy eating for parents vs children. Advice on making sure mum eats right and child eats right. Reflect on own eating patterns and provide ideas  The Big Balancing Act hand-out  Tempted by Takeaways hand-out  Start 4 Life hand-out-Introducing Solid Foods | BCT 1*; BCT 2*; BCT 6; BCT 5; BCT 8*  BCT 1*; BCT 2*; BCT 8*  BCT 8*  BCT 1*; BCT 2*; BCT 8* | P47-49  Additional resources: [The Big Balancing Act hand-out](file:///C:\Documents%20and%20Settings\ps06gl\My%20Documents\My%20Dropbox\BIB%20WORK%20FOR%20NAT\Final%20folder%20for%20submission\Happy%20Intervention%20Table%20and%20Handouts\The%20BIG%20balancing%20act%20handout.doc);  [Tempted by Takeaways hand-out](file:///C:\Documents%20and%20Settings\ps06gl\My%20Documents\My%20Dropbox\BIB%20WORK%20FOR%20NAT\Final%20folder%20for%20submission\Happy%20Intervention%20Table%20and%20Handouts\Tempted%20by%20takeaways%20handout.doc); [Start 4 Life hand-out-Introducing Solid Foods](file:///C:\Documents%20and%20Settings\ps06gl\My%20Documents\My%20Dropbox\BIB%20WORK%20FOR%20NAT\Final%20folder%20for%20submission\Happy%20Intervention%20Table%20and%20Handouts\Introducing%20solid%20foods%20handout%20Start%204%20Life.pdf) |
| Improves confidence to cook healthy meals (Beliefs about Capabilities) | PO5 | Session 4 PN | Feeding a family – what does it take (identification of current skills and problem solving) | BCT 5; BCT 19; BCT 33; BCT 35 | P77-80 |
| Improves confidence to cook healthy meals (Beliefs about Capabilities) | PO5 |  | Cook a healthy meal and report outcomes | BCT 4 | P81 |
| Develops skills to cook a meal from scratch (Skills) | PO1, PO2, PO3 |  | Ask for feedback off family | BCT 12; BCT 20 | P81 |
| **Change Objectives and Theoretical Domains Framework area(s)** | **Postnatal Diet Performance Objectives** | **Session Number** | **Content** | **Behaviour Change Techniques** | **Manual Mapping** |
| Improves confidence to face task (Beliefs about Capabilities)  Develops skills to cook a healthy meal from scratch (Skills)  Improves motivation to cook a healthy meal from scratch (Motivation and Goals)  Develops an ability to plan ahead for healthy meals (Behavioural Regulation) | PO5  PO1, PO2, PO3  PO1, PO2, PO3  PO5 | Session 4 PN | Break providing healthy meals into chunks: meal planning; shopping lists etc | BCT 12*; BCT 8*; BCT 31* | P78-81  Additional resources: [Happy Meal Planner hand-out](file:///C:\Documents%20and%20Settings\ps06gl\My%20Documents\My%20Dropbox\BIB%20WORK%20FOR%20NAT\Final%20folder%20for%20submission\Happy%20Intervention%20Table%20and%20Handouts\HAPPY%20meal%20planner%20handout.doc); [Happy Shopping List hand-out](file:///C:\Documents%20and%20Settings\ps06gl\My%20Documents\My%20Dropbox\BIB%20WORK%20FOR%20NAT\Final%20folder%20for%20submission\Happy%20Intervention%20Table%20and%20Handouts\HAPPY%20shopping%20list%20handout.doc) |
| Improves confidence to face task (Beliefs about Capabilities)  Develops skills to cook a healthy meal from scratch (Skills) | PO5  PO1, PO2, PO3 |  | Information about how healthy foods can be convenient and inexpensive  Reading food labels | BCT 1*; BCT 8* | P78-81  Additional resources: [Family Food- the HAPPY Guide to Getting Organised hand-out](file:///C:\Documents%20and%20Settings\ps06gl\My%20Documents\My%20Dropbox\BIB%20WORK%20FOR%20NAT\Final%20folder%20for%20submission\Happy%20Intervention%20Table%20and%20Handouts\Family%20food%20the%20HAPPY%20guide%20to%20getting%20organised%20handout.pub) |
| Recognises alternatives for when unhealthy foods are more convenient (Environmental Context and Resources) | PO1, PO2, PO3, PO4, PO5 |  | Impact of mother eating unhealthy foods  You are being Watched hand-out | BCT 2  BCT 1*; BCT 2*; BCT 8* | P77-78  Additional resources: [You are being Watched hand-out](file:///C:\Documents%20and%20Settings\ps06gl\My%20Documents\My%20Dropbox\BIB%20WORK%20FOR%20NAT\Final%20folder%20for%20submission\Happy%20Intervention%20Table%20and%20Handouts\You%20are%20being%20watched%20handout.pub) |
| Recognises alternatives for when unhealthy foods are more convenient (Environmental Context and Resources) | PO1, PO2, PO3, PO4, PO5 |  | Self-discussion and feedback – Food, physical activity and mood diaries at the end of the session | BCT 12 | P81 |
| **Change Objectives and Theoretical Domains Framework area(s)** | **Postnatal Diet Performance Objectives** | **Session Number** | **Content** | **Behaviour Change Techniques** | **Manual Mapping** |
| Learns how to resist unhealthy foods when upset or stressed (Emotion)  Improves ability to recollect what they have actually eaten (Memory, Attention, and Decision Processes) | PO4, PO5  PO5 | Session 4 PN | Plan food swaps or alternative cooking methods  Making Meals Healthier hand-out | BCT 31  BCT 8* | P80-81  Additional resources: [Making Meals Healthier hand-out](file:///C:\Documents%20and%20Settings\ps06gl\My%20Documents\My%20Dropbox\BIB%20WORK%20FOR%20NAT\Final%20folder%20for%20submission\Happy%20Intervention%20Table%20and%20Handouts\Making%20meals%20healthier%20handout.doc) |

**Table 3. Mapping HAPPY Intervention against Physical Activity during and after Pregnancy Performance Objectives**

**Desired Outcome: Mother increases physical activity during pregnancy and meets the guidelines of 150 minutes moderate intensity exercise per week by six months postnatal**

PERFORMANCE OBJECTIVES

1 = Mother meets the recommended guidelines of 150 minutes moderate PA/wk (can be done in 10 minute bouts) during and after pregnancy

2 = Mother performs physical activities that are safe during pregnancy

3 = Mother tries new physical activities during and after pregnancy

4 = Mother resists pressure from family/friends not to do PA during or after pregnancy

5 = Mother copes with problems faced with doing PA during or after pregnancy

| **Change Objectives and Theoretical Domains Framework area(s)** | **Antenatal Physical Activity Performance Objectives** | **Session Number** | **Content** | **Behaviour Change Techniques** | **Manual Mapping** |
| --- | --- | --- | --- | --- | --- |
| Develops an understanding about what constitutes physical activity and the guidelines for physical activity in pregnancy/postnatal **(**Knowledge)  Acknowledges the importance of physical activity for the baby in pregnancy and postnatal (Beliefs about Consequences) | PO1, PO2, PO3  PO2, PO3  PO4 | Session 1 AN | How the baby is developing in the womb and how physical activity can facilitate this  Time to spend reading the information they have been given | BCT 1*; BCT 2*; BCT 8; BCT 10; BCT 14*; BCT 20*; BCT 29*  BCT 30 | P18  Additional resources:  [Tommy’s Managing Your Weight in Pregnancy hand-out](file:///E:\Happy%20Intervention%20Table%20and%20Handouts\Managing%20Your%20Weight%20in%20Pregnancy%20handout%20Tommy's%20.doc)  P24 |
| Reduces worry about potential harm to baby from PA by developing understanding about the safe levels of PA during pregnancy **(**Knowledge and Beliefs about Consequences) |  |  |  |  |  |
| **Change Objectives and Theoretical Domains Framework area(s)** | **Antenatal Physical Activity Performance Objectives** | **Session Number** | **Content** | **Behaviour Change Techniques** | **Manual Mapping** |
| Develops an understanding of consistent and correct PA information from health professionals (Knowledge) | PO1, PO2, PO3 | Session 2 AN | Being Active during Pregnancy hand-out | BCT 1*; BCT 8* | P37  Additional resources: [Being Active during Pregnancy hand-out](file:///C:\Documents%20and%20Settings\ps06gl\My%20Documents\My%20Dropbox\BIB%20WORK%20FOR%20NAT\Final%20folder%20for%20submission\Happy%20Intervention%20Table%20and%20Handouts\Being%20active%20during%20pregnancy%20handout.pdf) |
| Develops an understanding of what physical activity is safe during pregnancy (Knowledge) | PO2, PO4 |  | Discussion regarding myths about physical activity in pregnancy; pros and cons; practitioner dispels myths  Discussion with family members dispelling physical activity danger myths and encouraging them to support mother in physical activity behaviour | BCT 1; BCT 2; BCT 34  BCT 6; BCT 20 | P33-37 |
| Develops an understanding of what activities are available to them and types of appropriate activities (Knowledge) | PO1, PO2, PO3, PO4 |  |  |  |  |
| Acknowledges the importance of physical activity for themselves in pregnancy and postnatal (Knowledge, Beliefs and Consequences)  Learns that physical activity can increase energy levels (Emotion) | PO2, PO3  PO1, PO5 |  |  |  |  |
| **Change Objectives and Theoretical Domains Framework area(s)** | **Antenatal Physical Activity Performance Objectives** | **Session Number** | **Content** | **Behaviour Change Techniques** | **Manual Mapping** |
| Overcomes social expectation that pregnant women should not exercise (Social Influences) | PO4 | Session 2 AN |  |  | P33-37 |
| Overcomes information from media that is conflicting and impersonal (Social Influences) | PO4 |  |  |  |  |
| Overcomes conflict of advice from family members (Social Influences) | PO4, PO5 |  |  |  |  |
| Improves confidence that what they are doing is right (Beliefs about Capabilities) | PO1, PO2, PO3, PO4, PO5 | Session 3 AN | List activities women think they could do, work through easy to difficult (decide on activity, make deal in pairs | BCT 7; BCT 19; BCT 39 | P50 |
| Develops skills to engage in physical activity e.g. swimming (Skills)  Learns that physical activity can increase energy levels (Emotion) | PO1, PO2, PO3  PO1, PO5 |  | Gentle strengthening and conditioning exercises: e.g. pregnancy yoga, easy introduction and demonstration by practitioners  List activities women think they could do and then work through easy to difficult  Set up homework task: do chosen activity and report back next week | BCT 9; BCT 8*;  BCT 14; BCT 35  BCT 4; BCT 19; BCT 20  BCT 30 | P41-43  Additional resources: [Physical Activity Ball for Adults hand-out](file:///C:\Documents%20and%20Settings\ps06gl\My%20Documents\My%20Dropbox\BIB%20WORK%20FOR%20NAT\Final%20folder%20for%20submission\Happy%20Intervention%20Table%20and%20Handouts\My%2030%20minute%20physical%20activity%20ball%20handout.pub)  P50  P50 |
| **Change Objectives and Theoretical Domains Framework area(s)** | **Antenatal Physical Activity Performance Objectives** | **Session Number** | **Content** | **Behaviour Change Techniques** | **Manual Mapping** |
|  |  | Session 3 AN | Change places if sentences- physical activity | BCT 13; BCT 19 | P43 |
| Improves self-confidence due to size (Beliefs about Capabilities)  Develops skills to engage in physical activity e.g. swimming (Skills)  Recognises alternatives for when there is a lack of facilities, pre-/postnatal classes, and no facilities with childcare (Environmental Context and Resources)  Develops an ability to prioritise PA as well as other day-to-day tasks (Behavioural Regulation) | PO1, PO3, PO5  PO1, PO2, PO3  PO1, PO2, PO3, PO5  PO1, PO3, PO5 |  | Worries about physical activity. Also reinforced in Postnatal Session 2  Visualize themselves doing physical activity and positive self-talk into doing physical activity  Identify points in time whereby activity can be freely integrated into normal life | BCT 24; BCT 32;  BCT 28; BCT 36  BCT 22; BCT 28  BCT 26; BCT 29 | P49  P51  P50 |
| Develops an ability to plan ahead when experiencing feelings of sickness (Emotion; Behavioural Regulation) | PO1, PO2, PO3, PO5 | Session 4 AN | If planned to do some activity but something gets in the way, e.g. morning sickness, doctor’s appointment etc., then make sure there is a back-up plan for a different time | BCT 35 | P79 |
|  |  |  |  |  |  |
| **Change Objectives and Theoretical Domains Framework area(s)** | **Antenatal Physical Activity Performance Objectives** | **Session Number** | **Content** | **Behaviour Change Techniques** | **Manual Mapping** |
| Improves motivation to engage in psychical activity (Motivation and Goals) | PO1, PO2, PO3 | Session 5 AN | Partners review their progress for the collaborative plans made for pa in AN Session 4; discussion about their feelings relating to whether they fulfilled their activity plans | BCT 12; BCT 27 | P84 |
| Increases motivation to do PA with family/child (Motivation and Goals)  Develops an ability to prioritise day-to-day tasks (Behavioural Regulation) | PO1, PO3  PO1, PO3, PO5 |  |  |  |  |
| Develops skills to engage in physical activity e.g. swimming (Skills) | PO1, PO2, PO3 | Session 6 AN | Pelvic floor exercises for immediately after pregnancy if uncomplicated; provide leaflet with activity examples. Also in PN Session 1 | BCT 1*; BCT 2*; BCT 8*; BCT 9 | P111  Additional resources: [Physical Activity for New Mums hand-out](https://dl-web.dropbox.com/get/BIB%20WORK%20FOR%20NAT/Final%20folder%20for%20submission/Happy%20Intervention%20Table%20and%20Handouts/Physical%20activity%20for%20new%20mums%20handout.pdf) |
| Develops an understanding of consistent and correct PA information from health professionals (Knowledge) | PO1, PO2, PO3 |  | What activities are okay for after pregnancy, e.g. pelvic floor exercises to reduce  urinary stress incontinence: see skills for rehearsal. Also in PN Sessions 1 and 2 | BCT 1 | P110-112 |
|  |  |  |  |  |  |

| **Change Objectives and Theoretical Domains Framework area(s)** | **Postnatal Physical Activity Performance Objectives** | **Session Number** | **Content** | **Behaviour Change Techniques** | **Manual Mapping** |
| --- | --- | --- | --- | --- | --- |
| Develops an understanding of consistent and correct PA information from health professionals (Knowledge) | PO1, PO2, PO3 | Session 1 PN | What activities are okay for after pregnancy, e.g. pelvic floor exercises to reduce urinary stress incontinence: see skills for rehearsal. Gradually introduce more strenuous activity | BCT 1 | P23-25 |
| Develops skills to engage in physical activity e.g. swimming (Skills) | PO1, PO2, PO3 |  | Pelvic floor exercises for immediately after pregnancy if uncomplicated; provide leaflet with activity examples | BCT 9; BCT 8 | P23-25 |
| Learns that physical activity can increase energy levels (Emotion) | PO1, PO5 |  | Physical Activity for New Mums quiz sheet hand-out  Practitioner dispels myths | BCT 1*; BCT 2*; BCT 34* | P23  Additional resources: [Physical Activity for New Mums quiz sheet hand-out](file:///C:\Documents%20and%20Settings\ps06gl\My%20Documents\My%20Dropbox\BIB%20WORK%20FOR%20NAT\Final%20folder%20for%20submission\Happy%20Intervention%20Table%20and%20Handouts\Physical%20activity%20for%20new%20mums%20quick%20quiz%20handout.pdf) |
| Develops an understanding of consistent and correct PA information from health professionals (Knowledge) | PO1, PO2, PO3 | Session 2 PN | Create flipchart of likes and dislikes about physical activity. Reiterate information regarding physical activity, what activities are okay for after pregnancy, e.g., pelvic floor to reduce urinary stress incontinence, gradually introduce of more strenuous activity. | BCT 1 | P35-38 |
| **Change Objectives and Theoretical Domains Framework area(s)** | **Postnatal Physical Activity Performance Objectives** | **Session Number** | **Content** | **Behaviour Change Techniques** | **Manual Mapping** |
| Increases motivation to spend time with family/child (Motivation and Goals)  Develops an ability to prioritise PA as well as other day-to-day tasks (Behavioural Regulation) | PO1, PO3  PO1, PO3, PO5 | Session 2 PN | Identify barriers to completing physical activity.  Identify points in time where activity can be integrated into normal life. Practitioner facilitates discussion of small changes to family environment | BCT 5; BCT 15; BCT 29 | P35-38 |
| Recognises alternatives for when they feel the neighbourhood is unsafe (Environmental Context and Resources)  Recognises alternatives for when they have no money for classes/equipment (Environmental Context and Resources)  Recognises alternatives for when the weather is too hot/cold/rainy/snowy to go out (Environmental Context and Resources)  Recognises alternatives for when there is a lack of facilities, pre-/postnatal classes, and no facilities with childcare (Environmental Context and Resources) | PO1, PO5 |  | What structured activities could be done in the house; e.g. yoga DVD, aerobics DVD; go for a walk with husband/partner/family member /member of HAPPY group; make a list of what the local area has to offer – practitioners will need to have pre-made list of these for the areas which the mothers come from | BCT 29; BCT 20; BCT 8; BCT 35 | P38-41 |
| Improves self-confidence due to size (Beliefs about Capabilities) | PO1, PO3, PO5 |  | Mum chooses a physical activity uses self talk and visualization to feel confident about performing it | BCT 4; BCT 19; BCT 24; BCT 32 | P37- 38 |
| **Change Objectives and Theoretical Domains Framework area(s)** | **Postnatal Physical Activity Performance Objectives** | **Session Number** | **Content** | **Behaviour Change Techniques** | **Manual Mapping** |
| Develops skills to engage in physical activity e.g. swimming (Skills) | PO1, PO2, PO3 | Session 2 PN | Mum is encouraged to choose a physical activity that she is willing to learn or can already do | BCT 7 | P37-38 |
| Increases motivation to engage in physical activity (Motivation and Goals) | PO1, PO3 | Session 3 PN | Barrier identification; coping and planning. Identify ways of overcoming the barriers faced ensure time is made for physical activity  Introduction to the ‘Pram Pedometer Challenge’ | BCT 5  BCT 19; BCT 20; BCT 6; BCT 10; BCT 14; BCT 12; BCT 11; BCT 39* | P56-58  Additional resources: [Pram Pedometer Challenge hand-out](file:///C:\Documents%20and%20Settings\ps06gl\My%20Documents\My%20Dropbox\BIB%20WORK%20FOR%20NAT\Final%20folder%20for%20submission\Happy%20Intervention%20Table%20and%20Handouts\Pram%20pedometer%20challenge%20steps%20record%20handout.pptx) |
| Improves motivation to engage in physical activity (Motivation and Goals) | PO1, PO3 | Session 4 PN | Review of group progress and setting goals for forthcoming weeks | BCT 19; BCT 20; BCT 6; BCT 10; BCT 14; BCT 12; BCT; BCT 39* | P76 |
| Improves motivation to engage in physical activity (Motivation and Goals) | PO1, PO3 | Session 5 PN | Review of group progress and setting goals for forthcoming weeks | BCT 19; BCT 20; BCT 6; BCT 10; BCT 14; BCT 12; BCT; BCT 39* | P88-89 |
| **Change Objectives and Theoretical Domains Framework area(s)** | **Postnatal Physical Activity Performance Objectives** | **Session Number** | **Content** | **Behaviour Change Techniques** | **Manual Mapping** |
| Improves motivation to engage in physical activity (Motivation and Goals) | PO1, PO3 | Session 6 PN | Review of physical activity achievements individually and group; reflect on good and not so good experiences; plan for physical activity future post-parenting programme – include some follow up monitoring plan by practitioners; announce group winners! | BCT 19; BCT 20; BCT 6; BCT 10; BCT 14; BCT 12; BCT 39* | P107-112 |

**Table 4. Mapping HAPPY Intervention against Breastfeeding Performance Objectives**

**Desired Outcome: Breastfeeding is encouraged until at least six months**

PERFORMACE OBJECTIVES

1 = Mother initiates breastfeeding at birth

2 = Mother exclusively breast feeds (or offers expressed milk) for 6 months

3 = Mother continues to breast feed once solids are introduced

4 = Mother introduces solids at about 6 months

5 = Mother/other guardian(s) uses bottle feed appropriately if this is the preferred feeding choice

6 = Mother copes with problems faced with breastfeeding

| **Change Objectives and Theoretical Domains Framework area(s)** | **Antenatal Breastfeeding Performance Objectives** | **Session Number** | **Content** | **Behaviour Change Techniques** | **Manual Mapping** |
| --- | --- | --- | --- | --- | --- |
| Improves motivation to breastfeed (Motivation and Goals) | PO1 | Session 1 AN | Write down concerns about infant feeding. Session 3 will address these barriers and plan for ways to overcome them | BCT 5 | P18 |
| Develops an understanding about how to breastfeed successfully/how to bottle feed correctly (Knowledge)  Acknowledges that the infant does not need to finish the whole bottle to put on enough weight (Beliefs about Consequences) | PO1, PO2  PO3 | Session 3 AN | Prompt thoughts about advantages/disadvantages of breast and bottle feeding | BCT 1*; BCT 2* | P57- 63  Additional resources: [Advantages and Disadvantages of Breast and Bottle Feeding hand-out](https://dl-web.dropbox.com/get/BIB%20WORK%20FOR%20NAT/Final%20folder%20for%20submission/Happy%20Intervention%20Table%20and%20Handouts/Advantages%20and%20disadvantages%20of%20breast%20and%20bottle%20feeding%20handout.doc) |
| **Change Objectives and Theoretical Domains Framework area(s)** | **Antenatal Breastfeeding Performance Objectives** | **Session Number** | **Content** | **Behaviour Change Techniques** | **Manual Mapping** |
| Acknowledges that they do not need to make baby wait between bottle feeds so they will sleep longer, therefore giving them too much milk in one feed (Beliefs about Consequences)  Overcomes pressure that mothers need to maximize weight gain by feeding child whole bottle (Social Influences)  Discovers ways to ensure formula milk is not wasted rather than force feeding child (Environmental Context and Resources) | PO5 | Session 3 AN | Start 4 Life hand-out - Off to the Best Start (applicable to content throughout session 3) | BCT 1*; BCT 2*; BCT 8*; BCT 20* | P63  Additional resources: [Start 4 Life hand-out - Off to the Best Start](file:///F:\Everything%20else%20pre%20Oz\BIB%20Happy%20Mapping\Final%20HAPPY%20manuscript%20files\Additional%20Files\Off%20to%20the%20best%20start%20handout%20Start%204%20Life.pdf) |
|  |  |  |  |  |  |
| Develops an understanding about how to increase milk supply (Knowledge) | PO1, PO2, PO3 |  | More you feed, more milk produced/more is more message re increasing milk supply | BCT 1 | P57- 62 |
| Improves motivation to breastfeed (Motivation and Goals) | PO1 |  | Persuasive communication: include advantages and disadvantages of breastfeeding and bottle feeding | BCT 1; BCT 2 | P57- 62 |
| Improves confidence to breastfeed at first (Beliefs about Capabilities) | PO1, PO6 |  | Persuasive communication: include advantages and disadvantages of breastfeeding and bottle feeding | BCT 1; BCT 2 | P57- 62 |
| **Change Objectives and Theoretical Domains Framework area(s)** | **Antenatal Breastfeeding Performance Objectives** | **Session Number** | **Content** | **Behaviour Change Techniques** | **Manual Mapping** |
| Improves confidence to increase milk supply (Beliefs about Capabilities) | PO2, PO3, PO4, PO6 | Session 3 AN | More you feed, more milk is produced- “more is more” message re increasing milk supply | BCT 1 | P57- 62 |
| Learns that breastfeeding alone would not lead to babies sleeping for less at night (Beliefs about Consequences) | PO2, PO3, PO4 |  | Prompt thoughts about advantages/disadvantages of breast and bottle feeding | BCT 1; BCT 2 | P57- 62 |
| Improves motivation to breastfeed - lengthy hours spent breastfeeding (Motivation and Goals) | PO2, PO3 |  | Prompt thoughts about advantages/disadvantages of breast and bottle feeding | BCT 1; BCT 2 | P57- 62 |
| Reduces feelings of worry that child is not happy breastfeeding (reduce feelings of inadequacy and failure if think not producing enough milk, and feeling as though child needs something ‘more’ than they can provide) (Emotion) | PO2, PO3, PO4, PO6 |  | More you feed, more milk produced/more is more message re increasing milk supply | BCT 1; BCT 22 | P57- 62 |
| Overcomes difficulties regarding telling relatives that breastfeeding is adequate and there is no need for additional food/milk (Social Influences) | PO1, PO2, PO3, PO6 |  | Encourage identifying influential family member, clarify likes and dislikes and to talk with the family about these issues to enlist their support | BCT 20; BCT 32; BCT 35 | P57-63 |
| **Change Objectives and Theoretical Domains Framework area(s)** | **Antenatal Breastfeeding Performance Objectives** | **Session Number** | **Content** | **Behaviour Change Techniques** | **Manual Mapping** |
| Develops an understanding about how to breastfeed successfully (Knowledge) | PO1, PO2, PO3 | Session 3 AN | Group discussion about difficulties mothers think they will face | BCT 5 | P60 |
| Develops an understanding about how much breast milk is enough (Knowledge) | PO1, PO2, PO3 |  | Group discussion about difficulties mothers think they will face | BCT 5 | P60 |
| Increases motivation to breastfeed (Motivation and Goals) | PO1 |  | Working in a group to learn more about breastfeeding skills. Modelling latching on using pictures. Signposting to relevant medical professionals for further development of skills | BCT 9; BCT 19; BCT 20; BCT 35; BCT 37 | P60 |
| Improves confidence to breastfeed at first (Beliefs about Capabilities)  Develops skills to increase milk supply (Skills) | PO1, PO6  PO2, PO3, PO6 |  | Working in a group to learn more about breastfeeding skills. Modelling latching on using pictures. Signposting to relevant medical professionals for further development of skills | BCT 9; BCT 19; BCT 20; BCT 35; BCT 37 | P60 |
| Develops skills to get the baby to latch on (Skills) | PO1, PO2, PO6 |  | Group discussion about difficulties mothers think they will face | BCT 5 | P60 |
| Learns how sore nipples and breasts following frequent feeds can be managed (Beliefs about Consequences) | PO1, PO2, PO3, PO6 |  | Group discussion about difficulties mothers think they will face | BCT 5 | P60 |
| **Change Objectives and Theoretical Domains Framework area(s)** | **Antenatal Breastfeeding Performance Objectives** | **Session Number** | **Content** | **Behaviour Change Techniques** | **Manual Mapping** |
| Learns that breastfeeding alone would not lead to babies sleeping for less at night (Beliefs about Consequences) | PO2, PO3, PO4 | Session 3 AN | Group discussion about difficulties mothers think they will face | BCT 5 | P60 |
| Recognises alternative for when there is a lack of private places to breastfeed (Environmental Context and Resources) | PO2, PO3, PO4, PO6 |  | Overcome stigma about breastfeeding in public, stories from other mothers | BCT 5; BCT 19 | P60 |
| Improves motivation and develops an ability to plan ahead - time consuming in comparison to bottle feeding (Motivation and Goals; Behavioural Regulation) | PO2, PO3, PO6 |  | Group discussion about difficulties mothers think they will face | BCT 5 | P60 |
| Overcomes pressure from parents and in-laws to introduce formula early to the infant (Social Influences), and reduces feelings of inadequacy and failure (Emotion) | PO2, PO3, PO3, PO6 |  | Group discussion about difficulties mothers think they will face | BCT 5 | P60 |
| Overcomes pressure from mother-in-laws who will take over the care of the babies, because they can give the baby a bottle while the mother is getting on with the chores (Social Influences) | PO2, PO3, PO6 |  | Group discussion about difficulties mothers think they will face | BCT 5 | P60 |
| Overcomes pressure from parents and in-laws to introduce formula early to the infant (Social Influences), and reduces feelings of inadequacy and failure (Emotion) | PO2, PO3, PO3, PO6 |  | Group discussion about difficulties mothers think they will face | BCT 5 | P60 |
| **Change Objectives and Theoretical Domains Framework area(s)** | **Antenatal Breastfeeding Performance Objectives** | **Session Number** | **Content** | **Behaviour Change Techniques** | **Manual Mapping** |
| Overcomes pressure from mother-in-laws who will take over the care of the babies, because they can give the baby a bottle while the mother is getting on with the chores (Social Influences) | PO2, PO3, PO6 | Session 3 AN | Group discussion about difficulties mothers think they will face | BCT 5 | P60 |
| Develops an understanding about how to breastfeed successfully (Knowledge) | PO1, PO2, PO3 | Session 4 AN | Values and Beliefs About Bringing Up Children hand-out | BCT 1*; BCT 8* | Additional resources: [Values and Beliefs About Bringing Up Children hand-out](file:///C:\Documents%20and%20Settings\ps06gl\My%20Documents\My%20Dropbox\BIB%20WORK%20FOR%20NAT\Final%20folder%20for%20submission\Happy%20Intervention%20Table%20and%20Handouts\Values%20and%20beliefs%20about%20bringing%20up%20children%20handout.doc) |
| Develops to make bottle up correctly (Skills) | PO5 | Session 6 AN | Educational tools- how to make up bottle correctly; signpost to health professionals who can demonstrate behavior | BCT 9 | P108 |
| Improves confidence to ask for help (Beliefs about Capabilities; Social Influences) | PO1, PO2, PO6 |  | ‘Don’t be scared to ask for help’: advice about help from midwives, health visitors etc | BCT 19; BCT 20 | P108 |
| Develops an ability to plan ahead and recognises alternative for when breastfeeding restricts movement outside of the house with the infant (Environmental Context and Resources; Behavioural Regulation) | PO2, PO3, PO6 |  | Environmental changes: clothes that facilitate; feeding and locations in Bradford | BCT 20 | P109-109 |
| Overcomes lack of support with housework (Social Influences) | PO2, PO3, PO6 |  | Family members can do other things with baby (than formula feed): e.g. bottle feed using expressed milk | BCT 36 | P108 |
| **Change Objectives and Theoretical Domains Framework area(s)** | **Antenatal Breastfeeding Performance Objectives** | **Session Number** | **Content** | **Behaviour Change Techniques** | **Manual Mapping** |
| Overcomes pressure from husband wanting to pitch in so they can have interaction with child and help out resulting in pressure to bottle feed (Social Influences) | PO2, PO6 | Session 6 AN |  |  | P108 |
| Develops an ability to plan ahead and recognises alternative for when they have to return to work which increases chance of discontinuing breastfeeding (Environmental Context and Resources; Behavioural Regulation) | PO2, PO3, PO4, PO6 |  | Looking after mum - Organising how to deal with life whilst trying to feed/do physical activity etc | BCT 31; BCT 35; BCT 37; BCT 10 | P107 |
| Learns how sore nipples and breasts following frequent feeds can be managed (Beliefs about Consequences) | PO2, PO3, PO4, PO6 |  | Following up progress from session 5 (AN) re. identifying support | BCT 10; BCT 20 | P109 |
| **Change Objectives and Theoretical Domains Framework area(s)** | **Postnatal Breastfeeding Performance Objectives** | **Session Number** | **Content** | **Behaviour Change Techniques** | **Manual Mapping** |
| Improves confidence that what they are doing is right (Beliefs about Capabilities) | PO1, PO3 | Session 1 PN | How is feeding going? | BCT 19; BCT 20; BCT 6; BCT 13 | P14 |
| Develops skills increase milk supply (Skills) | PO2, PO3, PO4 |  | Reinforce more you feed, more milk produced/’more is more message’ re increasing milk supply | BCT 1 | P12-14 |
| Learns how sore nipples and breasts following frequent feeds can be managed (Beliefs about Consequences) | PO1, PO2, PO3, PO6 |  | Group discussion about difficulties mothers think they will face | BCT 1*; BCT 2*; BCT 5; BCT 8* | P14  Additional resources:  [Happy Guide to Feeding your Baby hand-out](file:///C:\Documents%20and%20Settings\ps06gl\My%20Documents\My%20Dropbox\BIB%20WORK%20FOR%20NAT\Final%20folder%20for%20submission\Happy%20Intervention%20Table%20and%20Handouts\HAPPY%20guide%20to%20feeding%20your%20baby%20handout.pub) |
| Learns how sore nipples and breasts following frequent feeds can be managed (Beliefs about Consequences) | PO1, PO2, PO3, PO6 |  | Following up progress from session 5 (AN) re. identifying someone (friend/family/ practitioner) to obtain support from | BCT 10; BCT 20 | P14 |
| Develops skills to respond to babies distress (Skills) | PO1, PO6 |  | Responsive feeding – reminder from session 4 antenatal. Confirm that feeding is not the only way to soothe or show affection | BCT 1; BCT 2;  BCT 8 | P14 |
| **Change Objectives and Theoretical Domains Framework area(s)** | **Postnatal Breastfeeding Performance Objectives** | **Session Number** | **Content** | **Behaviour Change Techniques** | **Manual Mapping** |
| Learns how sore nipples and breasts following frequent feeds can be managed (Beliefs about Consequences) | PO2, PO3, PO4, PO6 | Session 1 PN | Following up progress from session 5 (AN) re. identifying someone (friend/family/practitioner) to obtain support from before/during breastfeeding | BCT 10; BCT 20 | P13 |
| Learns how sore nipples and breasts following frequent feeds can be managed (Beliefs about Consequences) | PO2, PO3, PO4, PO6 | Session 2 PN | Group discussion about difficulties mothers think they will face.  Responding to Concerns about Feeding Baby hand-out | BCT 5  BCT 1*; BCT 8*; BCT 6* | P31-33  Additional resources: [Responding to Concerns about Feeding Baby hand-out](file:///C:\Documents%20and%20Settings\ps06gl\My%20Documents\My%20Dropbox\BIB%20WORK%20FOR%20NAT\Final%20folder%20for%20submission\Happy%20Intervention%20Table%20and%20Handouts\Responding%20to%20concerns%20about%20feeding%20baby%20handout.doc) |
| Develops an ability to plan ahead and recognises alternative for when they have to return to work which increases chance of discontinuing breastfeeding (Environmental Context and Resources; Behavioural Regulation) | PO2, PO3, PO4, PO6 |  | Organising how to deal with life whilst trying to feed | BCT 4; BCT 10; BCT 35; BCT 37 | P32 |
| Overcomes pressure from parents and in-laws to introduce formula early to the infant (Social Influences), and reduces feelings of inadequacy and failure (Emotion) | PO2, PO3, PO4, PO6 |  | Assuming parenting programme covers necessary assertion skills, encourage asking for help in other useful areas to enable breastfeeding to continue and other things to get done | BCT; BCT 20; BCT 32; BCT 35 | P31-34 |
| **Change Objectives and Theoretical Domains Framework area(s)** | **Postnatal Breastfeeding Performance Objectives** | **Session Number** | **Content** | **Behaviour Change Techniques** | **Manual Mapping** |
| Overcomes pressure from parents and in-laws to introduce formula early to the infant (Social Influences), and reduces feelings of inadequacy and failure (Emotion)  Overcomes pressure from mother-in-laws who will take over the care of the babies, because they can give the baby a bottle while the mother is getting on with the chores (Social Influences) | PO2, PO3, PO4, PO6  PO2, PO6 | Session 2 PN | Assuming parenting programme covers necessary assertion skills, encourage asking for help in other useful areas to enable breastfeeding to continue and other things to get done | BCT 19; BCT 20;  BCT 35 | P31-34 |
| Improves confidence that what they are doing is right (Beliefs about Capabilities) | PO1, PO3, PO6 | Session 1-5 | How is feeding going? | BCT 19; BCT 20; BCT 13; BCT 6 | P14, 31, 54, 69, 87 |

**Table 5. Mapping HAPPY Intervention for Physical Activity for Infant Performance Objectives**

**Desired Outcome: Physical activity for infant is facilitated and sedentary time is limited**

PERFORMANCE OBJECTIVES

1 = Mother/other guardian(s) ensures that infant has daily PA interactions in several bouts of both structured and unstructured play across the day.

2 = Mother/other guardian(s) provides a safe, clean floor space large enough for playing, rolling, crawling and other large muscle activities.

3 = Mother/other guardian(s) provide age appropriate equipment which promotes motor skill acquisition

4 = Mother learns about the importance of PA for motor skill development and consequences for later life health

5 = Mother/other guardian(s) encourages and motivates the infant’s PA participation

6 = Mother/other guardian(s) ensures infant is not restrained in highchair/buggy/cot whilst awake for >1hr or watches TV for > 1hr

7 = Mother/other guardian(s) copes with problems faced with ensuring infant has daily PA interactions

| **Change Objectives and Theoretical Domains Framework area(s)** | **Antenatal Physical Activity Performance Objectives** | **Session Number** | **Content** | **Behaviour Change Techniques** | **Manual Mapping** |
| --- | --- | --- | --- | --- | --- |
| N/A | N/A | Session 1 AN | N/A | N/A |  |
| N/A | N/A | Session 2 AN | N/A | N/A |  |
| N/A | N/A | Session 3 AN | N/A | N/A |  |
| N/A | N/A | Session 4 AN | N/A | N/A |  |
| N/A | N/A | Session 5 AN | N/A | N/A |  |
| N/A | N/A | Session 6 AN | N/A | N/A |  |

| **Change Objectives and Theoretical Domains Framework area(s)** | **Postnatal Physical Activity Performance Objectives** | **Session Number** | **Content** | **Behaviour Change Techniques** | **Manual Mapping** |
| --- | --- | --- | --- | --- | --- |
| Develops an understanding about what constitutes physical activity and what the guidelines for physical activity are (Knowledge) | PO1, PO2, PO3 | Session 1 PN | Provide information on guidelines for baby activity | BCT 1 | P14-20 |
| Develops an understanding and acknowledges the importance of motor skill development for later life health (Knowledge; Beliefs about Consequences)  Develops an understanding of the benefits of active play and consequences of sedentary behavior (Knowledge; Beliefs about Consequences)  Overcomes the stereotype that sport is for boys and should not be encouraged in girls (Social Influences) | PO4, PO5  PO1, PO2, PO3, PO4, PO5, PO6  PO1, PO3, PO4, PO5, PO7 |  | Discussion regarding myths about physical activity for babies and infants; pros and cons; practitioner dispels myths – quiz | BCT 1; BCT 2; BCT 5; BCT 34; BCT 36 | P14-20 |
| Improves confidence that what they are doing/giving is right (Beliefs about Capabilities) | PO1, PO3, PO7 |  |  |  |  |
|  |  |  |  |  |  |
| **Change Objectives and Theoretical Domains Framework area(s)** | **Postnatal Physical Activity Performance Objectives** | **Session Number** | **Content** | **Behaviour Change Techniques** | **Manual Mapping** |
| Develops an understanding and skills for playing with her baby in a stimulating way (Knowledge; Skills) | PO1, PO3, PO5 | Session 1 PN | Weekly age appropriate activities for baby and mum is demonstrated and practiced. Mum is given the Happy Book of Play handout. Encourage mum to perform structured and unstructured play – as discussed in ‘time to play’ this week | BCT 1*; BCT 2*; BCT 8*; BCT 9; BCT 30; BCT 12; BCT 13; BCT 36; BCT 35; BCT 40 | P14-20  Additional resources: [The HAPPY book of play hand-out](file:///C:\Documents%20and%20Settings\ps06gl\My%20Documents\My%20Dropbox\BIB%20WORK%20FOR%20NAT\Final%20folder%20for%20submission\Happy%20Intervention%20Table%20and%20Handouts\The%20HAPPY%20book%20of%20play%20handout.pub); [BHF hand-out- Help your Baby Move and Play Everyday](https://dl-web.dropbox.com/get/BIB%20WORK%20FOR%20NAT/Final%20folder%20for%20submission/Happy%20Intervention%20Table%20and%20Handouts/Help%20your%20baby%20move%20and%20play%20every%20day%20handout%201%20BHF%20National%20Centre.pdf) |
| Recognises alternatives and develops an ability to plan ahead for when playing outside is unsafe (Environmental Context and Resources; Behavioural Regulation)  Recognises alternatives and develops an ability to plan ahead for when weather is too hot/cold/rainy/snowy to go out (Environmental Context and Resources; Behavioural Regulation)  Recognises alternatives and develops an ability to plan ahead for when there is no space/safe space inside the house to play actively (Environmental Context and Resources; Behavioural Regulation) | PO1, PO5, PO7  PO1, PO7  PO1, PO2, PO7 | Session 2 PN | What structured play activities could be done safely in the house without expensive toys etc. with the baby. This session focuses on very early years. Lollipop game | BCT 1; BCT 2; BCT 8; BCT 15; BCT 29 | P40-43 |
| **Change Objectives and Theoretical Domains Framework area(s)** | **Postnatal Physical Activity Performance Objectives** | **Session Number** | **Content** | **Behaviour Change Techniques** | **Manual Mapping** |
| Recognises alternatives and develops an ability to plan ahead for when they have no money for age-appropriate equipment (Environmental Context and Resources Behavioural Regulation)  Recognises alternatives and develops an ability to plan ahead for when they have no money for activities or to travel to activities (Environmental Context and Resources; Behavioural Regulation) | PO1, PO7  PO1, PO7 | Session 2 PN |  |  | P40-43 |
| Recognises alternatives and develops an ability to plan ahead for when playing outside is unsafe (Environmental Context and Resources; Behavioural Regulation)  Recognises alternatives and develops an ability to plan ahead for when weather is too hot/cold/rainy/snowy to go out (Environmental Context and Resources; Behavioural Regulation) | PO1, PO5, PO7  PO1, PO7 |  | What structured play activities could be done safely in the house without expensive toys etc. with the baby. This session focuses on very early years. Swotting game | BCT 1; BCT 2; BCT 8; BCT 15; BCT 29 | P40-43 |
| **Change Objectives and Theoretical Domains Framework area(s)** | **Postnatal Physical Activity Performance Objectives** | **Session Number** | **Content** | **Behaviour Change Techniques** | **Manual Mapping** |
| Recognises alternatives and develops an ability to plan ahead for when there is no space/safe space inside the house to play actively (Environmental Context and Resources; Behavioural Regulation)  Recognises alternatives and develops an ability to plan ahead for when they have no money for age-appropriate equipment (Environmental Context and Resources; Behavioural Regulation)  Recognises alternatives and develops an ability to plan ahead for when they have no money for activities or to travel to activities (Environmental Context and Resources; Behavioural Regulation) | PO1, PO2, PO7  PO1, PO7  PO1, PO7 | Session 2 PN |  |  | P40-43 |
| Improves motivation and develops an ability to plan ahead for when there is no time to engage child in structured play (Motivation and Goals; Behavioural Regulation)  Improves motivation and develops an ability to plan ahead for when child does not want to engage when mother is free to do so (Motivation and Goals; Behavioural Regulation) | PO1, PO5, PO7  PO1, PO5, PO7 |  | Recognising when baby wants to play; being flexible with play-time so that it occurs when the baby wants to play; making baby-active time a priority | BCT 15; BCT 26 | P40-43 |
| **Change Objectives and Theoretical Domains Framework area(s)** | **Postnatal Physical Activity Performance Objectives** | **Session Number** | **Content** | **Behaviour Change Techniques** | **Manual Mapping** |
| Develops skills to both cook nutrition meals and engage infant in activities (Skills) | PO1, PO5 | Session 2 PN | Encourage mum to perform structured and unstructured play – as discussed in ‘time to play’ this week | BCT 35 | P40-41 |
| Overcomes lack of family interest in physical activity (Social Influences) | PO1, PO5, PO7 | Session 3 PN | Importance of other family members engaging the baby in activity as this will alleviate time-related problems. As other family members become confident to play with baby, mum can do other things. Homework task to ensure each family member spends some time with infant | BCT 1; BCT 2; BCT 20; BCT 30 | P58-59 |
| Improves confidence that what they are doing/giving is right (Beliefs about Capabilities) | PO1, PO3, PO7 |  | Running theme outlined in the ‘Skills’ section. Mothers given positive feedback about their weekly physical activity homework tasks | BCT 13; BCT 6 | P58-59 |
| Develops an understanding and skills for playing with her baby in a stimulating way (Knowledge; Skills) | PO1, PO3, PO5 |  | Weekly age appropriate activities for baby and mum | BCT 5*; BCT 9; BCT 12; BCT 13; BCT 30;BCT 35* BCT 40 | P58-65  Additional resources: [Finding Time to Play Case Study hand-out](file:///C:\Documents%20and%20Settings\ps06gl\My%20Documents\My%20Dropbox\BIB%20WORK%20FOR%20NAT\Final%20folder%20for%20submission\Happy%20Intervention%20Table%20and%20Handouts\Finding%20time%20to%20play%20case%20study%20handout.pub); [Being Sedentary hand-out](file:///C:\Documents%20and%20Settings\ps06gl\My%20Documents\My%20Dropbox\BIB%20WORK%20FOR%20NAT\Final%20folder%20for%20submission\Happy%20Intervention%20Table%20and%20Handouts\Being%20sedentary%20questions%20and%20answers%20handout.doc) |
| **Change Objectives and Theoretical Domains Framework area(s)** | **Postnatal Physical Activity Performance Objectives** | **Session Number** | **Content** | **Behaviour Change Techniques** | **Manual Mapping** |
|  |  | Session 3 PN | Identify some home environment modifications to improve child physical activity and/or eating  Case study: The group are asked to find solutions for a mum who is juggling many priorities including food preparation | BCT 5; BCT 8; BCT 29; BCT 35 | P58-59  P58 |
| Recognises alternatives to using TV as a baby-sitter (Environmental context and Resources) | PO6, PO7 | Session 4 PN | Discussion of TV watching – TV is not a baby sitter. Share ideas of entertaining baby/keeping them safe when doing chores that do not involve being restricted or the TV | BCT 2; BCT 8 | P73-74 |

| **Change Objectives and Theoretical Domains Framework area(s)** | **Postnatal Physical Activity Performance Objectives** | **Session Number** | **Content** | **Behaviour Change Techniques** | **Manual Mapping** |
| --- | --- | --- | --- | --- | --- |
| Overcomes lack of family interest in physical activity (Social Influences) | PO1, PO5 | Session 4 PN | Importance of doing activity together as a family. Plan for family activity hour | BCT 10; BCT 5; BCT 8; BCT 34 | P71-73 |
| Improves confidence that what they are doing/giving is right (Beliefs about Capabilities) | PO1, PO3 |  | In reflective time at the beginning of the session mother provided with positive feedback when asked to discuss time to play homework | BCT 13; BCT 39 | P69 |
| Develops an understanding and skills for playing with her baby in a stimulating way (Knowledge; Skills) | PO1, PO3, PO5 |  | Weekly age appropriate activities for baby and mum are demonstrated and practiced | BCT 9; BCT 12; BCT 13; BCT 30; BCT 40 | P74-75 |
| Develops skills and an ability to plan ahead to prepare nutritious meals and engage infant in activities (Skills; Behavioural Regulation) | PO1, PO5 |  | In reflective time at the beginning of the session mother provided with positive feedback when asked to discuss time to play homework | BCT 5; BCT 8; BCT 35 | P69 |
| Recognises alternatives for when there is no space/safe space inside the house to play actively (Environmental Context and Resources; Behavioural Regulation) | PO2 |  | How to baby proof your house for safe physical activity | BCT 8* | P73-74  Additional resources: [Child Accident Protection Trust- Now That I Can Crawl hand-out](file:///C:\Documents%20and%20Settings\ps06gl\My%20Documents\My%20Dropbox\BIB%20WORK%20FOR%20NAT\Final%20folder%20for%20submission\Happy%20Intervention%20Table%20and%20Handouts\Now%20I%20can%20crawl%20handout%20Child%20Accident%20Prevention%20Trust.pdf) |

| **Change Objectives and Theoretical Domains Framework area(s)** | **Postnatal Physical Activity Performance Objectives** | **Session Number** | **Content** | **Behaviour Change Techniques** | **Manual Mapping** |
| --- | --- | --- | --- | --- | --- |
| Improves confidence to take child out of the house (Beliefs about Capabilities) | PO1, PO3 | Session 5 PN | Use visualisation to imagine a scenario taking infant out to play or to a group. Use self talk to build confidence, discussion of barriers and how to overcome them | BCT 32; BCT 22; BCT 28; BCT 36 | P94-96 |
| Improves motivation and develops an ability to plan ahead for when there is no time to engage child in structured play (Motivation and Goals; Behavioural Regulation)  Improves motivation and develops an ability to plan ahead for when child does not want to engage when mother is free to do so (Motivation and Goals; Behavioural Regulation)  Improve motivation and develops skills to prepare nutritious meals and engage infant in activities (Motivation and Goals and Skills) | PO1, PO5, PO7  PO1, PO5, PO7  PO1, PO5 |  | Plan the day to fit meal preparation in during infant nap, for example. So that when infant is awake, mother can spend some time in active play | BCT 8; BCT 26; BCT 35 | P89-93 |
| Improves motivation and develops an ability to plan ahead when too busy to go to activities outside the home (Motivation and Goals; Behavioural Regulation) | PO1, PO7 |  | Identify points in time where activity can be integrated into normal life. Practitioner facilitates discussion about small changes to family environment to increase physical activity | BCT 15; BCT 29 | P91-92 |
| **Change Objectives and Theoretical Domains Framework area(s)** | **Postnatal Physical Activity Performance Objectives** | **Session Number** | **Content** | **Behaviour Change Techniques** | **Manual Mapping** |
| Improves confidence that what they are doing/giving is right (Beliefs about Capabilities) | PO1, PO3 | Session 5 PN | In reflective time at the beginning of the session mother provided with positive feedback when asked to discuss time to play homework | BCT 13; BCT 36 | P84-85 |
| Develops an understanding and skills for playing with her baby in a stimulating way (Knowledge; Skills) | PO1, PO3, PO5 |  | Weekly age appropriate activities for baby and mum are demonstrated and practiced | BCT 9; BCT 12; BCT 13; BCT 40 | P96-97 |
| Develops skills and an ability to plan ahead to prepare nutritious meals and engage infant in activities (Skills; Behavioural Regulation) | PO1, PO5 |  | In reflective time at the beginning of the session mother provided with positive feedback when asked to discuss time to play homework | BCT 13; BCT 35 | 84 |
| Reduces feelings of embarrassment when playing with child in public (Emotion) | PO2, PO3, PO7 |  | Use visualisation to imagine a scenario taking infant out to play or to a group. Use self talk to build confidence discussion of barriers and how to overcome | BCT 5; BCT 24; BCT 22; BCT 28 | P94-96 |
| **Change Objectives and Theoretical Domains Framework area(s)** | **Postnatal Physical Activity Performance Objectives** | **Session Number** | **Content** | **Behaviour Change Techniques** | **Manual Mapping** |
| Recognises alternative to using TV as a baby-sitter (Environmental Context and Resources) | PO6, PO7 | Session 5 PN | Revisit discuss sedentary behaviour – TV is not a babysitter – discourage restriction >60 mins | BCT 2; BCT 8 | P97 |
| Recognises alternatives and develops an ability to plan ahead for when playing outside is unsafe (Environmental Context and Resources; Behavioural Regulation)  Recognises alternatives and develops an ability to plan ahead for when the weather is too hot/cold/rainy/snowy to go out (Environmental Context and Resources; Behavioural Regulation)  Recognises alternatives and develops an ability to plan ahead for when there is no space/safe space inside the house to play actively (Environmental Context and Resources; Behavioural Regulation)  Recognises alternatives and develops an ability to plan ahead for when they have no money for age-appropriate equipment (Environmental Context and Resources Behavioural Regulation) | PO1, PO5, PO7  PO1, PO7  PO1, PO2, PO7  PO1, PO7 | Session 6 PN | What structured play activities could be done safely in the house without expensive toys etc. with the baby | BCT 1*: BCT 8*; BCT 29 | P113-114  Additional resources: [BHF hand-out - Help your Child Move and Play Everyday](https://dl-web.dropbox.com/get/BIB%20WORK%20FOR%20NAT/Final%20folder%20for%20submission/Happy%20Intervention%20Table%20and%20Handouts/Help%20your%20child%20move%20and%20play%20every%20day%20handout%202%20BHF%20National%20Centre.pdf) |
| **Change Objectives and Theoretical Domains Framework area(s)** | **Postnatal Physical Activity Performance Objectives** | **Session Number** | **Content** | **Behaviour Change Techniques** | **Manual Mapping** |
| Recognises alternatives and develops an ability to plan ahead for when they have no money for activities or to travel to activities (Environmental Context and Resources; Behavioural Regulation) | PO1, PO7 | Session 6 PN |  |  |  |
| Recognises alternatives and develops an ability to plan ahead for when playing outside is unsafe (Environmental Context and Resources; Behavioural Regulation)  Recognises alternatives and develops an ability to plan ahead for when the weather is too hot/cold/rainy/snowy to go out (Environmental Context and Resources; Behavioural Regulation)  Recognises alternatives and develops an ability to plan ahead for when there is no space/safe space inside the house to play actively (Environmental Context and Resources; Behavioural Regulation)  Recognises alternatives and develops an ability to plan ahead for when they have no money for age-appropriate equipment (Environmental Context and Resources Behavioural Regulation) | PO1, PO5, PO7  PO1, PO7  PO1, PO2, PO7  PO1, PO7 |  | If want to take child out to play, take husband/partner/family member/member of PP group. Remind mothers of their lists of what local area has to offer and ask them to add to it now child has arrived. Practitioners need to have pre-made lists for areas from which mothers come. Discuss how to baby-proof your house in a physical activity -friendly way | BCT 29; BCT 36 | P112 |
| **Change Objectives and Theoretical Domains Framework area(s)** | **Postnatal Physical Activity Performance Objectives** | **Session Number** | **Content** | **Behaviour Change Techniques** | **Manual Mapping** |
| Recognises alternatives and develops an ability to plan ahead for when they have no money for activities or to travel to activities (Environmental Context and Resources; Behavioural Regulation) | PO1, PO7 | Session 6 PN | Weekly age appropriate activities for baby and mum are demonstrated and practiced | BCT 9; BCT 12; BCT 13; BCT 40 | P113-114 |
| Improves confidence that what they are doing/giving is right (Beliefs about Capabilities) | PO1, PO3, PO7 |  | In reflective time at the beginning of the session mother provided with positive feedback when discussing time to play homework | BCT 13; BCT 35 | P102-103 |
| Develops skills to prepare nutritious meals and engage infant in activities (Skills) | PO1, PO5 |  |  |  |  |
| Improves motivation and develops an ability to plan ahead for when too busy to go to activities outside the home (Motivation and Goals; Behavioural Regulation)  Reduces feelings of embarrassment when playing with child in public (Emotion) | PO1, PO7  PO2, PO3, PO7 |  | Planning for after the parenting programme, joining mum and baby activity groups – signposting by practitioners and goal setting for mums | BCT 4; BCT 10; BCT 36 | P112 |

**Table 6. Mapping HAPPY Intervention against (Parental and) Child Diet Performance Objectives**

**Desired Outcome:** **Infant develops healthy food preferences and dietary intake**

PERFORMANCE OBJECTIVES

1 = Mother/other guardian(s) is responsive to infant cues for hunger and fullness

2 = Mother/other guardian(s) adopts an authoritative parental feeding style (high control, high warmth)

3 = Mother/other guardian(s) does not use high energy foods as a reward

4 = Mother/other guardian(s) gives correct portion size for age of child

5 = Mother/other guardian(s) does not feed baby in front of television

6 = Mother/other guardian(s) encourages consumption of fruit and vegetables for child

7 = Mother/other guardian(s) discourages inappropriate consumption of high-calorie, energy-dense foods and drinks for child

8 = Mother/other guardian(s) cope with problems with ensuring infant has healthy dietary intake

NB: The interventions in this section also combat performance objectives 1 and 2 from ‘infant feeding’

NB: The interventions in this section also combat performance objectives 1 and 2 from ‘infant diet’

| **Change Objectives and Theoretical Domains Framework area(s)** | **Antenatal Diet Performance Objectives** | **Session Number** | **Content** | **Behaviour Change Techniques** | **Manual Mapping** |
| --- | --- | --- | --- | --- | --- |
| N/A | N/A | Session 1 AN | N/A | N/A |  |
| N/A | N/A | Session 2 AN | N/A | N/A |  |
| N/A | N/A | Session 3 AN | N/A | N/A |  |
| N/A | N/A | Session 4 AN | N/A | N/A |  |
| N/A | N/A | Session 5 AN | N/A | N/A |  |
| Improves motivation and develops an ability to plan ahead for when work makes it difficult to cook healthy meals for the child (Motivation and Goals; Behavioural Regulation)  Improves motivation and develops an ability to plan ahead for when there is variation in routine (Motivation and Goals; Behavioural Regulation) | PO1, PO6, PO7, PO8  PO1, PO7 PO8 | Session 6 AN | Planning ahead  Looking after mum | BCT 4; BCT 5; BCT 20 | P107 |
| **Change Objectives and Theoretical Domains Framework area(s)** | **Postnatal Diet Performance Objectives** | **Session Number** | **Content** | **Behaviour Change Techniques** | **Manual Mapping** |
| Develops an understanding about why/when to start weaning - including signs of readiness (Knowledge) | PO1 | Session 2 PN | Introducing solids: why and when; follow signs of readiness of weaning | BCT 1*; BCT 2*; BCT 8* | P33-35  Additional resources: [Start 4 Life hand-out - Introducing Solid Foods](https://dl-web.dropbox.com/get/BIB%20WORK%20FOR%20NAT/Final%20folder%20for%20submission/Happy%20Intervention%20Table%20and%20Handouts/Introducing%20solid%20foods%20handout%20Start%204%20Life.pdf) |
| Develops an understanding about what food and drinks to give (Knowledge) | PO7 | Session 3 PN | Provide information about what food and drinks to give | BCT 1 | P53-56 |
| Develops an understanding about what the right portion sizes are for their children (Knowledge)  Develops an understanding and skills for preparing fresh baby foods, as opposed to the easier option of ready meals (Knowledge; Skills) | PO2, PO5  PO7, PO8 |  | Weaning: how to do it. Also in PN Session 5 | BCT 1; BCT 2*; BCT 8*; BCT 12* | P53-56  Additional resources: [How to Start Weaning- A Practical Guide hand-out](file:///C:\Documents%20and%20Settings\ps06gl\My%20Documents\My%20Dropbox\BIB%20WORK%20FOR%20NAT\Final%20folder%20for%20submission\Happy%20Intervention%20Table%20and%20Handouts\How%20to%20start%20weaning%20a%20practical%20guide%20handout%20.docx); [Weaning: What and When hand-out](file:///C:\Documents%20and%20Settings\ps06gl\My%20Documents\My%20Dropbox\BIB%20WORK%20FOR%20NAT\Final%20folder%20for%20submission\Happy%20Intervention%20Table%20and%20Handouts\Weaning%20what%20and%20when%20table%20handout.doc); [The Dairy Council Menu Planner Stage 1- hand-out](file:///C:\Documents%20and%20Settings\ps06gl\My%20Documents\My%20Dropbox\BIB%20WORK%20FOR%20NAT\Final%20folder%20for%20submission\Happy%20Intervention%20Table%20and%20Handouts\Meal%20planner%20for%20weaning%20stage%201%20handout%20The%20Dairy%20Council.pdf); [Meal Planner for Weaning hand-out](file:///C:\Documents%20and%20Settings\ps06gl\My%20Documents\My%20Dropbox\BIB%20WORK%20FOR%20NAT\Final%20folder%20for%20submission\Happy%20Intervention%20Table%20and%20Handouts\Meal%20Planner%20for%20weaning%20handout%20.doc) |
| Develops skills to cook healthy (weaning) meals (Skills) | PO7, PO8 |  | Weaning: how to do it – texture and variety | BCT 1; BCT 9 | P53-56 |
| **Change Objectives and Theoretical Domains Framework area(s)** | **Postnatal Diet Performance Objectives** | **Session Number** | **Content** | **Behaviour Change Techniques** | **Manual Mapping** |
| Develops skills to get child to want to eat fruit and vegetables (Skills) | PO7 | Session 3 PN | Role of repeated exposure and Coping with food refusal |  |  |
| Develops an understanding and acknowledges the consequences of feeding their children an unhealthy diet (Knowledge; Beliefs about Consequences) | PO8 |  | Short- and long-term consequences of an unhealthy diet; short- and long-term benefits of a healthy diet in childhood | BCT 2 | P53-56 |
| Develops skills to give food and drinks- weaning (Skills) | PO3 |  | Weaning: how to do it – interpreting developmental stages, facial expressions and tongue thrusting | BCT 9 | P53-56 |
| Recognises alternatives for when they do not have the correct equipment for preparing and feeding nutritious –weaning meals (Environmental Context and Resources) | PO1, PO7, PO8 |  | Weaning: how to do it – no need for lots of expensive equipment | BCT 9 | P53-56 |
| Develops skills to get child to want to eat fruit and vegetables (Skills) | PO7 | Session 4 PN | Eating together; modelling behaviour and feeding role of family and parents  You are being Watched hand-out | BCT 9  BCT 1* BCT 2*; BCT 8* | P77-81  Additional resources: [You are being Watched hand-out](file:///C:\Documents%20and%20Settings\ps06gl\My%20Documents\My%20Dropbox\BIB%20WORK%20FOR%20NAT\Final%20folder%20for%20submission\Happy%20Intervention%20Table%20and%20Handouts\You%20are%20being%20watched%20handout.pub) |
| **Change Objectives and Theoretical Domains Framework area(s)** | **Postnatal Diet Performance Objectives** | **Session Number** | **Content** | **Behaviour Change Techniques** | **Manual Mapping** |
| Improves motivation and develops an ability to plan nutritious (weaning) meals for infant when time is lacking (Motivation and Goals; Behavioural Regulation)  Improves motivation and develops an ability to plan/prepare/cook healthier meals (Motivation and Goals; Behavioural Regulation)  Improves motivation and develops an ability to cook healthy meals for the child (Motivation and Goals; Behavioural Regulation) | PO1, PO6, PO7, PO8 | Session 4 PN | Eating together, modelling behaviour and feeding role of family and parents; time issues with food planning and preparation | BCT 9 | P77-81 |
| Develops an understanding of what the right portion sizes are for their children (Knowledge) | PO2, PO5 | Session 5 PN | Weaning: how’s it going? Introducing lumps | BCT 1; BCT 12*; BCT 19; | P87-88  Additional resources: [The Dairy Council Menu Planner Stage 2-hand-out](file:///C:\Documents%20and%20Settings\ps06gl\My%20Documents\My%20Dropbox\BIB%20WORK%20FOR%20NAT\Final%20folder%20for%20submission\Happy%20Intervention%20Table%20and%20Handouts\Menu%20planner%20for%20weaning%20stage%202%20handout%20The%20Dairy%20Council.pdf); [Weaning Recipes and Ideas hand-out](file:///C:\Documents%20and%20Settings\ps06gl\My%20Documents\My%20Dropbox\BIB%20WORK%20FOR%20NAT\Final%20folder%20for%20submission\Happy%20Intervention%20Table%20and%20Handouts\Weaning%20recipes%20and%20ideas%20handout.doc) |
| Improves motivation to make sure child is eating healthy (Motivation and Goals) | PO4, PO7 | Session 6 PN | Keeping motivated to feed family well | BCT 1; BCT 2; BCT 5*; BCT 8* | P109-111  Additional resources: [Keeping on the Right Track hand-out](file:///C:\Documents%20and%20Settings\ps06gl\My%20Documents\My%20Dropbox\BIB%20WORK%20FOR%20NAT\Final%20folder%20for%20submission\Happy%20Intervention%20Table%20and%20Handouts\Keeping%20on%20the%20right%20track%20handout.doc) |

**References**

Abraham, C., & Michie, S. (2008). A taxonomy of behavior change techniques used in interventions. *Health Psychology*, 27 (3),379-87.

Michie, S., Johnston, M., Francis, J., Hardeman, W., & Eccles, M. (2008). From Theory to Intervention: Mapping Theoretically Derived Behavioural Determinants to Behaviour Change Techniques. *Applied Psychology: an international review*, 57 (4), 660-680.

Gollwitzer, P. M. (1993). [Goal achievement: The role of intentions.](http://www.psych.nyu.edu/gollwitzer/93_Gollwitzer_Goal_Achievement_neu1.pdf) *European Review of Social Psychology,* 4, 141-185.

[Prestwich, A.](http://www.psyc.leeds.ac.uk/10/people/andrewp/index.htm), [Conner, M.T.](http://www.psyc.leeds.ac.uk/10/people/mc/index.htm), [Lawton, R.J.](http://www.psyc.leeds.ac.uk/10/people/rebeccal/index.htm), Ward, J., Ayres, K., & [McEachan, R.R.C.](http://www.psyc.leeds.ac.uk/10/people/rosiem/index.htm) (2012). Randomized controlled trial of collaborative implementation intentions targeting working adults' physical activity. *Health Psychology*, 31 (4), 486-495.
