# Supplementary material for: Using intervention mapping to develop a culturally appropriate intervention to prevent childhood obesity: the HAPPY (Healthy and Active Parenting Programme for Early Years) study
Source: Int J Behav Nutr Phys Act. 2013 Dec 28;10:142. doi: 10.1186/1479-5868-10-142 (PMC3895739; doi:10.1186/1479-5868-10-142)
Supplement: Additional file 3 — Cultural adaptation table. [file 1479-5868-10-142-S3.docx]

# Born in Bradford HAPPY study protocol – Response to considerations for adaptation

The Intervention Development group has reflected on the items suggested in Table 1. for the cultural adaptation of interventions and would like to present the following response for your consideration:-

Following discussions at the Steering Group meeting (22.10.11) it was agreed that for feasibility phase of the HAPPY trial due to current resources including time-constraints for developing translated resources and the capacity to run single ethnic intervention groups, the programme would be delivered in English. However non-English speaking parents and partners would be invited to participate and the demand and language preference will be assessed. The aim therefore is to incorporate a culturally adapted intervention in the intervention arm and S. Asian mothers will be randomised to either the culturally adapted intervention arm or the control group. Therefore there will be no element of self-selection by participants. As part of the process evaluation we aim to evaluate the acceptability of the culturally adapted intervention arm however it is unclear at this stage whether the culturally adapted component can be evaluated. We would therefore value advice on this aspect.

**Recruitment**

For the feasibility phase the recruitment will be undertaken by the research midwives and interpreters in the maternity unit at BRI. This is a method currently used to communicate with non-English speaking mothers attending the unit. We will produce the PIS and the Consent Form in Urdu for the feasibility stage. Follow- up for recruitment will be by telephone contact by multi-lingual speakers, thereby a full explanation can be offered in the appropriate language. The BiB research staff involved in recruitment are an experienced team who have been involved from the start of the project and diverse in nature (ethnicity, language, gender).

The initial plan for recruitment for the feasibility trial is via the maternity unit, however if this appears to be challenging, then using BiB’s excellent links, appropriate networks and local media will be approached to facilitate recruitment of mother’s from particular ethnic groups.

**Delivery**

Intervention delivery will be undertaken by experienced Family Links trained practitioners from a range of ethnic groups and who are familiar will the culturally diverse Bradford population.. They have experience of delivering parenting programmes to ethnically diverse, mixed and single-gender groups across a range of community-based settings.

A small scale survey about the concept, content and delivery aspects related to the HAPPY intervention undertaken with White and South Asian mothers attending the maternity unit indicated that 60% said they would attend the programme. There were no differences highlighted between the ethnic groups in terms of timing, length, duration and preference for delivery settings. Therefore it is planned to embrace the Family Links approach in terms of delivery however aspects such as group delivery, mixed ethnic groups and attendance by partners or a family member will be detailed in the participant information leaflets during recruitment. The above aspects will be evaluated as part of the acceptability of the intervention.

**Materials**

At this stage due to time-constraints and limited knowledge about levels of literacy, specific language requirements and the demand for translated materials in the target population, it has been decided that resources will not be translated into different languages. However if a requirement is highlighted, then currently available, non-branded resources from commercial companies will be used e.g. SMA. Meanwhile resources included will be those that have been tried and tested by practitioners working in Bradford and therefore appropriate for the cultural needs of the population. Involvement of a range of experienced practitioners including practitioners from diverse ethnic groups as part of Practitioner Working Group has strengthened this aspect. With respect to appropriate advice and resources related to diet and eating habits, the intervention group has sought the expertise of the dietetic dept. in Bradford who have much experience in this area.

**Evaluation**

A telephone survey is proposed as one of the evaluation methods. The group feel that this method has been tried and tested and its success is underpinned by the experienced, multi-lingual team of BiB community research team.

**Additional Considerations**

We agree that it would be useful to assess degree of cultural affiliation and therefore tease out perhaps levels of acceptable cultural adaptability to specific groups e.g. 1^st^, 2^nd^, 3^rd^ generation, which ethnic group etc… We would value advice about suitable tools to assess this aspect.

**Concluding remarks**

This project has thoroughly considered the possible relevant areas for adaptation using the tool featured in Table 1. The tool has allowed us to consider the 46 items identified to address cultural adaptations for the HAPPY intervention in a systematic manner at this stage of development. It has allowed us to identify areas that we feel able to address whilst providing justification why some adaptations are not possible because of available time and capacity. By undertaking this process we hope that we have demonstrated due diligence to the consideration of ethnicity in the development of the HAPPY intervention. Once the intervention has been finalised and the manual and resources developed we hope to provide more specific details of cultural adaptations applied in the development of HAPPY the intervention.

### Appendix 2. Typology of adaptations and suggestions/questions for the HAPPY protocol

|  | **Adaptation RECOMMENDATION** | **SUGGESTIONS and questions** | **BiB 1000 Response** |
| --- | --- | --- | --- |
| 1 | Exploratory phase with target population (same group as intervention group) | This has been covered by the group’s formative work. The intervention mapping approach has addressed these adaptation considerations. | This has been covered by the needs assessment. Suggestions have been weighed against logistical concerns and where possible, accounted for in the study design. Where it has not been possible to incorporate certain suggestions, these have been acknowledged. |
| 2 | Exploratory phase with target population (different group then intervention group or can’t tell) |  |  |
| 3 | Exploratory phase with community leaders |  |  |
| 4 | Ethnically-matched intervention staff or facilitator (with qualifications) | Are there ethnically matched staff within the Family Links team that could be employed to facilitate a culturally adapted group (this might mean having a specific group and not mixed groups)? | In the usual delivery of FL groups, the timing of delivery is not so critical so centres will plan groups across the whole year and if necessary will have a specific group for say, Urdu/Punjabi speaking or Bengali parents. Many trained facilitators are from the local populations so there is an ethnic matching.  For the intervention we propose that we seek pairings of practitioners with one white and one worker from SA community however groups will need to be mixed as there may be insufficient numbers to run ethnic specific groups. |
| 5 | Ethnically-matched peer role models or peer education | If it was not possible to have ethnically matched staff, it may be feasible to have ethnically matched peer role models or respected individuals take part in the sessions in collaboration with the project staff. | Ethnic matching may be difficult if there is a mixture of more than two ethnic groups in the group, as there are only two trainers per group.  See above  There are some barriers to the use of peer role models, local respected individuals or spiritual leaders in attending group sessions – without training they should not be leading sessions, and new people in and out of sessions disrupts group dynamics.  Long term it may be good to see if we can train some successful parents as peer role models to be co-facilitators.  The facilitators will also be referring to other sources of support within the local communities and these can be ethnically matched e.g.: breastfeeding peer supporters from local ethnic community, further support activities in local centres |
| 6 | Ethnically-matched facilitators and peer role models who have successfully changed their behaviour (both ethnically and behaviourally matched) |  | Ethnically-matched facilitators may wish to share own experiences if relevant. This aspect could be covered in training i.e. how and when this is appropriate because a sensitive approach is required as it could unintentionally alienate participants. Role models or peer supporters will be female as there will be SA mums in the group and male facilitators may be a barrier especially if discussing BF or pregnancy or “sensitive” topics  There may be an issue around dignity as SA mums will be less responsive to participating in exercise activities if there is a male role model as they may not be comfortable to bend over/change posture etc. However the use of appropriate peer-role models could prove to be useful. We could explore options through existing BiB networks. |
| 7 | Ethnically-matched high level/respected individuals to increase salience of program goals |  | We could explore this through BiB network however a sensitive approach is required as it might unintentionally alienate participants if cannot identify with them i.e. more than ethnically matching but social-class matching etc. |
| 8 | Utilises local/respected religious/spiritual leaders |  | Appropriate for single ethnic groups in future, however for our mixed groups this may not be helpful.  Re: SA recruits. We have involved the 2 female Muslim Chaplains at the hospital in early discussions and their input into considering the Islamic perspective.  However many spiritual leaders/respected representatives are predominantly male due to dynamics and norms of the SA culture. Through the existing BiB networks it is hoped to involve them in the consultation process, and obtain their support in raising awareness about this pilot intervention in order to encourage recruitment. |
| 9 | Ethnically- matched high level/respected individuals and community members throughout planning, directing, reviewing and implementing stages | Is there a steering group made up of community members? | Not directly involved in the steering groups – but the visit to the children’s centre aimed to gain feedback about the plan from members of the community. Plus, members of the community were consulted during the needs assessment phase of intervention development.  Additionally ethnically-matched mothers consulted in the HAPPY Survey undertaken in the maternity dept. at BRI on thoughts about the intervention.  Also see pt 8. |
| 10 | Ethnically-matched leadership within the study | The intervention team is diverse and members identifying with South Asian communities are involved | The steering group is comprised of a number of ethnically-matched academics from a range of disciplines including Epidemiology, Nutrition and Dietetics, Psychology and local NHS Public Health who are experienced in intervention development, implementation and evaluation. Additionally ethnically matched trainers of practitioners will be used. |
| 11 | Collaboration with ethnic specific institutions and professional organisations (formal) | Is it possible to form links with ethnic specific organisations or institutions that could be involved with/ support the intervention? Preferably this should be done prior to the start of the intervention. | We will be able to provide a list of all the links BIB have used in the development of the intervention.  We aim to contact the newly formed Bradford Muslim Women’s Council to canvass views on the intervention and help with recruitment.  E.g. Family Links collaborated with Family Action, Slough in production of the “Islamic Values and The Parenting Puzzle” booklet – this resource will be used in HAPPY Intervention. |
| 12 | Material depicts individuals from target population | The depicted individuals should also be presented according to appropriate cultural norms (e.g. some South Asian women may find an exposed pregnant stomach to be inappropriate) | We will ensure that all resources selected/used will be culturally appropriate and representative as far as possible of the target population. E.g. in the development of the HAPPY logo care was taken to ensure that it was applicable to target groups i.e. regarding clothing etc…  Also pictures showing health professionals or educators as female if they are shown giving advice particularly to pregnant or BF mums will be used  For the PA and diet resources that we will be developing from scratch we propose to include culturally appropriate images and a variety of ethnic models/cartoon images to representing the target population  Parenting Puzzle book is given to all parents – it contains cartoon images of parents/children which are representative of the target population  We could consider coding the list of resources as per 46 pts. for cultural adaptation as they are decided? |
| 13 | Material (video, booklet, skits, handouts, games) in target population’s language | Translation, bilingual worker and interpretation have been discussed and it was discussed that it would be too resource intensive at this stage. We would still advise this and if not for the pilot then definitely to be built into a full trial | There are members from a number of ethnic groups that will be invited to participate, each of which may have different languages. Therefore, given the additional resource requirements, it is likely that this will only happen should the full trial go ahead. Demand and language requirements will be evaluated during the feasibility phase. |
| 14 | Reflect target population’s language (usage – concepts, vocabulary) | Incorporate figurative language/sayings, commonly used by the target population where possible and when accurate or contemporary | Much of the intervention is based on group discussions and the words of the parents/carers themselves. Facilitators are aware of the importance of respectfully listening to the views and words of parents/carers themselves and repeating, recording them.  Eg: Session 1 of the ante-natal programme parents are invited to think about the Family Rules for creating a calm and happy family life – this will be different for each parent/carer and will reflect their own words.  Throughout the intervention participants are encouraged to share ideas/opinions regarding PA and dietary practices (e.g. Session 3 AN), facilitators will be instructed, during training, to listen to the language participant use and use these terms/sayings when communicating with participants and in the group discussions  Additionally from our formative work and involvement of ethnically matched practitioners and researchers we are aware that certain words have different meanings. E.g. healthy means chubby, bonny within S. Asian culture and therefore will be addressed in the training of FL practitioners. |
| 15 | Match reading level and literacy | It is important to consider/ assess the population’s reading level and literacy and match text with graphics, or provide assistance with questions | We can test the resources to ensure they are at the appropriate literacy level using the Flesch method and with a range of parents attending Children Centres with whom we have links with.  Within groups, facilitators are familiar with communicating messages to parents/carers with limited literacy and will always read through any flipcharts or information boards to assist with understanding.  To optimise effective communication if literacy is an issue, methods such as demonstration/using diagrams/pictures/contrast of colour/short and simple statements/text font size/symbols etc will be used.  Parenting Puzzle book does contain a variety of images and use of different methods to communicate the key messages ……  Furthermore resources selected/developed will be clear, concise, simple messages and avoid jargon etc… e.g. SMA website includes a range of A4 sized hand-outs in 8 different languages for key intervention messages.  A Parenting Puzzle booklet with key messages also translated into Urdu is available which parents/carers can use if they read Urdu  PA resources that are specifically developed for the intervention will use step by step pictorial instructions along with simple captions to deliver information – e.g. how to perform pelvic floor exercises (session 6 AN and session 1 PN) |
| 16 | Reflect target population’s preferred method of communication | Storytelling, poetry and literature; hands-on/interactive learning; testimonials; face-to-face may be appropriate | We can evidence a number of delivery techniques that will be used to meet the needs of all those attending the programme  Delivery techniques will include:  Group discussions, small groups or paired discussions, role play and modelling of behaviours, feedback sessions to share practice and successes. |
| 17 | Material presents ethnic specific data | If including statistics or background data in the material it is important that it is specific to the target population | If we need to present any information/data then we will ensure that it is ethnically specific. Local stats specific to BME groups in Bradford on diet/PA/BF etc. can be pulled from public health reports and other local sources |
| 18 | Material depicts appropriate graphics and scenarios (this can be heterogeneous) | Foods, clothing, logos, artwork within the materials should be targeted where possible. | Logo has been designed without any stereotypes associated.  Any information will include ethnically appropriate foods and clothing to help participants personally identify with key messages  See Section 15 above |
| 19 | Material/guidance based on preferences of target population | Foods, recipes, flavourings (plate demonstrations and portion sizes; sleep patterns) | Culturally diverse lifestyle patterns (eating, sleeping, physical activity etc) are acknowledged by practitioners working in Bradford and therefore all advice offered will be appropriate to the needs of the target groups. This will be addressed in the training of the FL practitioners who already have a good understanding of these aspects.  The dietetic dept has expertise in the area of cultural aspects of food and dietary patterns across cultural groups therefore appropriate resources will be developed.  Some specifics to include during diet sessions will focus on::  Eat well plate featuring culturally diverse foods  Traditional recipes using healthier methods of cooking; Traditional recipes modifications i.e. less fat, sugar or salt; menu planning/regularity in meal pattern/food choices etc. Promoting healthier eating using traditional recipes/spices/flavours without compromising taste  With regards to PA, parents will come up with their own PA plan with their own PA preferences rather than being given a structured exercise routine to follow. Parents will be encouraged to think and discuss their preferences and what will work for them in their daily routines. Different PA examples which reflect cultural diversity will be given to stimulate thinking: E.g. yoga, Bollywood dance home exercise video, zumba, swimming, walking, small lifestyle changes –(walk to shops rather than drive). Facilitators will listen to PA ideas generated in group discussions, record any new ideas and think will then inform material development for full trial |
| 20 | Material developed specifically for target population (by project investigators, expert opinion, tools) | It may not be possible in the pilot to create separate materials for the target population, but this should certainly be done for a full trial | Some of the materials may incorporate this – especially those developed from scratch, or amended based on other programme materials. |
| 21 | Materials created by members of the target population | It might be an opportunity in the pilot intervention for participants to create/ contribute relevant materials for a future trial e.g. recipe books, exercise ideas, knowledge of community networks, strategies to encourage family support etc. | This will be integral to lots of the group discussions and will ensure that information is relevant to the local community.  e.g. Ask group members to share tips on what works well with recipe modification etc. with each other during discussions or have a flip chart up so that they can jot down tips. i.e. using less fat by measuring it can still result produce an authentic taste…sharing information will also promote inclusion and cohesion  This could be collated into key information to be shared with subsequent groups, but it is important that each group is encouraged to share their own knowledge and information. This could feed into resources for the full trial  It does link to some “Time to Have a Go” activities eg:  Session 2 PN – Physical Activity Mingle to identify activities in the local area  Session 4 PN – Cook a Healthy Meal and report Outcomes |
| 22 | Utilise resources from target population |  | See above |
| 23 | Intervention content targets population’s social and cultural values | Could include spiritual themes, prayers, inclusion of kinship, collectivism; cultural traditions; family commitments; connection to history – the idea of the ‘Islamic values’ programme was very good in terms of incorporating faith into the intervention and anecdotally it appears that this helped women in justifying changes they wanted to make within the household. This should be considered for a full trial | Lots of examples within the parenting programme  Eg: Session 2 AN – discussion on A Celebration of Birth draws out family traditions, cultural issues, practices relating to birth, physical activity and dietary practices.  Session 4 AN – discussion and activity on Nurturing Ourselves draws out the place that spiritual themes and prayer can have for parents/carers in meeting their needs  Islamic Values and the Parenting Puzzle book makes connections between faith and all of the key principles of the parenting programme, such as empathy, positive discipline and self-esteem. This will be referenced by practitioners as they introduce concepts. |
| 24 | Provide ethnically/culturally appropriate food/activities/music | Offer culturally appropriate foods and dishes; portion and plate sizes; recommended exercises | See above |
| 25 | Maintaining cultural significance of food | Encourage different preparation rather than avoidance of food | There will be awareness of the strong cultural influences on diet e.g honey and new-borns. High fat, high sugar food to mothers following pregnancy. The concept of “hot and cold foods” which may challenge some HE principles for pregnancy/breastfeeding.  All advice regarding diet and food will be culturally relevant and therefore alternative foods and cooking methods will be encouraged e.g. instead of deep frying popular food items e.g. kebabs – baking or grilling will be encouraged  Fried rice vs plain boiled rice  Parathas/puris vs plain chapatti/tandoori roti/naan made without fat  Addition of veg to a curry or having a side salad as part of a meal  Using healthier sources of fat for sweet puddings such as veg marg instead of butter/semi skimmed milk in rice pudd instead of full cream  All above would be weaved into training programme  The idea of food swaps is also used in order to promote appropriate dietary changes. |
| 26 | Presents a pro-ethnic/race approach | Materials present cultural pride and have a positive approach, advocate changes that are culturally based | We hope to evidence this through the resources developed/selected for use**.** Also eliminating misconceptions that traditional ethnic diets are unhealthy and the western diet is superior through discussion and activity etc. |
| 27 | Intervention goals are culturally appropriate | Focus on reducing fat and not on losing weight when weight loss is not a priority for the population; emphasis on personal health improvement as means of assisting the family or community  There may be views that a heavier baby is healthier and, as above, health goals may be more appropriate than advocating weight loss | The evidence collected regarding barriers was based on the ethnic groups being targeted in Bradford (both directly and through the literature). Performance objectives were developed based on ethnicity-appropriate literature and data from the BIB cohort study (we will need to specify details about this). The intervention content has been carefully designed to ensure intervention goals are appropriate –  e.g. Key messages regarding diet and physical activity are evidence-based and under-pinned by the formative research. E.g. dietary practice s differ within groups e.g. S.Asian mothers more likely to breast-feed, introduce solids later than White British. Perceptions around obesity have also been explored therefore we are confident that our intervention goals are culturally appropriate. |
| 28 | Intervention delivered in culturally appropriate or preferred format | Joint sessions are not always acceptable and this needs to be assessed with the target population –may be particular issues with partners (male) attending sessions | Joint sessions were not particularly highlighted in the HAPPY intervention survey however we acknowledge that it may be an issue for some mothers. The intervention will include mixed ethnic groups and partners/family member in the feasibility stage however this will be explained at recruitment and views of those consenting or not will be collated to inform the full trial. We will however aim to maximise participation by encouraging the attendance of a chaperone a friend or member of the family. |
| 29 | Addresses emotional barriers to participation | Consider stress (of inner city life), safety in the community, family concerns | The BiB team are experienced in recruitment and retention of participants and we are confident that this will be well addressed. However we are aware that this is a 12 wk intervention which requires significant commitment from participants therefore staff undertaking recruitment will need to promote the benefits whilst dealing with the barriers. E.g. as the venue maybe outside the local area, transport will be provided and crèche facilities will be offered in order to address barriers to participation. Consideration may need to be given to the specific examples in the opposite column and those recruiting will need some specific briefing on how to best address these issues should they arise.  Also see pt 28 above |
| 30 | Address discrimination and mistrust | Address possible racism and stigma experienced by women | Facilitators have been trained and will be experienced in dealing with issues of discrimination and mistrust.  The approach taken to all parents/carers is one of respect, empathy and valuing the importance of their experiences.  Some specific activities will open up the discussion and provide parents/carers with strategies to better deal with discrimination  Eg: Session 2 AN – Discussion about Being Pregnant in My Family can raise issues of discrimination/body image, perception of self.  Session 4 AN – Introduction of Personal Power and the power of positive words as a strategy of coping with pressures and reducing the stress and impact of negative experiences. |
| 31 | Addresses physical/financial (structural) barriers to participation | Consider access to transport, time restrictions, safe locations, financial constraints addressed (providing compensations/suggesting alternatives); childcare | See 29 |
| 32 | Consider target population’s employment situations | Do their employment or family situations make it difficult for them to attend sessions and is there anything that can be done to facilitate attendance? | See 29. The intervention is planned to be delivered across a range of settings across Bradford and additionally a range of delivery times is currently being discussed in order to offer a flexible approach and enhance participation |
| 33 | Intervention addresses health behaviour patterns found in target population | From the formative work there should be a good idea of the target populations health behaviour patterns in terms of nutrition and physical activity and this is already informing the intervention as a result of the mapping process, it is therefore likely that the intervention will be relevant to the target population | Desired outcomes and performance objectives were developed based on ethnicity-appropriate literature and data from the BIB cohort study |
| 34 | Issues unique to their context | May need to consider addressing food at social gatherings, difficulty of exercising with lack of women-only facilities or with safety concerns | These are addressed in the intervention due to the barriers identified based on culturally relevant literature and discussions with members of the community/community practitioners  With regards to PA, parents will come up with their own PA plan with their own PA preferences rather than being given a structured exercise routine to follow. Parents will be encouraged to think and discuss their preferences and what will work for them in their daily routines. Different PA examples which reflect cultural diversity will be given to stimulate thinking: E.g. yoga, Bollywood dance home exercise video, zumba, swimming, walking, small lifestyle changes –(walk to shops rather than drive). Facilitators will listen to PA ideas generated in group discussions, record any new ideas and these will then inform material development for full trial |
| 35 | Utilises appropriate incentives | Held at times and venues that are suitable for women; use of small gifts given at intervals during the study found to enhance retention of participants in some studies (measuring spoons, exercise video, baby sling for exercise etc.); giving back in to the community in terms of training peer educators/supporters or creating community resources e.g. setting up women only exercise classes (baby sling exercise classes) | We have plans for this specified in the intervention content and will be able to provide specific details following the development of the intervention.  The Parenting Programme uses the idea of regular small incentives for parents such as relaxation gifts, candles, fridge magnets, bookmarks, relaxation exercises etc.  In addition to encourage attendance to the Post-natal component following the birth of the baby we are offering a group/individual photograph in session 1.  Another idea which needs further discussion is the setting up of a resources library – culturally diverse recipe books and exercise videos (preg yoga, Bollywood dance, aerobics for PN etc.?? |
| 36 | Utilises and addresses appropriate norms | BMI cut-offs, normative frameworks, body image | Family links practitioners should have broad knowledge of community norms and the intervention materials will also conform to norms. The recruitment criteria is obese women however a BMI of 25 has been adopted to take into account ethnic differences. |
| 37 | Address concerns with medical programmes, procedures and medication | Could explore the women’s previous experience with studies or medical programmes and address any issues or concerns that they may have had that could affect their retention and participation in the present study; this should include interactions with NHS or other health services, including doctors and nurses |  |
| 38 | Utilises appropriate evaluation instruments | Regionally and culturally specific foods added to Food Frequency Questionnaire (FFQ), appropriate BMI cut offs | Although we are using validated tools to evaluate outcomes, there are limited validated tools for specific ethnic groups. We have taken steps e.g. the Infant FFQ used at 12 and 18 months; parent FFQ at 6 months to ensure that they reflect culturally appropriate foods.  Likewise a BMI of 25 has been adopted to take into account the ethnic differences in defining obesity. |
| 39 | Located in ethnically/culturally appropriate/familiar location | It may be important to assess how comfortable the women are with the location and whether a community location may be more appropriate | Have we survey/anecdotal data from children’s centre to indicate attendees are happy with the locations?  The HAPPY intervention Survey with mothers attending the maternity unit at BRI indicated a preference for community and GP locations |
| 40 | Utilises ethnically/culturally appropriate media sources | Could consider appropriate media sources for recruitment and advertising, also in terms of referring people to materials e.g. it was discussed that for internet/computer savvy women, could refer them to information websites and there may be sites or e-networks that are culturally appropriate | Locally in Bradford we have access to the following resources – useof which requires discussion :  Sunrise Radio ( has an established based and has health focused programs)  Asian newspapers e.g. Asian Eye Watan, Daily Jang etc  Professional Muslim Network (run from the Council) |
| 41 | Utilises ethnically/culturally appropriate informal networks | Community groups, lay health educator networks | We need to think about culturally specific signposting?  Some suggestions could include:  Some suggestions:  Specific community development workers  Bradford Food Network (circulation list)  Manningham Advice Centre  Carlisle business centre (CBC)  Inner city based libraries  Community centres  Charities (COEMO based at CBC hold a district wide data base)  Bradford Interfaith Centre (broader reach) |
| 42 | Teach appropriate communication skills | Could think about the women learning to communicate their needs, in terms of changing behaviours within the family and being supported, to family and elders while maintaining harmony and respect (Islamic values materials useful here) | Some of the barriers identified relate to this area and therefore will be directly tackled as part of the programme  Many elements of the parenting programme focus on developing skills for parents/carers to make the changes that they want to make  Eg: Session 4 AN – Introduction of Personal Power and the power of positive words as a strategy of coping with pressures and reducing the stress and impact of negative experiences.  Session 6 PN – Problem Solving skills to develop strategies that encourage families to work together to change behaviours without anger  Islamic values will help with understanding and a cultural context for making changes.  Time to Have a Go activities encourage regular practice of activities and feedback at the following session to clarify the activities or strategies practised. |
| 43 | Encourages/involves social support | Invite families and friends to sessions – although the acceptability of this needs to be assessed, particularly in terms of male attendees  Could also go into the home for a visit to reinforce messages to families – it has been found that having a male health visitor/peer educator visit the home was effective in influencing change with spouses and family members | Again, this is embedded in the programme to tackle identified barriers. Partners and family members are invited to accompany the mother. However this will be evaluated. However introducing family and friends into the sessions can disrupt the group dynamic and the safe bond developing between group members unless they have been involved from the beginning.  The whole programme encourages implementing and practising the activities within the home as a whole family approach.  Although home visits are not an option due to resource and capacity issue, the intervention will use telephone contact and texting as appropriate to maintain contact with mothers and provide on-going support between sessions. |
| 44 | Purposefully maintaining an exclusive or open intervention environment | This can only be assessed with the groups if they are happy with a mixed group format or not and the decision to have mixed sessions also needs to be made with consideration of the resources available | It has been agreed that a mixed-group format will be offered during the feasibility stage as we may not be able to recruit sufficient participants for single ethnic group sessions. |
| 45 | Cross-cultural training for all study personnel | Cultural competency training for all staff | Cross cultural training including factors related to diet and physical activity will be addressed in the training programme,  Additionally The Family Links training addresses cultural competency by encouraging respectful acknowledgement of the experiences that all parents/carers bring to a group, of the importance of listening and of the importance of families identifying their own solutions to the changes they want to make. |
| 46 | Gender considerations | Need to assess the acceptability of mixed gender sessions and consider perhaps women-only sessions | This all depends on whether each woman follows the faith or cultural principles. Faith led women will require a male chaperone and culturally led women will accept a female or male chaperone.  Although mixed sessions will be run due to reason identified in pt. 44.above, the acceptability of this will be assessed as part of the process evaluation |
