# Supplementary material for: Using intervention mapping to develop a culturally appropriate intervention to prevent childhood obesity: the HAPPY (Healthy and Active Parenting Programme for Early Years) study
Source: Int J Behav Nutr Phys Act. 2013 Dec 28;10:142. doi: 10.1186/1479-5868-10-142 (PMC3895739; doi:10.1186/1479-5868-10-142)
Supplement: Additional file 4 — a. MASTER HAPPY Antenatal Manual FINAL VERSION. b. MASTER HAPPY Postnatal Manual FINAL VERSION. c. HAPPY manual resources. [file 1479-5868-10-142-S4.zip › 1523029418990916_add4a.doc]

**WELCOME TO THE HAPPY MANUAL!**

This introductory section aims to provide you as practitioners with some background information about the development of the HAPPY intervention.

BACKGROUND

Childhood obesity is associated with a range of adverse consequences including metabolic abnormalities, increased risk of Type II diabetes, musculoskeletal and psychological problems, and adult obesity. Recent obesity trends in children aged 2-11 years indicate that although the rate of increase of overweight and obesity is declining, obesity among children is still rising. There is also evidence that antenatal factors (e.g., GDM, maternal weight gain) and early postnatal factors (e.g., feeding practices) play a key role in the development of childhood obesity, and that growth trajectories of children indicate that the risk of childhood obesity can start before the baby has been born, and is present in the first few months of life. To summarise, evidence indicates that the following factors have been directly associated with childhood obesity:

- Mother is obese (genetic or environment)
- Rapid weight gain in the infant
- Short sleep duration
- Less than 30 minutes daily physical activity,
- Consumption of sugar-sweetened drinks,
- Screen-viewing,
- Parental feeding practices
- Parenting styles

It has been recommended that programmes to prevent the risk of childhood obesity should:

- Be multi-faceted (diet, p/a, sedentary behaviour, behavioural change techniques)
- Include parental involvement, family-based (wider family)
- Target skills and competencies
- Address parenting practices and parenting skills
- Be delivered by trained practitioners
- Be underpinned by behavioural theory

Therefore, the BIB childhood obesity prevention intervention has incorporated these components

OVERVIEW OF THE BIB CHILDHOOD OBESITY PREVENTION INTERVENTION: THE HAPPY PROGRAMME

The HAPPY programme has been developed based on an intervention mapping framework, which consists of six steps

1. Needs assessment
   - Research evidence
     - Published literature
     - Information from Bradford
   - In depth discussions with:
     - Practitioners
     - Parents and wider family
     - Parenting experts
2. Matrices
   - We used these to identify the desired behaviours, and the reasons why people might find it difficult to do these behaviours (barriers)
3. Theory-based intervention methods and practical applications
   - Identify and use theories that can help to create effective interventions
4. Intervention program
   - Develop the intervention alongside practitioners and users
5. Adoption and implementation
   - Pilot the intervention
   - Train delivery staff
   - Deliver the intervention
6. Evaluation plan
   - Identify ways to assess whether or not the intervention worked
     - E.g., if the intervention aims to increase physical activity, we need to measure physical activity levels before and after the intervention to see if the intervention helped to achieve better levels

THE ‘FAMILY LINKS’ PARENTING PROGRAMME

Family Links Nurturing Programme is a parenting programme to promote emotional health and well-being, relationship skills and positive behaviour management strategies for parents/carers. It is based on four key building blocks for successful relationships and confident parenting –

1. Self-esteem and self-awareness
2. Appropriate expectations
3. Positive discipline
4. Empathy

The Nurturing Programme has been successfully run across Bradford for the last 5 years, mainly in Children’s Centres

Family Links have recently developed an antenatal programme based on similar key principles which we are using for this intervention. Therefore, we decided to join forces and build on the existing structure and format to produce the HAPPY programme.

Here are some details about the HAPPY Programme:

- It is aimed at women who are pregnant with a BMI 25+
- Fathers, other significant carers will be invited to attend but expected to attend all sessions
- The intervention will last for 12 sessions – 6 during the antenatal phase and 6 for the post-natal phase until baby is about 9 months old
- It will be delivered by trained and experienced Family Links practitioners from Bradford
- 6 groups will run for 10-12 parents/carers each
- Sessions will run in community venues around Bradford
- Sessions will last around 2 ½ hours
- We plan to run at least one group evening and one group Saturday to increase access

We will focus on improving behaviours in the following areas:

- Parenting practices, parenting skills and parenting styles
- Maternal diet - pregnancy and post pregnancy
- Infant diet
- Maternal physical activity
- Infant physical activity and sedentary behaviour
- Knowledge, skills and competencies
- Culturally appropriate

What do we want to achieve?

- Infants with a healthy weight
- Confident and informed parents
- Healthy family diet
- Mothers feeling confident about how, what and how much to feed their infants – and doing it!
- Infants receiving a healthy diet
- Mothers being active and providing opportunities for physical activity for their children

**BACKGROUND INFORMATION**

BRAIN SCIENCE

Humans, of all animals, are the most dependant/helpless for a long period. It is preparing the body for independence.

The antenatal period and 0-3 years lay the foundations for lifelong learning, behaviour and health. Early experiences build into our bodies and brains for optimal function.

At birth, the brain weighs 400grammes. By one year old, it weighs 1000grammes – massive brain growth and wiring takes place.

Your baby’s brain development starts in pregnancy, but is not completed when your baby is born. Special fatty acids (LCPs) in breast milk help the baby’s brain and nervous system to develop and mature

Nature and Nurture coexist – most prescribed hard genetic factors can be modified by experience, e.g. risk factors for adult heart disease are embedded in adverse childhood experiences (Doug, 2004).

**Importance of early years’ experience**

Normal brain plasticity is influenced by experience and is at its most receptive in early years up to 10 years. The ability for the brain to change continues throughout life but the older we get the greater physiological “effort” is required to achieve change as plasticity reduces.

For example at birth the brain has the capacity to speak any language. Until 9/12 – 12/12 the ability to differentiate sounds is at its greatest, so an average intelligent young child can learn other languages more effectively than a highly intelligent 30 year old who will never get the intonation completely right.

**Probabilities and predetermined facts**

Early experiences aren’t final but are probabilities in how they will influence development.

**Genetic pre-determination:**

The wiring and connections for developmental stages are genetically programmed, e.g. crawling at 6-12 months; walking at 1 year. Antenatally there are only 4 crucial weeks of brain development when rubella can cause serious damage, whereas at any other time in life it is harmless. Similarly, the impact of alcohol on a foetal brain may lead to foetal alcohol syndrome.

**Through complex physiological pathways early life experiences are built into our bodies for better or worse**

Brains build over time and are, in fact, sculpted. The active ingredients are the baby’s relationships, language rich environments and mutually responsive interactions with the adult, based on contingent reciprocity (“serve and return”).

The baby’s brain is genetically programmed for responsiveness from adult carers. This promotes healthy brain architecture and adaptive regulatory systems. In biological terms, as the brain circuits get more and more complex, if the serve and return interaction between baby and carer has been inadequate, it takes more oxygen to repair the damage. The brain is at its most responsive up to 4 years and then the neuroplasticity decreases. Therefore the cost of remedial work in older children is much higher than prevention or early intervention.

So if the brain does not get the responses it is biologically ready for, e.g. no or little attention; inadequate food etc, the stress system becomes activated. Biological “memories” link neglect and maltreatment in childhood to greater risk of adult health disease; diabetes, depression etc.

**Early life experiences are built into our bodies (for better or for worse).**

Stable and supportive relationships, language-rich environments, and mutually responsive, “serve and return” interactions with adults promote health brain architecture and adaptive regulatory systems.

**Stress**

Excessive or prolonged activation of stress response systems are reduced availability of the buffering protection of supportive relationships can weaken brain architecture and disrupt the development of other organ systems.

**Three levels of stress response (**Shonkoff, 2011)

**Positive**

Brief increases in heart rate, mild elevations in stress hormone levels

**Tolerable**

Serious, temporary stress responses, buffered by supportive relationships

**Toxic**

Prolonged activation of stress response systems in the absence of protective relationships

**Cortisol**

Persistently elevated Cortisol creates chemical changes in the body that damage health. This stress hormone activates alertness for danger so the blood pressure and heart rate increases. If Cortisol levels are too high for prolonged periods the brain circuits in the Epicanthus and Pre Frontal cortex get damaged/destroyed. This is the part of the brain that develops social skills and influences memory development. Environmental stress e.g. angry or violent environment or maternal depression can also activate high blood sugar levels because of the sense of danger. In addition the inflammatory system is activated in preparation for tissue healing. These can lead to childhood diabetes because persistently raised blood sugar increases insulin resistance.

So a child whose stress response is constantly activated sees the world as a threatening place and over reads “anger” in facial expressions, tending to read most facial expressions as angry and therefore responds aggressively.

Therefore toxic stress in childhood creates lifelong problems physically, socially and emotionally.

**WELCOME TO THE WORLD**

**Week One**

**BRAIN SCIENCE AND BONDING**

**STEP BY STEP**

PLAN FOR TODAY

|  | **ACTIVITY** | **TIME** | **MATERIALS NEEDED** |
| --- | --- | --- | --- |
|  |  |  |  |
| 1 | Welcome | 10 mins | Refreshments, music & player, ‘Welcome’ sign, attractive table cloth, flowers, badges, stickers, felt-tip pens, plan for today, attendance sheet, reward system, etc. |
| 2 | Icebreaker: Introductions | 10 mins | Flipchart, pens |
|  |  |  |  |
| 3 | Introducing the Nurturing Programme | 5 mins | The Parenting Puzzle book  Post-it notes |
| 4 | Group Rules | 10 mins | Flipchart, pens  Hat or box  Fun group dividers |
|  |  |  |  |
|  | Exercise on Family Rules | 10 mins | Flipchart, pens |
|  |  |  |  |
| 5 | Nurturing My Baby’s Healthy Development and Growing Brain | 30 mins | Flipchart, pens, Post-it notes  Hand-outs |
|  |  |  |  |
|  | *TEA & COFFEE BREAK* | *10 mins* | *Refreshments* |
| 6 | Baby’s Brain Development | 45 mins | Board – Empathy Steps  Board – 4 Constructs |
| 7 | Name Game | 5 mins | Soft ball or beanbag |
|  |  |  |  |
| 8 | Time To Have A Go | 5 mins | Time To Have A Go Handout  Book – Why Love Matters, Sue Gerhardt |
|  |  |  |  |
| 9 | What did you think of today? (Feedback) | 5 mins | Feedback forms, pens, basket/ container, mints, relaxing music |
|  |  |  |  |
| 10 | Goodbye Circle: Smile | 5 mins |  |
|  |  | 150 mins |  |

**WELCOME TO THE WORLD**

**HAPPY Antenatal Programme**

**Week One**

**BRAIN SCIENCE AND BONDING**

**STEP BY STEP**

| **1** | **WELCOME** | **10 mins** |
| --- | --- | --- |
|  | **Display** board with ‘Welcome to Family Links Nurturing Programme for Parents’. |  |
|  |  |  |
|  | **Greet** group members warmly as they arrive, have background music playing, and offer refreshments. **Ask** parents to make name badges, and to sign the attendance sheet. **Ask** them to complete a registration form if they are happy to do so. |  |
|  |  |  |
|  | **Settle** the group, and then introduce yourself, mentioning the background to the Nurturing Programme and your work with it to foster parents’ confidence that you are qualified to lead the group. **Display** and briefly **go through** the Plan for Today of topics to be covered in the session. |  |
|  |  |  |
|  | **Go through** the ‘housekeeping’ items:  1. Tell everyone where the toilets and fire exits are.  2. Explain the each week there will be drinks on arrival and again in the break half way through the session; these are provided free of charge – as is coming to the group  3. Remind the group of the starting and finishing times, and stress the need for a punctual start each week.  4. Give a copy of the *The Parenting Puzzle* book to each member of the group .  5. Invite anyone to tell you if they have any special needs (in the group or one-to-one if they prefer) so you can give them the help they need, e.g. difficulties with seeing, hearing, reading, writing, etc, and ask if anyone has allergies or needs specific prayer time.  6. Give everyone a contact phone number for group facilitators so that group members can let them know in advance if they will have to miss a session. **Ask** if facilitators can ‘text’ between sessions if necessary.  7. If you do not already have one, make a list of everyone’s names, addresses and phone numbers (compiled from registration form).   8 Ask people to complete the Children’s Centre Enrolment Form and sign the consent section. |  |
| **2** | **ICEBREAKER: Introductions** | **10 mins** |
|  | Divide people into pairs in a fun way (e.g. different fruits). Ask the group, in pairs, to chat briefly about the following statements displayed on a board:  ‘My name is………… My family consists of………. My baby is due on……….’  ‘One thing I hope to gain by coming to the programme is……………’  After 5 minutes, invite each person to feed back on his/herself or partner – whichever each pair prefers. |  |
|  |  |  |
|  |  |  |
| **3** | **INTRODUCING THE NURTURING PROGRAMME** | **5 mins** |
|  | Begin by emphasising the value of parents joining the group, because it allows us to take stock of our needs as individuals and as parents. Suggest that parenting is the most demanding, important, skilled job there is – with no salary, no pension, and redundancy if we do it really well! The vital role of parents is not generally appreciated; in the group, it will be.  We hope the first 6 sessions during the antenatal period will provide a good opportunity to explore our roots, traditions, hopes and fears and to share experiences and ideas. This will help develop even stronger bonds and communication between you, your partner and your baby before it is born. This will develop firm, secure foundations for family life.  We will think about the baby’s development before and after birth.  We will particularly think about nurturing ourselves so that we can care for ourselves, others and the environment to provide the very best of opportunities for the baby to grow and flourish.  Mention that this is a programme particularly to focus on healthy growth and development of children to give them the best possible start in life.  Parents’ physical health is linked to the child’s health. Parents are important role models because children learn how to behave, what they eat and how active they are from their immediate family. We will explore the importance of parent’s role in providing the environment and opportunities that promote health and give their child the best start in life  Remind people that we will be meeting up again as a group once the babies are born and throughout the first 9 months of their life to see them develop and to be able to offer timely advice to ensure they have the best start in life, and that parents can get on-going support and information.    The specific topics surrounding labour and birth will not be covered in this group so parents should ask their midwife and or GP. |  |
|  |  |  |
| **4** | **GROUP RULES** | **10 mins** |
|  | **Explain** briefly the value of having rules – it helps if we all agree on guidelines for what is and what isn’t OK in the group. This helps everyone feel comfortable and safe.  **Introduce** the idea of brainstorming, which will be used a lot in the group. It is a great way of getting everyone’s ideas. All suggestions are welcome – whether they are useful, funny, extraordinary, original or a near-repeat of what someone else has said. |  |
|  | **Divide** the group into **small groups** with fun group dividers. Facilitators always join groups (see Facilitation Checklist on Pg 17 of new Parent Group Leader Handbook).  BRAINSTORM ON GROUP RULES  **Display** on the flipchart the following heading:  *HOW WE WOULD LIKE TO FEEL IN THE GROUP*  and on the bottom half of the sheet:  *What would help us (****Green****) What would be unhelpful (****Red****)*  **Suggest** an example for each heading (see opposite page 1.8).  **Give** each group a sheet of flipchart paper and pens, and ask them to copy the headings and brainstorm their ideas.  **Suggest** that for every ‘feeling’ in the top half they add a ‘helpful’ and ‘unhelpful’ idea in the bottom half.  After a few minutes, **invite** each group to read out their ideas, and compare them. (The suggestions are usually very similar).  **Discuss** briefly to make sure there is general agreement on what would help to make the group an enjoyable and safe environment for everyone. **Suggest** that you will condense their ideas into a list of Dos and DON’Ts, and bring to next week’s session.  CONFIDENTIALITY RULE  **Make sure** ‘confidentiality’ and the right to ‘pass’ are included: what is said in the room stays in the room. It is important that people can trust each other and not fear that there will be gossip about what is said. This usually comes up anyway.  **Emphasise** that the rules apply to everyone including the group facilitators.  **NB** There is one exception to the confidentiality rule. Parents need to know that if they mention any treatment of their own or others’ children that is a cause for concern, or if there is violence in the home, leaders have a responsibility under the Children Act 2004 to discuss this with the appropriate authority. Nothing will be done without their knowledge and involvement, but the law requires group leaders to take action. This needs to be stated clearly but in a low-key, gentle way, e.g. “I just need to mention that if we are concerned that you or your child are at risk of harm, then we have a responsibility to help keep you both safe. We will always talk to you before we talk to anyone else about you or your child”. (See page 1.6 of the new PGL Handbook for notes).  EXERCISE ON FAMILY RULES  Invite brief discussion on what rules people think are important for creating calm, happy family life. Suggest that under the “For Adults” heading they include their own rules as a couple, spoken or unspoken e.g. not opening each other’s post; household chores allocation etc. Under the “For Children” heading they may include having stories before bed time, or using kind words with each other (not swearing).  This can be a time to think of some healthy family rules such as all eating meals together, doing a physical family activity once a week such as swimming or playing in the park, or only having sweets on Sundays.  Display on the flipchart the following heading  Rules for calm, happy family life  And on the bottom half of the sheet  For Children For Adults  Suggest an example for each heading. Invite contributions and write on flipchart. Process similarities and differences.  Then in two groups discuss the family rules people experienced in their own childhoods and feedback.  Finally ask couples to briefly discuss together which rules they consider will be important for creating a calm, happy family in their new/extending family.  Parents on their own can make pairs or pair with facilitators. | **10 mins** |
| **5** | **NURTURING MY BABY’S HEALTHY DEVELOPMENT AND GROWING BRAIN**  KEY MESSAGE: Babies and young children’s development depend on both the traits he/she is born with (nature) and what he/she experiences (nature). The foundation of healthy development depends on the relationship you have with your child. All areas of development: social, emotional, intellectual language and physical are linked. Each depends on and influences the others.  What you eat and drink now whilst pregnant influences your baby’s health and development.  DISCUSSION: Reflect on own diet and importance of food in the family. Start a quick discussion with one or two of these questions   - What influences what you eat at home and when you’re out? - Do you always eat the food you would like to-if not why not? - If you could choose anything for your meal tonight what would it be…. - Are you going to have this? If not why not? If you are what have you done to make it happen?   Make a list/mind map quickly onto a flipchart of the factors that people mention  e.g. time, money, cooking skills, the rest of the family’s wants and needs, taste, likes dislikes, what’s in the cupboard/available, comfort eating, tradition, religion, culture, celebrations, health* and any others.  **Does health feature on the list? (It may not or may not have been mentioned explicitly–which is* ***not*** *surprising given how much else there is to think about sometimes when organising life, food and meals).*  We will talk more about this list and things that help or hinder healthier eating in week 3 so keep the list until then.  **Note for group leaders**:  **Eating Well in Pregnancy**. A healthy diet is an important part of a healthy lifestyle at any time, but especially vital if you're pregnant or planning a pregnancy. Eating healthily during pregnancy will help your baby to develop and grow, and will keep you fit and well.  You don’t need to go on a special diet, but it's important to eat a variety of different foods every day in order to get the right balance of nutrients that you and your baby need.  There are also some foods you need to avoid for safety. (Healthy eating information is given to all pregnant women at their booking appointment along with a lot of other information.-it may have been overlooked)  Friends and family may have given advice or have strong ideas about eating whilst pregnant.  Women may have also received information from the media, advertising and the internet –not all of this will be reliable, unbiased or based on scientific evidence. Information given by health professionals and in this course and the sources mentioned is reliable and evidence based  Members of the group may have cultural preferences for particular foods to choose or avoid in pregnancy e.g. ‘hot’ and ‘cold’ foods in some Asian groups, ‘Nutriment’ –the advice given on healthy eating in pregnancy here is food group based and so can accommodate these preferences. For example different groups may eat some different fruits and vegetables or prefer one source of starchy carbohydrate over another (e.g. chappatis, rice, maize, noodles, bread, yam potatoes) –however they are all suitable choices from that food group. If you are working with a group from a variety of cultural backgrounds ensure you acknowledge this -don’t just use examples traditional to one group only –if you’re not sure ask them to suggest foods they eat in each group  (OPTIONAL IF TIME ALLOWS: Ask group what information they have received or heard about what they should eat in pregnancy and where they heard it from.)  Look together at:  **‘**Eating Well in Pregnancy’ **hand-out 🕮**  You may have a poster or large picture of the Eat Well plate to look at as well.  Tips to highlight include:   - Have a healthy breakfast every day - Try to eat regular meals to keep hunger pangs at bay; - Eat a balanced diet and try to eat a variety of foods. If you get hungry between meals, try to choose healthy, nutritious snacks including fruit and vegetables and starchy foods, low fat dairy foods e.g. yoghurt and cheese and don’t rely on snacks and drinks high in fats and sugars; - Remember to take your Healthy Start vitamin every day. Ask your midwife if you need more information about these and about Healthy Start vouchers for fruit veg and milk if you are on benefits –see handout; - Follow food safety advice for pregnancy (see handout); - If group have lots of queries or questions or want to discuss food and health more than the brief time allows reassure them that this is just an introduction and we’ll be looking at other aspects in other sessions –meanwhile they have information to take home.   QUIZ/GROUP ACTIVITY  ‘Pregnancy Eating and Drinking Facts – Quiz’ **hand-out 🕮**  Look at the 4 questions below (transfer questions onto cards or flipchart) and answer true or false, then feedback to the whole group.  Discuss one question per group or if time permits all. You can ensure all questions are covered by someone if time is short by starting each group on a different one OR do as a quick group quiz Give the information in the answers as you discuss and to take home as a handout.  1. **What you eat affects you and the baby: True or False?**  **True** - a balanced diet & good nutrition prior to conception & throughout pregnancy will ensure the nutritional demands of pregnancy are met & help improve the chances of a successful pregnancy and healthy mum & baby  2. **Children with parents who are obese are more likely to be overweight/obese? True or False**  **True** - if both parents are overweight or obese there is a 46% chance of a child being overweight or obese compared to 16% if both parents are not overweight and obese (2 to 15 year olds): health survey for England 2008.  3. **It’s important to have full fat milk when you’re pregnant – you need the extra nutrients for your baby: True or False?**  **False** - it is important to have calcium as part of a balanced diet and milk provides a good source of calcium & many other nutrients. Semi skimmed & skimmed milk % low fat dairy products like cheese & yogurt will provide as much calcium (slightly more) as full fat versions without the additional calories. Aim for 2 to 3 portions a day of dairy foods  4. **Alcohol can harm your baby: True or False?**  **True** - alcohol is a toxin and if drunk during pregnancy can cause permanent damage to the baby causing problems from mild learning difficulties through to serious birth defects. It can also cause miscarriage or premature birth. It’s advisable to avoid alcohol consumption during pregnancy & especially during the first 3 months. If you do then limit to 1-2 units once or twice a week. Cutting down on your alcohol whilst pregnant will also help you manage your weight as alcoholic drinks add a lot of calories to your diet. More information about alcohol (and drugs) in pregnancy, how many units are in typical drinks and tips on making changes can be found on these websites**:**  [www.nhs.uk/change4life/Pages/pregnancy-and-alcohol.aspx](http://www.nhs.uk/change4life/Pages/pregnancy-and-alcohol.aspx)  [www.nhs.uk/Planners/pregnancycareplanner/pages/Alcoholanddrugs.aspx](http://www.nhs.uk/Planners/pregnancycareplanner/pages/Alcoholanddrugs.aspx)  [www.drinkaware.co.uk/tips-and-tools/drink-diary](http://www.drinkaware.co.uk/tips-and-tools/drink-diary)  EXERCISE ON BREASTFEEDING OR ARTIFICIAL FEEDING  We want to consider the different ways that babies will be fed after they are born.  The 2 main options are breastfeeding or bottle feeding with artificial formula.  To start parents thinking about the issues around the methods of feeding introduce a discussion.  Prepare a flipchart divided with the following headings:  Breastfeeding Bottle feeding   - Why would parents choose each method? - Any concerns or issues with each method?   Divide the group into small groups of 2 or 3 people and ask them to consider the questions and put their ideas onto post-it notes. Allow 5 minutes for discussion.  Invite the group to bring their post-it notes to the flipchart and add to the relevant section.  Read out the responses but explain that these issues will be discussed in more detail in future sessions. Facilitator to keep post-its for weeks 3 and 6.  PHYSICAL ACTIVITY IN PREGNANCY: Explain that physical activity is important during pregnancy for the healthy development of the baby. Regular light-to-moderate physical activity during pregnancy increases the amount of oxygen delivered to the foetus and reduces the baby’s risk of pre-term birth and low birth weight. Explain that physical activity means any type of body movement – not just formal exercise such as exercise classes or going to the gym. One of the best ways to be more active during pregnancy is to walk more.    Give out Tommy’s “Managing Your Weight in Pregnancy” booklet. | **15 mins**  **10 mins**  **5 mins** |
|  | **Tea and Coffee Break** | **10 mins** |

| **6** | **BABY’S BRAIN DEVELOPMENT**  Explain that a baby’s brain trebles in size in the first year and the brain is laying down its wiring for life, so parents are the sculptors of children’s brains, and three crucial ingredients are: attachment, empathy and attunement.  BONDING WITH MY BABY AND ATTACHMENT AT BIRTH  Suggest that some parents fall in love with their baby at birth; for others it can be gradual. Either is fine.  Mention the Birth Plan  Invite brief discussion to establish if parents are aware of this yet and how important it is that the father or supporter is involved to help create a relaxed, calm environment for the baby to be born into. | **5 mins** |
| --- | --- | --- |
|  | EXERCISE: THE BABY’S 5 SENSES  On a flipchart list: sight, sound, touch, taste, smell.  Suggest that at birth it is ideal to introduce the baby to each sense slowly and one by one. Invite ideas from the group about how each of the five senses can gradually be introduced to the baby.  Display on Board: Awakening the Baby’s Five Senses  **Sight** – low lights; let him/her gaze into your eyes  **Sound** – quite music (perhaps a favourite tune you played to the baby when he/she was in the womb), gentle talk  **Touch** – skin to skin contact  **Taste** – gradual introduction to the breast  **Smell** – defer washing the baby as mother and baby can identify their unique smell for several hours  Read out and/or invite parents to read out in turn if they are comfortable to do this and compare with the group’s flipchart ideas. | **15 mins** |
|  | **Bonding at Birth** |  |
|  | - Both mother and baby need time to recover. Relatives may need to be told to wait for a while before they see you and the new baby. You and one other close person to you should be alone with the baby for at least the first hour. - It should be quite with no distractions or disturbances, avoiding bright lights. You should be as comfortable and relaxed as possible. - Your baby needs to be around you as you provide them with a familiar sound and smell. - The skin to skin contact is especially important. A baby that is laid on their mother’s tummy will automatically inch their way towards her breast. - You and the baby both need to snuggle up and look and listen to each other.   Mention that even with an epidural or Caesarean Section all the above are possible and if the mother has had an anaesthetic then her birth partner can provide these experiences and the mother can join in when recovered.  **Special Care Baby Unit** – should the baby need this important care, then parents can still touch and stroke the baby; talk to him/her and there will be plenty of time once the baby comes out of the incubator to develop attachment. |  |
|  | EMPATHY  KEY MESSAGE: Empathy means tuning in to someone else’s feelings, understanding their emotional point of view. It is the cornerstone of the Nurturing Programme. We don’t have to agree with what the other person thinks – just to be sensitive to the way they feel, and to accept it. An empathic response to children’s moods makes our relationship with them happier and closer. Children who are treated with empathy and respect will learn to be empathic and respectful towards other people.  ROLE PLAY ON EMPATHY  Act the following two scenarios with one facilitator playing the parent and the other facilitator making the baby crying sounds. Use a soft toy/doll or cushion to represent the baby. Hold it in your arms (while co-facilitator “cries”)   a) In a frustrated tone say sharply, “What’s the matter with you? You’re always crying. You’re just trying to wind me up. I don’t know what to do with you”   Process the ‘parent’s’ reaction with group and then invite suggestions as to how the baby might be feeling.   b) The parent cuddles the crying ‘baby’ saying, “Here little one. You seem so upset. I wonder what the matter is – maybe a cuddle is what you need.”   Process reactions again with the group – both the parent’s and the baby’s feelings.  De-role both facilitators: “My name is…..and the colour of my eyes is……”  **Ask** the group to suggest the key components needed for an empathic response, taking the previous role-play as an example.  **Ensure** that eye contact, tone of voice, and gentle touch if appropriate are teased out.  **Display** the Steps for Empathy board, and compare with the group’s suggestions.  *STEPS FOR EMPATHY*   1 Stop what you are doing 2 Give full attention and listen 3 Give appropriate eye contact/body language and facial expressions 4 Try and find the feelings behind the crying 5 Tentatively suggest the feeling behind the crying, e.g. “It sounds like….you seem….you sound/look….” 6 Do use gentle touch if appropriate 7 Keep focussed on the baby’s feelings 8 Avoid over reacting   Display the 4 Constructs Board and say that empathy is the cornerstone of the 4 building blocks of the Nurturing Programme and happy family life. | **10 mins** |
|  | ATTUNEMENT: SERVE AND RETURN  KEY MESSAGE: Attunement is the art of being present and responsive. Tuning into our baby’s signals and reacting sensitively helps to meet their needs so that the baby feels safe, special and contained. It is a vital part of healthy brain development. The baby’s brain is biologically prepared to get responses such as eye contact; attention; gentle touch; the right amount of food etc. It is particularly important to react to signals of distress. This helps the child to gradually regulate their own emotions.  Flipchart:  EXERCISE ON ATTUNEMENT: THE CRYING GAME  Mention that all babies cry – it’s their main language. It will make you feel anxious because that is exactly what it is meant to do – it’s designed to help babies survive. Responding quickly helps him/her feel the world is a safe, friendly place.  **Display** on the flipchart the following heading:  *Ways To Meet The Needs of a Distressed/Crying Baby*  **Divide** the group into **small groups**, splitting them up with fun dividers, or going round the group and dividing them within categories, e.g. apple, lemon, banana; tiger, giraffe, baboon.  **Give out** a pen and some post-it notes to each group, and **ask** them to spend a few minutes writing one example per sheet of any response they might use themselves or are aware of others using – under two headings. Emphasise that all ideas are welcome – whether they themselves think response is OK or not.  **Divide** the flipchart sheet into two columns, and give them these headings:  POSITIVE (**Green**) NEGATIVE (**Red**)  e.g. cuddles, gentle voice e.g. shouting, shaking  **Ask** each group to read out their examples, and to stick each note under the appropriate heading. The group as a whole can support/challenge where each example is placed – **allow** brief discussion. Some examples can go on the line if they are sometimes OK and sometimes not OK (e.g. leaving the baby in its cot and going to another room when at the end of one’s tether)  **Encourage** all post-its to be included, even if some duplicate examples given by another group.  **Invite** comments.  **Explain** that babies who receive kind, loving, gentle responses become healthy, positive, kind people. Harsh responses can wire the baby’s brain for depression or aggression in the long term.  **Suggest** both parents start the bonding process by talking or singing to their baby now, since babies respond to sound from 5^th^ -6^th^ month of pregnancy, and newborn and older babies love nursery rhymes, lullabies and simple songs best. | **15 mins** |

| **7** | **NAME GAME**  **Stand** in a circle. **Check** that all name badges are clearly visible. One facilitator has a soft ball (or beanbag) and throws it underhand to someone in the circle, calling out the name of the person before they throw. That person then throws the ball on to a different person in the circle, calling out that person’s name before they throw, and so on until everyone has received the ball, and throws back to the original facilitator. **Repeat** the game until everyone feels comfortable.  **NB** Combine the Name Game with the Goodbye Circle if you prefer. | **5 mins** |
| --- | --- | --- |
|  |  |  |
|  |  |  |
| **8** | **TIME TO HAVE A GO**  Give Time to Have a Go **hand-outs 🕮**with the following ideas:   - Think about what music you might play during delivery and other ideas for your birth plan. - Think about songs and lullabies you can sing to your baby inside and outside the womb – have a go! Include older children and Dad. - If you want to read more about baby’s brain development try “Why Love Matters” by Sue Gerhardt. - Read through the “Eating Well in Pregnancy” **hand-out 🕮**and see if there are any changes you could make to what you’re eating now. - Read though the leaflets already provided in your birth pack which related to healthy diet and physical activity during pregnancy. Refer parents to section 3 -5 in Tommy’s “Managing your Weight in Pregnancy“ for physical activity advice. | **5 mins** |
| **9** | **FEEDBACK: What did you think of today?**  Give out feedback forms and pass mints. Play relaxing background music whilst forms are being completed; place a container on the floor to collect forms. Thank the group for completing them. If there are literacy issues the whole group could respond to questions on flipchart and facilitator can record responses. | **5 mins** |
|  |  |  |
| **10** | **GOODBYE CIRCLE: Smile goodbye**  Stand in a circle and invite everyone to pass a smile to their neighbour and say “see you next week” and then respond to the statement:  “One thing that was new, or interesting, or stuck in my mind this week was……”  Remind group of their right to pass.  Close session by saying how much you have enjoyed it and that you are looking forward to seeing everyone again next week. | **5 mins** |
|  |  |  |

**WELCOME TO THE WORLD**

**HAPPY Antenatal Programme**

**Week Two**

**A CELEBRATION OF BIRTH**

**STEP BY STEP**

PLAN FOR TODAY

|  | **ACTIVITY** | | | **TIME** | | **MATERIALS NEEDED** |  |
| --- | --- | --- | --- | --- | --- | --- | --- |
| 1 | Welcome | | | 10 mins | | Refreshments, music and player, ‘Welcome’ sign, attractive table cloth, flowers, badges, stickers, felt-tip pens, plan for today, attendance sheet, reward system, etc |  |
| 2 | Icebreaker:  Introductions Bingo | | | 10 mins | | Flipchart, pens |  |
| 3 | Feedback | | | 10 mins | | Group Rules Board |  |
| 4 | A Celebration of Birth | | | 30 mins | | Boards x 2, Memory Books  Baby Names Dictionary  Handouts |  |
|  | | *TEA & COFFEE BREAK* | *10 mins* | | *Refreshments* | | |
|  |  | | |  | |  |  |
| 5 | Being Pregnant in my Family | | | 60 mins | | Flipchart, pens  Handouts |  |
| 6 | Game: Going to Market | | | 5 mins | |  |  |
| 7 | Time To Have a Go | | | 5 mins | | Time To Have a Go Handout |  |
| 8 | What did you think of today?  (feedback) | | | 5 mins | | Feedback forms, pens, basket/container, mints, relaxing music |  |
| 9 | Goodbye Circle | | | 5 mins | | ‘What We Pay Attention to’ Magnets |  |
|  |  | | |  | | Basket |  |
|  |  | | | 150mins | |  |  |

**WELCOME TO THE WORLD**

**Family Links Antenatal Nurturing Programme**

**Week Two**

**A CELEBRATION OF BIRTH**

STEP BY STEP

| **1** | **WELCOME**  **Display** board with ‘Welcome to Family Links Nurturing Programme for Parents’. | **10 mins** |
| --- | --- | --- |
|  | **Greet** group members warmly as they arrive, have background music playing, and offer refreshments. **Ask** parents to make name badges, and to sign the attendance sheet.  **Start** the Icebreaker as soon as enough people have arrived for them to mingle. |  |
| **2** | **ICEBREAKER: Introduction Bingo**  **Give out** a Bingo Introductions **hand-out 🕮** to each parent when they are ready to join in, and **encourage** them to circulate among the group, asking others one question until they have filled the boxes with names and answers. After 5 minutes of this continue.  Go through the session topics briefly | **10 mins** |
| **3** | **FEEDBACK**   - Re-present the Group Rules - Comment briefly on last week’s parents’ feedback - Any questions about diet or physical activity? - Display last weeks’ Time to Have a Go suggestions and invite feedback | **10 mins** |
|  | TIME TO HAVE A GO   - Think about what music you might play during delivery and other ideas for your birth plan. - Think about songs and lullabies you can sing to your baby inside and outside the womb – have a go! Include older children and Dad. - If you want to read more about baby’s brain development try ‘Why Love Matters’ by Sue Gerhardt. - Read through the ‘Eating Well in Pregnancy’ **hand-out 🕮**and see if there are any changes you could make to what you’re eating now. - Read though the leaflets already provided in your birth pack which related to healthy diet and physical activity during pregnancy. Refer parents to section 3 -5 in Tommy’s ‘Managing your Weight in Pregnancy’ for physical activity advice. |  |
| **4** | **A CELEBRATION OF BIRTH**  PREPARING THE CHANGE: OUR HISTORY  KEY MESSAGE: Having a baby can bring back strong memories of childhood. It is important to recognise the influences that the past may bring to us as we become parents.  Say that we will begin by thinking about the significance of names.  Family traditions, cultural uniqueness and history, personal preferences and specific events all play a role in naming children. | **20 mins** |
|  | This **activity** allows participants to share information about their names. Divide the group into pairs using fun group dividers and ask each pair to respond to the following:   a) Who named you? b) Does your name have a special meaning? c) Does your name have a cultural significance?   Allow about 10 to 15 minutes for parents to share responses to the questions.  **Explain** to the parents that each culture and each family has its own traditions and practices related to birth. We are now going to share with each other the way in which birth is celebrated in the culture with which we identify.  **Explain** that culture is made up of one’s ethnicity, gender, religion, traditions, socio-economic level and family of origin. The interchange of these components forms our cultural frame of reference. From our culture comes our values and from our values come our expectations. From our expectations come our code of behaviour (how I expect you to act and how I expect myself to act in any given situation). The way cultures perpetuate themselves is through their children, so children and the birth of children are of great significance to any culture. Most cultures celebrate birth with traditions and activities to mark this most important event.  **Display** the following on a Board:   a) Where were you born? b) Where did you grow up? c) What is your main cultural group? d) Share any traditions, celebrations or practices surrounding birth in your culture.   **Ask** for one parent to start by sharing with the group their answers to the above.  Continue until all parents have shared. If the parents are too shy to start, you begin first and model appropriate behaviour. If parents share first, then all facilitators end the activity with sharing their backgrounds.  If this is a class with partners, have them pair off and share what they have learned from or about each other.  **Note for group leaders**: some parents have had unhappy and hurtful experiences in childhood. Ensure you are providing a nurturing, safe environment for parents to share or to pass.  Have available some baby name books for people to look up their names.  If the group is small (fewer than 10), you can have the group share in a large group setting rather than pairs.    MEMORY BOOK  Offer each parent a book for them to decorate if they wish and to record their thoughts and experiences during pregnancy. They might like to capture anything from this session about their own history and any of the ideas on the **hand-out 🕮**.  **Display Board**: What You Could Put in Your Memory Book  Write the following ideas on the board:   1. Your own history – where you were born; when; how your name was chosen etc. 2. Write about what feelings you are having during pregnancy 3. What do you like about being pregnant? 4. What don’t you like about being pregnant? 5. Some photos of your growing bump 6. What are you most looking forward to about becoming a mum/dad? 7. What are you not really looking forward to? 8. What lifestyle changes you have made during your pregnancy (diet, exercise, smoking, alcohol) and how they make you feel 9. Your birth wishes/plan 10. Your birth story (DOB, time of birth, weight, place etc) 11. Your memories and thoughts of the birth 12. Pictures of the baby 13. Hand and footprints of baby 14. A lock of baby’s hair 15. Have you changed what you eat because you are pregnant: have you made any healthier choices? 16. Have you had special issues regarding foods - cravings, sickness, changes in taste, 17. Have you started doing any new activities?   Also prepare this as a **hand-out 🕮** to give with each Memory Book  There is a **hand-out 🕮** to give at the end with Tips about Common Food and Health Problems in Pregnancy – issues like constipation, food aversions and cravings, indigestion, heartburn and nausea can make life difficult and lead to eating patterns that are not best for mum or baby –so encourage women to look at the advice and seek further help from their midwife or GP if they need to.  Give out the “How am I Doing Diary?” and ask parents to have a go at filling this in as honestly as possible. It is not to be shared with anyone else, and writing something in for only 2 or 3 days is great information for you to look at next week. | **5 mins**  **5 mins** |
|  | **Tea and Coffee Break** | **10 mins** |
|  |  |  |
| **5** | **BEING PREGNANT IN MY FAMILY**  ICEBREAKER: Display on flipchart ‘I used to think I would get pregnant when I was……. years old’   1. **Mention** that pregnancies impact families differently. During the next exercise, the group will get a chance to discuss their pregnancy by responding to open-ended statements. Group members can choose to respond to or pass on any questions. 2. Briefly ask each woman/couple to share the conditions of the pregnancy: a. Was the pregnancy planned or unplanned? b. How did you feel when you found out you were pregnant? c. When did the pregnancy begin to feel real to you? d. (optional) What was his first reaction? e. (optional) How did you think your parents would react? f. How did you feel about telling others of your pregnancy? | **15 mins** |
|  | **Note for group leaders:**  Sharing feelings about the pregnancy can be a difficult experience. If members present issues of concern, proper supportive action should be given be given. It may feel safer to discuss in two groups with each facilitator ensuring trust and the right to pass. |  |
|  | **THE PRESENT**  **CHANGES IN ME AND YOU**   1. **Mention** that pregnancy brings many changes in one’s life. The group will discuss these changes in two groups – one of mums and one of dads.   KEY MESSAGE: pregnancy causes enormous physical, social and emotional changes which impact on the individual and the couple. Many pregnant women say that they do not feel ‘emotionally ready’ to have a baby. They can be surprised by how tired and emotional they feel. Many feel under pressure to be ‘perfect’ and get overwhelmed by other women’s pregnancy advice and birth ‘horror stories’. N.B. Reference: Tommy’s baby charity – Dr Linda Papdopolous. | **20 mins** |
|  | 2. **Mention** that changes usually occur in three areas.   Display on the flipchart the following heading:  Changes in Me and You  Write these three sub heading and give to each group:   a. Physical (P) b. Emotional (E) c. Social (S) |  |
|  | **Beginning** with physical, have each person respond to changes they have experienced or are experiencing as a result of the pregnancy. Ask each person to respond to the following in each area: **Expectant woman**: - One (P) (E) (S) change I note in me is - One (P) (E) (S) change I notice in my partner is Record answers on the flipchart in the (P) (E) (S) columns **Partner**: - One (P) (E) (S) change I notice in my partner is - One (P) (E) (S) change I notice in me is **Compare** the two charts and invite discussion If not identified in the participants’ responses, ask the group to discuss the following: a. Changes in sexual activity b. Changes in communication between partners c. Changes in perceptions of self and others d. Changes in priorities – work, money, leisure time, friendships **Display** the board ‘The 4 Constructs’ **Present** the 4 Constructs board and mention that this session has related in particular to the Self-Esteem/Self Awareness building block of the Nurturing Programme **Give** out **hand-out 🕮** ‘Changing Me and You’ about physical; emotional; and social changes. **Pregnancy and Health: Myth Busting Activity** Produce a copy of this table for each person or small group or put on a flip chart and get people to tick or put a sticker in the column they think is the right one. Go through the answers and give hand-out to take home –there is quite a lot of information for people to digest so suggest they can look at again later –they may want to think about sharing it with others at home if they have any of these beliefs. **Myth or Fact? –tick the box** \| **True** \| **False**; **You need to eat for two in pregnancy** \| \|; **You need to eat more often when you are pregnant so extra snacks are important** \| \|; **You can’t do any exercise when you are pregnant** \| \|; **Pregnant women who are very overweight, should try to lose weight before the baby is born** \| \|; **Being more active and eating healthily now will help me after the birth** \| \| **Answers for myth busting activity (give as hand-out 🕮)** 1. **You need to eat for two in pregnancy: True or False?** **False** – amazingly up until the last 3 months of pregnancy your baby grows well without any extra calories. During the last 3 months an additional 200kcals is needed. However, most women meet this through natural slight changes in appetite and activity as their pregnancy progresses from 6 to 9 months. **Some examples of foods containing 200kcals** - ½ can of creamed tinned soup - 2 slices of bread - 1 banana and 1 apple - 1 glass of semi-skimmed milk and 1 pot of yoghurt 2. **You need to eat more often when you are pregnant so extra snacks are important: True or False?** **False** - you only need a small amount of extra calories in the last 3 months. (see above). If you do want to snack then try and choose healthier options (ones that are not fattening and also provide you with some other nutrients). Examples are fruit and low fat yogurt. You might want to try a drink first –often we confuse being thirsty for being hungry. **(There are more ideas on the Smart Snacks hand-out 🕮. Give out at the end).** 3. **You can’t do any exercise when you are pregnant: True or False?** **False** – you can still exercise in pregnancy and this will help keep you well and active. Moderate – intensity physical activity is recommended like brisk walking and swimming. 4. **Pregnant women who are very overweight, should try to lose weight before the baby is born: True or False?** **False** - Dieting (i.e. restrictive diet aimed at weight loss) is not recommended during pregnancy as it might harm the health of the unborn child. Pregnancy weight gain is due to: - Increase in fat and protein stores in the body to be ready to produce milk for the baby - Increased volume of blood - Weight of the baby, amniotic fluid and placenta You can manage your weight by eating well and keeping active throughout your pregnancy and this may help you to achieve a healthier pregnancy, safer birth and healthier baby. 5. **Being more active and eating healthily now will help me after the birth: True or False?** **True.** By eating sensibly during pregnancy you may not have so many extra pounds to lose afterwards and you will already have established some better habits which will be easier to continue. You may also feel better in yourself too. **Notes for group leaders**: significant issues regarding pregnancy will likely surface. Be prepared to be supportive. **Healthy Weight Gain** There is currently much debate over how much weight gain is ok is pregnancy. In America guidelines suggest that women who are very overweight at the start of their pregnancy (i.e. have a Body Mass index (or BMI) of 30 or more) should gain no more than 5- to 9kg. (9 kg is equivalent to about 1stone 6 lbs ) Most weight gain will be in the second and third trimester of pregnancy. | **10 mins** |
| **6** | **MY THOUGHTS ABOUT PHYSICAL ACTIVITY IN PREGNANCY**  DISCUSSION  In groups of mums and dads discuss what their **culture and families** say about whether they (or their partner) should do physical activity whilst they are pregnant. What do **they** think and feel about them or their partner doing physical activity whilst pregnant?  Ask each group to think of three reasons not to do physical activity whilst they/their partner are pregnant, what are their fears/worries? And three reasons to do physical activity during pregnancy – what might be the benefits?  **Flip chart**  Divide the flip chart into two vertically. Write **Fears/worries** as one heading and **Benefits** as another heading. Write up the fears and benefits that each group has come up with and add some other common fears and benefits. Be sure that the list includes:  **Fears/worries**  I might have a miscarriage It might harm the baby  I will not have any energy left to do other things I might hurt myself  **Benefits**  More energy Fewer aches and pains & less swelling in legs  Fewer problems sleeping Less anxiety and depression  Reduce length of labour Decrease delivery complication  Reduce risk of pre-eclampsia Lose weight more quickly after baby’s birth  Help you to recover more quickly after baby’s birth  Reduce risk of gestational diabetes – or helps to control gestational diabetes  Reduced risk of premature birth and low birth weight  **Take time going through each fear and worry and dispelling them.**  KEY MESSAGE: the risk of miscarriage is very low after 14 weeks and evidence shows that doing light-to-moderate physical activity does not increase risk of miscarriage or harm the baby. In fact it can reduce the risk of premature birth and low birth weight. Most pregnant women will have times when they feel very tired but many find that doing light-to-moderate exercise actually makes them feel more energised. Even a short walk for 10 minutes counts. You don’t have to do strenuous or difficult exercise where you might hurt yourself to get the benefits of exercise during pregnancy. Remember to wear shoes that support your ankles, which can twist more easily during pregnancy; low or no heels are best. Also wear a good supportive bra and loose comfortable clothing. Drink plenty of water and take water with you when you go out to exercise. On hot days do your exercise when it is cooler, in the morning or evening. **You might get conflicting advice from different sources (friends, family, internet or books) the advice today is sound, up-to-date and evidence based.**  **Don’t exercise:** to exhaustion; if it is very hot; when you feel unwell; if you have deep pelvic pain; if you have any vaginal bleeding; if you get excessive shortness of breath or chest pain or if your doctor or midwife has told you to stop exercising.  This information is in your “Being Active in Pregnancy” **hand-out 🕮** this week. Next week we will be thinking about different types of activity that you might like to do and talk some more about how much activity you should do  . | **20mins** |
| **7** | **GAME: ‘I WENT TO MARKET AND BOUGHT’**  Go round in a circle and invite someone to buy an item of healthy food – everyday; funny, outrageous, beginning with the first letter of their name e.g. I’m Chris and I went to market and I bought a cucumber; I’m Mumtaz and I bought a melon…..  **TIME TO HAVE A GO**  Give out **hand-out 🕮** with the following ideas:   - Continue talking and singing to your baby - Begin your Memory Book - Think about what and how you eat, when and how you are active, food and activity by completing the diary **hand-out 🕮** to bring back next week. This is just for your interest– you will not have to show it to anyone but it will help you get the most out of next week’s session - Complete ‘Being Active in Pregnancy’ worksheet and share it with family/partner to let them know about the benefits of physical activity in pregnancy. - Think about whether anyone at home or in your family worries about how much you eat or exercise –you could share the information from the myth busting activity with them. - Smart Snacks **hand-out 🕮** - Common Problems in Pregnancy **hand-out 🕮** | **5 mins**  **5 mins** |
| **8** | **FEEDBACK: WHAT DID YOU THINK OF TODAY?**  **Give out** feedback forms and pass mints. **Explain** that this is an opportunity for everyone to comment (anonymously) on today’s session. **Remind** the group of the topics you have covered. **Emphasise** that we take their views seriously, and want to know what they really think and feel about the session. **Place** a container on the floor, or collect the forms as they are completed. | **5 mins** |
| **9** | **GOODBYE CIRCLE**  Pass a basket round with magnets ‘What We Pay Attention To’. Invite everyone to take one and say, ‘one thing which was new, interesting or struck me this week was…..’.  **End** by saying how much you’ve enjoyed the session and that you look forward to seeing everyone next week. | **5 mins** |

**WELCOME TO THE WORLD**

**Week Three**

**PERSONAL POWER, SELF ESTEEM AND HEALTHY CHOICES ABOUT FOOD**

**STEP BY STEP**

PLAN FOR TODAY

|  | **ACTIVITY** | **TIME** | **MATERIALS NEEDED** |
| --- | --- | --- | --- |
| **1** | Welcome | 5 mins | Refreshments, music & player, ‘Welcome’ sign, attractive table cloth, flowers, badges, stickers, felt-tip pens, plan for today, attendance sheet, reward system, etc. |
| **2** | Gentle Exercise | 10 mins |  |
| **3** | Feedback: Change Places | 5 mins | Group Rules Board |
| **4**  **5** | Time to Have a Go  Influences on Behaviour  Self Esteem | 5 mins  25 mins | Memory Books  Boards x 2  Name Dictionary |
|  | What’s in a Name? |  |  |
|  |  |  |  |
| **6** | Personal Power | 20 mins | Flipchart |
|  |  |  |  |
|  | *TEA & COFFEE BREAK* | *10mins* | *Refreshments* |
|  |  |  |  |
|  |  |  |  |
| **7** | Self Esteem and Physical Activity | 15 mins | Hand-out |
| **8** | Healthy Eating Choices  Feeding my Baby | 25 mins  15 mins | 4 Constructs Board  Flipchart, Hand-outs,  Booklets and DVD on breastfeeding |
| **9** | Relaxation (Optional) | 5 mins |  |
|  |  |  |  |
| **10** | Time To Have A Go | 5 mins | Time To Have A Go Hand-out  Book on Breastfeeding  Relaxation Hand-out |
| **11** | What did you think of today? (Feedback) | 5 mins | Feedback forms, pens, basket/ container, mints, relaxing music |
|  |  |  |  |
| **12** | Goodbye Circle | 5 mins  **155 mins** | “Praise is Magic” Magnets  Basket |

**WELCOME TO THE WORLD**

**HAPPY Antenatal Programme**

**Week Three**

**PERSONAL POWER, SELF ESTEEM AND HEALTHY CHOICES ABOUT FOOD**

**STEP BY STEP**

| **1** | **WELCOME** | **5 mins** |
| --- | --- | --- |
|  | **Greet** group members warmly as they arrive, have background music playing, offer refreshments and name badges. |  |
|  | When they are all settled, **go through** the topics for the session briefly. |  |
|  | **Display** the Group Rules and the Four Constructs charts. |  |
| **2** | **GENTLE EXERCISE** | **10 mins** |
|  | Explain that we will do some gentle exercises that mum’s can incorporate into their everyday activities but all family members can join in too. Ask everyone again to take care of themselves. They should not do these exercises if they have been told by their doctor/midwife **not** to exercise. Reassure them that these exercises are safe during pregnancy.  Describe “zipping up” before you start. This is a way to strengthen your core muscles to protect you back and stomach from injury. It is easy to do, imagine a thread is attached to your belly button from the inside and it is pulling your belly button towards your spine, tighten your muscles inwards and upwards. You will still be able to breathe normally. |  |

|  | PELVIC FLOOR EXERCISES  Explain that pelvic floor exercises are very important to do during pregnancy but are also important for all people so fathers, supporting partners etc. can join in too.  Your pelvic floor muscles support and protect your bowels, uterus and bladder. These muscles can loosen during pregnancy and women sometimes leak urine with they exercise, cough or laugh.  This exercise will strengthen your pelvic floor muscles. You can do this exercise either sitting or standing.  Remember to ‘zip up’.   - Squeeze and draw in your back passage at the same time as you close up and draw your front passage upwards. - You may find it helps to imagine you’re stopping a bowel movement, or stopping your urine flow when you pee. - Do it quickly, tightening and releasing the muscles immediately. - Then do it slowly, holding the contractions for as long as you can (but not more than 10 seconds) before you relax. - Do five lots of pelvic floor quick squeezes and five lots of slow squeezes   Suggest to the group that they do the pelvic floor exercise five times a day and build up to doing ten squeezes five times a day  There are more easy toning exercises in the Tommy ‘Managing your Weight in Pregnancy’ leaflet which was given out in Session 1.  LET’S GET MOVING  Explain that to help the heart and lungs get stronger which will help them with stamina during labour then need to do activities which raise their heart rate for at least 10 minutes continuously. This level of activity is also required for other health benefits such as lowering blood pressure, cholesterol and weight loss. A good way to check that you are working at the right level to improve heart and lung fitness and general health is to do the talk test. You should be breathing a little harder than usual and still be able to talk.  Now ask them to stand in a circle, “Zip Up”, and do the following whilst talking through nursery rhymes, march on the spot, bend knees, wave hands above their head, punch arms out in front (for about 2 mins). Ask the group if they are breathing harder, can they still talk?  **KEY POINT** - You can use the talk test when you are out and about exercising to check you are working at the right level for you.  Give out the ‘Physical Activity Ball for Pregnancy’ **hand-out 🕮**and talk though each segment briefly, going over the guidelines for pregnancy. E.g. for aerobic activity: If you have not been doing much physical activity before now, start by doing just 15 minutes a day, three times a week. Gradually build this up to doing 30 minutes a day four times a week to daily. You can do your 30 minutes in 3, 10minute chunks if you like. Suggest that swimming or aqua-exercises are good during pregnancy as the water will support mum’s bump, taking the strain off the back. Go over the guidelines and examples of strength and flexibility exercises and lifestyle exercise which are detailed on the **hand-out 🕮**. Emphasis to the group that any physical activity is better than none.  Draw the group’s attention to the Inactivity segment. Explain that spending long amounts of time sitting has negative effects on health, e.g. increased risk of diabetes, heart disease, overweight, and that no-one should sit for no longer than 60 minutes without having a break from sitting. |  |
| --- | --- | --- |
|  |  |  |
|  |  |  |
| **3** | **FEEDBACK** | **5 mins** |
|  | **Change places if you:**   - watched a favourite TV programme this week; - made some healthy changes to your diet this week; - had some fruit each day this week; - had some quiet music on and listened to it sitting/lying down; - you’ve noticed the different ways your baby moves in response to noise; - have chosen a healthier snack or drink than usual; - had breakfast every or most days this week; - have got a nose on your face; - have taken your Healthy Start vitamins ! - have put anything in your memory book; - have done some physical activity this week |  |
|  |  |  |
| **4** | Comment briefly on last week’s parent feedback.  Display last week’s ‘Time To Have A Go’ suggestions and invite feedback.  **TIME TO HAVE A GO:**   - Continue talking and singing to your baby. - Begin your Memory Book. - Think about what and how you eat, when and how you are active, food and activity by completing the diary **hand-out 🕮** to bring back next week. This is just for your interest– you will not have to show it to anyone but it will help you get the most out of next week’s session. - Complete ‘Being Active in Pregnancy’ worksheet and share it with family/partner to let them know about the benefits of physical activity in pregnancy. - Think about whether anyone at home or in your family worries about how much you eat or exercise –you could share the information from the myth busting activity with them. - Smart Snacks **hand-out 🕮** - Common Problems in Pregnancy **hand-out 🕮** | **5 mins** |

| **5** | **INFLUENCES ON BEHAVIOUR**  PERSONAL POWER AND SELF ESTEEM  KEY MESSAGE: Our Personal Power is the emotional strength we have to get our needs met, and to make choices in our lives. It isn't about being physically strong, or about having power over other people. It's our inner strength - power for ourselves - not power against others. This idea is new to many people, so will need some time to explore.  **Display** the Influences on Behaviour board (see updated PGL Handbook page 4.4), and use it to **introduce** these linked topics. The group will look first at Self-Esteem, then at Personal Power, and lastly at Choices and Consequences.  SELF-ESTEEM  KEY MESSAGE: Self-esteem is the second bit of the triangle. The way we think and feel about ourselves- our self-esteem - is first shaped in childhood by the events we experience and the way we are treated.  **Display** the Cycle of Feelings and Behaviour board (see Updated PGL Handbook page 4. 7). It is also in The Parenting Puzzle (PP91). **Suggest** that the way others behave towards us affects the way we feel about ourselves and the way we in turn behave towards other people. As adults we can think more clearly about others’ attitudes towards us, and perhaps challenge them, but they still influence us. And we also affect those attitudes by the way we ourselves behave. On days when we are feeling fine, we’re more likely to be helpful and kind to others - and to get a positive response from them too. Invite group's agreement and comments on this idea.  **Invite** the group to suggest where on the cycle change in children needs to begin. **Explore** gently the possibility that many of us think that if children would behave better, the problems we have with them would melt away. But until children feel good about themselves, it is hard for them to change, so the place in the cycle where change needs to begin is with how we and others behave towards them. **Ask** which of the Four Constructs this refers to.  Children will grow up somehow, but the conditions adults provide can make all the difference. A baby is like a tomato seed. With the right balance of nurturing: soil, light, water, nutrients - the tomato plant reaches its full potential, it is full of vigour and has large, healthy fruit. With poor care it will still grow, but with a struggle, and the result will be a puny plant that is susceptible to disease, and produces small, poor fruit. (Facilitators who are artistic can draw a strong and a weak plant on the flipchart for comparison).  With children, one of the most important ingredients in healthy growth is ensuring that we build their self-esteem. Negative messages lower self­ esteem; there are also ways of increasing it.  WHAT’S IN A NAME AND NICKNAME?  **Introduce** briefly the idea of labelling.  We may be labelled from the moment we are born, e.g.: | **25 mins**  **10 mins**  **15 mins** |
| --- | --- | --- |
|  | “He looks just like his father",  “She doesn’t sleep as well as her sister did at 4 weeks”.  Both over-identification and undesirable comparisons are limiting to a child. Almost everyone is labelled as a child (some children's T-shirt slogans encourage labelling!) and we often carry the labels with us into adulthood. Labels may be positive or negative; positive labels are less damaging, though still not helpful (a child labelled 'good' may be burdened by the duty of never causing a stir; a 'neat and tidy' child may never feel able to play in sand or mud); negative labels (clumsy, fat, stupid, nosy, bad-tempered, useless at sport or drawing) are always a burden. |  |
| 1. | Ask each parent to respond to the following statements. It is not important that partners agree on the same names, but should feel free to share their preferences independently.  **Write** the following statements on a board:   a. “If my baby is a girl, I would like to name her . This name means to me and I like this name because.” b. “If my baby is a boy, I would like to name him . This name means to me and I like this name because.” c. “A nickname I had growing up was. I felt about this name”. d. “A nickname I may give my child is .” e. "A label I was given when I was a child was and I felt __.” |  |
| 2. | The purpose of this activity is to help parents become aware of why they are attracted to certain names for their baby and the kinds of expectations that their name choice may imply. Discuss the fact that while it is okay to hope that their baby will be endowed with certain abilities or qualities, that they must be open to discovering their baby's own unique personality. |  |
| 3.  4. | Often the choice of baby's name may indicate the personal characteristics that parents may be expecting their child to display. If named after Dad, he should be "a chip off the old block." If named after a movie star or rock singer, the expectation is that she be physically attractive and successful. The meaning of a name may indicate the kind of hopes a parent has for their child.  Sometimes the inability to choose a name by the third trimester may indicate confusion, indecision, unwillingness or inability of the parent to bond with the child or pregnancy. Delay in naming a child may also be a conscious decision to wait until the baby is born to determine a name that "fits" best. In this instance, not "knowing" is not "bad. |  |
|  | **Note for group leaders:**  1. If a parent is assigning a negative label to a child, try to help them redefine the quality as a positive attribute. For example, a dad wants to name his son "Butch" because he is expecting him to be a real "hell-raiser." You might redefine this quality by saying, "You want your son to be strong and assertive." Can he be strong and assertive without raising "hell"? |  |
|  |  |  |
|  | 2. Support parents in the cultural pride they show by choosing an ethnic name for their baby. Ask questions regarding the name's meaning and attributes. Encourage the fostering of the positive attributes a name implies. |  |
|  | 3. Discuss the concept of "nicknames." What are their functional purposes and why do parents use nicknames? |  |
|  |  |  |
|  | 4. Have a name dictionary available and offer to lend it. |  |

| **6** | **PERSONAL POWER**  PERSONAL POWER AND POSITIVE THINKING  KEY MESSAGE: We use our Personal Power according to how we feel about ourselves/our self-esteem. People with high self-esteem tend to act in a positive, caring way towards others and themselves. It is difficult for people with low self-esteem to do this (they tend to behave negatively towards others and themselves, or try to please others at any price). The choices we make about our behaviour and our lives reflect our self-esteem through our positive or negative use of Personal Power, so the concepts are closely connected. | | **20mins** | |
| --- | --- | --- | --- | --- |
|  | EXERCISE: POSITIVE THINKING  **Explain** to the group that during pregnancy the mother’s emotional outlook affects the baby’s inner world through the release of many hormones including adrenaline and stress hormones. Use of relaxation and positive self-talk through affirmations may enhance the baby's existence *in utero*.  If we have negative thoughts we can help to get rid of them by turning them around with a positive thought or saying something positive.  Brainstorm with participants a list of worries or negative thoughts they may be having about the baby or pregnancy. Write them on a flip chart for everyone to see, but leave a space between statements.  Now suggest to group that we will try and re-frame their worries and negative thought about the baby or pregnancy into positive alternatives.  **Negative**: I hope my baby isn't deformed or sick.  **Positive**: My baby is healthy and strong  Suggest that positive thinking won't solve all our problems but it helps us to direct our energy into positive thoughts and actions which is good for our health as well as our baby's health.  Invite parents to think in silence one positive thing about themselves; e.g. I’m kind; I’ve got nice eyes; I’m a good cook; I’ve got a wicked sense of humour. Ask them to say one positive thing to their partner: "One thing I like about you in this pregnancy is….... "  If parents are on their own encourage them to say a positive thing about themselves or invite the group to say something positive to them.  Suggest they give themselves one positive message a day. | |  | |
|  | **Tea and Coffee Break** | | **10 mins** | |
|  |  | |  | |
|  |  | |  | |
| **7** | **SELF-ESTEEM AND PHYSICAL ACTIVITY**  Give out the ‘Physical Activity Self-Esteem’ **hand-out 🕮** to the group. There are many people who do not feel confident about doing physical activity for many different reasons. What do you feel worried or anxious about when you think of doing physical activity? Here are some examples of people’s worries on the flip chart: are any of them true for you?  “I am worried that I won’t be able to do the exercises in a class and everyone will laugh at me”  “I am worried that someone who knows me will see me exercising”  “I am worried that people will look at me because I am overweight”  “I feel anxious at the thought of wearing sports clothes”  “I feel anxious if men are around when I am doing physical activities”  Put a mental tick next to those that are true for you. Do you have any other worries? Have a think; you can write them down in your memory book if you like. Does anyone want to share any of their physical activity worries?  Open up a group discussion. What could participants do to overcome their worries about physical activity? Suggestions may include:   - self-talk (repeat positive thoughts about yourself); - imagery (imagining feeling confident and positive); - wearing comfortable lose fitting everyday clothes instead of ‘sports’ clothes; - going to all women classes/groups, - doing exercises inside the home (pregnancy yoga DVD); - doing everyday physical activities such as walking to the shops rather than structured exercises classes; - choosing places where you feel safe and comfortable to be active rather than unsafe and uncomfortable. | **15 mins** | |  |
|  | MINGLE ACTIVITY AND DISCUSSION  Remind the group that physical activity includes both ‘formal’ exercise such as going to an exercise class, and ‘informal’ activities such as walking to the shops, taking the stairs instead of the lift. Ask the group to look oer their ‘Physical Activity Ball for Pregnancy’ **hand-out 🕮** and think of some other activities that they do or could do which would fit into the activity segments of the ball.  Ask everyone to stand up and find a partner, decide who will go first.  Think of a list of physical activities that you **could** do this week. They might be as simple as walking to the shop, or taking the stairs rather that the lift. Facing your partner you will complete the sentence “one physical activity that I **could** do this week is…”  For example: “one physical activity that I **could** do this week is walk to the doctors for my appointment”.  Your partner will then share one physical activity they **could** do with you. Make your answers short and quick. Raise your hand and find another partner to share with. Continue sharing with different partners until I ask you to stop. Make sure you start by sharing easy activities that you could do, when you run out of easy ones move on to ones that you might find more challenging. As you go around you can use some of the activities that the others share with you if you like.  Now think over all the activities that you just shared and heard. Choose one of these activities that you are willing to do this week. Choose sensibly; think about how, when and where you will be able to do it. When you are sure of your chosen activity turn to your partner and complete the sentence “the physical activity that I **will** do this week is…”  For example: “the physical activity that I **will** do this week is going for a 10 minute brisk walk”.  Sit down and close your eyes or look down. Picture yourself doing your chosen activity: what you look like, where are you, what you are wearing. Imagine that you are feeling happy, confident, positive and are enjoying doing that activity. In your mind, say to yourself, ‘I am happy, positive and confident and I am good at doing my activity’. Let the group sit in stillness for a moment. |  | |  |

|  | | CHOICES AND CONSEQUENCES  KEY MESSAGE: Explain that as already mentioned, if we feel good about ourselves we are likely to make choices that build on the good feelings. And of course if we don't feel good about ourselves we are more likely to make poorer choices. However as discussed we can use our Personal Power to make better choices even when we are feeling low. Explain that we will explore choices around eating and feeding the baby | |  | | |
| --- | --- | --- | --- | --- | --- | --- |
| **8** | | **HEALTHY EATING CHOICES: FEEDING ME AND MY BABY IN PREGNANCY**  **Note to practitioners:** There are 3 linked activities here. The main aim is to give participants a chance to start to think about how they can use the healthy eating information they have to think about the practicalities of healthier lifestyle choices and develop their own strategies & skills for putting them into practice and overcoming problems. We will spend more time on aspects of food and health in later sessions  COMPARING OWN EATING PATTERNS TO HEALTHY EATING ADVICE –WHAT’S GOING WELL WHAT’S NOT?  Have a picture/diagram of the ‘Eat Well Plate’ visible or refer to the **hand-out 🕮** from week 1. You can use a poster or just draw a quick version onto a flipchart like the one below to represent the proportions of different food groups we should be eating for a balanced diet. You can add pictures of foods if you feel creative, but it isn’t essential!    **Note for group leaders:**  If you do create any visual aids yourself remember to include food choices suitable for the culture of your group (e.g. a chappati and rice in the starchy section). Try to avoid using pictures of foods that aren’t considered Halal or are thought unclean by some communities (e.g. pork chop): use chicken instead. Also avoid foods which should be avoided in pregnancy (e.g. blue cheese).  If you are using a pre–printed version be aware that not all foods may be suitable in pregnancy and that members of your group might want to suggest other examples of foods they typically/traditionally eat in their family/culture/ ethnic group: e.g. yams and plantains, mangoes dumplings, maize, dahl etc.  GROUP DISCUSSION OR INDIVIDUAL REFLECTION  Ask group members to look briefly at their food and physical activity diary which they completed last week (or if they haven’t got this, just to think about what they ate over last few days or yesterday).   - Think for a moment about how what participants eat compares to the ‘Eat Well Plate’. Any comments or thoughts? Any surprises? - Are they in about the right proportions? Anyone got too much in one group, too little in another?   People often reflect that they are eating too little fruit and veg and too much fatty and sugary food.  Over the last few weeks we have looked at making healthier choices about what we eat and drink. In week 1 we made a list of some of the things that affected what we choose to eat: show this flipchart kept from week 1. Anything to add? What got in the way (barriers)? What helped you choose healthily (solutions)?  MAKING HEALTHIER CHOICES  You will need flipchart paper, pens, blu-tac and a wall! This is suggested as a moving exercise, but could equally well work by passing the sheets round the groups.  Display 4 or 5 pieces of flipchart paper, each with one ‘barrier’ written on it (some examples are below or use some from week 1).  Organise one small group around each piece of flipchart paper and ask them to write as many possible ideas for solutions or ways to overcome the barrier as they can in 2 minutes. Emphasize that different solutions may work for different people or at different times –what we want is a good list to pick from!  After two minutes, move the groups round and ask them to look at other sheet(s) to see what’s written and add any new ideas. Again don’t spend too long. Using the list below or your own ideas you can suggest key ideas that are missing at this point  Leave the sheets up for people to look at later/next week.  Suggest that this can be a useful way of thinking up new ways round things that get in the way of making healthier lifestyle choices at home: you can either do this alone (e.g. using the Memory Book) or in discussion with family. | | **25 mins** | | |
|  | **Barrier/Obstacle (examples)** \| **Possible solutions** **(just some of what group might come up with)**; Lack of time \| Plan ahead/know what you’re going to eat for a few meals ahead Quick and healthy cooking techniques and meal ideas (stir fry, grill, microwave); Lack of £ \| Maximise income: benefits advice/healthy start vouchers, older children accessing free school meals Planning food budget and meals in advance, sticking to shopping list, avoiding last minute panic buys and takeaways Where are the cheaper food shops? Shop in local markets/food co-ops Buy in-season, look out for offers etc Share economical meal ideas with friends Use own and economy brands of some foods Know how to store food and use leftovers safely Cut down on waste; Don’t know how to prepare/ cook it \| Ask for advice & help from friends/family Share ideas and recipes with friends Get together with a friend and have a go Get an easy everyday cooking book from library/use internet recipes Change4Life website has easy recipes and meal ideas Make a list of meals that you can make – probably more than you think–and look at the hand-outs. Lots of swaps don’t need more cooking Cooking lessons and ‘Cook and Eat’ group, Ministry of Food/local Children’s Centre; **Other Barriers** \| **Other solutions**; Family likes and dislikes Daily routines and habits Transport Access to/into shops Food preferences Living on own Unmotivated Stress eating/can’t resist temptation Etc! \| Planning Talking with family –enlisting their support. Sharing lifts Use your diary to identify what, when and why you eat and have coping strategies for weak moments Think of non-food alternatives when you need a treat: e.g. manicure, long bath etc. Identify easy food swaps e.g. healthier options (see Healthy Eating Choices **hand-out 🕮**and Smart Snacks **hand-out 🕮**from session 2) Food not immediately accessible to nibble on: put away unfinished food Make a list of the benefits of healthier eating –and the consequences of not changing – and stick it where you can see it Give out the **Healthy Choices hand-out 🕮**and briefly review the information on it. For each food group there are ideas about which foods to limit together with healthier things to swap them for. Swaps can be about choosing: - **a healthier version** (e.g. swapping sugary drinks for sugar-free alternatives); - **a different amount** of the food you have (portion size). For example, have a bigger portion of veg or salad, just one chocolate: not the whole box! - **how often you eat it** (e.g. reducing crisps as a snack to once a week and increasing fruit as a snack to daily); - **how it is cooked** (e.g. choosing to grill rather than fry or to use a spoon to measure oil into a curry so you can see how much you use rather than pouring from the bottle). One way of putting this into practice is to use the list to identify just 2-3 things you want to change. You can start with the things that are easiest or that you have most control over: for example, the food you eat at one mealtime may always be made by/decided upon by someone else (e.g. at work or by mother-in-law) so changes here might need their help. Meanwhile you could easily choose a healthier snack at your mid-morning break. GROUP CHALLENGE: HEALTHY MEAL IDEAS **(Optional – if you have time)** Show the group the meals/snacks in the first column (you could write them onto cards (or paper plates) and hand one to each group). Ask them to suggest swaps and changes to make them healthier. (Get small groups to each look at a different one and share their suggestions). **Meal /Snack** \| **Possible healthy swaps**; Toast, thickly spread with margarine and a fried egg \| Wholemeal bread Thinly spread with margarine Boiled or poached egg; Sugar coated cereal with full fat milk Tea with 3 sugars \| Unsweetened cereal –try a wholegrain version Semi skimmed milk Banana or a few raisins Tea with less/without sugar or try sweetener; White roll, sub or teacake cheese and mayonnaise, large bag of crisps Strawberry milkshake drink (500mls) \| Lower fat filling e.g. tuna, cold sliced meat, thinly sliced and/or lower fat cheese Add some salad/sliced tomato Low fat salad dressing or mayo Try wholemeal roll (it’s more filling) Swap crisps for fruit/small portion dried fruit Choose unflavoured milk, sugar free drink, water; Fried chicken Chips Chocolate mousse \| Grilled or stir-fried chicken Add veg/salad Oven chips or jacket potato Small low fat yogurt; Chicken curry with 2 chapattis Cola drink \| Remove skin and fat from chicken before cooking Use less oil to cook –measure with spoon Add more vegetables/dahl/salad Diet cola or water; Large hot chocolate topped with cream and several digestive biscuits \| Skip the cream! Try low calorie hot choc drink Make with semi skimmed milk or water Have just 1 biscuit or have an orange or a few grapes instead **TOP TIP:** Flavours of food transmit to the baby via the amniotic fluid. So a varied diet in pregnancy will help to prime the baby to enjoy a wide range of food and thereby reduce ‘fussy eating’ tendencies. **TOP TIP:** Suggest that it is worth consulting GP or midwife about Healthy Start vitamin supplements and vitamin D which is important to prevent bone problems like rickets in the baby and in mum in later life. Remind about earlier **hand-outs 🕮**: - Eating Well in Pregnancy (week 1). - Smart Snacks (week 2) **Display** the 4 Constructs Board and suggest that the building blocks of Self Awareness and Positive Discipline have been involved. | | | |  | |
|  |  | | | | |  |
|  | **FEEDING MY BABY** KEY MESSAGE: You will probably have already made some changes which you think will help your baby lead a healthy life. You may be taking things easy; wearing different clothes; eating differently; have stopped smoking and drinking since alcohol and smoking when pregnant cause high levels of anxiety and stress in a baby. So let’s consider how you plan to feed your baby. ADVANTAGES AND DISADVANTAGES OF BREAST AND BOTTLE-FEEDING Following some discussion in week 1 we want to spend more time thinking about the advantages and disadvantages of different feeding methods from the point of view of mothers and babies. ACTIVITY USING FLIPCHART Divide into 4 groups using group dividers. Hand out some prepared flipcharts ready to collect ideas. Ask one group to consider the advantages and disadvantages of breastfeeding to the mother, another to consider the advantages and disadvantages of breastfeeding for the baby. The third group will consider advantages and disadvantages of bottle feeding for the mother and the last group will consider advantages and disadvantages of bottle feeding for the child. Ask group members to discuss and collect their ideas on their flipchart. Briefly go through some of the ideas on the flipchart but moving onto Activity 2 will answer any further questions and cover more information. Group suggestions may include: **Breastfeeding** \| \| \| \| \| \|; **Baby** \| \| \| **Mother** \| \| \|; **Advantages** \| \| **Disadvantages** \| **Advantages** \| \| **Disadvantages** \|; **Complete food**: once established breast milk provides complete nutrition until around 6 months of age \| \| Difficult to know how much breast milk has been consumed \| **Helps with post-pregnancy weight-loss**: breastfeeding uses up c.500 extra calories a day so mums who breastfeed often find it easier to lose their pregnancy weight \| \| Demands on the mother’s time – day and night \|; **Immediately available** at the correct temperature without additional preparation \| \| \| **Reduces fertility** temporarily \| \| Physical discomfort from engorged breasts or sore nipples: *only when establishing breastfeeding so short-lived* \|; **Allows frequent contact** and assists in bonding \| \| \| **Reduced risk of breast and ovarian cancer** later in life \| \| Could be embarrassing e.g. due to attitude of partner/others \|; **Protection against infection** - Gastro-intestinal infections - ear, chest & tummy bugs \| \| \| **Reduced risk of thin bones** in later life (osteoporosis) \| \| Lack of facilities to feed away from home \|; Reduced risk of developing eczema \| \| \| **Breastfeeding is cheaper** than bottle-feeding even when costs of mother’s diet are taken into account. \| \| May create jealousy amongst partner, relatives or other children because of the exclusive role of the mother \|; Prevents constipation \| \| \| \| \| Incompatible when mother returns to work \|; Reduced risk of baby being overweight or being diabetic as a child \| \| \| \| \| \|; Reduced risk developing heart disease, diabetes or high cholesterol when they are older \| \| \| \| \| \|; Reduced risk of baby being fussy about new foods: baby will receive all flavours from mother’s diet via breast milk & be more accepting of new foods \| \| \| \| \| \|; Reduced risk of sudden infant death (cot death) \| \| \| \| \| \|; **Bottle-feeding** \| \| \| \| \| \|; **Baby** \| \| \| \| **Mother** \| \|; **Advantages** \| **Disadvantages** \| \| \| **Advantages** \| \| **Disadvantages**; Others can also feed Allows others to have contact and bonding with baby \| Baby had 1 flavour (formula) so may be less accepting of new flavours/foods during weaning \| \| \| Not dependent on mother alone – others can share the task of feeding \| \| Time-consuming to prepare feeds; \| \| \| \| Maybe less tiring for the mother \| \| Need equipment – sterilising; bottles, teats etc…; \| \| \| \| \| \| Cost of formula milk and equipment ACTIVITY: CONCERNS AROUND INFANT FEEDING Practitioner facilitates a group discussion using the concerns which were raised in week 1.Refer to the post-its from week 1 and any additional concerns below with the aim of generating solutions from the group from information they already have. Possible solutions are in italics below but there may be others. **Small breasts mean that mothers do not produce sufficient milk** - Size doesn’t matter! The more frequently mothers feed, the more milk is produced. Therefore more is more! - Do not give you baby other food or drink as this will reduce your milk supply. **How do you know that your baby is getting enough milk?** - Your baby will appear content and satisfied aftermost feeds; - S/he should be healthy and gaining weight after the first two weeks; - If breastfeeding your breasts and nipples should not be sore; - After the first few days, your baby should have at least six wet nappies a day; - From Day 4 s/he should also pass at least two yellow stools every day, for the first few weeks. **How will I know that the baby has finished his feed?** - If breastfeeding your baby finishes the feed and comes off the breast on his/her own; - If bottle-feeding you baby will stop feeding and will not take the teat if re-introduced; - Babies will take as much feed as they need and all babies have an in-built system to recognise that they have had enough food and will stop feeding. It is important for parents to recognise and respect this. However If parents encourage the baby to ‘finish the bottle’ or have more breast milk then the baby will ‘unlearn’ this important skill of recognising when they have had enough to eat/drink which in the future may lead them to over-eating and result in becoming overweight. **Remember: “Parents should provide and Babies should decide”** **How often will the baby feed?** - Very young babies feed little and often; - Usually infants settle down to feeds every 3 - 4 hours during the daytime with the occasional drink of water during the night. However over-consumption can occur if a baby regularly awakens during the night and is given milk feeds. This habit becomes a vicious circle and results in excess milk intake leading to weight gain; therefore water should be recommended instead of milk. Often parents believe that their infant does not like water and this should be explored. Parents should be made aware that infants have an innate preference for sweet foods such as milk, and therefore water may initially be rejected, however offering it repeatedly will increase acceptance. **Breast feeding makes big demands on the mother’s time – day and night** - Ensure family support; - Partners/family members can help with other children and household chores; - Ensure adequate rest for mothers. **Physical discomfort from engorged breasts or sore nipples** - Only when establishing breastfeeding therefore short-lived; - This is normal in early days; - Encourage to persevere. **Embarrassment** - Talk to other breastfeeding mothers; - Wear clothing that helps with being discreet; - Discuss with your midwife about Baby Friendly Initiative and support for breast feeding and facilities to feed away from home. **Breast feeding can cause jealousy** - Involve everyone in all other aspects of caring for the baby. **Incompatibility with work** - Partial breastfeeding (e.g. mornings and evenings is possible) and should be encouraged. **How long should I breast or bottle feed for?** Current guidance on infant feeding advises “***exclusive breastfeeding is nutritionally adequate for the first 6 months of an infant’s life”.*** (WHO 2001) Following this recommendation, the Department of Health (DH 2004) reviewed its guidance on the introduction of solid food which is summarised below:- - Breastfeeding is the best form of nutrition for infants; - Exclusive breastfeeding is recommended for the first six months (26 weeks) of an infant’s life; - Six months is the recommended age for the introduction of solid foods for infants whether breast, bottle or breast and bottle-fed; - Breastfeeding (and/or breast milk substitutes, if used) should continue beyond the first six months, along with appropriate types and amounts of solid foods. However it should be noted that all infants should be managed individually so that insufficient growth or other adverse outcomes are not ignored and appropriate interventions are provided. **Note for group leaders:** Remember you are not the experts so signpost accordingly and share useful resources. **Sources of help and resources**: The midwife should be contacted regarding advice about feeding your baby. [www.breastfeeding.nhs.uk](http://www.breastfeeding.nhs.uk/) (NHS choices website –info for parents on breastfeeding benefits, how to, support etc [www.babyfriendly.org.uk](http://www.babyfriendly.org.uk/) On Bradford Every Baby matters website: <http://www.bradford.gov.uk/bmdc/bdp/our_work/every_baby_matters/breastfeeding> Give out the following booklets and ask parents to read them at home to remind themselves of the key messages and help to make their choices about how to feed their baby. - **Every Baby Matters leaflet**: this leaflet contains locally agreed key messages to share and pass on including on breastfeeding. - **Off to the best start booklet**: important information about feeding your baby YOUR BABY NEEDS TO BE TOUCHED AND HELD Babies respond to touch at birth and remain especially sensitive during the first few days. Your baby has been contained safely in your womb for 9 months and will want to have that secure feeling recreated. You can do this by holding them. A father sometimes takes longer to 'warm' to his baby than a mother. It is important that he also has the chance to become physically close to his baby so he can form an attachment. Your baby receives many messages from the outside world through the skin, for example warmth, human touch, comfort, smell. **Mention** that during the 1900's, babies who went to care homes straight after they were born frequently died from lack of physical contact with their mothers and modern research on attachment in relation to maltreatment, foster care and post-institutionalised adoption produces results to concern (Zenah *et al* 2004) **Evidence**: that it is important to be held and touched. Testers put a group of new born monkeys into cages -one cage had a wire surrogate monkey that gave milk and one cage had a soft surrogate monkey that gave no milk. Given the choice, all the baby monkeys went to the soft monkey, even when it did not give milk. This was seen most when the baby monkeys were frightened. (Stoualt, McCiough & Dozier, 2004) | | **15 mins**  **5 mins** | | | |
|  |  | | | | |  |

| **9** | | **RELAXATION**  **Optional** if time. Give as a handout.  **Talking To My Baby**  **Ask** parents to feel as if they are communicating with their baby as you read. Begin by having parents assume a comfortable position, close their eyes (or lower them), dim the lights and take a few slow relaxing breaths.  Hello little baby, how are you? Are you nice and warm inside where you are? You started growing before I even knew you were there. You surprised everyone! Every day you are growing a little bit bigger and when you are big enough and ready, you will be born. I can hardly wait! Today we talked about healthy eating. I hope you like all the foods you're eating. I know sometimes I don't always eat the right things for you to grow. But today I am making a promise to you that I will eat all the right foods you need to grow healthy and strong. I am looking forward to seeing you and holding you and being able to call you by name. Be cozy and safe until then, my sweet little one.  **Encourage** parents to communicate with their baby through expectation stories whenever they have time and their thoughts drift to their baby. Instead of just thinking about the baby, they can talk to the baby about their hopes, thoughts and feeling. | | |  | | |  | | |
| --- | --- | --- | --- | --- | --- | --- | --- | --- | --- | --- |
| **10** | | | **TIME TO HAVE A GO**   - Give yourself 2 positive ‘self’ thoughts every day. - Reflect on the positive ways that you will care for your baby. - Read some information/watch ‘A Gift for Life’ DVD: section on the benefits of breastfeeding. - Talking To My Baby- A Relaxation **hand-out 🕮.** - Do your chosen physical activity, you can write about what you did and how you felt about it in your memory book if you like. - Plan to cook a healthy meal and tell everyone how it worked next week. - Have a think about how you are getting on with healthy eating and activity; where am I now and what can I change? Select 2-3 changes to try and make. Use your diet and activity diary and Healthy Eating Choices **hand-out 🕮.** - You can use Weekly Goal Grid in Tommy’s ‘Managing your Weight in Pregnancy’ booklet if this has been given. | | |  | | | |  |
| **11** | | | | **FEEDBACK: What did you think of today?**  **Give out** feedback forms and pass mints, as in previous weeks, and thank the group for completing them. Place a container on the floor or collect the forms as they are completed. | | | **5 mins** | | | |
| **12** | | | | **GOODBYE CIRCLE: Shaking hands or Thumbs-up**  **Stand** in a circle and say how much you’ve enjoyed the session. Say goodbye by shaking hands around the circle. If the group is very shy, give a thumbs-up around the circle instead. Invite each in turn to say "one thing which was new, interesting or struck me this week was... "  OPTIONAL. **Pass round** a basket with the key message magnet "Praise is Magic" for each parent. **Close** the session with an appreciative comment about the group. | | | **5 mins** | | | |
|  | | | |  | | |  | | | |
|  |  | | | | | | | |  | |

**WELCOME TO THE WORLD**

**HAPPY Antenatal Programme**

**Week Four**

**BOUNDARIES, BELIEFS AND VALUES**

**STEP BY STEP**

PLAN FOR TODAY

|  | **ACTIVITY** | **TIME** | **MATERIALS NEEDED** |
| --- | --- | --- | --- |
| 1 | Welcome | 10 mins | Refreshments, music & player, ‘Welcome’ sign, attractive table cloth, flowers, badges, stickers, felt-tip pens, plan for today, attendance sheet, reward system, etc. |
| 2 | Icebreaker: The Power of Positive Words | 5mins |  |
|  |  |  |  |
| 3 | Feedback | 10 mins |  |
|  |  |  |  |
| 4 | Physical Activity Feedback | 10 mins | Flipchart |
|  |  |  |  |
| 5 | Babies and Boundaries | 30 mins | The Social Baby book x 2  The Social Baby DVD & Player  Board – Values and Beliefs  Hand-out |
|  |  |  |  |
|  | *TEA & COFFEE BREAK* | *10mins* | *Refreshments* |
|  |  |  |  |
| 6 | Helping Baby Calm Down | 15 mins | Flipchart  Handout  Board – Soothing Techniques |
| 7 | Relaxation | 5 mins |  |
|  |  |  |  |
| 8 | Nurturing Ourselves | 40 mins | Board – The Nurturing Wheel  Dice  Jug & glasses  Hand-out |
|  |  |  |  |
| 9 | Time To Have A Go | 5 mins | Time To Have A Go hand-outs |
| 10 | What did you think of today? | 5 mins | Feedback forms, pens, basket/ container, mints, relaxing music |
|  |  |  |  |
| 11 | Goodbye Circle | 5 mins | Candles |
|  |  | 150 mins |  |

**WELCOME TO THE WORLD**

**Family Links Antenatal Nurturing Programme**

**Week Four**

**BOUNDARIES, BELIEFS AND VALUES**

**STEP BY STEP**

| **1** | **WELCOME** | **10 mins** |
| --- | --- | --- |
|  | **Greet** group members warmly as they arrive, have background music playing, offer refreshments and name badges.  When they are all settled, **go through** the topics for the session briefly.  **Introduce** a reward system for the session.  **Display** the Group Rules. |  |
|  |  |  |
| **2** | **ICEBREAKER: THE POWER OF POSITIVE WORDS** | **5mins** |
|  | The object of the game is to apply a positive adjective to themselves. **Invite** everyone to think of a positive word that starts with the same letter as their name, e.g. “Adorable Annette”, “Kind Kathy”, “Terrific Teresa”, etc.  **Go round** the circle and if anyone gets stuck let the group help out.  **Begin playing** the game as soon as possible, and encourage parents to join in as they arrive. |  |

| **3** | **FEEDBACK** | **10 mins** |
| --- | --- | --- |
|  | Comment briefly on last week’s feedback forms. Display last week’s ‘Time to Have a Go’ suggestions and invite brief feedback:  TIME TO HAVE A GO   - Give yourself 2 positive ‘self’ thoughts every day - Reflect on the positive ways that you will care for your baby - Read some information/watch ‘A Gift for Life’ DVD section on the benefits of breastfeeding. - Talking To My Baby- A Relaxation **hand-out 🕮** - Do your chosen physical activity, you can write about what you did and how you felt about it in your memory book if you like. - Plan to cook a healthy meal and tell everyone how it worked next week. - Have a think about how you are getting on with healthy eating and activity; where am I now and what can I change? Select 2-3 changes to try and make. Use your diet and activity diary and Healthy Eating Choices **hand-out 🕮** - You can use Weekly Goal Grid in Tommy’s ‘Managing your Weight in Pregnancy’ booklet if this has been given. |  |
|  |  |  |
| **4** | **PHYSICAL ACTIVITY FEEDBACK** |  |
|  | From last week’s ‘Time To Have a Go’ sheet, how did you get on with the following?   - *Do your chosen physical activity?* - *You can write about what you did and how you felt about it in your memory book if you like*   Ask the group to recall the activity that they chose for themselves last week. In pairs discuss what activity they did or didn’t do and what helped them or stopped them from doing their activity. Explain that it is not good or bad to have done or not done their activity, ask them just to notice why they did or did not do it.  Give prompts: did you think about doing your activity? Did something get in the way? Did you do your activity with your partner or a friend?  **Flip chart**:   - In the group ask people to share the barriers that came up for them around doing their activity, what stopped them from doing it. Write them up on the flip chart. - Then ask the group to think of ways they could overcome that barrier in the future and write it up on the flip chart. |  |
| **5** | **BABIES AND CHILDREN NEED SECURITY, CONSISTENCY AND BOUNDARIES** | **30 mins** |
|  | KEY MESSAGE: Babies enter the world with a readiness to engage socially with those around them. They soon learn about the individual characteristics of their family members and their distinctive ways of relating, and the baby develops ways of relating back to them. As attachment develops, strong emotions to do with dependency and security are established. So a baby and his mother may have different attachment patterns to the baby and his father or other close carers. If the care is consistent and reliable and sensitive to the baby's developing needs, then he is likely to feel secure in the relationship.  With at least two copies of “The Social Baby” Lynne Murray and Liz Andrew, each facilitator displays “The Baby's Response to Her Mother and a Stranger”, page 42, to the two groups and talks them through the pictures.  Then show the clip from the Social Baby DVD of a father sticking his tongue out at a new-born baby and how after a while the baby protrudes his own tongue in response  CONSISTENCY AND BOUNDARIES |  |
|  | KEY MESSAGE: Children need fair, firm and consistent boundaries/rules. Inconsistency leads to confusion: babies and children do not know what is expected of them, or what to expect of adults; confusion leads to a sense of insecurity and often to difficult behaviour.  **Example** of consistency  Parents agree on a time for bed and a routine leading up to it and the baby is always put to sleep in cot.  **Example** of inconsistency  Parents vary time for bed depending on their social life and sometimes the baby goes to sleep in their arms in living room and sometimes in cot.  **Invite** comments: how might a baby feel and behave? |  |
|  |  |  |
|  | EXERCISE ON BOUNDARIES  **Refer** to the cartoons on Types of Boundaries (*PP45*), and **invite** the group, **with their partner or if alone with a facilitator or another lone parent**, to discuss it together, thinking about these questions: Which types of boundary did you experience as a child - at home, with other relatives, at school? Was it difficult to work out what was OK if one parent's boundaries were different from the others, or if you spent a lot of time with a grandparent? Which types of boundaries do you think your children are or might experience? |  |
|  | **Invite** feedback and a brief general discussion about their experiences.  **Display** on Flipchart/Board and invite couples and lone parents in pairs to discuss the following for 10 minutes  **Values and Beliefs About Bringing Up Children**  Ask each other these questions and see how much you agree:   1. Should you go immediately to a crying baby? 2. Is breast feeding best for babies? 3. Should you give your baby a dummy? 4. Should your baby sleep in your bedroom? 5. How do you deal with sleepless nights? 6. How would you deal with naughty behaviour? 7. Should you ever smack your baby or child? 8. Are there particular do's and don'ts on your cultural or religious beliefs that are important to keep?   **Invite** discussion.  **Your Questions Answered**  **1. Should you go immediately to a crying baby?**  It isn't possible to spoil new babies - they only cry when they need something, whether it's food, a cuddle or your company.  **2. Is breast feeding best for babies?**  Yes- breast milk is the perfect food for newborn babies, and the close cuddles and eye contact while feeding are enjoyable for mothers and babies. If you do decide to bottle feed, make sure you provide the same close cuddles as you would when breastfeeding.  **3. Should you give your baby a dummy?**  It's up to you - provided the dummy is kept clean and sterilised. On the plus side, sucking is a great comfort and many babies use their own thumbs or fingers once they get the hang of it. On the downside, you cut down opportunities for babbling conversation later on and you may not like the look of a dummy. Opinions are divided whether long term use can affect teeth but you certainly don't have to worry about that for a very young baby.  **4. Should your baby sleep in your bedroom**?  Many parents find it easier in the early weeks when night feeds are frequent - do what feels right for you- it is up to you. Babies can move in to a room of their own when you feel the time is right.  **5. How do you deal with sleepless nights?**  In the early days it is probably best to try and rest when your baby is asleep. Babies don't know night from day for the first few weeks, and this will prevent you from becoming too exhausted. Now is the time to take up any offers of help to let you catch up on sleep.  **6. How would you deal with naughty behaviour?**  It is quite impossible for babies to be 'naughty' in the sense that they have no idea of the effect of their behaviour on others. They never cry or fuss to get at you. Even when babies become toddlers, they may behave in ways that can be maddening for adults, but this is almost always a normal part of their development - they simply can't help themselves. For ideas on positive discipline, see The Parenting Puzzle.  **7. Should you ever smack your baby or child?**  There is lots of evidence now that smacking is a very negative way to discipline children. It doesn't work, it leads to harder smacks, and to children feeling resentful. They may also copy the behaviour, and be aggressive with other children. Smacking or shaking babies could be dangerous and is totally unacceptable. For alternatives to smacking see The Parenting Puzzle, Session 6, on kinds of touch (PP.133-143)  **8. Are there particular do’s and don'ts in your cultural or religious beliefs that are important to keep?**  It is important to discuss these with your partner to make sure you both agree.  **If you find you have different opinions, now's the time to reach a compromise - not while the baby is crying for your attention. It will be much easier looking after your baby if you have a clear, shared view of how to go about it and if you agree on who you both trust for help and advice.**  Give a copy of ‘Your Questions Answered’ **hand-out 🕮** to every parent for them to take away and suggest the group follows up any queries in feedback next week.  Draw the session to a close by saying how important gentle, nurturing touch is in communicating love and security. It helps babies learn to relax and also helps them to recover after distress.  This is something which all family members can join in with and is a good way for fathers to bond with their baby as well as helping to soothe them.  Another way to offer skin to skin contact is through baby massage, since massage is a wonderful way to help babies get rid of stress and relax.  Note to facilitator: offer information about this now, but more details can be given during the postnatal programme. |  |
|  | **Tea and Coffee Break** | **15mins** |
|  |  |  |

| **6** | **HELPING THE BABY CALM HIM/HERSELF** KEY MESSAGE: Parents need to be alert to the situations likely to cause distress and respond quickly when a baby gets distressed. It is also important to notice whether the baby is able to calm him/herself. EXERCISE ON SOOTHING TECHNIQUES: Introduce the exercise by pointing out that some babies are more sensitive than others and what works in calming one baby may over stimulate and distress another. Early days are different to later. **Display** on Flipchart the following heading: **Ways To Help Babies Soothe Themselves** Divide into small groups using fun divides. Give out a felt tip pen and Post-It notes to each group. Ask them to spend a few minutes writing one example per sheet on causes of distress in babies and ideas for soothing babies. Divide the flipchart sheet into two columns with these headings: Soothing Techniques (Green) \| Causes of Distress (Red); Invite parents for put Post Its under these two headings, e.g.: \|; Calm, familiar voice \| Frustrated, cross, angry tone of voice; Lullaby \| Intense noise; Vacuum cleaner \|; Rocking (for some babies) \| Rough handling (shaking, smacking, not OK at all); Quiet, darkened room \| Over handling; No rocking or handling (for some babies) \| Too much stimulation; Cradling, cuddling, rocking, walking, skin to skin contact \| Lack of attention; Predictable daily routine (from 3 months) \| Inconsistent boundaries and routines; - Feeding, changing, bedtime routine \| Too much tickling; - Helps baby tolerate short delays in parental response \|; Massage \|; Offer breast or bottle feed \| Hunger,; Offer cooled boiled water for bottle fed babies \| Thirst; Check for dirty nappy/ nappy rash \| Uncomfortable | **15 mins** |
| --- | --- | --- |
|  | Then Display the "Causes of Distress" on your prepared board and keep "Soothing Techniques" covered. One by one discuss possible techniques to soothe the distress and then reveal what is covered in the "Soothing Techniques" column and compare with the list generated by the group.  As you can see there are lots of different ways of soothing a crying baby. Remember that feeding them does not have to be the first and only way to soothe a crying baby or to show affection.  It is important to recognize the signs that a baby is hungry - recognize Feeding Cues –shoulder wriggling, rapid eye movement, licking lips and rooting.  It is equally important to know when it is full – Responsive Feeding – baby turns away from the breast or bottle.  It is important to remember that babies are born with an in-built mechanism that knows when it is full. As adults we need to respond to this mechanism and not over-ride it. This will last throughout their life and help them to regulate how much food they eat.  **Remember: “Parents should provide and Babies should decide”**  Preventing distress – Refer to the “Crying” handout. |  |
| **7** | **RELAXATION**  BABY MASSAGE IN THE WOMB  If you would like to start practising infant massage, the following exercise is a great way to begin between you and your "inside baby".  **Remember** that your baby's sense of touch and awareness of movement develops early in the pregnancy. Babies experience all kinds of stimulation in the womb. By six months' gestation, your baby is aware of your touch and may feel your hand move over your bump when you stroke or massage it. Your gentle stroking may have a calming, soothing effect on your baby.  Get into a comfortable position. Support partners should be relaxed and participate in the visualization without stroking their pregnant partner's abdomen at this time. Begin by having each pregnant woman place her hands comfortably on her abdomen. Have participants take several slow, breaths allowing their bodies to relax. Continue with the following massage and visualization exercise which the partner can read out at home.  Visualize the shape of your baby's body under your hands. Is your baby resting or active? Alert or asleep? If you like, begin to slowly stroke your abdomen upward from below your belly button to below your breasts using long, broad stokes, or you may choose to make circular strokes.  As you continue gentle, slow stroking, imagine a warm glow coming from your hands which expresses your love for your baby. This warm light reaches your baby within and touches baby's soft head ... back ... shoulders, arms and hands ... little chest and tummy ... and legs and tiny feet. Feel the love you have for your baby radiating through your hands to your baby within as you continue stroking.  If your baby seems active, see and feel your baby relax as you stroke him/her. Imagine that your baby senses your love and feels safe and secure. Continue to relax and massage your baby for a few more minutes ...  Slow the strokes of your massage, but continue to hold your hands on your abdomen. When you are ready, you may open your eyes and return your attention to the group.  Ideally mothers should repeat this exercise frequently as a way to make contact with their baby and communicate love and nurturing. Encourage partners to stroke the mother's abdomen if she is comfortable with this. Parents may choose to talk or sing to their babies during the massage.  The massage can be done through clothing. When done at home without clothing, parents may use lotion or a cold-pressed fruit or vegetable oil, such as coconut or almond oil, to allow the hand to glide smoothly over the abdomen. | **5 mins** |

| **8** | **NURTURING OURSELVES**  KEY MESSAGE: Self-nurturing is very important. We cannot continually nurture other people without giving something back to ourselves. Looking after ourselves is not selfish: others benefit from the time we give to ourselves as we cope better when our own needs are being met. We can use the analogy of passenger safety procedures on an aeroplane: adults are advised to put on their own oxygen-masks first before attending to anyone else.  **Introduce** the idea that taking time for ourselves and meeting our own needs is important.  **Demonstrate** the importance of taking good care of ourselves by pouring water from a jug (to represent ourselves) into several cups or glasses (to represent family members, friends, colleagues, pets, etc.).  **Invite** examples of ways we look after others, e.g. make lunch boxes; shop; cook; school run; kind words; listening; walk the dog (and pour more water into the cups until the jug is empty). You can't pour water from an empty jug, and we can't indefinitely meet other people's needs without also meeting our own.  **Ask** the group to think of examples of ways in which they look after themselves, give themselves small treats, and pour water back into jug to symbolize topping ourselves up through nurturing ourselves.  **Explain** that there are six different areas of need: social, physical, intellectual, creative, emotional and spiritual. The needs are easy to remember by taking their initial letters to make the word SPICES - nurturing ourselves spices up our lives.  Draw a nurturing wheel on the flip chart and **brainstorm** possible ways in which we can nurture ourselves, going through each segment at a time. Make sure that small, everyday treats (e.g. bath oil in the bath, a cup of tea in a favourite mug, calling a friend, going for a walk) are included as well as the larger ones(e.g. a holiday, starting an evening class); use PP148 to trigger ideas if necessary.  If the group do not bring up food/drink to nurture themselves, prompt: do you ever use any particular food or drinks as a reward for yourself? E.g. chocolate, biscuits, alcoholic drink etc. When we feel like a treat it is often easiest to reach for these foods but we sometimes eat too much and do not always feel better in ourselves afterwards.  Are there other things you could choose or do instead?  Ask the group to look at the ‘Smart Snacks’ **hand-out 🕮** - is their usual treat/snack on there? What swap does the hand-out suggest? Discuss whether they would make the swap or choose another swap from the list.  Remind the group that it is advisable to avoid alcohol consumption during pregnancy and especially during the first 3 months of pregnancy. If you do drink alcohol, limit intake to 1-2 units once or twice a week. What treat could you use instead of alcohol next time?.  **Play** the Nurturing Wheel game.  **Display** the Nurturing Wheel (see opposite, page 6.10). Group members take turns to throw the dice and say how they do, or would like to, nurture themselves – depending on which segment is selected.  **Allow** time for discussion and for any discomfort about being too busy, feeling selfish, etc. to emerge. **Encourage** every member of the group to choose one thing they will try to do for themselves, in the coming week (however small) – and to tell the rest of the group what it is. | **40 mins**  **25 mins** |
| --- | --- | --- |
|  | **Nurturing Physical Activity and Reward**  Discuss the importance of nurturing ourselves by using physical activity as a way to boost our energy, relax and unwind and look after our bodies. Ask the group to add some nurturing physical activities onto their flip chart nurturing wheel. Refer to the ‘Physical Activity Ball for Pregnancy’ **hand-out 🕮** for examples of nurturing physical activity. Tell the group you would like them to focus of the ‘P’ of SPICE. Ask them to choose a nurturing physical activity to do, this might be going for a walk, yoga, dancing, swimming, strengthening exercises (encourage them to do at least 15 minutes).  With a partner (their husband/partner or accompanying person if they are there) ask the group to make an “if then” plan. This involves choosing a nurturing physical activity that they would like to do, how long they will do it for, and a time that they will be able to do it. The IF is the condition, i.e. the day or the time. The THEN is what you will do: walking, yoga, dancing.  Suggest that if they would like, they could make their “if then” plan together. Write a couple of examples up on the flip chart.  For example:  **If** it is Sunday afternoon **then** I will walk for 15 minutes in the park with Joshua  **If** it is after 7pm on Thursday evening **then** I will go to the yoga class in the community centre.  If…………………………………. then………………………………….  Walk around the group as they are talking and ensure that everyone has an achievable nurturing physical activity.  Ask the group to discuss some of the barriers that might come up for them around doing their activity and how they can overcome them– refer back to the flip chart created during the feedback at the beginning of the sessions.  Encourage them to write down their “if then” plan along with how they will overcome their barriers in their memory book.  Ask the group to discuss with their partner what reward they would like to give themselves for completing their activity – suggest something low cost, e.g. a soak in the bath, time to do my nails. Again, encourage them to write it in their memory book.  Suggest that if they would like extra support to do their nurturing physical activity they can sign and date their “if then” plan and their reward, making a commitment to themselves. | **15 mins** |
|  |  |  |
| **9** | **TIME TO HAVE A GO**   - Read “Values and Beliefs About Bringing Up Children: Your Questions Answered” - Try the Relaxation on Baby Massage in the Womb - Think about touch; your experience of it and what kind of gentle/nurturing touch you like to give and receive - Nurture yourself in a new way - Complete your nurturing physical activity (the P in SPICE) - Keep filling in your Memory Book | **5 mins** |
| **10** | **FEEDBACK: What did you think of today?**  **Give out** feedback forms and mints, as in previous weeks, and thank the group for completing them. | **5 mins** |
|  |  |  |
| **11** | **GOODBYE CIRCLE**  **Stand** in a circle, and say how much you’ve enjoyed the session. Pass a basket with a candle for each parent. Invite them to take one and say “One way I plan to nurture myself this week is…”  Close the session with an appreciative comment about the group. | **5 mins** |

**WELCOME TO THE WORLD**

**HAPPY Antenatal Programme**

**Week Five**

**FEELINGS and HOW WE COMMUNICATE**

**STEP BY STEP**

PLAN FOR TODAY

|  | **ACTIVITY** | **TIME** | **MATERIALS NEEDED** |
| --- | --- | --- | --- |
| **1** | Welcome | 5 mins | Refreshments, music & player, ‘Welcome’ sign, attractive table cloth, flowers, badges, stickers, felt-tip pens, plan for today, attendance sheet, reward system, etc. |
| **2** | Icebreaker: Right Now I am Feeling | 5 mins | Flipchart |
| **3** | The Mood Thermometer | 5 mins | Red, orange, green slips & container |
| **4** | Feedback | 10 mins |  |
| **5** | Physical Activity Feedback | 5 mins |  |
| **6** | Feelings and what we do with them   - What we pay attention to - Feelings brainstorm - Suppressing feelings - Managing anger | 40 mins | Flipchart, pens  Cut out Statements  I statement hand-out  Small pieces of paper  Dealing with stress hand-out |
|  |  |  |  |
|  | *TEA & COFFEE BREAK* | *10 mins* | *Refreshments* |
|  |  |  |  |
| **7** | Communication for Adults and Babies   - The art of listening - What does a newborn see, hear and feel - Small changes, big difference | 50 mins | Handouts   - Making eye contact with my baby - What I can do to help my baby and me talk to each other |
| **8** | Time to Have a Go | 10 mins | Hand-outs  Book – “The Social Baby” by Lynne Murray & Liz Andrews |
| **9** | What did you think of today? (Feedback) | 5 mins | Feedback forms, mints, pens, basket/container, relaxing music |
|  |  |  |  |
| **10** | Goodbye Circle | 5 mins | Basket, Magnets “Have I nurtured myself today?” |
|  |  | 150 mins |  |

**WELCOME TO THE WORLD**

**HAPPY Antenatal Programme**

**Week Five**

**STEP BY STEP**

| **1** | **WELCOME** | **5 mins** |
| --- | --- | --- |
|  | **Greet** group members warmly as they arrive, have background music playing,  offer refreshments and name badges.  When they are all settled, **go through** the topics for the session briefly.  **Display** the Group Rules and the Four Constructs charts. |  |
|  |  |  |
| **2** | **ICEBREAKER: RIGHT NOW I AM FEELING** | **5 mins** |
|  | Display on the flipchart: “Right now I am feeling….”  “I cope best when I am feeling….”  **Explain** that the main focus of this session will be on our feelings and our baby’s feelings and how this triggers behaviour and how we can intercept and deal with difficult feelings. |  |
|  |  |  |
| **3** | **INTRODUCE the Mood Thermometer** | **5 mins** |
|  | **Display** the Mood Thermometer board. **Explain** it is important to be aware of our feelings and how they connect with our behaviour, so we will begin by taking our emotional temperature. **Invite** parents to take a small slip of coloured paper (red, orange or green) to indicate their mood. **Notice** the general mood of the group; if there are a few red/orange slips **ask** whether they would like an ENERGISER/CALMER to shift the mood towards green (see new PGL Handbook Mood-shifters in Appendix 1, pages A1-3). |  |
|  |  |  |
|  |  |  |
| **4** | **FEEDBACK** | **10 mins** |
|  | **Comment** briefly on last week’s feedback forms. **Display** last week’s ‘Time to Have a Go’ suggestions and invite brief feedback:  ***TIME TO HAVE A GO***   - Read ‘Values and Beliefs About Bringing Up Children: Your Questions Answered’. - Try the Relaxation on Baby Massage in the Womb. - Think about touch; your experience of it and what kind of gentle/nurturing touch you like to give and receive. - Nurture yourself in a new way. - Complete your nurturing physical activity (the P in SPICE). - Keep filling in your Memory Book. |  |
| **5** | **Physical Activity Feedback** | **5 mins** |
|  | From last week’s ‘Time to Have a Go’ activity how did you get on with the following?  *Complete your nurturing physical activity (the P in SPICE)*  Ask the members of the group to find a partner, and discuss whether or not they did their nurturing physical activity. The discussion should be around their feelings…  How did they feel before, during and afterwards – what affect did it have on their mood? Suggest that they are more likely to feel energised and positive after doing the physical activity they set for themselves, and less positive if they did not do their physical activity. Did they reward themselves for doing their activity? How? Did their reward affect what they did and how they felt? Would they do the activity again?  Ask each person to give a piece of praise and positive feedback to their partner about their nurturing physical activity. |  |
| **6** | **FEELINGS AND WHAT WE DO WITH THEM : ALL BEHAVIOUR HAS A REASON**  **Write** on two different coloured cards in black felt tip ‘All behaviour has a reason: it is being driven by a feeling’. Cut each card into 5 pieces and distribute to each person. If they receive more than one piece ensure it is the same colour.  **Explain** that those with the same colour cards should form a group and there will be a race to put the statement together.  KEY MESSAGE: Pregnancy produces a wide range of emotions from feeling excited to scared.  “Can I cope? How will life change? Will I be a good enough mother? Will my baby be OK?”  It can be normal to feel down during pregnancy (roughly one in ten mothers do some of the time). For others you may never have felt happier.  Dad can feel low as well sometimes, stirring up thoughts like "What am I getting into?" and if your own childhood wasn't great you might be worried about repeating history.  We are emotional beings. All feelings are valid -they contain important information about comfort and discomfort, safety and danger, trust and hurt. No feelings are intrinsically bad (though many people are made to feel that they are). Everyone needs to be helped to identify and understand their feelings, to find ways of expressing them honestly and healthily and to respect the feelings of others. | **5 mins** |
|  | BRAINSTORM on Feelings  **Invite** the group, in **small groups**, to have a brainstorm-race - listing as many feelings as they can in two minutes, writing them all down (uncategorised). Then **ask** them to circle (using a red pen) any feelings that met with disapproval or got them into trouble when they were children.  **Discuss** what messages we received as children about feelings and about expressing them. **Encourage** the group to think about the effects of having to hide their feelings, and whether they might/are doing the same with their children. Explore briefly why adults deny children's feelings e.g. want children to be happy; unhappy; difficult child reflects on them; don't know how to help child deal with difficult feelings because they don't know themselves; challenging feelings erupt into difficult behaviour etc. | **5 mins** |
|  | **Display** the What We Do With Difficult Feelings cartoons chart (see page New PGL Handbook 5.6), without the captions and with the feelings indicated by swirls coloured red. **Refer to** *The Parenting Puzzle* (PP112,113). **Go through** these eight ways of handling feelings briefly, encouraging the group to guess what is going on in each cartoon. **Invite** the group to say which ones feel familiar, in themselves or in other people they know, and allow a few minutes for discussion.  **Introduce** the idea that five of these - suppressing, bottling up, withdrawing, dumping and acting out- are the less healthy ways we deal with troublesome feelings. Reflecting on our feelings, expressing them, and letting go of them when we have learned what we need to from them, are all healthier - for us and for our relationships with others. **Suggest** that it is worth looking in more detail at what happens when we suppress our feelings, and at ways of expressing rather than acting out angry feelings. We will end with a relaxation exercise that helps to release feelings. See page 5.11. |  |
|  | EXERCISE ON SUPPRESSING FEELINGS  NB This is a powerful self-awareness exercise so it is important to encourage parents to take part but remember to **mention** their right to 'pass'. It aims to help troubled parents understand how suppressed feelings can undermine quality of life. It reinforces the idea that they have choice about how to meet their own needs.  **Reassure** the group before they start that this exercise is fun to do and what they draw or write will remain entirely private: nobody else will see their slips and they won't be asked to talk about what they have chosen.  **Give** each person four small pieces of paper. **Ask** them to draw a sketch or symbol (or write a word or phrase) on each one to represent a feeling they find difficult to cope with or an unresolved issue in their lives. They can choose four different feelings and/or issues, or one feeling four times, or two feelings twice, etc. Remember to refer to the slips of paper as FEELINGS. **Proceed** as follows:   1. When they are ready, ask them to fold each piece of paper once or twice (to the size of a postage stamp) to hide the feeling. 2. Invite them to stand up and walk around. Urge everyone to get on with their lives - going to work, doing the shopping, collecting children from school, taking the dog for a walk, cooking, meeting friends, etc. 3. Ask them to place two pieces of paper under their shoes, one under the toe on each foot, and to carry on with their busy day (and be sure to keep the 'feelings' hidden). After a few moments, ask them to put the remaining two pieces under the heels of their shoes and again to move through the day (without showing their 'feelings'). 4. 'Freeze' the group and encourage them to notice how others look - what does their posture seem to say about them? 5. Invite the group to remove two of the four folded slips to symbolise recognising/dealing with those feelings or issues, and again to get on with their lives. Pause after a few more moments to notice whether it is any easier to move around. 6. Remove the other two slips, and walk around in freedom again. | **5 mins** |
|  | **Discuss** together how slowed down, bowed over, sluggish you all looked, how almost all your attention and energy went on suppressing the feelings and keeping them out of sight. This is what depression is like.  **Encourage** the group to collect their pieces of paper and to treat them with respect - remember they are not 'bad' feelings, though they may be very uncomfortable. It is up to each parent to decide whether to throw them away or keep them to reflect on or dispose of at home.  **Reflect** that if we have something difficult to communicate we may concentrate on the words and overlook what we communicate through body language and tone of voice. Others pick up our moods more easily than we think, so words, tone of voice and body language must match so we don't confuse babies and children. Adults can be dishonest in trying to protect children, e.g. if a child sees you weeping it is more important to say you are sad (and perhaps why) than to say “Oh, I'm just tired”. |  |
|  | MANAGING ANGER KEY MESSAGE: Research shows that if babies and children live in "angry" households it can have a negative impact on their mental health and behaviour. Perhaps you are feeling anxious, even angry about being a parent too soon? Perhaps everyday problems trigger anger? Anger is a normal feeling but it isn't good for you, your baby or your family's health. **Invite** ideas on how to release angry feelings such as: - Talking things over before getting angry - Listening to music to de-stress - Walk fast; swim; dig the garden; kick a ball - Try a massage - Practice pregnancy relaxation and breathing exercises to help you feel calm. Good preparation for labour DEALING WITH STRESS **Reflect** that everyone gets anxious from time to time but recent research has shown that extreme anxiety can cut down on the amount of blood going to the uterus. This can mean your baby being born earlier or smaller than normal. The effects of stress are also bad for you and for dads too, so it's really important to recognise the signs. You can keep stress to a minimum by avoiding situations that stress you and looking for ways to handle it when it does arise. This will be of enormous benefit when you are faced with the inevitable stresses of a new baby. Distribute the following as a **hand-out 🕮**and invite parents to complete: Tick Yes or No to these questions: \| Yes \| No; 1. Are you looking forward to having a baby? \| \|; 2. Was your baby planned? \| \|; 3. Are you over 19 years old? \| \|; 4. Are you in a happy relationship now? \| \|; 5. Were your parents warm, loving, firm but fair? \| \|; 6. Have you done something nurturing in the last 2 weeks? \| \|; 7. Do you feel life isn’t a struggle money-wide? \| \|; 8. Are you getting enough sleep? \| \|; 9. When things go wrong can you shrug them off? \| \|; 10. (if applicable) Is being a step-parent going well? \| \| | **10 mins** |
|  | If you answered "no" to most questions, there's probably already a lot of stress in your life. Think about asking for support from your partner, family, friends, Children's Centre, GP or midwife.  **Serious Anger**  If anger ever makes you violent it's important to get help. Violence in the family is damaging for everyone. If this is happening in your life you need to ask for help straight away.  Optional Alternative Exercise for Dealing with Stress  Choose which one you think the group would prefer. |  |

|  | | **HANDLING STRESS**  **I want to be a good parent. I want to do what is expected of me.**  Self  Parents    Partner  Children  In-laws  Church  School  Society  Friends    Write on the top of the figure, “I want to be a good parent. I want to do what is expected of me.” State, “That's where our problems begin - meeting expectations. When we try to meet all these expectations and can't, anger and frustration build up and we may begin to direct our anger at anyone including our babies, our partners and ourselves”.  Write the word “Self” on the side of the figure with an arrow pointing to the figure. Ask the group to share one expectation each has for themselves as a parent. (Example: I will never feel anger toward my baby).   - Write the word “Parents” on the side of the figure with an arrow pointing to the figure. Ask the group to share expectations our parents have of us as parents. (Example: To raise our children the way they raised us). - Continue writing all the different sources of expectations. Ask the group to share examples of the expectations each source has on us in our role as parents and parents to be. When the figure is complete, it will look very stressful to everyone. - Say to the group, “It is impossible to meet everyone's expectations. Every day in our role as a parent there is someone who will not get expectations met. If I do what my partner expects, my parents may not be pleased. If I do what my children expect, my partner may not be pleased. So what can I do? The clearer I am about what I want as a parent, the better I will be in performing successfully in my role as a parent. The attention I give to myself to take care of me is a key in stress reduction”.   MANAGING EMOTIONS AND EATING  We have heard how damaging anger and stress can be for our growing baby and also on children as they grow up.  Other emotions can also be difficult to manage and means that we resort to unhealthy ways of managing them.  Many people use food as a means of dealing with emotions such as anxiety, boredom, sadness, anger. These techniques might help:   - Identify the cause of the emotion – remember as we said at the beginning “all behaviour has a reason”. - Complete a food and mood diary to identify any patterns, including where or who you were with as well as what you ate. - Plan ahead and identify some other things you could do to distract you from eating or help you feel better about yourself. These are some suggestions: - Read a newspaper or book. - Buy a magazine. - Go for a walk. - Phone or visit a friend or family member. - Play a board game with the children. - Have a trip to the park with the children. - Listen to music. - Dancing. - Gardening. - Cleaning. - Go for a swim. - Have a relaxing bath. - Start a new hobby. - Cook a meal to freeze for a busy time. - Use the computer/go on the internet. - You could also keep some healthy snacks available for those times when you know you will be tempted to eat unhealthy foods. See the Snack Swap Sheet **hand-out 🕮** for ideas. | | **10 mins** |
| --- | --- | --- | --- | --- |
|  | STRATEGIES FOR REDUCING STRESS:  “I” STATEMENTS AND RELAXATION  Mention that anger is triggered by stress so the group will explore two ways of intercepting difficult feelings that can escalate into anger.  EXERCISE USING ‘I’ STATEMENTS  GIVING AN “I” STATEMENT  **Ask** the group, **in pairs**, to think of a blaming “You” message they tend to use, and to convert it using the practice sheet (see **hand-out 🕮** and *PP123*).  **Invite** anyone who would like to share their alternative to read it out to the group. **Encourage** the idea that parents do not have to reveal their most troubling feelings - they can choose ones that are true but do not make them feel too vulnerable in a given situation. **Check** that the strategy is clear; encourage the group to try it at home.  GIVING AN “I” STATEMENT  I feel……………………………………………………………………………………...  (*say how you feel*)  when……………………………………………………………………………………  (*describe the situation, and avoid using the word “you”*)  …………………………………………………………………………………………….. because……………………………………………………………………………………  (*state your need not being met*)  ……………………………………………………………………………………………….  What I’d like is…………………………………………………………………………….. (*describe the behaviour that would meet your need*)  ……………………………………………………………………………………………...  For example:  I feel ….. disappointed  When ….. I am not supported in my efforts to do some exercise  Because ….. I need some support to become more healthy.  What I would like is …..to get support and encouragement for my efforts.  Or:  I feel ….. upset  When ….. the healthy meal I have cooked is not eaten  Because ….. I have taken time and effort to prepare the food.  What I would like is ….. for all the family to eat healthy food together on some nights of the week. | | **10 mins** | |
|  | RELAXATION HOLIDAY  One way we can reduce stress in ourselves is to find a few moments to relax each day.  **Invite** the group to check that they are sitting comfortably, and to lower their gaze or turn their chairs outwards to avoid seeing each other. Read the following script in a calm, quiet, slow voice, pausing for 5 slow counts for each image to build up in people's minds.  *Sit comfortably, start with a few slow, easy breaths and begin to relax...*  *and now let your mind create these sensations:*  *Imagine the colours of the sky in a beautiful sunset...*  *Imagine the sound of water flowing in a stream...*  *Imagine the smell of a bonfire on a crisp autumn day...*  *Imagine the feel of smooth, clean sheets...*  *Imagine the sound of rain on the roof ..*  *Imagine the smell of a field of grass in the hot summer sun...*  *Imagine the stars and moon in the dark sky on a clear night...*  *Imagine the taste of a favourite food...*  *Imagine the sound of a beautiful song...*  *Imagine the wind blowing on your face...*  *Imagine floating on the still water of a peaceful, shallow lake. ...*  *Notice the feeling of calm and peace, and take time to enjoy it ...*  *When you are ready, remember where you are and come back to us here.*  **Allow** a few moments for the group to refocus, then ask them to wiggle their fingers and toes to reconnect with reality. **Invite** comment. **Refer** to *The Parenting Puzzle (PP145)* or the audio CD Session Six where the Relaxation Holiday is reproduced. **Mention** it is also a great way to help children relax at bedtime; they can use their vivid imaginations to transport themselves based on the five senses.  **Remind** the group of how we explored the baby's 5 senses in Week One around the time of delivery. | | **5 mins** | |
|  | **Tea and Coffee Break** | | **10 mins** | |
|  |  | |  | |
| **7** | **COMMUNICATION FOR ADULTS AND BABIES** | | **40 mins** | |
|  | KEY MESSAGE: Communicating clearly with each other is essential for happy family life. Good quality listening is essential. | |  | |
|  | EXERCISE ON THE ART OF LISTENING FOR ADULTS  KEY MESSAGE: One of the greatest gifts we can give each other is to listen. Being listened to really well, attentively and kindly, encourages trust, honesty and openness between people. It's a great way to improve communication in all our relationships, and particularly in how we relate to our children.  For babies communication often involves non-verbal signals and can be encouraged by facial expressions and eye-contact, see opposite page 3.10. | | **10 mins** | |
|  | **Model** open and closed body language, an inviting and a threatening stance and discuss what we do when we listen well.  **Ask** the group to form pairs. **Explain** that in turn, each partner will talk for about 30 seconds to a minute, while the other person listens, without saying anything. At first, the listener is to be as attentive as possible; at a signal from you, they are to listen less well (glance out of the window or at the clock, fidget, tie a shoelace, fiddle with a ring, etc.). Then at another signal, go back to listening well.  **Invite** everyone to think of an easy topic to talk about (not a major crisis!) before you begin. **Do** the exercise twice, with partners exchanging roles.  **Ask** for feedback from the group, and discuss briefly. **Draw out** their feelings of being ignored, feeling small/unimportant, giving up, etc. **Reflect** on how often we complain that children don't listen to us- are we modelling listening skills to them, or do we usually half-listen while we are doing something else, interrupt and finish their sentences, or talk at them without giving them a chance to express their opinions?  **Suggest** that parents might find a time with children - perhaps at bedtime­ when they will listen to whatever the child would like to tell them, without interrupting, correcting, advising or doing anything other than simply listen. | |  | |
|  |  | |  | |
|  | **HOW BABIES COMMUNICATE**  WHAT DOES A NEWBORN SEE?  KEY MESSAGE: At birth a baby can only focus up to 9-12 inches. The baby can track or follow movement but people and objects look a bit fuzzy. Up to 3 months a baby sees best "out of the corner of their eyes" - peripheral vision. They really notice contrast -light versus dark patterns and movement best. They may cry because they get "visually stuck" on one object, although after one month they learn to shift their gaze.  At 3 months babies like to watch their hands and circular movements, e.g. toys on a mobile.  By 9 months the brain development around sending and receiving visual messages enables the baby's vision to be as clear as an adult's. So plenty of visual stimulation, especially your smiling face, helps this to happen.  EXERCISE  **Display** on the flipchart the following heading:  Making Eye Contact With My Baby  **Invite** the group to discuss what they can do and write on the chart.  After a few minutes, present a board with the following ideas and discuss further.  What I Can Do To Make Good Eye Contact With My Baby   - Hold or place the infant about 9-12 inches from your face; this distance keeps you in the infant's visual field. - Don't worry if her eyes wander independently or if she looks at you “out of the corner of her eye” in the early months, this is normal. - By 2 months the infant especially likes to look at your face if you widen your eyes and move your mouth. - You can move your head slowly so that the infant can follow it from side to side or up and down. - You can hang a mobile within the infant's visual range. - You can encourage an infant to follow an object with her eyes by slowly moving an object, such as a stuffed animal or toy 10-15 inches from the infant's face. - Pay attention to each of the infant's cues. - Remember when looking at infants that they need to take breaks, and may tell you this by looking away. When they do this, be quiet for a moment. - Give the infant time to “take a breather” from activity. - When the infant looks back at you, begin the “conversation” again.   Offer these suggestions as a **hand-out 🕮.** . | | **10 mins** | |

|  | WHAT DOES A NEWBORN HEAR?  KEY MESSAGE: a newborn can hear many sounds and they have been hearing the rhythm and tone of their mother’s voice before birth (and dad’s too if he talks to “the bump”).  **Display** a board with the following and invite group members to read out loud:  What Can I Do To Help My Baby and Me Talk To Each Other?   - Talk to the infant during caregiving such as changing nappies and feeding - Notice how you speak to an infant. At this point the baby is most attracted to the sound, pitch and rhythm of your voice - the music of language. - Softly sing songs and watch for the infant's response. - Listen and enjoy the sounds a newborn makes in the first 2 months. - Pretend that the infant is “telling you” something when she coos and gurgles. Expand on what she “says” and answer her “questions”. Respond to her vocalisations by imitating her sounds. - Remember to pay attention to the infant's cues. If, for example, the infant looks away, she may be telling you a break is needed.   Offer these suggestions as a **hand-out 🕮.** .  Suggest you can make a real difference to your baby's chances in life by talking and singing to your baby before and after he/she is born (both mum and dad) and by sharing activities together. A big vocabulary will give your child a flying start at school later on.  Mention that **your baby can hear before it is born**. If you stimulate a baby's hearing before birth they find it easier to listen as they grow up.  By the 24^th^ week (6^th^ month) of pregnancy, your baby can hear music and voices. Have you noticed what the baby does when you play loud music? Your baby does not like loud noises and will tell you so by kicking or moving.  Your baby will pick up the emotional tone of your voice and other familiar voices. Newborn babies can pick up their mother's and father's voice within an hour of birth.  **Evidence** that babies can hear before they are born: tapes of a human heartbeat were played into a nursery of newborn babies. Those babies who heard the tape did much better those who did not hear the tape (they ate more, weighed more, slept more, breathed better and cried less).  **TOP TIP**: Beware spending more time talking on your mobile than to your baby.  Ask if any in the group have a nursery rhyme or baby game that they can share with the group.  With a doll role-play the following fun game that babies love: Round and Round The Garden  On a doll/soft toy, open the palm of the hand and make circular action with index finger of other hand while saying:  “Round and round the garden  Like a teddy bear  One step, two step, tickly under there.”  As you say “one step” put finger on lower arm; “two step” put finger in the crease of elbow; “three step” tickle the armpit.  Suggest people might like to do this on their tummy at home and register the baby's response. | **10 mins** |
| --- | --- | --- |
|  | LINKING SIGHT, SOUND AND TOUCH  KEY MESSAGE: babies need gentle touching, holding and eye contact, just as they need food to grow and develop. Studies show that a nurturing touch actually helps many babies gain weight and develop healthy relationships with caregivers. Sharing books and stories as part of quiet cuddling time can be a great way for caregivers and infants to connect with each other.  Mention that at birth a baby’s sensitivity to touch is well developed, especially the mouth, palms of hands and bottom of the feet. The brain registers the slightest touch so gently holding your baby, gazing into her eyes, talking and vocalising will all help healthy brain development that promotes a happy, contented child (Hellen, 1987).  Within Session 1 when we talked about the baby’s 5 senses we mentioned the importance of skin-to skin contact, and physical closeness to the baby during feeding and cuddling times.  Refer to the booklet “Off to the Best Start” given out in Session 3  On Pg 6 it describes holding your baby against your skin straight after birth as it will help to calm the baby, steady its breathing and keep it warm.  This is also a great time to start the first breastfeed and help will be available from midwives.  Check with the group if they are aware of this information and if they have any thoughts about it. Will it be possible for them? Will their partner or whoever supports them at the birth encourage this?  Once you are home with the baby, how can you and other family members continue to have skin-to skin contact with your baby?  Consider the role of fathers, older siblings in helping to calm and cuddle the baby whilst building a bond with them, bath times together, infant massage, use of slings and wraps to hold baby close whilst moving around.  **How much holding does an infant need?**  Everybody has different levels of liking to be touched. Look for his/her cues and respond appropriately. Some like to be held a lot and stroked, others can feel “over handled”. If the baby turns away he/she probably needs a rest from being touched.  **Can I spoil a newborn baby by holding him too much?**  You cannot spoil a newborn by holding, touching, caressing, comforting and meeting needs. In fact, the best evidence says that infants who receive a lot of loving attention in these early months become more independent, resourceful, and less demanding toddlers.  **What can you do:**   - Hold, cuddle, hug and rock an infant in your arms - watch for what the baby likes best. - Caress or gently stroke the head and skin. - Bathing, changing nappies and feeding also involve your touch. - Avoid leaving an infant for long periods of time in a swing or infant seat. This keeps the infant away from your loving touch. - Be aware that infants do feel pain, and may cry in response to it. - When infants cry, look for anything that may be hurting them, as well as considering whether they are hungry or wet or bored, before assuming that they are “just fussing for attention”. - Remember to watch for cues about what kind of touch and how much touch an infant likes. - Watch for cues that tell you the infant wants to take a break. | **10 mins** |
|  | **MINGLE ACTIVITY: SMALL CHANGES BIG DIFFERENCE**  We have been talking about the fact that we don’t just communicate through our words we communicate through our actions. As your children grow-up they will copy adult behaviour and so as parents you are/will be role models for your children.  Think about some of the small lifestyle changes that you could make which you would like your child to copy so that they are healthy and happy. They don’t have to be big changes.  Open up for a quick group discussion.  **Prompt**: food swops, changing sitting time for active time, cut down on TV watching, get up from my desk at work at least once an hour. Lifestyle physical activity – climbing stairs, walking to the shop etc. Ask the group to look over the ‘Physical Activity Ball for Pregnancy’ **hand-out 🕮** and ‘Smart Snacks’ **hand-out 🕮** to give themselves some ideas.  LEAD INTO THE MINGLE ACTIVITY.  Ask everyone to stand up and find a partner, decide who will go first. Think of some small lifestyle changes that you could make which you would like your child to copy. Turn to face your partner, one of you will go first and say “one small lifestyle change I **could** make is….” For example: park further away from the supermarket entrance so I walk a little further. Your partner will then share one small lifestyle change they **could** make with you. Make your answers short and quick. Raise your hand and find another partner to share with. Continue sharing with different partners until I ask you to stop. As you go around you can use some of the activities that the other share with you if you like. | **10 mins** |
| **8** | **TIME TO HAVE A GO**  Read the **hand-outs 🕮**:   - What I Can Do To Make Good Eye Contact With My Baby - What Can I Do To Help My Baby and Me Talk To Each Other? - Continue with Memory Book - Try playing “Round and Round the Garden” with your unborn baby - Practice Relaxation or an “I” Statement - Lend or recommend “The Social Baby” by Lynne Murray and Liz Andrews | **10 mins** |
|  |  |  |
| **9** | **FEEDBACK: What did you think of today?**  Give out feedback forms and mints, as in previous weeks, and thank the group for completing them. | **5 mins** |
| **10** | **GOODBYE CIRCLE: Smile goodbye**  **Display** 4 constructs board and mention that today in particular the Self Awareness/Self Esteem building block; Appropriate Expectations and Empathy building blocks have been addressed.  **Seek** group’s agreement as to whether they would like a **hand-out 🕮** next week with each other’s phone numbers and email addresses.  **Stand** in a circle, and say how much you’ve enjoyed the session. Pass round a basket with fridge magnets “Have I nurtured myself today?” and invite them if they wish to say “One way I plan to nurture myself or relax this week is…”  **Close** the session by saying how much you appreciate their honesty and contributions. | **5 mins** |

**WELCOME TO THE WORLD**

**HAPPY Antenatal Programme**

**Week Six**

**BEYOND LABOUR DAY**

PLAN FOR TODAY

|  | **ACTIVITY** | **TIME** | **MATERIALS NEEDED** |  |
| --- | --- | --- | --- | --- |
| **1** | Welcome | 5 mins | Refreshments, music & player, ‘Welcome’ sign, attractive table cloth, flowers, badges, stickers, felt-tip pens, plan for today, attendance sheet, reward system, etc. |  |
| **2** | Icebreaker: One Way I Like To Relax… | 5 mins |  |  |
| **3** | Feedback | 10 mins |  |  |
| **4** | Quick Quiz | 10 mins | Quiz hand-outs |  |
| **5** | Labour Day and Beyond   - Planning ahead - Establishing feeding - Bed-sharing - Avoiding Cot Deaths - Getting back to being physically active | 50 mins | Local Information  Hand-outs with website references  Bed sharing leaflet  SIDS leaflet |  |
|  |  |  |  |  |
|  | *TEA & COFFEE BREAK* | *10 mins* | *Refreshments* |  |
|  |  |  |  |  |
|  | Labour Day and Beyond cont’d   - Spoiling babies - Common Complaints | 20 mins |  |  |
| **6** | Empathy for Adults | 10 mins |  |  |
|  |  |  |  |  |
| **7** | Post Natal Depression | 5 mins |  |  |
| **8** | Special Gifts for My Baby | 10 mins | Small pretty cards |  |
| **9** | Staying in Touch | 5 mins | Small pretty cards |  |
| **10** | What did you think of today? (Feedback) | 5 mins | Feedback forms, mints, pens, basket/container, relaxing music |  |
|  |  |  |  |  |
| **11** | Goodbye Circle: A Wish for the Future  Presentation of Certificates  Web of Friendship | 5 mins | Small boxes for gift cards  Certificates  Ball of String  List of email addresses & phone numbers |  |
|  |  | 150 mins |  |  |

**WELCOME TO THE WORLD**

**HAPPY Antenatal Programme**

**Week Six**

**BEYOND LABOUR DAY**

| **1** | **WELCOME** | **5 mins** |
| --- | --- | --- |
|  | **Greet** group members warmly as they arrive, have background music playing,  offer refreshments and name badges.  When they are all settled, **go through** the topics for the session briefly.  **Display** the Group Rules and the Four Constructs charts. |  |
|  |  |  |
| **2** | **ICEBREAKER: ONE WAY I LIKE TO RELAX IS…** | **5 mins** |
|  |  |  |
| **3** | **FEEDBACK** | **10 mins** |
|  | **Comment** briefly on last week’s feedback forms. Display last week’s ‘Time to Have a Go’ suggestions and invite brief feedback:   - What I Can Do To Make Good Eye Contact With My Baby. - What Can I Do To Help My Baby and Me Talk To Each Other?. - Continue with Memory Book. - Try playing ‘Round and Round the Garden’ with your unborn baby. - Practice Relaxation or an ‘I’ Statement. - Lend or recommend “The Social Baby” by Lynne Murray and Liz Andrews. |  |
| **4** | **QUICK QUIZ – MY BABY’S DEVELOPMENT IN THE WOMB** | **10 mins** |
|  | **Divide** the group in two teams. Explain that as in TV game shows you will ask a question. The teams have 10 seconds to confer and raise a hand to provide the answer. If correct they get one point. If incorrect it goes to the other side. If both sides give incorrect answers then the quizmaster gives the right answer.  When the quiz is over give each parent a **hand-out 🕮** with the answers filled in.   - He/She has the basis of the brain, spiral cord and eyes at weeks. - His/her jaw and mouth are developing and there are 10 tooth buds growing in each jaw at weeks. - He/she is just beginning to have the sucking and grasping reflex at weeks. - He/she is 1 inch (2.5cm) long at weeks; 3¾ inches (8-9cm) long at   weeks; 6¾ inches (16cm) long at weeks; 4-5 pounds (16 inches) at  weeks; 7 pounds (20 inches) at weeks.   - My unborn baby is completely formed at weeks. - My baby is as sensitive to touch as a one year old at weeks. - My baby can see, hear, taste, experience, feel, remember and learn at   weeks.   - My baby reacts differently to voices of mother, father, unknown people at   weeks.   - My baby may be practising slight breathing movements, although there is no air in its lungs. This can cause hiccups at around weeks. - A new born baby can focus best on an object/face that is about away. - A baby under one year can be spoiled by being picked up too much. True/False - A baby's brain development from birth to 4-6 months may cause it to wake every 3 to 4 hours. True/False - A baby who is cuddled; not left to cry for more than 10 minutes; is smiled at and talked to in a warm tone of voice develops a brain that helps him/her be happier, more positive and healthier than a baby who is treated harshly or coldly. True/False - A good way to discipline a child under 2 years is to distract him/her. True/False - A good way to be a confident parent is to know it is important to look after myself as well as the rest of the family. True/False |  |
| **5** | **LABOUR DAY AND BEYOND**  This is our opportunity to discuss some of the issues to consider in planning for the birth of baby and things to be aware of immediately after baby’s birth.  Remind parents that we will be joining together again once babies are about 6 weeks old and that there will be contact between now and then by phone.  This session is focussed on introducing or reminding about a number of key issues.  Remind parents that if specific obstetric concerns arise discuss with individual parents the best antenatal professional for them to approach.  Recommend [www.nhs.uk](http://www.nhs.uk/) and go to Pregnancy/ Planner/Pregnancy and Labour.  You will possibly have given some thought to a birth plan and packing a bag ready for hospital, but how much planning have you done ready for when you get home from hospital.  **BEYOND LABOUR DAY**  **Looking after mum and planning ahead**  ACTIVITY:  Ask the group to work in pairs.  Give out cards with topics on and ask each pair what they could plan in advance to help cope with their topic  Topics – getting enough sleep, cooking, eating – meals and snacks, resting, housework, feeding baby, occupying other children, including physical activity, visitors.  Invite each pair to feed back on their topic and ensure that we hear what partners, parents, family members etc can be doing too.  Refer to the “Coping with the first weeks with your new baby” **hand-out 🕮** for ideas and pass this out to parents to take home.  **Consider showing video from Australian website showing Dad helping out after the birth – includes Russell Crowe talking!! – or if not possible then share the link with parents to watch at home and get some more ideas.**  How dads can help.  <http://raisingchildren.net.au/articles/dads_help_video.html/context/807>  **Establishing Feeding**  Return to the flipchart and post-it notes from Week 1 regarding advantages and disadvantages of different feeding methods.  Ensure you address the following issues, but also consider any other concerns that parents still have which have not already been addressed.  Signpost to the relevant professional if you don’t have the knowledge or expertise to respond.  **1. Choosing to Bottle feed**  If you choose to bottle feed it is important that you know exactly how to make up a baby’s bottle.  Share the resource “Guide to Bottle Feeding” from NHS which gives all the detail needed about making up a bottle safely, how to store it, what to do with unfinished milk etc.  If you choose to give your baby a bottle its really important to be shown how to make it up correctly to ensure each feed is hygienic and safe as well as the correct concentration. Ask your midwife or health visitor to show you. If you aren’t using expressed breast milk only use whey based infant formula in a bottle and never add anything else to the feed (unless on the advice of a health professional). You will need to make each bottle up as you need it and throw away un-drunk milk.  This information also needs to be shared with anyone in the family who may be helping with making up bottles for the baby.    **2. Getting Support to Establish Breastfeeding**  As discussed breastfeeding can be time-consuming, particularly in the early days, but is the most important job you can be doing for your baby.  Practical help is necessary as highlighted on the **hand-out 🕮**, but what other help might you need (emotional support)? Who do you think within your family, friends or networks can help and encourage you to breastfeed?  **3. Building Confidence to feed in Public**  Some parents can feel embarrassed feeding in public or in front of family members.  What things can you do to overcome these fears? Location, clothing, attitude of others?  Ask parents to discuss in pairs/threes the last 2 questions about getting emotional support and confidence in feeding in public.  What suggestions /solutions can they offer?  What will they do differently as a result of this information?  Refer to the materials in Session 3 and other resources for prompts and solutions.  **Remind parents** ‘Don’t be scared to ask for help’: advice about help from midwives, health visitors etc.  Can you provide phone numbers of local sources of support, including any that are available 24 hours a day?  **What about Bed-sharing?**  KEY MESSSAGE: The Department of Health, Foundations for the Study of Infant Death (FSID) and UNICEF Baby Friendly Initiative state that the safest place for a baby to sleep is in a cot by the side of the bed until six months of age.  Divide into 3s and ask the group to think of what particular factors might make it dangerous for a baby to sleep with the parents. Write up ideas on flipchart. The key points to include are:   - Smoking in pregnancy - Smoking any time - Breastfeeding in bed - how to do it safely - Tired parents - Co-sleeping on sofa - Conflict of advice   Mention that bed sharing is especially dangerous if either parent is a smoker, even if they never smoke in bed; or if they have been drinking alcohol, or taking drugs or medication that make them feel sleepy; or if they're just very tired. Bed-sharing is also dangerous if the baby was born premature or low birth weight.  Give fuller explanations as required from the following bullets:   - Smoking in pregnancy increases the baby's basal temperature and increase in temperature is a risk factor therefore a cot is safer. - Smoking at any time raises people’s body temperature so the adult bed would be hotter therefore a cot is the best option. - Even with a non-smoker the risk increases if baby is under three months old. - Best for a nursing mum to finish with breast on the outside of the bed so the baby does not end up in between parents - extra heat with two bodies and duvet. - Some risk of a very tired parent rolling onto a baby. - Co-sleeping with your baby on a sofa is especially dangerous. - Those who promote attachment urge feeding the baby in bed and letting it sleep with the parent. This conflicting advice is hard for parents.   Recommend [www.babyfriendly.org.uk](http://www.babyfriendly.org.uk/) as a good website for more information on bed-sharing.  **Avoiding Cot Deaths**  Ensure that parents are aware of the key advice given about avoiding cot death -  **TOP TIP**: babies are safest sleeping on their backs and playing on their fronts.  Obtain free leaflets to give parents from [www.fsid.org.uk](http://www.fsid.org.uk/) | **5 mins**  **10 mins**  **15 mins**  **10 mins** |
|  | WHEN CAN I GET BACK TO BEING PHYSICALLY ACTIVE?  Give out the HAPPY postnatal physical activity **hand-out 🕮** and split the group into two or four (depending on number of people in the group, each small group should have 3 or 4 people in it. If there are 2 groups ask one to read the front page of the leaflet, “when can I start exercising?” and the other to read “what should I be aware of before exercising?”. If there are 4 groups, 2 will read “when can I start exercising?” and 2 will read “what should I be aware of before exercising?”. Ask each group to discuss their thoughts about exercising after pregnancy, what are their worries, what are they looking forward to doing? Divide the group again into pairs, one partner should have read, “when can I start… and the other “what should I be aware of…” Ask each partner to teach each other about the section that they read.  Give out the physical activity for new mum’s quick quiz and ask the group to complete it. Go over the answers with the group.  Invite the group to practice pelvic floor exercises and deep stomach exercises if they feel comfortable to.  **Pelvic floor exercise**  Your Pelvic floor muscles support and protect your bowels, uterus and bladder. Hormonal changes in pregnancy and the pressure of your baby on the pelvic floor can loosen these muscles.  This exercise will strengthen your pelvic floor muscles. You can do this exercise either sitting or standing.   - To find your pelvic floor muscles imagine you’re stopping your urine flow when you pee, the muscles contracting are your pelvic floor. - Squeeze and draw up the muscles. - Do it quickly, tightening and releasing the muscles immediately. - Then do it slowly, holding the contractions for as long as you can (but not more than 10 seconds) before you relax. - Do five lots of pelvic floor quick squeezes and five lots of slow squeezes five times a day and build up to doing ten squeezes five times a day.   Ask if they are able to locate their pelvic floor muscles and go over the exercise with anyone who is struggling.  **Deep stomach exercise**  This exercise will help to firm your stomach:   - Lie on your side with your knees slightly bent. - Let your tummy sag and breathe in gently. - As you breathe out, gently draw in the lower part of your stomach like a corset, narrowing your waistline. - Squeeze your pelvic floor at the same time. - Hold for a count of 10 and remember to keep breathing, then gently release. Repeat 10 times. | **10 mins** |
|  | **Tea and Coffee Break** | **10 mins** |
|  |  |  |
|  | **Spoiling Babies**  Suggest it is important to recognise that children approximately one year and younger are never considered “spoiled”. Babies under one year require their needs to be met immediately. As they grow older, their needs become less demanding and parents can build in a little waiting time if the need is not urgent.  **Myths and Fact About Spoiling Children**  **Myth:** You will spoil your child by picking him up when he cries.  **Fact**: If the only time you hold your child is when he cries, the child soon learns he has to cry to be held. Picking him up only when he cries is a good way to spoil a child. Make sure you spend twice as much time holding your child when he’s not crying. The child will at least learn he doesn’t have to cry to be held. | **10 mins** |
|  | **Common Complaints**  **Invite** second time parents to list the usual worries: jaundice; temperatures; teething; colic; nappy rash; oral thrush; and listen to their opinions. N.B Before this session ensure information is accurate by referring to the Department of Health website or consult a health visitor.  Conclude by encouraging parents to go to GP or midwife up to 10 days post-delivery; health visitor or pharmacist for advice that will be up-to-date and safe. | **10 mins** |

| **6** | **EMPATHY FOR ADULTS** | **10 mins** |
| --- | --- | --- |
|  | Remind the group of the empathy role play for the distressed baby in Week One. Mention that we will now revisit the importance of empathy between adults.  ROLEPLAY  Invite a parent to play the “tired parent” and write the following script on a card for them to say:  a) Tired parent snaps “about time you're home to take your share of this parenting business. My day's been awful”.  Home coming parent (PGL) says “Oh no! It's a misery coming home when you're always so bad tempered. And my day has been tough too.”  Discuss how the tired parent might feel and behave and then how the coming home parent might feel and behave.  b) Tired parent repeats the same script as in role play (a).  Homecoming parent (PGL) says “Wow, you seem exhausted. Shall I make you a cup of tea while you put your feet up?”  Process as before. Remember to de-role: “My name is... I was born in...” |  |

| **7** | **POSTNATAL DEPRESSION** | **5 mins** |
| --- | --- | --- |
|  | Ask the group for their understanding of what the term means - from 5 day baby blues to full postnatal depression (the latter can start any time in the first 6 months post-delivery). N.B. 10-15% experience postnatal depression ­ Reference Women's Mental Health: In to the Mainstream 2002 (s12) DoH 2002.  Encourage the idea that it is important to seek professional medical help sooner rather than later. If the mother's behaviour is worrying it is nothing to be ashamed of and must not be explained away as “tiredness” or “she'll pull herself together soon”.  Most depressed mums turn to their partner for emotional support rather than anyone else. This can be a huge strain since dads feel the serious responsibility of loving and providing for a vulnerable baby. They may also feel excluded from the mother and baby “bubble” and may also feel depressed.  **TOP TIP**: Signpost to the local Children's Centre and encourage parents to find contacts and sources of support within and outside their families.  **TOP TIP**: Be alert to changes in each other and if feeling under strain, ask for help.  See [www.oxpip.org.uk](http://www.oxpip.org.uk/) - offers intensive therapeutic help to mothers with severe postnatal depression in Oxford and also offers training to professionals nationally on attachment theory and infant mental health. |  |

| **8** | **SPECIAL GIFTS FOR MY BABY**   - Ask parents to remember a special gift they received from their parent or parents. Not a material gift, but rather a gift they have been able to take with them into adulthood. Give an example such as, “The gift of the appreciation of nature”. - Have parents recall the ways in which that gift has enabled them or empowered them in their lives. - Ask parents to share their gift with the group. Suggest that if they can't remember any then think of one they would have liked. - Ask parents to find a comfortable position and close their eyes. Assure parents that they are closing their eyes to block outside stimulation and to be able to fully participate in the activity. Have parents practice slow breathing to relax. - Ask parents to listen to your voice.   *Imagine a beautiful box. The box may be any colour, size, style. This box is a rare treasure, not only because of its beauty, but because inside the box are five gifts you wish to give your child or children. The gifts are not material gifts, but rather treasures that will enable your child or children to be happy, healthy, successful human beings. These gifts will be with them for the rest of their lives. Such a gift may be the gift of honesty, or love of nature. [Long pause to choose the “gifts”] Now, imagine your new baby and/or your other children gathered around you. See yourself giving the treasure box to them. Watch the delight on their faces as they open the box, discover the treasures and embrace them for their own. Feel yourself experiencing an overwhelming sense of well-being and peace.*  *Now, open your eyes, wiggle your fingers and toes and come back to the group.*   - Ask parent to share one of the gifts they would like to give to their child. Is it the same gift they received from their parents? - Invite parents to write their five gifts on small, pretty cards.   IDEAS AND SUGGESTIONS  Parents will be touched by this activity. Be prepared to assist those parents who could not identify any gifts. Use universal values as their gifts, for example, the gift of love. | **10 mins** |
| --- | --- | --- |
| **9** | **STAYING IN TOUCH**  Remind parents again that the group will meet again once babies are about 6 weeks old – confirm that date now if you have it agreed.  As an incentive, tell parents that a photographer has been arranged to take photos of the parents and their baby at the first group which they will get a copy of.  Explain that group facilitators will also be in touch by phone about once every 2 weeks to see how things are going after the group so make sure that you have everyone’s contact details.  Encourage the group to think of other ways they may want to stay in touch with each other. | **5 mins** |
|  |  |  |
| **10** | **FEEDBACK** | **5 mins** |
|  | **Give out** feedback forms and mints, as in previous weeks, and thank the group for completing them. |  |
| **11** | **GOODBYE CIRCLE – A WISH FOR THE FUTURE**  **Stand** in a circle, and say how much you’ve enjoyed being with the group over these six sessions and one special memory that you will take away from them is….  Provide every parent/couple with a small box for their 5 gifts/cards.  **PRESENTATION OF CERTIFICATES**  **Give out** to each parent a certificate with someone else’s name on it; **invite** someone to present the certificate they are holding to the appropriate person, while the others applaud; this parent then presents to the next person, and so on until everyone has received their own certificate.  GOODBYE CIRCLE: Web of Friendships  **Invite** the group to stand in a circle. **Mention** that you are going to weave a web of friendship as a way of saying goodbye.  **Ask** the group to think of one important thing they will take away from the course; **give** everyone a few moments to reflect.  Keeping hold of the free end, and unravelling a length of string, **throw** a ball of string to one parent; **invite** them to say what they will take away, then to throw the ball of string to someone else, unravelling enough to throw it across the circle while holding onto the string with their other hand. The person now holding the string does the same; **repeat** until everyone has had a turn and you are all connected together.  **Pause** to let people comment if they wish, taking time to enjoy the moment together before you ask them to drop the web onto the floor. | **5 mins** |
