# Supplementary material for: Using intervention mapping to develop a culturally appropriate intervention to prevent childhood obesity: the HAPPY (Healthy and Active Parenting Programme for Early Years) study
Source: Int J Behav Nutr Phys Act. 2013 Dec 28;10:142. doi: 10.1186/1479-5868-10-142 (PMC3895739; doi:10.1186/1479-5868-10-142)
Supplement: Additional file 4 — a. MASTER HAPPY Antenatal Manual FINAL VERSION. b. MASTER HAPPY Postnatal Manual FINAL VERSION. c. HAPPY manual resources. [file 1479-5868-10-142-S4.zip › 1523029418990916_add4b.doc]

**CONTENTS**

Section	Page

Welcome to the HAPPY Postnatal Manual	3

Session One	9

Session Two	29

Session Three	51

Session Four	67

Session Five	83

Session Six	101

**WELCOME TO THE HAPPY POSTNATAL MANUAL!**

This introductory section aims to provide you as practitioners with some background information about the development of the HAPPY intervention.

BACKGROUND

Childhood obesity is associated with a range of adverse consequences including metabolic abnormalities, increased risk of Type II diabetes, musculoskeletal and psychological problems, and adult obesity. Recent obesity trends in children aged 2-11 years indicate that although the rate of increase of overweight and obesity is levelling off, obesity among children is still high. There is also evidence that antenatal factors (e.g., gestational diabetes, maternal weight gain) and early postnatal factors (e.g., feeding practices) play a key role in the development of childhood obesity, and that growth trajectories of children indicate that the risk of childhood obesity can start before the baby has been born, and is present in the first few months of life. To summarise, evidence indicates that the following factors have been directly associated with childhood obesity:

- Mother is obese (genetic or environment)
- Rapid weight gain in the infant
- Short sleep duration
- Less than 30 minutes daily physical activity,
- Consumption of sugar-sweetened drinks,
- Screen-viewing,
- Parental feeding practices
- Parenting styles

It has been recommended that programmes to prevent the risk of childhood obesity should:

- Be multi-faceted (diet, physical activity, sedentary behaviour, behavioural change techniques)
- Involve parents and be family-based (wider family)
- Target skills and competencies to promote healthy behaviours
- Address parenting practices and parenting skills
- Be delivered by trained practitioners
- Be underpinned by behaviour change theory

Therefore, the BIB childhood obesity prevention intervention has incorporated these components.

BABIES WITH SPECIAL NEEDS

It is possible that you may have a baby arrive in your group who has specific or additional health needs e.g. cleft lip, premature, a health or developmental condition. In this case, they may be given specific feeding or other care advice by their health care professional or team that meets the clinical needs of that baby and which may be different to some of the general information aimed at healthy term babies here in this pack –be sure to acknowledge this and encourage parents to follow the advice they have been given by their health professional (e.g. dietician, physiotherapist, paediatrician, specialist nurse) and discuss any issues or queries with their health care team. Encourage them to participate as fully as possible with the group discussions and activities whilst adapting to suit their own baby’s needs.

VITAMIN D

Vitamin D is important in bone development and strength, as well as muscle strength and the immune system. It is thought that about 1 in 4 people do not have enough vitamin D in their body. We get most of our Vitamin D from sunlight on our skin – about 90% is made this way. The amount of sunlight we need to make enough vitamin D varies – going outside for 15-20 minutes most days in the summer months without sunscreen should be enough to last throughout the year. People with darker skins need more time to make the same amount as people with fairer skins. It’s important to take care never to redden or burn. A few foods do contain vitamin D (e.g. oily fish, fortified breakfast cereals and fat spreads, eggs) so these are useful foods to eat but they do not contain Vitamin D in high enough quantities to give us all that we need. Babies rely on body stores laid down during pregnancy so if Mum’s vitamin D levels were poor during pregnancy (e.g. due to spending a lot of time indoors or covering up) they may not have enough. Taking vitamin D supplements can be a useful way for people who need extra (e.g. during pregnancy , breastfeeding or children who are growing) or whose bodies can’t make enough from sunlight for other reasons (e.g. covering up, spending most of their time indoors). NHS Healthy Start vitamins for women and for children provide Vitamin D. They are available free of charge to eligible children and women and at low cost to others. Distribution schemes and policies about when they are recommended from vary locally. It is important to encourage parents to find out where they can get these from (Health visitors are usually the best starting point) and to encourage them to use them. Group leaders should check local NHS policy and recommendations about vitamin D supplements as these may vary according to the needs of the local community (e.g. in some areas where the community is particularly at risk, public health departments may suggest starting supplementation earlier than 6 months, when the Healthy Start scheme begins.)

HEALTHY START FOOD VOUCHER AND VITAMINS SCHEME

This scheme is available to families with children under five on certain benefits and provides vouchers for free milk or fruit, vegetables and vitamins. More information should be available to parents from health visitors and the healthy start website at <http://www.healthystart.nhs.uk/>

LOSING WEIGHT AFTER HAVING A BABY

For women who are overweight or obese it’s really important to start to think about some weight loss now the baby has arrived and particularly before another pregnancy. The important things to know and pass on when appropriate are ….

- Mums who want to lose weight after having a baby should avoid very restrictive or unbalanced diets.
- The 6-8 week check is a good time to start to think about this for many women and discuss weight loss with a health professional.
- The healthy eating and exercise information given in this course will help.
- Some may also want to join a weight loss group or follow a programme at some point after their 6-8 week check–choose one where the dietary advice is based on the Eat Well plate (i.e. a balanced healthy diet) and target weight loss is about 0.5-1 Kg/week (1-2lbs), no faster.
- Weight loss at this rate achieved by healthy eating and regular exercise will not affect the quality or quantity of milk produced. Breastfeeding Mums who join a weight loss programme should let the group leader know that they are .breastfeeding when they join.

OVERVIEW OF THE BIB CHILDHOOD OBESITY PREVENTION INTERVENTION: THE HAPPY PROGRAMME

The HAPPY programme has been developed based on an intervention mapping framework, which consists of six steps

1. Needs assessment
   - Research evidence
     - Published literature
     - Information from Bradford
   - In depth discussions with:
     - Practitioners
     - Parents and wider family
     - Parenting experts
2. Matrices
   - We used these to identify the desired behaviours, and the reasons why people might find it difficult to do these behaviours (barriers)
3. Theory-based intervention methods and practical applications
   - Identify and use theories that can help to create effective interventions
4. Intervention program
   - Develop the intervention alongside practitioners and users
5. Adoption and implementation
   - Pilot the intervention
   - Train delivery staff
   - Deliver the intervention
6. Evaluation plan
   - Identify ways to assess whether or not the intervention worked
     - E.g., if the intervention aims to increase physical activity, we need to measure physical activity levels before and after the intervention to see if the intervention helped to achieve better levels

THE ‘FAMILY LINKS’ PARENTING PROGRAMME

Family Links Nurturing Programme is a parenting programme to promote emotional health and well-being, relationship skills and positive behaviour management strategies for parents/carers. It is based on four key building blocks for successful relationships and confident parenting –

1. Self-esteem and self-awareness
2. Appropriate expectations
3. Positive discipline
4. Empathy

The Nurturing Programme has been successfully run across Bradford for the last 5 years, mainly in Children’s Centres

Family Links have recently developed an Ante-natal programme based on similar key principles which we are using for this intervention. Therefore, we decided to join forces and build on the existing structure and format to produce the HAPPY programme.

Here are some details about the HAPPY Programme:

- It is aimed at women who are pregnant with a BMI 25+
- Fathers, other significant carers will be invited to attend but expected to attend all sessions
- The intervention will last for 12 sessions – 6 during the ante-natal phase and 6 for the post-natal phase until baby is about 9 months old
- It will be delivered by trained and experienced Family Links practitioners from Bradford
- 6 groups will run for 10-12 parents/carers each
- Sessions will run in community venues around Bradford
- Sessions will last around 2 ½ hours
- We plan to run at least one group evening and one group Saturday to increase access

We will focus on improving behaviours in the following areas:

- Parenting practices, parenting skills and parenting styles
- Maternal diet - pregnancy and post pregnancy
- Infant diet
- Maternal physical activity
- Infant physical activity and sedentary behaviour
- Knowledge, skills and competencies
- Culturally appropriate

What do we want to achieve?

- Infants with a healthy weight
- Confident and informed parents
- Healthy family diet
- Mothers feeling confident about how, what and how much to feed their infants – and doing it!
- Infants receiving a healthy diet
- Mothers being active and providing opportunities for physical activity for their children

**WELCOME TO THE WORLD!**

**Postnatal Session One (babies aged approx. 6 weeks)**

**STEP BY STEP**

PLAN FOR TODAY

|  | **ACTIVITY** | **TIME** | **MATERIALS NEEDED** |
| --- | --- | --- | --- |
| 1 | Welcome | 5 mins | Refreshments, music and player, ‘Welcome’ sign , attractive table cloth, flowers, badges, stickers, felt-tip pens, plan for today, attendance sheet, reward system, etc |
| 2 | Icebreaker: Introductions | 10 mins | Group dividers |
| 3 | Celebration of Birth | 20 mins | Group Rules from AN sessions  Flipchart  Board: 4 Constructs |
| 4 | Feeding Baby | 10 mins | Hand-outs -‘Happy Guide to Feeding your Baby’,‘Crying’ (AN4), ‘Coping with the First Few Weeks’ (AN6) |
| 5 | Infant Play | 15 mins | Hand-out: ‘HAPPY Book of Play’ Booklet’, Flip-chart |
|  | *TEA & COFFEE BREAK* | *10 mins* | *Refreshments& Photographer* |
| 6 | Time to Play | 15 mins | Empty containers, popcorn kernels, tape, lollipop shapes on card, Baby Sam, mat, towel. |
| 7 | Giving Praise Effectively | 20 mins | Board: 4 Constructs  Board – Steps in Giving Praise Effectively  Flipchart |
| 8 | Looking after Mum | 25 mins | Handouts –  ‘Physical Activity for new mums quiz’  ‘Physical Activity for new mums’ (AN6) ‘Smart Snacks’ (AN2)  ‘Healthy Eating Choices’ (AN3) |
| 9 | Game: The Power of Positive Words | 5 mins |  |
| 10 | Time to Have a Go | 10 mins | Hand-out - ‘Time to Have a Go’ |
| 11 | What did you think of today? (Feedback) | 5 mins | Hand-out - ‘Weekly Feedback’ |
| 12 | Goodbye Circle | 5 mins |  |

150 mins

**WELCOME TO THE WORLD**

**HAPPY Postnatal Programme (babies aged approx. 6 weeks)**

**Session One**

**A CELEBRATION OF BIRTH**

**STEP BY STEP**

**1.	WELCOME	5 mins**

**Display** board with ‘Welcome to HAPPY!’

**Greet** group members warmly as they arrive, have background music playing, and offer refreshments. **Ask** parents to make name badges, and to sign the attendance sheet.

**Welcome** the group back again after a long break and **congratulate** them on the birth of their babies. **Display** and briefly go through the Plan for Today of topics to be covered in the session and the plan for the postnatal programme.

**Remind** people of ‘housekeeping’ items:

1. Tell everyone where the toilets and fire exits are.
2. Explain where facilities are for changing babies and feeding them if necessary.
3. Explain about refreshments and the break mid-way through each session.
4. Remind the group of starting and finishing times, and stress the need to be punctual, although this will be more difficult as they have to get babies ready as well!

**2.	ICEBREAKER: INTRODUCTIONS	10 mins**

**Divide** people into pairs in a fun way (e.g.: different colours, fruits etc.). Ask the group to move into their pairs and introduce each other once again and introduce their new baby if present, or share information about the new baby responding to the following statements:

“My name is ………………. My baby’s name is ……………………………..

My baby was born on ……………………. and weighed ……………………

My family consists of ………………………”

After up to 5 minutes, invite each person to feedback on their partner or themselves – whichever each pair prefers.

**3.	CELEBRATION OF BIRTH	20 mins**

Begin by emphasising that this first section will be a chance to remember many of the things we talked about in the antenatal programme and to see how much we have already been able to put into practice with our babies and in our family life now.

**Re- present** the Group Rules which were used in the antenatal sessions and remind people why they are useful in helping us all feel safe and supported in the group.

**Check** that everyone is still happy with them.

- Would anyone like to change anything on them now?
- Do we need to change anything now that we have babies in the group?
- Has anyone made any changes to their family rules since the baby has been born e.g.: sharing out chores in the family, finding time for family activity together with the baby, making sure mum or dad gets a bit of nurturing time away from the baby?

**Divide** people into groups of 3 or 4 people using fun group dividers.

**Share** experiences of how life has been with the baby, highlighting successes and joys as well as any issues or concerns about progress and caring for baby.

After up to 10 minutes, **ask** parents to contribute their ideas to a prepared flipchart:

Successes and Joys (Green)	Issues or Concerns (Red)

NB – don’t address the issues now. Identify when there will be a chance to address the issues within today’s session or the rest of the postnatal programme and offer time to talk in the break for any particular worries which may need signposting.

**Remind** people of some of the key messages they were given in the ante-natal programme and ask them to make connections with the list on the flipchart: e.g.: empathy; baby’s 5 senses; self-esteem; using our personal power; soothing techniques; nurturing ourselves; tuning into baby’s needs; making eye contact; talking to my baby; baby massage; using your Memory Book to record what’s happened, thoughts and feelings.

**Display** relevant boards and flipcharts from previous sessions as prompts.

**Re-visit** the 4 constructs as a quick reminder of how they link with all areas of life and with the issues raised above.

**4.	FEEDING BABY	10 mins**

**Note to group leaders:** we will talk about infant feeding at various stages in the course –the information contained in the **hand-outs 🕮**, booklets and recommended leaflets is all up-to-date, evidence-based guidance. It will be useful to have this in mind and use as you go along.

**Remember that all babies are different and these are only guidelines** – if in any doubt remind parents to discuss with their health visitor or other health professional.

Breastfeeding is the best way to feed a baby but the first few weeks may not have been easy so mums who are breastfeeding may need a lot of encouragement and support.

Within your group, parents may be breastfeeding, bottle feeding with expressed breast milk or infant formula, or doing a bit of both. The group facilitator therefore has the potentially tricky task of encouraging and supporting the breastfeeding mums whilst remembering the needs of those who are at this time are not breastfeeding. The emotions behind this may be complicated for some.

For any discussion/group/pairs work around feeding baby that you do it may be best to group mums together with those currently feeding in the same way as they can support each other and share ideas.

KEY MESSAGE:

Regardless of the chosen method, feeding babies in the first few weeks and months can be one of the most time consuming, demanding and sometimes worrying parts of caring –but it is worth the effort!

Feeding well now is vital to our babies’ short and long term health and development.

GROUP ACTIVITY – FEEDING BABY

Building on the discussion earlier about successes and joys or issues and concerns this will be a chance to share with each other some thoughts about feeding the baby.

**Ask** the group to work in **pairs or small groups**, grouping people with those feeding in a similar way.

**Ask** the group to discuss the following (**prepare** a flipchart in advance, enough for each group/pair, with the questions on them):

How is feeding going?

What has helped you in your chosen method of feeding?

How have you got on with seeking support from friends or family member with their chosen method of feeding – has it helped?

Is there anyone else that has helped? Any surprises?

Give out ‘HAPPY Guide to Feeding your Baby’ **hand-out 🕮** and invite parents to have a quick look through it in the group, drawing attention to the relevant sections depending on the issues that parents are facing.

**Signpost/remind** about previous information given in antenatal course e.g.

- Size of baby’s stomach, (about the size of a ping pong ball);
- Coping with crying – **hand-out 🕮** from antenatal session 4 called ‘Crying’ (have some spares available in case);
- ‘Coping with the first few weeks with your new baby’ **hand-out 🕮** from antenatal session 6;
- Breastfeeding and bottle feeding resources and information (if needed);
- Advise to seek advice from Health visitor if any concerns

**5.	INFANT PLAY	15 mins**

GROUP DISCUSSION

**Ask** the group the following questions:

What are your thoughts about playing with your baby?

What are your worries about playing with your baby?

Do you play any games now? What ways do you play at the moment with your baby?

Does your baby spend any time on their tummy when they are awake?

**Explain** what Tummy Time is. Tummy Time is when you place your baby on their tummy whilst they are awake. It helps babies learn to push up, roll over, sit up, crawl, and pull up to standing.

Flip chart: **Divide** a flip chart into two vertically, head one column ‘WORRIES/RISKS’ and complete this column in red pen. Head the other column ‘BENEFITS’ and complete this in green pen.

**Ask** the group about the risks/worries, ensure the list includes:

- hurting/injuring my baby, especially the neck/head
- worried that putting my baby on their tummy could cause sudden infant death
- my baby doesn’t like to play
- my baby is not old enough to play yet
- I don’t know how to play with my baby

**Dispel** myths: **read** the following passage out to the parents, explain that there is a lot of information and tell the group that they will get this information in a booklet that they can take home and read again.

Babies can begin to play from being new-born. As long as you play carefully with your baby you won’t hurt or injure them. You can move their limbs around gently and guide their movements; this will help them to develop strength in their muscles and co-ordination. When your baby is very young and cannot control their head yet, make sure that when you are playing their head and neck is supported. If your baby starts to be irritable or cry they are telling you that they don’t want to play anymore. There will be times that they don’t want to play and times that they do. Play with your baby when they are attentive and engaged.

If you’re finding it difficult to play with your baby the first thing to do is stop giving yourself a hard time about it. Playing doesn’t come naturally to everyone. One reason that you find it difficult could be that your parents didn’t play with you during your own childhood. Remember to keep your voice light and fun, if you get too serious you baby might not want to play.

Putting a baby on their tummy whilst they are awake is not at all dangerous and will help to develop strength in your baby’s neck and back, this will help them to reach developmental mile stones such as rolling over, crawling, sitting up and walking. If your baby is uncomfortable on their tummy then look at pages 6-7 of the ‘HAPPY book of play’ **hand-out 🕮** for some hints and tips about how to make Tummy Time more enjoyable. If the baby falls asleep when they are on their tummy they must be placed on their back. Make sure you or someone else is always supervising your baby when they are spending time on their tummy and places them on their back if they fall asleep.

**Ask** the group for a list of benefits, ensure the list includes:

- Having fun and bonding with your baby
- Giving the baby love and attention
- Developing movement skills
- Promotes a healthy weight
- Improves brain development
- Improves bone development
- Better sleep
- Develops creativity, curiosity and motivation
- Improves social skill development
- Ensures babies become active toddlers and children: this can lead to the child enjoying and being good at sport/exercise during adulthood.

**Clarify** benefits: **read** the following information to the group.

Playing with your baby is one way to give them love and attention. It is also incredibly important so that your baby develops movement skills. Infancy is a critical time for developing the connections between the brain and the muscles. Research shows that stimulating play activities positively affect brain, bone and muscle development, promotes healthy weight and social skills. It also helps babies to become active, fit and healthy toddlers and children. Babies who spend time on their tummies will have better development of their movement skills such as sitting up, rolling over and crawling if you encourage and support them to do them. Infants who are confined to a small play space or spend most of the day in a car seat are likely to take more time to reach movement milestones such as rolling over, sitting, crawling and walking.

Playing with your baby can activate their appetite for life, energise them to explore new things, allow them to engage with the world around them and develop their curiosity, creativity and motivation. Physical interactive play is also very important for long-term emotional health. This form of play has anti-stress effects and increases positive emotions such as joy and happiness. If you have lots of physical playtimes with your baby you will be developing the emotional areas of their brain so that they will grow up to manage their emotions and stress more successfully.

Playing with a very young baby can be as basic as placing them in different positions — sometimes on their front, sometimes on their back — and helping them to move their arms and legs around. As you explore ways to interact with your baby, keep in mind that infants this age are easily over-stimulated, your baby will let you know if he/she doesn’t want to play anymore by becoming irritable and crying. Don’t force your baby to play: only play when your baby is attentive and engaged.

**Tea and Coffee Break	10 mins**

**(Photographer will offer photos of babies with parents/carers)**

**6.	TIME TO PLAY	15 mins**

**Explain** that at each HAPPY session the group will get new ideas for games to play with their babies. They can also have a go at the games in the sessions if their baby is alert and ready to play. Reassure mums that it’s okay if their baby is asleep or doesn’t want to play; they can try the games when they get home and their baby is ready to play.

**Remind** the group that it is important to remember that each baby is unique and will develop and reach milestones at different rates and times. If their baby isn't quite ready for the different play activities, don't worry — just try them again a few weeks later. **Introduce** to the group that it is important to praise even very young babies for what they do and how they play. It is never too early to start giving positive praise for all that your baby does and how they are. We will spend some more time thinking about this and having a go later in the session.

LOLLIPOP GAMES

**Explain** that this game will strengthen baby’s neck muscles. Once a baby has the strength to move and hold their head up they can begin to enjoy the sights and sounds around them.

For this game you will need thick white card and black marker pens.

**Show** the group how to make a lollipop. Use pre-cut lollipop shapes cut from white card and encourage the group to draw a simple black and white design on the card. Explain that these very young babies love simple black and white designs. e.g.

**Demonstrate** using the Lollipop with Baby Sam. **Explain** that the baby is held on the lap and its head is supported. Allow the baby to focus on the lollipop and slowly move it from side to side so that the baby tracks the lollipop with its head and eyes. You can tell when the baby loses interest because they turn away. Encourage mums to give the game a go if they feel that their baby is ready to engage in play.

TUMMY TIME!

You will need a mat/blanket and a towel

**Remind** the group that Tummy Time helps babies learn to push up, roll over, sit up, crawl, and pull up to standing. It helps them to develop balance and strength in their neck, back and limbs.

**Explain** that the best time for Tummy Time is when the baby is awake, alert and not crying. A good time is after their bath or burping. At this very young age do Tummy time for 2 – 5 minutes, 2 – 4 times a day. Demonstrate by putting down a clean mat/blanket out on the floor and put Baby Sam down lying on his/her front. **Explain** that the position can be uncomfortable for some babies because they are used to lying on their backs. **Demonstrate** the following to the group to show them techniques if their baby doesn’t like being on their tummy, encourage them to join in with their baby if they are awake and engaged:

- Place a rolled up towel under their chest keeping their arms over and in front of the towel, this balances their weight and helps them to lift their head
- Lie on the floor in front of them so they can see your face
- Lay them on your chest: they will enjoy being close to you
- Place a baby-safe mirror in front of them where they can see it

SING-A-LONG AND MOVE – THE HOKEY COKEY

**Explain** that games where you guide a baby’s limbs gentle are great for developing muscle strength, co-ordination and the connections between the brain and muscles. **Sit** on the floor with your knees bent and feet flat on the ground, lay Baby Sam on your lap looking up at you. **Invite** the group to join in with or without their baby as you sing the hokey cokey. Gently move Baby Sam’s arms and legs in and out with the song. On the line “ohhh the hokey cokey”, roll the babies hands around each other.

At the end of the song encourage the whole group to clap: mums can gently guide their baby’s hands into clapping if the baby is alert and engaged.

**7.	GIVING PRAISE EFFECTIVELY	20 mins**

KEY MESSAGE: Praise is the magic ingredient in relationships. It’s a powerful way of encouraging and supporting babies, children and adults, showing that we are pleased, enthusiastic and grateful. Praise works wonders in helping children and their behaviour and gives guidance to even the youngest children in what is expected of them.

**Introduce** the topic briefly, referring to the Four Constructs and reminding people that this is a method of positive discipline.

Demonstrate a brief role play of giving praise to a baby – as a facilitator/parent with baby Sam give praise to the baby such as “What a lovely smile”, “Ooh, you smell lovely”, “I’m so glad you’re my baby” etc.

Demonstrate only the positive version of praise for this age group, but ask the group what they notice about how you gave praise.

Display the “Steps in Giving Praise Effectively” board and make connections with what the group has shared.

STEPS IN GIVING PRAISE EFFECTIVELY

1. Give the child/baby all your attention
2. Move close to the child/baby
3. Look pleased and share their pleasure
4. Be specific: describe what you like
5. Ask the child what he/she thinks (even if they can’t reply at this age)
6. Seek eye contact
7. Mean it – be sincere and let it show in a warm tone of voice
8. Touch the child/baby gently
9. Give pride to the child/baby (‘You deserve to feel proud of yourself’)

**Allow** a few minutes for discussion and mention that it may be hard to introduce all 9 steps straight away. **Suggest** using one or two at first and gradually build up to using all nine. **Refer** parents to Parenting Puzzle Pg 24 for a reminder of the steps.

EXERCISE ON PRAISE FOR DOING AND PRAISE FOR BEING

**Explain** the difference between the two kinds of praise. Praise for doing tells someone that we have noticed and appreciated their efforts. Praise for being tells someone that we value them just for being who they are, for their appearance or their innate qualities. We will look at some of the differences to help explain this.

**Display** on the flipchart the headings in the central column as below, including some of the suggestions listed and omitting the arrows for being and doing.

**Encourage** the parents to come up with more ideas in small groups so you are not offering all the ideas. Facilitators should join groups to help generate a good list.

**Write up** the groups’ suggestions and then ask them to decide whether the praise is for being or doing and draw an arrow accordingly.

Example:

| For doing | What we can praise/encourage babies for | For being |
| --- | --- | --- |
|  | 0 – 3 months  *Gurgling, smiling*  *Dimples*  *Cooing*  *Kicking legs*  *Curly hair* |  |
|  | 3 – 6 months  *First tooth*  *Smiling*  *Rolling over*  *Sitting*  *Babbling*  *Soft hair, skin etc*  *Being my baby* |  |

**Remind** the group of the value of praising other adults, family members etc. as well as the children in the family and to praise themselves.

**8.	LOOKING AFTER MUM	25 mins**

It is important for mums (and dads) to look after themselves so that they can cope with the demands of life with a new baby. Good intentions about eating regularly and healthily and getting enough exercise and rest can get side lined by other things. Tell the group that you are going to spend the next 20 minutes thinking about what they can do to stay healthy**.**

**Give out** the ‘Physical Activity for New Mums quiz sheet **hand-out 🕮** (also given in session 6 of the antenatal programme) and **ask** the group to split up into pairs to answer the questions. Once complete, give the group the ‘Physical Activity for New Mums’ **hand-out 🕮** and go through it with them to answer the questions.

**Invite** the group to practice pelvic floor exercises.

**Pelvic floor exercise**

Your pelvic floor muscles support and protect your bowels, uterus and bladder. Hormonal changes in pregnancy and the pressure of your baby on the pelvic floor can loosen these muscles.

This exercise will strengthen your pelvic floor muscles. You can do this exercise either sitting or standing.

- To find your pelvic floor muscles imagine you’re stopping your urine flow when you pee: the muscles contracting are your pelvic floor.
- Squeeze and draw up the muscles.
- Do it quickly, tightening and releasing the muscles immediately.
- Then do it slowly, holding the contractions for as long as you can (but not more than 10 seconds) before you relax.
- Do five lots of pelvic floor quick squeezes and five lots of slow squeezes five times a day and build up to doing ten squeezes five times a day.
- Ask group members if they are able to locate their pelvic floor muscles and go over the exercise with anyone who is struggling.

**Remind** the group that a healthy balanced diet is important for all parents to help them recover from pregnancy and birth, cope with the demands of a baby and keep healthy for the future. If they are breastfeeding it is important to be eating well to replenish the body’s supplies of the nutrients they are passing onto their baby.

**Give out** the ‘Smart Snacks’ and ‘Healthy Eating Choices’ **hand-outs 🕮** from ante-natal sessions 2 and 3, and allow the group a few minutes to read these. **Ask** the group to think about their fruit, vegetable and fibre intake: are they eating enough? Ask the group what types of drinks they are having and suggest that they swap sugary drinks to lower calorie drinks. For those mums who are breastfeeding, there are particular messages to be aware of:

- Breastfeeding does increase mum’s requirement for some nutrients and these can be met by the body’s stores built up in pregnancy and eating a balanced varied diet and eating to appetite –**there is no need to ‘eat for two’ but very restrictive diets should be avoided.**
- Breastfeeding mums are recommended to take a supplement containing 10mcg of Vitamin D –this is the amount in the free or low cost NHS Healthy Start Vitamins for Women –ask your Health Visitor about them if you are breastfeeding. (Women qualifying for free vitamins under the Healthy Start Scheme will also be eligible for vouchers for free fruit and vegetables and cows’ milk. More information is available at [www.healthystart.nhs.uk](http://www.healthystart.nhs.uk/))
- Many people forget to drink enough when they are busy or tired and breastfeeding increases fluid requirements. Practical advice that can help is to encourage women to have a drink themselves when they sit down to feed.
- Breastfeeding mums should limit their intake of caffeine containing drinks such as cola, and coffee and should limit alcohol to 1-2 units, once or twice a week.

Some mums may be thinking about losing weight now that the baby has arrived and especially before another pregnancy. It is important that this is done safely. **Refer** to the information in the Introduction to this manual for guidance around this issue.

**9.	GAME: THE POWER OF POSITIVE WORDS	5 mins**

The object of this game is to apply positive and praising adjectives to themselves. Invite everyone to think of a positive word that starts with the same letter as their name e.g.: Adorable Abda, Magnificent Mandy and to think of something that they can praise themselves for e.g.: getting themselves to the group, having a go at some physical activity, doing their pelvic floor exercises regularly etc.

**10.	TIME TO HAVE A GO	10 mins**

Display on a board the appropriate Time To Have a Go suggestions for the week.

- Praise everyone in the family at least once – for being and for doing.
- If you already give quite a lot of praise, notice what kind of praise you give, and how you give it – more for being or for doing? How about other adults in the house? We all need it.
- Look through the ‘HAPPY guide to feeding your baby’ **hand-out 🕮** to check that you are doing the best for feeding your baby and getting enough support in this.
- Think about whether you are managing to eat and drink regularly and are getting enough fruit and veg and fibre in your diet
- Try to spend a few moments planning some easy meals and thinking about how to organise them and who you can get to help if needed (refer to the ideas in ‘Coping with your Baby in the First Few Weeks’ **hand-out 🕮**).
- If you find you have increased your portion sizes or are relying on sugary or fatty snacks and takeaways to keep you going, have a look back at the ‘Smart Snacks and Healthy Eating Choices’ **hand-outs 🕮** and see if you can find some easy swaps to make. Record these in your Memory Book as a reminder.
- Keep practicing your pelvic floor exercises and increase your activity levels if you are able to do so.

**11.	WHAT DID YOU THINK OF TODAY? (FEEDBACK)	5 mins**

**Give out** weekly feedback forms.

**Play** relaxing background music whilst forms are being completed; place a container on the floor/table to collect forms.

**Thank** the group for completing them. If there are literacy issues the whole group could respond to questions on flipchart and facilitator can record responses.

**12.	GOODBYE CIRCLE	5 mins**

**Stand** in a circle and say what a good session it has been and thank everyone for coming back to the group.

Take turns around the circle to put a hand on the shoulder of the person next to you and thank them for being in the session.

Optional – Give out a ‘Praise is Magic’ magnet, Tip Sheet or bookmark as a tangible reminder of the session.

**Close** the session by saying how much you have enjoyed it and that you are looking forward to seeing everyone again at the next session and remind them of the date.

**WELCOME TO THE WORLD!**

**Postnatal Session Two (babies aged approx. 9 weeks)**

**STEP BY STEP**

PLAN FOR TODAY

|  | **ACTIVITY** | **TIME** | **MATERIALS NEEDED** |
| --- | --- | --- | --- |
| 1 | Welcome | 5 mins | Refreshments, music and player, ‘Welcome’ sign , attractive table cloth, flowers, badges, stickers, felt-tip pens, plan for today, attendance sheet, reward system, etc |
| 2 | Icebreaker: Changing Places | 10 mins |  |
| 3 | Family Feedback | 10 mins | Flipchart |
| 4 | Feeding Baby | 20 mins | Flipchart  Hand-outs - ‘Responding to concerns about feeding baby’, Start 4 Life – Introducing Solids’, others for reference. |
| 5 | Mums’ Physical Activity | 20 mins | Flipchart  Hand-out - ‘Physical Activity Ball for Adults’ |
| 6 | The Need for Structure and Routines | 10 mins | Board – Types of Boundaries  Social Baby DVD |
|  | *TEA & COFFEE BREAK* | *10 mins* | *Refreshments* |
| 7 | Infant Play | 20 mins | Group dividers  Baby Sam, mat, towel  Hand-out – ‘Time to Play Quiz’ |
| 8 | Self-Esteem and Personal Power | 20 mins | Flipchart, Boards – Influences on Behaviour, Cycle of Feelings and Behaviour |
| 9 | Maternal Diet | 10 mins | Hand-outs – ‘The Big Balancing Act’, ‘Tempted by Takeaways’ |
| 10 | Time to Have a Go | 5 mins | Hand-out – ‘Time to have a Go’ |
| 11 | What did you think of today? (Feedback) | 5 mins | Hand-out - Weekly feedback |
| 12 | Goodbye Circle | 5 mins | Gift or incentive |

150 mins

**WELCOME TO THE WORLD**

**HAPPY Postnatal Programme**

**Session Two (babies aged approx. 9 weeks)**

**BRINGING STRUCTURE TO YOUR LIFE**

**STEP BY STEP**

**1.	WELCOME	5 mins**

**Display** board with ‘Welcome to HAPPY!’

**Greet** group members warmly as they arrive, have background music playing, and offer refreshments. **Ask** parents to make name badges, and to sign the attendance sheet.

When they are all settled, go through the topics for the session briefly and introduce the reward system for today’s session.

**2.	ICEBREAKER: CHANGING PLACES	10 mins**

Ask the group to change places if …..

…..you are wearing anything blue

…..you have had some fruit or vegetables to eat today

…..you enjoy watching soap operas on TV

…..you have had extra physical activity this week

…..you have brothers or sisters

…..you have praised someone else in the family

…..you have nurtured yourself in the last week

**3.	FAMILY FEEDBACK	10 mins**

**Comment** briefly on last session’s feedback forms. Display the ‘Time to Have a Go’ from the last session and invite brief feedback:

- Praise everyone in the family at least once – for being and for doing.
- If you already give quite a lot of praise, notice what kind of praise you give, and how you give it – more for being or for doing? How about other adults in the house? We all need it.
- Look through the ‘HAPPY guide to feeding your baby’ **hand-out 🕮** to check that you are doing the best for feeding your baby and getting enough support in this.
- Think about whether you are managing to eat and drink regularly and are getting enough fruit and veg and fibre in your diet
- Try to spend a few moments planning some easy meals and thinking about how to organise them and who you can get to help if needed refer to the ideas in ‘Coping with your Baby in the First Few Weeks’ **hand-out 🕮** .
- If you find you have increased your portion sizes or are relying on sugary or fatty snacks and takeaways to keep you going, have a look back at the ‘Smart Snacks’ and ‘Healthy Eating Choices’ **hand-outs 🕮** and see if you can find some easy swaps to make. Record these in your Memory Book as a reminder.
- Keep practicing your pelvic floor exercises and increase your activity levels if you are able to do so.

**4.	FEEDING BABY	20 mins**

GROUP ACTIVITY - DISCUSSION:

**Check** with the group how feeding is going for them at present and if they have any particular concerns or questions at present.

**Record** these concerns on a flipchart.

**Discuss** the difficulties with feeding that mothers might be having using the following ‘what if …’ scenarios. If the parents in your group have particular concerns not covered by these examples, then add in their own questions.

1. What if my baby doesn’t want to feed for very long or doesn’t finish the bottle?
2. What if my baby feeds more on one day and less on the next?
3. What if members of my family are telling me to change the feed; start adding rusks to the bottle; start giving solids?
4. What if I need to feed my baby when I am out of the house?
5. What if my baby seems unhappy and unsettled in the evenings when I’m trying to make a meal for the family?

**Give** each small group 2 or 3 scenarios and ask them to identify some possible solutions to the questions they have and record them on flipchart. **Invite** them to use a range of booklets and **hand-outs 🕮** to help them find the useful information. Ensure you are familiar with current local guidelines on infant feeding – you may wish to have a copy available to help the group. (In Bradford this is the Every Baby Matters Guidelines for Good Nutrition in Bradford and Airedale – Infant Feeding, Weaning and Diet after Pregnancy and whilst Breastfeeding’).

Suggested booklets and **hand-outs 🕮** are:

- HAPPY Guide to feeding your baby (all parents should have one);
- Off to the Best Start (Start 4 Life booklet given out in antenatal group);
- NHS Guide to Bottle Feeding (http://www.unicef.org.uk/Documents/Baby_Friendly/Leaflets/3/guide_to_bottle_feeding.pdf);
- Expressing and Storing Breast milk (from Breastfeeding network);
- ‘Crying’ **hand-out 🕮** (given in antenatal session 4);
- ‘Coping with First Weeks with my New Baby’ (**hand-out 🕮** given in antenatal session 6).

Ensure that you have copies of all the booklets available for parents to look at during the session and ask if parents want any additional copies to borrow or take home to share with family members.

**Invite** the groups to feed back the answers they have come up with. **Share** with the group the **hand-out 🕮** ‘Responding to concerns about feeding baby’.

INTRODUCING SOLIDS: WHY, WHEN AND RECOGNISING

SIGNS IN THE BABY

KEY MESSAGES

In terms of infant feeding, the next stage that a parent will be planning for is the introduction of solids. Discussions on this aspect need to take place well in advance of the 6 months recommended time of weaning. This section is aimed to be a very brief introduction: there is more time in subsequent sessions to get into more detail. The key objective is to encourage parents to delay weaning until as close to 6 months as possible.

- Weaning is the gradual process of introducing semi-solid food whilst continuing with breast or infant formula milk.
- It takes place over a period of months when infants progress through different food consistencies, from puréed to mashed, to minced, to cut-up food until they can eat normal family meals.
- The purpose of introducing solid foods alongside an infant’s milk feeds is to:
- Give extra energy and nutrients (vitamins and minerals such as iron for healthy blood) when milk no longer provides enough to ensure the baby’s rapid growth, health and development.
- Provide the chance to try new tastes and textures based on family foods.
- Some infants may need weaning earlier than 6 months* however solid foods should not be introduced before 17 weeks (4 months) and the closer to 6 months the better.
- Exclusive breastfeeding (or formula feeding) is recommended for the first six months (26 weeks) of an infant’s life.
- Six months is the recommended age for the introduction of solid foods for infants whether breast, bottle or breast and bottle-fed.
- Breastfeeding (and/or breast milk substitutes, if used) should continue beyond the first six months, along with appropriate types and amounts of solid foods

*It should be noted that the age which infants need solid foods varies and all infants should be managed individually.

GROUP DISCUSSION

Have a flipchart prepared with the following 3 questions and invite some discussions to see what parents know already.

What is weaning?

Why do we have to wean infants?

When should weaning start?

**Ensure** the discussion includes the Key Messages identified above. **Give out** the booklet ‘Start 4 Life - Introducing Solid Foods’. **Invite** the group to work in pairs or small groups and using the information in the booklet on pages 6 and 7 answer the following question.

WHAT ARE THE 3 SIGNS THAT YOUR BABY IS READY FOR WEANING?

**Ensure** that they identify the 3 key facts:

•	Can sit up (initially with support) and hold head steady.

•	Can co-ordinate their eyes, hands and mouth.

•	Can swallow food.

**Invite** a brief discussion about some of the signs that can be mistaken for babies being ready for solid food. Refer to ‘Introducing Solid Foods’ for information.

**5.	MUMS’ PHYSICAL ACTIVITY	20 mins**

**Remind** mums about the guidelines for physical activity after giving birth.

- Walking and pushing the pram is safe to do in the first 6 weeks.
- If you have had your six-week postnatal check and the all clear from your doctor you can start to be more active.
- If you had a caesarean delivery or a difficult pregnancy you should be able to start exercising again at around 10 weeks postnatal. Make sure you talk to your midwife or health visitor before starting anything strenuous.
- When your postnatal bleeding has stopped, you can try swimming.

**Divide** the flip chart into two vertically with the titles ‘what I don’t like about physically active is…’ and ‘what I do like about physical activity is…’ Make sure that the lists include the following…

What I don’t like about physically activity is…

It makes me tired

It is boring

My muscles hurt

It takes too long/I don’t have time to do it

I might get injured

Doing it on my own OR doing it with other people

Doing it outside in my neighbourhood because it’s unsafe

What I do like about physical activity is…

It makes me feel energised

I sleep better after exercise

It makes me feel happy (reduces depression)

It makes me feel good about myself

It can help me lose weight more quickly

It is fun

It keeps my body including my heart and lungs healthy

KEY POINTS.

Take time to counter the negatives that the group has come up with.

You don’t have to do tiring vigorous exercise that makes your muscles hurt or where you might injure yourself to get health benefits from physical activity. 30 minutes of moderate activity that makes you breathe a little harder but you can still speak is enough. If it’s hard for you to find 30 minutes each day to be active don’t worry, you can do your physical activity in blocks of 10 minutes. If you find formal exercise boring you can still be active in your everyday life: e.g. swap driving to the shops for a brisk walk with the pram to the shop. Also remember that you don’t have to do physical activity outside. You could exercise with an exercise DVD or even while cleaning the house with energy. It counts! Or if you are worried about being active outdoors alone, ask a friend or your partner to join you.

**Ask** mums if they remember what ‘moderate’ activity is. **Remind** them about the ‘let’s get moving’ activity in antenatal session 3. **Talk them through** the ‘let’s get moving’ activity or if you have mums in the group who are safe to exercise (i.e. they have the ‘all clear’ from the doctor/health visitor) try the following activity.

LET’S GET MOVING

**Explain** that to help the heart and lungs get stronger they need to do activities which raise their heart rate for at least 10 minutes continuously. This level of activity is also required for other health benefits such as lowering blood pressure, cholesterol and weight loss. A good way to check that you are working at the right level to improve heart and lung fitness and general health is to do the talk test. As you exercise you should be breathing a little harder than usual and still be able to talk.

Now **ask** them to stand in a circle, ‘Zip Up’, and do the following: march on the spot, bend knees, wave hands above their head, punch arms out in front (for about 2 mins). **Ask** the group if they are breathing harder: can they still talk? **Check** by saying a nursery rhyme out loud as a group.

KEY POINT

You can use the talk test when you are out and about exercising to check you are working at the right level for you.

DISCUSSION AND MINGLE ACTIVITY

**Ask** the group to share any activities or groups that are available in their local areas which are good for mums to get back to physical activity. For example, is there a Fit Mums group near you? (Fit Mums is an exercise group available to mums to attend with babies)

**Write** a list of groups/activities on the Flip Chart

**Explain** to the group that now their baby is born the types of physical activities mums are able to do might be different, so the next exercise is about sharing ideas about how to be active. Remind the group that physical activity includes both “formal” exercise such as going to an exercise class or pushing the pram in the park, and “informal” activities such as walking to the shops, cleaning the house with energy and dancing with your baby in the living room. **Give out** the ‘Physical Activity Ball for Adults’ **hand-out 🕮**. Ask the group to look over the **hand-out 🕮** and think of some other activities that they do or could do which would fit into the activity segments of the ball.

**Ask** everyone to stand up and find a partner, decide who will go first.

‘Think of a list of physical activities that you could do this week. If you had a difficult pregnancy or a caesarean and are still recovering, make sure that you chose activities that are not too strenuous. Your activities might be as simple as walking to the shop or dancing with your baby. Facing your partner you will complete the sentence ‘one physical activity that I could do this week is…’

For example ‘one physical activity that I could do this week is push the pram in the park’.

Your partner will then share one physical activity they could do with you. Make your answers short and quick. Raise your hand and find another partner to share with. Continue sharing with different partners until I ask you to stop. As you go around you can use some of the activities that the other share with you if you like.

Now think over all the activities that you just shared and heard. Choose one of these activities that you are willing to do this week. Choose sensibly; think about how, when and where you will be able to do it. Think about whether your body is ready to do it, be gentle with yourself if you are still recovering from giving birth. When you are sure of your chosen activity turn to your partner and complete the sentence ‘the physical activity that I will do this week is…’

For example ‘the physical activity that I will do this week is going for a 10 minute brisk walk pushing the pram in the park’.

Sit down and close your eyes or look down. Picture yourself doing your chosen activity, what do you look like, where are you, what are you wearing. Imagine that you are feeling happy, confident, positive and are enjoying doing that activity. In your mind, say to yourself: ‘I am happy positive and confident and I am good at doing my activity’. Let the group sit in stillness for a moment.

**6.	THE NEED FOR STRUCTURE AND ROUTINES	10 mins**

KEY MESSAGE: Babies enter the world with a readiness to engage socially with those around them. They soon learn about the individual characteristics of their family members and their distinctive ways of relating, and the baby develops ways of relating back to them.

As attachment develops, strong emotions to do with dependency and security are established. So babies develop different attachment patterns with different members of the family and this is common.

However if the care is consistent and reliable and sensitive to the baby’s developing needs, then they will be likely to feel secure in the relationship.

**Introduce** the idea of time to communicate and play with your baby as a great way to develop attachments but also to help your baby learn social skills and to know what to expect – developing consistency and routines.

**Show** the clip from the Social Baby DVD of parents ‘playing’ with their babies and of the parents having to learn what is the right time/right place for play. (11.20 to 14.58 in the ‘Let’s Chat’ chapter)

**Invite** some discussion about what parents have seen and learned already about what their babies like to play, how they like to communicate and when they want to play. (May have to leave this out if not enough time).

**Remind** parents of the discussion in the antenatal programme about boundaries and consistency.

**Display** the Types of Boundaries board and/or refer to the cartoons in the Parenting Puzzle (PP45).

**Invite** parents in pairs to discuss the advantages of clear and consistent boundaries for children and babies and how they will feel.

**Invite** feedback and record on flipchart (ensure a mention of feeling safe, fair limits, clear expectations, stability with room to grow and explore, sociable, emotional needs met).

An authoritative (positive) style of parenting and routines within a baby’s day (including around sleep, play and eating) has been shown to have benefits for healthy outcomes in a child’s future.

**Tea and Coffee Break	10 mins**

**7.	INFANT PLAY	20 mins**

IN PAIRS

**Ask** the group to divide up into pairs using fun dividers. If they have a partner/friend/family member with them ask them to work with that person. **Give out** the ‘Time to Play Quiz’ and ask the pairs to complete it together. Come back as a group and **go over** the answers using a group discussion.

- How can you tell if your baby is ready to play?

**Give** the answer: Your baby will tell you that they are ready to play by becoming calm and alert, and looking at you with wide eyes and raised eyebrows. They may open their mouth into an ‘o’ shape or make noises as if to call you.

- What is the best environment for a baby to play in?

**You might need to prompt:** quiet/noisy? Busy or tranquil place? Clean and safe? TV/Radio on?

**Give** the answer: Make use of the short time your baby is fully awake and alert, turn off any distractions such as the TV or radio so that you can fully engage with each other. Even if it appears that your baby likes it, don’t put your baby in front of the TV or electronic noisy toys and flashing lights. This can over stimulate them and make it hard for them to get their all-important sleep. Also make sure your baby has a clean and safe place to play, you might use a blanket or mat for them to play on and then store it away when you have finished. You might need to make sure that you shut pets out of the room whilst your baby is playing on the floor.

- What are signs that your baby doesn’t want to play anymore?

**Give** the answer: When your baby no longer wants to play they will look away from you and maybe close their eyes. If your baby shows signs of tiredness like grizzling, crying, fist clenching, eye rubbing or yawning stop playing and work on settling your baby.

Make sure the group understands the difference between structured and unstructured play, and that babies should do both types of play each day:

- **Unstructured** play is led by your baby. It is when they engage themselves in play and are discovering the environment around them (for example laying on their tummy on a play mat). This does not mean that they play alone or are unsupervised.
- **Structured** play is planned and led by parent/carer. It is when parent and baby interact with each other using play to develop movement skills (for example playing a game like peek-a-boo in which you move their arms and hands around carefully).

TIME TO PLAY: SWATTING GAME

You will need strong string and some tin foil and a ball or two of bright coloured wool.

This game helps to develop co-ordination. Attach different objects with different textures and colours to a piece of strong string. Choose objects that are light and too big to fit inside a toilet roll so that there is no risk of your baby choking, for example; a ball of scrunched up foil, a rattle, a ball of wool. **Demonstrate** by laying Baby Sam on a clean mat/blanket on the floor, dangle the objects above the baby’s chest, just far enough from your baby so that they can touch it but not grab it. The baby will reach out and swat the object. Encourage the group to join in with their babies if they are alert and ready to play. Encourage the group to praise their babies for playing the game.

TUMMY TIME!

You will need a mat/blanket and a towel

**Remind** the group that Tummy Time helps babies learn to push up, roll over, sit up, crawl, and pull up to standing. It helps them to develop balance and strength in their neck, back and limbs.

**Explain** that the best time for Tummy Time is when the baby is awake, alert and not crying. A good time is after their bath or burping. At this very young age do Tummy time for 5-10 minutes, 2 – 4 times a day. Demonstrate by putting down a clean mat/blanket out on the floor and put Baby Sam down, lying on his/her front. Explain that the position can be uncomfortable for some babies because they are used to lying on their backs. **Demonstrate** the following to the group to show them techniques if their baby doesn’t like being on their tummy. Encourage them to join in with their baby if they are awake and engaged:

- Place a rolled up towel under their chest, keeping their arms over and in front of the towel. This balances their weight and helps them to lift their head.
- Lie on the floor in front of them so they can see your face.
- Lay them on your chest: they will enjoy being close to you.
- Place a baby-safe mirror in front of them where they can see it.

SING-ALONG AND MOVE: WIND THE BOBBIN UP (Or chose another song with actions that mums can gently move the babies’ limbs around to).

**Explain** that games where you guide a baby’s limbs gently are great for developing muscle strength, co-ordination and the connections between the brain and muscles. **Sit** on the floor with your knees bent and feet flat on the ground. Lay Baby Sam on your lap looking up at you. Invite the group to join with or without their baby as you sing ‘Wind the Bobbin Up’. Gently move Baby Sam’s arms and legs with the song.

Wind the bobbin up

Wind the bobbin up

Pull pull, clap clap clap

Wind it back again

Wind it back again

Pull pull, clap clap clap

Point to the ceiling

Point to the floor

Point to window

Point to the door

Clap your hands together 1,2,3

Put your hands upon your knee

**8.	SELF-ESTEEEM AND PERSONAL POWER	20 mins**

**Remind** the group about the discussion in session 3 of the antenatal programme.

**Display** the Influences on Behaviour board (see updated PGL Handbook page 4.4), and use it to **introduce** these linked topics. The group will look first at Self-esteem, then at Personal Power, and identify some positive thinking to cope with challenging situations and making positive choices.

SELF-ESTEEM

KEY MESSAGE: Self-esteem is the second bit of the triangle. The way we think and feel about ourselves - our self-esteem - is first shaped in childhood by the events we experience and the way we are treated.

**Display** the Cycle of Feelings and Behaviour board (see Updated PGL Handbook page 4. 7). It is also in The Parenting Puzzle (PP91). **Suggest** that the way others behave towards us affects the way we feel about ourselves and the way we in turn behave towards other people. As adults we can think more clearly about others' attitudes towards us, and perhaps challenge them, but they still influence us. And we also affect those attitudes by the way we ourselves behave. On days when we are feeling fine, we’re more likely to be helpful and kind to others - and to get a positive response from them too. **Invite** group's agreement and comments on this idea.

**Invite** the group to suggest where on the cycle change in children needs to begin. **Explore** gently the possibility that many of us think that if children would behave better, the problems we have with them would melt away. But until children feel good about themselves, it is hard for them to change, so the place in the cycle where change needs to begin is with how we and others behave towards them. **Ask** which of the Four Constructs this refers to.

Children will grow up somehow, but the conditions adults provide can make all the difference. A baby is like a tomato seed. With the right balance of nurturing: soil, light, water, nutrients - the tomato plant reaches its full potential, it is full of vigour and has large, healthy fruit. With poor care it will still grow, but with a struggle, and the result will be a puny plant that is susceptible to disease, and produces small, poor fruit. (Facilitators who are artistic can draw a strong and a weak plant on the flipchart for comparison).

With children, one of the most important ingredients in healthy growth is ensuring that we build their self-esteem. Negative messages lower self­ esteem; there are also ways of increasing it.

**Divide** the group into small groups of 3 or 4 and ask them to think about what they can be doing right now with very young babies to boost their self-esteem – positive ways of behaving. (Praise, having fun, love, consistency, empathy, playing, understanding, cuddles, kindness).

**Ask** the group to share their ideas and record on a flipchart.

Briefly **discuss** how babies and children will feel and behave if they are treated like this …. And link it back to the earlier discussion about routine and consistency.

PERSONAL POWER

KEY MESSAGE: Our Personal Power is the emotional strength we have to get our needs met, and to make choices in our lives. It isn't about being physically strong, or about having power over other people. It's our inner strength - power for ourselves, not power against others. This idea is new to many people, so will need some time to explore. The choices we make about our behaviour and our lives reflect our self-esteem through our positive or negative use of Personal Power, so the concepts are closely connected.

**Display** on the flipchart the following statements as headings:

When I feel comfortable with the power I have I also feel ……(Green)

When I feel powerless, I also feel ……(Red)

**Invite** parents, in pairs, to reflect on the feelings they experience when feeling powerless. **Ask** the group to feedback and **write up** their responses under the ‘powerless’ heading in red pen. Then invite the same pairs to reflect on the feelings they experience when comfortable with the power they have, and write up their responses under the ‘comfortable power’ heading in green pen.

**Invite** comments from the group on the strength of feelings expressed.

**Refer** to the cartoons in the Parenting Puzzle (PP86) and check with the group that the idea of Personal Power is clear to them: it is power for ourselves, not power over others.

Life with a new baby can be tough and there are likely to be some challenges to deal with at present. Let’s look at some examples of using Personal Power to ease some pressure points in life.

**Display** on the flipchart three vertical columns with the following headings and examples in the centre column:

| **Positive Choices** | **Areas in our lives we have power** | **Negative Choices** |
| --- | --- | --- |
|  | What we eat in the family |  |
|  | Disagreements and difficulties in your relationship |  |
|  | Use of leisure time |  |

**Brainstorm** with the group and see if they have other ideas to add to the central column. Further examples for new parents might include money worries, what time we go to bed, how often to feed the baby, who decides when to feed the baby, conflicts with extended family etc.

Give an example of a positive and negative use of power under the relevant headings. **Write up** the positive in green and the negative in red again.

| **Positive Choices** | **Areas in our lives we have power** | **Negative Choices** |
| --- | --- | --- |
| Mostly healthy family meals | What we eat in the family | All junk food and lots of treats |
| Discuss difficulties to find shared solutions | Disagreements and difficulties in your relationship | Arguing, shouting, silence or criticism |
| Regular physical activity | Use of leisure time | Not making time for any |

**Ask** the group to think of the positives and negatives for some of the different headings they identified.

**Check** that the group that the idea of Personal Power is becoming clearer to them.

**9.	MATERNAL DIET	10 mins**

This is a brief time to **remind** mums and parents about the importance of eating well themselves and getting back into/establishing good habits for the future – there are a couple of fun activities and some information about energy balance to introduce to (re)motivate some thinking and self-awareness about what parents are eating. It is a chance to use some of their Personal Power in a healthy way.

**Ask** the group….

How are parents getting on with eating well? Still a bit chaotic or are things settling back into a routine?

Big life changes like becoming parents often bring about changes to our eating and activity that we don’t even notice - but even small changes can make a big difference to our body weight.

Have a look at ‘The Big Balancing Act’ **hand-out 🕮** together and give to take home. There is a question on the bottom of page one that leads into a look at page 2 for the answer.

In pairs or as a big group **have a quick go** at the takeaway and calorie matching game. (See the ‘Tempted by Takeaways **hand-out 🕮**). **Remind** the group that drinks and sweet treats afterwards often add extra . The answers are at the end of the **hand-out 🕮** (although they are all similarly calorie rich and contrast drastically with the two alternatives given at the bottom).

There are 2 quick alternative ideas to try too instead of a takeaway. A recipe for the stir fry is also given.

**Remind** them to add any ingredients they would need to their shopping list in advance if they want to try it.

If in the busyness of recent weeks (when all the focus is on the baby’s needs), takeaways, sugary drinks and snacks high in fat and sugar have become a more regular feature of life – perhaps just because there hasn’t been time to think of alternatives -now may be the time to think about limiting them – **remind** the group again about the **hand-outs 🕮** from the antenatal sessions (which were given out again in the last session).

Session 2: ‘Smart Snacks’

Session 3: ‘Healthy Eating Choices’

Also highlight the website links given on the Tempted by Takeaways **hand-out 🕮** - http://www.nhs.uk/Change4Life and select the ‘recipe finder’ section or

http://www.nhs.uk/Livewell/men1839/Pages/Dodgytakeaways.aspx for a discussion on how to make takeaways healthy.

These links are to NHS websites with lots of recipe ideas for healthy meals. (Practitioners may want to check them out first and perhaps print out a few as examples).

**10.	TIME TO HAVE A GO	5 mins**

Display on a board the appropriate Time To Have a Go suggestions for the week:

- Don’t forget to boost your own self esteem with self-praise and nurturing yourself.
- Do the physical activity that you chose for yourself during the mingle exercise.
- Read the **hand-out 🕮** ‘Start 4 Life – Introducing Solid Foods’.
- Be alert to the correct signs that your baby is ready for weaning.
- Is there anything about your own eating patterns that could be healthier or that have become unhealthier since having your baby -why is this and what might help? Identify one or a few swaps or changes to make a start on getting it back on track. .Be realistic though!

Some ideas:

Healthier snack or drink choices

Takeaways - cut down - try the recipe in the **hand-out 🕮**!

Fruit and veg -eat more

Get back to regular meals including breakfast

Do you need to talk with your partner or family about any of this -perhaps working out a way to find time to getting food organised e.g. shopping and /or cooking?

**11.	WHAT DID YOU THINK OF TODAY? (FEEDBACK)	5 mins**

**Give out** weekly feedback forms. **Play** relaxing background music whilst forms are being completed; place a container on the floor/table to collect forms. **Thank** the group for completing them. If there are literacy issues the whole group could respond to questions on flipchart and facilitator can record responses.

**12.	GOODBYE CIRCLE	5 mins**

**Stand** in a circle and say what a good session it has been.

**Encourage** everyone to say goodbye by touching the foot of the next person and leaving it touching until the feet form a circle on the floor.

Optional – Give out a small gift such as a glass bead or key ring as a tangible reminder of the session.

**Close** the session by saying how much you have enjoyed it and that you are looking forward to seeing everyone again at the next session and remind them of the date.

**WELCOME TO THE WORLD!**

**Postnatal Session Three (babies aged approx. 12 weeks)**

**STEP BY STEP**

PLAN FOR TODAY

|  | **ACTIVITY** | **TIME** | **MATERIALS NEEDED** |
| --- | --- | --- | --- |
| 1 | Welcome | 5 mins | Refreshments, music and player, ‘Welcome’ sign, attractive table cloth, flowers, badges, stickers, felt-tip pens, plan for today, attendance sheet, reward system, etc |
| 2 | Icebreaker: Right Now I am Feeling… | 10 mins | Flipchart |
| 3 | Family Feedback | 10 mins | Flipchart |
| 4 | Feeding | 30 mins | Flipchart  Hand-outs – ‘How to Start Weaning – Practical Guide’, ‘Weaning – What and When’, ‘Dairy Council Menu Planner Stage 1’, ‘Food Photographs’, ‘Meal Planner for Weaning’, others for reference. |
| 5 | Mums’ Physical Activity | 20 mins | Flipchart,  Board – The Nurturing Wheel  Hand-out – Pram Pedometer Challenge  Pedometers |
|  | *TEA & COFFEE BREAK* | *10 mins* | *Refreshments* |
| 6 | Infant Play & Time to Play | 20 mins | Baby Sam, baby toys  Hand-outs – ‘Finding Time to Play’, ‘Being Sedentary: Activities and Questions’, ‘Being Sedentary: Answers’ |
| 7 | Ages & Stages Quiz | 15 mins | Hand-outs - ‘Happy Steps in Growing’, ‘Happy Steps Quiz’, ‘Happy Steps Answers’ |
| 8 | Feelings…and what we do with them | 10 mins | Flipchart, Board – What we do with difficult feelings |
| 9 | Relaxation Exercise: the Hot Air Balloon | 5 mins | Hand-out – Relaxation Exercise |
| 10 | Time to Have a Go | 5 mins | Hand-out |
| 11 | What did you think of today? (Feedback) | 5 mins | Hand-out |
| 12 | Goodbye Circle | 5 mins | Parting gifts |
|  |  |  |  |

150 mins

**WELCOME TO THE WORLD**

**HAPPY Postnatal Programme**

**Session Three (babies aged approx. 12 weeks)**

**FEEDING AND FEELINGS**

**STEP BY STEP**

**1.	WELCOME	5 mins**

**Display** board with ‘Welcome to HAPPY!’

**Greet** group members warmly as they arrive, have background music playing, and offer refreshments. **Ask** parents to make name badges, and to sign the attendance sheet.

**2.	ICEBREAKER: RIGHT NOW I AM FEELING…	10 mins**

**Display** on the flipchart:	‘Right now I am feeling ……’

‘I cope best when I am feeling ….’

**Explain** that today’s session will be a busy one looking at introducing solids to the baby and having a go at some more play activities but will end with a reminder of how to manage our feelings and be sensitive to the others.

**3.	FAMILY FEEDBACK	10 mins**

**Comment** briefly on last session’s feedback forms. Display the ‘Time to Have a Go’ from the last session and invite brief feedback.

- Don’t forget to boost your own self esteem with self-praise and nurturing yourself.
- Do the physical activity that you chose for yourself during the mingle exercise.
- Read the **hand-out 🕮** ‘Start 4 Life – Introducing solid foods
- Be alert to the correct signs that your baby is ready for weaning.
- Is there anything about your own eating patterns that could be healthier or that have become unhealthier since having your baby -why is this and what might help? Identify one or a few swaps or changes to make a start on getting it back on track. .Be realistic though!

Some ideas:

Healthier snack or drink choices

Takeaways - cut down - try the recipe in the **hand-out 🕮**!

Fruit and veg -eat more

Get back to regular meals including breakfast

Do you need to talk with your partner or family about any of this -perhaps working out a way to find time to getting food organised e.g. shopping and /or cooking?

**4.	FEEDING	30 mins**

**Note for group leaders:** It is recommended that weaning should start at 6 months of age and not before 17 weeks (4 months) – the closer to 6 months the better.

In terms of obesity prevention, it is felt that this delay in introducing solids will result in a reduced energy dense diet and will also reduce the opportunity for the early introduction of food high in fat and sugar.

There is good evidence that delaying weaning is beneficial in reducing obesity. Rates of obesity are higher in babies who have been weaned before they are 17 weeks old. So timing of weaning is important, as is the sort of foods that are introduced at weaning.

The choice of foods given at weaning in general is poor, often excessively high in foods high in fat and sugar with inadequate amounts of fruit and vegetables. Within the UK surveys show that many infants rarely eat fruit and vegetables, and that foods high in fat and sugar make a significant contribution to the overall diet.

However, as the practice of early weaning has been a constant issue even when the recommended time to begin was between 4-6 months, it is felt that many parents will need to be convinced of the recommendations.

The following activities provide information and guidance on infant feeding with the aim of establishing healthy eating habits and thereby reducing the risk of obesity.

The aim of this session is to introduce people to the key information resources but you won’t have time to go through all of them in detail.

HOW IS FEEDING GOING?	**5 mins**

**Check** with the group if they have any particular concerns or questions regarding feeding their baby at present. **Respond** to their questions based on the information in the **hand-outs 🕮** and booklets used in previous sessions.

GROUP ACTIVITY –STARTING WEANING	**10 mins**

**Divide** the group into small groups of 2 or 3 using fun dividers. **Ask** each group to discuss with each other the following questions written up on a flipchart:

When do you plan to start weaning your baby?

What sort of equipment will you require?

How will you start weaning your baby and what will you feed him/her?

**Share out** the following resources:

- Start 4 Life – Introducing Solid foods
- ‘How to Start Weaning – A Practical Guide’ **hand-out 🕮**
- ‘Weaning – What and When’ **hand-out 🕮**
- Dairy Council Menu Planner Stage 1 **hand-out 🕮**
- Food Photographs **hand-outs 🕮**

**Invite** feedback from the group to the questions and ensure that the information given is consistent with the guidelines in the **hand-outs 🕮** and booklets.

Prompt with some additional questions if you have time:

What time of day is best for the first feed?

How much should you offer at first?

Does a big baby need to be weaned earlier?

Do you need to wean if you are going back to work?

GROUP ACTIVITY – PLANNING AHEAD	**15 mins**

Using all the resources provided for this activity, **ask** the groups to write a feeding plan for 3 whole days and specify when, what and how much they will feed their baby over the 3 days. Assume that babies are on three meals a day of the Dairy Council Menu Planner.

In the meal planning exercise think about a variety of tastes, colours and quantities i.e. how much will be offered? Give out the ‘Meal Planner for Weaning’ **hand-out 🕮** which uses the following format:

|  | Day 1 | Day 2 | Day3 |
| --- | --- | --- | --- |
| Breakfast |  |  |  |
| Mid-morning |  |  |  |
| Midday |  |  |  |
| Afternoon |  |  |  |
| Early evening |  |  |  |
| Bedtime |  |  |  |

**Obtain feedback** from each group.

**Prompt** with more questions or comment if you have time.

Should you add salt or sugar when preparing food for baby?

What if you don’t like vegetables?

What if baby spits the food out?

What drinks should you be offering?

Should you be using tins, jars and packets or making my own?

Should you be giving snacks?

The answers to these questions are all in the booklets and **hand-outs 🕮** used earlier in the session. **Ensure** that parents have all the resources and encourage them to look at them at home as they plan when and how to wean their baby.

**5.	MUMS’ PHYSICAL ACTIVITY	20 mins**

IN PAIRS

**Ask** the group to recall the activity that they chose for themselves last session. Divide the group into pairs using group dividers and **discuss** what activity they did or didn’t do and what helped them or stopped them from doing their activity. **Explain** that it is not good or bad to have done or not done their activity; ask them just to notice why they did or did not do it.

**Give prompts**: did you think about doing your activity? Did something get in the way? Did you do your activity with your partner or a friend?

FLIP CHART

In the group **ask** people to share the barriers that came up for them around doing their activity: what stopped them from doing it? **Write** them up on the flip chart. **Ask** the group to think of ways they could overcome that barrier in the future and **write** it up on the flip chart.

**Remind** mums that it is important that they make time for physical activity for themselves as a way of nurturing and taking care of themselves. **Remind** them of the nurturing wheel in session 4 of the ante-natal programme.

INTRODUCE THE PRAM PEDOMETER CHALLENGE

The Pram Pedometer Challenge is a fun way for us to work together as a group to be more active. You will each get a pedometer which will count the number of steps that you take when you are wearing it - you can wear it when you are doing physical activities like taking your baby out in the pram for a walk, or at a zumba class, and for everyday activities. As a group we will chose our imaginary target destination. There are three destinations to choose from: bronze, silver or gold. At each of the remaining sessions we will add up the group’s total number of steps, convert them to miles and see how close we’re getting to our goal. You can earn extra miles by doing additional activities each week which I’ll tell you about. For example: this session you will earn 50 extra miles if you teach your family games to play with your baby. You should not wear your pedometer if you go swimming as it’s not waterproof. You can write down your steps each day on your personal record sheet which you will be given today. At each HAPPY session we will add the miles in the group up and see where we are at with reaching our target. If we meet our target there will be a reward for the group at the end!

**Make sure** that everyone understands the challenge and then as a group decide where your destination will be.

The possible destinations are…

Bronze: London which is 200 miles

Silver: Paris which is 500 miles

Gold: Barcelona which is 700 miles

**Ensure** that everyone is involved with the decision and that everyone (as much as possible) is willing to take on the challenge and thinks the goal is achievable.

**Write up** the target (Bronze, Silver, Gold), destination and total number of miles the group will need to travel on the group Pram Pedometer Challenge Record flip chart. **Give out** individual record sheets – ‘Pram Pedometer Challenge’ **hand-out 🕮** - and a pedometer to each person in the group. **Explain** how to use the pedometer and complete the record sheet.

As a group **discuss** and **set** the group goal for next session. **Ask** each member to set their own individual target goal and write it on their record sheet

**Tea and Coffee Break	10 mins**

**6.	INFANT PLAY	& TIME TO PLAY	20 mins**

DISCUSSION

**Facilitate** the group discussing types of play they have been doing with their baby. **Ask** the group to share what games their babies enjoy playing and let them trade ideas, demonstrating if possible. **Ask** the group if their babies have been having tummy time. **Suggest** using a rolled up towel to make tummy time more comfortable if their baby is struggling. **Tell** the group that tummy time should now be for 10 - 15 minutes, 2 – 3 times a day. **Ask** the group to share other problems that they have come up against when playing with their baby and encourage the rest of the group to share ways to overcome the problem. Ensure that this discussion covers the barrier of not having enough time to play with the baby and that one way to overcome this is to encourage other family/household members to spend time playing with the baby. As other family/household members become familiar and confident in playing with the baby, mum will have more time to do other things.

CASE STUDY

**Read** out the case study **hand-out 🕮** ‘Finding Time to Play’ and **ask** the group to come up with solutions for this mum who is juggling many different priorities so that she can find time to play with her baby. **Prompt** the group to write down the solutions as a reminder for them to take home.

BEING SEDENTARY

**Ask** the group to complete the Being Sedentary **hand-out 🕮**. Look at each activity/question in turn and answer them as a group as you go along. Start with activity 1. Once you are all finished with activity 1 as a group talk through the answer before you move onto the next question/activity.

TIME TO PLAY

**Reassure** mums that it’s okay if their baby is asleep or doesn’t want to play; they can try the games when they get home and their baby is ready to play. **Remind** the group that it is important to remember that each baby is unique and will develop and reach milestones at different rates and times. If their baby isn't quite ready for the different play activities, don't worry — just try them again a few weeks later.

PULL ME UP!

This game will help to develop strength in your baby’s core muscles (their stomach and back). When your baby is lying on the floor on a clean mat/blanket, gently take hold of their wrists and pull them gently up to sitting. Then lower them back down. At first you may need to support their head and shoulders. You can make noises as you pull them up and lower them down to make the game fun and engaging: this will also help your baby to develop communication skills. Demonstrate the game with baby Sam and encourage mums, dads and other family members to join in with the game.

TOYS

Now that your baby can see further they will be more interested in the things around them and may start to show an interest in toys. Place your baby on their tummy and place some toys so that they have to reach out for them. The best toys are ones that make gentle sounds and/or have interesting textures and shapes. It is very important that toys are safe for your baby so make sure that there are no small choking hazards. Now try placing your baby on their back and do the same thing.

SING-ALONG AND MOVE: ‘Row row row your boat’ (or chose another song with actions that mums can gently move the babies’ limbs around to). Explain that games where you guide a baby’s limbs gently are great for developing muscle strength, co-ordination and the connections between the brain and muscles. Sit on the floor with your knees bent and feet flat on the ground, lay Baby Sam on your lap looking up at you. Invite the group to join with or without their baby as you sing ‘row row row your boat’, gently move Baby Sam backwards and forwards and side-to-side along with the words of the song:

Row row row your boat gently down the stream

merrily merrily merrily merrily life is but a dream

Rock rock rock your boat gently to and fro

if you see a waterfall over the edge you go

Row row row your boat gently down the stream

If you see a crocodile don’t forget to scream

**7.	AGES AND STAGES QUIZ	15 mins**

KEY MESSAGE:

Many of us worry about whether our children are developing ‘normally’. It can be hard to know what is normal and what isn’t. Also children, even in the same family, develop at different rates in different ways, so just because the first child started doing something at a certain age, doesn’t mean the second one will too.

We may tend to compare babies and children, and become anxious if another child is doing better than ours. This can lead to rivalry between children and adults and creates stress where there doesn’t need to be any.

Being aware of what is reasonable to expect from our babies as they grow up will reduce our frustration and anxiety, and consequently create happier, more contented children.

A child who is able to do more than we allow will feel held back, will be frustrated, rebellious and difficult.

A child who is expected to do more than he or she is ready for will probably be difficult too and may feel inadequate.

**Introduce** the topic briefly using ideas in the key message.

**Invite** the group to complete a short quiz on their own, or in pairs if they want to discuss it.

**Pass round** the HAPPY Steps in Growing **hand-out 🕮** and the HAPPY Steps in Growing Quiz **hand-out** **🕮.** Ask people to have a go at answering the questions using the **hand-out 🕮** as a prompt. Re-assure the group that this is not a test, and we will go through the questions together.

Give them about 5 minutes to think about a few of the answers.

**Share** the HAPPY Steps in Growing Quiz answers **hand-out 🕮** with the group to compare answers and gain reassurance about what to expect and when.

**8.	FEELINGS…AND WHAT WE DO WITH THEM	10 mins**

KEY MESSAGE:

As we discussed in the antenatal programme: ‘all behaviour has a reason and is driven by a feeling’.

Being a parent of a young baby brings up a wide range of emotions from excitement, pride and joy to exhaustion, worry, and frustration. This will be felt by mothers, fathers and others who have contact with the baby and family at this time.

All feelings are valid and they contain important information about what is happening for us. Everyone needs help to identify and understand feelings, to find ways of expressing them honestly and healthily and to respect the feelings of others.

This takes practice, but being able to listen to our feelings and understand what is driving them is a valuable skill.

**Remind** people of the exercises in the antenatal programme of naming feelings, of suppressing feelings on slips of paper under our feet and of dealing with stress.

**Display** the ‘What we do With Difficult Feelings’ Board and remind people of the relevant section in the Parenting Puzzle (PP112,113).

There are different ways to respond to difficult feelings and we can use our Personal Power to make choices about healthy or unhealthy ways to respond to difficult feelings.

**Invite** the group to contribute ideas to a prepared flipchart:

| **What to do with difficult feelings** | |
| --- | --- |
| Unhealthy responses (Red) | Healthy responses (Green) |
| (*e.g.:* *aggression, shouting, blaming, hiding away, comfort eating)* | (*e.g.:* *talking, letting go, relaxing, thinking, taking exercise)* |

Start with the unhealthy responses, so that we end with the healthy/positive options of responding to difficult feelings. **Ask** the group to identify one thing that they could do more of to deal with difficult feelings.

**9.	RELAXATION EXERCISE:	5 mins**

**RELEASING FEELINGS – THE HOT AIR BALLOON**

**Invite** the group to take part in a relaxing visualisation exercise that helps to release feelings. **Check** that everyone can picture a hot air balloon and ensure everyone is sitting comfortably. **Remind** the group that they are to put their troublesome feelings and not themselves into the basket, and that at any time during the visualisation they can stop. **Suggest** that they lower their gaze to the floor to avoid making eye contact with others. **Read** the following script in a soft, slow voice, with five second pauses.

*Start to relax by taking some slow, easy breaths …. And now begin to picture a brightly coloured hot air balloon, hovering ready to lift its basket off the ground and up into the sky. (Pause)*

*When the balloon and basket are clear in your mind, take your troublesome feelings and put them into the basket. Let them take up as much space as they need – there’s plenty of room. (Pause)*

*Now picture the balloon slowly lifting the basket off the ground: gradually it rises up and up until it is outlined against the clear blue sky …. keep watching as it rises into the air, getting higher and higher, until it begins to seems smaller as the breeze blows it gently away from you, carrying away the troublesome feelings. (Pause)*

*Where these old feelings were inside you, imagine a new sense of lightness, or space, or peace …. And as you watch the balloon drift away until it is only a speck in the distance and becomes part of the blue sky, let yourself be filled with this lightness, space and peace ….*

*Then, when you are ready, and remembering that you can return to feel this new peace whenever you want to, come slowly back to where you are here, and now.*

**Allow** the group to refocus, then ask them to wiggle their fingers and toes to reconnect with reality.

**Mention** that this relaxation will be given out as a **hand-out 🕮** for them to read again at home and to share with other family members, and possibly their baby as it grows up.

**10.	TIME TO HAVE A GO	5 mins**

- Refer to the **hand-out 🕮** ‘How to Start Weaning – Practical Guide’ for a summary of weaning guidance.
- Refer to the **hand-out 🕮** ‘Weaning: What and When’ for guidance on specific foods to offer.
- Refer to the **hand-out 🕮** ‘Start for Life: Introducing Solid Foods: Giving Your Baby a Better Start in Life’.
- Have a look through the ‘HAPPY Steps to Growing’ **hand-out 🕮** and discuss it with others in the family to share key information.
- Notice your feelings, and try to sense how others in the family will be feeling too
- Take the time to teach games to each family/household member and encourage each person to play the games with the baby at least once before the next session. For each person that plays with the baby the group will earn **50** extra miles towards the Pram Challenge goal. Take some photographs of the games you are playing with your baby and put them in your Memory Book.
- Complete the target that you set for yourself for the Pram Challenge.
- Write a list of quick and easy light and main meals you can make, perhaps using the things in your cupboard or fridge and pin it on the wall to inspire you when writing a shopping list or planning meals for the next few days.
- Have a look again at the ‘Big Energy Balance’ **hand-out 🕮** from session 2. Think about your everyday activity since having your baby –how do you think your activity and eating has changed (if at all) since having your baby compared to before? You have had a massive life change and that often changes how we eat or how active we are –it could have changed in any direction! Is there anything you will need to think about adjusting now or when things settle –make a note in your memory book.

**11.	WHAT DID YOU THINK OF TODAY? (FEEDBACK)	5 mins**

**Give out** weekly feedback forms. **Play** relaxing background music whilst forms are being completed; place a container on the floor/table to collect forms. **Thank** the group for completing them. If there are literacy issues the whole group could respond to questions on flipchart and facilitator can record responses.

**12.	GOODBYE CIRCLE	5 mins**

**Stand** in a circle and say what a good session it has been.

Encourage everyone to say give a gentle touch by placing their hand on the to the person next to them and saying goodbye.

Optional – Give out a small gift such as a laminated copy of the Relaxation Exercise as a tangible reminder of the session.

**Close** the session by saying how much you have enjoyed it and that you are looking forward to seeing everyone again at the next session and remind them of the date.

**WELCOME TO THE WORLD!**

**Postnatal Session Four (babies aged approx. 5 months)**

**STEP BY STEP**

PLAN FOR TODAY

|  | **ACTIVITY** | **TIME** | **MATERIALS NEEDED** |
| --- | --- | --- | --- |
| 1 | Welcome | 5 mins | Refreshments, music and player, ‘Welcome’ sign, attractive table cloth, flowers, badges, stickers, felt-tip pens, plan for today, attendance sheet, reward system, etc |
| 2 | Icebreaker: Singing Games | 5 mins |  |
| 3 | Family Feedback | 10 mins | Flipchart |
| 4 | Use of Family Rules | 15 mins | Flipchart |
| 5 | Infant Play | 20 mins | Flipchart, felt-tip pens,  Hand-out – ‘Now that I can crawl’ |
| 6 | Time to Play | 10 mins | Baby Sam |
| 7 | Mums’ Physical Activity | 5 mins | Pram Challenge slip |
|  |  |  |  |
|  | *TEA & COFFEE BREAK* | *10 mins* | *Refreshments* |
| 7 | Mum’s Physical Activity contd. | 5 mins | Pram Challenge map |
| 8 | Families and Food | 45 mins | Flipchart paper, pens.  Hand-outs – ‘You are Being Watched’, ‘Family Food – The HAPPY Guide to getting organised’, ‘HAPPY Shopping List’, ‘HAPPY Meal Planner’,  Board – 4 Constructs, ‘Making Meals Healthier’ |
| 9 | Time to Have a Go | 5 mins | Hand-out – ‘Time to Have a Go’ |
| 10 | What did you think of today? (Feedback) | 5 mins | Hand-out – ‘Weekly Feedback’ |
| 11 | Goodbye Circle | 5 mins | Parting gift |
|  |  |  |  |

150 mins

**WELCOME TO THE WORLD**

**HAPPY Postnatal Programme**

**Session Four (babies aged approx. 5 months)**

**FAMILIES AND FOOD**

**STEP BY STEP**

**1.	WELCOME	5 mins**

**Display** board with ‘Welcome to HAPPY!’

**Greet** group members warmly as they arrive, have background music playing, and offer refreshments. **Ask** parents to make name badges, and to sign the attendance sheet.

**2.	ICEBREAKER: Singing Games	5 mins**

**Invite** parents to join you in a couple of singing and activity games to play with children, for example: ‘Row, row, row your boat’; ‘Round and round the garden’; ‘Twinkle, twinkle little star’.

**3.	FAMILY FEEDBACK	10 mins**

**Comment** briefly on last session’s feedback forms. Display the ‘Time to Have a Go’ from the last session and invite brief feedback:

- Refer to the **hand-out 🕮** ‘How to Start Weaning – Practical Guide’ for a summary of weaning guidance.
- Refer to the **hand-out 🕮** ‘Weaning – What and When’ for guidance on specific foods to offer.
- Refer to the **hand-out 🕮** ‘Start for Life: Introducing Solid Foods: Giving Your Baby a Better Start in Life’.
- Have a look through the ‘HAPPY Steps to Growing’ **hand-out 🕮** and discuss it with others in the family to share key information.
- Notice your feelings, and try to sense how others in the family will be feeling too.
- Take the time to teach games to each family/household member and encourage each person to play the games with the baby at least once before the next session. For each person that plays with the baby the group will earn X extra miles towards the Pram Challenge goal. Take some photographs of the games you are playing with your baby and put them in your Memory Book.
- Complete the target that you set for yourself for the Pram Challenge.
- Write a list of quick and easy light and main meals you can make, perhaps using the things in your cupboard or fridge and pin it on the wall to inspire you when writing a shopping list or planning meals for the next few days.
- Have a look again at the ‘Big Energy Balance’ **hand-out 🕮** from session 2. Think about your everyday activity since having your baby –how do you think your activity and eating has changed (if at all) since having your baby compared to before? You have had a massive life change and that often changes how we eat or how active we are –it could have changed in any direction! Is there anything you will need to think about adjusting now or when things settle –make a note in your memory book.

GROUP ACTIVITY: HOW IS FEEDING GOING? ANY CONCERNS?

Parents will need to be assured that it is ok to wait until their baby is ready to wean and the following signs do not necessarily mean that their baby is hungry.

Some signs mistaken for a baby being ready for solid foods

- Chewing fists
- Waking in the night when they have previously slept through
- Wanting extra milk feeds

These are normal baby behaviours and not necessarily a sign of hunger, nor being ready to start solid food. Starting solid foods will not make them any more likely to sleep through. Sometimes a little extra milk will help until they are ready for food.

Remind the group of the 3 key signs for being ready for weaning

- Staying in a sitting position and holding their head steady.
- Co-ordinating their eyes, hand and mouth so that they can look at the food, pick it up and put it in their mouth all by themselves.
- Swallowing food. Babies who are not ready will push their food back out, so they get more around their face than they do in their mouths!
- It’s rare for these signs to appear together before 6 months.

**4.	USE OF FAMILY RULES	15 mins**

For today’s session we’re going to think more about meeting the needs of the growing babies and of developing some structures and routines into family life. It’s important that we consider how to make plans and decisions as a family.

**Re-visit** family rules from Antenatal Session 1. **Ask** parents to consider family rules and how they have changed/what needs changing to adapt to their new situation. **Invite** brief discussion on what rules people think are important for creating calm, happy family life.

**Display** on the flipchart the following heading:

Rules for calm, happy family life

And on the bottom half of the sheet:

For Children	For Adults

**Suggest** an example for each heading. **Invite** contributions and write on flipchart. **Process** similarities and differences.

**Suggest** that under the ‘For Adults’ heading they include their own rules as a couple, spoken or unspoken: e.g. not criticising each other’s parenting; household chores allocation etc. Under the ‘For Children’ heading they may include having stories before bed time, or having some fun and play time with both parents.

This can be a time to think of some healthy family rules such as all eating meals together, doing a physical family activity once a week such as swimming or playing in the park, or only having sweets on Sundays.

**Acknowledge** that it can be difficult to introduce new ideas or rules into your family, so what tactics can we use? **Invite** a brief discussion about options for making changes: e.g.: talking quietly with partner once children are in bed, having a regular family meeting, getting one member of the family on board to help encourage other family members to make changes, using ‘I Statements’ from session 5 of the antenatal programme and Parenting Puzzle Pg 120.

**Invite** parents to make a commitment to developing some family rules during the next 2 months before the group meets again. **Suggest** they write them in their Memory Book and note what differences it makes to their family life.

**5.	INFANT PLAY	20 mins**

**Remind** the group that babies and children copy what their parents do. If children belong to families who do physical activity together they are more likely to enjoy being active and more likely to grow up to be fit and healthy.

**Flip chart**: **write** the heading **‘being active as a family’** and then write the subheadings: ‘**Benefits’** and ‘**Barriers’**.

**Ask** the group to give benefits and barriers (i.e. what gets in their way of being active as a family). By now the group should be very familiar with these types of lists and so shouldn’t need too much prompting.

Start with the **barriers** which should be written in red pen and are likely to include:

- Not having time
- Don’t know anywhere to go/no-where to go nearby
- Don’t have family nearby

Make sure the **benefits** list includes:

- Having fun together
- Bonding as a family
- Being a fit and healthy family
- De-stressing, relaxing and unwinding together

**Ask** the group to share solutions to overcoming the barriers, e.g. plan a time together in advance; ask friends where they go; ask Children’s Centre staff where they can recommend; ask a friend to go with you or someone from the HAPPY group. **Ask** the group members to plan an activity hour for their family to do together. You might need to give them some prompts, e.g. taking a walk in the park; going to the play park; if their babies can hold their heads up by now they can safely go in a baby swing and be pushed gently; or perhaps going to the local swimming pool. **Give** the group some felt-tip pens so that they can draw a picture of how the activity looks.

Who is there?

Where is it?

What are they doing?

Here’s an **example** of a drawing:

**Ask** them to decide on a date and a time that they will do it and write it on the picture. **Encourage** them to display their picture at home, on the fridge or noticeboard to remind them to do the family activity.

**Crawling and safety**

**Tell** the group that now their babies are moving more and perhaps rolling over, soon - usually between 7 and 10 months - they will start to crawl.

**Explain** that even though they may think it is best to restrict a baby’s movement to keep them safe, this can be very damaging for their health. Baby equipment like a pushchair, car seat, rocker, or swing is very restrictive and can affect the way that the muscles develop. Spending more than 2 hours per day in equipment has been linked with a delay in the development of movement skills such as rolling, crawling and walking.

Babies’ brains are developing rapidly so they need to play actively even though the constant supervision that they need may be tiring. Try to rest when they rest. Avoid using TV or DVDs as a baby sitter. Television is not recommended for children under 2 years old. You can use a safety gate or play pen to keep your baby safe and allow them to interact with you when you are in the kitchen cooking, or in the bathroom showering. You can keep a few special toys for them to play with at these times

**Give out** the Child Accident Protection Trust ‘Now that I Can Crawl’ booklet and explain that it gives information about how to make the home a safe place for babies and children to play and live in. **Give** the group a couple of minutes to flick though the booklet. **Encourage** them to spend longer looking though it at home and make any changes that they need to their home environment.

**6.	TIME TO PLAY	10 mins**

**Reassure** mums that it’s okay if their baby is asleep or doesn’t want to play; they can try the games when they get home and their baby is ready to play. **Remind** the group that it is important to remember that each baby is unique and will develop and reach milestones at different rates and times. If their baby isn't quite ready for the different play activities, don't worry — just try them again a few weeks later.

**Up, Up and Away!**

By around 5 months babies can usually hold their heads up. Now you can play together pretending that your baby is flying! This will help your baby to develop strength in their core muscles, their stomach and back.

**Rocket ship**: sit on the floor with your baby facing you and take hold of them underneath their armpits. Pretend you are the rocket commander. You can say ‘baby, prepare for take-off’… build up your babies’ anticipation using noises and then lift them into the air with a Whoosh! Bring them back down gently to land on the floor. You can also turn your baby to face outwards so that they get a different view on take-off!

**Lift (elevator)**: sit on the floor with your baby facing you and take hold of them underneath their armpits. Pretend you are the lift. Lift the baby up in stages, count up floor by floor and pause at each floor. Then sink your baby quickly back down and as they come back to the floor say ‘Ding!’

You could also play at being an aeroplane flying off to your favourite holiday destination, or a hot air balloon, or a helicopter pilot!

Demonstrate the game with baby Sam and encourage the group to join in with the game with their babies if their baby is awake, alert and ready to play.

**Sing-along and move**: your baby may be beginning to enjoy bath-time more now. Teach the group the bath-time song and encourage them to sing along mimicking with their baby splashing in the bath and then at the end of the song they can give their baby a big hug! Encourage the group to sing the song during bath-time at home where their baby can splash in the water.

Rub a dub dub

Rub a dub dub

Taking a bath in the tub

Rub a dub dub

Rub a dub dub

Finding the dirt then we scrub

Snug a lug lug

Snug a lug li

Take that towel

And dry, dry, dry

Snug a lug li

Snug a lug lug

Wrap it up with a great big hug!

**7.	MUM’S PHYSICAL ACTIVITY	5 mins**

**Ask** the group to write + 50 miles on their pram pedometer step record if their families played any time to play games with their baby over the last month. **Ask** each member of the group to fold their step record in half so that no one else can see it and pass it into you.

The practitioner totals the group activity during the break. The scoring system is 2000 steps = 1 mile, remember to add on the additional bonus miles.

**Tea and Coffee Break	10 mins**

**7.	MUM’S PHYSICAL ACTIVITY CONTINUED	5 mins**

**Ask** the group to reflect silently and think about what they did or didn’t do towards the challenge. Ask them to come up with a rating for themselves based on what they did or didn’t do and share that rating with the group. Ask if anyone wants to share what they did or didn’t do toward the Challenge and why they have given themselves the rating. Ask the group to give the people who share positive feedback, praise or encouragement.

**Reveal** to the group how many miles they have travelled and remind them about how far they have to go to reach their target. Encourage everyone to clap and cheer!

**Ask** the group to decide on a goal for the next session, bearing in mind what they achieved this time. **Write up** the target on a flip chart.

**8.	FAMILIES AND FOOD	45 mins**

**Note for group leaders**: the aim of this section is to look at the influences parents have on their children’s eating and attitudes to food and health –and then explore what parents need to think about or do to give their child a good chance of building healthy eating habits and attitudes.

From this we look at some practical skills that parents have or can develop to help them in the months and years ahead. Some group members will be highly skilled in or even just natural at some things e.g. meal planning, modelling healthy eating practices, cooking, food budgeting, and others may not be – and you can harness this to enable people to learn from each other as well as from the ideas and resources suggested. Before the session make sure you are familiar with the content of the **hand-outs 🕮** – there isn’t time to cover it all in the session but you will be able to draw on it and show them what is there –or talk about one aspect if the group is particularly keen on that –and it may be useful in future weeks.

GROUP ACTIVITY: ‘WHAT THEY SEE IS WHAT YOU GET…’

You will need flip-chart paper, coloured pens and floor/wall-space.

**Put** the following questions onto flip chart paper and **ask** the group (or sub-divide into smaller groups) to come up with as many ideas as they can in a couple of minutes –use a timer if you want to!

They can write or draw or call out to a scribe. **Ask** the group to think about now and into the future when their children are older.

**Q1.	How do babies and children learn about food and eating?**

**Consider** both good habits and bad habits.

**Anticipate** answers such as - What they see, What they hear, What they taste, Food replacing love or attention, Food as a pacifier, Family food ‘rules’ and habits ,Family perceptions of weight and health

Now **show** the group the second question –and do the same thing again, i.e. get as many ideas down as possible in a couple of minutes.

**Q2.	How do/can parents have a positive influence on their children’s tastes, eating habits and attitudes to food and health?**

When you have the two lists, see if anyone wants to **add** anything to either. The key points to draw out are that children learn

- because of how and what we feed them AND
- by seeing and hearing what is going on around them.

**Relate** this to the 4 Constructs and the parenting skills we’ve been looking at – appropriate expectations, positive discipline, praise, appropriate use of rewards, personal power, family rules.

It’s often quite a surprise the first time a parent witnesses a baby/toddler do something they would prefer they didn’t and then realise how they got the idea in the first place!!

**You are being watched…. Food for thought indeed!**

Share with the group the **hand-out 🕮** – ‘You are being watched - How to have a positive influence on children’s tastes, eating habits and attitudes to food and health’.

FEEDING THE FAMILY

As well as modelling the behaviours we want to encourage, it’s clear that the daily task of providing regular healthy meals that are suitable for all the family is a big part of parenting – this takes some planning, organisation and skills….and new parents vary in their experience and confidence.

If money is tight –the more food management and cooking skills parents have –the better they will be able to manage.

GROUP ACTIVITY –FEEDING A FAMILY – WHAT DOES IT TAKE?

**Explore** what information, knowledge, skills and experience you have within your group and where the gaps are.

**Prepare** the following list on flip-chart and get group members to talk about them.

FEEDING A FAMILY –WHAT DOES IT TAKE?

- Planning and shopping
- Knowing what to serve/Ideas for different kinds of meals/family meals that suit everyone
- Knowing how much food to serve –portion sizes
- Understanding food labels
- Food & family budgeting
- Cooking skills
- Adapting familiar or traditional recipes to make them healthier
- Finding time to do all this
- Knowing how to use leftovers
- Knowing how to store food safely

**Ask** the group to identify which issues they think are the most important to them and mark on the flipchart with a star, then ask the group to identify which skills that they don’t feel confident in at the moment by marking the flipchart with a question mark.

Having identified the issues which people think are most important or where they have gaps in knowledge or skills, **pick** 4 or 5 issues to focus on for a problem solving discussion.

**Write** these 4 or 5 key issues at the top of a sheet of flipchart, one on each sheet and place the sheets around the room.

This is designed as a moving exercise, with people spending a short amount of time at each sheet and writing down as many possible ideas or solutions to helping address the issue identified at the top of the sheet. **Emphasise** that different solutions will work for different people or at different times. What we want is as many ideas as possible for people to pick from.

**Keep people moving** from sheet to sheet so that all the parents have had a chance to contribute their ideas to each issue.

**Bring the group back together** to review what has been written.

There are some **resources** you can use together or hand out:

- ‘Family Food – the HAPPY guide to Getting Organised’ **hand-out 🕮**:

This is a resource or toolkit that parents can dip into for ideas, tips and pointers to further reliable and practical information (mainly web-based) to help them with any of the items listed above. If parents have identified something they feel is important and especially if they are not confident in that area –they could choose to start there.

It might be a good idea to encourage them to bring this back for the next two sessions as you might want to dip into it again together. There is a lot in it and some of it might become more useful in the future as their child/family grows.

- ‘Making Meals Healthier – Cutting the Fat but not the Taste’ in everyday recipes **hand-out 🕮**.
- ‘Happy Meal Planner’ **hand-out 🕮**.
- ‘Happy Shopping List’ **hand-out 🕮**.

It might be worth having some extra copies of the shopping list and planner for future weeks too.

If you have time **ask** the group to have a look through the resources within the session and pick one or two ideas from the ‘Getting Organised’ **hand-out 🕮** that they can have a go at before the next session.

**Remind** the group about where to find healthy recipes on the NHS Change for Life website - http://www.nhs.uk/Change4Life

**9.	TIME TO HAVE A GO	5 mins**

**Display** on a board the appropriate ‘Time To Have a Go’ suggestions for the week and give out the ‘Time to Have a Go’ **hand-out 🕮**

- Develop some family rules. Write them in your Memory Book and note what differences it makes to your family life.
- Get cooking! Try a new healthy recipe or make a favourite dish in a healthier way. Make a note in your memory book of what you made, how you adapted it and how it worked –what did others think –is there anything you would change if you did it again? You could bring a photo of it and the recipe to share with your group next session.
- Get organised. Have a go with using the meal planner and shopping list (see hints on getting started with this in the ‘Getting Organised’ **hand-out 🕮**) if this isn’t something you tend to do. If a full week seems too much try it for 2 or 3 days.
- Look at the **hand-outs 🕮**from this week and pick another topic from the ‘Getting Organised’ **hand-out 🕮**to think about more and use at home.
- Continue with the Pram Challenge and do a family physical activity together.
- Practice the time to play games.

**10.	WHAT DID YOU THINK OF TODAY?	5 mins**

**Give out** weekly feedback forms. **Play** relaxing background music whilst forms are being completed; place a container on the floor/table to collect forms. **Thank** the group for completing them. If there are literacy issues the whole group could respond to questions on flipchart and facilitator can record responses.

**11.	GOODBYE CIRCLE	5 mins**

**Stand** in a circle and say what a good session it has been.

Encourage everyone to say goodbye by giving a wave or thumbs up to encourage them to keep going with their physical activity pram challenge and other new ideas.

Optional – Give out a small gift such as a feeding bowl, feeding cup or bib as a tangible reminder of the session.

**Close** the session by saying how much you have enjoyed it and that you are looking forward to seeing everyone again at the next session and remind them of the date.

**WELCOME TO THE WORLD!**

**Postnatal Session Five (babies aged approx. 7 months)**

**STEP BY STEP**

PLAN FOR TODAY

|  | **ACTIVITY** | **TIME** | **MATERIALS NEEDED** |
| --- | --- | --- | --- |
| 1 | Welcome | 5 mins | Refreshments, music and player, ‘Welcome’ sign, attractive table cloth, flowers, badges, stickers, felt-tip pens, plan for today, attendance sheet, reward system, etc |
| 2 | Icebreaker: Ways of Praising | 10 mins | Flip-chart |
| 3 | Family Feedback | 10 mins | Flip-chart |
| 4 | Rewards, Praise and Communication | 15 mins | Flipchart, Board: What can I do to help my baby and me talk to each other? |
| 5 | Infant Diet | 15 mins | Hand-outs - ‘Dairy Council Menu Planner for Stage 2’, others for reference  Photos of first foods and finger foods |
| 6 | Mums’ Physical Activity | 5 mins | Pram Challenge slip |
|  | *TEA & COFFEE BREAK* | *10 mins* | *Refreshments* |
| 6 | Mums’ Physical Activity contd. | 5 mins | Pram Challenge map |
| 7 | Making the Most of Your Day | 50 mins | Daily Planner activities  Hand-outs – ‘Weaning Recipes and Ideas’, others for reference |
| 8 | Time to Play | 10 mins | Baby Sam, baby toys, balls |
| 9 | Time to Have a Go | 5 mins | Hand-out – ‘Time to have a Go’ |
| 10 | What did you think of today? | 5 mins | Hand-out – ‘Weekly Feedback’ |
| 11 | Goodbye Circle | 5 mins | Parting gift |

150 mins

**WELCOME TO THE WORLD**

**HAPPY Postnatal Programme**

**Session Five (babies aged approx. 7 months)**

**MAKING THE MOST OF YOUR DAY**

**STEP BY STEP**

**1.	WELCOME	5 mins**

**Display** board with ‘Welcome to HAPPY!’

**Greet** group members warmly as they arrive, have background music playing, and offer refreshments. **Ask** parents to make name badges, and to sign the attendance sheet.

**2.	ICEBREAKER: WAYS OF PRAISING	10 mins**

**Display** on the flipchart the phrases:

One way I can praise my baby is ……………

One way I can praise others is……………….

One way I can praise myself is……………….

**Invite** the group to introduce themselves this session responding to these phrases. If the group is taking a while to settle, parents can walk around the group sharing these ideas in a “mingle” before settling to begin the session.

**3.	FAMILY FEEDBACK	10 mins**

**Comment** briefly on last session’s feedback forms. Display the ‘Time to Have a Go’ from the last session and invite brief feedback:

- Develop some family rules. Write them in your Memory Book and note what differences it makes to your family life.
- Get cooking! Try a new healthy recipe or make a favourite dish in a healthier way. Make a note in your memory book of what you made, how you adapted it and how it worked –what did others think –is there anything you would change if you did it again? You could bring a photo of it and the recipe to share with your group next session.
- Get organised. Have a go with using the meal planner and shopping list (see hints on getting started with this in the ‘Getting Organised’ **hand-out 🕮**) if this isn’t something you tend to do. If a full week seems too much try it for 2 or 3 days.
- Look at the **hand-outs 🕮**from this week and pick another topic from the ‘Getting Organised’ **hand-out 🕮**to think about more and use at home.
- Continue with the Pram Challenge and do a family physical activity together.
- Practice the time to play games.

**4.	REWARDS, PRAISE AND COMMUNICATION	15 mins**

Last session we talked about developing some family rules for creating a happy and calm family life. We have also spoken in other sessions about the value of praise for children, for other adults and for praising ourselves. We have talked about praise for being and praise for doing.

**Invite** parents to give examples of how they have been using this with their babies – brief discussion for a few minutes.

Let’s spend a bit more time today thinking about appropriate use of rewards for very young children and a useful behaviour encouragement technique to encourage co-operation in children and reduce power struggles.

With very young children and babies we should only be giving rewards and praise for their good behaviour and not imposing penalties for unwanted behaviour – they are too young to understand this yet.

The greatest reward for babies and toddlers is the positive attention and pleasure shown to them by their carers.

‘WHAT WE PAY ATTENTION TO IS WHAT WE GET MORE OF’

Children and babies love attention and will do what they need to get it. The more attention is given to good behaviour the more good behaviour there will be.

**Invite** the group to think of things they can do to give attention to their babies and write the responses down on a flipchart (e.g.: cuddles, play, smiling, singing, massage, positive phrases, looking at books).

**Re-introduce** the board from session 5 antenatal programme about talking to babies - What Can I Do To Help My Baby and Me Talk To Each Other? – and invite some discussion about what people have been doing with their babies and what response they have received? Have they noticed others in the family being involved too?

**Suggest** to parents that it is important to be as descriptive as possible about the behaviour that they want, or descriptive about the behaviour that they see e.g.: ‘thank you for letting me wipe your sticky fingers’, ‘that’s a lovely smile’, ‘I can hear you’re telling me a lovely story’. Try to avoid vague concepts such as ‘be good’ or ‘behave nicely’.

As children get older we want them to take some responsibility for themselves by encouraging their use of personal power. The concept of giving children choices is a first step towards this and one that can be introduced to babies from about 6 months old. Finding appropriate choices to give very young children reduces conflict and builds a sense of independence. So for babies at 6 months old offering 2 different finger foods to choose from such as pieces of banana or orange already give the baby a choice even before they have the language to understand this. As they get older they can be given more choices such as water or milk to drink, a cheese or a tuna sandwich, this story to that story.

This behaviour encouragement technique allows adults to stay in control of the choices but encourages children to start taking some responsibility and can be a great way of avoiding conflict in toddlers and children.

**5.	INFANT DIET	15 mins**

Ask mothers how is feeding going?

Most mothers will have started weaning by now. In pairs ask mothers to briefly share experiences and any concerns.

Use the food photographs that were used in session 3 for the planning weaning exercise.

**Ask** mothers to identify foods that they have offered to their baby. Did they like them? Are there any that they spit out? What does this mean? What did they do when baby did this?

**Identify** 3 new foods that mothers will offer their baby to encourage variety.

Collate the concerns on a flipchart and add the following ‘what if’ scenarios, if necessary

What if my baby spits out a particular food - should I avoid it as he/she does not like it?

What if my baby makes a lot of mess ….. is this normal?

What if my baby eats very slowly…. what should I do?

Refer to the **hand-outs 🕮** and booklets used in previous sessions to answer any concerns and remind parents to find solutions within the resources they have.

Start 4 Life – Introducing Solid foods

‘How to Start Weaning – A Practical Guide’ **hand-out 🕮**

‘Weaning – What and When’ **hand-out 🕮**

Next Stages of Weaning

Use the resources from above and some additional photos of finger foods. Also give out **hand-out 🕮** ‘Dairy Council Menu Planner for Stage 2’

**Invite** parents to discuss the following questions.

What should 6-9 month olds eat and drink?

Why do we introduce finger foods?

How much milk will they be drinking as they eat more solid foods?

The answers to these questions will be found in the **hand-outs 🕮** referred to above.

The aim of this exercise is to ensure that parents are aware of the progression to finger foods and towards food with more lumps and texture, as well as an increased variety. We will spend a bit more time later this session planning some meals for your baby.

**6.	MUM’S PHYSICAL ACTIVITY	5 mins**

**Ask** the group to write + 50 miles on their pram pedometer step record if their families played any time to play games with their baby over the last month. **Ask** each member of the group to fold their step record in half so that no one else can see it and pass it into you.

The practitioner totals the group activity during the break. The scoring system is 2000 steps = 1 mile, remember to add on the additional bonus miles.

**Tea and Coffee Break	10 mins**

**6.	MUM’S PHYSICAL ACTIVITY CONTINUED	5 mins**

**Ask** the group to reflect silently and think about what they did or didn’t do towards the challenge. Ask them to come up with a rating for themselves out of 10 based on what they did or didn’t do.

Did they do the activities which they had planned to do?

How many times did they do something towards the Challenge?

How long did they do physical activity for, how much effort did they put in?

**Check** that everyone has given themselves a score and ask them to write their score in the Memory Book: ‘My pram challenge score out of 10 on the [date] was…’ **Ask** if anyone wants to share what they did or didn’t do toward the Challenge, what their rating was and why they have given themselves that rating. Ask the group to give the people who share positive feedback, praise or encouragement. **Praise** the group yourself by saying well done to those of you who really went for it and worked hard at the Pram Challenge over the past few weeks. **Ask** the group to write in their Memory Book what rating they would like to give themselves at the next session. For example, if you rated yourself as an 8 this time, next time you might want to be 10! Write down what you would need to do so that you could rate yourself with a higher score at the next session.

**Reveal** to the group how many miles they have travelled and remind them about how far they have to go to reach their target. Encourage everyone to clap and cheer!

**Ask** the group to decide on a goal for the next session, bearing in mind what they achieved this time. **Write up** the target on a flip chart.

**7.	MAKING THE MOST OF YOUR DAY	50 mins**

**Explain** to the group that their daily routines will be changing now that their babies are beginning to eat solids and will also still be having milk feeds. By 7-9 months of age, a baby should have started on a range of puréed and mashed foods and some finger foods, and be eating three meals a day, as well as having several (about 4) milk feeds of breast milk or infant formula (a total of about 600ml a day). This time can be difficult time to stay organised and maintain healthy habits for the family.

Give each member of the group a **Day Planner Board** and put the **Daily Activity Cards**, **Time Cards** and **Blank Cards** in the middle of the table/floor.

Think about a typical day for you: what do you usually do in the morning, in the afternoon and in the evening?

**Show** the group the Daily Activity Cards and explain: these are routine activities such as waking up, household chores, sleeps, and going to bed. There are also some ‘healthy’ activity cards, such as baby’s healthy meal, my healthy meal, my nurturing time, my physical activity, and baby’s tummy/floor time.

**Plan** a typical day for yourself and your baby. For mums/dads, each of them should include somewhere in their day going to a parent and baby group activity, for example a parent and toddler group, or baby massage, or baby sing-a-long or perhaps meeting up with a friend and going to the park.

**Reassure** the group: you may not be able to fit all the activities into one day! It is up to you to choose the activities that you plan for, as long as they include a parent and baby group activity. There are blank cards to add in any other routine activities that you do, for example if you usually visit a relative, or walk the dog then you can write these activities on a blank card and include it in your plan.

**Suggest**: for mums who are working, it may be best if you plan a day when you are not at work, perhaps a weekend day.

**Place** each activity in order in the ACTIVITY column of your Day Planner Board. You can place two activities next to each other on your Day Planner Board if you are able to do them at the same time. For example you may be doing household chores whist your baby is having some tummy time. Or, your partner may be playing with the baby doing some ‘time to play’ activities whilst you have some nurturing time for yourself, like having a relaxing bath or painting your nails.

Also select how long you plan to do each activity for. There are Time Cards to place in the ‘How long for?’ column on your Day Planner Board.

Give the group a **demonstration** by sticking a few activities onto a board with some timings for each activity. You don’t need to complete a full day. For example:

| **Day Planner** | | | |
| --- | --- | --- | --- |
|  | Activity 1 | Activity 2 | How long for? |
| Morning | Wake-up  Milk feed  Baby’s healthy meal | My healthy meal | 15 mins  15 mins |
| Afternoon | Baby’s healthy meal  Parent and child group | My healthy meal | 30 mins    1 ½ hrs |
| Evening | Baby’s healthy meal  Time to play | Nurturing time  My healthy meal | 30 mins  15 mins  30 minutes |

Before you start, remember these important recommendations for your own physical activity and for your babies’ play time.

It is recommended that adults do 30 minutes of moderate intensity activity on 5 days of the week. This can be built up in blocks of 10 minutes throughout the day. **Show** the group the physical activity ball **hand-out 🕮** as a reminder of different types of physical activity that they could be doing during the day. **Explain**, if you are scrubbing the bathroom for 10 minutes, this counts as physical activity if you feel that your heart rate is slightly raised and you are breathing a little harder. On your Day Planner you could have two activities next to each other, household chores and my physical activity.

It is recommended that babies do 10-15 minutes of tummy time (or floor time, i.e. crawling, now that they are older) 2-3 times each day.

It is recommended that babies play in a structured way with an adult several times throughout the day, the ‘time to play’ activities that we do here are examples of structured play.

Also remember that your baby should be eating three meals a day, as well as having several (about 4) milk feeds of breast milk or infant formula (a total of about 600ml/a pint a day).

**Write** these recommendations up on a flip chart where the group can see to remind them as they are planning their day.

Once the group has finished planning their routine, **continue** with the following exercises. **Ask** the group to use the **hand-out 🕮** ‘Weaning – What and When’ (PN3) for ideas of foods to offer and other **hand-outs 🕮** for meal ideas:

- ‘Dairy Council Menu Planner for Stage 2’;
- Photographs of meal ideas from CHEW;
- ‘Weaning Recipes and Ideas’

Using the structure below ask parents individually to plan a day in the life of their baby. What will they eat and drink at the following times of day? **Reinforce** the message of responsive feeding: parents should provide food and the baby will decide whether they eat it or not. Reassure parents that it is okay for their baby to refuse the food. When choosing food and drink **encourage** mothers to think about the variety of colours, textures and amounts of food they will offer.

|  | **Food** | **Drink** |
| --- | --- | --- |
| **Breakfast** |  |  |
| **Midmorning** |  |  |
| **Midday** |  |  |
| **Afternoon** |  |  |
| **Evening** |  |  |
| **Bedtime** |  |  |
| **Night time** |  |  |

**A thought about portion sizes**:

Parents are often not sure about how much to food should be given and there is generally a lot of confusion about this. Portion sizes for this age group of infants are not available. They are available for toddlers i.e. 1-3 year olds from the Infant and Toddler Forum. <https://www.infantandtoddlerforum.org/portion-sizes-table>

Therefore **remind** mothers that their baby’s stomach is small and they should only offer enough for his/her needs. Be responsive to their baby’s appetite and do not be tempted to overfeed by making them finish all the food they have prepared. If baby stops eating, try once to encourage them to eat a little more however if they does not want any more then take the food away. You should decide what is offered however **be guided by your baby’s appetite.**

Remember that it can take several experiences of new tastes and textures for babies to get used to a new food – there is a lot for them to learn about chewing and eating so don’t assume that because they pulled a funny face or pushed a food out that they won’t like it. Try again another time.

**Ask** mother’s in pairs to list some signs of when your baby has had enough: e.g., they turn head away, scream, shout or cry, spit food out repeatedly

PARENT AND BABY GROUP DISCUSSION

**Ask** the group to talk about their experience of attending other parent and baby groups; what have you been to? What have you liked and disliked about these other groups? What have your children enjoyed doing? **Explain** to the group that they will get 50 extra Pram Challenge miles if they attend a group with their child before the next HAPPY session, as a way of supporting them to continue to socialise with other parents and get support once the HAPPY programme comes to an end. **Ask** them to look out for parent and baby activities in their local area and take a note of them in their Memory Books, so that at the next session they can share these details with the group.

Ask the group about some of the challenges they face when they want to take their baby out to a parent and baby group, like HAPPY. You might need to prompt:

Do you feel low in confidence?

Or stressed when you are getting ready to go out?

What can you do to over-come these challenges?

**Make sure** someone suggests asking a partner, friend or family member to go to the group with them, if not add this suggestion yourself; suggest asking one of their friends from this group to go along with them. Also suggest that that they could use self-talk, this is repeating positive thoughts about yourself, or imagery; imagining feeling confident and positive. Tell the group you are going to practice these techniques now.

**Ask the group to close their eyes or look down and read the following scenario. Ask them to feel the emotions as you read through it.**

You have booked onto a 5 week baby massage course. You don’t know anyone else in the group and you are feeling nervous and apprehensive about going along. What if you do it wrong or your baby won’t stop crying during the session? You feel embarrassed talking to people that you don’t know: what if they don’t like you, and you don’t make friends with anyone?

As you are just about to leave you realise that your partner has not left the money for you that he said he would and you begin to feel angry towards him. Then the baby is sick everywhere and you have to change their clothes. You’re just about to leave again and now you can’t find your keys. The whole world seems against you and all you are trying to do is go to a parent and baby group.

Feel all those emotions of nervousness, worry, anxiety embarrassment, anger and frustration (allow the group to sit quietly for 10 seconds).

Now take some deep breaths in and out. As you breath out let go of all your negative emotions, the nervousness, anxiety, and frustration and as you breath in begin to feel calm, confident, energised and happy. Picture how you look, and see yourself looking happy, positive and confident. In your mind, say to yourself: I am happy, positive and confident. Keep repeating this to yourself in your mind. Let the group sit in stillness for a moment.

**8.	TIME TO PLAY	10 mins**

As your baby gets stronger they will be able to sit without support. This usually happens between 6 and 8 months. Remember to celebrate these milestones, get excited for your baby cheer and clap when they develop a new skill.

**Playing Ball**

Babies are fascinated by balls. **Roll** a soft ball toward your baby and watch them grab and squeeze it. Eventually, with encouragement, they'll roll the ball back toward you. When your baby starts to crawl they will love chasing a ball! **Encourage** the group to practice playing ball with their baby.

**Tummy/floor time**

When the group is sitting quietly again remind them of the following: babies love to move around, make sure your baby has plenty of floor time so that they can practice moving between crawling and sitting. You can encourage your baby to move around by placing interesting toys just outside of their reach on the floor or on a low sofa; this will encourage them to pull themselves up to standing.

Baby equipment like a pushchair, car seat, rocker, or swing is very restrictive and can affect the way that the muscles develop. Spending more than 2 hours per day in equipment has been linked with a delay in the development of movement skills such as rolling, crawling and walking.

Your babies’ brains are developing rapidly so they need to play actively even though the constant supervision that they need may be tiring. Try to rest when they rest. Avoid using TV or DVDs as a baby sitter. Television is not recommended for children under 2 years old. You can use a safety gate or play pen to keep your baby safe and allow them to interact with you when you are in the kitchen cooking, or in the bathroom showering. You can keep a few special toys for them to play with at these times.

**Sing-along and move: Ring a Ring o’ Roses**

Your baby may like being helped to stand and take the weight through their legs. This will help them to develop strength in their legs.

As a group (using baby Sam to demonstrate) **sing** the song together. **Encourage** parents to hold their babies under their armpits, in a standing position.

**For the first verse**: for the first three lines demonstrate to parents bouncing the baby up and down on their feet, on the last line lift them up and then pop them down to sitting.

**For the second verse**: clap hands together for the first three lines, then on the final line, hold the baby underneath the arm-pits and bounce them up to standing again.

Ring a ring o’ roses

A pocket full of posies

Atishoo, Atishoo,

We all fall down

The cows are in the meadow

Eating butter cups

Atishoo, atishoo

We all jump up!

**9.	TIME TO HAVE A GO	5 mins**

**Display** on a board the appropriate Time To Have a Go suggestions for the week and give Time to Have a Go **hand-out 🕮**.

- Notice how you are using praise, rewards and communication with your baby to encourage positive behaviour and encourage others in your family to do the same.
- Continue with the Pram Challenge including going to an existing group or a new group with your baby.
- Keep an eye out for other mother and baby groups near to you so that you can share these with the group at the next session.
- Have a go at the ‘Time to Play’ activities.
- Introduce 3 new foods to your baby and record which ones and how this went in your Memory Book.
- Think about your meals and eating -could they (or some of the foods they include) soon be shared with your baby -would you need to change anything about how you prepare them to make them suitable
- Use the Getting Organised resources from week 4 to help you think about what you could do to make some meals for you that can also be adapted to feed your baby or think about any of the other topics covered that might be useful for you to think about now.

**10.	WHAT DID YOU THINK OF TODAY?	5 mins**

**Give out** weekly feedback forms. **Play** relaxing background music whilst forms are being completed; place a container on the floor/table to collect forms. **Thank** the group for completing them. If there are literacy issues the whole group could respond to questions on flipchart and facilitator can record responses.

**11.	GOODBYE CIRCLE	5 mins**

**Stand** in a circle and say what a good session it has been. **Encourage** everyone to say goodbye by touching the foot of the next person and leaving it touching until the feet form a circle on the floor.

Optional – Give out a small gift such as a glass bead or key ring as a tangible reminder of the session.

**Close** the session by saying how much you have enjoyed it and that you are looking forward to seeing everyone again at the next session and remind them of the date.

**WELCOME TO THE WORLD!**

**Postnatal Session Six (babies aged approx. 9 months)**

**STEP BY STEP**

PLAN FOR TODAY

|  | **ACTIVITY** | **TIME** | **MATERIALS NEEDED** |
| --- | --- | --- | --- |
| 1 | Welcome | 5 mins | Refreshments, music and player, ‘Welcome’ sign , attractive table cloth, flowers, badges, stickers, felt-tip pens, plan for today, attendance sheet, reward system, etc |
| 2 | Icebreaker: Reflections | 10 mins | Flipchart |
| 3 | Family Feedback | 10 mins | Flipchart |
| 4 | Continuing the Family Journey | 30 mins | Flipchart  Prepared post-it notes |
| 5 | Mum’s Physical Activity | 5 mins |  |
|  | *TEA & COFFEE BREAK* | *10 mins* | *Refreshments* |
| 5 | Mums’ Physical Activity Continued | 5 mins | Reward for Pram Challenge |
| 6 | Keeping on the Right Track | 20 mins | Hand-out – ‘Keeping on the Right Track’ |
| 7 | Infant Feeding/Family Feeding | 10 mins | Hand-out – ‘Ten Steps for Healthy Toddlers’  Me-Sized Meals Activity |
| 8 | Mum’s Physical Activity | 10 mins | Hand-outs – ‘Physical Activity Ball for Adults’, ‘My 30 Minute Activity Ball’ |
| 9 | Infant Play | 10 mins | Baby Sam, baby toys  Hand-out – ‘Help your child move and play every day’ |
| 10 | Time to Play | 10 mins |  |
| 11 | What did you think of today and the whole programme? | 15 mins | Ball of string/wool  Hand-out – Weekly Feedback Certificates |

150 mins

**WELCOME TO THE WORLD**

**HAPPY Postnatal Programme**

**Session Six (babies aged approx. 9 months)**

**CONTINUING THE HAPPY JOURNEY**

**STEP BY STEP**

**1.	WELCOME	5 mins**

**Display** board with ‘Welcome to HAPPY!’

**Greet** group members warmly as they arrive, have background music playing, and offer refreshments. **Ask** parents to make name badges, and to sign the attendance sheet.

**2.	ICEBREAKER: REFLECTIONS	10 mins**

**Display** on the flipchart the following statements:

*One thing I have gained by coming on the HAPPY Programme is……………….*

*One way I have changed by being in the group with all of you is………………..*

**Invite** everyone to complete the statements.

**3.	FAMILY FEEDBACK	10 mins**

Comment briefly on last session’s feedback forms. Display the ‘Time to Have a Go’ from the last session and invite brief feedback:

**4.	CONTINUING THE FAMILY JOURNEY	30 mins**

**Note for group leaders**: Why have we developed this course? The 12 week programme - HAPPY - was developed with the aim of supporting parents at an important time in their lives to think about their own lifestyle and what sort of environment they would be providing for their new baby. It has aimed to provide parents with an opportunity to think about what sort of parent they are/will be and has offered appropriate knowledge and skills they require so they are in a good position to give their baby the best start in life in terms of their diet, physical activity and emotional health. We realise that putting some of the advice into practice will require the support of family and friends and we hope the sessions have also given parents the opportunity to think through what might be challenging situations related to changing behaviours or adopting new ones, and through the activities and group support to generate some solutions on how these will be put into practice. In addition it is hoped that the programme has provided parents with the skills and confidence to ask for support/help as they require so that they also make time for themselves to look after themselves too.

KEY MESSAGE: Now that we’ve reached the end of the HAPPY Programme we hope everyone feels better equipped to provide their children with the best healthy start in life and for you all to enjoy and cope with your family’s journey. We want you to use all the tips and suggestions, the books and the leaflets that you’ve been given as a guide to keep you and your family on track.

**Refer** the group to the illustration of the family journey in The Parenting Puzzle (Pg231). If you can, draw a large version onto the flipchart for group discussion and point out the key points:

- Where are we now – how far we’ve come/what have we learnt
- Where we’d like to finish – our long term aims in our relationships and our healthy lifestyles
- Toolbox – what tools, tips and strategies do we have
- Family Maintenance Kit – what helps us cope
- Early Warnings – what are the stress warning signs for us and for children
- Signposts – what are the signs that we’re on the right track
- Major Hazards – what are the extra stressful situations that may come along in life
- In Case of Breakdown – what’s in our emergency survival kit to get us back on track

We’re going to spend some time reminding ourselves of what we’ve learnt and then thinking about what will help us in the future.

WHERE ARE WE NOW?	**10 mins**

**Invite** the group to consider how far they have come by contributing their ideas about what they have learnt under a few key headings. Put up sheets of flipchart paper around the room with one or two headings on each and invite parents to write their ideas on the paper (assist those with any literacy issues or with some prompts). Remind the group this is not a test, but an opportunity to refresh our ideas about some of the things we’ve learnt.

- **My Physical Activity**

(Active lifestyle, 30 minutes of physical activity each day, pelvic floor exercises)

- **Baby’s Play**

(Tummy Time every day, playing and exploring their environment every day, playing together every day, not restricted in a car seat, buggy, high chair, rocker or walker for more than two hours each day)

- **Family Time**

(Being active as a family, giving praise for being and praise for doing, building our self-esteem, recognising feelings and what they tell us, talking about family rules)

- **TV time**

(Limiting how much TV is watched by all the family, TV is not on when baby is playing or eating)

- **Healthy eating for adults**

(Eating more fruit and vegetables; setting a good example by eating healthier food; reducing portion sizes; eating less fried food, less sugary foods, less takeaways; eating a more varied diet; eating together)

- **Healthy eating for babies**

(Establishing a routine around meals, eating together, making more homemade food from healthy ingredients, allowing baby to decide when they are full, introducing solids later – around 6 months, giving water and fewer juice drinks, offering more fruit and vegetables, introducing a varied diet)

WHERE WOULD WE LIKE TO FINISH?	**15 mins**

The aim of the HAPPY programme has been to develop a healthy lifestyle for our family to help give the best start in life to our children. We’ll spend a time now thinking about what a healthy baby looks like and what are the benefits of a healthy lifestyle long term.

ACTIVITY DISCUSSION

Ask mothers to think of a list of words that describe what a healthy baby looks like: e.g. not too skinny; chubby; active; happy; etc…

**Feedback**….. focussing on the concept of a healthy weight. What is normal? Is it normal to be chubby? What does not too skinny mean? How do we know whether our baby is a healthy weight? This programme can’t give an answer to these questions.

One of the key aims of the programme is to prevent childhood obesity. In our communities however parents generally find it difficult to judge whether their child is overweight. This is not surprising because as the number of overweight/obese children is increasing, it becomes difficult to tell whether your child is a healthy weight or overweight.

One of the ways to keep an eye on this is to check with your Health Visitor when they weigh and measure your baby at clinic and plot the information on growth charts. She will then be able to assess how your baby is growing. The growth charts are made up by using the information on children from this country and show how children grow according to their age and their gender (there are separate ones for boys and girls). The charts consist of a number of lines (centiles) – each of these centiles show the normal growth patterns of children. Health professionals generally want to see babies following the same growth lines and not suddenly going up or down. They also look at weight in relation to height. Your health visitor may also work out the body mass index (BMI) of your child and then plot it on a children’s BMI growth chart.

Talk to your health visitor if you have any more questions about this. There is more information on the NHS website too. [www.nhs.uk/conditions/pregnancy-and-baby/pages/baby-screening.aspx#close](http://www.nhs.uk/conditions/pregnancy-and-baby/pages/baby-screening.aspx#close)

**Invite** the group to consider the long term benefits of a healthy lifestyle and the consequences of an unhealthy lifestyle. When the going gets tough it’s helpful to remember why we’re trying to live in a healthier way.

**Prepare** a flipchart paper with the 2 headings –

Benefits of a healthy lifestyle	Consequences of an unhealthy

lifestyle

Using the examples below **prepare** some post-it notes in advance with a number of the statements. **Share** the post-it notes around the group and invite them to come to the flipchart and put their statement under the heading they think is right.

Have a **brief discussion** to summarise the learning from this and check that all agree on the placement of the post-it notes.

| **Benefits of a healthy lifestyle**  Healthy lifestyle-including  Good diet and enough physical activity | **Consequences of an unhealthy lifestyle**  Unhealthy lifestyle-including  Poor diet and too little physical activity |
| --- | --- |
| *Healthy weight, height and growth*  *Better routine –less arguments*  *Healthy and happy approach to food and play/exercise*  *Likes a varied and balanced diet*  *Less faddy eating*  *Can save money on food by planning and put it towards a holiday or a n outing*  *Strong bones & teeth*  *Good level of physical fitness and stamina – less tired and more capable*  *More confident and higher self-esteem –happier*  *Better sleep*  *Less illness*  *Miss less school/work*  *Does better at school/in education*  *Less health problems as adult*  *Healthier future pregnancies and babies for girls when they grow up*  ***Longer higher quality life*** | *Does not get used to or learn how to eat healthy balanced diet*  *Does not develop ability to join in recreational physical activity with others*  *Become Overweight or obese*  *Weaker bones and teeth, sleep problems*  *More illness, miss more school*  *Associated longer term health problems –*  *Diabetes, breathing problems, higher risk of cancer*  *Infertility (male and female), pregnancy problems and risks for girls*  *Poor level of physical fitness –easily tired, less capable of coping with physical demands of school/work*  *Joint Problems, reduced mobility and disability*  *Difficulty with clothes*  *Less confident /lower self esteem*  *Possible impact on school and educational attainment*  ***Shorter poorer quality life*** |

**5.	MUM’S PHYSICAL ACTIVITY	5 mins**

**Ask** each member of the group to copy their totals from their individual record onto an anonymous ‘Pram Challenge’ slip. **Encourage** the group to be honest and tell the truth about what they did/didn’t do.

Totals are for:

1. Steps recorded from previous to present session
2. Hours and minutes spent swimming
3. Hours and minutes spent cycling
4. Hours and minutes doing strength/flexibility exercises
5. X extra miles for attending another parent/baby group

**Ask** the group to write + 50 miles on their pram pedometer step record if their families played any time to play games with their baby over the last month. **Ask** each member of the group to fold their step record in half so that no one else can see it and pass it into you.

The practitioner totals the group activity during the break. The scoring system is 2000 steps = 1 mile, remember to add on the additional bonus miles.

**Tea and Coffee Break	10 mins**

**5.	MUM’S PHYSICAL ACTIVITY CONTINUED	5 mins**

**Announce** where the group has got to on the Pram Challenge and **celebrate** the achievement whether they have reached their destination or not. **Give out** an appropriate reward. **Encourage** the group to cheer and clap loudly!

**6.	KEEPING ON THE RIGHT TRACK	20 mins**

We have been talking about healthy family lifestyles and getting good habits and patterns of eating and activity established for now and as our families grow up.

Some of the changes can be quite hard to make or to sustain because lots of things get in the way. Referring again to the illustration of the family journey in The Parenting Puzzle (page 231), let’s be aware that there may be ‘Major Hazards’ – the extra stressful situations - that come along in life and put us off course or get in the way of leading a healthy and active lifestyle.

**Invite** the group to consider what those may be:

E.g.: death of someone close, marriage, divorce, new partner, moving house, changing school or job, redundancy, illness, new baby, influence of others, parental pressure, extra caring responsibilities etc. etc.

**Ask** the group to work in pairs to think how they will respond ‘In Case of Breakdown’ – what’s in your emergency survival kit to get you back on track. E.g.: Look at the Parenting Puzzle; read the leaflets and hand-outs from HAPPY; talk to a friend or family member; use my Memory Book to remind myself of why I should lead a healthy active lifestyle and write new plans; set aside time to think about where my family is going; ask for help from family, friends, agencies, groups.

**Share** the ideas with the whole group.

Using the ‘Keeping on the Right Track’ **hand-out 🕮**, ask parents to fill this in using the ideas that have been talked about so far in the session. **Ensure** that all parents are confident in finding solutions to the difficulties they have now or the challenges that they may be anticipating in future.

**7.	INFANT FEEDING/FAMILY FEEDING	10mins**

Refer to ‘Ten Steps for Healthy Toddlers - Good Habits for Health, Growth and Development’ **hand-out 🕮**,

This **hand-out 🕮** gives 10 steps for healthy toddlers. It covers the main areas that parents should focus on achieving in relation to healthy eating, family food, rewards and physical activity. Page 2 gives appropriate portion sizes of food that should be offered to toddlers i.e. 1-3 year olds.

GROUP ACTIVITY

in groups **ask** parents to look at the **hand-out 🕮** and discuss whether they feel confident in following the 10 steps. Do they foresee any barriers? If so what solutions can be offered by the group?

**Portions and Me–Sized Meals**

KEY MESSAGE: A lot of families forget that different family members need different amounts to eat – they give small children adult portions or divide food equally between everyone. If they then insist on children eating everything on their plate they teach the child to override their in-built appetite controls and large becomes normal which can lead to overweight and obesity.

It’s also important when living and eating together with others to remember that people within the family need different amounts of food according to their age, whether they are growing, level of physical activity – one of the skills of parenting is learning to judge the right sized portions for different family members –think ‘me-sized meals’.

GROUP ACTIVITY: MATCHING GAME WHO’S BREAKFAST IS WHO’S?

**Preparation**

Stick pictures (or find photos) of the following family members

- 10 month old (baby in high chair)
- Woman – medium height
- Man – tall
- 15 year old boy – school bag and football

**Write** (or even draw!) the 4 breakfasts onto large paper plates or just sheets of paper.

- 1 Weetabix or 3 tablespoons of cereal with full fat milk, half a banana sliced, pure fruit juice well diluted with water in a lidded cup (belongs to baby)
- Medium bowl cereal (e.g. 2 Weetabix), banana and semi skimmed milk, orange juice (belongs to Mum – reasonably active – 5’4’’)
- Same as above – plus a slice of toast (belongs to Dad - 6’2’’ – not very active at the moment. He has noticed that since he stopped walking to work because of the winter weather his appetite has reduced a little and has reduced his portion sizes a bit)
- Large bowl of cereal, semi skimmed milk on cereal and half a pint to drink, 2or 3 or more slices of toast, low fat spread, juice to drink. Plus Takes hot cross bun or cereal bar as snack for mid-morning and money for a full school dinner(belongs to 15 year old boy – height seems to change daily as does his shoe size – walks half an hour to and from school daily and plays sport most days - likely to be hungry again within the hour !!)

**Ask** the group to match the breakfast to the family member quickly.

It’s normal for the adults in the family to need larger portions than the smallest family members and smaller portions than teenagers. Those who are more active may need more food than those who are more sedentary. If parents provide a balanced diet and children are allowed to regulate the amount of it they eat themselves then things usually work out!

Children need foods and drinks that give them energy (calories) and protein and are also rich in nutrients like calcium, vitamins and iron –many fat and sugar rich foods, drinks and snacks (e.g. chips, crisps, biscuits, sugary drinks, sweets) are high in calories but poor in these important nutrients for growth and development – they are ‘empty calories’ Getting the balance right for family meals is important.

**8.	MUM’S PHYSICAL ACTIVITY	10mins**

**Tell** the group that together you will do one final activity around planning physical activity for yourself.

The steps we will go through are:

1.	Identifying what the physical activity goal is: this is to do 30 minutes of activity.

2.	Decide on the activity/activities (break them down into manageable chunks of 10 mins).

3.	Decide when you are going to do it.

4.	Write down the problems might come up and how will you overcome them.

5.	Identify what support you need to do it – and ask for it.

**Remind** the group that it is recommended that adults do 30 minutes of moderate intensity activity on 5 days of the week. This can be built up in blocks of 10 minutes throughout the day. Show the group the ‘Physical Activity Ball’ **hand-out 🕮** as a reminder of different types of physical activity that they could be doing during the day. Explain that scrubbing the bathroom for 10 minutes counts as physical activity if you feel that your heart rate is slightly raised and you are breathing a little harder.

Ask the group to complete the **hand-out 🕮** ‘My 30 Minute Activity Ball’. Draw or write onto the ball the activity/activities that they are going to do and complete the questions.

**9.	INFANT PLAY	10 mins**

Enrolling at a children’s centre or going to parent and baby groups are a great way to stay connected to other parents, and get the support you need to continue yours and your baby’s health lifestyle.

**Ask** the group to feed back about any groups they have attended and can recommend or have heard about to other parents. Did they write any down in their Memory Book? Ask the group to share information about any outdoor spaces, parks, playgrounds that they use, and any indoor spaces, like soft play venues, children’s centres and playgroups. **Gather** all of the information and tell the group that you will send a printed list though the post to them to support them to stay connected with other parents.

Suggest to the group that they use each other to stay active, for example they can meet up to go to a park, have a walk and let the children play together.

**Give** out the **hand-out 🕮** ‘Help your child move and play every day’. **Explain** that as your baby becomes a toddler the types of activities and games that they can play will change. **Ask** the group to look through the **hand-out 🕮**: are there any suggestions in here that they could start to do already? Ask them to put a star next to the things that they would like to try.

**10.	TIME TO PLAY	10 mins**

**Balancing babies**

To help improve your babies’ balance, **sit** them on a soft surface or place a cushion behind them as they may still fall backwards. **Place** toys/interesting household objects so that they can just reach them without tipping over. **Encourage** them to reach forward, sideways, upwards and later slightly behind them. **Praise** them when they reach the object and then give them plenty of time to play with the toy/object **Demonstrate** the game using baby Sam and encourage the group to join in with their babies if they are there, awake, alert and ready to play.

**Crawling and walking**

Your baby will love physical challenges as they are growing and developing. Give them cushions to crawl over or a large cardboard box on its side to crawl into. To encourage stepping and walking, position your baby so they are holding onto a sturdy piece of furniture like a sofa. Place toys along the sofa to encourage them to step. Later they may be able to walk forward if they hold your fingers. Their balance will be better in bare feet so leave shoes off when it is safe. Babies will often fall, so have them practice walking on carpet or grass rather than a hard floor.

**Sing along and move ‘When you’re happy and you know it’**

**Encourage** the group to sing along and perform the actions. If their child is alert and ready to play they can join in the actions too and encourage parents to help them. **Tell** the group they can have fun making up the verses as they go along. At the end of the song ask if anyone has a verse they would like to add and then sing the new verses too.

***When you’re happy and you know it***

*When you’re happy and you know it clap your hands*

*(clap clap)*

*When you’re happy and you know it clap your hands*

*(clap clap)*

*When you’re happy and you know it and you really want to show it*

*When you’re happy and you know it clap your hands*

*(clap clap)*

*When you’re happy and you know it stamp your feet*

*(stamp stamp)*

*When you’re happy and you know it stamp your feet*

*(stamp stamp)*

*When you’re happy and you know it and you really want to show it*

*When you’re happy and you know it stamp your feet*

*(stamp stamp)*

*When you’re happy and you know it wave your arms*

*(wave wave)*

*When you’re happy and you know it wave your arms*

*(wave wave)*

*When you’re happy and you know it and you really want to show it*

*When you’re happy and you know it wave your arms*

*(wave wave)*

**11.	WHAT DID YOU THINK OF TODAY & THE WHOLE PROGRAMME?	15 mins**

**Give out** feedback forms for the whole programme as in previous weeks, and thank the group for completing them.

GOODBYE CIRCLE – A WISH FOR THE FUTURE

**Stand** in a circle, and say how much you’ve enjoyed being with the group over these twelve sessions and one special memory that you will take away from them is….

PRESENTATION OF CERTIFICATES

**Give out** to each parent a certificate with someone else’s name on it; **invite** someone to present the certificate they are holding to the appropriate person, while the others applaud; this parent then presents to the next person, and so on until everyone has received their own certificate.

GOODBYE CIRCLE: Web of Friendships

**Invite** the group to stand in a circle. **Mention** that as at the end of the ante-natal programme you are going to weave a web of friendship as a way of saying goodbye.

**Ask** the group to think of one important thing they will take away from the course; **give** everyone a few moments to reflect. Keeping hold of the free end, and unravelling a length of string, **throw** a ball of string to one parent; **invite** them to say what they will take away, then to throw the ball of string to someone else, unravelling enough to throw it across the circle while holding onto the string with their other hand. The person now holding the string does the same; **repeat** until everyone has had a turn and you are all connected together. **Pause** to let people comment if they wish, taking time to enjoy the moment together before you ask them to drop the web onto the floor.

**Thank** the group for their contributions throughout the HAPPY programme, and offer your warm good wishes for their future.
