# Supplementary material for: Using intervention mapping to develop a culturally appropriate intervention to prevent childhood obesity: the HAPPY (Healthy and Active Parenting Programme for Early Years) study
Source: Int J Behav Nutr Phys Act. 2013 Dec 28;10:142. doi: 10.1186/1479-5868-10-142 (PMC3895739; doi:10.1186/1479-5868-10-142)
Supplement: Additional file 4 — a. MASTER HAPPY Antenatal Manual FINAL VERSION. b. MASTER HAPPY Postnatal Manual FINAL VERSION. c. HAPPY manual resources. [file 1479-5868-10-142-S4.zip › 1523029418990916_add4c1.zip!/Pregnancy eating and drinking facts quiz handout.doc]

**Pregnancy Eating and Drinking Facts – Quiz Answers**

**1.	What you eat affects you and the baby: True or False?**

**True** - a balanced diet and good nutrition prior to conception and throughout pregnancy will ensure the nutritional demands of pregnancy are met and help improve the chances of a successful pregnancy and healthy mum and baby.

**2.	Children with parents who are obese are more likely to be overweight / obese: True or False?**

**True** - if both parents are overweight or obese there is a 46% chance of a child being overweight or obese compared to 16% if both parents are not overweight and obese (2 to 15 year olds): health survey for England 2008.

**3.	It’s important to have full fat milk when you’re pregnant – you need the extra nutrients for your baby: True or False?**

**False** - it is important to have calcium as part of a balanced diet and milk provides a good source of calcium and many other nutrients. Semi skimmed and skimmed milk and low fat dairy products like cheese and yogurt will provide as much calcium (slightly more) as full fat versions without the additional calories. Aim for 2 to 3 portions a day of dairy foods.

**4.	Alcohol can harm your baby: True or False?**

**True** - alcohol is a toxin and if drunk during pregnancy can cause permanent damage to the baby causing problems from mild learning difficulties through to serious birth defects. It can also cause miscarriage or premature birth. It is advisable to avoid alcohol consumption during pregnancy and especially during the first 3 mths. If you do, then limit to 1-2 units once or twice a week. Cutting down on your alcohol whilst pregnant will also help you manage your weight as alcoholic drinks add a lot of calories to your diet. More information about alcohol (and drugs) in pregnancy, how many units are in typical drinks and tips on making changes can be found on these websites**.**

www.nhs.uk/change4life/Pages/pregnancy-and-alcohol.aspx

www.nhs.uk/Planners/pregnancycareplanner/pages/Alcoholanddrugs.aspx

[www.drinkaware.co.uk/tips-and-tools/drink-diary](http://www.drinkaware.co.uk/tips-and-tools/drink-diary)
