# Supplementary material for: Using intervention mapping to develop a culturally appropriate intervention to prevent childhood obesity: the HAPPY (Healthy and Active Parenting Programme for Early Years) study
Source: Int J Behav Nutr Phys Act. 2013 Dec 28;10:142. doi: 10.1186/1479-5868-10-142 (PMC3895739; doi:10.1186/1479-5868-10-142)
Supplement: Additional file 4 — a. MASTER HAPPY Antenatal Manual FINAL VERSION. b. MASTER HAPPY Postnatal Manual FINAL VERSION. c. HAPPY manual resources. [file 1479-5868-10-142-S4.zip › 1523029418990916_add4c1.zip!/Eat well in pregnancy handout.zip!/Eat well in pregnancy handout.pdf]

You and your baby will benefit from:

- Enjoying and eating a variety of foods
- Eating the right amount to avoid gaining too much weight. There is no need to 'eat for 2'. Ask your midwife how much weight you should gain.
- Eating a portion of oily fish each week to help baby's development.
- Trying to drink at least 6-8 mugs or glasses of fluid a day.
- Reducing foods high in fat
- Limiting sugary foods and drinks.
- Being active and exercising for 30 minutes on 5 days of the week. If you haven't exercised before, start with 15 minutes on 3 days of the week and gradually build it up.

HAPPY
Healthy and Active Parenting Programme for early Years
eat well, drink well, play well

Eating Well in Pregnancy

The Eat Well Plate shows you the right balance of nutrients for you and your growing baby. It shows a healthy diet based on fruits and vegetables, starchy foods with smaller amounts of meat, fish, eggs, beans and dairy foods.

Fruits and vegetables. These provide vitamins, minerals and fibre. Have them with meals and as snacks to get your 5 a day. Vitamin C in them will help you absorb iron in other

Bread, rice, potatoes, chapatti, pasta and other starchy foods. These give you energy so try to include them with all meals. Have wholegrain varieties to fill you up.

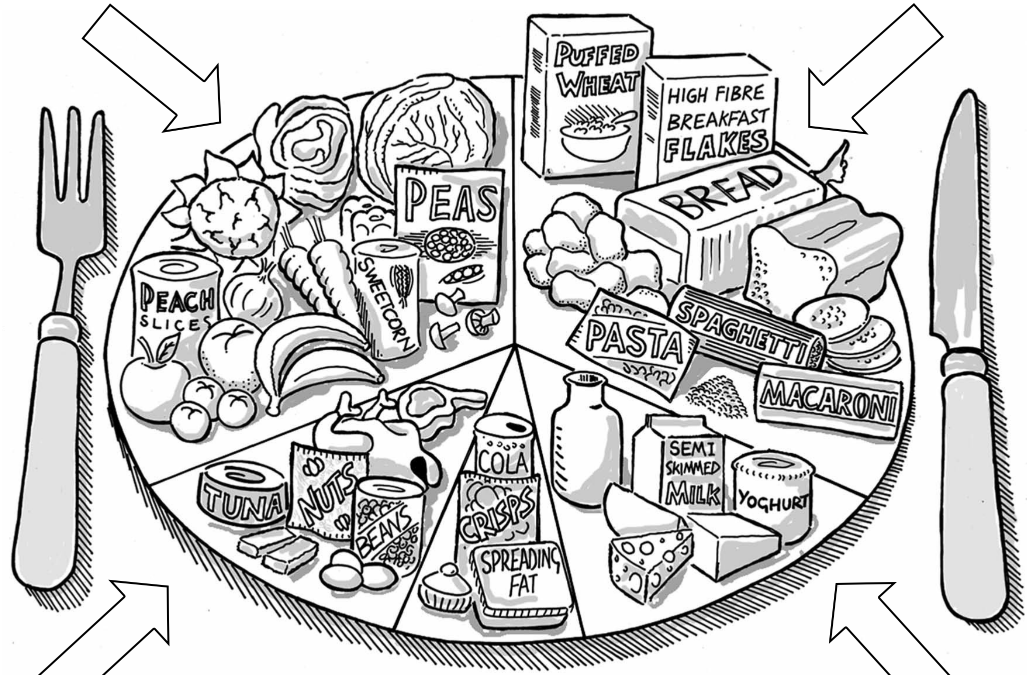

Meat, fish, eggs, beans and other non-dairy sources of protein. Include these foods with 2 meals a day. They are important for growth and development and provide iron for healthy blood.

Foods and drinks high in fat and/or sugar. Try to keep these for occasional treats.

Milk and dairy foods. These are needed to build bones and teeth. Include 2-3 servings a day and choose low fat varieties.

HAPPY
Healthy and Active Parenting Programme for early Years
eat well, drink well, play well

DURING PREGNANCY YOU NEED TO TAKE EXTRA CARE WITH:

Supplements for pregnancy

You should take a 400 microgram tablet of folic acid from when you start trying to conceive until the 12th week of pregnancy. This can help to reduce the risk of neural tube defects such as spina bifida. Some women need a higher dose of folic acid, ask your GP or midwife how much you need.

It is also important to take your Healthy Start vitamin tablets. These contain vitamin D that helps your body to absorb calcium. You need a supplement throughout pregnancy and breastfeeding to ensure you have enough for your baby. Ask your midwife about them.

If you are taking another multi-vitamin preparation you must make sure it is specially formulated for pregnancy.

Foods and drinks to avoid

- **Liver** and foods containing liver such as pate. It may contain high levels of vitamin A that can harm your baby.
- **Shark, swordfish and marlin** as it can contain mercury that is harmful to your baby.
- **Raw shellfish** can contain bacteria that cause food poisoning. All shellfish needs to be cooked thoroughly.
- **Mould ripened cheeses**, such as Camembert and Brie and **blue veined cheeses**, such as Danish Blue.
- **Unpasteurised dairy foods** such as unpasteurised cow's, goat's or sheep's milk and foods made from them.
- **All types of pate**
- **Raw eggs** and foods made with them such as home-made mayonnaise or ice-cream. All eggs need to be cooked until hard right through to avoid the risk of food poisoning.
- **Undercooked meat and poultry** as these can contain a parasite that can cause harm to your baby.
- **Alcohol** is best avoided altogether, especially during the first 3 months, as it can cause permanent damage. Otherwise limit it to 1-2 units, once or twice each week.

Food hygiene

Always wash hands before starting to prepare food and after touching raw meat.

Fruits and vegetables should always be washed before eating.

Food storage – foods that need to be refrigerated should be kept below 5°C. Store raw foods separately from cooked foods.

All foods need to be cooked or reheated until piping hot right through. Take care to thoroughly reheat takeaway and ready meals.

Use by dates are important, don't eat food if it is past this date.

Gardening or handling cat litter – need strict hand hygiene – wear gloves and wash hands thoroughly.

Foods and drinks to limit

Oily fish such as salmon, mackerel, sardines and fresh tuna, needs to be limited to **two portions each week** due to pollutants that can be harmful.

Canned tuna should be limited to 4 medium tins each week.

Drinks with caffeine such as tea, coffee and cola should be drunk in moderation. Limit caffeine to 1 mug of filter coffee, or 2 mugs of instant coffee, or 2 mugs of tea, or 5 cans of cola, or 2 cans of energy drinks. Try de-caffeinated tea and coffee instead.

Healthy Start Scheme

Some families may be eligible for Healthy Start vouchers which can be exchanged for free fruit, vegetables and milk at local shops. Ask your midwife for more information or look on the Healthy Start website *www.healthystart.nhs.uk*

Further information about healthy eating and exercise during pregnancy is available from the NHS Choices website at http://www.nhs.uk/Planners/pregnancycareplanner/pages/Healthinpregnancyhome.aspx.

Information provided by Bradford Nutrition and Dietetics Service, BTHFT 2 12
