# Supplementary material for: Using intervention mapping to develop a culturally appropriate intervention to prevent childhood obesity: the HAPPY (Healthy and Active Parenting Programme for Early Years) study
Source: Int J Behav Nutr Phys Act. 2013 Dec 28;10:142. doi: 10.1186/1479-5868-10-142 (PMC3895739; doi:10.1186/1479-5868-10-142)
Supplement: Additional file 4 — a. MASTER HAPPY Antenatal Manual FINAL VERSION. b. MASTER HAPPY Postnatal Manual FINAL VERSION. c. HAPPY manual resources. [file 1479-5868-10-142-S4.zip › 1523029418990916_add4c1.zip!/Managing your weight in pregnancy handout Tommy's.doc]

Managing your weight in pregnancy

**– REFERENCED VERSION**

(To order the designed and printed version, please visit www.tommys.org/shop)

**Contents**

INTRODUCTION

SECTION 1 HOW DOES MY WEIGHT AFFECT MY PREGNANCY?

SECTION 2 EATING FOR A HEALTHY PREGNANCY

SECTION 3 HOW MUCH WEIGHT GAIN IS OK

SECTION 4 KEEPING ACTIVE IN PREGNANCY

SECTION 5 GETTING STARTED AND KEEPING MOTIVATED

SECTION 6 GETTING YOUR PARTNER AND FAMILY INVOLVED

SECTION 7 BREASTFEEDING

SECTION 8 WEEKLY GOAL GRID

SECTION 9 HELP AND SUPPORT

Acknowledgements

Tommy’s has been certified as a producer of reliable health and social care information by the Information Standard,

First published in 2010 ©Tommy’s. This updated version (V2) published in 2011. The first edition for Northern Ireland (v1) published in October 2012.
Planned review date: July 2013.

For any comments or feedback on this publication, please email mailbox@tommys.org

Fully referenced version available. Visit www.tommys.org/references

All rights reserved. No part of this publication may be reproduced, stored in a retrieval system or utilised in any form by any means, electronic, mechanical, photocopy, recording or otherwise, without prior permission of the publisher.

Disclaimer
Tommy’s has ensured that this book has been checked by experts and to the best of our knowledge the information was accurate at the time of going to printing. Every effort is taken to make sure that advice given here is accurate and appropriate in the context of current UK practices. However, Tommy’s cannot guarantee this and you should not rely purely on advice from us as we will not have had a chance to examine you and therefore can’t make a full assessment of your particular case and circumstances. Information from Tommy’s should not replace the advice of your GP or other healthcare professional. We do not take responsibility for the content of any external websites or other references listed in this document. Whilst we do not seek to exclude our liability if we make any negligent error causing physical harm, we will not otherwise be responsible for any claims arising out of any advice we give or action taken.

**INTRODUCTION**

**This guide is for you if you are pregnant and if you weigh more than is recommended for your height.**

**It’s all about looking after yourself and your growing baby by eating well and keeping active. It has some great ideas for simple things you can do that will make a big difference to your health and your baby’s health.**

To help us write this guide we asked women who were overweight at the start of their pregnancies to share their thoughts and feelings about managing their weight during pregnancy. With their help, and the help of experts in nutrition and exercise, we have put together this guide for managing your weight in pregnancy.

SECTION 1: How does my weight affect my pregnancy?

Before we begin, experts do not recommend weight loss in pregnancy!

**What does managing my weight mean?**

**Managing your weight is not about dieting or trying to lose weight. It’s about looking after yourself and your baby by eating healthily and keeping active. This can help you feel better and limit the extra pounds you might otherwise put on which you may struggle to lose afterwards.**

**Who should manage their weight during pregnancy?**

**EVERYONE!**

It’s important to manage your weight during pregnancy especially if your body mass index (BMI) was 30 or more before you became pregnant^[1]^.

To find out what your body mass index is, visit [www.tommys.org/bmi](http://www.tommys.org/bmi)

**Body mass index and pregnancy**

Your BMI is a calculation that health professionals use to work out whether you are a healthy weight for your height (weight (kg)/ height (m²)). People with a healthy weight have a BMI of between 18.5 to 24.9, people who have a BMI of 25 to 29.9 are ‘overweight’; those with a BMI of more than 30 are ‘obese’ and people who are ‘underweight’ have a BMI of 18.5 or less ^[2]^.

The women who helped us design this guide told us that they do not like to be described as obese as they find the term offensive. However, ‘obese’ is a word that you will find doctors and midwives using. They do this because they need to describe your weight to make sure you get the right type of care during your pregnancy.

Above, a midwife explains why they use the word ‘obese’.

**A MIDWIFE’S VIEW - BOX**

It’s very important that we make a note of clients who are obese because they may need extra special care and advice to help them have a healthy pregnancy. They may also need special facilities during the birth of their baby. For example, a woman who is obese should have the opportunity to see an obstetrician (a doctor specialising in pregnancy), who will work with her midwife to monitor her health and will talk to her about the options for her antenatal care and the birth. The services on offer differ according to the needs of the woman and what is available in her local area. If a midwife or doctor asks you to step on the scales please do not feel they are judging you, they just want to make sure you get the care that is right for you.’

**Why do I need to manage my weight when I’m pregnant?**

If you are a larger pregnant woman, being careful about your weight might help you to:

- **have a more comfortable and enjoyable pregnancy,** enabling you to be more mobile and less likely to suffer skin problems and pelvic pain ^[3]^.
- **reduce the risk of pregnancy complications which can be serious**. These include miscarriage and birth defects; gestational diabetes and pre-eclampsia; complications in labour, including the need for a Caesarean delivery; stillbirth and short and long term health problems for the baby ^[4]^ ^[5]^ ^[6]^.
- **have less weight to lose after you have had your baby,** and could also mean your baby is less likely to develop obesity during his/her life **^[7]^ ^[8]^.**

**What is it?**

**Gestational diabetes (diabetes in pregnancy)** is a condition in which the amount of sugar (glucose) in your blood is too high because your body can’t store it properly^[9]^. This increases the risk that your baby will be larger than normal, which can lead to complications during pregnancy and birth, and health problems for the baby, including the risk of developing diabetes and becoming obese ^[10]^ ^[11]^ ^[12]^.

**Pre-eclampsia** is a condition that can occur when there is a problem with the placenta (the organ that links the baby’s blood supply to yours). As a result, you can develop high blood pressure and protein in your urine. It can also affect the way the baby grows. Most cases are mild, but serious cases can be dangerous and may cause death ^[13]^.

**Will managing my weight now help me after the birth?**

Yes! By eating sensibly in pregnancy you will feel better and will be doing the best for your baby. It also means that you may not have so many extra pounds to lose after the birth of your baby.

**What should I do to manage my weight during pregnancy?**

This is not the time to take on a massive lifestyle challenge. There are lots of little things you can do to manage your weight by **eating healthily** and **keeping active**. The next sections of this guide will show you how.

SECTION 2: EATING FOR A HEALTHY PREGNANCY

It’s hard to think about eating healthily if you are feeling sick or tired, or have food cravings. But there has never been a time in your life when it’s more important to try to eat well. Everything you eat and drink reaches your baby in some way and influences your baby’s health. You should never go hungry and you don’t need to go on a special diet, but you do need to get a good balance of food in order to make sure your baby gets all the nutrients he or she needs.

**Eating for you or eating for two?**

During pregnancy almost everyone is told by some well-meaning person to have a second helping, or to eat more of what they fancy, *‘…because you’re eating for two now’.*

**It’s not true!** Amazingly, your baby takes everything he or she needs from your body. Amazingly, your baby takes everything he or she needs from your body. Up until the third trimester (six months into your pregnancy) your baby grows well without you needing to eat any extra calories at all.^[14]^ In the last three months you may need a little extra food, but only up to 200 extra calories a day, which is about two slices of wholemeal toast and butter^[15]^

Not all calories are equal. Choosing 200 calories of healthy food, such as a low-fat yoghurt with banana, will provide you with essential nutrients, such as calcium, which is important for the growth of your baby. However, 200 extra calories from crisps will provide you with very few essential nutrients.

When such a small amount of food can add so many calories, it is important to keep an eye on the amount of food on your plate. Family and friends might offer you large portions, but it is important to eat in moderation, eat regularly and avoid skipping meals.

Many pregnant women ‘get the munchies’ at times. If you have snacks, try to make sure they are healthy (see our ‘snack attack’ table on page 13).

**Eating a balanced diet in pregnancy**

Getting the right balance of different food types is important to help your baby’s growth and development. Foods are divided into five groups. This ‘plate’ shows the amount of each group you should eat.

**PIC: EATWELL PLATE**

**Portion sizes**

What exactly is a portion? Use the following as a guide

| Food | One portion equals... | And looks like... |
| --- | --- | --- |
| Vegetables | 3 tablespoons | half a tennis ball |
| Salad | 80g | large cereal bowl |
| Fresh fruit | 1 medium whole fruit | whole tennis ball |
| Cooked rice | 2-3 heaped tablespoons | half a tea cup |
| Peas, lentils and beans | 2-3 heaped tablespoons cooked | half a tennis ball |
| Nuts | 2 tablespoons | small handfull |
| Cheese | 30g | small matchbox |
| Meat, poultry, fish | 80g | pack of cards |

**What to eat and why?**

| **Food group** | **Foods in this group.** | **It’s good for you and your baby because...** | **How often should I eat them?** |
| --- | --- | --- | --- |
| Starchy carbohydrates | Bread, rice, potatoes, pasta, porridge, cereals. Wholegrain versions are best. | ...these are packed with energy and are a good source of fibre. | These should make up the main part of every meal. |
| Fruit and vegetables | All fruit and vegetables except potatoes. They can be fresh, frozen, tinned (avoid fruit in syrup), dried or juiced (choose juice without added sugar). | ...these are full of essential vitamins, minerals and fibre. Green leafy vegetables like broccoli and spinach are especially good because they contain lots of folate which is vital for your baby’s development. | Aim to eat at least five portions (one apple, two plums or seven strawberries are examples of a portion) of fruit and vegetables a day. Fruit juice only counts as one portion though, however much you drink! |
| Protein rich foods | Meat, chicken, fish, eggs, beans, pulses, nuts and seeds. * | ...these provide protein, which is vital for your baby’s growth as it builds new tissue for bones, muscles and organs. These foods are also a good source of iron, which is especially important when you are pregnant. Oily fish (sardines, mackerel, salmon, fresh tuna) contain Omega 3 fatty acids, which help the baby’s nervous system to develop. | Aim to include protein rich foods in eat at one to two meals a day. Try to choose lean varieties of meat. Remember to cook these properly and avoid liver, liver products and pâté when pregnant. Limit oily fish to two  portions a week (the  same as four medium  tins of tuna). |
| foods and drinks high in fat and/or sugar | Sweets, crisps, cakes, biscuits, chocolate, fizzy drinks. | They provide very few essential nutrients. Try to avoid saturated fats, found in foods such as biscuits, crisps and chips.  Try to eat unsaturated fats (good fats) such as those found in vegetable oils and spreads, seeds and nuts* | Only in small amounts as they are not needed for a healthy diet – keep these for occasional treats!  Try to eat unsaturated fats (good fats) such as those found in vegetable oils and spreads, seeds and nuts. |
| Milk and Dairy Foods | Milk, cheese, yoghurt. | ...these are a good source of calcium which helps your baby’s bones grow strong, as well as other important vitamins and minerals. | Try to have two to three portions a day, but choose low-fat versions. |

*** Not to be eaten if you have a nut allergy.**

**Stop and swap**

Sugar and saturated fat (fat found in butter and animal fats) are hidden in many of the foods that we eat. It’s important to avoid these because eating a diet high in fat or sugar is more likely to make you put on extra weight^[16]^. Being heavier in pregnancy increases the risk of complications for you and your baby.^[17]^. You need to make smart choices about what you eat and drink, for example by:

- aiming to swap bad fats for good fats
- aiming to swap sugary food and drinks for sugar-free alternatives

**Swapping some of the following foods for low-fat and sugar-free alternatives will help your health and your baby’s health.**

| **Instead of...** | **Try...** |
| --- | --- |
| - Fizzy drinks - Squash, cordials, juice drinks containing sugar - Whole milk | - Water (still, sparkling or tap water) - Fruit or herbal tea - Sugar-free flavoured water - Sugar-free or low calorie (diet) fizzy drinks* - Sugar-free (or “no added sugar”) squash or cordials - Semi-skimmed or skimmed milk |
| - Sugar (white or brown) | - Cutting down the amount you use - Lower calorie sugar - Artificial sweeteners* (liquid, tablet or granulated) |
| - Butter, jam, marmalade or honey** on toast | - Low-fat spread, reduced-sugar jam, sliced banana on toast |
| - Cake or biscuits | - Currant bun (no icing) or fruit loaf, oatcakes, wholegrain cereal bar or fruit |
| - Tinned fruit in syrup | - Tinned fruit in juice, fresh or frozen fruit |
| - Breakfast cereals coated with sugar or honey | - Traditional porridge oats or wholegrain cereals without sugar or honey coating, sugar-free muesli or Shredded Wheat |
| - Sausages, burgers, nuggets, kebabs, pies or other processed meats | - A lean cut of meat or chicken - Fish (oily or white) - Beans and pulses (e.g. reduced salt and sugar baked beans, chickpeas, kidney beans, lentils) |
| - takeaway chips | - New boiled potatoes, baked potatoes, oven chips made at home |
| - Full fat mince | - Lean mince, with fat strained off when cooking |
| - Streaky bacon or smoked bacon | - Unsmoked, back bacon with fat trimmed off |
| - Full fat milk | - Semi-Skimmed or Skimmed milk |
| - Full fat cheese (e.g. cheddar) | - Half fat or reduced fat cheese - Edam or cottage cheese |
| - Creamy or cheesy sauce | - Tomato or vegetable based sauces |
| - Butter on bread | - Lower-fat spread on bread. Look for the ones labelled ‘low in saturates’ or ‘low in saturated fat’ or ‘high in monounsaturates and polyunsaturates’ |
| - Butter, lard or ghee in cooking | - Vegetable, olive or rapeseed oil |
| - Cream or coconut cream | - Reduced-fat yoghurt, reduced-fat crème fraiche |
| - Ice cream | - Frozen low fat yoghurt |
| - Speciality coffees (e.g. latte, cappuccino) made with full fat milk *** - Hot chocolate, instant malt drinks | - Decaffeinated coffees made with skimmed milk (known as “skinny”), or fruit/ herbal tea |

*There are no specific recommendations on avoiding artificial sweeteners in pregnancy although some women may choose to avoid them.

** NHS Choices recommends that honey is fine for pregnant women but not suitable for babies under a year old.

*** NHS Choices recommends that it's best not to have more than 200mg of caffeine per day when you're pregnant which is roughly equivalent to 2 mugs of instant coffee or 2 mugs of tea

**Food to keep you satisfied for longer**

As well has having regular meals it is important to choose foods that release their energy slowly rather than converting quickly to sugar^[18]^.

There are lots of foods that you can include in your meals that release their energy slowly, these include:

- multigrain or granary bread and toast
- rice that is NOT easy cook –basmati rice is the best
- potatoes – new, boiled, baked (eat the skin!), or sweet potato.
- pasta

**Ease up on the fruit juice**

Women often think that fruit juice is the healthiest drink option in pregnancy, however you should aim to have no more than one glass a day. Fruit juice can be high in natural sugar, which can make your blood sugar levels rise and fall again rapidly. Choose fresh juice with pulp where possible and avoid those with added sugar or ‘made from concentrate’.

**Start right with breakfast**

Many people skip breakfast because they think they don’t have time for it but studies have shown that women who have breakfast are more likely to be able to control their weight than those who don’t^[19]^. Our bodies have been fasting while we are asleep, and if we don’t eat in the morning we will feel tired and hungry and are more likely to overeat later. If you’re not used to having breakfast, here are some ideas to try:

- porridge (not instant) with a chopped banana
- sugar free cereals such as Shredded Wheat or sugar-free muesli with skimmed or semi-skimmed milk (add some chopped or dried fruit to give some natural sweetness)
- low-fat spread or low-sugar jam on granary toast
- fresh fruit salad with a low-fat yoghurt
- cereal bar made from wholegrains
- poached egg* on multigrain or wholegrain toast

* NHS Choices recommends that you should only eat eggs cooked enough for both the white and the yolk to be solid ^[20]^.

*Tip*

If you normally add sugar to your coffee, tea or cereal, try cutting down by a tiny amount each day. If you do this gradually you give your taste buds time to adapt, and soon you’ll find the unsweetened taste just as delicious.

*Tip*

If you feel too sick to eat first thing in the morning, or you’re really pushed for time, try making a packed breakfast the night before to take to work or choose a wholegrain based cereal bar without added sugar.

**Prepare for snack attacks**

Many of us when find that when we are pregnant it is more comfortable to eat little and often. But when we feel like a snack it’s often easiest to reach for the biscuits, chocolate or crisps. However, while they may be quick, they aren’t usually healthy.

Making smart choices about your snacks can help to keep you feeling fuller for longer.

**Try swapping some of your normal snacks for these slow energy release alternatives:**

| ***Instead of this ……..*** | ***Try this……………*** |
| --- | --- |
| - Chocolate and sweets - Crisps - Biscuits, cookies, buns, cakes, sweet pastries and doughnuts - Full fat yoghurt | - Cereal bar based on oats (but not brands with lots of fat and sugar) - Small bag of unsalted nuts or mixed fruit and nuts - Oatcakes or rye crisp bread, with lower-fat spread, Fruit loaf or bun (no icing), Plain semi-sweet biscuit e.g. rich tea, plain digestive or fruit oat biscuit - Fresh fruit e.g. banana, apple, grapes, pear, orange, strawberries, raspberries, cherries, blueberries, peach, pineapple, plum; raw chopped vegetables, low-fat yoghurt |

**Be supermarket savvy**

One of the easiest ways to help you and your baby be healthy is to shop smart.

- Don’t go shopping when you’re hungry – few of us can resist our favourite treats if we’re already hungry.
- Make a shopping list and promise yourself to stick to it, even if that means walking past a ‘2 for1’ bargain.
- Follow the swaps in this guide and fill your basket with tasty, healthy food choices that nourish your baby.
- Shop online rather than visiting the supermarket – it will help you resist temptations as well as saving time!

**Become a label reader**

Often, the packaging of foods can help you decide whether it is a good choice. Most food packaging now comes with the Guideline Daily Amount (GDA). This tells you, at a glance, how much of your daily limit of sugar, fat, saturates and salt is present in a portion of the food.

Something else you will see a lot on packaging is the word ‘Light’; but what does it actually mean? To be ‘light’ the food must be at least 30% lower in at least one value, such as calories or fat than the standard version of the same brand. The label must explain exactly what has been reduced and by how much, for example ‘light – 30% less fat’. Keep in mind that a ‘light’ or ‘lite’ version of one brand of crisps may contain the same amount of fat or calories as the standard version of another brand.

Some foods make claims in big letters on the front, such as ‘No added sugar!’ or ‘Low fat!’, but don’t be fooled – foods that are low in sugar may be high in fat, and foods that are low in fat may be high in sugar. Always look at the ingredients list on the back of a packet.

**The traffic light system**

Many foods now have traffic light labels on the front of the pack to show you at a glance if the food has high, medium or low amounts of fat, saturated fat, sugar and salt, helping you to make healthier choices. You should look for labels that are mainly green, avoiding labels with a red section.

**Healthier takeaways**

If you are buying fast food or takeaways, think about making the best choice for you and your baby.

Takeaway and fast food is often:

- very high in saturated fat, salt and sugar
- larger than your normal portion size
- more expensive than a homemade version of the same meal.
- Keep takeaways as an occasional treat, or make your own version at home.

If you are buying a takeaway, there are still healthy choices you can make:

- **Burgers and chips** Ask for them without cheese or mayonnaise and ask for extra salad instead. You could try chicken, fish or vegetable burgers rather than beef. Try to avoid the chips or, if you really want them, have some oven chips at home instead.
- **Pizza** Choose pizzas without extra cheese or cheese crust stuffing. Choose healthy toppings such as peppers, mushrooms or chicken instead of pepperoni or salami.
- **Indian** Swap meals with creamy sauces (such as korma) for sauces based on yoghurt or tomatoes (such as jalfrezi), or those without sauces (such as tandoori). Swap naan bread for chapatis. Have a side dish of vegetables or dhal (lentils or pulses) and split a main between two.
- **Chinese** Choose plain boiled rice, and pick a stir-fry that includes vegetables. Avoid the deep fried dishes, such as prawn toast and spring rolls.
- **Kebabs or fish and chips** These have very high levels of salt and saturated fat. Keep them as an occasional food.

**Don’t forget your vitamins**

You can get most of the vitamins needed for a healthy pregnancy by eating a tasty, healthy balanced diet as described in this guide, but there are a couple of vitamins that need particular attention when you’re pregnant:

***Folic acid*** is a vitamin that helps in the early formation of your baby’s nervous system. Lack of folic acid can cause spina bifida, where the baby’s spine and nerves do not form properly in the womb ^[21]^. Some foods contain folic acid naturally (it’s called folate when it’s in food), such as broccoli, green beans, oranges, chickpeas and brown rice; and some breakfast cereals have folic acid added. But food won’t give you enough folic acid to protect your baby, so it’s important to take a folic acid supplement of 400 micrograms (400mcg) every day for the first 12 weeks of pregnancy. For women with a BMI higher than 30, a supplement of 5 milligrams (5mg) is recommended.^[22]^

***Vitamin D*** protects your baby from the bone disease rickets ^[23]^. It’s found naturally in some foods such as eggs and oily fish, and is sometimes added to breakfast cereals and margarine. The main source of vitamin D is produced in our skin after being in the sun (not sunbathing!) – just 15 minutes in the sun, two or three times a week will be enough. If you have dark skin, or always cover your skin, or if it’s winter, you will be more likely to need a vitamin D supplement. Current recommendations are for all pregnant women to have a 10 micrograms (10mcg) supplement of vitamin D each day. Ask your midwife or GP for more information^[24]^.

*Tip*

If you want to get your folic acid and vitamin D from a multi-vitamin, make sure you choose one that is sold especially for pregnant women.

*Tip*

Always forgetting to take your vitamins? Keep the bottle somewhere you will see it every day, next to your toothbrush for example.

**Healthy Start**

If you receive Income Support, income-based Jobseeker’s Allowance, or Child Tax Credit, or you are under 18, you may be eligible for the Healthy Start scheme. Healthy Start gives you **free vouchers** to spend on **fruit, vegetables or milk** each week and **free folic acid, vitamin D and vitamin C**.

Ask your midwife for an application form if you think you might qualify, or call 08701 555 455 or visit www.healthystart.nhs.uk.

**Is this you?**

(thought bubbles around woman in picture)

- I’m too queasy to eat!
- Fruit and veg are so expensive!
- I don’t know how to cook!
- I’m too tired in the evening to start cooking meals, it’s easier to get a takeaway!
- If I crave a food, I’ve got to eat it!

If this is you, don’t give up on eating healthily! Remember that:

- **Morning sickness doesn’t last forever.** Listen to your body and eat when you can. Some studies suggest eating a little as soon as you wake up and before getting out of bed^[25]^. And for some women, ginger or foods containing ginger can help. There is also a link with being tired and morning sickness so rest, when you can^[26]^.
- **Fruit and veg don’t have to be expensive**. Eat fruit and veg when it’s in season (that’s also when they taste best) and look out for supermarket offers on fruit and veg. Remember fruit and veg that are frozen are just as good for you and are often cheaper.^[27]^ So are fruits tinned in juice (not in syrup), and tinned vegetables without added salt or sugar.
- **Food cravings can be good for you**. Lots of pregnant women get food cravings, but they are not always for unhealthy foods – some people crave fruits like oranges. If you are worried you could discuss any food cravings you have with your midwife.
- **Not all takeaways are equal**. See the section *Healthier takeaways* to make better choices for you and your baby, whatever you are eating.

**There are some foods and drinks that are important to avoid during pregnancy. These are listed in Tommy’s guide ‘Having a Healthy Pregnancy’, which you can order from the Tommy’s website. You can also find the list of foods to avoid on the website itself:** [**www.tommys.org/what-to-eat**](http://www.tommys.org/what-to-eat)

SECTION 3: HOW MUCH WEIGHT GAIN IS OK

Doctors in the UK are still debating how much weight gain is ok in pregnancy ^[28]^. All of us gain some weight when we’re pregnant, especially at the end of pregnancy, and each of us is different, that is why doctors here find it hard to say how much weight gain is OK for each person.

In America, guidelines suggest that women who are overweight at the start of their pregnancies (or have a Body Mass Index (BMI) of 30 or above) should not put on more than 9kg or 1stone and 6lbs (as recommended by the Institute of Medicine) ^[29]^. But the important thing is to keep weight gain to a safe and healthy level for you and your baby: Your doctor or midwife may be able to advise you on what’s right for you.

SECTION 4: KEEPING ACTIVE

**Looking after yourself and your baby by eating well is only half the story. Keeping physically active is great for you and for your baby. Experts advise that in most cases physical activity is not only safe but also improves the health of both the baby and the mother^[30]^.**

Motherhood is the biggest workout of your life, but you can prepare your body for the challenges of pregnancy, labour and childcare by staying active and keeping yourself strong and healthy.

If you weren’t very active before your pregnancy, don’t worry – there are lots of small changes you can make to your everyday lifestyle that will make a big difference to your health and your baby’s health^[31]^. Physical activity means any activity you do in your daily routine, including walking, and not just planned or organised exercise.

**Benefits of being active ^[32]^**

- It makes you feel good and gives you more energy. You are less tired and sleep better.
- It improves and strengthens muscles, so you’ll have fewer aches and pains. You may also have less chance of varicose veins and swollen legs.
- It will help improve your blood sugar control and may help prevent you getting gestational diabetes.
- It will increase your stamina, which will help you and your baby through labour.
- It will help you to recover more quickly from your baby’s birth.
- It means you are less likely to hang on to those extra pounds after you have had your baby.

**When you need to take care**

You should ask your midwife or doctor for advice about physical activity:

- if you have any serious medical conditions or health problems.

- if you have had complications in a previous pregnancy.

- if you bleed vaginally at any time and any amount.

- if you are told that your placenta is low on your ultrasound scan at around 20 weeks.

- if you develop high blood pressure.

- if your ‘waters’ break early.

- if you go into labour early.

**What activities can I do?**

**1) Everyday activities**

- Walking is probably the easiest way to fit activity into your already busy life. This is a good type of activity for everyone.
- Walk rather than catching the bus or driving the car. Popping to the shops or strolling through the park count too.
- If you have children, try to walk when you take them to playgroup or school.
- If you have a dog, he/she won’t mind going for an extra walk.
- Climb the stairs rather than take the lift. Or take the lift part way!
- Keep busy around the home. Vacuuming or doing the laundry to your favourite tunes will give you a good workout, as long as you keep moving.
- If you have a garden, take some time to mow the grass, add a new plant or get those weeds out.
- When shopping carry the lighter bags to exercise your arms (remember to keep your tummy muscles held in and your back straight if you bend to pick them up). You will have to lift your baby and carry him/her around after the birth, so you’ll need strong arms – even for a newborn 7lb (3kg) baby.
- If you like cycling, you can carry on with this throughout pregnancy, but remember to always wear a helmet.
- Go for a walk or bike ride with your partner, family or friend at the weekend.
- Gentle stretching will help you to stay flexible.

**2) Organised or group activities**

Keep doing any activity that you were doing before pregnancy as long as it feels comfortable (and is not listed below as activities to be avoided during pregnancy) ^[33]^. If you regularly attended a class, speak to your instructors and tell them you are pregnant; they will be happy to advise you.

Swimming or exercising in water is really good in pregnancy as the water supports your bump, taking the strain off your back. It helps your muscles too.

If you like the idea of joining in a class with other pregnant women, ask your midwife, leisure centre or children’s centre what’s going on in your area. Look out for these types of classes:

- Aqua-natal – pregnancy exercises in a swimming pool. You don’t normally have to be able to swim to join in.
- Pregnancy exercise – some gyms offer special exercise classes for pregnant women.
- Yoga – not all yoga exercises are suitable for pregnancy, so it’s a good idea to join a class for pregnant women.

**3) Try to keep moving**

Any kind of activity or movement is more beneficial than just sitting or standing for long periods.

Try to limit long periods (more than 30mins) of sitting still or lying down (e.g. watching TV, using the computer or reading a book). Get up and do something at every advertisement break.

**Activities to avoid**

- Don’t exercise on your back after 16 weeks into your pregnancy as the weight of your baby may press on major blood vessels and reduce the blood and oxygen flow to the baby^[34]^.
- Avoid activities that have an increased risk of falling or excessive joint stress (e.g. running down stairs or escalators, running for the bus, horse riding, skiing, and step aerobics including jumps) or physical contact (e.g. many vigorous ball games). Scuba diving is also not recommended for pregnant women.

**How much should I do?**

Experts recommend that pregnant women (like all healthy adults) should do at least 30 minutes of moderate physical activity every day, but it is safe to do as much moderate activity as you like ^[35]^. This is anything that makes your breathing and heart rate increase, but you are still able to hold a conversation.

If you weren’t this active before pregnancy, it’s a great time to start now. Start at a level that suits you and build it up gradually. You can for example start off doing 15 minutes of continuous activity three times a week and increase it to 30 minutes four times a week ^[36]^. If possible you are aiming to get to 30 minutes of activity every day. Continue being active as long as possible, until the end of your pregnancy if you can – and beyond!

**Remember**

- Stay as active as you can – how you do it is up to you.

- Do something you like, so it is enjoyable.

- Ask a friend or family member to join you.

- Use your favourite music to help you keep active.

**Top tips**

- Wear shoes that support your ankles. Low heels or no heels are best. High heels throw your body weight forward and put more stress on your back, leading to aches and pains and so should be avoided throughout pregnancy.
- Wear a good supportive bra.
- Loose comfortable clothing can stop you getting too hot.
- Remember to drink water. Drink a glass of water before you go out and take a bottle of water with you. This is especially important when the weather is warm, but you need to make sure you drink enough, even on the coldest days.
- On hot days walk in the early morning or evening, when it is cooler.
- If you have other children, try activities that they can join in with, like playing in the park.
- Plan some activities you can do indoors, if the rain stops you getting out – we’ve given you some ideas below (‘five for fifteen’).

**Caution: Don’t exercise:^[37]^**

- to exhaustion.

- if it is very hot.

- when you feel unwell.

- if you have deep pelvic pain.

- if you have any vaginal bleeding.

- if you are getting lots of tightenings.

- if your doctor or midwife has told you to stop exercising.

**I HAVE SOME QUESTIONS!**

**‘I feel so tired all the time and do not feel like being active!’**

Most pregnant women have times when they feel incredibly tired, but many find that having made a small effort they feel better for it. Walking even a short distance counts. After you have walked 5 minutes you might feel a little more energetic and perhaps you could walk a little further. If not, don’t worry- the short walk is better than nothing!

**‘I’m working full time and when I come home I need to look after my family and the household. I simply can’t find time to be more active!’**

Could you walk part of the way to work? Or walk to pick the children up from school? How about a 10 minute walk at a coffee break or at lunch time? Remember that the heavier household jobs, like vacuuming and active playing outside with children (or pets) also count as physical activity.

**‘Exercising is uncomfortable as I have back pain and my feet are swelling. Isn’t it better just to put my feet up and relax on the sofa?’**

Physical activity has been shown to help. It tones muscles, reducing backache and improves circulation, which reduces swelling^[38]^. Swimming, in particular, may be comfortable for you because everyone weighs less in water. If you have severe or persistent back pain, please speak to your midwife for advice; she may refer you to a physiotherapist.

**‘I’m concerned about having a miscarriage and don’t want to take any extra risks by over-exercising.’**

If you have already passed the 14th week, the risk of miscarriage is very low. The things we are asking you to do are unlikely to cause any problems and should make you feel better. There is lots of evidence that babies whose mums are active are healthier than those who are not^[39]^.

**‘I like playing exercise games such as the Wii Fit. Can I still play them when I am pregnant?’**

Experts say in general the WII Fit and other similar games can be a good way of keeping fit^[40]^. There are no current recommendations for the use of these in pregnancy. You can continue playing them as long as you feel comfortable and don’t play to exhaustion. Avoid any activities done on your back or activities where you could lose your balance.

TOP TIPS

**Keep your pelvic muscles strong**

Your pelvic floor muscles support and protect your bowels, womb and bladder. Hormonal changes in pregnancy loosen these muscles and, with the added pressure of the baby, women sometimes leak urine when they exercise, cough or laugh. Exercising your pelvic muscles can help you control this and will help you with your birth and recovery.

To find your pelvic floor muscles imagine trying to stop your urine flow when you pee – the muscles you tighten to do this are your pelvic floor muscles. Once you know where they are, you can tighten them any place any time: on the bus, out shopping or collecting the kids – no one will know! Try to do five lots of pelvic floor squeezes five times a day and build up to doing ten squeezes five times a day.

**‘Five for Fifteen’**

*Five activities you can fit into fifteen minutes of your daily life*

These are five exercises that you can fit into your everyday life, while you’re washing up or watching the TV. If you are not very active they are a great way to get going. Try to do them for fifteen minutes to start with and build up from there. Add some walking, housework or other activities suggested on page 19 and you will really start to feel the benefits. If you are already active, these exercises are a great way to boost to your activity levels and keep you flexible.

**‘Zip Up’ before you start**

*It is essential that before any exercise or movement which exerts you (sitting, standing, lifting, carrying, walking etc) to ‘zip up’. This simply means strengthening the core muscles to protect your back and stomach from injury.*

It’s easy to do; just imagine a thread is attached to your belly button from the inside and it is pulling your belly button towards your spine. If you put your finger on your belly button and tighten your muscles up and inwards this way you will feel belly button move upwards slightly and the muscles around it will tighten, but you will still be able to breathe normally. Now you are zipped up and protected.

**Sink stretches**

*Can be done whilst washing dishes or waiting for the kettle to boil.*

Stand feet hip width apart, toes knees and hips facing forwards. ‘Zip up’ and then slowly lift yourself up onto your toes, feeling the muscles in the lower leg working. Hold for a count of 3 and then slowly lower back down. Repeat 15-20 times, rest for 30 seconds and perform another 15-20.

**TV toners**

*These are great for during the advert breaks!*

While sat on the sofa make sure your feet are hip width apart, and again with your toes, knees and hips facing forwards. ‘Zip up’ and then slowly stand up, taking your weight through the heels of your feet and keeping your shoulders back and down. Keep your pelvic floor and abdominals nice and tight. Once you are standing (with knees slightly bent) slowly lower yourself back down into the chair with your bum pushed out towards the back of the seat, sit down and repeat. Repeat this 6 times, take a break of a 30 seconds and gradually work up from there.

**Chair legs**

*Seated leg extensions that are great for the muscles at the front of the thighs.*

Sit on a chair, feet hip width apart, toes knees and hips facing forwards, shoulders back down and ‘zip up’. Lift one knee to a comfortable height, keeping the leg bent. Keeping the thigh lifted, slowly straighten the leg without locking out the knee. Then slowly return the leg back to the bent position whilst keeping the thigh lifted. Perform 8 -12 on one side and then do the other. This is a great activity to do if you are sat at an office desk all day.

**Kitchen lifts 1**

*These can either be done with products in your kitchen while you wait for dinner to cook or the kettle to boil. You can try them seated or standing.*

Stand up straight, feet hip width, toes knees and hips facing forwards. You can use bags of sugar, tins of beans or bottles of water. Hold one in each hand, arms by your side and palms facing up. ‘Zip up’ and then slowly tense the muscles in the top of the arms and bring your hands up to touch the front of your shoulders. Keep ‘zipped up’ and then slowly lower back down to the start position. Repeat 12-15 times, rest for 30 seconds and repeat.

**Kitchen lifts 2**

*These can also be done with when whiling away time in the kitchen. You can do them seated or standing.*

Keep shoulders back and down, chest proud, and back nice and tall. You can use two bags of sugar, tins of beans, or bottles of water. Hold one in each hand, arms by your side. Straighten one arm above the head, ‘Zip up’ and then keeping the arm as close to the ear as possible, bend at the **elbow only** and drop the hand behind the head, feeling the muscles in the back of the arm working. Hold for a count of 2 and then (keeping the arm next to the ear) slowly lift the arm back up to the start position straight over the head. Repeat nice and slowly 12-15 times with one arm and then the other.

SECTION 5: GETTING STARTED AND KEEPING MOTIVATED

It’s important to get started with looking after yourself and managing your weight as soon as possible to get the maximum benefits. But where should you start, and how should you keep going on bad days? The following tips are designed to help you on your way.

**Tip 1 Make a plan**

Research has shown that people who have step-by-step plans to help them manage their weight are more likely to be successful ^[41]^. You need to choose some practical things you can do today, tomorrow, this week. The clearer you are about what you plan to do, the more likely you are to do it!^[42]^

**Tip 2 Give yourself goals you can manage**

Start by choosing the easiest things to change and make the changes very simple. To get you started you could try the goal grid in the back of this guide. It gives you some ideas about small changes you can start off with that will make a big difference to helping you feel great and manage your weight.

**Tip 3 Reward your successes**

It’s a great feeling to succeed at a goal that you have set yourself. For many people that feeling is enough to motivate them to carry on, but you could also reward yourself when you stick to your plans. Choose something you like that doesn’t involve food or drink, for example buy yourself your favourite magazine, have a bubble bath, watch a DVD you love, ask your partner to give you a foot rub or splash out on a manicure. It’s up to you!

**Tip 4 Don’t punish yourself**

Everyone has days when they just don’t feel like doing much or when they ate those biscuits that they didn’t mean to. If this happens to you, try not to get angry with yourself or feel that you can’t do anything right. Negative feelings are likely to make you feel low and in the end that is more likely to make you reach for the snacks or give up your daily walk. Believe in yourself, and remind yourself that tomorrow is another day. You could try writing down the reasons that you are trying to be healthy, look at these when you are feeling negative to remind you why it is worth the effort.

**Tip 5 Be aware of emotional eating^[43]^**

One of the most important things you can do to succeed in managing your weight is to stop and think before you reach for your favourite between-meal snack. Ask yourself before you eat anything: am I really hungry? Or is something bothering me? Or have I just got used to having a snack at this time of day? Or am I just bored?

Being aware of why you eat is a great step forward! To remind yourself to stop and think, why not put a sign for yourself on your fridge or food cupboard that asks ‘Am I really hungry right now?’ Or tape up a scan picture of your baby so you always think about making a good choice for you both.

**Tip 6 Develop a distraction**

If you find yourself craving a food when you know that you aren’t hungry, try doing something else to distract yourself. You’ll need to find an activity lasting around 20 minutes to let the craving go down, for example doing housework or ironing or making a phone call, emailing a friend, going for a walk or reading a book. It doesn’t matter what it is as long as it takes your mind off food!

**Tip 7 Get active with a friend**

Keeping active with a friend is more fun than on your own and most people find they are more likely to stick to an activity if they do it with someone else^[44]^. A weekly walk or a trip to the local pool is a great chance to have a catch up.

SECTION 6: GETTING YOUR PARTNER & FAMILY INVOLVED

**Get your family and friends onside**

Many people don’t know that keeping active when you’re pregnant is safe and is actually really important for you and your baby. Your family or friends may encourage you to ‘take it easy’ or to ‘eat for two’ because they think they are giving you good advice.

Show them this guide and ask them to support you in doing the best for your baby and yourself by keeping active and eating well. In particular you may need your family’s support if you decide not to buy so many ‘treat’ foods like crisps, biscuits and chocolate – your family may be used to having them in the cupboard, but it’s so much easier to keep to your good intentions if you just don’t buy them. And why not ask your family and friends to make keeping active more fun for you by joining in!

**Exercise you might enjoy with family and friends**

- Walking the kids to school or the park
- Swimming at the local pool
- A brisk walk round the shops with your girl friends
- Walking after a family meal
- Ball games with the kids
- A night out dancing

# SECTION 7: SOME HEALTHY AND EASY MEAL SUGGESTIONS

## Breakfast

- Scrambled eggs on wholemeal toast
- Kedgeree (boiled basmati rice mixed with hard boiled eggs and smoked mackerel – all mixed with a spoon of curry paste and garnished with parsley) Also suitable for lunch.
- Wholewheat sugar-free cereal with milk, natural yoghurt and banana

## Lunch

- Soup with wholemeal toast/bread
- Basmati rice salad, with small cubes of feta, red pepper and chicken. Add cherry tomatoes, quartered.
- Baked potato, with sugar-free baked beans and a sprinkling of grated cheese
- Potato salad with low-fat mayonnaise, mixed with hard boiled egg and tinned tuna. Serve with lettuce and tomato slices.
- Pasta salad (cooled cooked pasta), with a small amount of olive oil, baby spinach leaves, basil, grilled cherry tomatoes and toasted pine nuts.

## Dinner

- Grilled fish with new potatoes and steamed vegetables
- Grilled chicken and vegetable kebabs (lighly brush with oil) with basmati rice
- Homemade burgers with homemade wedges (chop potatoes, toss in a small amount of oil and bake until crisp)
- Pasta with a shop-bought tomato-based sauce (add tinned tuna in spring water and some chopped cooked peppers for extra taste). If you have the time, make your own tomato sauce with tinned tomatoes, onions and garlic

## Snacks

- Banana with oatcakes or rice cakes
- Crudités (raw vegetables) with two spoonfuls of dips (hummus, tzatziki)
- Yoghurt, with a handful of nuts/ seeds and some fresh fruit
- An apple, with a matchbox sized piece of cheese

SECTION 8: BREASTFEEDING – GREAT FOR YOUR BABY AND GREAT FOR YOU

Your breast milk is the only food designed perfectly for your baby, giving him or her absolutely everything she needs for the first six months, and breastfeeding has lots of benefits for your baby and for you ^[45]^.

There are very few women who cannot or should not breast feed, but the early days can be tough! After all it is a new skill that both you and your baby need to learn. Ask your midwife for help and look for breast feeding café’s and postnatal support groups in your area. Once established, breast feeding is easy and convenient, without the hassle of preparing bottles and always making sure you have enough milk with you when going out.

**What does breastfeeding have to do with managing my weight?**

Breastfeeding is a really important part of managing your weight after you have your baby. The longer you breastfeed, the more weight you lose!^[46]^ When you are pregnant, your body automatically stores some fat to give you a store of energy for making breast milk. Once you have given birth, if you breastfeed your baby this energy store will be used up gradually and you will lose that weight without even trying. If you choose not to breastfeed, your body is left with this unnecessary store of fat which many women find really hard to shift. There is growing evidence that breast feeding can help prevent your baby getting overweight in childhood and as an adult^[47]^.

**How long should I breastfeed my baby?**

Every day that you breastfeed your baby is a plus for both of you. Doctors recommend that you should breastfeed your baby exclusively (with no other food or drink) for around the first six months. After that, you can carry on breastfeeding alongside the solid food for as long as you like. In reality many women stop exclusively breast feeding before this time usually because they need to return to work, but even breast feeding once or twice a day is beneficial for you both. Talk to your Health Visitor or visit your local baby clinic for advice about this.

**To find out more about breastfeeding, including what to eat and where to get support, go to the *Help and Support* section of this guide.**

SECTION 9: GOAL GRID

**Weekly Goal Grid**

| **What** | **Which days ?** | **Starting from** | | **My weekly reward** | **What will stop me** | **What will help me achieve this goal?** | | |
| --- | --- | --- | --- | --- | --- | --- | --- | --- |
| I swap my fizzy drinks for water or sugar free cordial | (Please tick)  Monday □  Tuesday □  Wednesday □  Thursday □  Friday □  Saturday □  Sunday □ |  | (Please tick)  A big bubble bath  My favourite magazine  Trip to the cinema  Rent that DVD I want to see  My own reward idea is……………………………………………. | |  | |  | |
| I will eat one of the healthy breakfast options from this guide | (Please tick)  Monday □  Tuesday □  Wednesday □  Thursday □  Friday □  Saturday □  Sunday □ |  | (Please tick)  A big bubble bath  My favourite magazine  Trip to the cinema  Rent that DVD I want to see  My own reward idea is……………………………………………. | |  | |  |  |
| I will do the hovering and get my heart rate up | (Please tick)  Monday □  Tuesday □  Wednesday □  Thursday □  Friday □  Saturday □  Sunday □ |  | (Please tick)  A big bubble bath  My favourite magazine  Trip to the cinema  Rent that DVD I want to see  My own reward idea is……………………………………………. | |  | |  |  |
| I will go for a brisk walk with a friend | (Please tick)  Monday □  Tuesday □  Wednesday □  Thursday □  Friday □  Saturday □  Sunday □ |  | (Please tick)  A big bubble bath  My favourite magazine  Trip to the cinema  Rent that DVD I want to see  My own reward idea is……………………………………………. | |  | |  |  |
| I will do the ‘Five for Fifteen’ work out in this book | (Please tick)  Monday □  Tuesday □  Wednesday □  Thursday □  Friday □  Saturday □  Sunday □ |  | (Please tick)  A big bubble bath  My favourite magazine  Trip to the cinema  Rent that DVD I want to see  My own reward idea is……………………………………………. | |  | |  |  |
| I will pack a healthy snack in my handbag (see options in this guide) | (Please tick)  Monday □  Tuesday □  Wednesday □  Thursday □  Friday □  Saturday □  Sunday □ |  | (Please tick)  A big bubble bath  My favourite magazine  Trip to the cinema  Rent that DVD I want to see  My own reward idea is……………………………………………. | |  | |  |  |
| I will …………………………………………….  ………………………………………………………  ……………………………………………………..  Set your own goals here | (Please tick)  Monday □  Tuesday □  Wednesday □  Thursday □  Friday □  Saturday □  Sunday □ |  | (Please tick)  A big bubble bath  My favourite magazine  Trip to the cinema  Rent that DVD I want to see  My own reward idea is……………………………………………. | |  | |  |  |
| I will ………………………………………………  ……………………………………………………….  ……………………………………………………….  Set your own goals here | (Please tick)  Monday □  Tuesday □  Wednesday □  Thursday □  Friday □  Saturday □  Sunday □ |  | (Please tick)  A big bubble bath  My favourite magazine  Trip to the cinema  Rent that DVD I want to see  My own reward idea is……………………………………………. | |  | |  |  |
| I will ………………………………………………  ……………………………………………………….  ……………………………………………………….  Set your own goals here | (Please tick)  Monday □  Tuesday □  Wednesday □  Thursday □  Friday □  Saturday □  Sunday □ |  | (Please tick)  A big bubble bath  My favourite magazine  Trip to the cinema  Rent that DVD I want to see  My own reward idea is……………………………………………. | |  | |  |  |

SECTION 10: HELP AND SUPPORT

**Information and advice on eating well and keeping active**

**Eat well, be well website**

Detailed advice on everything to do with healthy eating, questions answered by experts, and a special section on healthy eating when you’re pregnant.

<http://www.eatwell.gov.uk/agesandstages/pregnancy/>

**NHS Choices website**

Advice on getting active, including sections on low impact exercise and how to do a housework workout.

<http://www.nhs.uk/LiveWell/Fitness/Pages/Fitnesshome.aspx>

**Change 4 Life website**

Quizzes, recipes, and a free personalised pack of tools, tips and activities.

<http://www.nhs.uk/change4life/>

**Weight Wise website**

A website run by a charity (the British Dietetic Association), including food and activity diaries and a progress chart that you can download, tips on keeping going and what to do when things go wrong.

<http://www.bdaweightwise.com/index.html>

**All about pregnancy**

**Pregnancy guides from Tommy’s**

*Your guide to a healthy pregnancy*

*The young woman’s guide to a healthy pregnancy*

These guides and additional pregnancy health information materials are available free from [www.tommys.org/shop](http://www.tommys.org/shop)

**NHS Choices website**

Everything you need to know about your health in pregnancy, your choices and your antenatal care.

<http://www.nhs.uk/LiveWell/Pregnancy/Pages/Pregnancyhub.aspx>

**All about breastfeeding**

**NHS breastfeeding website**

<http://www.breastfeeding.nhs.uk/>

**Eat well, be well website**

What to eat while you are breastfeeding

<http://www.eatwell.gov.uk/agesandstages/baby/breastfeed/>

National Breastfeeding helpline

0300 100 0212

National Childbirth Trust Breastfeeding helpline

0870 444 8708

**When a pregnancy goes wrong**

**The Miscarriage Association**

Information and support for women who have had a miscarriage.

01924 200799 (helpline) [www.miscarriageassociation.org.uk](http://www.miscarriageassociation.org.uk/)

**Stillbirth and Neonatal Death Society (SANDS)**

Support for anyone affected by the death of a baby through a helpline and local support groups.

020 7436 5881 (helpline) [www.uk-sands.org](http://www.uk-sands.org/)

**Tommy’s PregnancyLine**

Staffed by midwives to provide expert advice on how to have a healthy pregnancy.

0800 0147 800 / [info@tommys.org](mailto:info@tommys.org)

1. **References:**

   ^?^ Denison F, Norrie G, Graham B et al. *Increased maternal BMI is associated with an increased risk of minor complications during pregnancy with consequent cost implications* British Journal of Obstetrics and Gynaecology, 2009 116(11):1467-72.
2. World Health Organisation. *Obesity: preventing and managing the global*

   *epidemic.* Report on a WHO Consultation on Obesity. World Health Organisation:

   Geneva, Switzerland [WHO/NUT/NCD/98.1. Technical Report Series Number 894]

   June 3–5, 1997
3. Denison F, Norrie G, Graham B et al. *Increased maternal BMI is associated with an increased risk of minor complications during pregnancy with consequent cost implications.* British Journal of Obstetrics and Gynaecology, 2009 116(11):1467-72.
4. Ibid
5. Villamore E and Cnattingius S. *Interpregnancy weight change and risk of adverse pregnancy outcomes: a population-based study* The Lancet, 2006, 1164. 368(9542):1164-70.
6. Heslelhurst N, Lang R, Rankin J et al. *Obesity in pregnancy: a study f the impact of maternal obesity on NHS maternity services*, British Journal of Obstetrics and Gynaecology, 2007 Mar;114 (3):334-42.
7. Catalano PM. *Obesity and pregnancy– the propagation of a viscous cycle?* Journal Clinical Endocrinology, 2003 88(8):3505-6
8. Oken E and Gillman M. *Fetal origins of obesity* Obesity Research, 2003 11(4):496-506.
9. Cosson E. *Diagnostic criteria for gestational diabetes mellitus* Journal of Gynecological Obstetric Biological Reproduction, 2010, Paris, Dec; 39(8 Suppl 2):S239-50.
10. Curhan G, Chertow G, Willet W et al. *Birth weight and adult hypertension and obesity in women* Circulation, 1996 94: 1310
11. Foresen T, Eriksson J. Tuomilehto J et al. *Mother’s weight in pregnancy and coronary heart disease in a cohort of Finnish men: follow up study* British Medical Journal, 1997, 315(7112):837-40.
12. Oken E and Gillman M. *Fetal origins of obesity* Obesity Research, 2003, 11(4):496-506.
13. Duley L. *Pre-eclampsia, eclampsia, and hypertension* Clinical Evidence (Online) 2011, Feb 14;2011. pii: 1402.
14. NICE, *Dietary interventions and physical activity interventions for weight management before, during and after pregnancy,* Public health guidance 27, 2010
15. Department of Health, *Dietary reference values for food, energy and nutrients for the United Kingdom,* 1991, London HMSO
16. Galgani J, Ravussin E. *Energy metabolism, fuel selection and body weight regulation*, International Journal of Obestetrics, 2008, Dec;32 Suppl 7:S109-19.
17. Barger MK. *Maternal nutrition and perinatal outcomes* Journal of Midwifery and Women’s Health, 2010, Nov-Dec;55(6):502-11.
18. Scholl TO, Chen X, Khoo CS et al. *The dietary glycaemic index during pregnancy: influence on infant birth weight, fetal growth, and biomarkers of carbohydrate metabolism* American Journal of Epidemiology, 2004, Mar 1;159(5):467-74.
19. Elfhag K, Rössner S. *Who succeeds in maintaining weight loss? A conceptual review of factors associated with weight loss maintenance and weight regain,* Obesity Reviews, 2005, Feb;6 (1):67-85
20. NHS Choices, www.eatwell.gov.uk/agesandstages/pregnancy/
21. Czeizel AE. *Periconceptional folic acid and multivitamin supplementation for the prevention of neural tube defects and other congenital abnormalities,* Birth Defects Research, Part A: Clinical and Molecular Teratology, 2009, Apr;85(4):260-8.
22. RCOG, ‘Why Your Weight Matters in Pregnancy and Birth’, November 2011, http://www.rcog.org.uk/files/rcog-corp/Why%20Your%20Weight%20Matters%20During%20Pregnancy.pdf
23. Thacher TD, Clarke BL. *Vitamin D insufficiency,* Mayo Clinic Proceedings, 2011 Jan;86(1):50-60.
24. Department of Health *Dietary reference values for food, energy and nutrients for the United Kingdom*, 1991, London HMSO
25. Henderson C and Macdonald S, *Mayes’ Midwifery A textbook for midwives* 1997 Elsevier
26. Jewell D and Young G., Interventions for nausea and vomiting in early pregnancy. *Cochrane Database of Systematic Reviews* 2003 Issue 4. Art. No.: CD000145. DOI: 10.1002/14651858.CD000145
27. [www.nhs.uk/change4Life](http://www.nhs.uk/change4Life), *Food exercise mythbuster*, accessed 2 Aug 2011
28. Institute of Medicine. *Weight Gain During Pregnancy:*

    *Re-examining the Guidelines*, Institute of Medicine (US) guidelines, 2009
29. Ibid
30. Royal College of Obstetricians and Gynaecologists, *Exercise in Pregnancy*, RCOG Statement 4, 2006
31. Gross H and Bee P *Perceptions of effective advice in pregnancy – The case of activity* Clinical Effectiveness in Nursing, 2004
32. Royal College of Obstetricians and Gynaecologists *Exercise in Pregnancy*, RCOG Statement 4, 2006
33. Duncombe D, Wertheim E, Skouteris H et al. *Factors related to exercise over the course of pregnancy including women’s beliefs about the safety of exercise during pregnancy*Midwifery, 2009, (25)
34. Royal College of Obstetricians and Gynaecologists. *Exercise in Pregnancy*, RCOG Statement 4, 2006
35. Ibid
36. Ibid
37. Ibid
38. Gaston A, Cramp A. *Exercise during pregnancy: A review of patterns and determinants*, Journal of Science and Medicine Sport, 2011 Jul;14(4):299-305.
39. Ibid
40. Miyachi M, Yamamoto K, Ohkawara K, Tanaka S *METs in adults while playing active video games: a metabolic chamber study* Medicine and Science in Sports and Exercise, 2010, Jun;42(6).
41. Taylor S E, Pham L B, Rivkin I D et al. *Harnessing the imagination. Mental simulation, self-regulation and coping,* American Psychologist, 1998, Apr 53(4) 429-39.
42. Westenhoesfer J, Von Falck B, Stellfeldt A et al. *Behavioural correlates of successful weight reduction over 3 years. Results from the Lean Habits Study*, International Journal of Obesity Related Metabolic Disorders, 2004, Feb; 28 (2) 334-5.
43. Blair AJ, Lewis VJ, Booth DA. *Does emotional eating interfere with success in attempts at weight control?* Appetite, 1990, Oct; 15(2):151-7.
44. Kumanyika SK, Wadden TA, Shults J*,Trial of Family and Friend Support for Weight Loss in African American Adults* Archives of Internal Medicine 2009[;169[19]:1795-1804](http://archinte.ama-assn.org/cgi/content/short/169/19/1795)
45. Department of Health. Infant Feeding Recommendation, 2003
46. Dewey KG, Heinig MJ, Nommsen LA. *Maternal weight-loss patterns during prolonged lactation*, American Journal of Clinical Nutrition, 1993, Aug;58(2):162-6.
47. Davis MK. *Breastfeeding and chronic disease in childhood and adolescence* Pediatric Clinics of North America, 2001, Feb;48(1):125-41,
