# Supplementary material for: Using intervention mapping to develop a culturally appropriate intervention to prevent childhood obesity: the HAPPY (Healthy and Active Parenting Programme for Early Years) study
Source: Int J Behav Nutr Phys Act. 2013 Dec 28;10:142. doi: 10.1186/1479-5868-10-142 (PMC3895739; doi:10.1186/1479-5868-10-142)
Supplement: Additional file 4 — a. MASTER HAPPY Antenatal Manual FINAL VERSION. b. MASTER HAPPY Postnatal Manual FINAL VERSION. c. HAPPY manual resources. [file 1479-5868-10-142-S4.zip › 1523029418990916_add4c2.zip!/How am I doing diary handout.doc]

**‘How am I Doing?’ Diary**

This diary can be used to help you think about your food and activity through the day and any changes you’d like to make. You don’t need to share it with anyone, but you can show it to the HAPPY group leader once completed. There is no need to keep a diary all the time but it can help you to get started or back on track.

| **Day** | **What did I eat or drink?**  **How much? Where was I?** | **What physical activity have I done today?** | **My thoughts about today: what went well? Any problems? Ideas on solving them?** |
| --- | --- | --- | --- |
| **Monday** |  |  |  |
| **Tuesday** |  |  |  |
| **Wednesday** |  |  |  |
| **Thursday** |  |  |  |
| **Friday** |  |  |  |
| **Saturday** |  |  |  |
| **Sunday** |  |  |  |
