# Supplementary material for: Using intervention mapping to develop a culturally appropriate intervention to prevent childhood obesity: the HAPPY (Healthy and Active Parenting Programme for Early Years) study
Source: Int J Behav Nutr Phys Act. 2013 Dec 28;10:142. doi: 10.1186/1479-5868-10-142 (PMC3895739; doi:10.1186/1479-5868-10-142)
Supplement: Additional file 4 — a. MASTER HAPPY Antenatal Manual FINAL VERSION. b. MASTER HAPPY Postnatal Manual FINAL VERSION. c. HAPPY manual resources. [file 1479-5868-10-142-S4.zip › 1523029418990916_add4c2.zip!/Pregnancy and health myth buster handout.doc]

**Pregnancy and Health – Myth Buster**

1. **You need to eat for two in pregnancy: True or False?**

**False** – amazingly up until the last 3 months of pregnancy your baby grows well without any extra calories. During the last 3 months an additional 200kcals is needed. However, most women meet this through natural slight changes in appetite and activity as their pregnancy progresses from 6 to 9 months.

**Some examples of foods containing 200kcals**

- ½ can of creamed tinned soup
- 2 slices of bread
- 1 banana and 1 apple
- 1 glass of semi-skimmed milk and 1 pot of yoghurt

2. **You need to eat more often when you are pregnant so extra snacks are important: True or False?**

**False** - you only need a small amount of extra calories in the last 3 months. (see above). If you do want to snack then try and choose healthier options (ones that are not fattening and also provide you with some other nutrients). Examples are fruit and low fat yogurt. You might want to try a drink first –often we confuse being thirsty for being hungry.

There are more ideas on the **Smart Snacks Handout**.

3. **You can’t do any exercise when you are pregnant: True or False?False** - you can still exercise in pregnancy and this will help keep you well and active. Moderate – intensity physical activity is recommended like brisk walking and swimming.
4. **Pregnant women who are very overweight, should try to lose weight before the baby is born: True or False?**

**False** - dieting (i.e. restrictive diet aimed at weight loss) is not recommended during pregnancy as it might harm the health of the unborn child.

Pregnancy weight gain is due to:

- Increase in fat and protein stores in the body to be ready to produce milk for the baby
- Increased volume of blood
- Weight of the baby, amniotic fluid and placenta

You can manage your weight by eating well and keeping active throughout your pregnancy and this may help you to achieve a healthier pregnancy, safer birth and healthier baby.

5. **Being more active and eating healthily now will help me after the birth: True or False?**

**True** - by eating sensibly during pregnancy you may not have so many extra pounds to lose afterwards and you will already have established some better habits which will be easier to continue. You may also feel better in yourself too.
