# Supplementary material for: Using intervention mapping to develop a culturally appropriate intervention to prevent childhood obesity: the HAPPY (Healthy and Active Parenting Programme for Early Years) study
Source: Int J Behav Nutr Phys Act. 2013 Dec 28;10:142. doi: 10.1186/1479-5868-10-142 (PMC3895739; doi:10.1186/1479-5868-10-142)
Supplement: Additional file 4 — a. MASTER HAPPY Antenatal Manual FINAL VERSION. b. MASTER HAPPY Postnatal Manual FINAL VERSION. c. HAPPY manual resources. [file 1479-5868-10-142-S4.zip › 1523029418990916_add4c2.zip!/Being active during pregnancy handout.zip!/Being active during pregnancy handout.pdf]

Do's and Don'ts

Don't exercise…

To exhaustion
If it is very hot
When you feel unwell
If you have deep pelvic pain
If you have any vaginal bleeding
If you doctor/midwife has told you to stop exercising

Do…

Wear shoes that support your ankles
Wear a good supportive bra
Wear loose comfortable clothes
Drink plenty of water

I will not have any energy left to do other things

You may have times when you feel very tired and you may find that doing light-to-moderate exercise actually energises you

How can I stay on track?

Talk to the people who are close to you at home. It might be your partner, a member of the family or a close friend. You can give them this information to read though and ask them to support you and cheer you on to do physical activity. You can even ask them to join you in being more active

Fill in the table below with the names of the people you would like to support and how you would like them to support you.

Name	I would like them to support me by…
e.g Mohammad my husband	*Taking a 15 min walk with me twice a week*

HAPPY
Healthy and Active Parenting Programme for early Years
eat well, drink well, play well

Being Active During Pregnancy

Why is physical activity important?

Physical activity includes both "formal" exercise, like going to an exercise class and "informal", lifestyle exercise like walking to the shops.

Physical activity during pregnancy is safe in most cases and can improve the health of both the baby and mother.

Doing regular physical activity can help you have a more comfortable and enjoyable pregnancy. It will prepare your body for the challenges of labour and childcare once your baby is born .

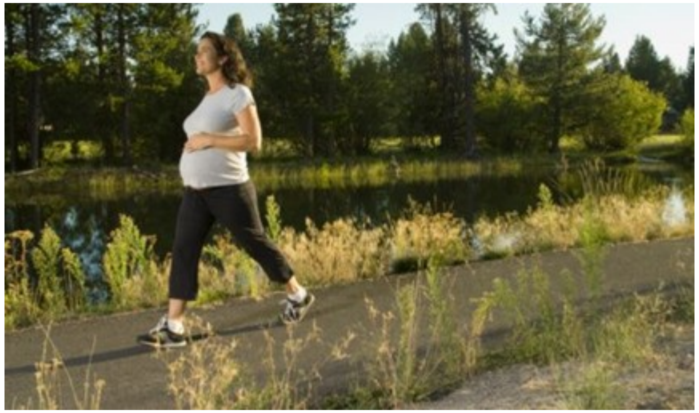

Here are some **benefits** of being physically active during pregnancy. Which are most important to you? Rank the benefits from 1 to 10 where 1 is the highest and 10 is the lowest importance.

Benefit	importance
It improves and strengthens muscles so you'll have fewer aches and pains	
You will have less chance of varicose veins and swollen legs	
It will help improve your blood sugar control and may help prevent you getting gestational diabetes	
It will increase your stamina which will help you and your baby though labour	
Your baby's risk of pre-term birth and low birth weight is lower.	
It will help you to recover more quickly from your baby's birth	
You may have less complications during delivery	
You will be able to sleep better and you will have more energy	
It will help you to be less anxious and depressed	
You will be able to lose those extra pounds more easily once you have your baby	

I might have a miscarriage or harm the baby

The risk of miscarriage is very low after 14 weeks. Doing light-to-moderate physical activity does not increase risk of miscarriage or harm the baby.

What types of physical activity are safe for me to do?

Most activities are safe to do during pregnancy.

- Walking is one of the easiest and best ways to be active and you can fit it into your daily routines.
- Low impact aerobics and dance are fun ways to be active and will keep your muscles, heart , and lungs strong.
- Some women enjoy swimming when they are pregnant as the water supports their bump and eases strain on the back.

What activities should I avoid?

- It's best to avoid activities that have an increased risk of falling like gymnastics, skating and physical contact sports (e.g. vigorous ball games).
- Avoid exercising on your back after 16 weeks into your pregnancy. The weight of your baby may press on major blood vessels and reduce oxygen and blood supply to the baby.
- Scuba diving should also be avoided during pregnancy.

How much physical activity should I do?

Pregnancy is a great time to start being more active. Remember activity includes formal exercise (e.g. going to the gym) and informal exercise (walking to the shops).

If you are new to physical activity start by doing 15 minutes of continuous weight bearing activities that make your heart and lungs work harder (e.g. walking or dancing) on 3 days of the week.

When you are exercising at the right level for health benefits, you should be breathing a little harder than usual and still be able to hold a conversation comfortably.

You should aim to build up to 30 minutes of activity everyday. **If you like, you can do your 30 minutes in 10 minute blocks.**
