# Supplementary material for: Using intervention mapping to develop a culturally appropriate intervention to prevent childhood obesity: the HAPPY (Healthy and Active Parenting Programme for Early Years) study
Source: Int J Behav Nutr Phys Act. 2013 Dec 28;10:142. doi: 10.1186/1479-5868-10-142 (PMC3895739; doi:10.1186/1479-5868-10-142)
Supplement: Additional file 4 — a. MASTER HAPPY Antenatal Manual FINAL VERSION. b. MASTER HAPPY Postnatal Manual FINAL VERSION. c. HAPPY manual resources. [file 1479-5868-10-142-S4.zip › 1523029418990916_add4c2.zip!/Smart snacks handout.docx]

**Smart Snacks**

You may find you want to eat ‘little and often’ as pregnancy progresses. Some snack foods like cakes, biscuits, crisps and confectionery are high in calories. Swapping these for healthier alternatives will provide more nutrients for you and your baby.

There are also many emotions that might make you want to eat and these include stress, anxiety, anger, sadness, depression, boredom, or loneliness. Varying life events and life stages can also trigger you to overeat and indulge in less healthy foods.

The checklist below might be helpful in identifying whether sometimes you do eat for emotional reasons:-

- Do you eat when you are not hungry?
- Do you eat when you are bored or unoccupied?
- Do you eat immediately following a fit of anger or when you are lonely or sad?
- Does eating make you feel better for a short-time but then you start feeling guilty?

If your answer is “yes” to most of the questions listed above then you do eat for emotional reasons.

If this is the case then try to identify the situations or circumstances that may result in you eating for emotional reasons and plan ahead for such times by having healthier options accessible e.g. chopped up fruit and vegetables in the fridge – ready to eat. However remember to watch your portion sizes too.

Here are some ‘Smart’ swaps for you to try:

| **Try to limit these:** | **Choose these instead:** |
| --- | --- |
| Cakes  Chocolate or cream biscuits  Fruit pies  Chocolate mouse  Chocolate  Sweets  Ice cream  Crisps and savoury snacks  Sausage roll and pastries  Takeaways and ready meals | Fruit, fruit loaf, small scone, half breakfast muffin.  Fresh or dried fruit, fruit oat biscuits, fig rolls, plain biscuits  Baked fruit with low fat yoghurt  Low sugar jelly with tinned fruit in juice.  Small handful mixed fruit and nuts  Strawberries, grapes or dried fruit  Frozen low fat yoghurt  Homemade lollies using pure fruit juice.  Rice cakes, bread sticks, plain popcorn  Crisp breads or plain crackers with low fat cream cheese, raw veg with low fat dips  Have as occasional treats.  Choose tomato based sauces rather than creamy ones.  Have baked potatoes, boiled rice or plain chapattis rather than chips, fried rice or parathas.  Add some salad or vegetables. |

For more ideas about snack swaps and recipes for healthy meals on a budget go to the Change4Life website on <http://www.nhs.uk/Change4Life/Pages/change-for-life.aspx>
