# Supplementary material for: Using intervention mapping to develop a culturally appropriate intervention to prevent childhood obesity: the HAPPY (Healthy and Active Parenting Programme for Early Years) study
Source: Int J Behav Nutr Phys Act. 2013 Dec 28;10:142. doi: 10.1186/1479-5868-10-142 (PMC3895739; doi:10.1186/1479-5868-10-142)
Supplement: Additional file 4 — a. MASTER HAPPY Antenatal Manual FINAL VERSION. b. MASTER HAPPY Postnatal Manual FINAL VERSION. c. HAPPY manual resources. [file 1479-5868-10-142-S4.zip › 1523029418990916_add4c2.zip!/TIPSFO~1.DOC]

**Tips for Coping with Common Food and Health Problems in Pregnancy**

Women experience lots of physical changes during pregnancy. Some of these changes can affect what you want to eat. The following information gives you some ideas of how to deal with the more common problems associated with food during pregnancy. There are also some website links to where you can find more support.

**Nausea and morning sickness**

Nausea and/or vomiting are commonly experienced during pregnancy, particularly in the first 3 months of pregnancy but it can continue longer. Some women are sick whilst others feel sick without actually being sick. It may affect you first thing in the morning, at other times or all day. The causes of this aren’t fully understood but it is thought that it is linked to hormonal changes during the first 3 months of pregnancy. Some women suffer with extreme vomiting at this time and will need more specialist support from their health professionals – speak to your midwife if you have any concerns.

Sickness can be difficult to cope with as it happens at the same time as you may be feeling very tired and emotional. Things that may make it worse are traveling, food smells and food preparation. Here are some ideas to try that may help:

- Frequent snacks with starch foods like toast, breakfast cereal,

sandwich, crackers or crisp bread.

- **Before getting out of bed** eat some dry bread, plain biscuits or cereal.
- When getting out of bed, do so slowly and avoid sudden movements.
- Drink small amounts of fluid, preferably in between meals to avoid

your stomach becoming bloated which can trigger vomiting.

- Avoid large meals - try having small meals more often
- Avoid meals that are high in fat or spicy foods. Try bland foods like baked potatoes, pasta and milk puddings.
- Suck sour foods, like lemon or sharp boiled sweets.
- Sip a fizzy drink slowly if feeling nauseous.
- Try food or drink containing ginger (ginger biscuits, non-alcoholic

ginger beer).

**Constipation**

Some women experience constipation early in pregnancy due to hormonal changes whilst others may suffer with it later on as the growing womb presses on internal organs and as they become less physically active as they get bigger.

These are some ideas for how to prevent constipation:

- Try to have at least 8 cups of fluid a day.
- Include some high fibre foods such as:

- wholegrain breads, breakfast cereals and flours (e.g. for chappatis)

- brown rice and pasta

- beans, dahls, peas, lentils and other pulse vegetables

- fruit and vegetables.

- Getting yourself moving helps -Try to include some physical activity every day.

**Indigestion and heartburn**

Most women suffer indigestion and heartburn during pregnancy. Indigestion is often experienced as the growing womb presses on the stomach. Symptoms can include feeling full, sick or burping.

Heartburn feels like a burning pain in the chest and is caused by acid from the stomach leaking into the oesophagus. This is because the valve between the stomach and oesophagus relaxes during pregnancy.

The following suggestions can help to manage the symptoms but if they persist speak to your midwife – she may be able to prescribe an antacid.

- Try smaller, balanced meals and healthy snacks more often rather just one or two big meals in the day.
- Avoid highly seasoned or spicy foods and fizzy drinks if these trigger

symptoms

- Some women find milk and yoghurt can help to relieve symptoms –choose low fat versions.
- Wear loose fitting clothes.
- Don’t lay down soon after eating – make sure you have meals a few hours before going to bed.
- Sleeping with the bed head raised or propped up on pillows may also be of benefit.

**Food aversions and cravings**

Some women experience food aversions and cravings during pregnancy. Common

aversions include tea, coffee, alcohol, fried foods and meat.

If you have food aversion or cravings it is important to ensure that you are still having a varied, balanced diet and including foods from the main 4 food groups.

If you crave substances which are not usually food based (e.g. clay, chalk, ice, paper) you should speak to your midwife or GP to ensure that any substances are non-toxic.

Information on these and other common problems experienced in pregnancy can be found on the Pregnancy Planner on the NHS Choices website, available from <http://www.nhs.uk/Planners/pregnancycareplanner/>

And in the Pregnancy Book your midwife may have given you or at this link

<http://www.dh.gov.uk/prod_consum_dh/groups/dh_digitalassets/@dh/@en/@ps/@sta/@perf/documents/digitalasset/dh_107681.pdf>
