# Supplementary material for: Using intervention mapping to develop a culturally appropriate intervention to prevent childhood obesity: the HAPPY (Healthy and Active Parenting Programme for Early Years) study
Source: Int J Behav Nutr Phys Act. 2013 Dec 28;10:142. doi: 10.1186/1479-5868-10-142 (PMC3895739; doi:10.1186/1479-5868-10-142)
Supplement: Additional file 4 — a. MASTER HAPPY Antenatal Manual FINAL VERSION. b. MASTER HAPPY Postnatal Manual FINAL VERSION. c. HAPPY manual resources. [file 1479-5868-10-142-S4.zip › 1523029418990916_add4c3.zip!/Physical activity ball for pregnancy handout .pdf]

Physical Activity Ball for Pregnancy

Inactivity

Limit the amount of time you spend sitting for extended periods.

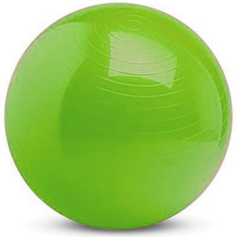

Strength and flexibility

Do these exercises 2 times a week

Lifestyle activities

Do these activities everyday

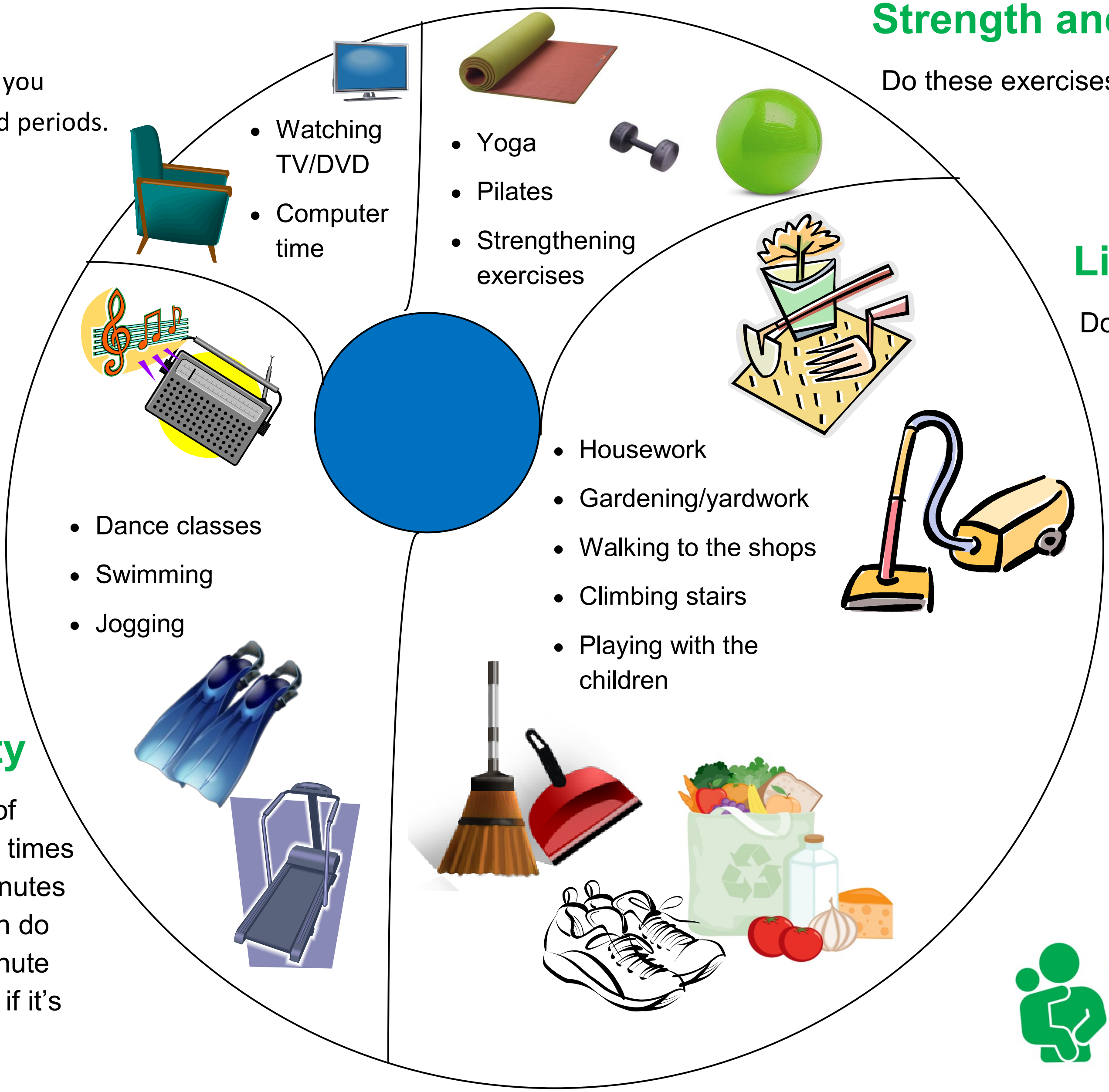

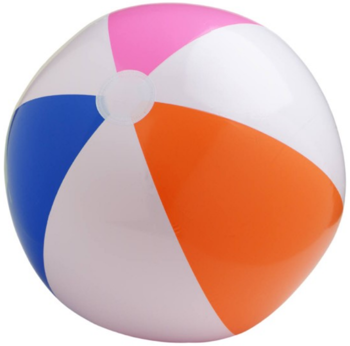

Aerobic activity

Start with 15 minutes of moderate aerobic activity 3 times a week. Work up to 30 minutes 5 times a week. You can do your 30 minutes in 10 minute blocks throughout the day if it's easier for you.

HAPPY
Healthy and Active Parenting Programme for early Years
eat well, drink well, play well
