# Supplementary material for: Using intervention mapping to develop a culturally appropriate intervention to prevent childhood obesity: the HAPPY (Healthy and Active Parenting Programme for Early Years) study
Source: Int J Behav Nutr Phys Act. 2013 Dec 28;10:142. doi: 10.1186/1479-5868-10-142 (PMC3895739; doi:10.1186/1479-5868-10-142)
Supplement: Additional file 4 — a. MASTER HAPPY Antenatal Manual FINAL VERSION. b. MASTER HAPPY Postnatal Manual FINAL VERSION. c. HAPPY manual resources. [file 1479-5868-10-142-S4.zip › 1523029418990916_add4c3.zip!/ADVANT~1.DOC]

 ADVANTAGES AND DISADVANTAGES OF BREAST AND BOTTLE-FEEDING

| **Breastfeeding** | | | |
| --- | --- | --- | --- |
| **Baby** | | **Mother** | |
| **Advantages** | **Disadvantages** | **Advantages** | **Disadvantages** |
| **Complete food**:  once established breast milk provides complete nutrition until around 6 months of age | Difficult to know how much breast milk has been consumed | **Helps with post-pregnancy weight-loss**:  breastfeeding  uses up c.500 extra calories a day so mums who breastfeed often find it easier to lose their pregnancy weight | Demands on the mother’s time – day and night |
| **Immediately available** at the correct temperature without additional preparation |  | **Reduces fertility** temporarily | Physical discomfort from engorged breasts or sore nipples: *only when establishing breastfeeding so short-lived* |
| **Allows frequent contact** and assists in bonding |  | **Reduced risk of breast and ovarian cancer** later in life | Could be embarrassing e.g. due to attitude of partner/others |
| **Protection against infection**   - Gastro-intestinal infections - ear, chest & tummy bugs |  | **Reduced risk of thin bones** in later life (osteoporosis) | Lack of facilities to feed away from home |

| **Breastfeeding** | | | |
| --- | --- | --- | --- |
| **Baby** | | **Mother** | |
| **Advantages** | **Disadvantages** | **Advantages** | **Disadvantages** |
| Reduced risk of developing eczema |  | **Breastfeeding is cheaper** than bottle-feeding even when costs of mother’s diet are taken into account. | May create jealousy amongst partner, relatives or other children because of the exclusive role of the mother |
| Prevents constipation |  |  | Incompatible when mother returns to work |
| Reduced risk of baby being overweight or being diabetic as a child |  |  |  |
| Reduced risk developing heart disease, diabetes or high cholesterol  when they are older |  |  |  |
| Reduced risk of baby being fussy about new foods:  baby will receive all flavours from mother’s diet via breast milk & be more accepting of new foods |  |  |  |
| Reduced risk of sudden infant death (cot death) |  |  |  |

| **Bottle-feeding** | | | |
| --- | --- | --- | --- |
| **Baby** | | **Mother** | |
| **Advantages** | **Disadvantages** | **Advantages** | **Disadvantages** |
| Others can also feed  Allows others to have contact and bonding with baby | Baby had 1 flavour (formula) so may be less accepting of new flavours/foods during weaning | Not dependent on mother alone – others can share the task of feeding | Time-consuming to prepare feeds |
|  |  | Maybe less tiring for the mother | Need equipment – sterilising; bottles, teats etc… |
|  |  |  | Cost of formula milk and equipment |
