# Supplementary material for: Using intervention mapping to develop a culturally appropriate intervention to prevent childhood obesity: the HAPPY (Healthy and Active Parenting Programme for Early Years) study
Source: Int J Behav Nutr Phys Act. 2013 Dec 28;10:142. doi: 10.1186/1479-5868-10-142 (PMC3895739; doi:10.1186/1479-5868-10-142)
Supplement: Additional file 4 — a. MASTER HAPPY Antenatal Manual FINAL VERSION. b. MASTER HAPPY Postnatal Manual FINAL VERSION. c. HAPPY manual resources. [file 1479-5868-10-142-S4.zip › 1523029418990916_add4c3.zip!/Healthy eating choices handout.doc]

**Healthy Eating Choices**

During pregnancy it is important to make sure you get all the nutrients needed for your growing baby. It is also very important to look after yourself and manage your weight gain during pregnancy. Being overweight means you and your baby are at risk of complications. Making some small changes can help you to adopt a healthier lifestyle that will benefit your whole family – and mean you have less weight to lose after you’ve had your baby!

Many of us eat too many fatty and sugary foods. These are high in calories and often don’t have many other nutrients that are needed to keep you and your baby healthy. The following guide provides ideas of food, drink and cooking swaps.

**Try and identify 2 or 3 things that you could change and plan to make the swap**

**When you’ve tried those, look for 2 or 3 more.**

|  | **Try to limit:** | **Choose these instead:** |
| --- | --- | --- |
| **Drinks** | Fizzy drinks with added sugar  Squashes and cordials with added sugar  Milkshakes  Latte, cappuccino, hot chocolate drinks  Adding sugar to drinks | Diet fizzy drinks  Sugar free squash, cordial or flavoured water.    Home made fruit smoothie made with semi or skimmed milk  De-caffeinated coffee made with skimmed milk (eg ‘skinny latte’) or fruit/herbal tea.  Cut down by a small amount each week or try a sweetner |
| **Starchy foods** | Adding butter or margarine to potatoes, bread and chapattis  Rich creamy/cheesy sauces with pasta and rice  Fried rice  Chips or potatoes roasted in fat  Sugar coated breakfast cereals | A low fat spread or only use a scraping of ordinary fat spread.  Use low fat fillings in baked potatoes like baked beans or vegetable curry.  A tomato and vegetable sauces  Boiled rice  Oven chips or homemade potato wedges with small amount of oil.  Wholegrain and high fibre cereals. Use a sweetner or add some dried fruit to sweeten them. |
| **Fruit and vegetables** | Tinned fruit in syrup  Adding ice cream or cream to fruit  Adding butter/margarine to vegetables  Oil added to vegetables for cooking  Mayonnaise, salad creams and oil based salad dressings | Tinned fruit in juice  Low fat yoghurts or fromage frais  Experiment with different herbs and spices  Measure oil with a spoon (1 tablespoon is enough for 4 people) or try a spray oil.  Try reduced fat versions and use small amounts. |
| **Dairy foods** | Full fat milk  Hard cheeses  Cream cheeses  Full fat or rich and creamy yoghurts | Semi-skimmed, 1% or skimmed milk instead.  Use Edam cheese or half-fat cheese. Try grated cheese – you will use less.  Cottage cheese or reduced fat cream cheese.  Low fat or diet yoghurts and fromage frais. |
| **Protein foods** | Full fat mince  Streaky bacon  Sausages, burgers, kebabs  Fried meats, fish, vegetarian alternatives  Processed meats and pies/pastries | Lean mince, strain off any fat at beginning of cooking.  Back bacon with fat trimmed off.  Try to buy good quality, high meat content version. Make your own at home and make sure you grill them.  Alternative cooking methods – grill, bake, steam, microwave.  Lean cuts of meat, chicken and fish. Cut off any fat. Beans, peas and lentils can be used in place of meat in casseroles and curries and are cheaper. |
| **Fats** | Butter, lard, dripping, ghee | Vegetable oils such as rapeseed (used for most own brand supermarket oils), olive or sunflower oil. |
