# Supplementary material for: Using intervention mapping to develop a culturally appropriate intervention to prevent childhood obesity: the HAPPY (Healthy and Active Parenting Programme for Early Years) study
Source: Int J Behav Nutr Phys Act. 2013 Dec 28;10:142. doi: 10.1186/1479-5868-10-142 (PMC3895739; doi:10.1186/1479-5868-10-142)
Supplement: Additional file 4 — a. MASTER HAPPY Antenatal Manual FINAL VERSION. b. MASTER HAPPY Postnatal Manual FINAL VERSION. c. HAPPY manual resources. [file 1479-5868-10-142-S4.zip › 1523029418990916_add4c3.zip!/Off to the best start handout Start 4 Life.pdf]

NHS

off to the best start

Important information about feeding your baby

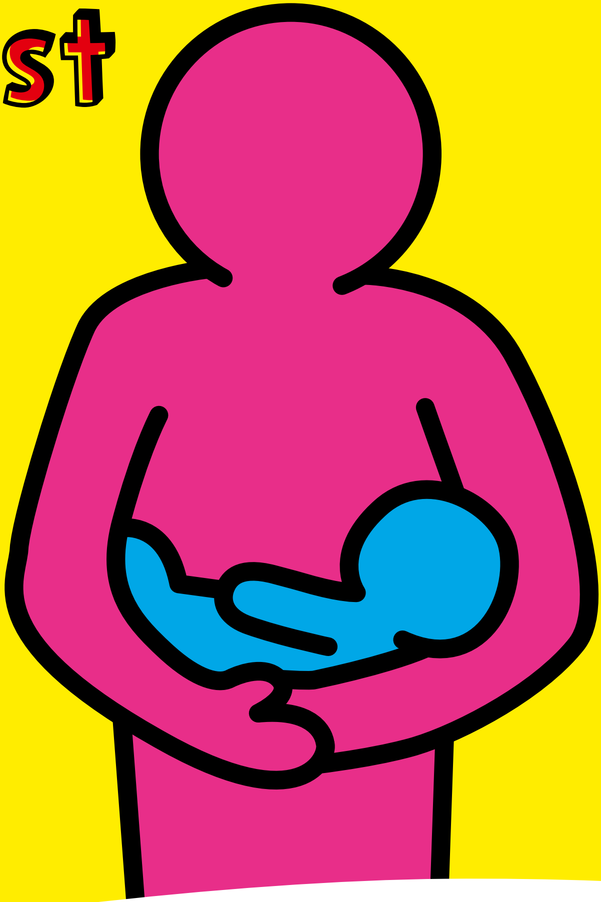

UK BABY FRIENDLY INITIATIVE

unicef

start 4 life

A good start for a healthier life

in this leaflet

What is Start4life?	3
The best start for your baby	4
What does breastfeeding help protect against?	5
After your baby is born	7
How to breastfeed	8
Signs that your baby is feeding well	11
Top tips	13
Expressing milk	15
How do I know my baby is getting enough milk?	18
How can dads and partners support breastfeeding?	20
What is the information service for parents?	23

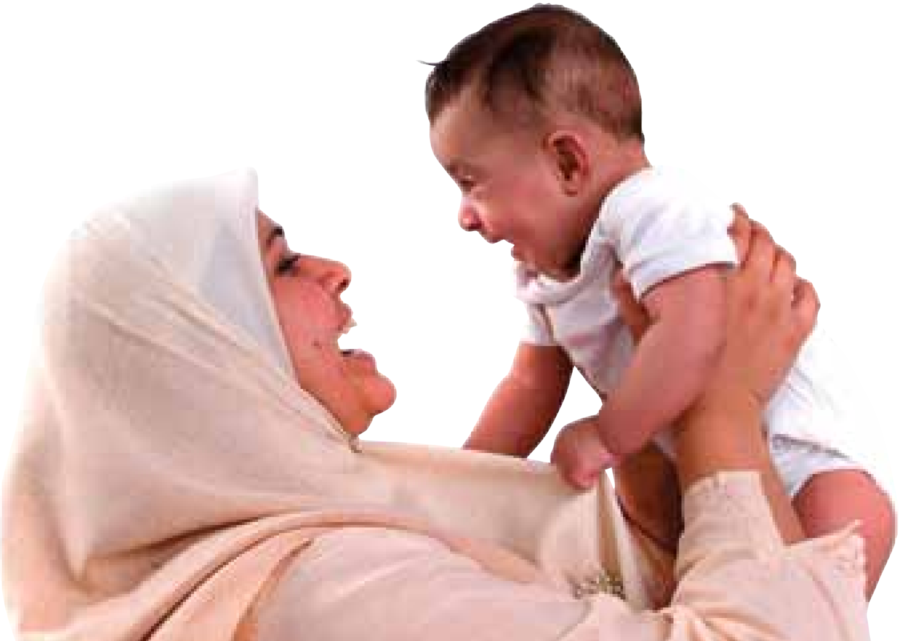

what is start4life?

Start4Life is here to help you give your baby a healthier start in life. There are 6 Start4Life building blocks, based on the latest infant health research:

1. mum's milk – why mums are the baby milk experts
2. every day counts – how each day of mum's milk makes a difference to your baby's health
3. no rush to mush – 3 signs that your baby is ready to start on solid foods
4. taste for life – how giving your baby a variety of food now may stop them turning into a fussy eater later
5. sweet as they are – how to avoid giving your baby a sweet tooth
6. baby moves – why it's important for all little ones to be lively and active

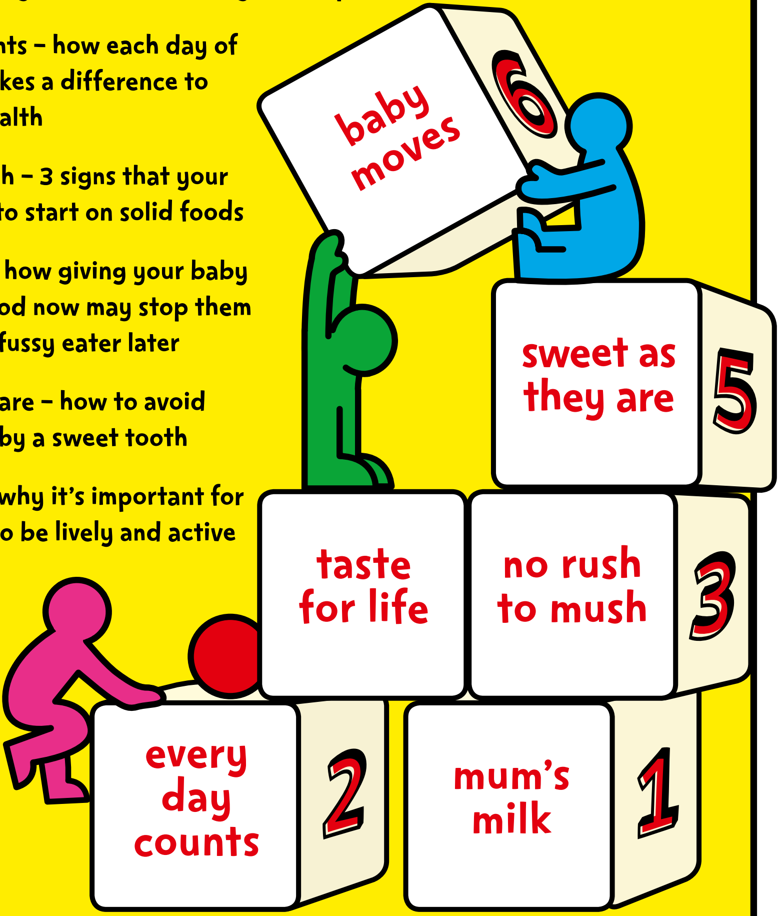

mum's milk

the best start for your baby

What happens in your baby's first years has a big effect on how healthy he or she will be in the future.

Mum's milk gives your baby all the nutrients he or she needs for around the first 6 months of life (and it's important beyond 6 months too). It helps to protect your baby from infection and other diseases, and as a mum, it also reduces your chances of getting some illnesses later in life.

Infant formula is made from cows' milk and other ingredients. It doesn't contain the ingredients that help protect your baby from infection and disease. Only your body can make those.

Breastfeeding also helps you and your baby to get closer – physically and emotionally. So while you are feeding your baby, the bond between you grows stronger.

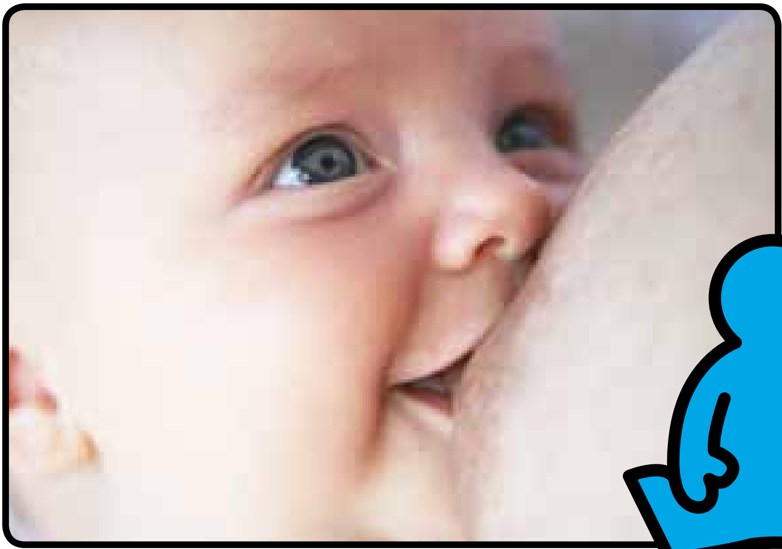

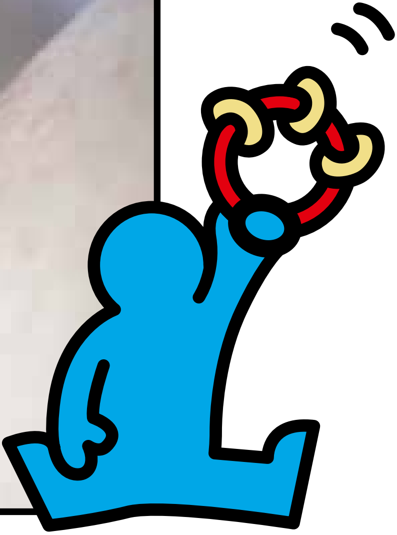

what does breastfeeding help protect against?

Your milk is perfect and uniquely made for your growing baby's needs. Giving your milk to your baby makes a big difference to both your baby's health and yours. And every day counts: the longer you feed your baby mum's milk, the more they benefit.

Babies who are NOT breastfed have an increased chance of:

- Diarrhoea and vomiting and having to go to hospital as a result
- Chest infections and having to go to hospital as a result
- Ear infections
- Being fussy about new foods
- Being constipated
- Being obese, which means they are more likely to develop type 2 diabetes and other illnesses later in life
- Developing eczema

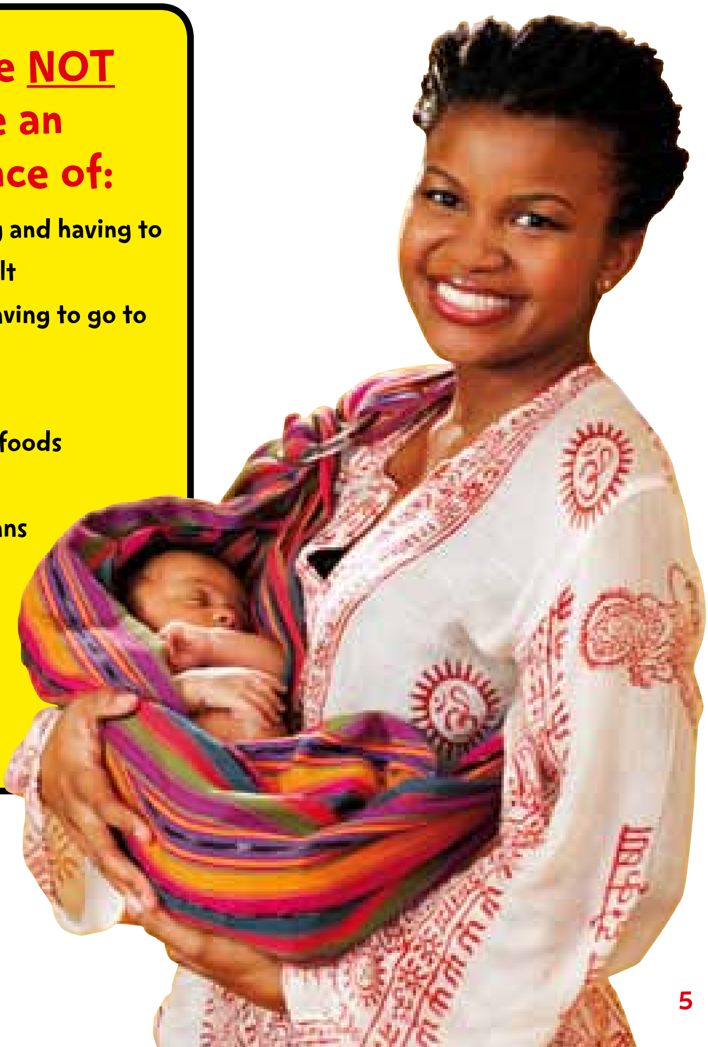

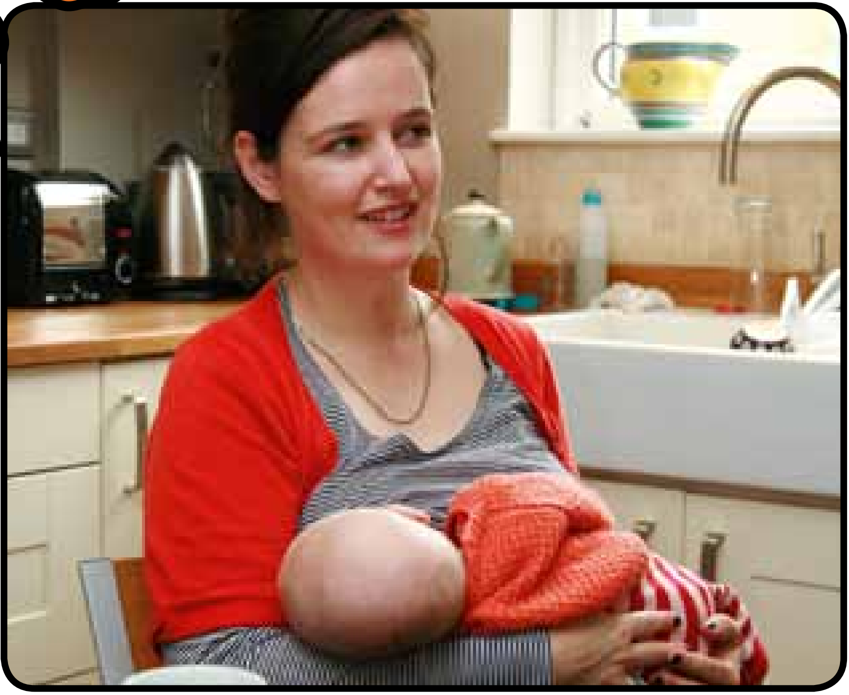

Breastfeeding is good news for mums as:

- It lowers the risk of mum getting breast and ovarian cancer
- It naturally uses up about 500 extra calories a day so mums who breastfeed may find it easier to lose their pregnancy weight
- It saves money – breast milk is FREE and formula feeding costs will add up

after your baby is born

Holding your baby against your skin straight after birth will calm your baby. It will also steady his breathing and help to keep him warm.

This is a great time to start your first breastfeed because your baby will be alert and will want to feed in the first hour after birth. Your midwife can help you with this.

Your baby will be happier if you keep him near you and feed him whenever he is hungry. This will remind your body to produce plenty of milk.

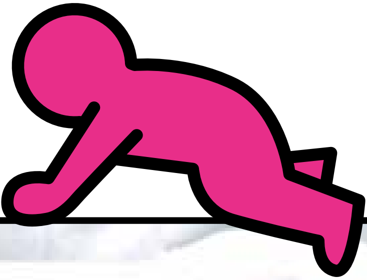

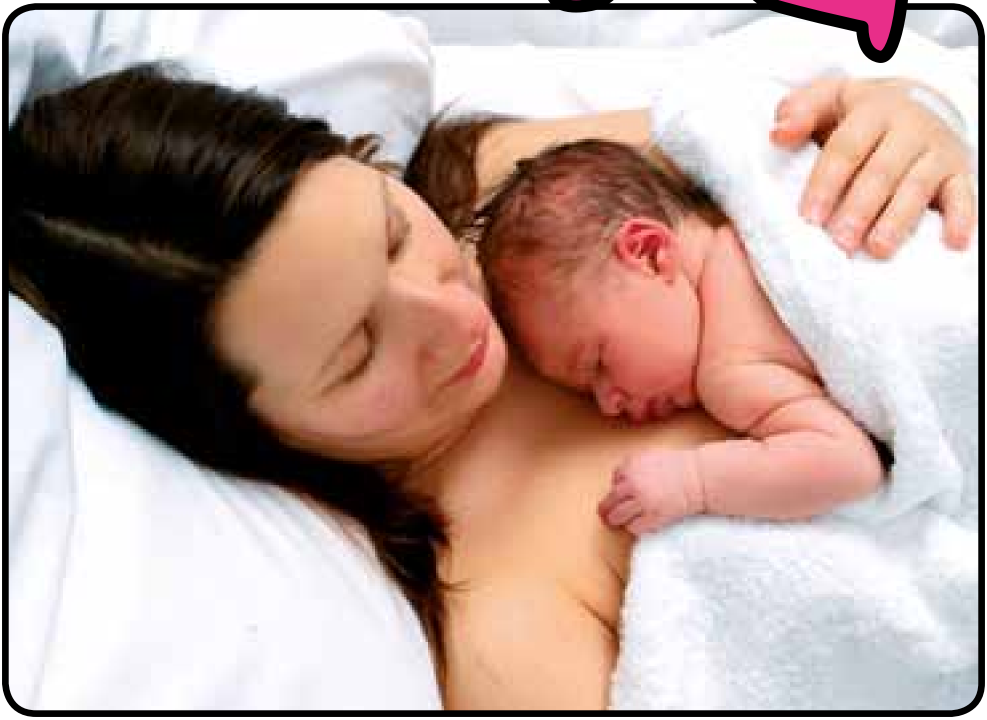

how to breastfeed

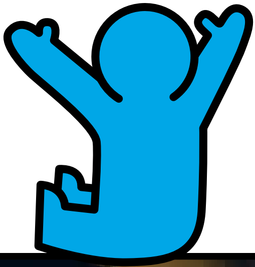

What position should you use?

There are lots of different positions for breastfeeding. You just need to check the following:

Are your baby's head and body in a straight line?

If not, your baby might not be able to swallow easily.

Are you holding your baby close to you?

Support his neck, shoulders and back. He should be able to tilt his head back easily, and he shouldn't have to reach out to feed.

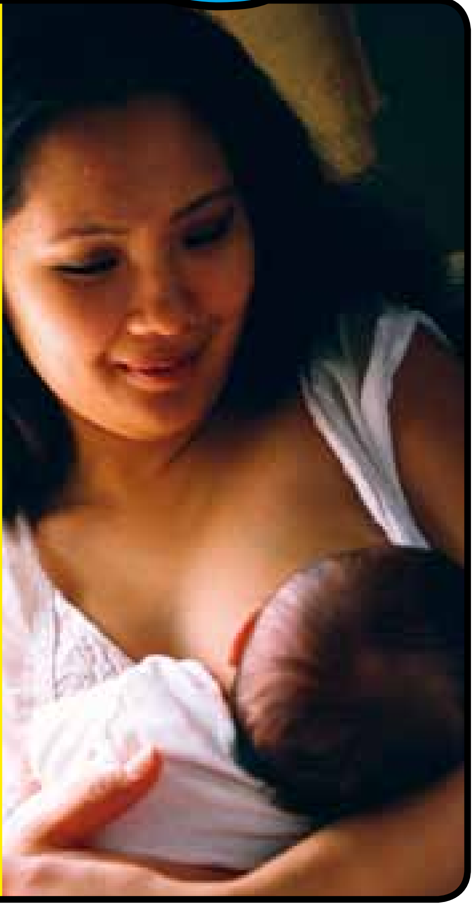

Are you comfortable?

It's worth getting comfortable before a feed, although it's ok to change your position slightly once your baby is attached to your breast.

Is your baby's nose opposite your nipple?

Your baby needs to get a big mouthful of breast from underneath the nipple. Placing your baby with his nose level with your nipple will allow him to reach up and attach to your breast well.

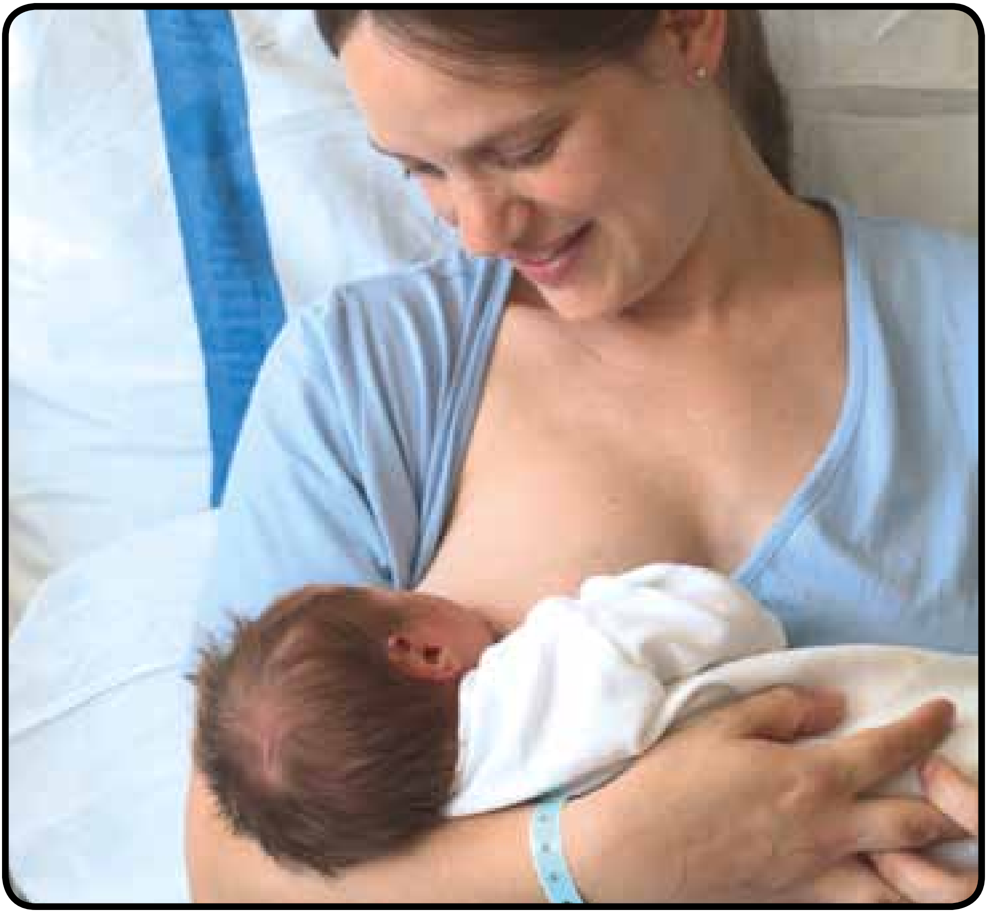

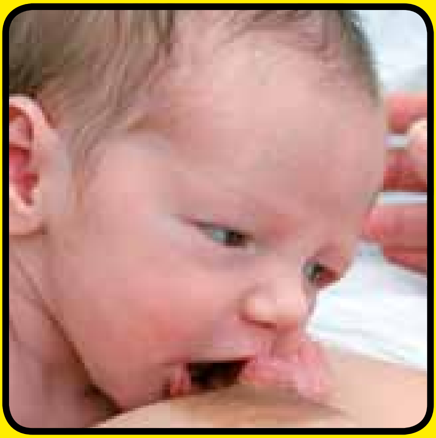

1. Hold your baby's whole body close with his nose level with your nipple.

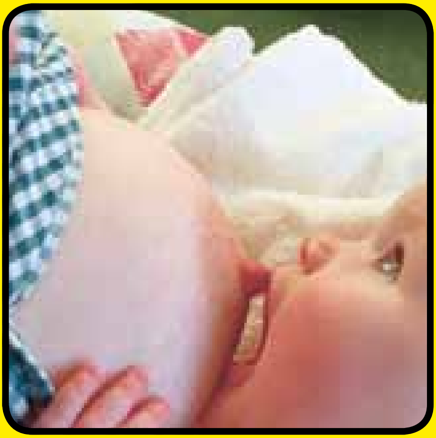

2. Let your baby's head tip back a little so that his top lip can brush against your nipple. This should help your baby to make a wide open mouth.

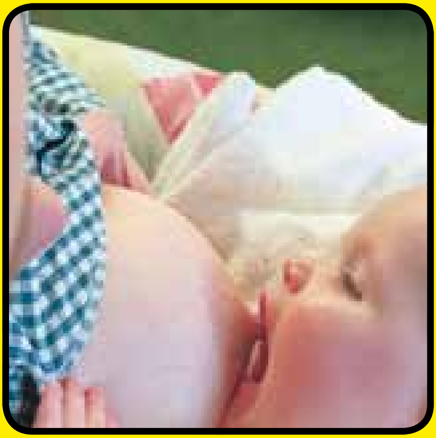

3. When your baby's mouth opens wide, his chin is able to touch your breast first, with his head tipped back so that his tongue can reach as much breast as possible.

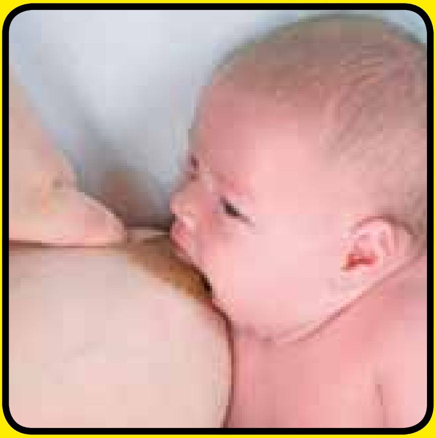

4. With his chin firmly touching and his nose clear, his mouth is wide open and there will be much more of the darker skin visible above your baby's top lip than below his bottom lip. Your baby's cheeks will look full and rounded as they feed.

signs that your baby is feeding well

- Your baby has a large mouthful of breast.
- Your baby's chin is firmly touching your breast.
- It doesn't hurt you when your baby feeds (although the first few sucks may feel strong).
- If you can see the dark skin around your nipple, you should see more dark skin above your baby's top lip than below your baby's bottom lip.
- Your baby's cheeks stay rounded during sucking.
- Your baby rhythmically takes long sucks and swallows (it is normal for your baby to pause from time to time).
- Your baby finishes the feed and comes off the breast on his or her own.

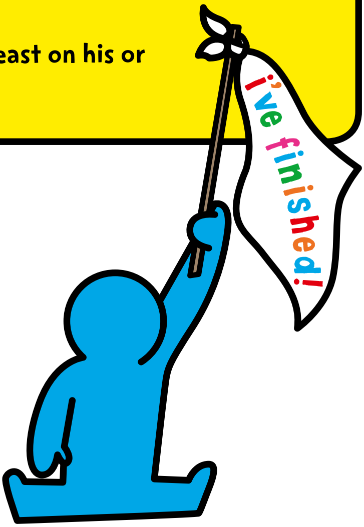

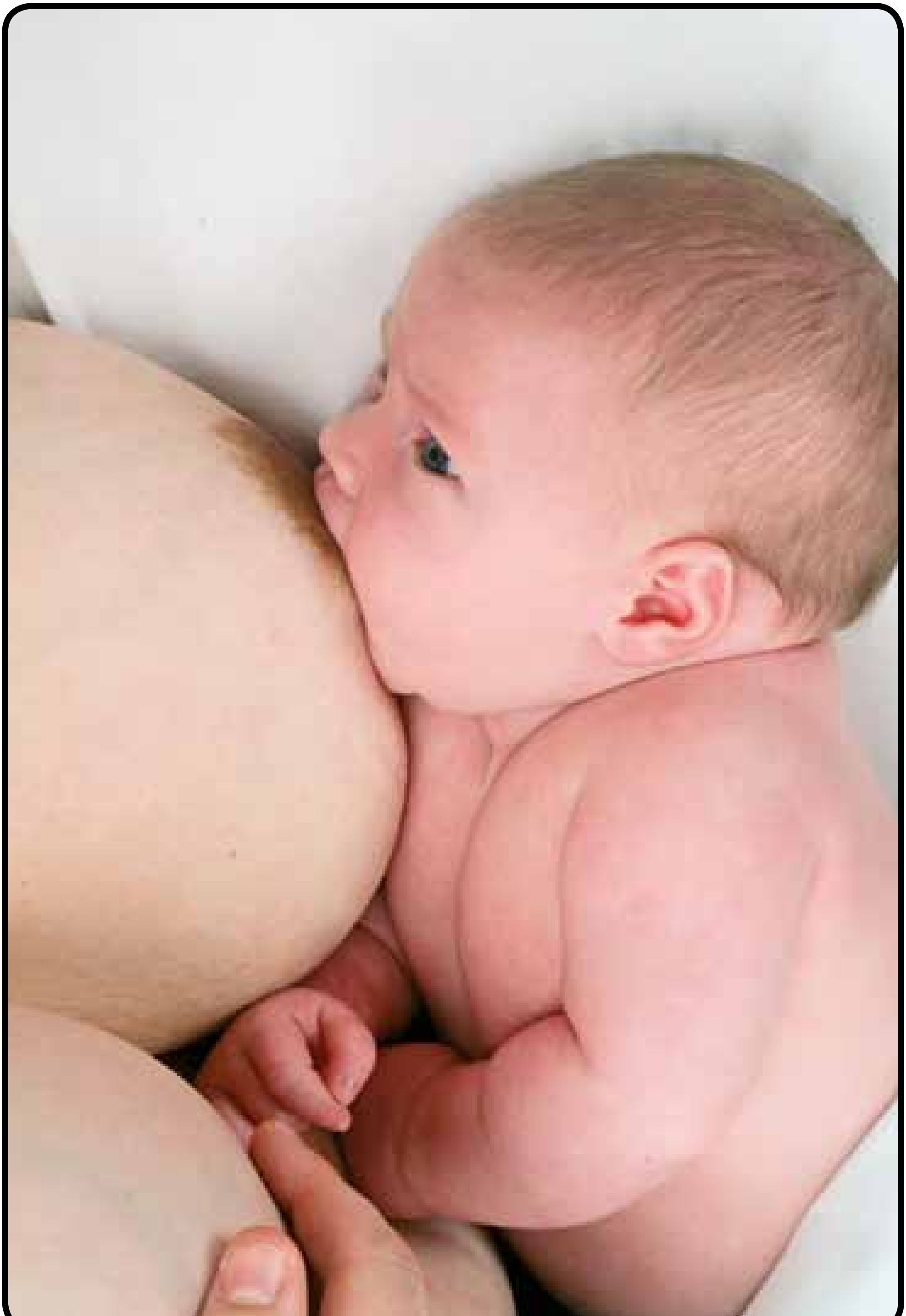

top tips

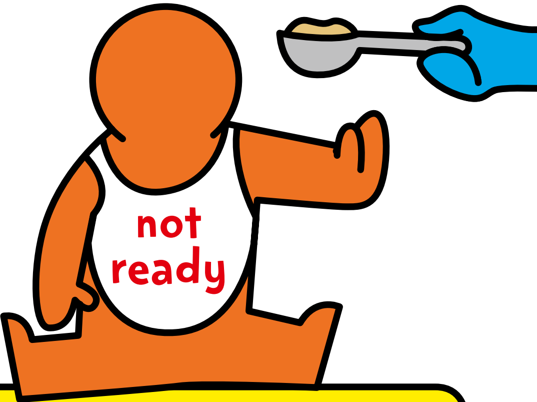

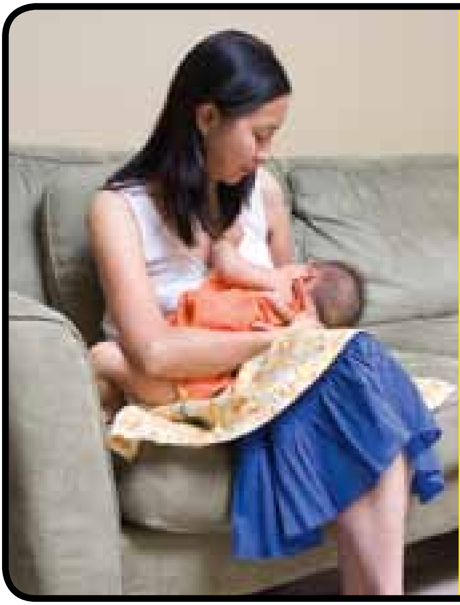

Try not to give your baby other food or drink

- The more mum's milk you give your baby, the more milk you will produce. Giving other food or drink will reduce your milk supply.
- If you give your baby less mum's milk, it will not protect your baby against illness as effectively.
- Feeding your baby solid food before they are ready (they are ready at around 6 months) could lead to him or her getting an upset tummy.

Make sure your baby is properly attached to your breast

(see pages 8–11)

- You will have a good supply of milk and your baby will get a good feed.
- It will help stop your breasts getting sore.

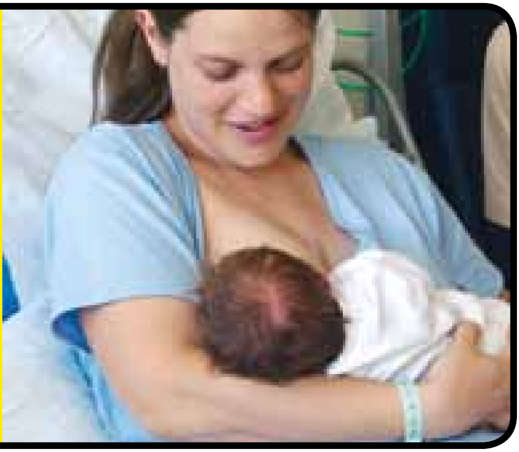

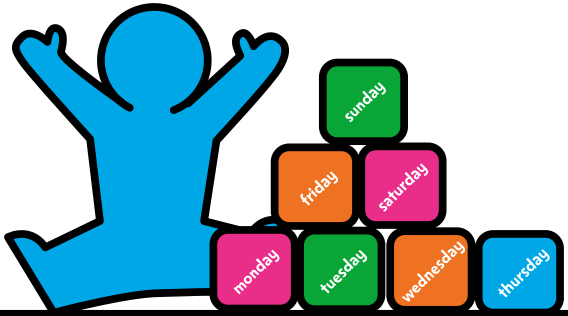

Try not to give a dummy before breastfeeding is established – usually around a month

- Babies who have a dummy sometimes find it difficult to remember how to attach to mum's breast.
- Your baby will be less likely to feed when they need to, so won't take in as much milk.

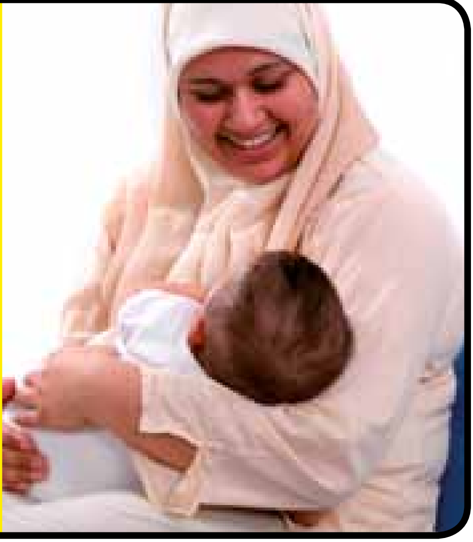

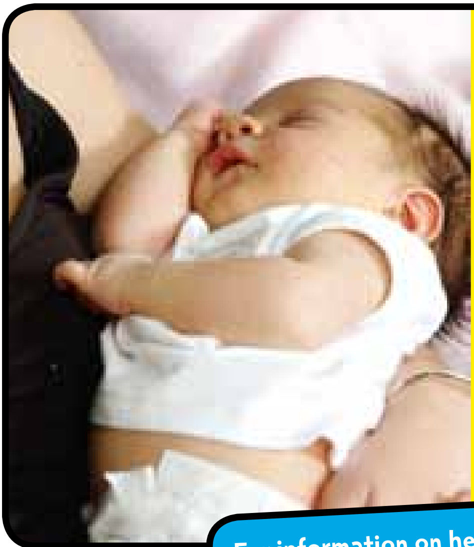

Don't be scared to ask for help

It can take a while before you feel confident. You can ask your midwife, health visitor or peer supporter to help you with breastfeeding, or call the National Breastfeeding Helpline: 0300 100 0212*

You can also call Start4Life on 0300 123 1021* or visit www.nhs.uk/start4life

For information on healthy eating for you while breastfeeding, see www.nhs.uk/conditions/pregnancy-and-baby

expressing milk

What does 'expressing milk' mean?

- Expressing milk means squeezing milk out of your breast.
- You can express milk by hand or with a hand pump or an electric pump.
- Different pumps suit different women, so ask for information to compare them.
- A pump needs to be clean and sterilised each time it is used.

Why express milk?

If you express milk, your baby will still be able to have mum's milk even if somebody else is feeding them.

This may be useful if you are away from your baby or returning to work.

It's best to wait until your baby is a little older before regularly expressing milk, so you have a chance to get feeding going well first.

Why express by hand?

If your breasts feel uncomfortably full.

If your baby isn't sucking well but you still want to give him mum's milk.

If you don't want to buy or use a pump to express milk.

In the first few days it is easier to express by hand.

How to express milk by hand

1. Have a clean sterilised container to hand before you start.

2. Cup your breast and feel back from the end of the nipple to where the texture of your breast feels different.

3. Using your thumb and the rest of your fingers in a C shape, gently squeeze this area – this shouldn't hurt.

4. Release the pressure and then repeat again and again, building up a rhythm. Avoid sliding your fingers over the skin. At first, only drops will appear, but just keep going as it will help build up your supply. With practice and a little more time, milk will flow freely.

5. When the flow slows down, move your fingers round to try a different section of your breast and repeat. When this happens again swap to the other breast. Keep changing breasts until the milk is dripping very slowly or stops altogether.

6. If the milk doesn't flow, try moving your fingers slightly towards the nipple or further away, and try a gentle breast massage.

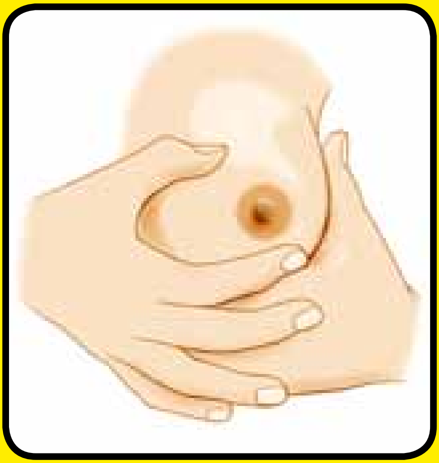

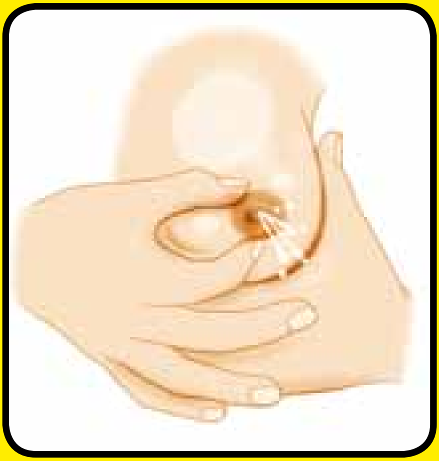

Storing milk

- Remember to use a sterilised container to put the milk in.
- You can store mum's milk in the fridge for up to 5 days at $4^{\circ}C$ or lower (usually at the back, never in the door).
- Mum's milk can be stored for 2 weeks in the ice compartment of a fridge or for up to 6 months in a freezer.
- Defrost frozen mum's milk in the fridge. Once thawed, use it straight away.
- If your baby prefers, you can warm the milk up to body temperature before feeding. Never heat milk in the microwave as it can cause hot spots which can burn your baby's mouth.

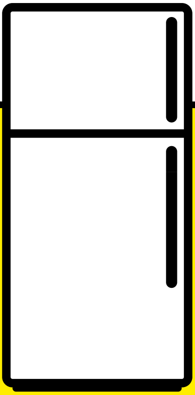

Expressing milk for a baby who is premature or ill

If your baby has to stay in hospital, it is important to start expressing your milk as soon as possible after your baby is born.

In order to ensure that you produce plenty of milk, you will need to express at least 8 times over 24 hours, including during the night.

Ask the hospital staff about holding your baby in skin-to-skin contact. This can help with bonding and keeping up your milk supply.

If you are freezing your milk because your baby is premature or ill, ask the staff caring for your baby for advice.

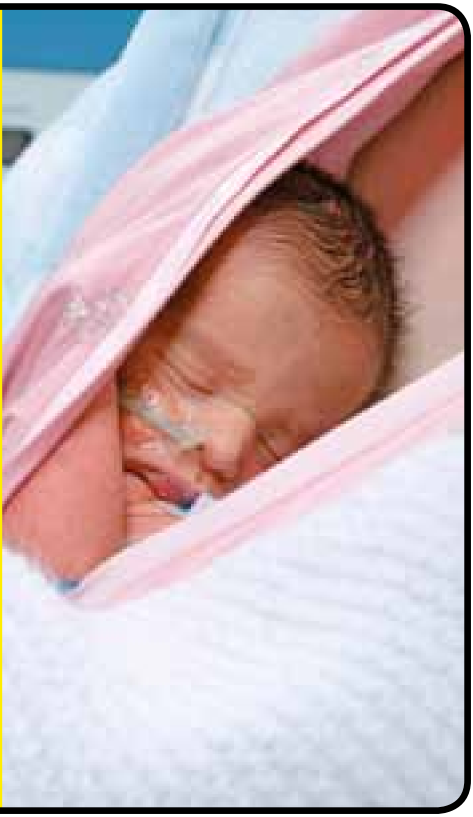

how do i know my baby is getting enough milk?

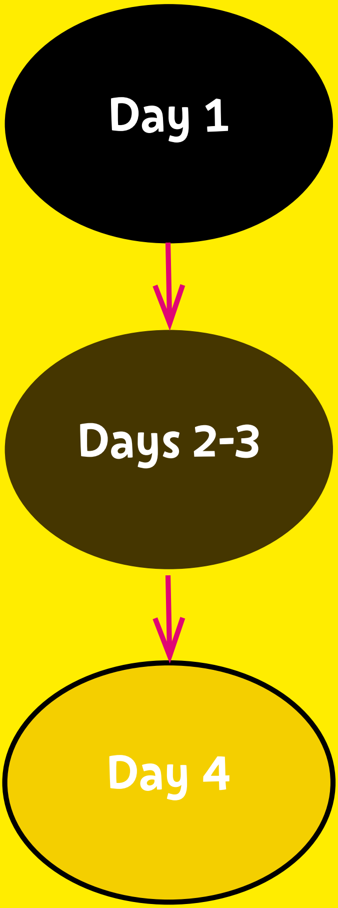

- Your baby should be healthy and gaining weight after the first 2 weeks.
- In the first 48 hours, your baby is likely to have only 2 or 3 wet nappies. Wet nappies should then start to become more frequent, with at least 6 every 24 hours from day 5 onwards.
- At the beginning, your baby will pass a black tar-like stool (poo) called meconium. By day 3, this should be changing to a lighter, runnier, greenish stool that is easier to clean up. From day 4 and for the first few weeks, your baby should pass at least 2 yellow stools every day. Remember, it's normal for breastfed babies to pass loose stools.
- Your breasts and nipples should not be sore. If they are, do ask for help.
- Your baby will be content and satisfied after most feeds and will come off the breast on their own.
- If you are concerned about any of these points, speak to your midwife or health visitor.

For online information about breastfeeding, visit www.breastfeeding.nhs.uk

Mum's milk is the healthiest way to feed your baby. If you decide not to breastfeed or to stop breastfeeding, it is possible to restart but it will be difficult. Giving infant formula to a breastfed baby will reduce your supply of mum's milk.

You do not need to eat any special foods while breastfeeding, but it is a good idea for you, just like everyone else, to eat a healthy diet. It is recommended that all pregnant and breastfeeding women take a daily supplement of 10 mcg of Vitamin D.

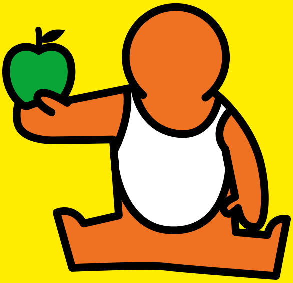

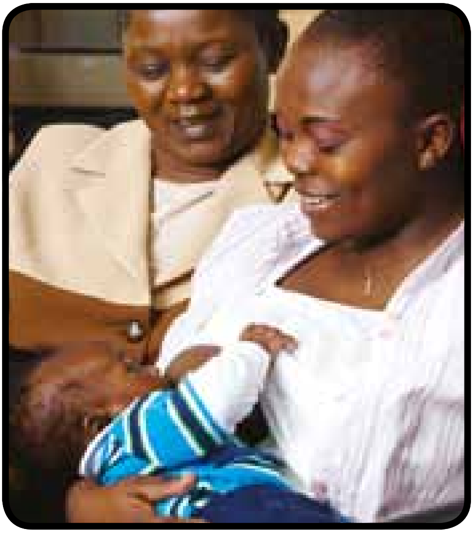

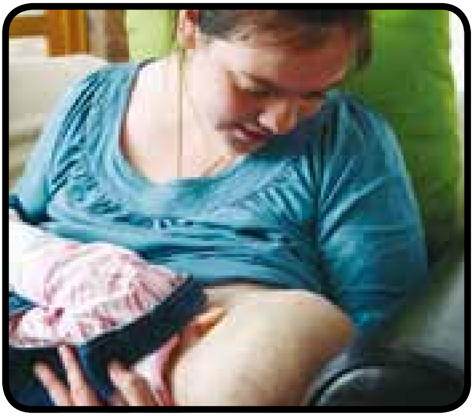

how can dads and partners support breastfeeding?

As a dad your role is to respond to your baby's need for love, comfort and security. Your support and understanding will help your partner breastfeed your baby.

Women are much more likely to breastfeed for longer when they have their partner's support. This leads to a greater sense of achievement for mum, and health benefits for both mum and baby. It can be a bonding experience for the whole family.

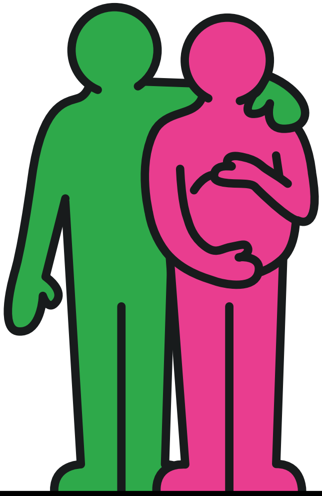

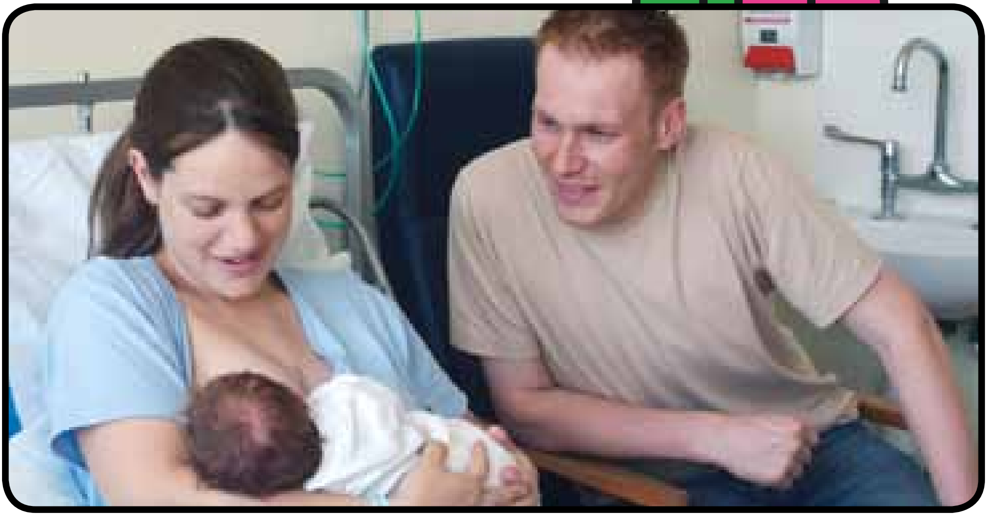

Practical ways partners can help

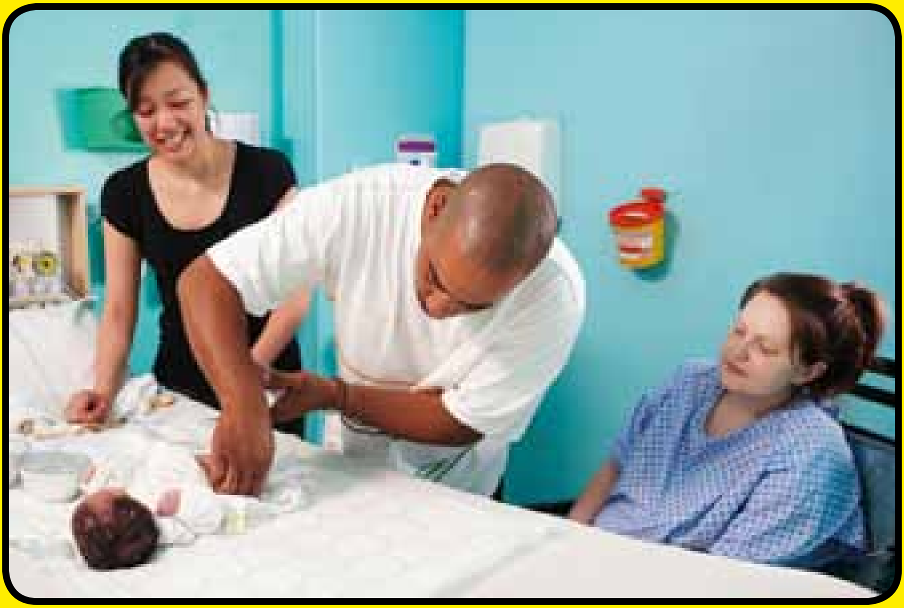

Go to antenatal or breastfeeding sessions. Some sessions are organised especially for dads. Ask your midwife, or at your local Children's Centre for further details. Learning the same information as your partner and discussing it together can be really helpful, especially in the early days when everything is very new and may sometimes feel overwhelming.

Give emotional and practical support. It can be a really hard time in the early days when babies and children take up all of your energy. But it doesn't last forever. Try to make time for each other when you can. Do little things to make each other feel cared for and included.

Arrange paternity leave so that you can get your new family off to a good start. Talk to your employer about paternity leave early on, so that you can plan how this best suits your family needs.

Try to make life easier. It's the little things that make a big difference. For example, preparing meals that fit around your baby's feeds, giving your partner a cup of tea while she feeds your baby, or even cutting up her dinner. She'll appreciate your help and being able to feed herself with her free hand.

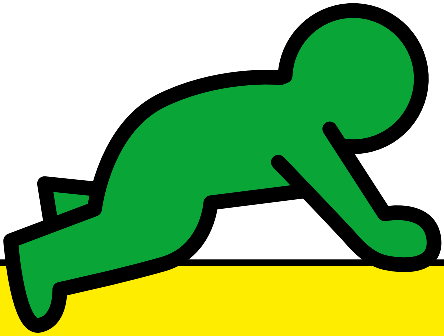

Provide some stress relief. If you already have young children, take the stress away from mum by keeping them entertained while she feeds the baby and give your children the security and reassurance they need that you still have plenty of time and love for them. Look at how you can get the chores done so that you and your partner have some time to relax.

You will want some time with your new baby so give them a cuddle and get involved in their care. Giving your baby their bath, changing nappies and being part of getting ready for bed are great ways of becoming close to your baby and sharing responsibilities with your partner.

If your partner decides to express her breast milk, you could give your baby a bottle of breast milk. Not all babies are keen to bottle feed; some prefer only to breastfeed. At around six months, your baby will be ready to try solid foods, and you can enjoy introducing your baby to a range of healthy foods and share this exciting time with them.

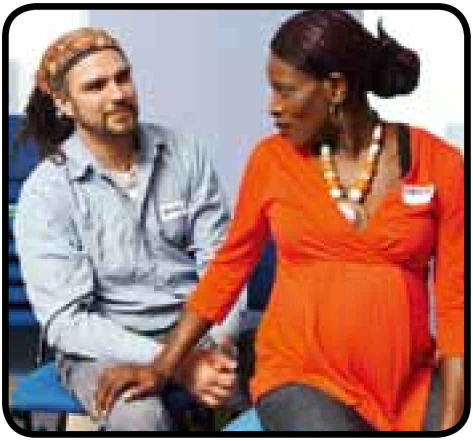

More information for dads

The Fatherhood Institute website at www.fatherhoodinstitute.org also provides useful information specifically for fathers. For a summary of research into the importance of fathers as part of breastfeeding, check out the website.

what is the information service for parents?

A free service for both mums and dads offering regular emails or texts about pregnancy, the first weeks of your baby's life and beyond. You can sign up to receive NHS advice and trusted information on a range of topics, including:

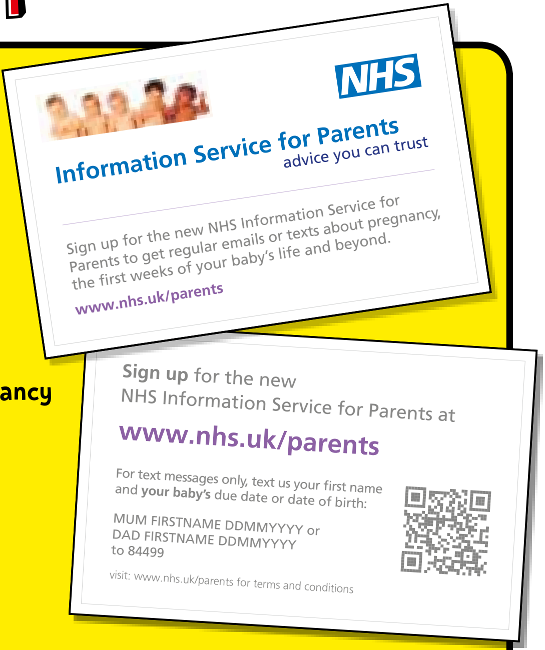

- How your baby's developing
- Keeping fit and healthy during pregnancy
- Getting ready for your baby's arrival
- Breastfeeding
- Immunisations
- Benefit advice
- Who's there to support you

The service can be accessed online at www.nhs.uk/InformationServiceForParents and includes video clips showing midwives demonstrating practical advice and other parents discussing their own experiences.

Who can sign up?

Mums and dads in England who are expecting a baby or who have a baby of up to four weeks old.

Benefits

- Links to trusted NHS information on pregnancy, babies and your own health
- Links to other reliable sources of information on parenting, covering topics such as benefits advice, childcare and relationship support
- Timely advice tailored to your baby's age
- All content via email and text is free, up to date and regularly reviewed

We're here to help you

If you need to talk to someone about anything in this booklet, or anything to do with breastfeeding your baby, please ask.

National Breastfeeding Helpline
0300 100 0212*
www.nationalbreastfeedinghelpline.org.uk

Staffed by volunteers from:

- Association of Breastfeeding Mothers
  www.abm.me.uk
- The Breastfeeding Network
  www.breastfeedingnetwork.org.uk

Start4Life
0300 123 1021*
www.nhs.uk/start4life

The Breastfeeding Network Supportline in Bengali/Sylheti
0300 456 2421*

NCT Breastfeeding Line
0300 330 0771*
www.nct.org.uk

La Leche League
0845 120 2918
www.laleche.org.uk

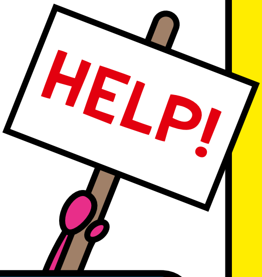

UNICEF UK Baby Friendly Initiative

UNICEF is the world's leading children's charity.

The Baby Friendly Initiative provides training and assessment for hospitals and community health services to enable them to give breastfeeding mothers the help and support they need to breastfeed successfully. Visit www.babyfriendly.org.uk

50% recycled
When you have finished with this booklet please recycle

*Calls to 03 numbers should cost no more than geographic 01 or 02 UK-wide calls and may be part of inclusive minutes subject to your provider and your call package. The National Breastfeeding Helpline is open from 9.30am to 9.30pm. The Start4Life lines are open from 9am to 8pm. Both are open 7 days a week.

© Crown copyright 2012. 4p 1m Oct 12 (ANT) (2900142)
Produced by Williams Lea for the Department of Health.

start 4 life
A good start for a healthier life
