# Supplementary material for: Using intervention mapping to develop a culturally appropriate intervention to prevent childhood obesity: the HAPPY (Healthy and Active Parenting Programme for Early Years) study
Source: Int J Behav Nutr Phys Act. 2013 Dec 28;10:142. doi: 10.1186/1479-5868-10-142 (PMC3895739; doi:10.1186/1479-5868-10-142)
Supplement: Additional file 4 — a. MASTER HAPPY Antenatal Manual FINAL VERSION. b. MASTER HAPPY Postnatal Manual FINAL VERSION. c. HAPPY manual resources. [file 1479-5868-10-142-S4.zip › 1523029418990916_add4c4.zip!/Values and beliefs about bringing up children handout.doc]

**Values and Beliefs about Bringing Up Children**

**1. Should you go immediately to a crying baby?**

It isn't possible to spoil new babies - they only cry when they need something, whether it's food, a cuddle or your company.

**2. Is breast feeding best for babies?**

Yes- breast milk is the perfect food for newborn babies, and the close cuddles and eye contact while feeding are enjoyable for mothers and babies. If you do decide to bottle feed, make sure you provide the same close cuddles as you would when breastfeeding.

**3. Should you give your baby a dummy?**

It's up to you - provided the dummy is kept clean and sterilised. On the plus side, sucking is a great comfort and many babies use their own thumbs or fingers once they get the hang of it. On the downside, you cut down opportunities for babbling conversation later on and you may not like the look of a dummy. Opinions are divided whether long term use can affect teeth but you certainly don't have to worry about that for a very young baby.

**4. Should your baby sleep in your bedroom**?

Many parents find it easier in the early weeks when night feeds are frequent - do what feels right for you - it is up to you. Babies can move in to a room of their own when you feel the time is right.

**5. How do you deal with sleepless nights?**

In the early days it is probably best to try and rest when your baby is asleep. Babies don't know night from day for the first few weeks, and this will prevent you from becoming too exhausted. Now is the time to take up any offers of help to let you catch up on sleep.

**6. How would you deal with naughty behaviour?**

It is quite impossible for babies to be 'naughty' in the sense that they have no idea of the effect of their behaviour on others. They never cry or fuss to get at you. Even when babies become toddlers, they may behave in ways that can be maddening for adults, but this is almost always a normal part of their development - they simply can't help themselves. For ideas on positive discipline, see The Parenting Puzzle.

**7. Should you ever smack your baby or child?**

There is lots of evidence now that smacking is a very negative way to discipline children. It doesn't work, it leads to harder smacks, and to children feeling resentful. They may also copy the behaviour, and be aggressive with other children. Smacking or shaking babies could be dangerous and is totally unacceptable. For alternatives to smacking see The Parenting Puzzle, Session 6, on kinds of touch (PP.133-143)

**8. Are there particular dos and don'ts on your cultural or religious beliefs that are important to keep?**

It is important to discuss these with your partner to make sure you both agree.

**If you find you have different opinions, now's the time to reach a compromise - not while the baby is crying for your attention. It will be much easier looking after your baby if you have a clear, shared view of how to go about it and if you agree on who you both trust for help and advice.**
