# Supplementary material for: Using intervention mapping to develop a culturally appropriate intervention to prevent childhood obesity: the HAPPY (Healthy and Active Parenting Programme for Early Years) study
Source: Int J Behav Nutr Phys Act. 2013 Dec 28;10:142. doi: 10.1186/1479-5868-10-142 (PMC3895739; doi:10.1186/1479-5868-10-142)
Supplement: Additional file 4 — a. MASTER HAPPY Antenatal Manual FINAL VERSION. b. MASTER HAPPY Postnatal Manual FINAL VERSION. c. HAPPY manual resources. [file 1479-5868-10-142-S4.zip › 1523029418990916_add4c5.zip!/Physical activity for new mums handout.pdf]

Preventing back pain

The following tips will help prevent an aching back:

- While feeding, always sit with your back well supported and straight. Place a pillow or cushion behind your waist.
- Kneel or squat to do low-level jobs, such as bathing your baby or picking things up off the floor. Avoid bending your back.
- Make your knees work instead. Change nappies on a waist-level surface or while kneeling on the floor.

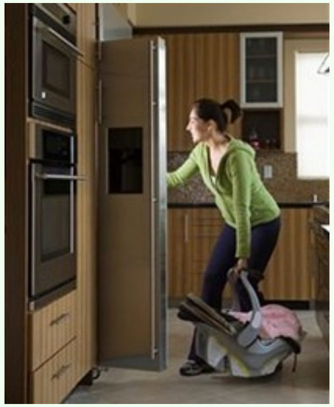

- To lift heavy objects, such as a carrycot or an older child, bend your knees, keep your back straight and hold the object close to your body. Make your thigh muscles work as you lift.
- Keep a straight back when you push a pram or buggy. Alternatively, carry your baby in a sling.

Visit: the NHS Options website to see the video clip about exercising for new mums again:

http://www.nhs.uk/Planners/birthtofive/Pages/Healthydietandfitness.aspx

HAPPY

Healthy and Active Parenting Programme for early Years

eat well, drink well, play well

Physical activity for new mums

When you're feeling tired, being active or taking more exercise may seem like the last thing you need, but activity can relax you; it can help your body recover after childbirth, keep you fit or improve your fitness, and make you feel better and more energetic.

When can I start exercising?

You can begin practising your pelvic floor exercises from day one. Once you feel comfortable with pelvic floor exercises you can begin to work on your lower tummy muscles (instructions are given inside this leaflet). It's fine to do these gentle toning exercises in the first six weeks.

Walking and pushing the pram briskly is great exercise and safe to do in the first 6 weeks. You can begin walking again when you feel ready to do so.

For other more strenuous exercises wait until after your six-week postnatal check before you start again.

If you had a caesarean delivery or a difficult pregnancy your recovery time will be longer. You should be able to start exercising again at around 10 weeks postnatal. Make sure you talk to your midwife or health visitor before starting anything too strenuous.

When your lochia (postnatal bleeding) has stopped, you can try swimming. Swimming is good exercise and it's relaxing too.

What should I be aware of before exercising?

Be careful not to stretch or twist too much. Your lower back and stomach muscles are weaker than they used to be and your joints are also pliable so it's easier to injure yourself.

Be gentle with yourself as you get back into exercise. If your joints are achy or you are feeling extremely tired after exercise you are doing too much. If you bleed after exercise you should visit your GP.

You will need a good supportive bra. Your bra size is likely to have changed and you will probably need to be measured for a new one. A sport bra can compress your breasts so if you are breastfeeding only wear it whilst you are exercising.

If you are breastfeeding you might want to plan your exercise for after a feed as this will be more comfortable for you.

Deep stomach exercise

This exercise will help to firm your stomach:

- Lie on your side with your knees slightly bent.
- Let your tummy sag and breathe in gently.
- As you breathe out, gently draw in the lower part of your stomach like a corset, narrowing your waistline.
- Squeeze your pelvic floor at the same time.
- Hold for a count of 10 and remember to keep breathing, then gently release. Repeat 10 times.

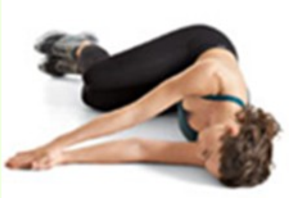

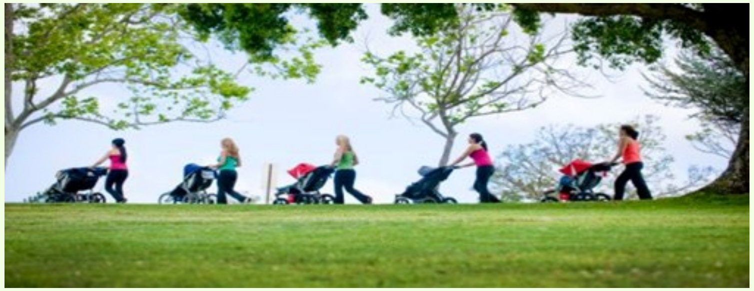

Pelvic floor exercise

Your Pelvic floor muscles support and protect your bowels, uterus and bladder. Hormonal changes in pregnancy and the pressure of your baby on the pelvic floor can loosen these muscles.

This exercise will strengthen your pelvic floor muscles. You can do this exercise either sitting or standing.

To find your pelvic floor muscles imagine you're stopping your urine flow when you pee, the muscles contracting are your pelvic floor.

- Squeeze and draw up the muscles.
- Do it quickly, tightening and releasing the muscles immediately.
- Then do it slowly, holding the contractions for as long as you can (but not more than 10 seconds) before you relax.
- Do five lots of pelvic floor quick squeezes and five lots of slow squeezes five times a day and build up to doing ten squeezes five times a day.
