# Supplementary material for: Using intervention mapping to develop a culturally appropriate intervention to prevent childhood obesity: the HAPPY (Healthy and Active Parenting Programme for Early Years) study
Source: Int J Behav Nutr Phys Act. 2013 Dec 28;10:142. doi: 10.1186/1479-5868-10-142 (PMC3895739; doi:10.1186/1479-5868-10-142)
Supplement: Additional file 4 — a. MASTER HAPPY Antenatal Manual FINAL VERSION. b. MASTER HAPPY Postnatal Manual FINAL VERSION. c. HAPPY manual resources. [file 1479-5868-10-142-S4.zip › 1523029418990916_add4c6.zip!/HELPYO~1.PDF]

Being active is important for your baby's health

Being physically active is important for young children because it helps them build and maintain a good level of health. It helps them develop movement skills, a strong heart, muscles and bones, keep to a healthy weight, social skills and much more. It is recommended that:

Babies
From playing on the floor to splashing in the pool, encourage your baby to be active from day one.

Toddlers
With lots of fun play from morning to bedtime, encourage your child to be active for at least 180 minutes (3 hours) every day.

Under fives
Your child should stay on the move and avoid sitting still for too long. Limit the time your child spends in a buggy, car seat or high-chair.

What types of movement does my baby need?

Babies should be able to move freely in safe spaces and especially have time on their tummy every day.

Babies need to practise important movements such as, reaching and grasping for objects; turning their heads, pulling and pushing and playing with toys and people.

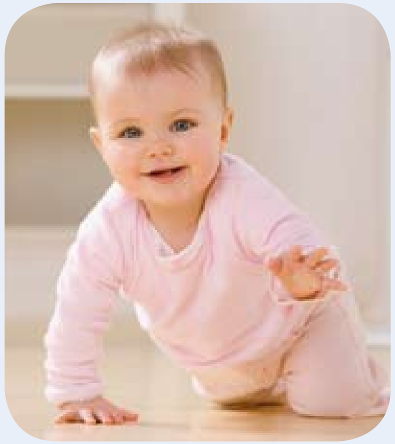

Don't forget healthy eating is important for babies too

Your baby's food needs will change a lot over their first year. **Breast milk is the best food for young babies, it is recommended for the first six months.**

Moving onto solid food (weaning) should be started when your baby is ready, usually around six months old. You know they are ready when they can stay in a sitting position with a steady head, recognise and pick up food, put it in their mouth themselves and then chew and swallow it. Your baby should not have solid foods before they are four months old.

First foods – begin with baby rice mixed with breast milk or formula, pureed or soft cooked sticks of fruit or root vegetables. Gradually move on to a wider range of foods and textures, from purees to mashed foods and then on to soft lumps. Aim for chopped (rather than mashed) foods between 10 and 12 months old.

Babies are very good at knowing when they have had enough to eat. Never force a child to eat.

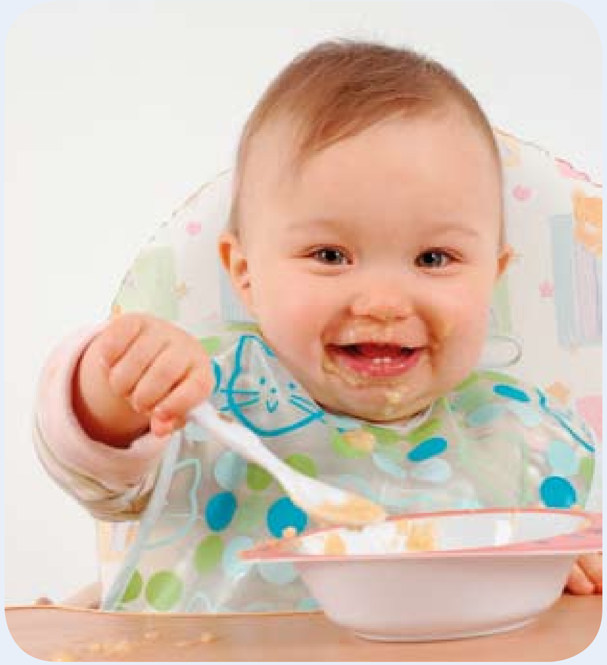

Published by BHF National Centre www.bhfactive.org.uk

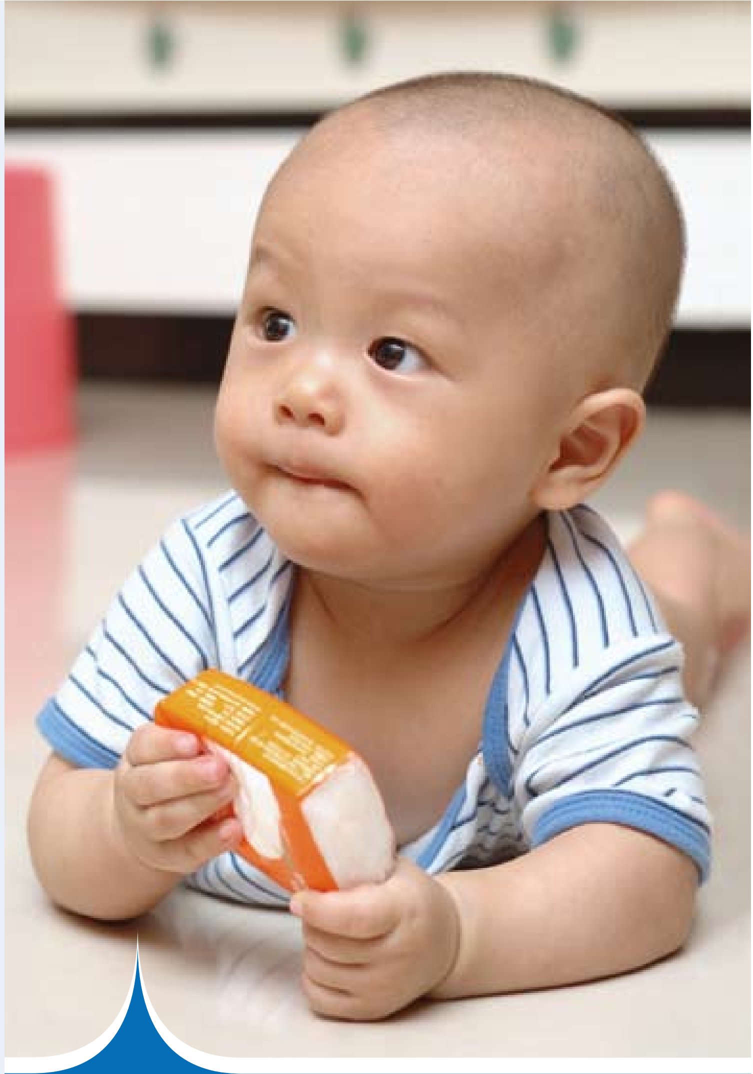

Help your baby move and play every day

Information for parents

BHF National Centre
physical activity+health

Funded by
British Heart Foundation

Tips and ideas for encouraging your baby to move

Provide plenty of tummy time

Tummy time is when your baby is placed or carried on their front. This helps babies use and strengthen their muscles, which they will need for crawling and other activities as they grow up including speech and writing. Very young babies can find it difficult to be on their tummy for a long time so try 'little and often'.

Remember tummy time is only for when babies are awake and supervised. Babies should always be placed on their backs to sleep.

- Place toys all around that encourage grasping, leg kicking and head lifting.
- Try shaking toys, playing games such as peekaboo or singing nursery rhymes.

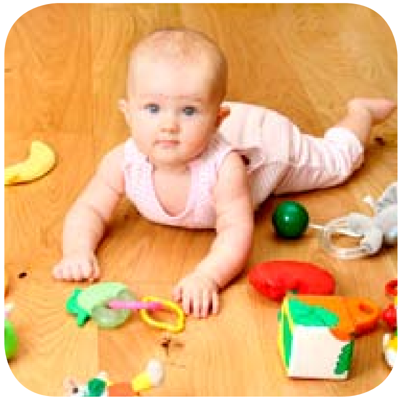

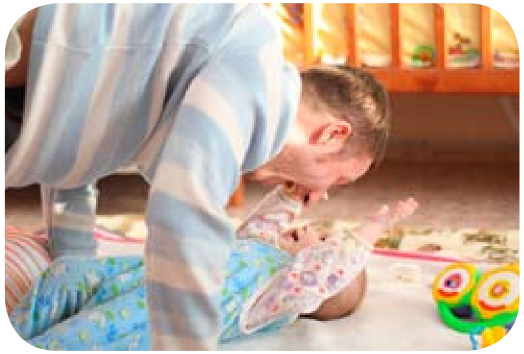

Provide lots of soft areas

- Place a blanket on the floor and encourage your baby, on their front or back, to practise kicking, rolling, reaching for objects and turning their head towards a sound.
- Let your baby wear clothing which allows them to move freely.

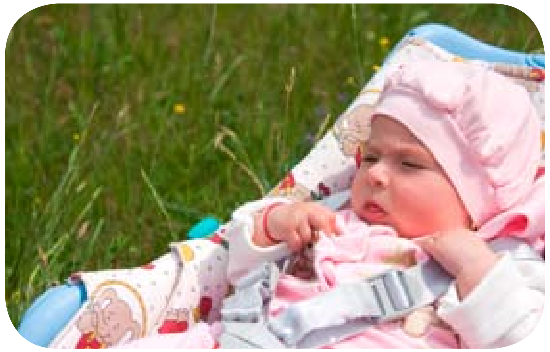

Take your baby outside

- New sounds, smells and sights outside will be interesting for your baby and encourage them to move.
- For young babies, place a blanket in a safe place in the garden or in a park. Try placing under a tree so that your baby can see leaves and branches moving.

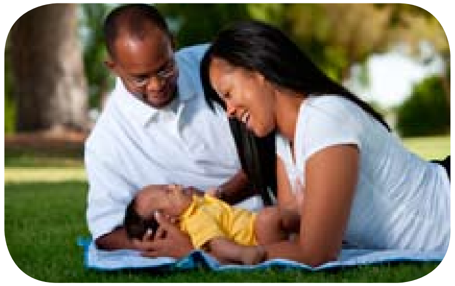

Reduce sitting time

There are times when your baby will need to sit in a buggy, highchair or car seat, but try to reduce these times and break them up where possible.

- Try and take breaks on long car trips – give your baby time out of the car seat.
- Reduce time spent in walkers or baby bouncers as these reduce how much your baby can move.
- Consider not leaving the television on during mealtimes or when your child is playing.

Give your baby suitable toys

- Toys don't need to be expensive, they can be everyday objects.
- **Noisy toys** can help movement and development linked to hearing and balance. Try rattles, wooden spoons and saucepans, playing music and singing nursery rhymes.
- **Textured toys** will help develop your baby's sense of touch. Place toys slightly out of reach so your baby has to stretch to get them.
- Once your baby can move, use tunnels or empty boxes to crawl through, cushions to climb over and furniture they can pull up on.
- **Moving toys** can help develop your baby's sight. For very young babies use a hanging mobile, a play gym, playing peekaboo or sharing colourful picture books together. Once your baby can sit, roll a ball towards them or dangle scrunched paper in a sock and encourage them to hit it.

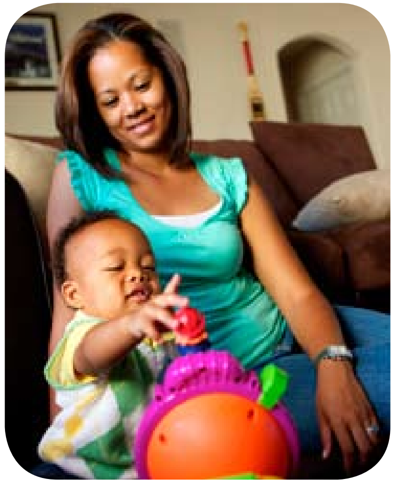

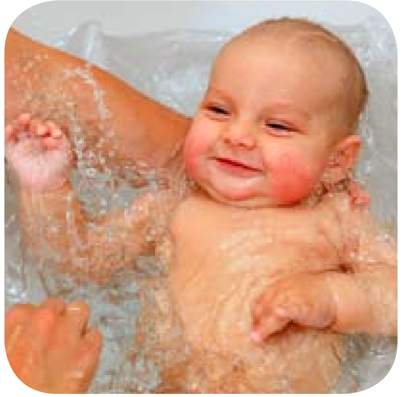

Enjoy splash time

- Make bath times fun with lots of splashing, fun play and singing.
- Visit your local pool, they may have parent/carer and baby sessions.
