# Supplementary material for: Using intervention mapping to develop a culturally appropriate intervention to prevent childhood obesity: the HAPPY (Healthy and Active Parenting Programme for Early Years) study
Source: Int J Behav Nutr Phys Act. 2013 Dec 28;10:142. doi: 10.1186/1479-5868-10-142 (PMC3895739; doi:10.1186/1479-5868-10-142)
Supplement: Additional file 4 — a. MASTER HAPPY Antenatal Manual FINAL VERSION. b. MASTER HAPPY Postnatal Manual FINAL VERSION. c. HAPPY manual resources. [file 1479-5868-10-142-S4.zip › 1523029418990916_add4c7.zip!/The BIG balancing act handout.doc]

**The BIG Balancing Act - Facts about Energy and Body Weight**

- Food and many drinks supply our bodies with energy ( measured in calories or kcals)
- Energy is used to keep our bodies functioning 24/7, growth, repair of body tissues and keeping us at the right temperature as well as for physical activity
- An average man needs to eat 2500 kcals a day	to stay the same weight

An average woman needs 2000kcals a day (more if breastfeeding –but body stores laid down in pregnancy provide a lot of the extra)

**IF we eat (or drink) more energy than we use –the excess is stored as body fat.**

Some body fat is essential for health –too much is NOT-and causes overweight and obesity

1 lb of body fat is a store of 3500 kcals	(1kg is a store of over 7000kcals)

This sounds like a lot of extra food –and it is if you eat it in one go

But the effect of small (& even unnoticed) changes in what you are eating or the amount of physical activity you are doing can easily add up over time

Eg an extra biscuit or two, portions getting gradually larger, driving to work instead of walking part of the way, eating like you did as a teenager after you have stopped growing in height,

| **Excess calories eaten each day** | **Weight gain after 1 year** | **Weight gain after 10 years** |
| --- | --- | --- |
| 50 | 2.6 kg  (5.7lbs) | 26 kg  (over 4 stone) |
| 100 | 5.2 kg  (11.5lbs) | 52kg  (over 8 stone) |

**Q. How much chocolate gives you 100 Kcals?**

Turn over for the answer and to find out how much of other foods gives you 100kcals and how much activity uses up 100kcals

There are also some top tips for getting YOUR energy balance right!

**Eating 100 kcals**

1 fun-sized chocolate bar

Half pint beer

2 portions fruit

1small slice garlic bread

1 slice toast and low fat spread

Half a bag of crisps

**Using 100 Kcals (average adult)**

40 mins ironing

10 mins digging

10 mins running (11m/hr)

50 mins standing

65 mins sitting

20 mins cycling (moderate)

22 mins walking (average pace)

5 mins walking uphill

**TO stay the same weight	Energy IN = Energy OUT**

**To lose some weight	Energy IN must be LESS than Energy Out**

**Tips to get your energy balance right**

- Get (more) active – walking is a good start
- Eat a balanced healthy diet with plenty of fruit and vegetables
- Fatty & sugary foods and alcohol are very rich in calories –cut them down
- Watch your portion sizes
- Choose snacks and drinks carefully
