# Supplementary material for: Using intervention mapping to develop a culturally appropriate intervention to prevent childhood obesity: the HAPPY (Healthy and Active Parenting Programme for Early Years) study
Source: Int J Behav Nutr Phys Act. 2013 Dec 28;10:142. doi: 10.1186/1479-5868-10-142 (PMC3895739; doi:10.1186/1479-5868-10-142)
Supplement: Additional file 4 — a. MASTER HAPPY Antenatal Manual FINAL VERSION. b. MASTER HAPPY Postnatal Manual FINAL VERSION. c. HAPPY manual resources. [file 1479-5868-10-142-S4.zip › 1523029418990916_add4c7.zip!/Introducing solid foods handout Start 4 Life.pdf]

NHS

introducing solid foods

Giving your baby a better start in life

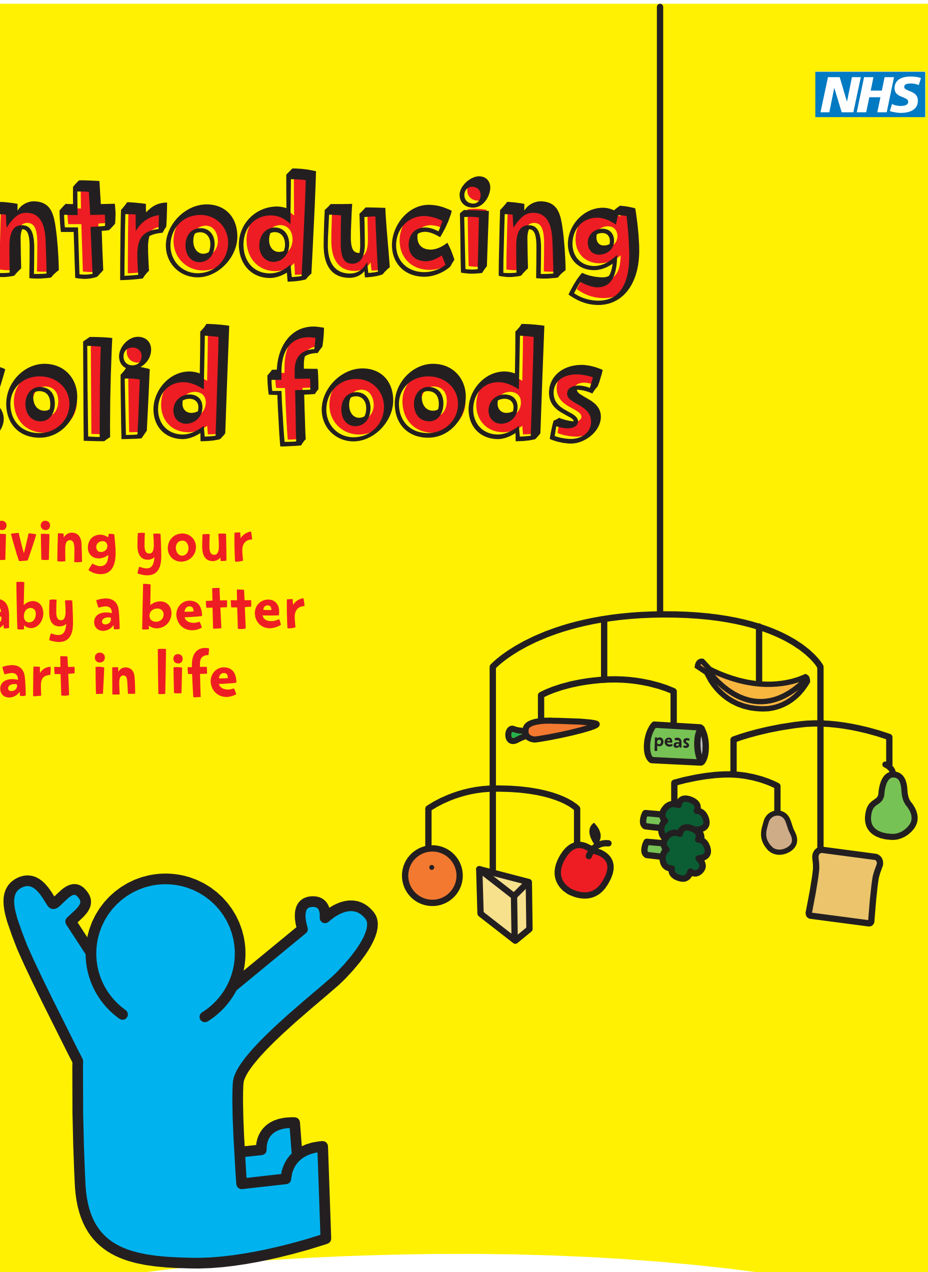

UK BABY FRIENDLY INITIATIVE

unicef

start 4 life

A good start for a healthier life

56447 COI Solid Food.indd 1 11/02/2011 12:52

what is start4life?

Start4Life is an initiative to help you give your baby a healthier start in life. There are 6 Start4Life building blocks, based on the latest infant health research:

The Start4life building blocks

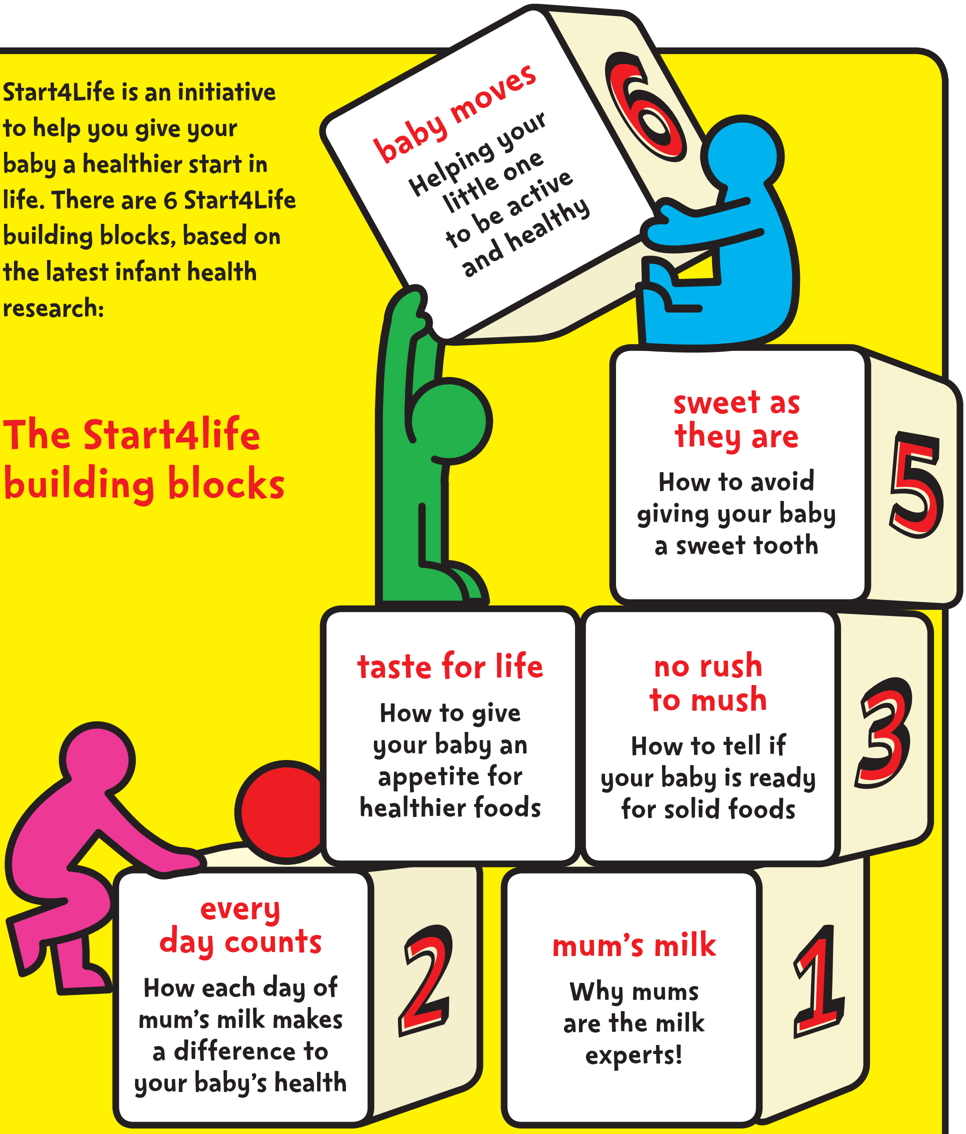

For more information please visit www.nhs.uk/start4life or refer to the 'Building blocks for a better start in life' booklet (product code: C4L176).

56447 COI Solid Food.indd 2 11/02/2011 12:52

starting your baby on solid foods

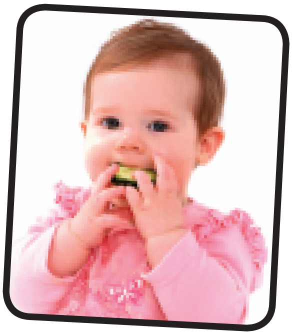

Introducing your baby to solid foods, often called weaning onto foods, should start when your baby is around 6 months old.

It's a really important step in your baby's development and it can be great fun to explore new flavours and textures together. This booklet is here to help guide you through this exciting time, so you can be sure you're giving your baby a healthy start in life.

To begin with, how much your baby takes is less important than getting them used to the idea of eating. They will still be getting most of their nutrition from milk. Babies don't need three meals a day to start with, so you can start by offering foods at a time that suits you both.

Gradually, you'll be able to increase the amount and variety of food your baby eats until they can eventually eat the same as the rest of the family, in smaller portions.

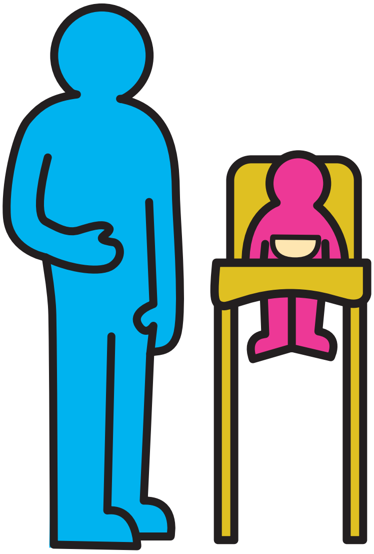

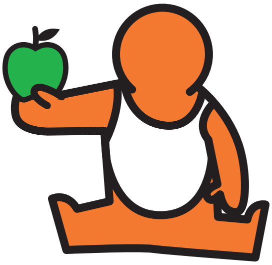

in this booklet

What is Start4Life? 2

Starting at around 6 months 5

3 signs your baby is ready 6

Some signs mistaken for a baby being ready 7

First foods first! 8

Getting started 9

Top tips to encourage and explore 10

Things to watch 12

Snack time 13

They're sweet enough already! 14

Allergies 15

Safety and hygiene 16

Vitamins 19

56447 COI Solid Food.indd 4 11/02/2011 12:52

no rush to mush

Starting at around 6 months

The latest research reviewed by the World Health Organization shows that babies need nothing but mum's milk or infant formula for the first 6 months of life. This gives a baby's digestive system time to develop so that they cope fully with solid foods. This includes solid food made into purees and cereals added to milk.

Why it pays to wait until they're ready

If you are breastfeeding, feeding only mum's milk up to around 6 months will give them extra protection against infection, and it will continue to protect them for as long as you carry on.

Whether your baby has mum's milk or infant formula, waiting till your baby is ready for food will save a lot of time too, as they will quickly be able to feed themselves and with less mess, as they'll be able to swallow properly.

First foods

From around 6 months your baby's first foods can include soft cooked vegetables like parsnip, potato, yam, sweet potato or carrot. Soft fresh fruit like banana, avocado, peach or melon are good too. Babies often like to start eating these by having them as finger foods, or mashed. You can also spoon-feed your baby, although they will soon be able to do it for themselves.

Keep feeding mum's milk or infant formula as well, but don't give cow's milk as a drink until they are 1 year old.

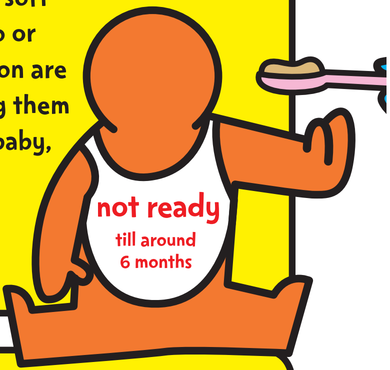

Finger foods

Finger food is food that's cut up into pieces big enough for your baby to hold in their fist and stick out of the top of it - pieces about the size of your own finger work well. Your baby learns to chew this way.

56447 COI Solid Food.indd 5 11/02/2011 12:52

no rush to mush

3 signs your baby is ready for their first food

Every baby is an individual, but there are 3 clear signs which, together, show your baby is ready for these types of first solid foods alongside mum's milk or infant formula.

Your baby is ready if they can:

Stay in a sitting position and hold their head steady

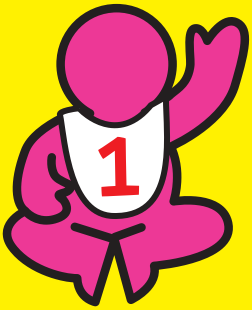

&

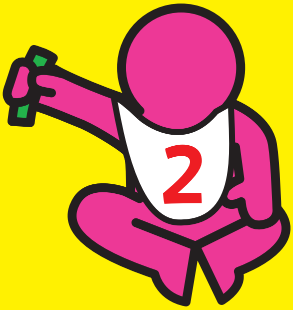

Co-ordinate their eyes, hand and mouth so that they can look at the food, pick it up and put it in their mouth all by themselves

&

Swallow food. Babies who are not ready will push their food back out, so they get more round their face than they do in their mouths!

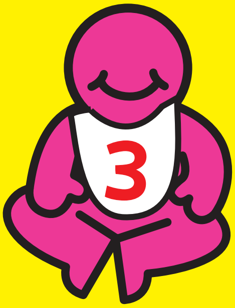

It's rare for these signs to appear together before 6 months.

See the safety and hygiene section on page 16.

56447 COI Solid Food.indd 6 11/02/2011 12:52

Some signs mistaken for a baby being ready for solid foods

- Chewing fists
- Waking in the night when they have previously slept through
- Wanting extra milk feeds

These are normal baby behaviours and not necessarily a sign of hunger, nor being ready to start solid food. Starting solid foods will not make them any more likely to sleep through. Sometimes a little extra milk will help until they are ready for food.

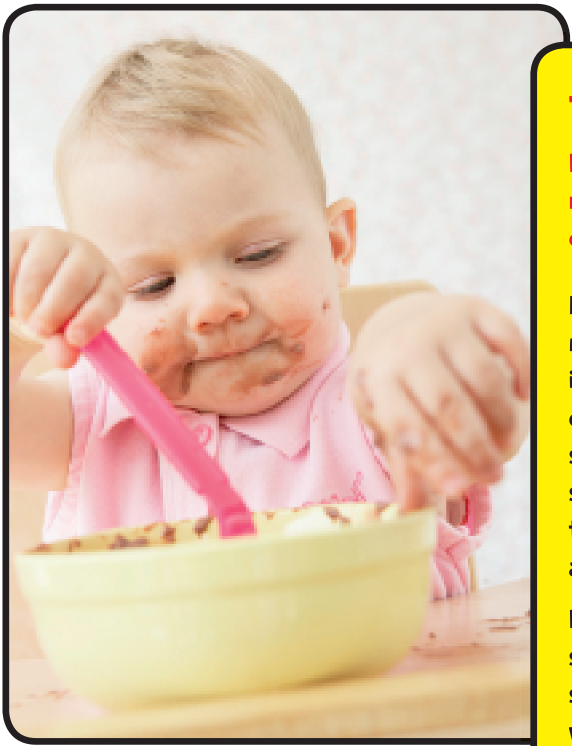

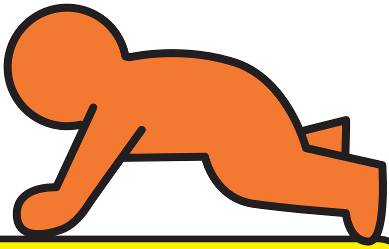

True or false?

Babies who are big for their age need solid foods earlier than other babies.

False. It's easy to see why people might think that. But remember, it's what's going on inside that counts. Babies are ready for solid foods when their digestive systems are developed enough to cope – and this is usually at around 6 months.

But every baby is an individual so if you think yours is ready, speak to a member of your health visiting team.

56447 COI Solid Food.indd 7 11/02/2011 12:52

taste for life

First foods first!

Here's a handy guide to what foods you should give:

From 0-6 months

Mum's milk or infant formula.

'Follow on' formula is definitely not suitable for babies under six months. You don't need to introduce it after six months either.

From 6 months

First foods: mashed or soft cooked sticks of fruit and vegetables like parsnip, potato, yam, sweet potato, carrot, apple or pear, all cooled before eating. Or try soft peach or melon, or grabbable bits of soft ripe banana or avocado. Or baby rice mixed with your baby's usual milk.

Next foods: soft cooked meat such as chicken, mashed fish (check very carefully for any bones), pasta, noodles, toast, pieces of chapati, lentils, rice and mashed hard boiled eggs. Also, full fat dairy products such as yoghurt, fromage frais or custard (choose products with no added sugar or less sugar). Cow's milk can be used in cooking or mixed with food from 6 months.

Cups: introduce a cup from around 6 months and offer sips of water with meals. Using an open cup or a free-flow cup without a valve will help your baby learn to sip and is better for your baby's teeth.

From 8-9 months

Gradually, in this time, your baby will move towards eating 3 meals a day. It will be a mixture of finger foods, mashed and chopped foods.

From 12 months

Your baby will now be eating 3 meals a day, chopped if required, plus mum's milk or cow's milk and healthier snacks like fruit, vegetable sticks or toast and rice cakes. They can now drink whole cow's milk and have full fat dairy products. Choose full fat because children under 2 need the extra fat and vitamins in full fat dairy products. From 2 years old if they are a good eater and growing well they can have semi-skimmed milk. From 5 years old, 1% or skimmed milk is ok.

56447 COI Solid Food.indd 8 11/02/2011 12:52

Getting started

- Let your baby enjoy touching and holding the food
- Always stay with your baby when they're eating in case they choke
- Don't force your baby – wait until the next time if they are not interested this time
- Your baby can feed themselves using their own fingers, as soon as they show an interest
- If you're using a spoon, wait for your baby to open their mouth before you offer the food. Your baby may like to hold a spoon too
- Start by offering just a few pieces or teaspoons of food, once a day
- If the food is hot, allow it to cool and test it before giving it to your baby
- Don't add salt, sugar or stock cubes to your baby's food or cooking water.

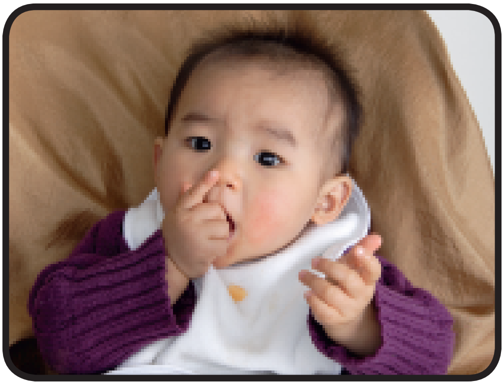

What milk when?

Only mum's milk or infant formula for around the first 6 months

Then mum's milk or infant formula alongside food from 6-12 months

Cow's milk can be mixed with food from 6 months

Whole cow's milk can be given as a drink from 1 year

Carry on with mum's milk for as long as you both want.

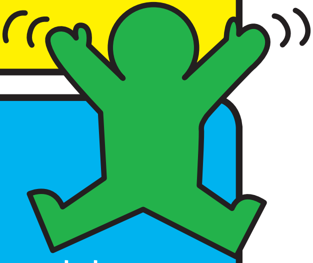

Try this

Start by offering cooked vegetables, soft pieces of fruit or a small amount of mashed fruit or vegetable. Try it after a milk feed (or try in the middle of one) and see what works best for your baby.

True or false?

My baby needs to start eating before I go back to work.

False. Your baby can begin eating food any time you are together to fit in around your work. They will soon get the hang of it.

taste for life

Top tips to encourage and explore

Starting on solid foods is a real milestone for you and your baby. It's their first chance to explore the wonderful variety of food textures and tastes that the world has to offer. Have a look at our top tips for more ideas.

Healthy eaters start here!

Babies like the food they get used to so give them as many different, healthier foods as you can – this way they are more likely to keep eating them as they grow up. It's a great habit to get into and one that will hopefully make your life a little easier as they get older. Also, don't give them foods or drinks with added sugar or salty or fatty foods, as this will make them more likely to want them as they get older.

Take time

Allow plenty of time for eating, especially at first. Rushing or forcing your baby could lead to problems. Go at your baby's pace and stop when your baby shows you they have had enough.

It's messy!

It can get messy but this is an important part of your baby's development. You may want to cover the floor with newspaper or a protective mat to make clearing up easier!

Show them

Babies copy their parents and other children so you can help them by showing them that you eat healthier foods.

Solid foods and milk

As your baby eats more solid food, they may want less milk at each feed, or even drop a milk feed altogether. Babies should have mum's milk (or infant formula) for at least the first year, and can carry on with mum's milk for as long as you both want.

Finger foods

Let your baby feed themselves with their fingers. This way they can show you how much they want to eat, and it gets them familiar with different types of food. It also makes eating more enjoyable.

56447 COI Solid Food.indd 10 11/02/2011 12:53

Eat together

Babies enjoy watching you eat, and learn from being a part of family meal times. Help them join in by talking to them and giving them food when you or the rest of the family is eating. Having mealtimes around the same time every day can make it easier for your baby to know when to expect to eat.

3rd time lucky... or 15th

Babies like to choose for themselves, and sometimes take their time getting used to different foods. So offer a small amount, lots of times, to let them gradually get used to new foods.

How much?

Don't worry if your baby hasn't eaten much in a meal or a day, what they eat over a week is more important.

Homemade is best

The best baby food is homemade from simple ingredients with no added sugar or salt. Any unused food can be kept in the fridge or frozen. Then all you have to do is reheat the amount you need. Quick and easy – and it's a lot cheaper than jars!

Tiny tums

Most babies know when they are full up, so don't make them finish a portion when they don't want to. Smaller, more frequent meals and healthier snacks will suit them better when they are young.

Sit up straight

Make sure your baby is sitting up straight so that they are able to explore foods better and are less likely to choke.

Jars or packets just sometimes

Baby food in jars or packets can be handy but portion sizes are often too big and much of it has the same texture. This might stop your baby from liking other foods. That's why jars are best left for when you don't have much time or you're out with your baby. Even if the label says from 4 months, the research reviewed by the World Health Organization shows that your baby is not usually ready for solid foods until around 6 months.

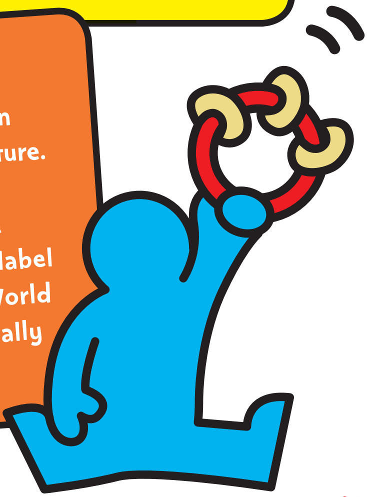

Things to watch

If after checking with your health visitor or doctor, you decide to introduce solid foods before 6 months, you should avoid giving your baby certain foods. These include foods that contain wheat, gluten, nuts, peanuts, peanut products, seeds, liver, eggs, fish, shellfish, cows' milk and soft or unpasteurised cheese.

Watch out for the following when starting to feed your baby

Sweet drinks like squash, fizzy drinks, milkshakes and some fruit juices can have lots of added sugar so avoid these if you can and protect your baby's teeth. Other drinks to avoid include tea, coffee, diet drinks, low-calorie drinks and no-added-sugar drinks. They will fill up your baby so they're not hungry for healthier food. Instead, offer sips of water from a cup with meals.

Cows' milk as a drink shouldn't be given before 1 year as it doesn't have the right balance of nutrients. Also iron from cow's milk is not absorbed as well as it is from mum's milk.

Sweet foods like chocolate, sweets, Indian sweets (mishit or mithai), chin chin, rusks, biscuits, cakes and pastries have lots of added sugar and fat. Babies don't need them.

Salt and salty food like cheese, bacon, sausages, chips with added salt, crisps, convenience food, takeaways, gravy and meals made with stock cubes are bad for babies, so limit these.

Choking can happen with hard foods, bones, small round foods like grapes and cherry tomatoes, and food with skin, like sausages. Peel the skin off fruit, vegetables and sausages and remove all bones from meat or fish. Avoid whole nuts – these shouldn't be given until your child is 5 years old.

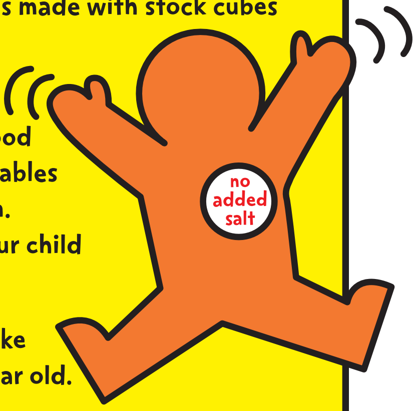

Honey occasionally contains bacteria which can make babies ill, so should not be given before they are 1 year old.

sweet as they are

Snack time

To give your baby the healthiest start, instead of chocolate, ice cream and crisps give these delicious, healthier snacks:

rice pudding or porridge (with no added sugar or salt)
canned fruit in fruit juice
fresh fruit
yoghurt (unsweetened)
toast, pitta or chapati fingers
unsalted rice cakes
bagels
small cubes of cheese

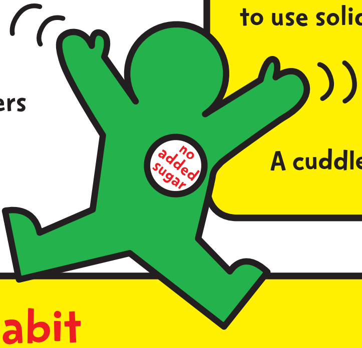

True or false?

You shouldn't give your baby extra solid foods to stop them crying.

True. Babies learn very quickly to use solid food or drinks from a bottle as a comforter. This could lead to weight problems later in life. A cuddle or mum's milk is better.

Get into the habit

It's much easier to get your baby on the right track now, rather than try and change what they eat later. Always offer water with food, rather than sugary drinks. And tempting though it is, avoid using food as a reward. Instead, spend time with them – you can take your children to the park, play a game or read a book.

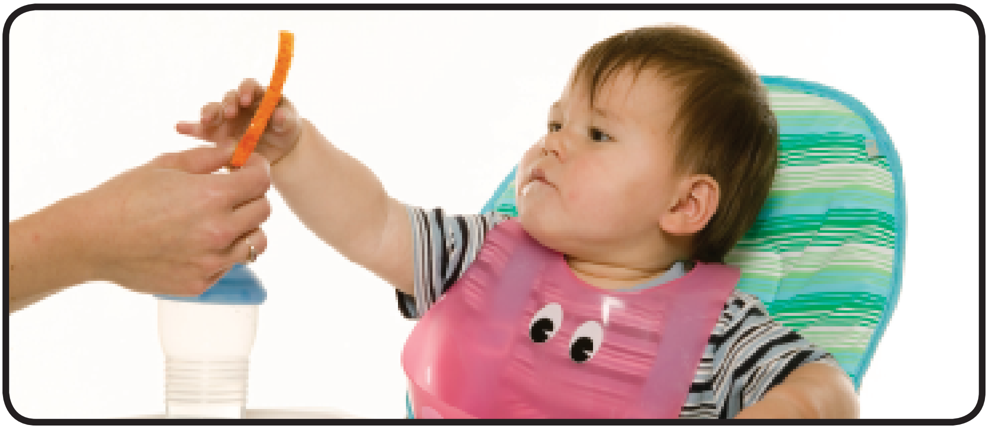

They're sweet enough already!

If you give your baby sweet things they are likely to get a taste for it. In fact giving babies food and drink with added sugar or honey can cause serious problems. Here's why sugar is not so sweet:

- Sugar can rot their tiny teeth. Drinks with added sugar are really bad news for baby teeth
- Giving babies sugary things makes them more likely to pester for more
- Too much sugar means excess energy, which can give babies a higher risk of becoming overweight or obese. This can lead to type 2 diabetes, heart disease and some cancers in later life.

Offer water with meals instead of sugary drinks, and choose healthier snacks like fruit or toast, pitta or chapati fingers.

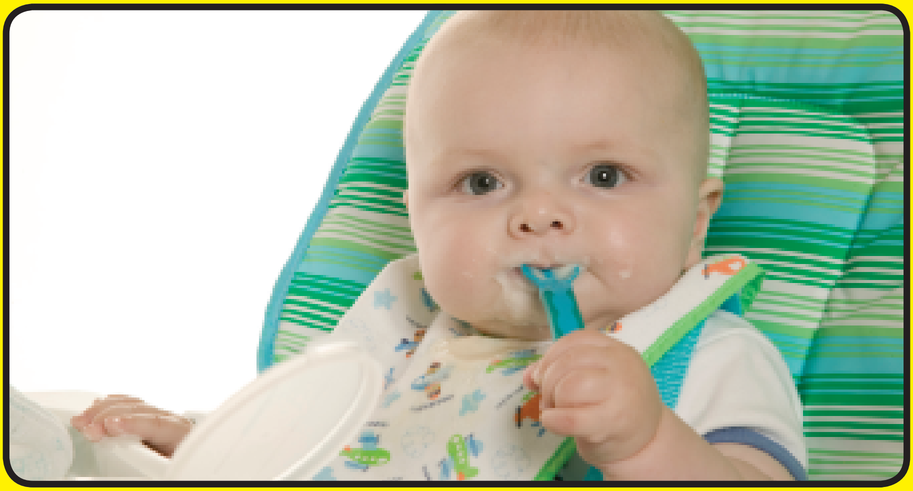

And don't forget...

Babies love to kick their legs, crawl and toddle about as much as possible. This is essential for their development. So make some safe space clear of furniture for them to move around.

56447 COI Solid Food.indd 14 11/02/2011 12:53

other useful information

Allergies

While variety in your baby's diet is really important, there is a chance they may be allergic to certain foods. That's why it's important to introduce cow's milk, eggs, wheat, gluten, nuts, peanuts, peanut products, seeds, fish, and shellfish one at a time and not before 6 months. There is no evidence that waiting until your child is older will prevent them from developing a food allergy. Give them to your baby in very small amounts and watch carefully for any signs or symptoms of an allergic reaction (see our 'things to look out for' box below).

If your baby already has a known allergy such as a diagnosed food allergy or eczema, or you have a family history of food allergies, eczema, asthma or hay-fever, you may need to be particularly careful when introducing peanuts and peanut products, so talk to your GP or health visitor first. Remember peanuts, like all nuts, should be crushed or ground.

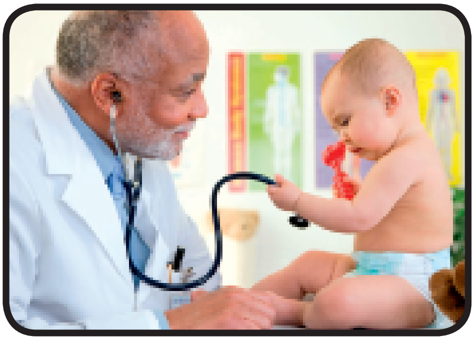

You can find out more at
www.food.gov.uk/safereating/allergyintol/peanutspregnancy

Things to look out for:

- breathing difficulties
- runny nose
- stomach upset
- skin reaction: blotchy, raised red rashes, itchy patches and swelling around the mouth or sore, red and itchy eyes.

If your child shows any of these symptoms, talk to your GP for advice or contact NHS Direct at www.nhsdirect.nhs.uk or call 0845 4647

If you think your is baby is suffering a severe allergic reaction, always call 999 and ask for a paramedic.

56447 COI Solid Food.indd 15 11/02/2011 12:53

Safety

Remember, you should always stay with your baby while they are eating in case they choke. If your child does choke and cannot breathe properly, shout for help, pick up your child and support their chest and chin with one hand. Give 5 sharp blows between the shoulder blades with the heel of the other hand to help dislodge the object.

Learn how to deal with choking by visiting www.nhs.uk/planners/birthtofive/pages/howtohelpchoking.aspx or, even better, do a first aid course.

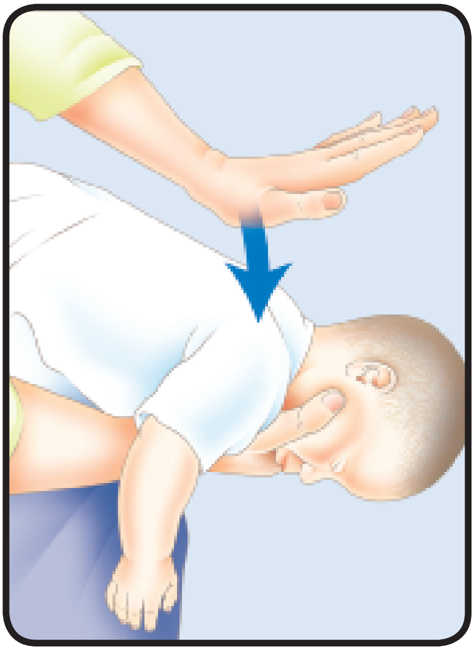

Hygiene

Ensure your baby is not at risk from germs as a result of food preparation and serving.

- Always wash and dry your hands well before preparing your baby's food
- Check that your baby's hands are clean before feeding
- Keep surfaces clean and prevent pets from coming near food or surfaces where food is prepared or eaten
- Keep chopping boards thoroughly clean
- Keep raw meat and eggs covered and away from other foods in the fridge including cooked or ready to eat meats. Raw meat should ideally be kept in a sealed container at the bottom of the fridge so it can't touch or drip onto other foods.

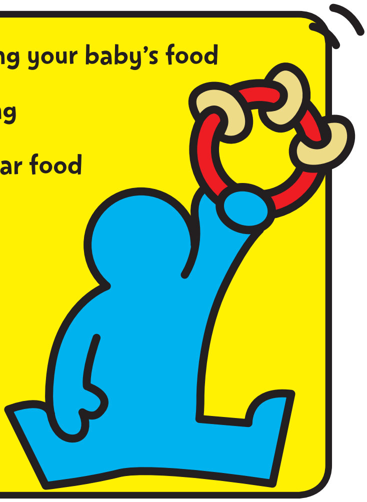

56447 COI Solid Food.indd 16 11/02/2011 12:53

- Thoroughly wash and dry all bowls and spoons for feeding in hot soapy water
- You can make up a batch of baby food and freeze it in individual portions, but make sure you only serve each portion once. Don't re-use food that hasn't been eaten as this might make your baby ill
- Cooked food should not be reheated more than once
- Cook all food thoroughly and cool it as quickly as possible to a lukewarm temperature before giving it to your baby
- Wash and peel fruit and vegetables, such as apples and carrots
- Avoid raw eggs and raw shellfish.

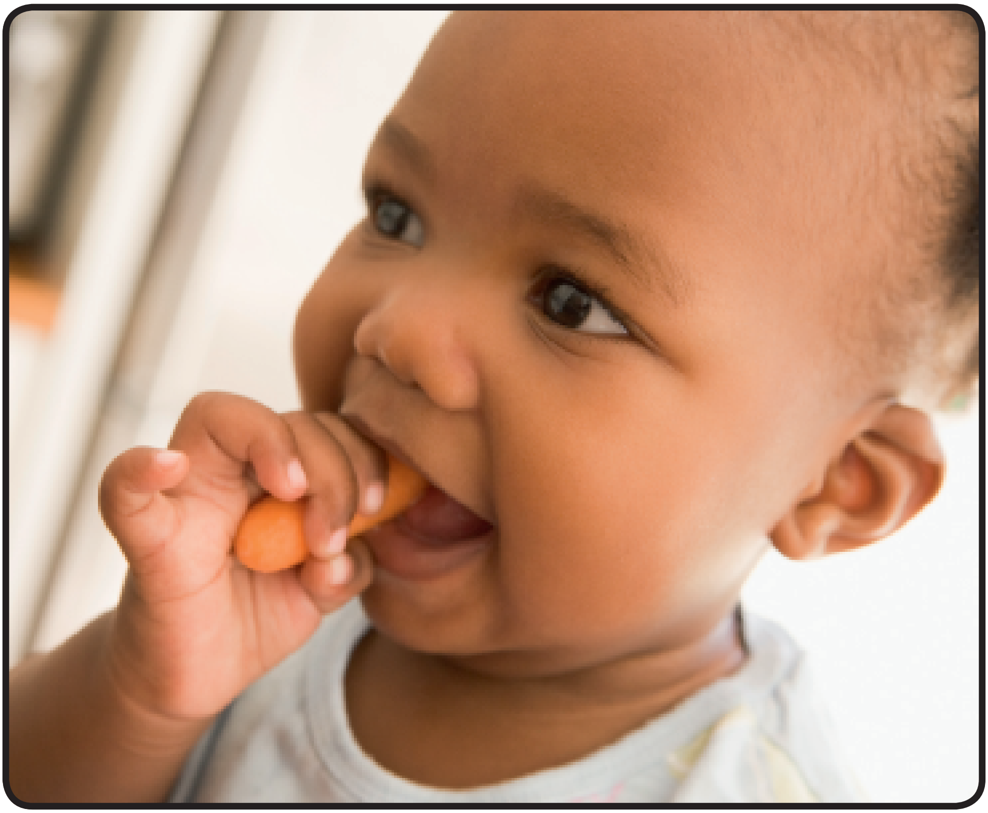

56447 COI Solid Food.indd 17 11/02/2011 12:53

Storing and reheating food

- Cool food as quickly as possible (ideally within one to two hours) and place it in the fridge or freezer. Food placed in the fridge should be eaten within two days
- Make sure that frozen food is thoroughly defrosted before reheating. The safest way to do this is in the fridge overnight or using the defrost setting on a microwave
- Reheat food thoroughly so it is very hot all the way through. Allow it to cool before offering it to your baby
- To cool food quickly, place the food in an airtight container and hold it under cold running water, stirring the contents from time to time to allow it to cool all the way through.

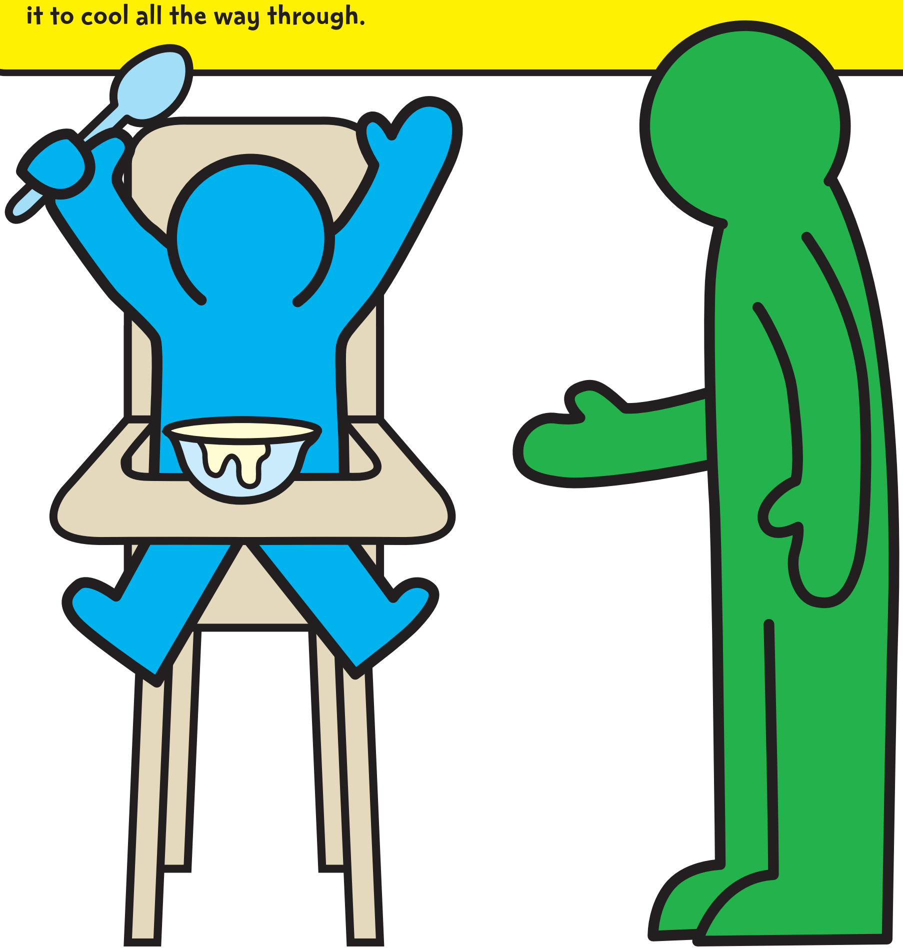

56447 COI Solid Food.indd 18 11/02/2011 12:53

Vitamins

If you are pregnant or breastfeeding your baby, you need to take a daily supplement of 10mcg of vitamin D.

If your baby is 6 months or older and is having less than 500ml (1 pint) of infant formula per day, give them vitamin drops containing vitamins A, C and D.

Continue to give your child vitamin drops until they are 5.

Be careful not to give your baby two supplements at the same time.

For example, don't give them cod liver oil as well as vitamin drops – one on its own is strong enough.

Your health visitor or midwife can give you information on vitamins. Remember, vitamin drops for your baby are free if you qualify for Healthy Start.

If you have children under four or are pregnant and on benefits, or if you're pregnant and under 18, you could qualify for Healthy Start. With Healthy Start, you can get free vouchers every week which you can spend on milk, fruit, vegetables and infant formula milk. You can also get free vitamins. Ask your health visitor or midwife for details or visit www.healthystart.nhs.uk

Even if you don't qualify for Healthy Start you might still be able to buy Healthy Start children's vitamin drops or Healthy Start vitamins for women. Ask a member of the health visiting or midwifery team for details.

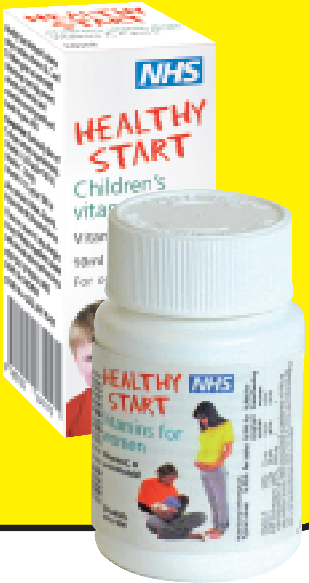

We’re here to help you

If you need to talk to someone about anything in this booklet, or anything to do with your baby’s health:

- Talk to your midwife or a member of your health visiting team
- Visit your local Sure Start Children’s Centre
- Call Start4Life on 0300 123 1021* or visit www.nhs.uk/start4life
- Call the National Breastfeeding Helpline on 0300 100 0212* or visit www.nationalbreastfeedinghelpline.org.uk
- Ask your midwife or health visitor for a copy of the ‘Birth to Five’ booklet or view online
- Read the information on introducing solid food at www.nhs.uk/breastfeeding

If you are a parent or carer of a baby aged 5 - 8 months then find out how you’re doing at www.nhs.uk/babylifecheck and get some answers to those tricky questions we all have.

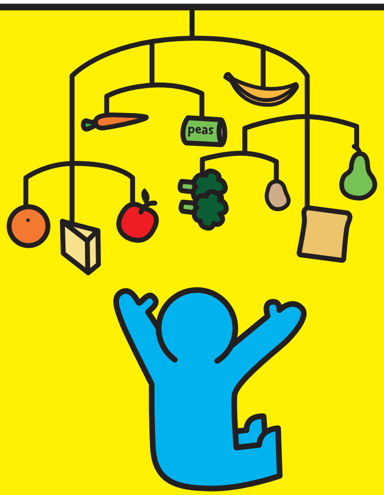

50% recycled
When you have finished with this leaflet please recycle it

*Calls to 03 numbers should cost no more than geographic 01 or 02 UK-wide calls and may be part of inclusive minutes subject to your provider and your call package. The National Breastfeeding Helpline is open from 9.30am to 9.30pm. The Start4Life lines are open from 9am to 8pm. Both are open 7 days a week.

© Crown copyright 2011. C4L175 (Feb 2011) Brochure 750K Feb 2011 (How).
Produced by COI for the Department of Health.

start 4 life
A good start for a healthier life

56447 COI Solid Food.indd 20 11/02/2011 12:53
