# Supplementary material for: Using intervention mapping to develop a culturally appropriate intervention to prevent childhood obesity: the HAPPY (Healthy and Active Parenting Programme for Early Years) study
Source: Int J Behav Nutr Phys Act. 2013 Dec 28;10:142. doi: 10.1186/1479-5868-10-142 (PMC3895739; doi:10.1186/1479-5868-10-142)
Supplement: Additional file 4 — a. MASTER HAPPY Antenatal Manual FINAL VERSION. b. MASTER HAPPY Postnatal Manual FINAL VERSION. c. HAPPY manual resources. [file 1479-5868-10-142-S4.zip › 1523029418990916_add4c7.zip!/Responding to concerns about feeding baby handout.doc]

**Responding to Concerns about Feeding Baby**

1. **What if my baby doesn’t want to feed for very long or doesn’t finish the bottle?**

**Feeding responsively**. Parents should be encouraged to trust their baby’s appetite. When a breastfed baby turns away from the breast or a bottle-fed baby stops feeding, parents should not be tempted to encourage the baby to have more as this will result in over-feeding. This is the baby’s way of saying that he has had enough. Additionally if baby is overfed regularly then he may not be able to recognise the feeling of fullness and will therefore be at risk of gaining too much weight.

2. **What if my baby feeds more on one day and less on the next?**

**Feeding to appetite** – your baby’s appetite will vary from day to day and as baby grows. For breast-feeders, as baby grows s/he may suddenly appear to want to feed more often or for longer: this is the way the baby is able to stimulate an increase in mums milk supply at each feed to meet their needs and it will settle down in a day or two – people often take this as a sign that mum’s milk is no longer enough, lose confidence and start to add bottles at this point –so less feeding and less milk is produced – **reassure** the group that this is the way it works and encourage them to go with the flow!

3. **What if members of my family are telling me to change the feed; start adding rusks to the bottle; start giving solids?**

Do not feel pressured by others to change type of feed, add anything to bottles, start weaning early: discuss all changes with your Health visitor. Do not start weaning until your baby is ready which should be around 6 months and not before 17 weeks.

4. **What if I need to feed my baby when I am out of the house?**

It is possible to feed your baby when out and about but you will need to make some preparations.

If you are breastfeeding, you can breastfeed your baby in public but will want to think about where you do this, as some members of the public and even family can feel offended if they are not used to it.

Consider your clothing – a feeding bra, loose fitting clothing and a scarf or cloth to cover yourself can make it easier.

Many public places welcome breastfeeding with the Breastfeeding Welcome Here initiative.

If you are bottle feeding, you will need to take the necessary equipment with you to make up bottles whilst out and about such as hot water in a flask, sterilised bottles, infant formula etc.

5. **What if my baby seems unhappy and unsettled in the evenings when I’m trying to make a meal for the family?**

Babies can often be more unsettled in the evening. There are a number of reasons why a baby can be unhappy so check to see if they are tired, bored, uncomfortable etc before assuming they are hungry and thirsty, but they may want feeding.

Try to get some help from other family members at this busy time of day to help entertain baby or help with household tasks.

Try to plan your evening meal earlier in the day to save time in the evening.

**If you have any specific concerns about your baby and it’s feeding, check them out with your Health Visitor as needed.**
