# Supplementary material for: Using intervention mapping to develop a culturally appropriate intervention to prevent childhood obesity: the HAPPY (Healthy and Active Parenting Programme for Early Years) study
Source: Int J Behav Nutr Phys Act. 2013 Dec 28;10:142. doi: 10.1186/1479-5868-10-142 (PMC3895739; doi:10.1186/1479-5868-10-142)
Supplement: Additional file 4 — a. MASTER HAPPY Antenatal Manual FINAL VERSION. b. MASTER HAPPY Postnatal Manual FINAL VERSION. c. HAPPY manual resources. [file 1479-5868-10-142-S4.zip › 1523029418990916_add4c7.zip!/Tempted by takeaways handout.doc]

**TEMPTED BY TAKEWAYS?**

Match the takeaway (1 serving) to its calorie content by drawing a line to link them.

(Answers overleaf).

½ lb / 8oz burger

+ roll	750 kcals

+ medium French fries

Fish shop fish

+ (medium) chips	1050 kcals

Chicken Tikka Masala	1280 kcals

+ Naan bread

Large Doner kebab	980 kcals

Chinese spare ribs + egg	1050 kcals

Fried rice ( ½ tray of each)

Drinks and Sweet extras

350ml can of regular fizzy drinks	139 kcal	Jam doughnut	239 kcal

500ml bottle Coke	200 kcal	100g bar chocolate	530 kcal

440ml can Lager	190 kcal

175ml glass wine	120 kcal

If you are having a takeaway because you need a meal fast –think about some alternatives you could have

E.g. ½ large tin of beans on toast (2 slices)	only 350kcals

Quick homemade sweet & sour chicken with rice	only 300kcals

see recipe overleaf – for more quick, cheap and healthy meals, look at <http://www.nhs.uk/Change4Life> and select the “recipe finder” section

If you are having a takeaway as a planned meal or weekend treat –watching the size of your portions and choosing carefully can help reduce the fat and calorie content. For more information

<http://www.nhs.uk/Livewell/men1839/Pages/Dodgytakeaways.aspx>

**Sweet and Sour Chicken Recipe**

Swap the chicken for a selection of vegetables or Quorn for a meat free version.

Serves: 4 adults

Preparation time: 20 mins

Cooking time: 30 mins

**Ingredients**:

150g easy cook white or brown rice

227g pineapple pieces in natural juice

1 tbsp cornflour

2 tsp tomato puree

1 tsp reduced salt soy sauce

2 tsp vegetable oil

300g skinless uncooked chicken (thigh or breast) cut into chunks

1 medium onion thinly sliced

1 peppers, any colour deseeded and cut into chunks

3 celery sticks sliced

2 tomatoes sliced into wedges

1 pinch ground black pepper

**What to do**:

1. Put the brown rice on to cook in plenty of boiling water for 30 minutes, or until tender. If using white rice, remember it needs to cook for just 12 minutes.
2. Meanwhile, drain the juice from the pineapple into a small bowl or jug. Cut the pineapple rings into chunks. Add the cornflour to the juice and stir until smooth, then mix in the tomato puree and soy sauce. Set to one side.
3. 10 minutes before the rice is cooked, heat the oil in a wok or very large frying pan. Add the chicken and stir-fry over a high heat for 3-4 minutes.
4. Add the onion, pepper and celery to the wok or frying pan. Stir-fry for another 3-4 minutes, then add the tomato wedges and pineapple.
5. Stir the pineapple juice mixture, then add it to the wok, stirring until hot and thickened. Season with pepper, and serve with the drained rice.

**Why not try adding extra or different vegetables? Try broccoli florets, red onion, sliced green beans and carrot strips for a change.**

Per portion (i.e. ¼ recipe)

295kcals

From Change 4 Life supermeals menu planner and recipe finder

[www.nhs.uk/Change4Life/Pages/supermeals-menu-planner-recipe-finder.aspx](http://www.nhs.uk/Change4Life/Pages/supermeals-menu-planner-recipe-finder.aspx)

**Quiz Answers**

80z burger, roll and medium French fries	980 kcals

Fish shop fish and medium chips	1280 kcals

Chicken tikka masala and naan bread	1050 kcals

Large doner kebab	1050 kcals

Chinese spare ribs and egg fried rice (1/2 tray of each)	750kcals
