# Supplementary material for: Using intervention mapping to develop a culturally appropriate intervention to prevent childhood obesity: the HAPPY (Healthy and Active Parenting Programme for Early Years) study
Source: Int J Behav Nutr Phys Act. 2013 Dec 28;10:142. doi: 10.1186/1479-5868-10-142 (PMC3895739; doi:10.1186/1479-5868-10-142)
Supplement: Additional file 4 — a. MASTER HAPPY Antenatal Manual FINAL VERSION. b. MASTER HAPPY Postnatal Manual FINAL VERSION. c. HAPPY manual resources. [file 1479-5868-10-142-S4.zip › 1523029418990916_add4c8.zip!/Being sedentary questions and answers handout.doc]

**Being Sedentary: Activities and Questions**

To be sedentary you must meet 3 basic criteria

1. You must be using very little energy
2. You must be sitting or lying down
3. You must be awake

We will look at each activity/question below in turn and answer them as a group as we go along. Start with activity 1. Once you are all finished with activity 1 as a group we will talk through the answer before we move onto the next question/activity.

**PART 1: Sedentary activities and me**

1. With a partner look through the word pool below and circle all of the activities that you think would be classified as “sedentary” activities

doing the washing-up	working on the computer	**RIDING A BIKE**

***Sleeping at night***	Watching TV

walking to the shop	**driving**

playing on the computer	**sunbathing on holiday	swimming**

reading a book or magazine	**washing the car**

**watching a movie at the cinema**	snoozing in the day-time

sitting having a cup of tea and a chat

**2.**	Which of the following do you think are the risks for **you** spending too much time doing “sedentary activities”?

Overweight or obese	□

Hayfever	□

Heart problems	□

Loss of muscle strength	□

Diabetes	□

Itchy feet	□

High blood pressure	□

3. Put a tick next to all of the “**sedentary activities**” from the pool above that you do every day.
4. Government recommendations for adults are to minimise the amount of time you spend doing sedentary activities. Put a star next to those activities in the word pool above that you **could** cut down on.

**PART 2: TV viewing and my baby**

Watching the television is one activity that some infants and toddlers spend many hours a day doing. New research has looked at some of the risks for very young children (aged 0 – 4 years old) of spending a long time watching TV:

**1.** Tick the risk that you think too much TV watching might have for your baby as they grow up:

Overweight or obese	□

Hayfever	□

Poor language development	□

Low attention span	□

Itchy feet	□

Low self esteem	□

Weak bones	□

Hair that knots easily	□

Poor movement skills	□

**2.** How long do you think an infant less than 2 years old should watch TV for each day? (Tick one box)

No TV	□

Less than 1 hour a day	□

1-2 hours a day	□

2-3 hours a day	□

3 or more hour a day	□
3. Remember that you baby will copy what you do. If you spend a lot of time watching TV or doing sedentary activities your baby will too. As a group discuss ways that you could lower the amount of time you spend doing the “sedentary activities” that you have stared in the word pool. Write down below any of the changes that you have discussed that you would like to and be willing to make. Who will you ask to support you?

…………………………………………………………………………………………………………………………………………………………………………………………………………………………………………………………………………………………………………………………………………………………………………………………………………

**Being Sedentary: ANSWERS**

1. With a partner look through the word pool below and circle all of the activities that you think would be classified as “sedentary” activities

doing the washing-up	working on the computer	**RIDING A BIKE**

***Sleeping at night***	Watching TV

walking to the shop	**driving**

playing on the computer	**sunbathing on holiday	swimming**

reading a book or magazine	**washing the car**

**watching a movie at the cinema**	having a nap in the day-time

sitting having a cup of tea and a chat

**2.**	Which of the following do you think are the risks for **you** spending too much time doing “sedentary activities”?

Overweight or obese	□ TICK

Hayfever	□

Heart problems	□ TICK

Loss of muscle strength	□ TICK

Diabetes	□ TICK

Itchy feet	□

High blood pressure	□ TICK

5. Put a tick next to all of the “**sedentary activities**” from the pool above that you do every day.
6. Government recommendations for adults are to minimise the amount of time you spend doing sedentary activities. Put a star next to those activities in the word pool above that you **could** cut down on.

**PART 2: TV viewing and my baby**

Watching the television is one activity that some infants and toddlers spend many hours a day doing. New research has looked at some of the risks for very young children (aged 0 – 4 years old) of spending a long time watching TV:

**1.** Tick the risk that you think too much TV watching might have for your baby as they grow up:

Overweight or obese	□ TICK

Hayfever	□

Poor language development	□ TICK

Low attention span	□ TICK

Itchy feet	□

Low self esteem	□ TICK

Weak bones	□ TICK

Hair that knots easily	□

Poor movement skills	□ TICK

A1. ***This is very new research, as research continues we will probably find that there are many other risks for children who spend too much time watching TV or doing other “sedentary activities”.***

**2.** How long do you think an infant less than 2 years old should watch TV for each day? (Tick one box)

No TV	□

restrict TV viewing	□	TICK

Less than 1 hour a day	□

1-2 hours a day	□

2-3 hours a day	□

4 or more hour a day	□

There is not a clear answer yet as to how much time an infant should spend watching TV. In the UK our government guidelines are to restrict the amount of time infants and toddlers spend watching TV. As more research is done this may change.

In Australia their guidelines are that TV/computer time is not appropriate for children younger than 2 years old. For children who are 2 – 5 years old, TV/computer time should be less than 1 hour per day.

In America they recommend no TV/computer usage for children younger than 2 years old and less than 2 hours a day for all children older than 2 years old.

In Canada they say TV viewing should be 1-to-2 hours per day for all children.

**The bottom line is to limit the amount of time your infant watches TV. It’s best to switch the TV off during meal times and when your baby is playing so that they are not distracted from what they are doing.**
