# Supplementary material for: Using intervention mapping to develop a culturally appropriate intervention to prevent childhood obesity: the HAPPY (Healthy and Active Parenting Programme for Early Years) study
Source: Int J Behav Nutr Phys Act. 2013 Dec 28;10:142. doi: 10.1186/1479-5868-10-142 (PMC3895739; doi:10.1186/1479-5868-10-142)
Supplement: Additional file 4 — a. MASTER HAPPY Antenatal Manual FINAL VERSION. b. MASTER HAPPY Postnatal Manual FINAL VERSION. c. HAPPY manual resources. [file 1479-5868-10-142-S4.zip › 1523029418990916_add4c8.zip!/How to start weaning a practical guide handout .docx]

**HOW TO START WEANING – PRACTICAL GUIDE**

**HOW TO START WEANING AT ABOUT 6 MONTHS**

- **Equipment** – small shallow plastic spoon and plastic feeding bowl, bib and plastic mat or newspaper to protect the floor as weaning can be messy.
- **Seating** – infants need to be sitting up to avoid choking. A highchair is best so that your infant is sat at your level and will also be better able to explore foods.
- **Quantity** – one or two teaspoons should be offered at first.
- **Consistency** – smooth puree mixed with usual breast/formula milk. For infants starting weaning at 6 months, this stage may be very short or even bypassed. At this age infants will need to quickly move to a thicker texture with soft lumps.
- **Foods** – Start with baby rice or other non-wheat cereal such as sago, maize, cornmeal or millet, smooth vegetables or fruit. Once used to these, other foods should be included: pureed/mashed meat, fish, lentils or pulses; full fat dairy products e.g. yoghurt or custard; other cereal foods and soft finger foods. Please refer to hand-out ‘Summary of recommendations for weaning at different stages from 6-12 months’ for examples.
- **New foods** do not need to be introduced one at a time unless there is an immediate family history of allergy.
- **Encourage the use of family foods** but avoid adding salt and sugar to the food that will be offered to the baby.
- **Breastfeeding on demand or at least 500**-**600mls infant formula/day** should continue until one year of age.
- **Give children’s vitamin drops containing vitamins A, C and D.** Even on a healthy balanced diet infants and children under 5 years may not get enough of these vitamins especially vitamin D. Healthy Start vitamin drops are recommended for all babies and children and are available free of charge or at low cost.

**DRINKS**

- A lidded, free flowing cup can be introduced from 6 months. Water should be offered so that infants learn to expect this rather than sweet drinks.
- Well diluted pure fruit juice (half and half) can be given at meal times and will increase iron absorption from vegetable sources of iron and vegetarian meals.
- Cow’s milk should not be offered as a drink until after 1 year of age as it is a very poor source of iron. It can be used in cooking e.g. custards and sauces.

**PROGRESSING THROUGH WEANING STAGES**

- **Eating family foods** with the family is important as it helps infants to develop self-feeding and social skills and promotes good eating habits from an early age. Seeing the family enjoying a variety of foods will encourage infants to try new foods and mean they are less likely to be fussy eaters as they get older.
- Infants need to be introduced to a wide range of new tastes and textures – some foods may need to be tried 6-8 times before they are accepted!
- Parents should be guided by infant’s appetite and gradually increase the amount offered, progressing to different foods being offered over 3 meal times.
- **Feeding from a bottle** should be discouraged by one year instead us a feeding cup
- **Self-feeding skills** – these can be encouraged from the beginning of weaning by offering finger foods and giving infants their own spoon to try, whilst you continue to help with feeding.
- **Amounts and types of foods for the first year** – please refer to hand-out ‘Summary of recommendations for weaning at different stages from 6-12 months’ for examples.

**PROMOTING HEALTHY EATING**

**RECOMMENDATIONS FOR HEALTHY EATING BY 1 YEAR OF AGE**

- Eat three set meals and two-three healthy snacks.
- Meals should be presented at timely intervals
- Meals should be provided at set family mealtimes
- The infants food should aim to resemble healthy family meals
- The practice of constant eating - “grazing” should be discouraged
- Snacks offered in between meals should include foods such as fruit, vegetables, bread and plain biscuits.
- Avoid giving sugary drinks
- Give water and milk (aim for no more than a pint of milk/day)
- Avoid adding extra sugar to drinks and food
- Use only a small amount of butter and margarine
- Avoid cooking methods such as frying and roasting that use
  a lot of fat
- Avoid giving too many fatty foods such as crisps, chocolate
- Offer your infant a wide variety of fresh fruit and vegetables at mealtimes and as snacks

**FOODS TO AVOID**

**Salt** – infants’ kidneys are not fully developed at six months of age and high amounts of salt/salty foods can be harmful. Salt should not be added to foods. Processed foods such as stock cubes, gravy mixes, packet soups, instant mashed potato, sauces and ready meals should be avoided.

**Sugar** – encourages a sweet tooth and can lead to dental decay and obesity

**Honey** – can contain botulinum bacteria and needs to be avoided until after the age of one year.

**Nuts** – children under 5 should avoid whole nuts due to a risk of choking. Peanut butter, pastes and ground peanuts can be included after 6 months of age. *Parents should talk to their GP or health visitor before giving peanuts for the first time if infant has a diagnosis of eczema/food allergy or a history of allergies in the family.*

**Some Fish** -avoid shark, swordfish or marlin due to mercury content. Avoid raw shellfish due to the risk of food poisoning. Fish especially oily fish e.g. mackerel, salmon, fresh tuna are a valuable source of protein and essential fatty acids in the diet and can be introduced during weaning.

**High fibre/low fat diets** - are not recommended as these diets are very bulky and can make it difficult for infants and young children to eat enough to meet their energy requirements for growth. Fibre (eg added bran) can reduce the nutrients absorbed. Full fat dairy products (ie using full fat milk in food preparation, yogurts and cheese) are the best usual choice for babies and children under 2 years.
