# Supplementary material for: Using intervention mapping to develop a culturally appropriate intervention to prevent childhood obesity: the HAPPY (Healthy and Active Parenting Programme for Early Years) study
Source: Int J Behav Nutr Phys Act. 2013 Dec 28;10:142. doi: 10.1186/1479-5868-10-142 (PMC3895739; doi:10.1186/1479-5868-10-142)
Supplement: Additional file 4 — a. MASTER HAPPY Antenatal Manual FINAL VERSION. b. MASTER HAPPY Postnatal Manual FINAL VERSION. c. HAPPY manual resources. [file 1479-5868-10-142-S4.zip › 1523029418990916_add4c8.zip!/Menu planner for weaning stage 1 handout The Dairy Council.pdf]

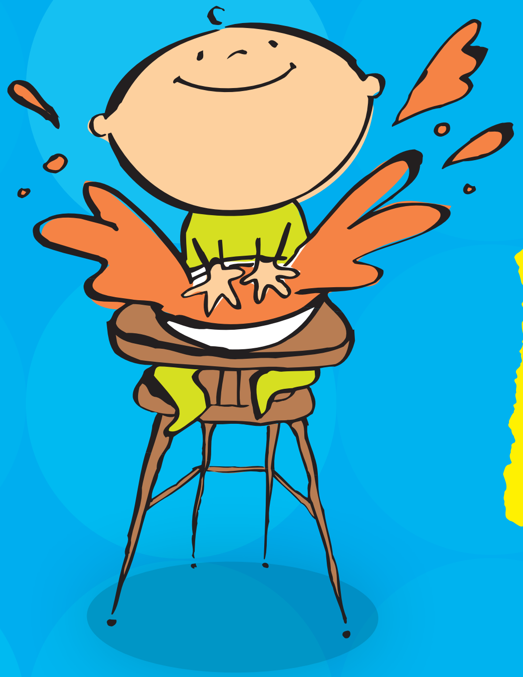

Menu planner for weaning

STAGE 1

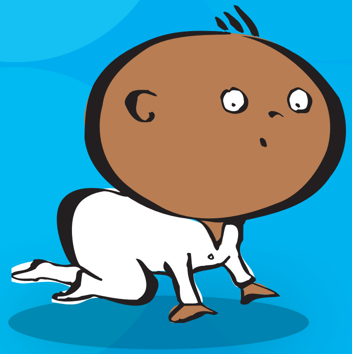

	Start with:	Then move on to:	Finally increase to:
Number of meals a day	1	2	3
Breakfast	Breast or infant formula	Baby rice mixed with breast or infant formula	Baby rice mixed with breast or infant formula
Midday	Breast or infant formula	Breast or infant formula	Purée or mash* of meat or pulses and vegetables
Early evening	Baby rice mixed with breast or infant formula	Fruit or vegetable purée or mash* and breast or infant formula	Fruit purée or mash* and breast or infant formula
Late evening	Breast or infant formula	Breast or infant formula	Breast or infant formula

* Mashed food with soft lumps should be introduced from around six months

The Dairy Council

www.milk.co.uk

© The Dairy Council 2011
