# Supplementary material for: Using intervention mapping to develop a culturally appropriate intervention to prevent childhood obesity: the HAPPY (Healthy and Active Parenting Programme for Early Years) study
Source: Int J Behav Nutr Phys Act. 2013 Dec 28;10:142. doi: 10.1186/1479-5868-10-142 (PMC3895739; doi:10.1186/1479-5868-10-142)
Supplement: Additional file 4 — a. MASTER HAPPY Antenatal Manual FINAL VERSION. b. MASTER HAPPY Postnatal Manual FINAL VERSION. c. HAPPY manual resources. [file 1479-5868-10-142-S4.zip › 1523029418990916_add4c8.zip!/Menu planner for weaning stage 2 handout The Dairy Council.pdf]

Menu planner for weaning

STAGE 2

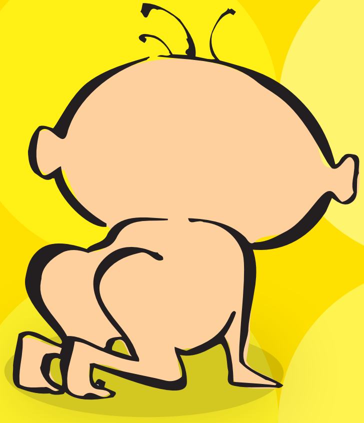

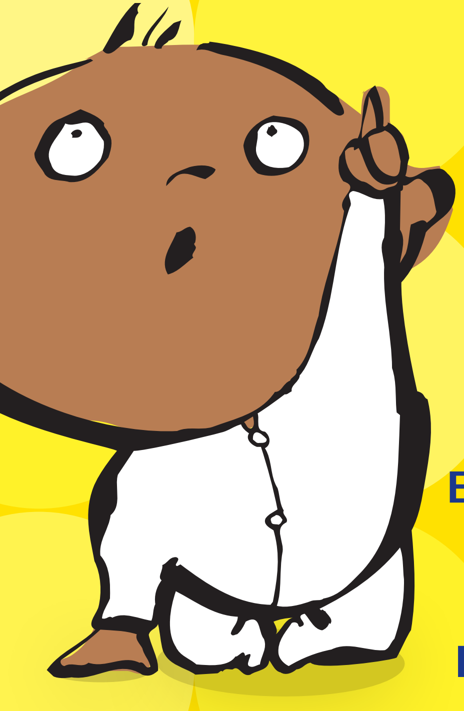

	Day 1	Day 2	Day 3
**Breakfast**	Baby rice or porridge*<br>Small cup of orange juice (diluted)	Wheat-based cereal*<br>Small cup of orange juice (diluted)	Mashed banana and toast fingers<br>Small cup of orange juice (diluted)
**Midday**	Spaghetti bolognese (mashed)<br>Plain fromage frais with stewed apple<br>Breast or infant formula	Minced lamb and vegetable casserole with mashed potato<br>Soft, ripe peeled pear or peach (finger food)<br>Breast or infant formula	Lentil and vegetable bake<br>Semolina with strawberries<br>Breast or infant formula
**Early evening**	Fish, broccoli and mashed potato<br>Cooked carrot (finger food)<br>Breast or infant formula	Scrambled egg with toast, chapatti or pitta bread<br>Breast or infant formula	Macaroni cheese<br>Breast or infant formula
**Late evening**	Breast or infant formula	Breast or infant formula	Breast or infant formula

* Mixed with either breast, whole cows' milk or infant formula

The Dairy Council
www.milk.co.uk

© The Dairy Council 2011 For details on the information source please contact The Dairy Council Tel 020 7467 2629 info@dairycouncil.org.uk Last reviewed: 06/10/2011 Next review due: 06/10/2012
