# Supplementary material for: Using intervention mapping to develop a culturally appropriate intervention to prevent childhood obesity: the HAPPY (Healthy and Active Parenting Programme for Early Years) study
Source: Int J Behav Nutr Phys Act. 2013 Dec 28;10:142. doi: 10.1186/1479-5868-10-142 (PMC3895739; doi:10.1186/1479-5868-10-142)
Supplement: Additional file 4 — a. MASTER HAPPY Antenatal Manual FINAL VERSION. b. MASTER HAPPY Postnatal Manual FINAL VERSION. c. HAPPY manual resources. [file 1479-5868-10-142-S4.zip › 1523029418990916_add4c9.zip!/Making meals healthier handout.doc]

**MAKING MEALS HEALTHIER – CUT THE FAT BUT NOT THE TASTE**

**Meat and poultry**

- Buy lean meat and trim away excess fat; discard the skin on poultry
- Use a smaller amount of meat in stews, casseroles, soups
- Use pulses, peas or lentils to extend or replace meat
- Use methods of cooking which don’t need added fat such as grilling, roasting with a roasting bag, browning in a non-stick pan
- Use plenty of herbs and seasonings in place of fat and salt
- Don’t add salt to food for babies.

**Minced beef**

Mince is usually high in fat. Try to buy ‘extra lean’ mince or turkey mince instead.

**How to reduce fat**:

1. Place the mince in a dry saucepan over a low heat on the cooker.
2. Stir all the time, to prevent it burning, until it browns.
3. Throw away all the fat which melts out from the meat, alternatively leave overnight in the fridge so that it solidifies, then skim it off.
4. Then add onions, vegetables, garlic, tinned tomatoes, herbs and spices etc. (if the vegetables are added before stage 3 they will absorb most of the fat!).

**Curries and fried food**

**Reduce fat** – use less oil or ghee for cooking. Measure oil in a spoon and use not more than half a tablespoon per person. Before eating, skim off any oil or fat that rises to the surface.

**Eat more vegetables** – try adding extra vegetables to meat or chicken curries. Replace some meat curries with dahls but make sure you use less oil or ghee.

‘Fried’ foods (e.g. kebabs, burgers, fish fingers). Most foods which you would normally fry can be **grilled or baked** instead without adding any fat or oil.

**Adding flavour – without the fat**

**Herbs & spices**

Herbs and spices may be used in cooking to bring out the flavour of the following foods. This is useful when you are reducing fat in cooking.

**Stews and casseroles**

**Reduce fat** – choose a small quantity of lean meat or skinned chicken or turkey. Cut all visible fat from raw meat. Brown meat or vegetables in a non-stick frying pan before cooking.

**Eat more vegetables** – add more vegetables and include potatoes with their skins on.

**Reduce salt** – try mixed herbs and black pepper instead.

**White Sauce**

**Reduce fat** – use skimmed or semi-skimmed milk. Make by blending milk with cornflour rather than the ‘roux’ flour and fat method.

**Cheese Sauce**

**Reduce fat** – make a white sauce as above and add a low fat cheese or half the amount of a strong flavour cheese.

**Reduce salt** – add mustard and black pepper to bring out the flavour of the sauce.

**Vegetables**

Cook vegetables in a steamer or microwave for the best taste and to preserve vitamins and minerals. Otherwise boil in a covered saucepan with the minimum of water. Serve without butter or margarine. Fresh and frozen vegetables are equally as good.

**Sausage and Mash**

**Reduce fat** – use low fat sausages and grill. Do not add fat to mash. Use low fat milk.

**Eat more vegetables** – serve with vegetables e.g. peas, sweetcorn, carrots, broccoli.

**Chilli Con Carne**

**Reduce fat** – cook mince as overleaf, add cooked red kidney beans with vegetables.

**Eat more vegetables** – add extra vegetables, e.g. mushrooms. Red kidney beans are high in fibre.

**Reduce salt** – if using tinned kidney beans wash well before adding to the mince.

**Spaghetti Bolognese**

**Reduce fat** – cook mince as overleaf.

**Eat more vegetables** – add extra vegetables including mushrooms, courgettes and carrots. Serve with side salad.

**Shepherd’s Pie**

**Reduce fat** – cook mince as overleaf, add onions. Do not add fat to the mash. Use low fat milk.

**Eat more vegetables** – add extra vegetables e.g. celery and carrot to the mince before cooking in the oven. You could also add cooked beans or lentils.
