# Supplementary material for: Using intervention mapping to develop a culturally appropriate intervention to prevent childhood obesity: the HAPPY (Healthy and Active Parenting Programme for Early Years) study
Source: Int J Behav Nutr Phys Act. 2013 Dec 28;10:142. doi: 10.1186/1479-5868-10-142 (PMC3895739; doi:10.1186/1479-5868-10-142)
Supplement: Additional file 4 — a. MASTER HAPPY Antenatal Manual FINAL VERSION. b. MASTER HAPPY Postnatal Manual FINAL VERSION. c. HAPPY manual resources. [file 1479-5868-10-142-S4.zip › 1523029418990916_add4c9.zip!/Meal planner for weaning handout.doc]

**Meal Planner for Weaning**

|  | **Day 1** | **Day 2** | **Day3** |
| --- | --- | --- | --- |
| **Breakfast** |  |  |  |
| **Mid-morning** |  |  |  |
| **Midday** |  |  |  |
| **Afternoon** |  |  |  |
| **Early evening** |  |  |  |
| **Bedtime** |  |  |  |
