# Supplementary material for: Using intervention mapping to develop a culturally appropriate intervention to prevent childhood obesity: the HAPPY (Healthy and Active Parenting Programme for Early Years) study
Source: Int J Behav Nutr Phys Act. 2013 Dec 28;10:142. doi: 10.1186/1479-5868-10-142 (PMC3895739; doi:10.1186/1479-5868-10-142)
Supplement: Additional file 4 — a. MASTER HAPPY Antenatal Manual FINAL VERSION. b. MASTER HAPPY Postnatal Manual FINAL VERSION. c. HAPPY manual resources. [file 1479-5868-10-142-S4.zip › 1523029418990916_add4c9.zip!/Now I can crawl handout Child Accident Prevention Trust.pdf]

If you have a new baby,
ask for our booklet called
I'm only a baby but...

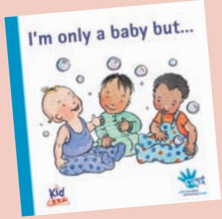

child accident
prevention trust

Child Accident Prevention Trust
Canterbury Court (1.09)
1-3 Brixton Road
London SW9 6DE
tel 020 7608 3828
fax 020 7608 3674
email safe@capt.org.uk
web www.capt.org.uk

registered charity number 1053549

illustration: Fran Jordan
design and artwork: www.wave.coop

© Child Accident Prevention Trust 2006
BKT006 reprinted February 2009

This publication is not meant as a substitute for advice on particular issues and is written in general terms. If you have any concerns or if you have particular questions you should seek specific advice before taking any action based on the information that this publication contains.

SAMPLE

Now I can crawl I can...

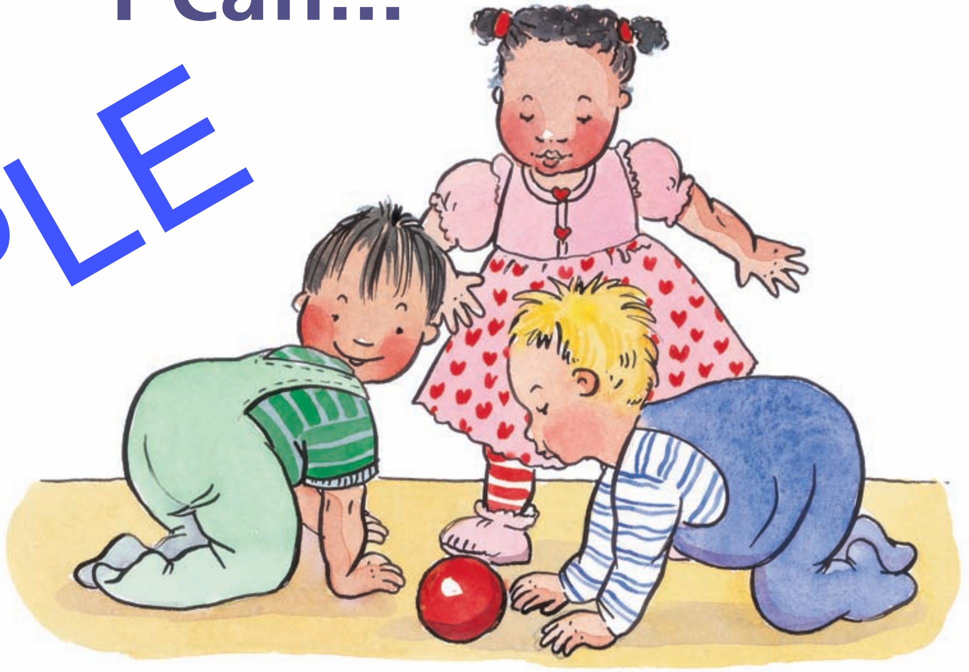

child accident
prevention trust

I can help you care for me...

- **Hello!** If you've got a baby like me, this booklet is for you.
- **Why?** Because it's here to help you keep me safe now that I can crawl or stand.
- It tells you about some of the ways I can get hurt in an accident.
- And it tells you how to try and make sure that I don't.

SAMPLE

The accidents I've chosen are the ones I'm most likely to get hurt in.

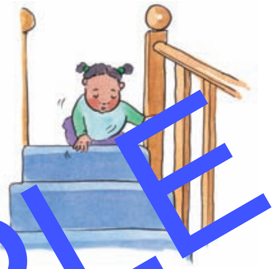

Falling

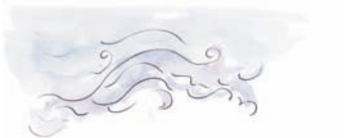

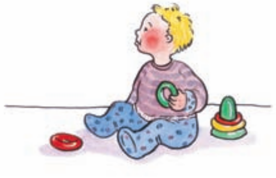

Fire in my home

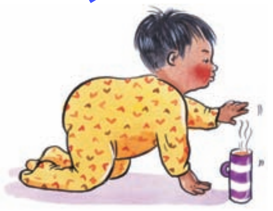

Burns to my skin

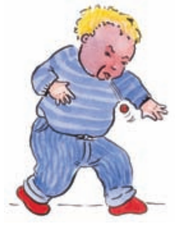

Getting into trouble

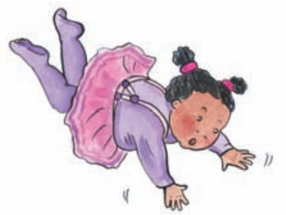

Riding in the car

Turn over to find out more...

Now I can crawl or stand...

- I'm still very little but I'm learning new things every day.
- In fact, I like to surprise you about what I can do.
- But remember! Surprises can end up with me getting hurt in an accident.

Watch out!

- Every year, lots of babies like me are rushed to hospital because we've been hurt in accidents.

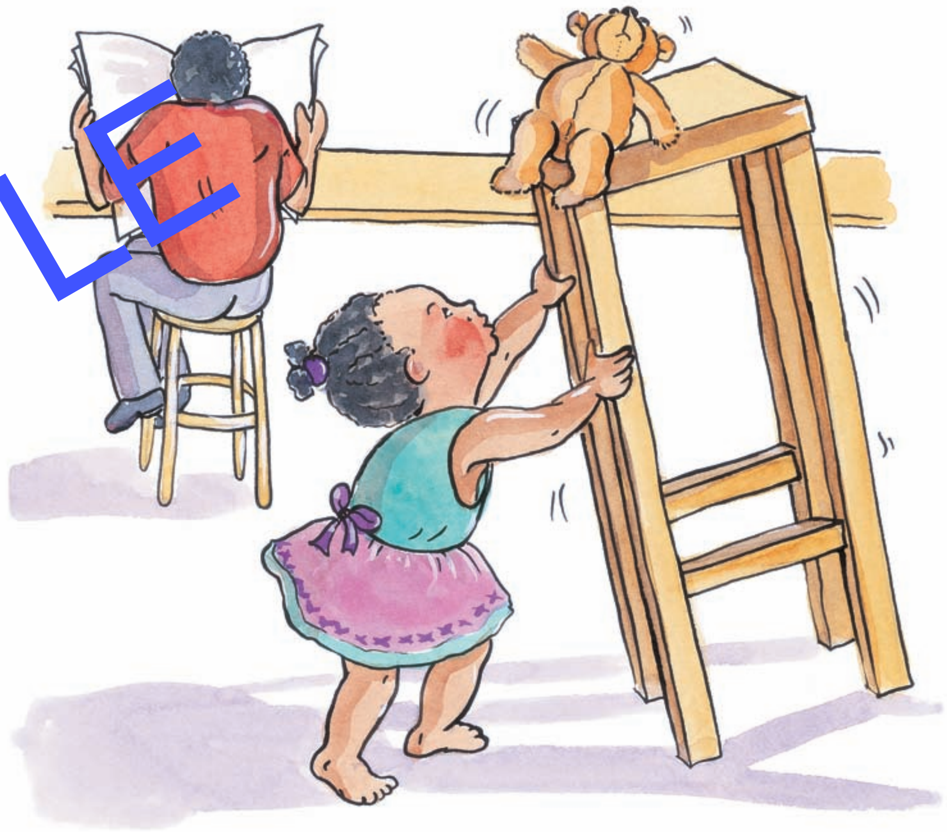

SAMPLE

You need to keep me safe...

- I know you're really busy looking after me.
  And you may be really tired too.
- So I'm making it as quick and easy as I can.
- Look at a page with a sad face.
  Sad faces show you how babies like me can get hurt.
- Now look at a page with a happy face.
  That's how to keep me safe.

SAMPLE

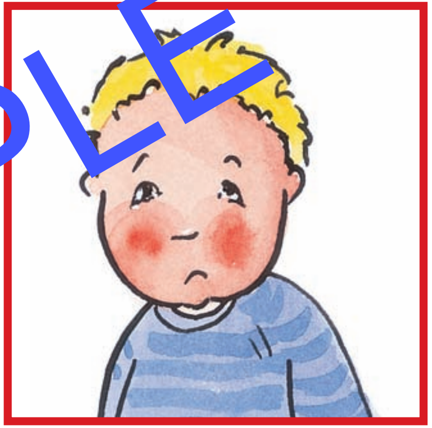

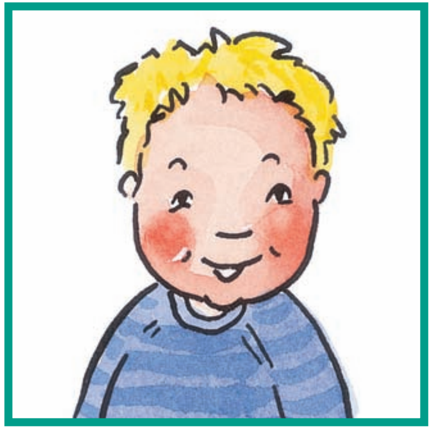

I could get badly hurt if I fall...

Now I can crawl and maybe stand up,
I may even be able to climb onto things.

Also, I'm big enough to stand up
in my highchair.

And to stand up in my pushchair.

Make sure I don't have a fall...

SAMPLE

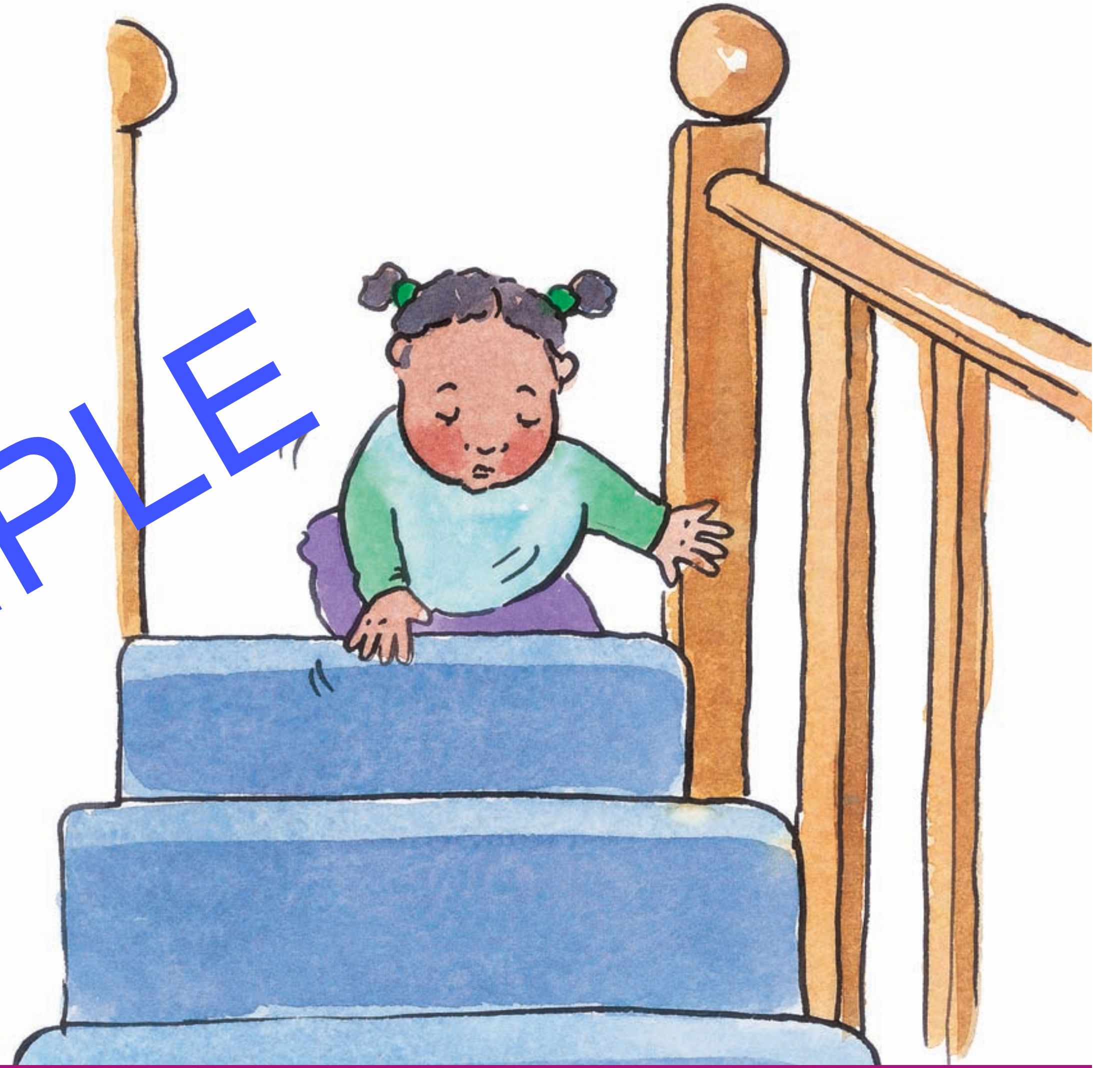

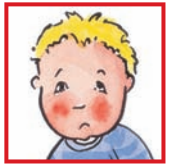

I could roll off a bed or a table while you're changing my nappy.

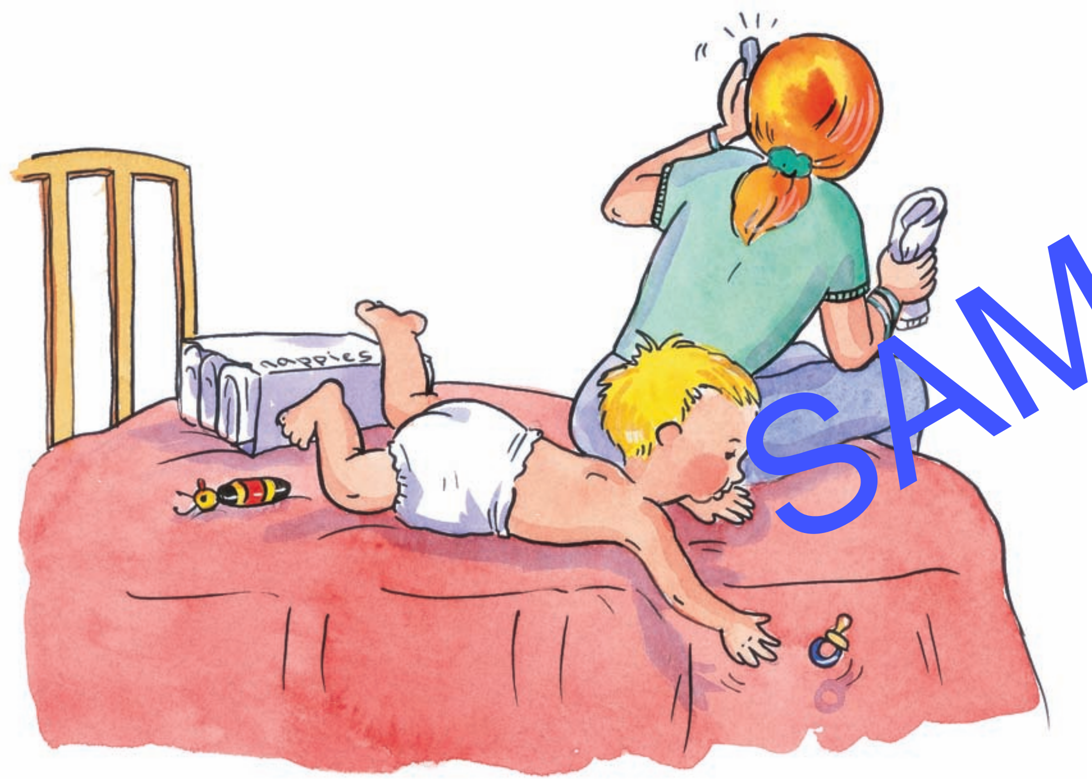

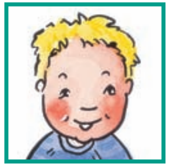

Change my nappy on the floor.

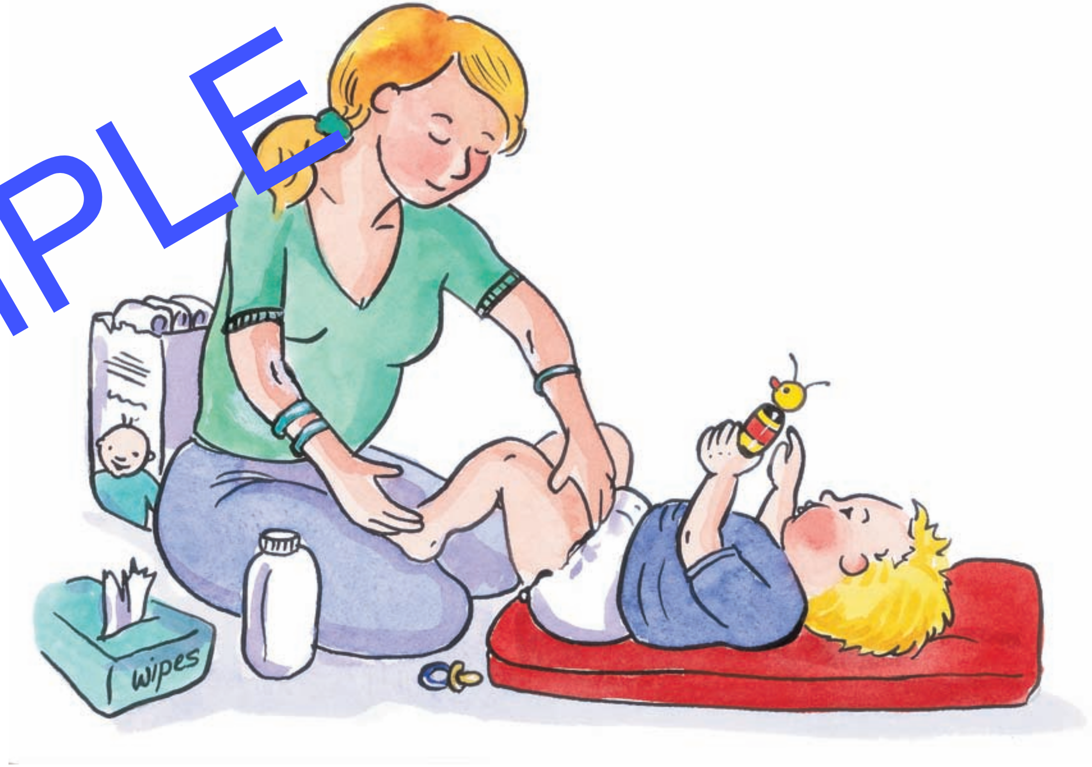

SAMPLE

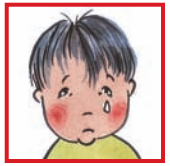

I might try to go up or down the stairs on my own.

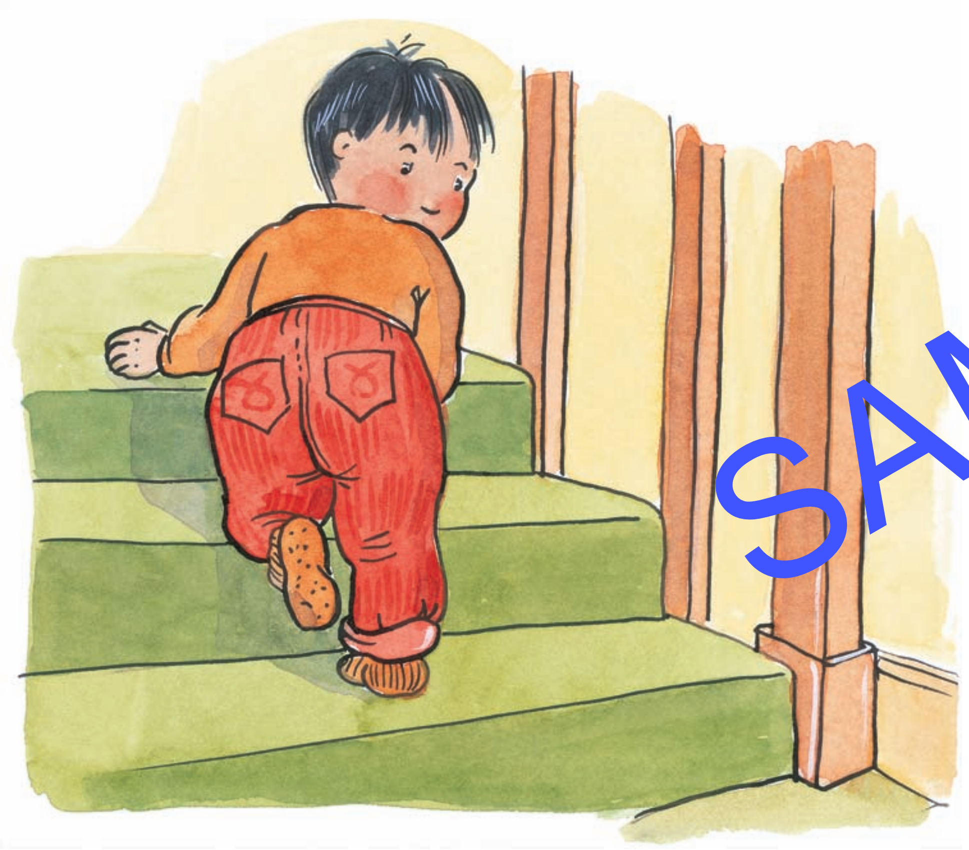

SAMPLE

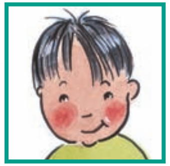

Use safety gates at the top and bottom of the stairs.

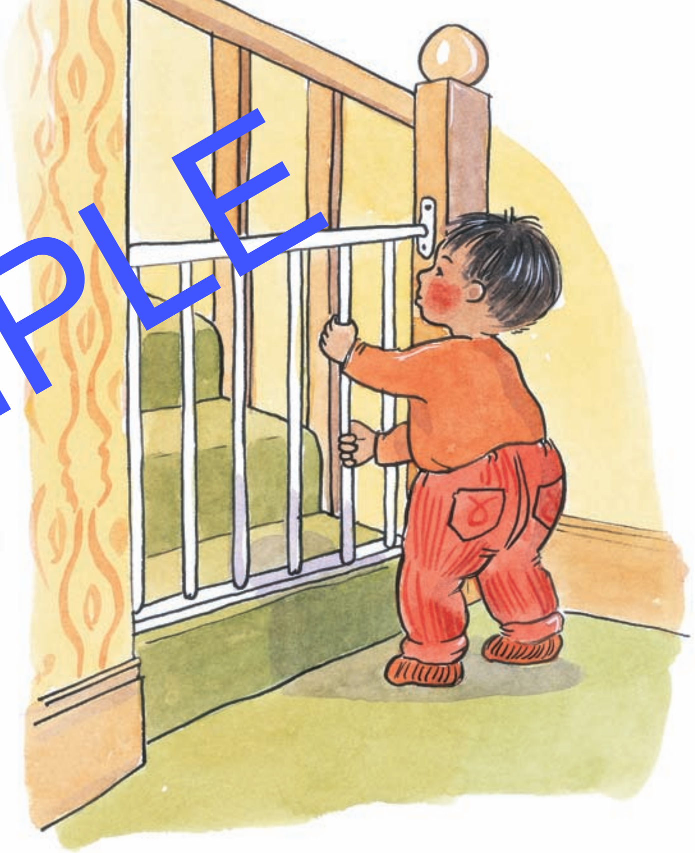

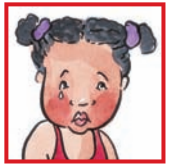

You might forget to strap me into my highchair.

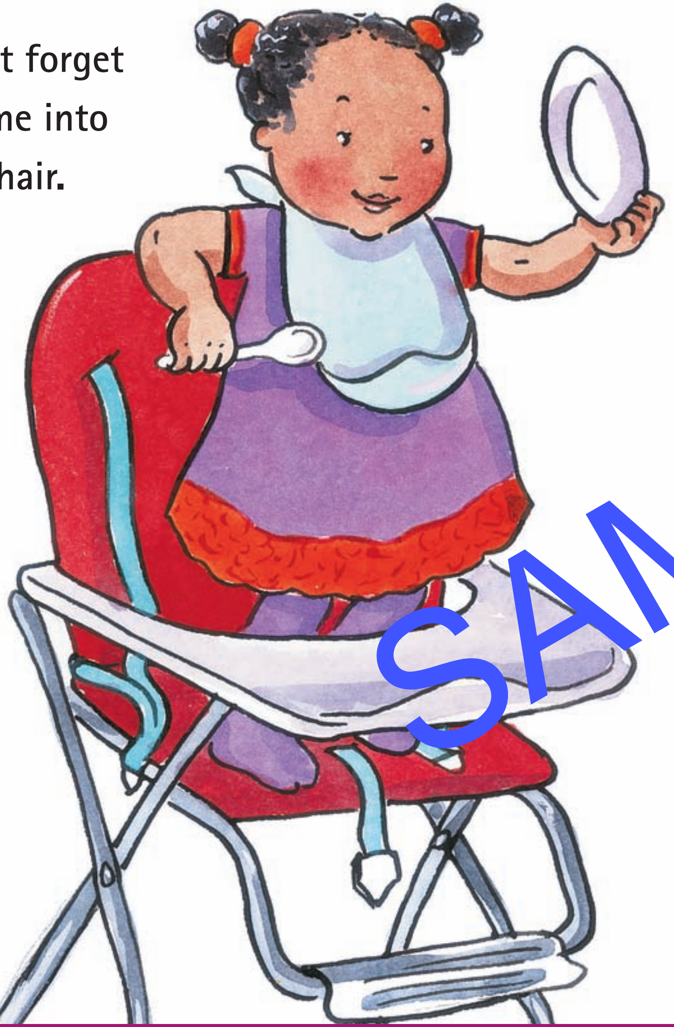

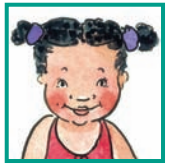

Use a harness with five straps like the one in the picture when I'm sitting in my highchair.

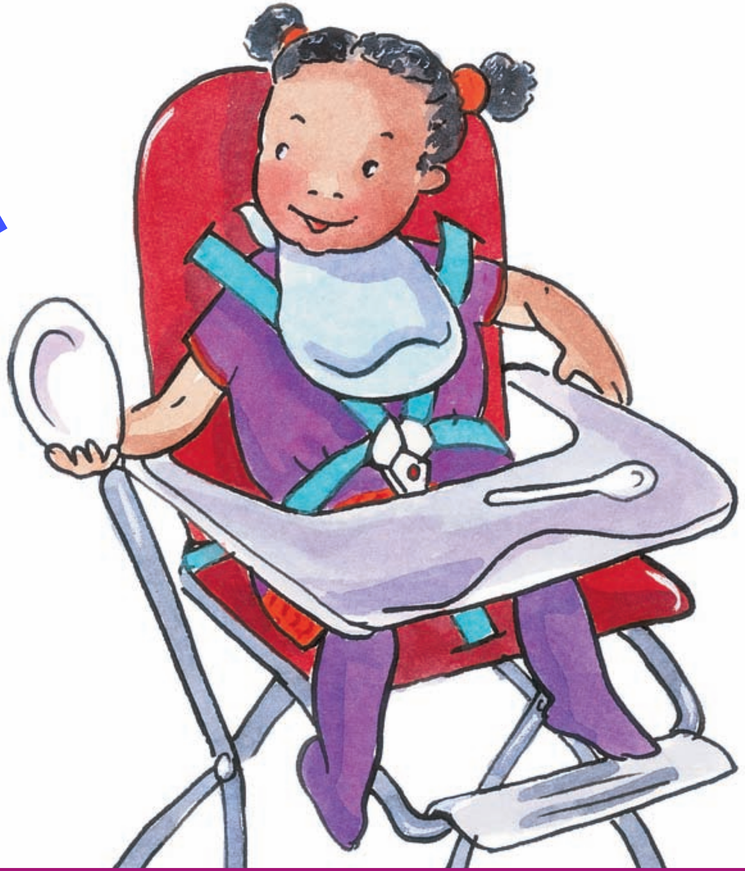

SAMPLE

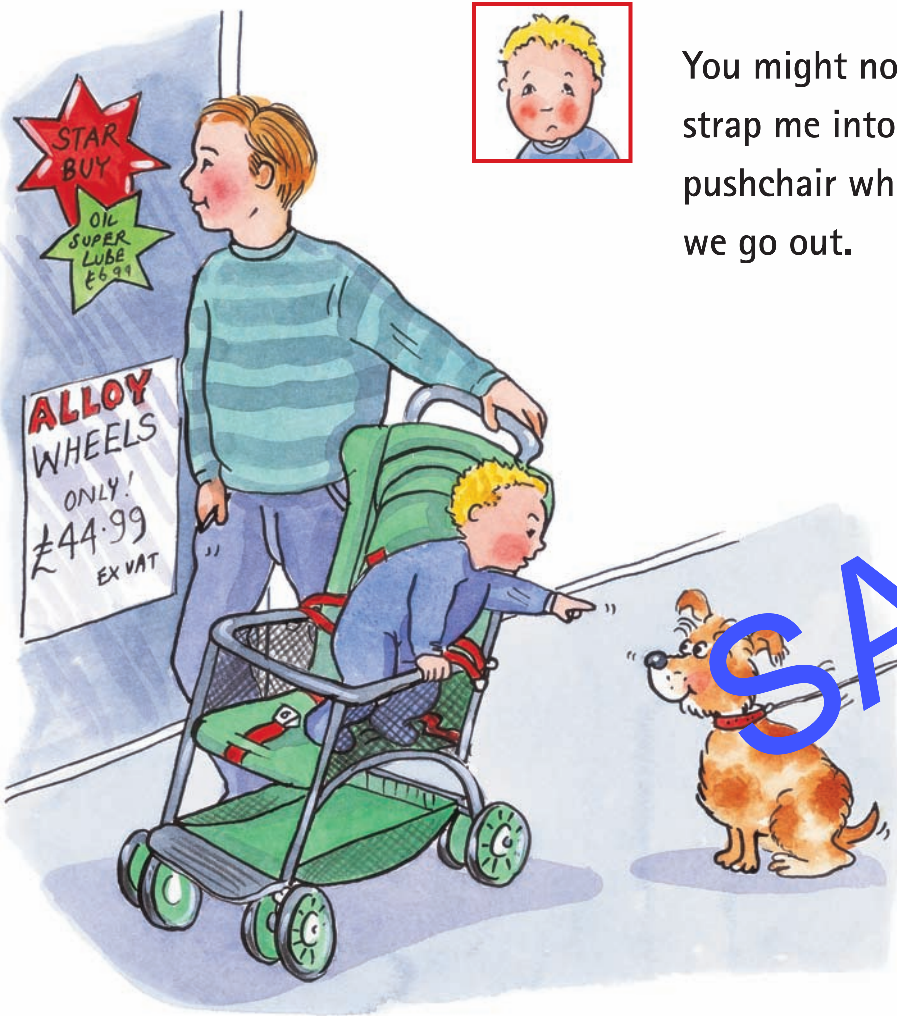

You might not strap me into my pushchair when we go out.

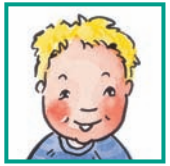

Use a harness with five straps like the one in the picture when you take me out in my pushchair.

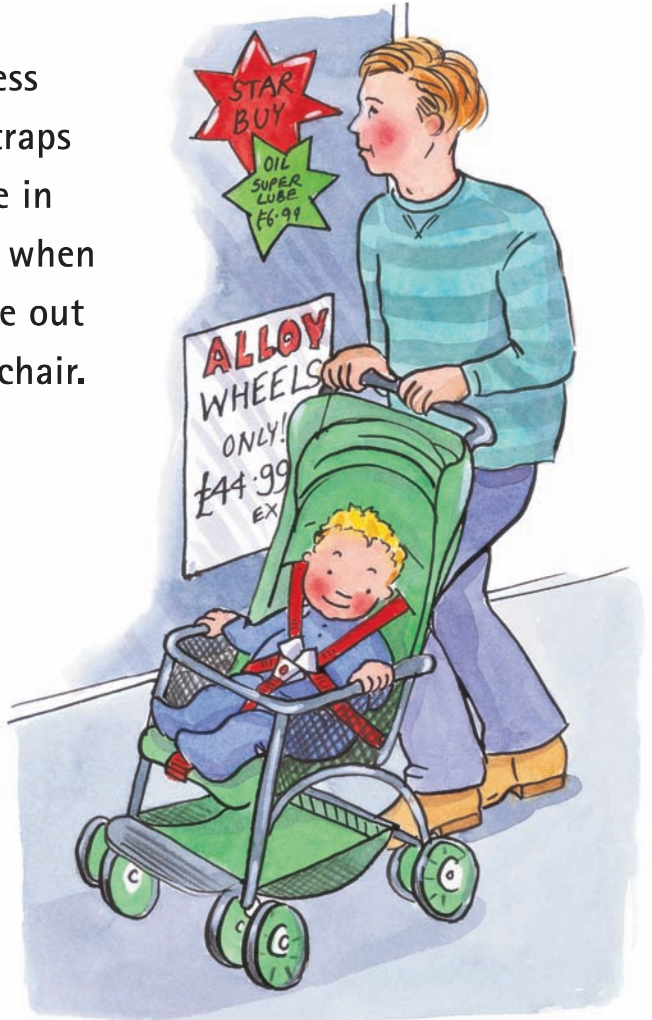

SAMPLE

If my home catches fire, I could get hurt or die...

If there's a fire in my home, I might breathe in the smoke.

Or the flames might stop the fire brigade from getting to me in time.

Make sure my home doesn't catch fire...

SAMPLE

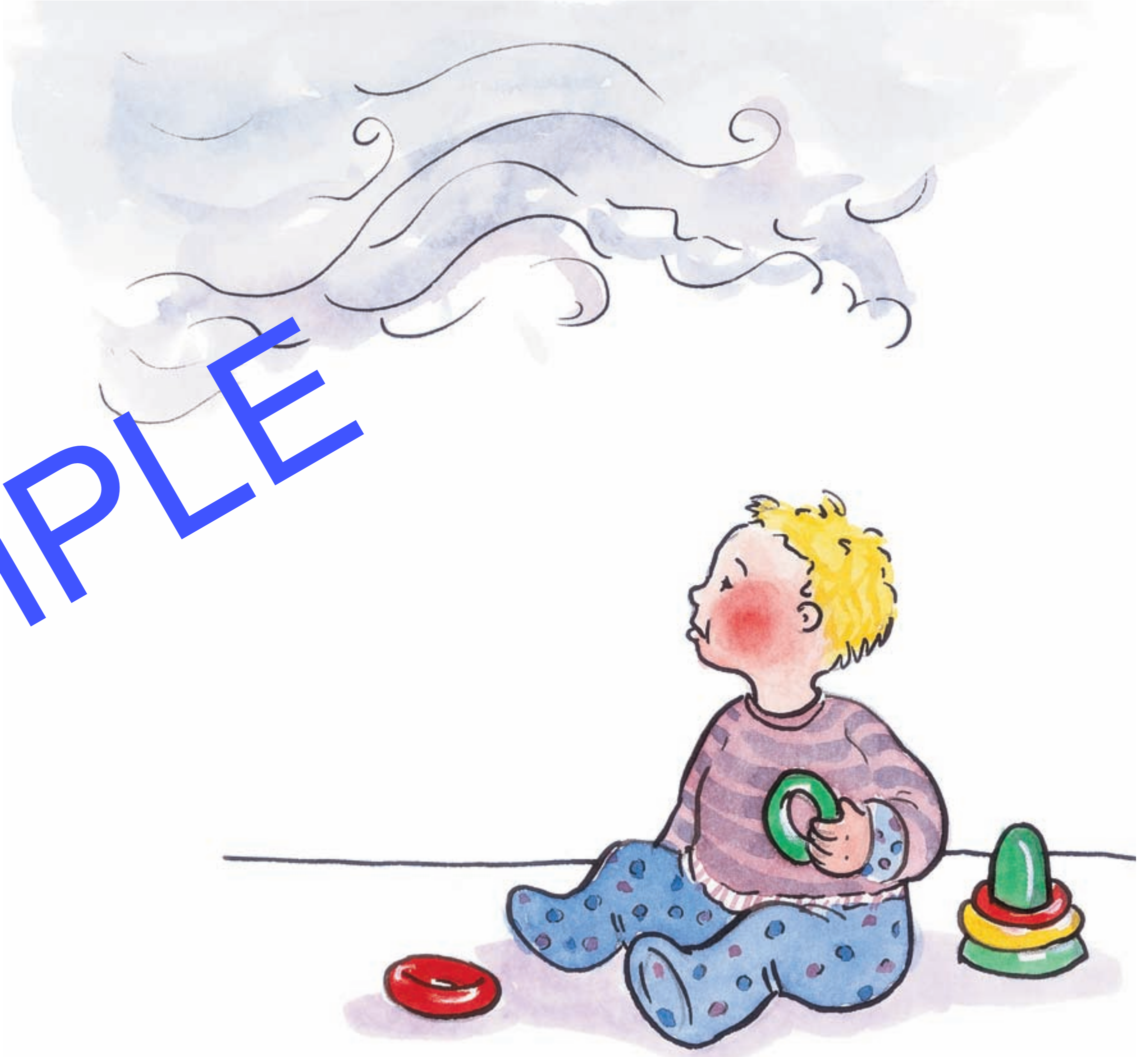

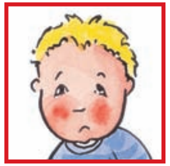

A hot pan could catch fire if you turn your back on it.

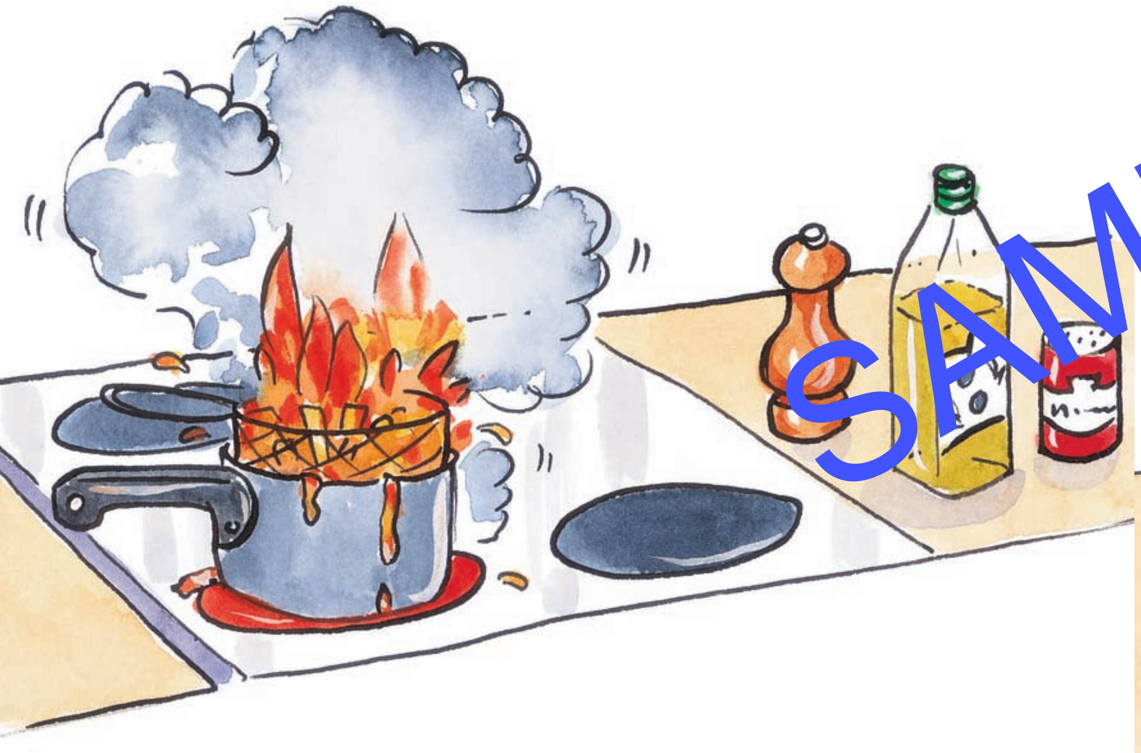

SAMPLE

Keep a close eye on hot pans like chip pans, frying pans and woks.

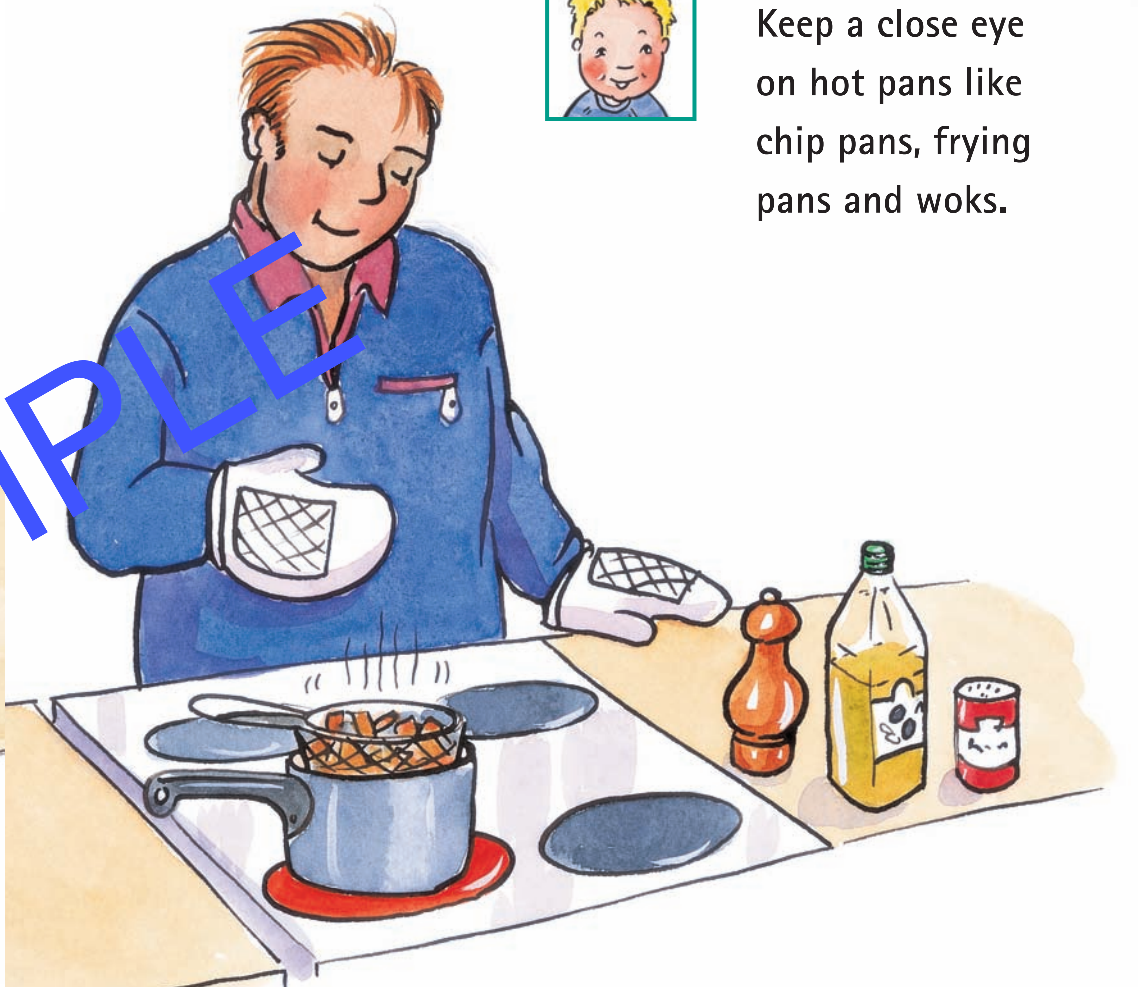

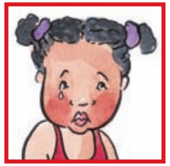

You might fall asleep in the armchair
and drop your cigarette.

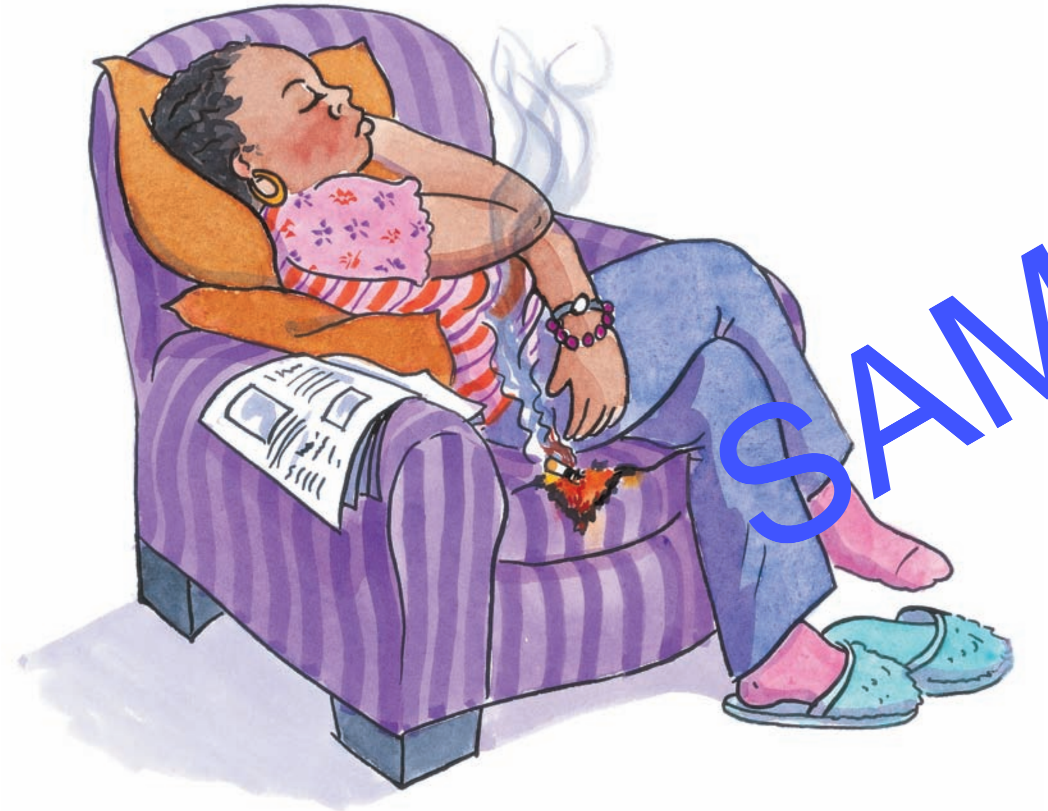

SAMPLE

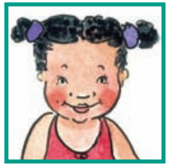

Stub your cigarette right out
if you feel sleepy.

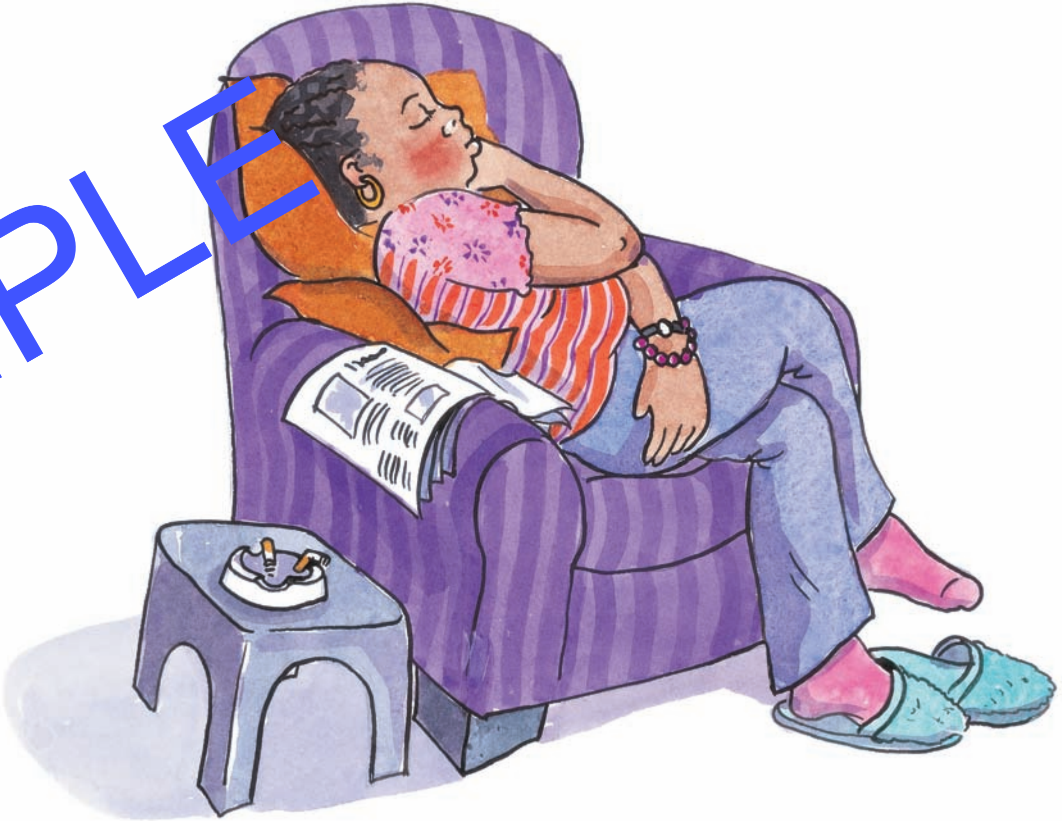

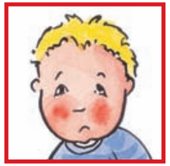

You might not smell smoke
until it's too late.

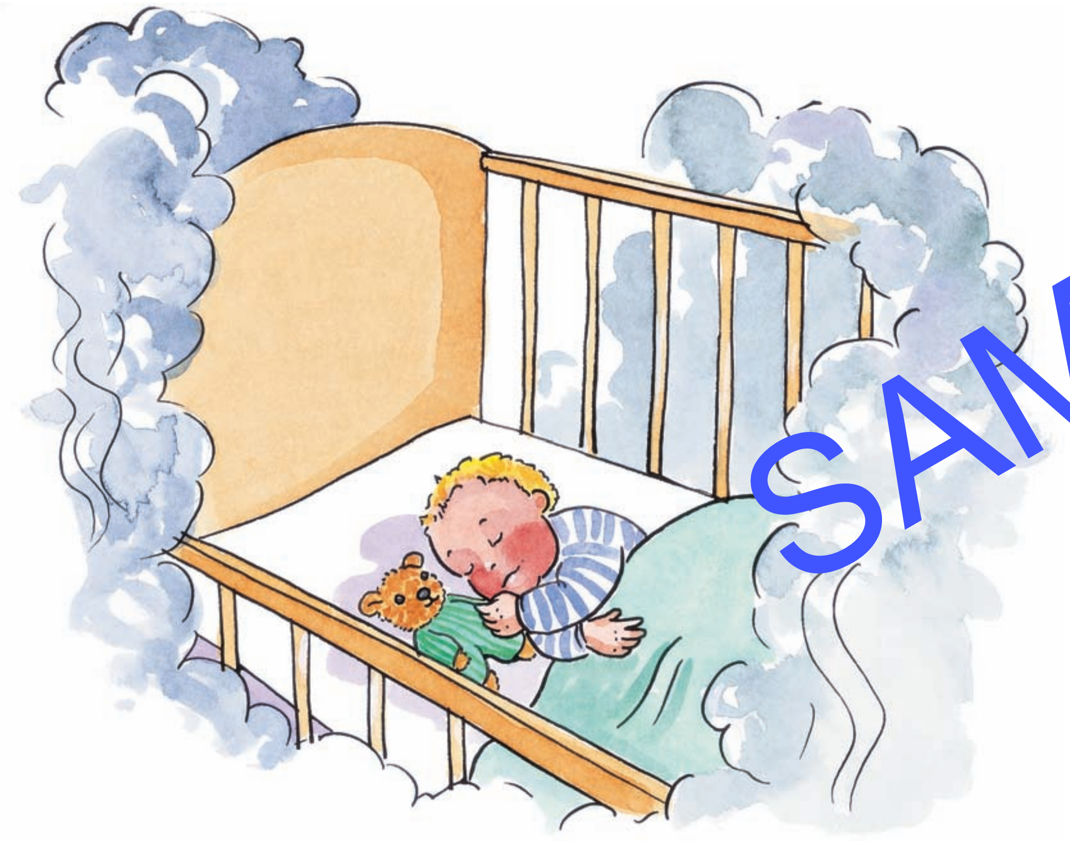

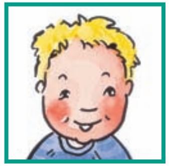

Get smoke alarms fitted to the ceilings
upstairs and downstairs in my home.

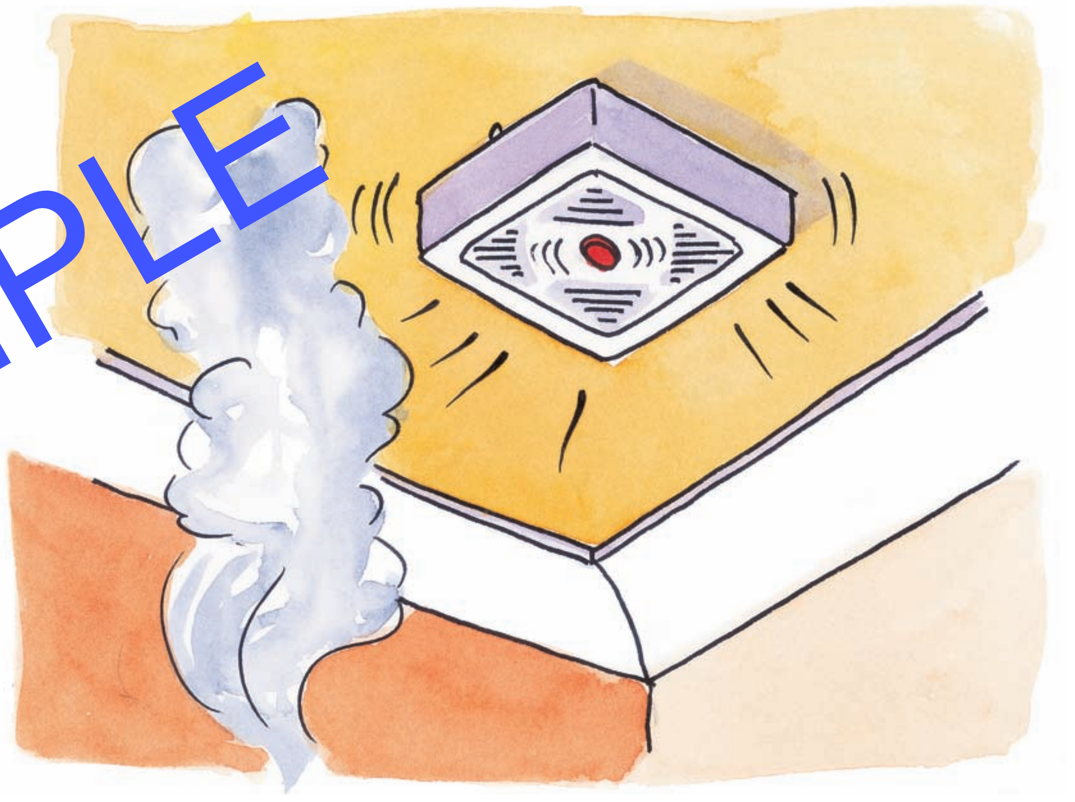

SAMPLE

My skin will burn very quickly...

I like to reach out and touch things.

It helps me learn about them.

But you need to take extra care of me around hot drinks and hot baths.

Make sure my skin doesn't get burnt...

SAMPLE

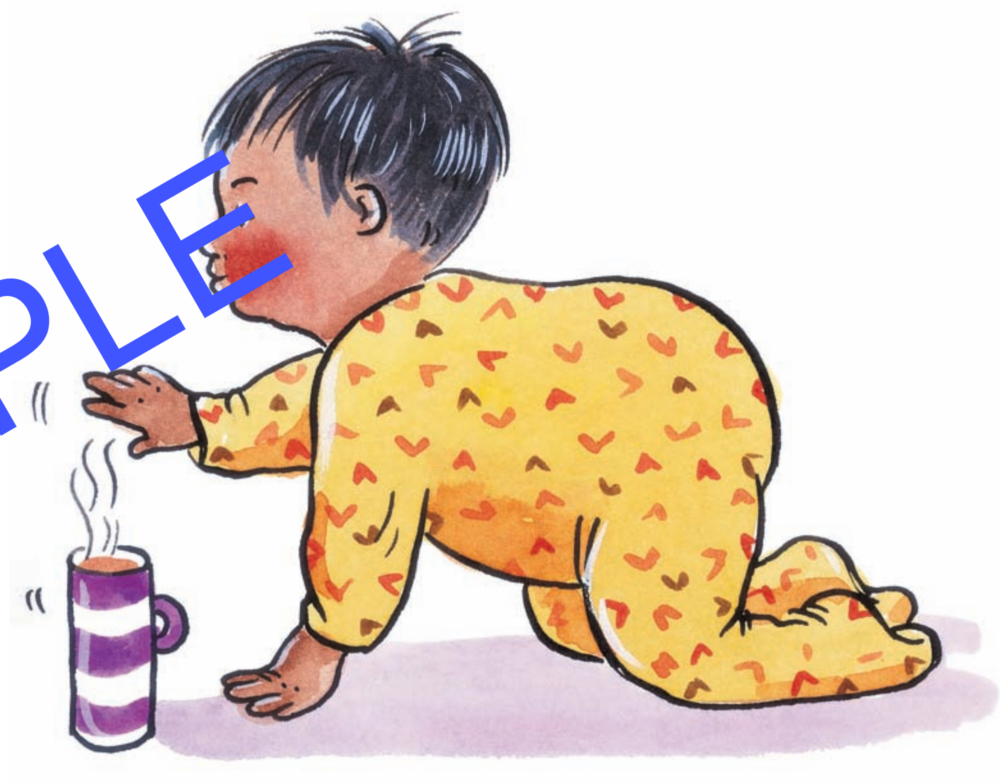

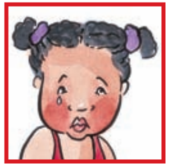

I might burn myself
on a fire or heater.

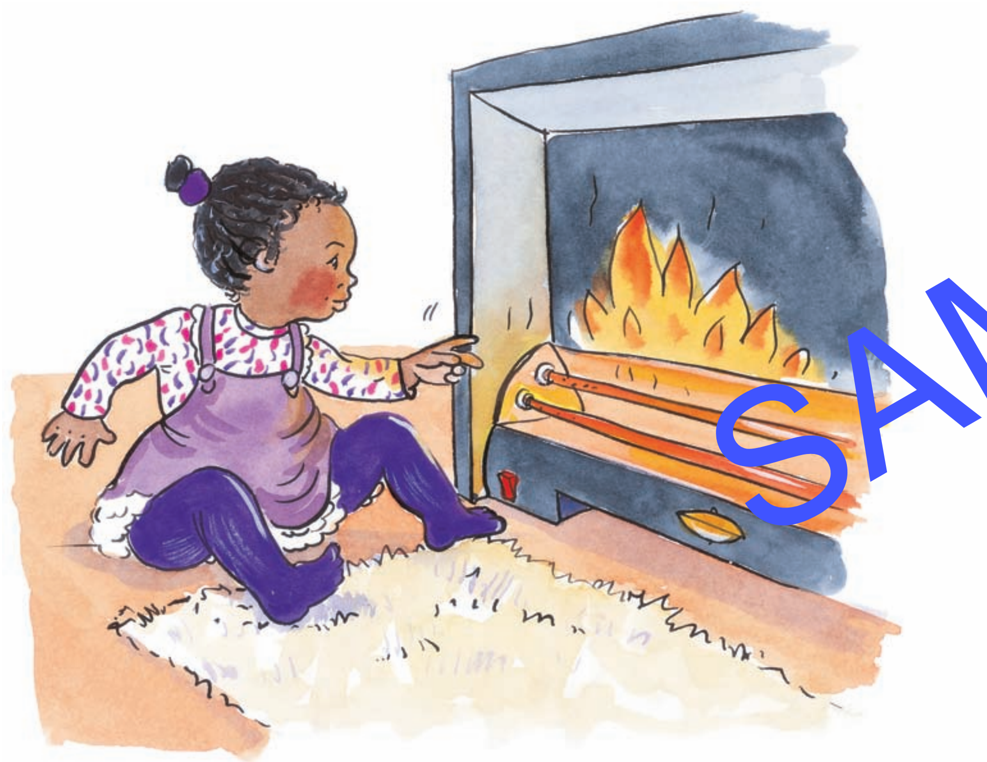

SAMPLE

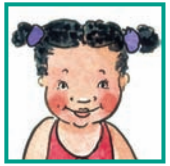

Put fireguards in front of fires
and heaters in my home.

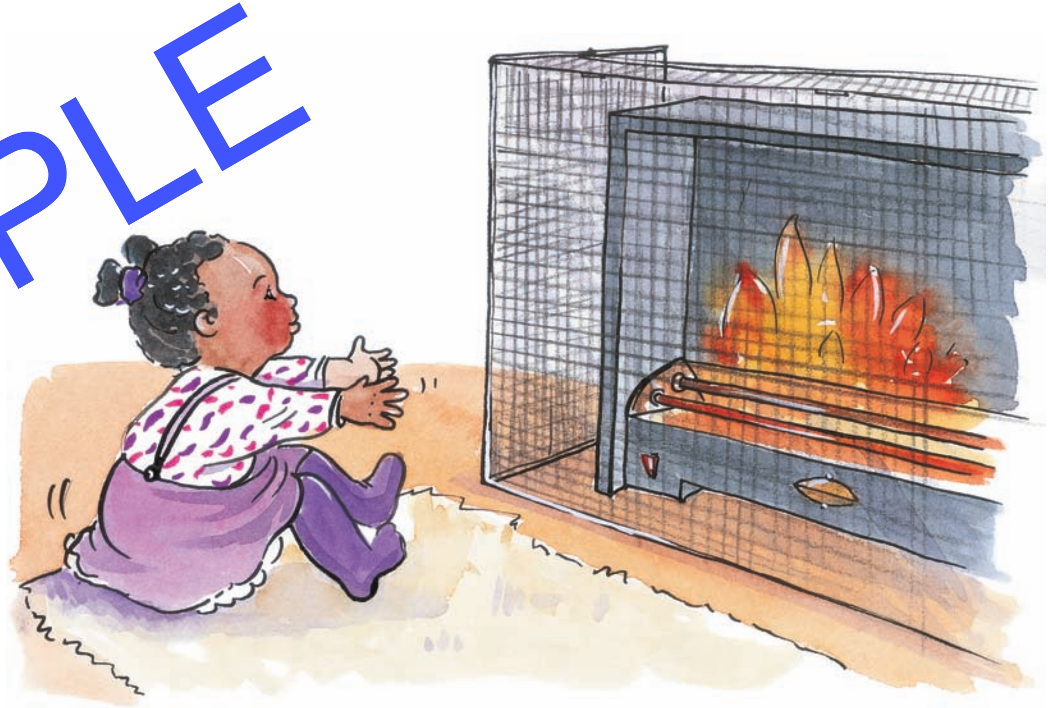

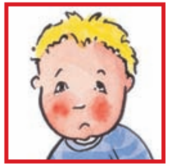

I might reach out for a mug
of hot drink.

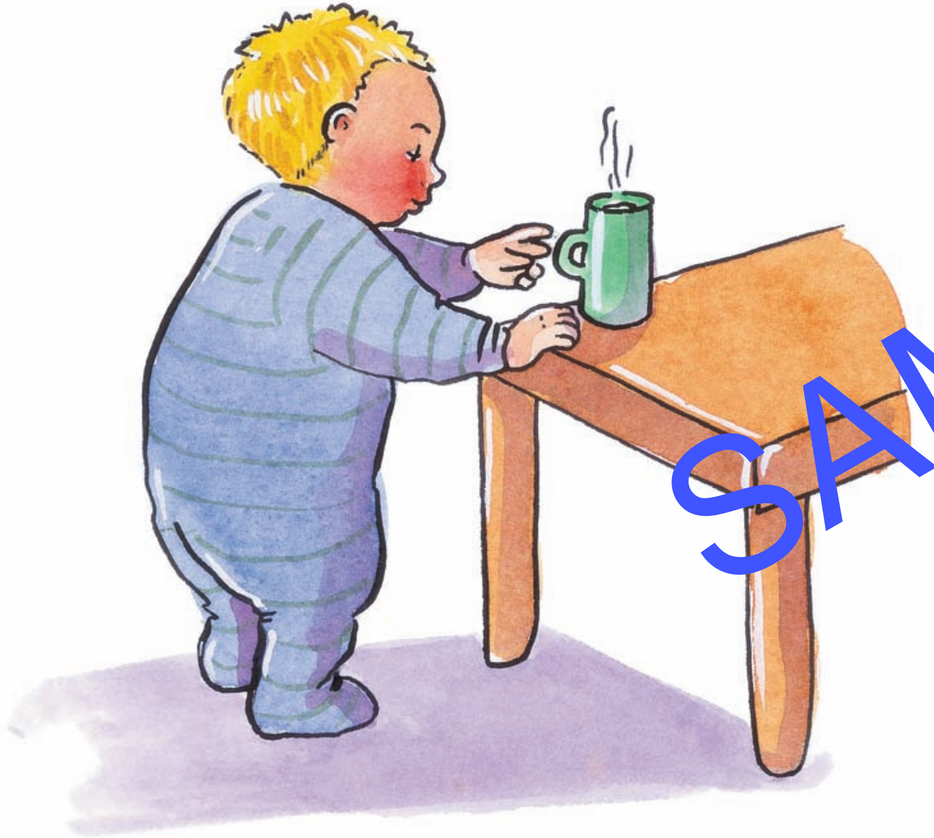

SAMPLE

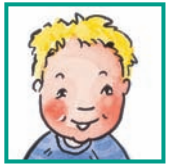

Put your hot drink
out of my reach.

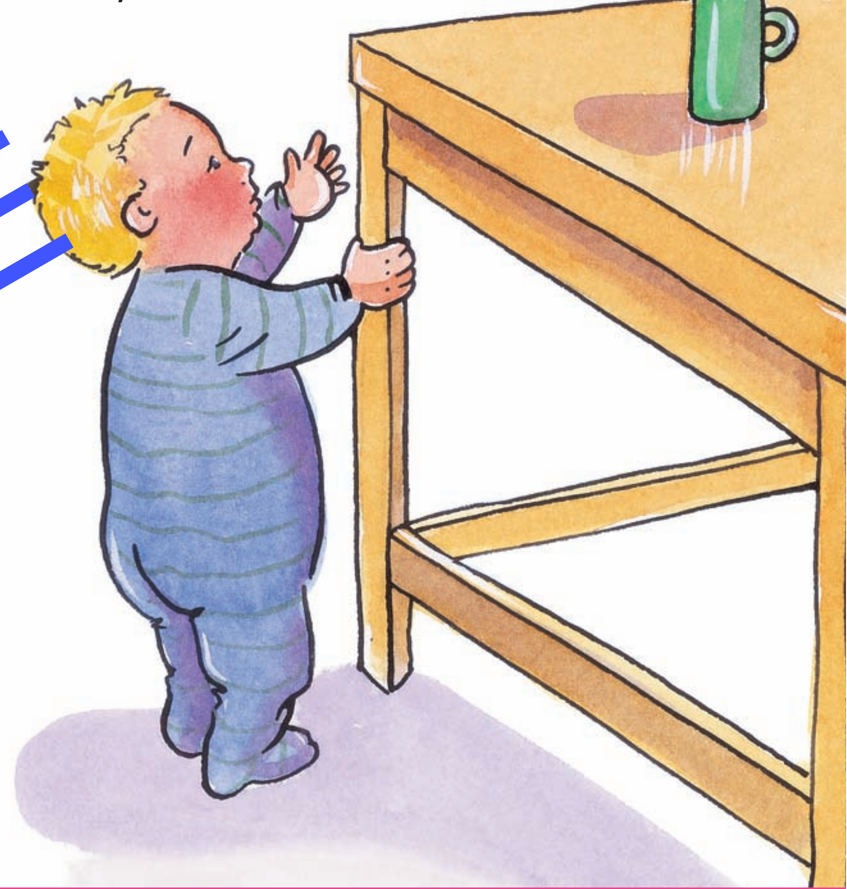

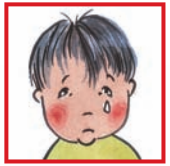

You might put too much hot water in my bath.

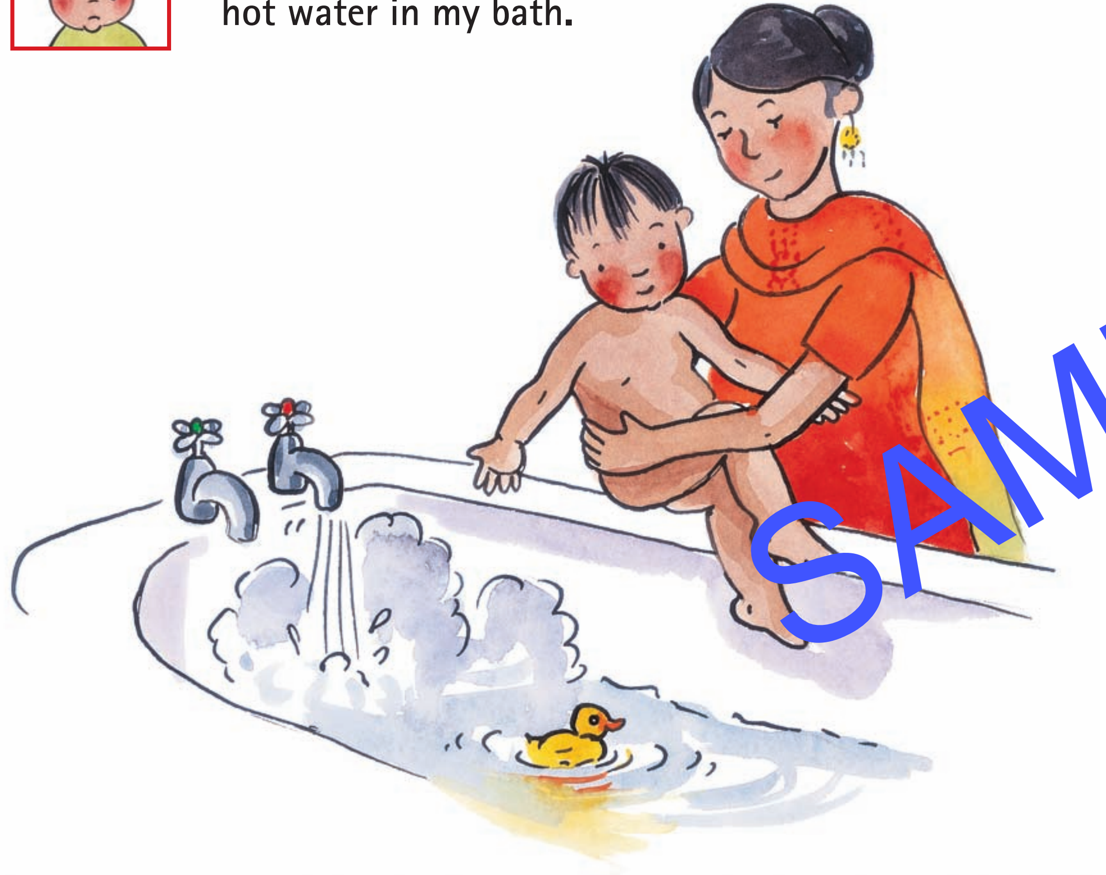

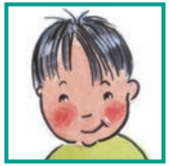

Test my bath water with your elbow before putting me in it. It should not feel hot or cold.

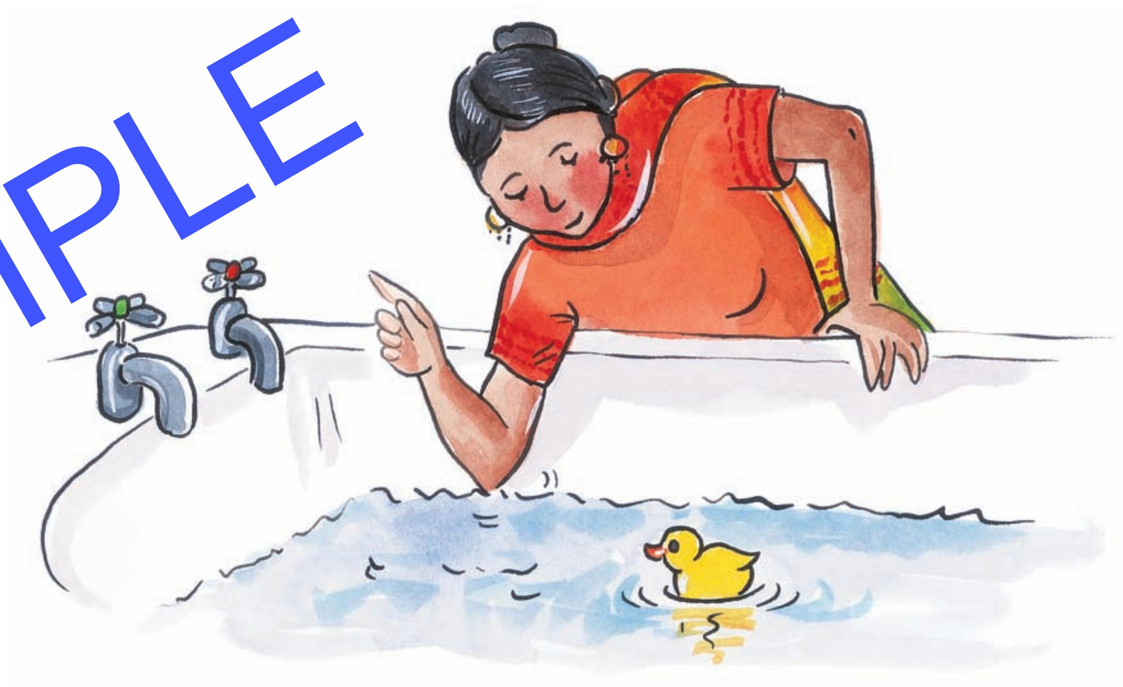

SAMPLE

I'm still too little to get myself out of trouble...

I like to play with things I find – small things, big things, anything.

Bath time is fun too but it's easy to slip over in the bath when I'm wet.

Make sure I don't get myself into trouble...

SAMPLE

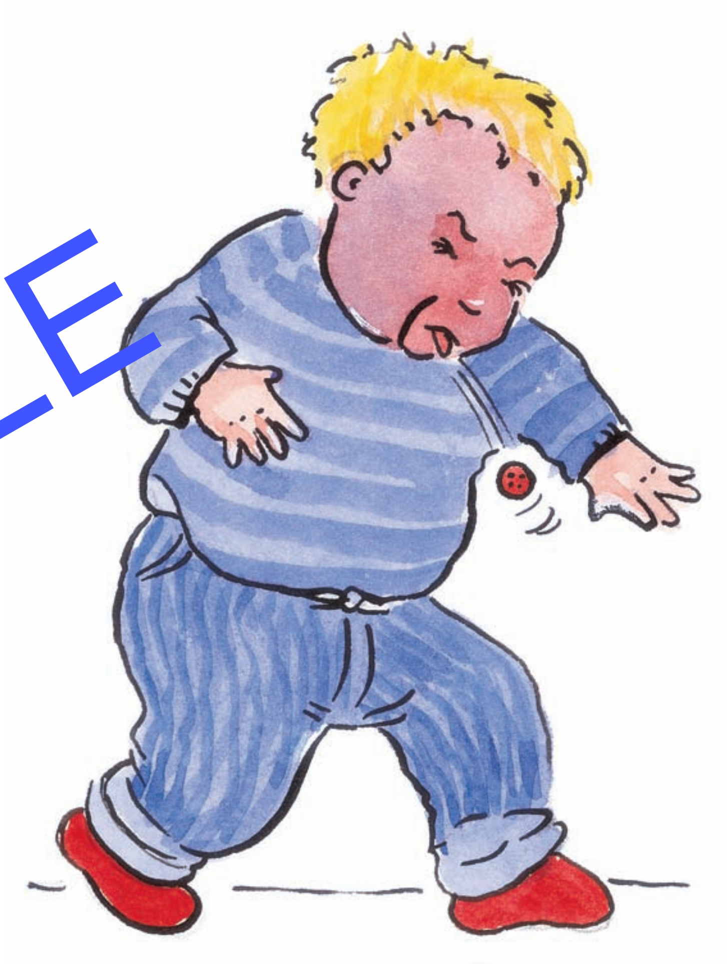

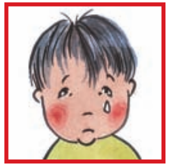

I can choke on small things I find
like coins or little toys.

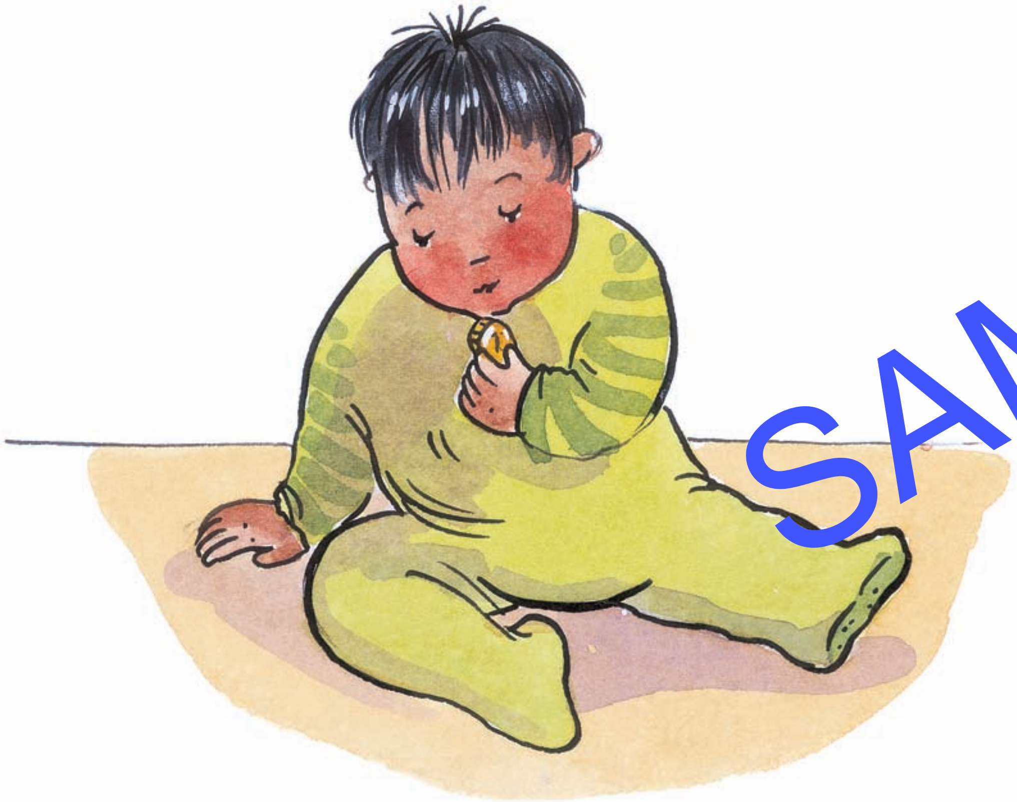

SAMPLE

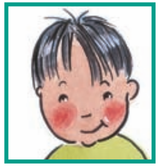

Put small things where
I can't find them.

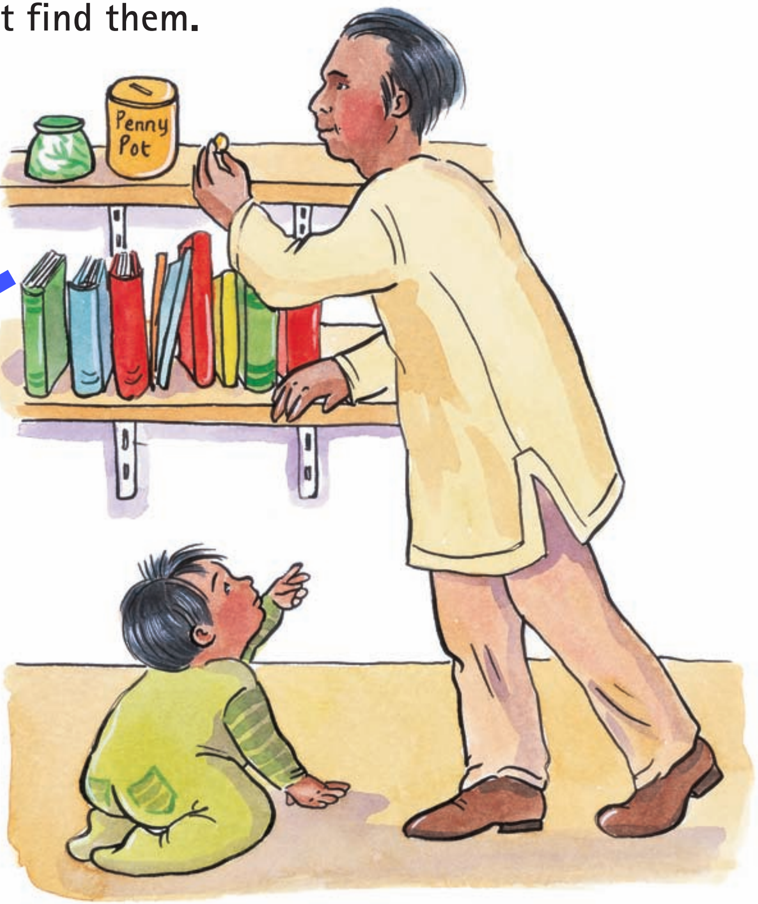

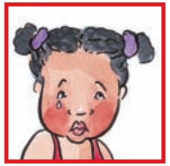

You might leave cleaning things on the floor.

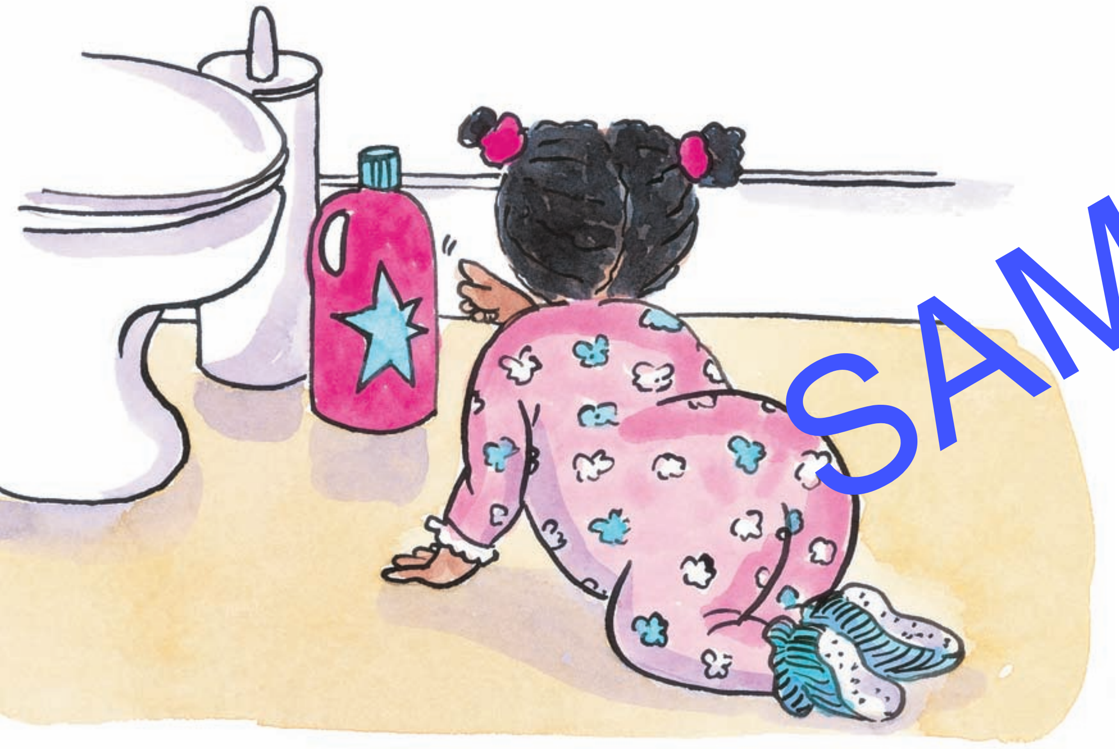

SAMPLE

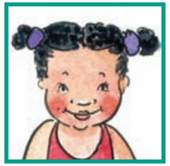

Keep all cleaning things out of my reach.

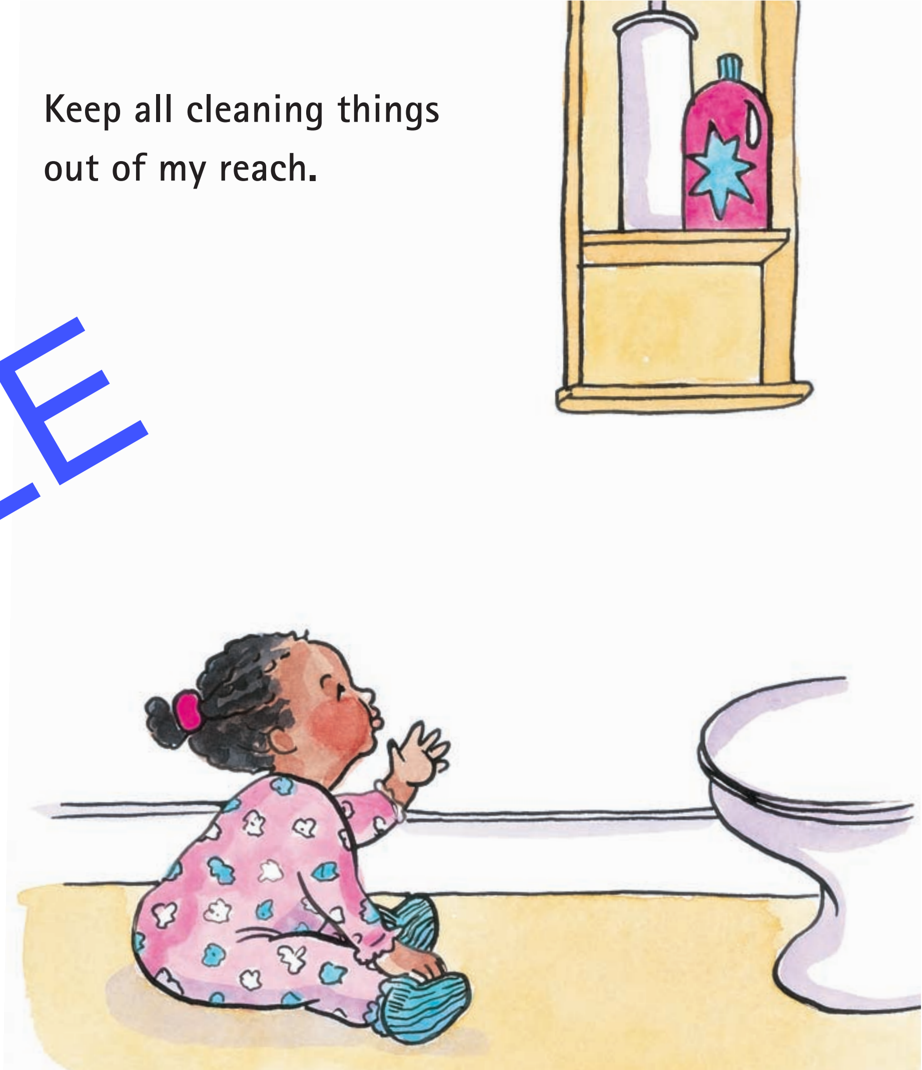

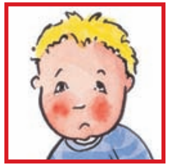

I could drown if you leave the room while I'm in the bath.

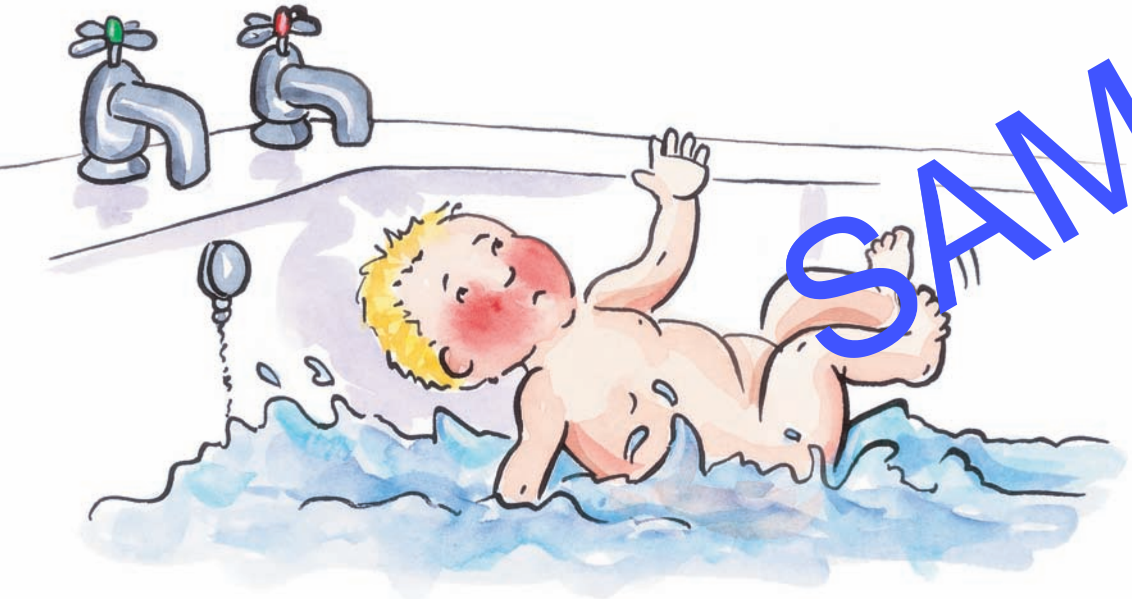

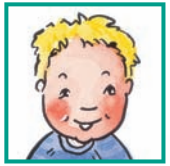

Don't leave the room while I'm in the bath – even for a second.

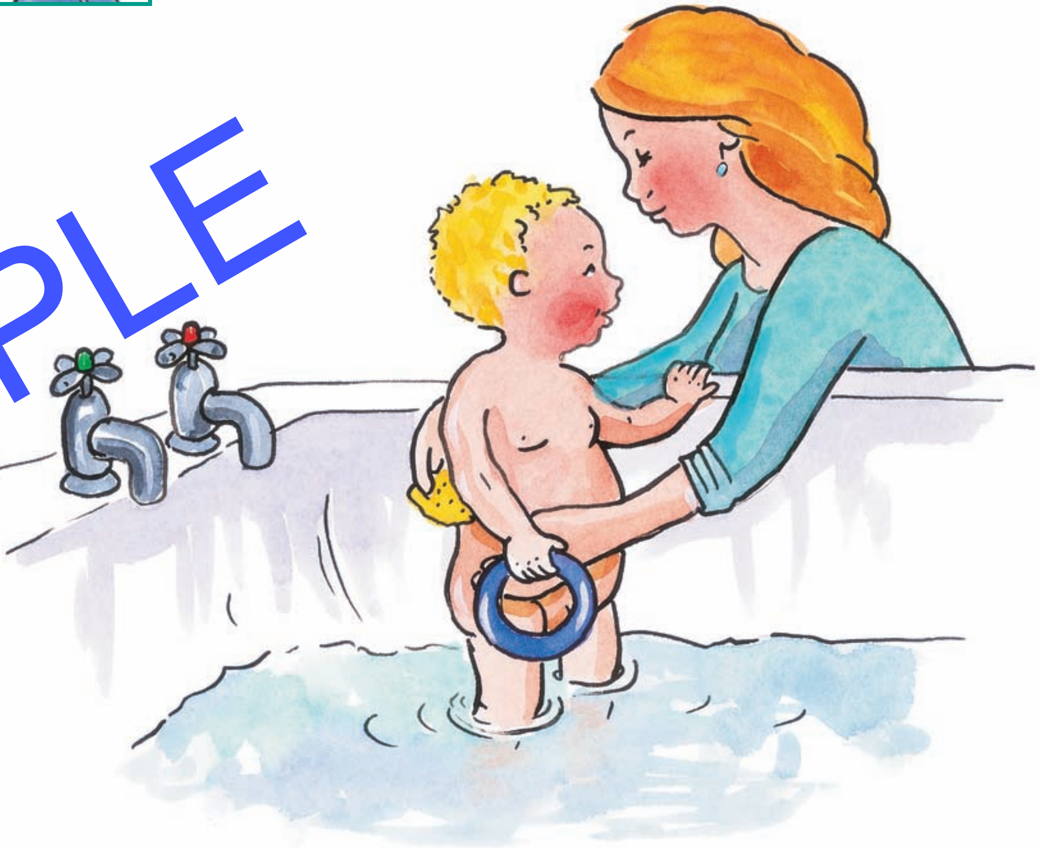

SAMPLE

I could get hurt in the car...

You might put me in the car to get my brother or sister from school.

Or to go to the shops.

Make sure I'm safe in the car...

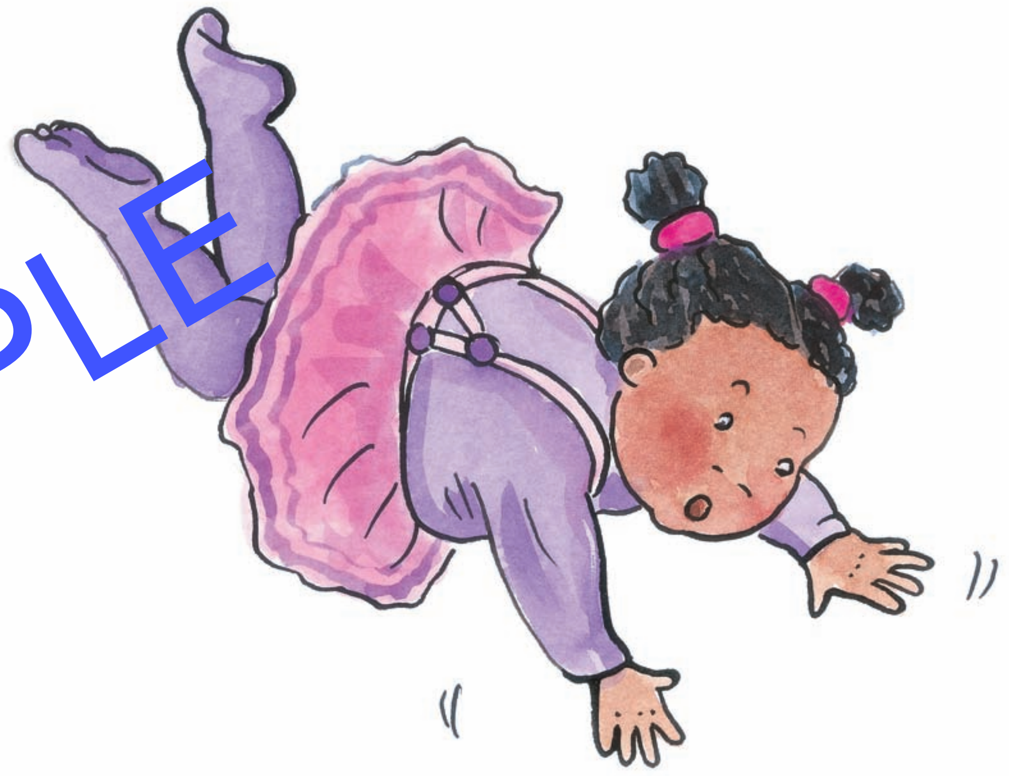

SAMPLE

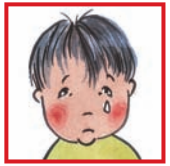

You might not use
a car seat.

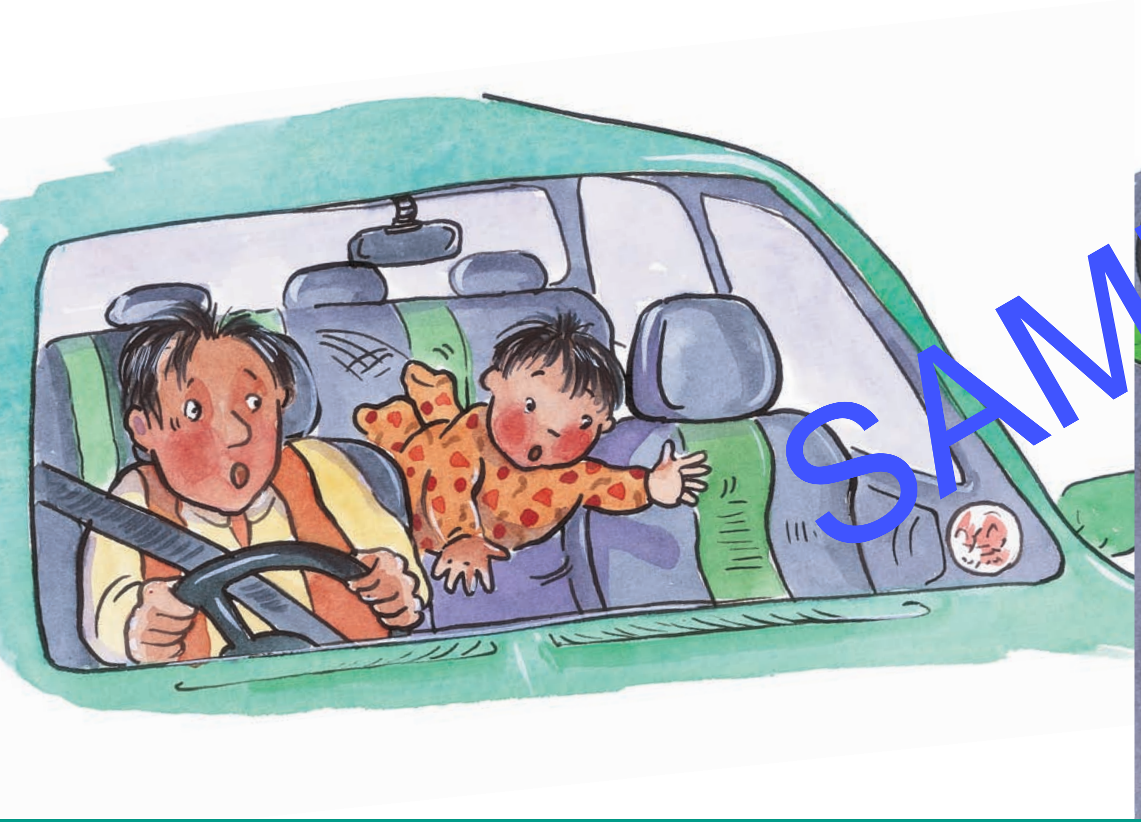

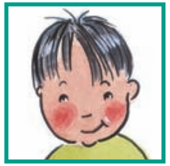

Use a car seat every time I go out in the car.
Fit my car seat properly using the seat belt
and do up all my straps.

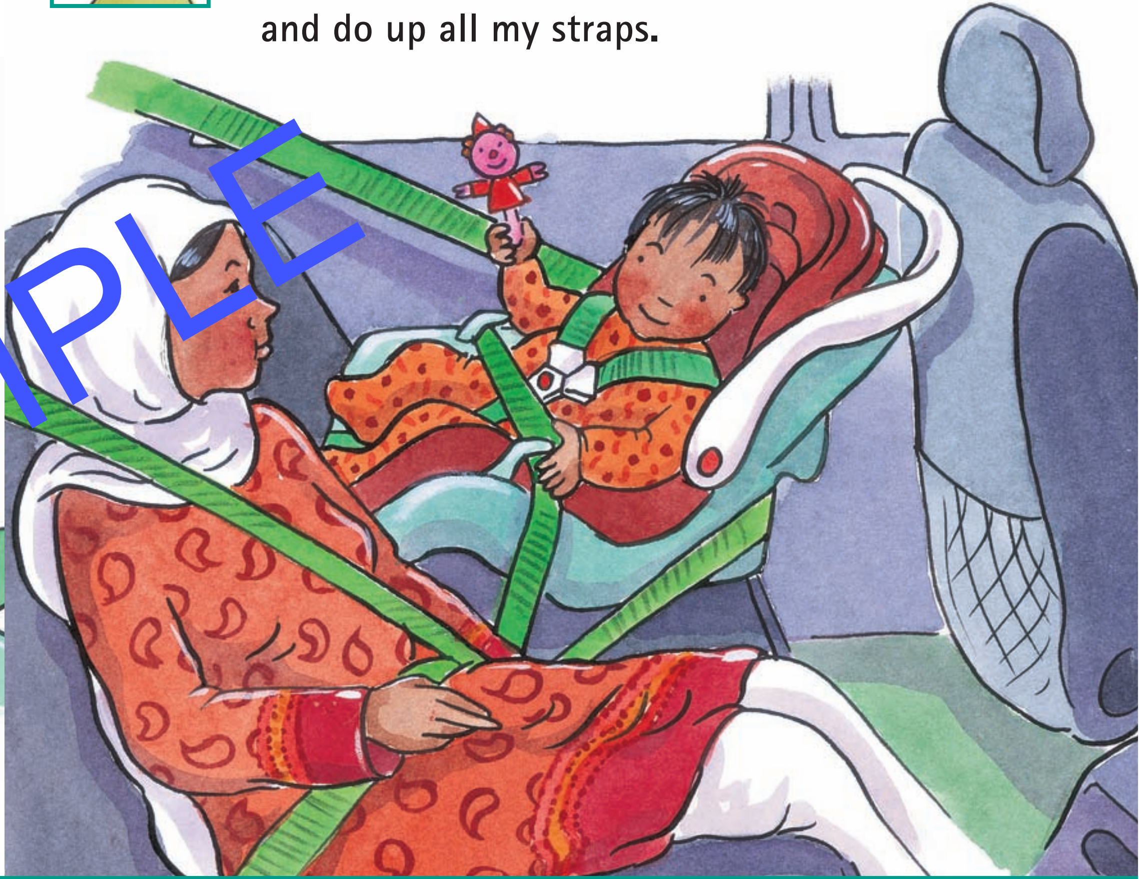

SAMPLE

Here's all my advice together

Falling

I could roll off a bed or table while you're changing my nappy	Change my nappy on the floor
I might try to go up or down the stairs on my own	Use safety gates at the top and bottom of the stairs
You might forget to strap me into my highchair	Use a harness with five straps like the one in the picture when I'm sitting in my highchair
You might not strap me into my pushchair when we go out	Use a harness with five straps like the one in the picture when [illegible]ou take me out in my pushchai[illegible]

Fire in my home

A hot pan could catch fire if you turn your back on it	Keep a close eye on hot pans like chip pans, frying pans and woks
You might fall asleep in the armchair and drop your cigarette	Stub your cigarette right out if you feel sleepy
You might not smell smoke until it's too late	Get smoke alarms fitted to the ceilings upstairs and downstairs in my home

Burns to my skin

I might burn myself on a fire or heater	Put fireguards in front of fires and heaters in my home
I might reach out for a mug of hot drink	Put your hot drink out of my reach
You m[illegible]ht put too much hot wat[illegible] my bath	Test my bath water with your elbow before putting me in it

Getting into trouble

I can choke on small things like coins or little toys	Put small things where I can't find them
You might leave cleaning things on the floor	Keep all cleaning things out of my reach
I could drown if you leave the room while I'm in the bath	Don't leave the room while I'm in the bath – even for a second

Riding in the car

You might not use a car seat	Use a car seat every time I go out in the car. Fit my car seat properly using the seat belt and do up all my straps.

SAMPLE
