# Supplementary material for: Using intervention mapping to develop a culturally appropriate intervention to prevent childhood obesity: the HAPPY (Healthy and Active Parenting Programme for Early Years) study
Source: Int J Behav Nutr Phys Act. 2013 Dec 28;10:142. doi: 10.1186/1479-5868-10-142 (PMC3895739; doi:10.1186/1479-5868-10-142)
Supplement: Additional file 4 — a. MASTER HAPPY Antenatal Manual FINAL VERSION. b. MASTER HAPPY Postnatal Manual FINAL VERSION. c. HAPPY manual resources. [file 1479-5868-10-142-S4.zip › 1523029418990916_add4c10.zip!/Weaning recipes and ideas handout.doc]

**CARROT AND POTATO PUREE**

Preparation:	25 Mins

Age range 6 months+ (first stage)

Approximately 12 – 14 servings

Suitable for freezing

Keeps for up to 24 hrs in the fridge

**Ingredients:**

1 medium potato – peeled and diced

1 medium carrot – peeled and diced

Babies usual milk to thin

**Method:**

- Put the water into a small pan and bring to the boil.
- Add the vegetables and cover. Bring back to the boil and simmer for 10 mins until tender.
- Puree the vegetables with the cooking water.
- Take out one portion to serve and thin with babies usual milk.

**TIP**

Any root vegetables can be used, (parsnips, swede, sweet potato). Or a mixture of root and green vegetables e.g. potato, cauliflower and broccoli.

**DAHL**

Preparation:	30 mins

Age range 6 months+ (first or second stage)

Make 6 portions

Suitable for freezing

Keeps for 24 hours in the fridge

**Ingredients:**

1 tablespoon vegetable oil

1 small onion, finely chopped

½ teaspoon turmeric

½ teaspoon ground coriander or cumin powder

125g (4oz) red lentils, washed and picked over 300ml (½ pint) water

**Method:**

- Heat the oil in a pan over a medium heat.
- Add the onion and fry gently until soft (about 5 mins).
- Mix the spices and cook for a further 2 mins.
- Add the lentils and water and bring to the boil.
- Reduce the heat and simmer for 25 mins, until lentils are soft and the dahl has a smooth consistency.

This can be served blended or mashed as appropriate for stage of weaning.

**FRESH PEACH YOGHURT**

Preparation:	10 mins

Age ranges 6 months+ (all stages)

Makes 2 – 4 servings

Keeps for up to 24 hours in the fridge

**Ingredients:**

1 ripe peach, peeled, stoned and diced

1 tablespoon water

4 tablespoons natural full fat yoghurt

**Method:**

- Place the peach and the water in a small saucepan, cover with a tight fitting lid and bring to the boil.
- Reduce the heat and simmer for about 5 mins until the fruit is tender.
- Puree the peach with the cooking water and allow to cool.
- Stir in the yoghurt and mix well.

**TIP**

Tinned fruit in juice can be used or mashed banana (no need to cook first).

Yoghurt can be served with mashed fruit or finely chopped for second stage.

**LAMB WITH SPINACH**

**AND POTATO**

Preparation:	40 mins

Age range 6 months+ (first or second stage)

Makes 6 – 10 servings

Suitable for freezing

Keeps for up to 24 hours in the fridge

**Ingredients:**

100 mls of water

85g (3½oz) lean lamb, diced

Half medium potato, peeled and diced

50g (2oz) spinach, washed thoroughly and chopped

Cool boiled water and thin

**Method**

Put the water into a small saucepan and bring to the boil. Add the lamb. Cover with a tight fitting lid, reduce heat and simmer for 5 mins. Remove the lid and add spinach, replace the lid when the spinach wilts. Continue simmering for about 15 mins until the meat is cooked right through and the vegetable are soft. Puree with the cooking water until smooth for the first stage or fork mash for second stage. Take out one portion to serve and add cooled, boiled water to thin, if necessary.

**TIP**

Swap lamb for chicken and spinach for any other vegetable. Add a pinch of mild spices e.g. coriander or a few mixed herbs if you want to.

**TUNA PASTA BAKE**

Preparation:	45 mins

Age range 9 months (second stage)

Makes 2-4 servings

Keeps for up to 2 days in the fridge

**Ingredients**:

85g (3½oz) pasta shapes

55g (2oz) tinned tuna, drained

55g (2oz) frozen peas, defrosted

1 egg

150ml (¼ pint) milk

4 tablespoons Cheddar cheese grated

**Method**:

- Preheat the oven to 190°C/375°F/Gas Mark 5
- Cook the pasta as directed on the packet
- Stir the peas and tuna into the pasta and place in a small, greased ovenproof dish
- Beat together the egg and milk and half of the Cheddar cheese and pour over the pasta
- Sprinkle the rest of the cheese on top and bake for 20 mins until golden brown
- Fork mash/chop and serve with an extra vegetable such as carrots

This can be served mashed or chopped as appropriate for stage of weaning.

**FINGER FOODS FROM 6 MONTHS**

- Cooked pieces/sticks of vegetable e.g. carrot, broccoli, potato, courgette
- Cooked pasta shapes
- Fingers of bread, toast or chappati
- Banana, ripe melon, peach

From about 9 months add

- Cubes of cheese
- Raw fruit and vegetable pieces

e.g. cucumber, carrot, apples, pears

**TOP TIPS for WEANING**

- Always wash your hands before preparing food
- Do not add salt to food for baby
- Expect Mess! –Cover the floor with newspaper or a plastic sheet, use a bib and try not to be anxious about mess whilst baby is learning to eat solids –relax and leave face and hand wiping and cleaning up until baby has finished
- Always stay with your baby when they are feeding
- Talk to your baby and make eye contact during their meal –relax and enjoy the fun!
- Turn the TV off (and other screens that may distract you !) during meals
- When your baby shows signs of having had enough, end the meal –and don’t force them to eat the rest
- Try and let your baby eat together with other family members as much as is practical.
