# Supplementary material for: Using intervention mapping to develop a culturally appropriate intervention to prevent childhood obesity: the HAPPY (Healthy and Active Parenting Programme for Early Years) study
Source: Int J Behav Nutr Phys Act. 2013 Dec 28;10:142. doi: 10.1186/1479-5868-10-142 (PMC3895739; doi:10.1186/1479-5868-10-142)
Supplement: Additional file 4 — a. MASTER HAPPY Antenatal Manual FINAL VERSION. b. MASTER HAPPY Postnatal Manual FINAL VERSION. c. HAPPY manual resources. [file 1479-5868-10-142-S4.zip › 1523029418990916_add4c11.zip!/Physical activity for new mums quick quiz handout.pdf]

# Quick Quiz
# Physical activity for new mums

HAPPY
Healthy and Active Parenting Programme for early Years
eat well, drink well, play well

## When can I start exercising?

There is one correct answer for each statement, chose the correct answer by ticking the box…

1. I can start my pelvic floor exercises…
   - a. Straight away ☐
   - b. After my 6 week postnatal check ☐
   - c. After 10 week ☐

2. I can start walking with the pram…
   - a. As soon as I feel ready ☐
   - b. After my 6 week postnatal check ☐
   - c. After 10 week ☐

3. If I have a normal pregnancy and birth I can do more strenuous exercise like going to my dance class…
   - a. As soon as I feel ready ☐
   - b. After my 6 week postnatal check ☐
   - c. After 10 weeks ☐

4. If I have a complicated pregnancy or caesarean delivery I can do more strenuous exercise like jogging…
   - a. As soon as I feel ready ☐
   - b. After my 6 week postnatal check ☐
   - c. After my 10 week postnatal check ☐

## What should I be aware of before exercising?

Below there are 6 statements, chose true or false for each statement…

| Statement | True | False |
|---|---|---|
| 1. Your joints will be more pliable so it is easier to injure yourself, be careful not to stretch or twist too much. | ☐ | ☐ |
| 2. If your joints are achy or you are feeling extremely tired after exercise you are doing too much | ☐ | ☐ |
| 3. If you bleed after exercise you are doing to much, you should visit your GP | ☐ | ☐ |
| 4. Don’t do exercise if you are breastfeeding because it effects the quality of your breast milk | ☐ | ☐ |
| 5. A sports bra can compress your breasts so if you are breastfeeding only wear it whilst you exercise | ☐ | ☐ |
| 6. Don’t exercise in the morning as your body will not be ready until after 12 noon | ☐ | ☐ |
