# Supplementary material for: Using intervention mapping to develop a culturally appropriate intervention to prevent childhood obesity: the HAPPY (Healthy and Active Parenting Programme for Early Years) study
Source: Int J Behav Nutr Phys Act. 2013 Dec 28;10:142. doi: 10.1186/1479-5868-10-142 (PMC3895739; doi:10.1186/1479-5868-10-142)
Supplement: Additional file 4 — a. MASTER HAPPY Antenatal Manual FINAL VERSION. b. MASTER HAPPY Postnatal Manual FINAL VERSION. c. HAPPY manual resources. [file 1479-5868-10-142-S4.zip › 1523029418990916_add4c11.zip!/HELPYO~1.PDF]

Being active is important for your child's health

Being physically active is important for young children because it helps them build and maintain a good level of health. It helps them develop movement skills, a strong heart, muscles and bones, keep to a healthy weight, social skills and much more. It is recommended that:

Babies

From playing on the floor to splashing in the pool, encourage your baby to be active from day one.

Toddlers

With lots of fun play from morning to bedtime, encourage your child to be active for at least 180 minutes (3 hours) every day.

Under fives

Your child should stay on the move and avoid sitting still for too long. Limit the time your child spends in a buggy, car seat or high-chair.

What types of physical activity are best for my child?

Lots of different activities can count towards your child's daily physical activity. Anything where they are moving counts. Make it as fun as possible!

Remember your child doesn't have to do their 180 minutes (3 hours) of activity all in one go. They can build it up during the day.

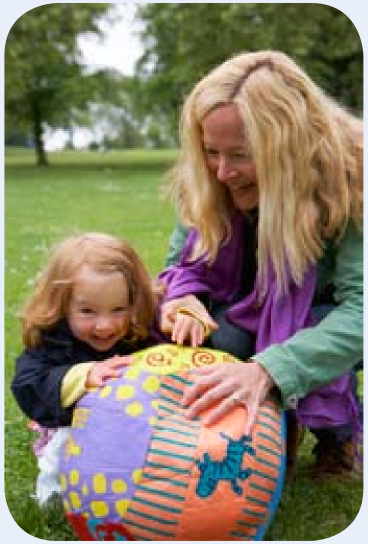

Don't forget healthy eating is important too

Children under five need regular meals and healthy snacks to make sure they get all the energy they need.

What should my child eat?

Meals should include a lot of different foods. Children should eat food from the four main food groups:

- bread, rice, potatoes, pasta and other starchy carbohydrates
- fruit and vegetables
- milk and dairy foods
- meat, fish, eggs and other vegetarian alternatives.

It's alright if they don't like it today...

Children's tastes will change from day to day. Just because they don't like something one day, doesn't mean they'll never like it. Keep giving them a wide range of foods, even ones they don't like.

Never force a child to eat, they will know best when they are full. If you think that they haven't eaten enough, consider if they could be full up on drink or how big their last snack was.

It's rare for a healthy child to refuse food if they are hungry, but if you are concerned then talk to your health visitor or doctor.

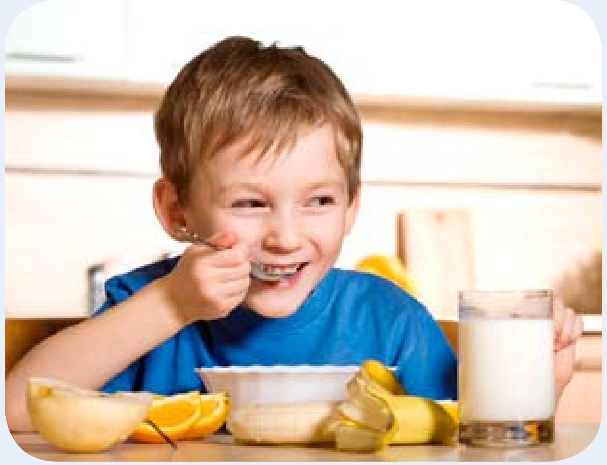

Published by BHF National Centre www.bhfactive.org.uk

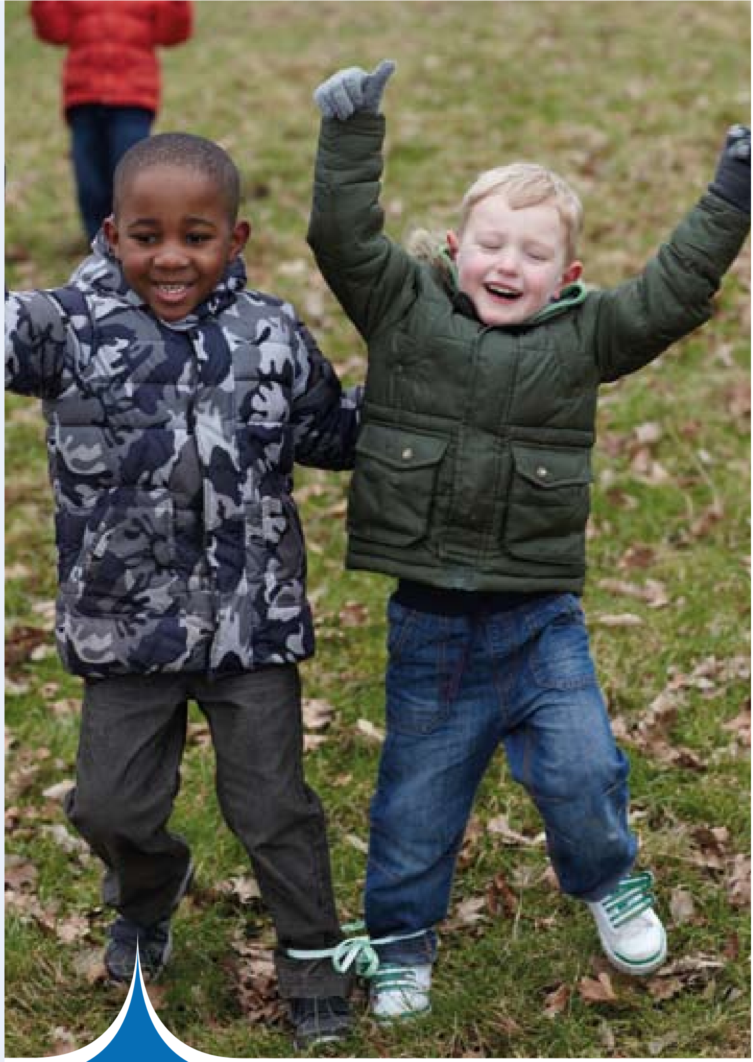

Help your child move and play every day

Information for parents

BHF National Centre physical activity+health

Funded by British Heart Foundation

Ideas for active play with your child

These are just a few examples. Try making up some games of your own and have fun!

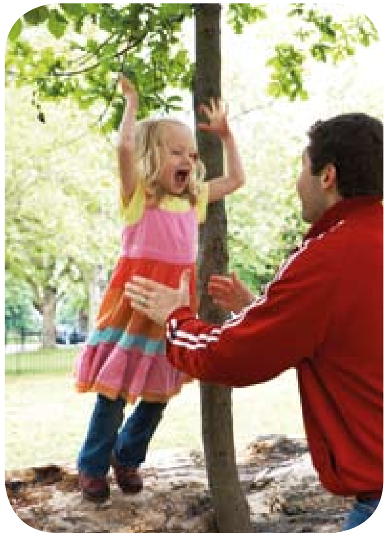

In the garden or park

- do some gardening together
- play with simple equipment such as balls, bats and boxes
- play chasing games like tig
- set up simple obstacle courses and have objects to run around, jump over or crawl through
- collect objects like twigs, leaves and pebbles
- set up a treasure hunt of objects to find
- kick or roll a ball gently to your child, ask them to stop it and kick or roll it back

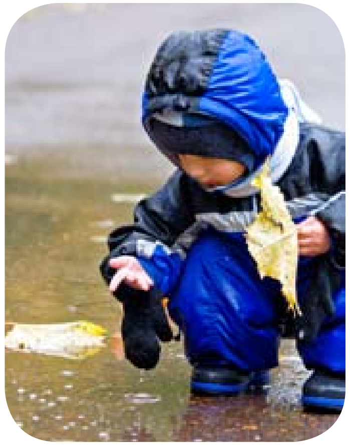

In the house

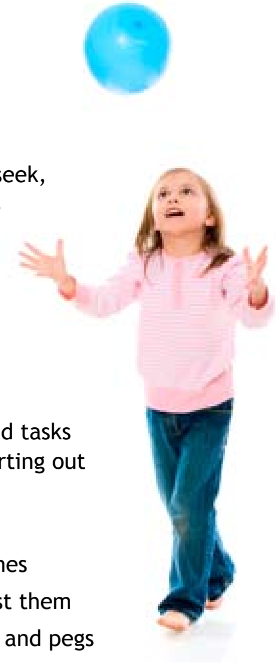

- play active games like hide and seek, follow the leader and Simon says
- blow up balloons and try to keep them in the air
- make tidying up into a game by choosing a colour, such as green, and then tidying everything that is that colour
- let your child help with household tasks like emptying the dishwasher, sorting out washing and unpacking shopping
- dance to music
- do action songs and nursery rhymes
- play with bubbles and try to burst them
- make a den out of chairs, sheets and pegs
- dress up and play act

What if my child's not interested?

- support them in their activities, showing them things they can copy
- give them lots of praise to improve their confidence
- focus on what they can do rather than what they can't
- be active together with your child

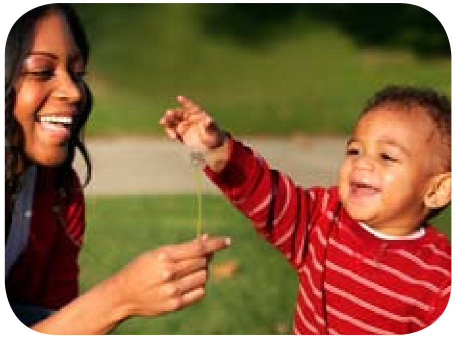

Tips for helping your child to be more physically active

- meet up with friends to go for a walk or meet at the local park
- set aside time to do something with all the family, you could play a game together or go for a walk
- get outdoors as much as you can and let your child explore
- children can play outside in all weather but they need the right clothing
- choose 'active' toys for birthday presents such as a ball, bat, scooter or kite
- include active games at birthday parties and when your child's friends come over to play
- keep a box of simple equipment in the boot of your car which you can use on breaks during long car journeys
- have one of your child's friends round to play, children may be more active with a playmate
- encourage your child to get out of the buggy and walk, starting with short distances and build up

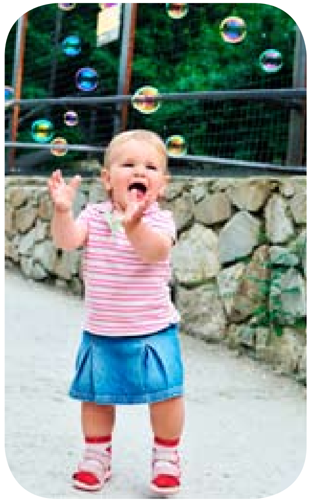

Reduce sitting time

There are times when your child will need to sit in a buggy, highchair or car seat. Try and reduce these times and break them up where you can.

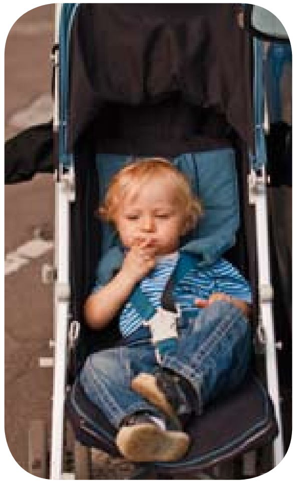

- allow extra time on long journeys and take regular breaks
- try not using the car for short trips
- try just using the highchair at mealtimes
- think about not leaving the TV on during mealtimes or when your child is playing
- your child will copy you so take care with how often they see you watching television or playing with the computer
